# Supplementary material for: A bacterial pan-genome makes gene essentiality strain-dependent and evolvable
Source: Nat Microbiol. 2022 Sep 12;7(10):1580–92. doi: 10.1038/s41564-022-01208-7 (PMC9519441; doi:10.1038/s41564-022-01208-7)
Supplement: Supplementary file 1 — Supplementary Table 1, SDMM composition, Supplementary data descriptive captions and Glossary of terms. [file 41564_2022_1208_MOESM1_ESM.pdf]

nature portfolio

https://doi.org/10.1038/s41564-022-01208-7

Supplementary information

A bacterial pan-genome makes gene essentiality strain-dependent and evolvable

In the format provided by the authors and unedited

Supplementary Table 1: Amino acid polymorphisms in SP_1751 across the PG-collection. Strains in red indicate SP_0185 essentiality. Residues polymorphisms in SP_1751 have the same color coding as in Figure 4a.

**Strain**<br>(SP_0185 is essential/non essential)	**SP_1751 allele**
TIGR4, PG18, PG28, PG27	**E57**, **N110**, **V160**
BHN97	**Y86**
Taiwan19F	**G102**
D39, PG04, PG29	**N110**
PG15, PG16	**V160**
All the others	G57, H86, E102, S110, A160

SDMM medium preparation and composition

	For **1 Liter** of SDMM add:	Of **stock** solutions (%)	Final concentration
**Acid hydrolyzed Casein**	5 grams		
**Enzyme hydrolyzed Casein**	1 gram		
**K_2HPO_4**	8.5 grams		49 mM
**NaOAc**	2 grams		24 mM
**$NaHCO_3$**	0.4 grams		5 mM
**$MgCl_2.6H_2O$**	0.5 grams		2.5 mM
**Yeast Extract**	0.5 grams		254 μM
**L-cysteine.HCL**	4 mL	1	253.79 μM
**L-tryptophan**	1.05 mL	0.57	32.14 μM
**L-asparagine**	10 mL	0.5	438.20 μM
**L-glutamine**	10 mL	0.1	78.05 μM
**Adenine**	31.25 mL	0.016	37.00 μM
**Ca-pantothenate**	1.2 mL	0.1	5.47 μM
**Nicotinic Acid**	1 mL	0.03	2.44 μM
**Pyridoxine.HCL**	0.428 mL	0.07	1.46 μM
**Thiamine.HCL**	0.33 mL	0.09	880.52 nM
**Riboflavine**	7 mL	0.002	371.98 nM
**Biotin**	0.017 mL	0.0035	2.44 nM
**$CaCl_2$**	1 mL	0.6	54.06 μM
**$CuSO_4.5H_2O$**	0.5 mL	0.1	2.00 μM
**$ZnSO_4.7H_2O$**	0.16 mL	0.3	1.67 μM
**$MnSO_4.4H_2O$**	0.286 mL	0.07	1.33 μM
pH to 7.3 using 6N HCL			
Filter sterilizing			
Storage at 4°C protected from light			
**Fresh same-day media**			
**Choline.Cl**	1.6 mL	0.3% fresh stock	34.38 μM
**$FeSO_4.7H_2O$**	1.25 mL	0.04% fresh stock	1.80 μM
**Catalase**			30 units/mL
**Oxyrase**	5 mL		
**Glucose**	20 mL	1 M	20 mM

Supplementary Data 1: *S. pneumoniae* Pan-Genome collection.

Supplementary Data 2: Average Nucleotide Identity Values between *S. pneumoniae* Pan-Genome collection.

Supplementary Data 3: Pan-genome 208 strains study group data.

Supplementary Data 4: Pan-genome study group strains genes with BF-Clust method clusters IDs.

Supplementary Data 5: Strains from the Pan-Genome study group with genome sequences as single closed contigs with BF-Clust clusters subdivided by genomic neighborhood.

Supplementary Data 6: Binomial gene essentiality calls and RNA-Seq results for the Pan-Genome collection strains.

Supplementary Data 7: Gene cluster essentialome classification.

Supplementary Data 8: Functional categories enrichment analyses data.

Supplementary Data 9: Tn-Seq fitness of non-essential genes in SDMM.

Supplementary Data 10: WGS breseq summary results of the different clones and populations sequenced.

Supplementary Data 11: Genetic interactions identified in this study.

Supplementary Data 12: Primers used in this study.

Supplementary Data 13: Set of knockouts constructed and characterized in this study.

Glossary of terms.

Pan-genome: The sum of all genes present in the 208 sequenced genomes used in this study.

Core genome: Set of the genes from the pan-genome that are present in all, or almost all of the 208 genomes.

Accessory genome: Set of the genes from the pan-genome that are present in not all of the 208 genomes.

Cluster: Group of genes from the pan-genome identified as orthologs by BF-Clust method[1].

Subcluster: Cluster of ortholog genes with the same syntheny.

Cluster diameter: Metrics obtained from BF-Clust method that represents the sequence diversity of a cluster.

Binomial z-value: Statistical value calculated by the TRANSIT Binomial method[2,3] to determine gene essentiality from Tn-Seq experiments.

Essentialome: Set of genes from the pan-genome identified as essential in at least one of the 17 strains with library saturation higher than 35%.

Universal essentials: Set of genes from the essentialome that belong to the core genome and were identified as essential in all the 17 strains with library saturation higher than 35%.

Core strain-dependent essentials: Set of genes from the essentialome that belong to the core genome and were identified as essential in some, but not all of the 17 strains with library saturation higher than 35%.

Accessory essentials: Set of genes from the essentialome that belong to the accessory genome and were identified as essential when present in any of the 17 strains with library saturation higher than 35%.

Merodiploid: Clone obtained after site-directed mutagenesis that conserves both the targeted gene and the selection marker used[4].

References

1 Surujon, D., Bento, J. & van Opijnen, T. Boundary-Forest Clustering: Large-Scale Consensus Clustering of Biological Sequences. bioRxiv, 2020.2004.2028.065870, doi:10.1101/2020.04.28.065870 (2020).

2 DeJesus, M. A., Ambadipudi, C., Baker, R., Sassetti, C. & Ioerger, T. R. TRANSIT--A Software Tool for Himar1 TnSeq Analysis. PLoS Comput Biol 11, e1004401, doi:10.1371/journal.pcbi.1004401 (2015).

3 DeJesus, M. A. & Ioerger, T. R. Capturing Uncertainty by Modeling Local Transposon Insertion Frequencies Improves Discrimination of Essential Genes. IEEE/ACM Trans Comput Biol Bioinform 12, 92-102, doi:10.1109/TCBB.2014.2326857 (2015).

4 Johnston, C. et al. Natural genetic transformation generates a population of merodiploids in Streptococcus pneumoniae. PLoS Genet 9, e1003819, doi:10.1371/journal.pgen.1003819 (2013).
