# Supplementary material for: A bacterial pan-genome makes gene essentiality strain-dependent and evolvable
Source: Nat Microbiol. 2022 Sep 12;7(10):1580–92. doi: 10.1038/s41564-022-01208-7 (PMC9519441; doi:10.1038/s41564-022-01208-7)
Supplement: Supplementary file 2 — Reporting Summary [file 41564_2022_1208_MOESM2_ESM.pdf]

nature portfolio

Corresponding author(s): Tim van Opijnen

Last updated by author(s): Jul 12, 2022

Reporting Summary

Nature Portfolio wishes to improve the reproducibility of the work that we publish. This form provides structure for consistency and transparency in reporting. For further information on Nature Portfolio policies, see our Editorial Policies and the Editorial Policy Checklist.

Statistics

For all statistical analyses, confirm that the following items are present in the figure legend, table legend, main text, or Methods section.

n/a	Confirmed	
☐	☒	The exact sample size (*n*) for each experimental group/condition, given as a discrete number and unit of measurement
☐	☒	A statement on whether measurements were taken from distinct samples or whether the same sample was measured repeatedly
☐	☒	The statistical test(s) used AND whether they are one- or two-sided<br>*Only common tests should be described solely by name; describe more complex techniques in the Methods section.*
☒	☐	A description of all covariates tested
☐	☒	A description of any assumptions or corrections, such as tests of normality and adjustment for multiple comparisons
☐	☒	A full description of the statistical parameters including central tendency (e.g. means) or other basic estimates (e.g. regression coefficient) AND variation (e.g. standard deviation) or associated estimates of uncertainty (e.g. confidence intervals)
☐	☒	For null hypothesis testing, the test statistic (e.g. *F*, *t*, *r*) with confidence intervals, effect sizes, degrees of freedom and *P* value noted<br>*Give P values as exact values whenever suitable.*
☒	☐	For Bayesian analysis, information on the choice of priors and Markov chain Monte Carlo settings
☒	☐	For hierarchical and complex designs, identification of the appropriate level for tests and full reporting of outcomes
☒	☐	Estimates of effect sizes (e.g. Cohen's *d*, Pearson's *r*), indicating how they were calculated

Our web collection on statistics for biologists contains articles on many of the points above.

Software and code

Policy information about availability of computer code

Data collection	Tn-Seq, RNA-seq and WGS experiments data was obtained using the Nextera Illumina platform and analyzed using the aerobio pipeline (https://github.com/jsa-aerial/aerobio).
Data analysis	For analysis of the data we used Aerobio 2.3.0 (https://github.com/jsa-aerial/aerobio), R Studio 1.4.1106, Chimera.X.1.2.5, GraphPad 9, SMRTAnalysis pipeline version 8.0.0.80529. PanX analysis results, MAP files prepping for TRANSIT analysis, and Tn-Seq statistical testing codes can be found in the fork repositories at https://github.com/frosconi.

For manuscripts utilizing custom algorithms or software that are central to the research but not yet described in published literature, software must be made available to editors and reviewers. We strongly encourage code deposition in a community repository (e.g. GitHub). See the Nature Portfolio guidelines for submitting code & software for further information.

Data

Policy information about availability of data

All manuscripts must include a data availability statement. This statement should provide the following information, where applicable:

- Accession codes, unique identifiers, or web links for publicly available datasets
- A description of any restrictions on data availability
- For clinical datasets or third party data, please ensure that the statement adheres to our policy

Genome assemblies, assembly information, Tn-Seq, RNA-Seq and WGS raw data are available as part of the Bioproject PRJNA514780 (https://www.ncbi.nlm.nih.gov/bioproject/PRJNA514780). Accesion codes for GenBank biosamples (SAM###) are indicated in Supplementary Data 1.

Field-specific reporting

Please select the one below that is the best fit for your research. If you are not sure, read the appropriate sections before making your selection.

☒ Life sciences ☐ Behavioural & social sciences ☐ Ecological, evolutionary & environmental sciences

For a reference copy of the document with all sections, see nature.com/documents/nr-reporting-summary-flat.pdf

Life sciences study design

All studies must disclose on these points even when the disclosure is negative.

Sample size	The primary dataset of this study (208 strains) was chosen based on geography and date of isolation. The 36 strains of the pan-genome collection were selected based on phylogeny diversity. This collection covers 68% of the pan-genome, and is a number suitable to handle for bench experiments. Sample sizes for Tn-Seq consist of 6 independent libraries for each strain with 10,000 to 20,000 mutants per library. Tn-Seq power increases if different libraries with different mutants frequencies are used. From our previous works, we know 6 libraries are sufficient to obtain confident Tn-Seq results. Sample sizes of 3 were performed for growth assays and all other experiments. The high reproducibility observed among the different replicates proved the number of replicates to be sufficient.
Data exclusions	For determination of the essentialome by comparison of the different strains Tn-Seq results, libraries with less than 35% of saturation were excluded. For TPM normalization of RNAseq results, genes with 0 feature counts in at least one replicate were excluded. Genes without assigned clusters (mostly mobile elements, pseudogenes) are not part of the gene clusters analysis.
Replication	For Tn-Seq six independent libraries were constructed for each strain. RNA-seq experiments consisted of three independent replicates. Knockout validations consisted in three transformation experiments performed in four different strains. Growth curves were repeated at least three times with three replicates each. All attempts at replication were successful.
Randomization	Not applicable since samples were not allocated to experimental groups.
Blinding	Not applicable since samples were not allocated to experimental groups.

Reporting for specific materials, systems and methods

We require information from authors about some types of materials, experimental systems and methods used in many studies. Here, indicate whether each material, system or method listed is relevant to your study. If you are not sure if a list item applies to your research, read the appropriate section before selecting a response.

Materials & experimental systems

n/a	Involved in the study	
☒	☐	Antibodies
☒	☐	Eukaryotic cell lines
☒	☐	Palaeontology and archaeology
☒	☐	Animals and other organisms
☒	☐	Human research participants
☒	☐	Clinical data
☒	☐	Dual use research of concern

Methods

n/a	Involved in the study	
☒	☐	ChIP-seq
☒	☐	Flow cytometry
☒	☐	MRI-based neuroimaging
