# Supplementary material for: A bacterial pan-genome makes gene essentiality strain-dependent and evolvable
Source: Nat Microbiol. 2022 Sep 12;7(10):1580–92. doi: 10.1038/s41564-022-01208-7 (PMC9519441; doi:10.1038/s41564-022-01208-7)
Supplement: Source Data Fig. 4 — Swiss models data, genetic interactions (a subset of Supplementary Data 11), growth curves data and RNA-seq data for the two transporters (taken from Supplementary Data 6). [file 41564_2022_1208_MOESM8_ESM.zip › Fig_4a_models/SP_1751_swissmodel/SP_1751/templates.html]

Untitled Project | Templates


SWISS-MODEL

### Untitled Project

### Created: May 16, 2021, 3:29 a.m. at 03:29

- Templates
- Models

Models | Name | Description | GMQE | QSQE | Seq Id | Coverage | Range | Method | Resolution | Oligo-state | Ligands | Found by | Seq Similarity ||  | 4ev6.1.A | Magnesium transport protein CorA  *The complete structure of CorA magnesium transporter from Methanocaldococcus jannaschii* | 0.69 | 0.49 | 20.07 | 0.94 | 7-297 | X-ray | 3.20 | homo-pentamer | 8 x UMQ, 28 x MG | HHblits | 0.31 |
| ``` target    MVLEKQLGNGCTWIDLDLGKLNKLEDLSEIYGLDKETIEYALDRNERAHMDYHRESETVTFIYNVLDVKKDKAYYETFPM 4ev6.1    ------EDYRLIWIDCYDPKDEELYKLSKKIGISVSDLQIGLDEQEIPRVEEDEDF--YLIIYKAPLFE---EDITTTSL  target    TFIVEHRRLITISNTKNAYVIEQMTRYLENHD-----TLSIYKFLFASLEIISNAYYPVIEQMDKSRDEVNDLLRQRTTK 4ev6.1    GIYIKNNLLLTIHSDKIK-AIGRLHKLISTKKPRIVFERGIGFLLYHILNEITRSYSRILMNLEDELEELEDKLLAGYDR  target    KNLFVLSDLETGMVYLTAAAKQNRILLEHIQGHALYRSFDEIEREQFDDAMIEAHQLVSMTDLISQILQQLSASYNNILN 4ev6.1    EVMEKILGLRKTLVYFHKSLIANRDVLVLLKRKY-LPITTKEDRENFEDLYYDTLQLIDMSATYREVLTSMMDITLSLEN  target    NNLNDNLTTLTIISVLLAVLAVVTGFFGMNVP-LPLTDEPHAWLYISLASAGLWIVLSLLLRKIAKKS 4ev6.1    IKMNQIMKILTMVTTIFAVPMWITGIYGMNFSYLPLANNPQGFWLVMALMVVIIMIFVYIFRR----- ``` | | | | | | | | | | | | | | | | | | | | | | | | | | | | | | | | | | | | | | | | | | | | | | | | | |
|  | 4ev6.1.B | Magnesium transport protein CorA  *The complete structure of CorA magnesium transporter from Methanocaldococcus jannaschii* | 0.68 | 0.49 | 20.07 | 0.94 | 7-297 | X-ray | 3.20 | homo-pentamer | 8 x UMQ, 28 x MG | HHblits | 0.31 |
| ``` target    MVLEKQLGNGCTWIDLDLGKLNKLEDLSEIYGLDKETIEYALDRNERAHMDYHRESETVTFIYNVLDVKKDKAYYETFPM 4ev6.1    ------EDYRLIWIDCYDPKDEELYKLSKKIGISVSDLQIGLDEQEIPRVEEDEDF--YLIIYKAPLFE---EDITTTSL  target    TFIVEHRRLITISNTKNAYVIEQMTRYLENHD-----TLSIYKFLFASLEIISNAYYPVIEQMDKSRDEVNDLLRQRTTK 4ev6.1    GIYIKNNLLLTIHSDKIK-AIGRLHKLISTKKPRIVFERGIGFLLYHILNEITRSYSRILMNLEDELEELEDKLLAGYDR  target    KNLFVLSDLETGMVYLTAAAKQNRILLEHIQGHALYRSFDEIEREQFDDAMIEAHQLVSMTDLISQILQQLSASYNNILN 4ev6.1    EVMEKILGLRKTLVYFHKSLIANRDVLVLLKRKY-LPITTKEDRENFEDLYYDTLQLIDMSATYREVLTSMMDITLSLEN  target    NNLNDNLTTLTIISVLLAVLAVVTGFFGMNVP-LPLTDEPHAWLYISLASAGLWIVLSLLLRKIAKKS 4ev6.1    IKMNQIMKILTMVTTIFAVPMWITGIYGMNFSYLPLANNPQGFWLVMALMVVIIMIFVYIFRR----- ``` | | | | | | | | | | | | | | | | | | | | | | | | | | | | | | | | | | | | | | | | | | | | | | | | | |
|  | 4ev6.1.C | Magnesium transport protein CorA  *The complete structure of CorA magnesium transporter from Methanocaldococcus jannaschii* | 0.69 | 0.49 | 20.07 | 0.94 | 7-297 | X-ray | 3.20 | homo-pentamer | 8 x UMQ, 28 x MG | HHblits | 0.31 |
| ``` target    MVLEKQLGNGCTWIDLDLGKLNKLEDLSEIYGLDKETIEYALDRNERAHMDYHRESETVTFIYNVLDVKKDKAYYETFPM 4ev6.1    ------EDYRLIWIDCYDPKDEELYKLSKKIGISVSDLQIGLDEQEIPRVEEDEDF--YLIIYKAPLFE---EDITTTSL  target    TFIVEHRRLITISNTKNAYVIEQMTRYLENHD-----TLSIYKFLFASLEIISNAYYPVIEQMDKSRDEVNDLLRQRTTK 4ev6.1    GIYIKNNLLLTIHSDKIK-AIGRLHKLISTKKPRIVFERGIGFLLYHILNEITRSYSRILMNLEDELEELEDKLLAGYDR  target    KNLFVLSDLETGMVYLTAAAKQNRILLEHIQGHALYRSFDEIEREQFDDAMIEAHQLVSMTDLISQILQQLSASYNNILN 4ev6.1    EVMEKILGLRKTLVYFHKSLIANRDVLVLLKRKY-LPITTKEDRENFEDLYYDTLQLIDMSATYREVLTSMMDITLSLEN  target    NNLNDNLTTLTIISVLLAVLAVVTGFFGMNVP-LPLTDEPHAWLYISLASAGLWIVLSLLLRKIAKKS 4ev6.1    IKMNQIMKILTMVTTIFAVPMWITGIYGMNFSYLPLANNPQGFWLVMALMVVIIMIFVYIFRR----- ``` | | | | | | | | | | | | | | | | | | | | | | | | | | | | | | | | | | | | | | | | | | | | | | | | | |
|  | 4ev6.1.D | Magnesium transport protein CorA  *The complete structure of CorA magnesium transporter from Methanocaldococcus jannaschii* | 0.68 | 0.49 | 20.07 | 0.94 | 7-297 | X-ray | 3.20 | homo-pentamer | 8 x UMQ, 28 x MG | HHblits | 0.31 |
| ``` target    MVLEKQLGNGCTWIDLDLGKLNKLEDLSEIYGLDKETIEYALDRNERAHMDYHRESETVTFIYNVLDVKKDKAYYETFPM 4ev6.1    ------EDYRLIWIDCYDPKDEELYKLSKKIGISVSDLQIGLDEQEIPRVEEDEDF--YLIIYKAPLFE---EDITTTSL  target    TFIVEHRRLITISNTKNAYVIEQMTRYLENHD-----TLSIYKFLFASLEIISNAYYPVIEQMDKSRDEVNDLLRQRTTK 4ev6.1    GIYIKNNLLLTIHSDKIK-AIGRLHKLISTKKPRIVFERGIGFLLYHILNEITRSYSRILMNLEDELEELEDKLLAGYDR  target    KNLFVLSDLETGMVYLTAAAKQNRILLEHIQGHALYRSFDEIEREQFDDAMIEAHQLVSMTDLISQILQQLSASYNNILN 4ev6.1    EVMEKILGLRKTLVYFHKSLIANRDVLVLLKRKY-LPITTKEDRENFEDLYYDTLQLIDMSATYREVLTSMMDITLSLEN  target    NNLNDNLTTLTIISVLLAVLAVVTGFFGMNVP-LPLTDEPHAWLYISLASAGLWIVLSLLLRKIAKKS 4ev6.1    IKMNQIMKILTMVTTIFAVPMWITGIYGMNFSYLPLANNPQGFWLVMALMVVIIMIFVYIFRR----- ``` | | | | | | | | | | | | | | | | | | | | | | | | | | | | | | | | | | | | | | | | | | | | | | | | | |
| ✓ | 4ev6.1.E | Magnesium transport protein CorA  *The complete structure of CorA magnesium transporter from Methanocaldococcus jannaschii* | 0.70 | 0.49 | 20.07 | 0.94 | 7-297 | X-ray | 3.20 | homo-pentamer | 8 x UMQ, 28 x MG | HHblits | 0.31 |
| ``` target    MVLEKQLGNGCTWIDLDLGKLNKLEDLSEIYGLDKETIEYALDRNERAHMDYHRESETVTFIYNVLDVKKDKAYYETFPM 4ev6.1    ------EDYRLIWIDCYDPKDEELYKLSKKIGISVSDLQIGLDEQEIPRVEEDEDF--YLIIYKAPLFE---EDITTTSL  target    TFIVEHRRLITISNTKNAYVIEQMTRYLENHD-----TLSIYKFLFASLEIISNAYYPVIEQMDKSRDEVNDLLRQRTTK 4ev6.1    GIYIKNNLLLTIHSDKIK-AIGRLHKLISTKKPRIVFERGIGFLLYHILNEITRSYSRILMNLEDELEELEDKLLAGYDR  target    KNLFVLSDLETGMVYLTAAAKQNRILLEHIQGHALYRSFDEIEREQFDDAMIEAHQLVSMTDLISQILQQLSASYNNILN 4ev6.1    EVMEKILGLRKTLVYFHKSLIANRDVLVLLKRKY-LPITTKEDRENFEDLYYDTLQLIDMSATYREVLTSMMDITLSLEN  target    NNLNDNLTTLTIISVLLAVLAVVTGFFGMNVP-LPLTDEPHAWLYISLASAGLWIVLSLLLRKIAKKS 4ev6.1    IKMNQIMKILTMVTTIFAVPMWITGIYGMNFSYLPLANNPQGFWLVMALMVVIIMIFVYIFRR----- ``` | | | | | | | | | | | | | | | | | | | | | | | | | | | | | | | | | | | | | | | | | | | | | | | | | |
|  | 4eed.1.C | Magnesium transport protein CorA  *CorA coiled-coil mutant under Mg2+ presence* | 0.65 | 0.50 | 15.63 | 0.95 | 7-298 | X-ray | 3.92 | homo-pentamer | 14 x MG | HHblits | 0.30 |
| ``` target    MVLEKQLGNGCTWIDLDLGKLNK-LEDLSEIYGLDKETIEYALDRNERAHMDYHRESETVTFIYNVLDVKKDKAYYETFP 4eed.1    ------DSSTPTWINITGIHRTDVVQRVGEFFGIHPLVLEDILNVHQRPKVEFFENY--VFIVLKMFTYDKNLHELESEQ  target    MTFIVEHRRLITISNTKNAYVIEQMTRYLENHD----TLSIYKFLFASLEIISNAYYPVIEQMDKSRDEVNDLLRQRTTK 4eed.1    VSLILTKNCVLMFQEKIGD-VFDPVRERIRYNRGIIRKKRADYLLYSLIDALVDDYFVLLEKIDDEIDVLEEEVLERPEK  target    KNLFVLSDLETGMVYLTAAAKQNRILLEHIQGHALYRSFDEIEREQFDDAMIEAHQLVSMTDLISQILQQLSASYNNILN 4eed.1    ETVQRTHQLKRNLVELAATIWPLREVLSSLYRDV-PPLIEKETVPYFRDVYDHTIQIADTVETFRDIVSGLLDVYLSSVS  target    NNLNDNLTTLTIISVLLAVLAVVTGFFGMNVP-LPLTDEPHAWLYISLASAGLWIVLSLLLRKIAKKS 4eed.1    NKTNEVMKVLTIIATIFMPLTFIAGIYGMNFEYMPELRWKWGYPVVLAVMGVIAVIMVVYFKKK---- ``` | | | | | | | | | | | | | | | | | | | | | | | | | | | | | | | | | | | | | | | | | | | | | | | | | |
|  | 4eeb.1.A | Magnesium transport protein CorA  *CorA coiled-coil mutant under Mg2+ absence* | 0.65 | 0.38 | 15.63 | 0.95 | 7-298 | X-ray | 3.80 | homo-pentamer | 7 x CS | HHblits | 0.30 |
| ``` target    MVLEKQLGNGCTWIDLDLGKLNK-LEDLSEIYGLDKETIEYALDRNERAHMDYHRESETVTFIYNVLDVKKDKAYYETFP 4eeb.1    ------DSSTPTWINITGIHRTDVVQRVGEFFGIHPLVLEDILNVHQRPKVEFFENY--VFIVLKMFTYDKNLHELESEQ  target    MTFIVEHRRLITISNTKNAYVIEQMTRYLENHD----TLSIYKFLFASLEIISNAYYPVIEQMDKSRDEVNDLLRQRTTK 4eeb.1    VSLILTKNCVLMFQEKIGD-VFDPVRERIRYNRGIIRKKRADYLLYSLIDALVDDYFVLLEKIDDEIDVLEEEVLERPEK  target    KNLFVLSDLETGMVYLTAAAKQNRILLEHIQGHALYRSFDEIEREQFDDAMIEAHQLVSMTDLISQILQQLSASYNNILN 4eeb.1    ETVQRTHQLKRNLVELAATIWPLREVLSSLYRDV-PPLIEKETVPYFRDVYDHTIQIADTVETFRDIVSGLLDVYLSSVS  target    NNLNDNLTTLTIISVLLAVLAVVTGFFGMNVP-LPLTDEPHAWLYISLASAGLWIVLSLLLRKIAKKS 4eeb.1    NKTNEVMKVLTIIATIFMPLTFIAGIYGMNFEYMPELRWKWGYPVVLAVMGVIAVIMVVYFKKK---- ``` | | | | | | | | | | | | | | | | | | | | | | | | | | | | | | | | | | | | | | | | | | | | | | | | | |
|  | 4eeb.1.B | Magnesium transport protein CorA  *CorA coiled-coil mutant under Mg2+ absence* | 0.65 | 0.38 | 15.63 | 0.95 | 7-298 | X-ray | 3.80 | homo-pentamer | 7 x CS | HHblits | 0.30 |
| ``` target    MVLEKQLGNGCTWIDLDLGKLNK-LEDLSEIYGLDKETIEYALDRNERAHMDYHRESETVTFIYNVLDVKKDKAYYETFP 4eeb.1    ------DSSTPTWINITGIHRTDVVQRVGEFFGIHPLVLEDILNVHQRPKVEFFENY--VFIVLKMFTYDKNLHELESEQ  target    MTFIVEHRRLITISNTKNAYVIEQMTRYLENHD----TLSIYKFLFASLEIISNAYYPVIEQMDKSRDEVNDLLRQRTTK 4eeb.1    VSLILTKNCVLMFQEKIGD-VFDPVRERIRYNRGIIRKKRADYLLYSLIDALVDDYFVLLEKIDDEIDVLEEEVLERPEK  target    KNLFVLSDLETGMVYLTAAAKQNRILLEHIQGHALYRSFDEIEREQFDDAMIEAHQLVSMTDLISQILQQLSASYNNILN 4eeb.1    ETVQRTHQLKRNLVELAATIWPLREVLSSLYRDV-PPLIEKETVPYFRDVYDHTIQIADTVETFRDIVSGLLDVYLSSVS  target    NNLNDNLTTLTIISVLLAVLAVVTGFFGMNVP-LPLTDEPHAWLYISLASAGLWIVLSLLLRKIAKKS 4eeb.1    NKTNEVMKVLTIIATIFMPLTFIAGIYGMNFEYMPELRWKWGYPVVLAVMGVIAVIMVVYFKKK---- ``` | | | | | | | | | | | | | | | | | | | | | | | | | | | | | | | | | | | | | | | | | | | | | | | | | |
|  | 2bbj.1.A | divalent cation transport-related protein  *Crystal structure of the CorA Mg2+ transporter* | 0.64 | 0.41 | 15.33 | 0.95 | 8-298 | X-ray | 3.90 | homo-pentamer |  | HHblits | 0.30 |
| ``` target    MVLEKQLGNGCTWIDLDLGKL-NKLEDLSEIYGLDKETIEYALDRNERAHMDYHRESETVTFIYNVLDVKKDKAYYETFP 2bbj.1    -------SSTPTWINITGIHRTDVVQRVGEFFGIHPLVLEDILNVHQRPKVEFFEN--YVFIVLKMFTYDKNLHELESEQ  target    MTFIVEHRRLITISNTKNAYVIEQMTRYLENHD----TLSIYKFLFASLEIISNAYYPVIEQMDKSRDEVNDLLRQRTTK 2bbj.1    VSLILTKNCVLMFQEKI-GDVFDPVRERIRYNRGIIRKKRADYLLYSLIDALVDDYFVLLEKIDDEIDVLEEEVLERPEK  target    KNLFVLSDLETGMVYLTAAAKQNRILLEHIQGHALYRSFDEIEREQFDDAMIEAHQLVSMTDLISQILQQLSASYNNILN 2bbj.1    ETVQRTHQLKRNLVELRKTIWPLREVLSSLYRDV-PPLIEKETVPYFRDVYDHTIQIADTVETFRDIVSGLLDVYLSSVS  target    NNLNDNLTTLTIISVLLAVLAVVTGFFGMNVP-LPLTDEPHAWLYISLASAGLWIVLSLLLRKIAKKS 2bbj.1    NKTNEVMKVLTIIATIFMPLTFIAGIYGMNFEYMPELRWKWGYPVVLAVMGVIAVIMVVYFKKK---- ``` | | | | | | | | | | | | | | | | | | | | | | | | | | | | | | | | | | | | | | | | | | | | | | | | | |
|  | 2hn2.1.C | Magnesium transport protein corA  *Crystal structure of the CorA Mg2+ transporter homologue from T. maritima in complex with divalent cations* | 0.63 | 0.45 | 15.33 | 0.95 | 8-298 | X-ray | 3.70 | homo-pentamer | 12 x CA | HHblits | 0.30 |
| ``` target    MVLEKQLGNGCTWIDLDLGKL-NKLEDLSEIYGLDKETIEYALDRNERAHMDYHRESETVTFIYNVLDVKKDKAYYETFP 2hn2.1    -------SSTPTWINITGIHRTDVVQRVGEFFGIHPLVLEDILNVHQRPKVEFFEN--YVFIVLKMFTYDKNLHELESEQ  target    MTFIVEHRRLITISNTKNAYVIEQMTRYLENHD----TLSIYKFLFASLEIISNAYYPVIEQMDKSRDEVNDLLRQRTTK 2hn2.1    VSLILTKNCVLMFQEKI-GDVFDPVRERIRYNRGIIRKKRADYLLYSLIDALVDDYFVLLEKIDDEIDVLEEEVLERPEK  target    KNLFVLSDLETGMVYLTAAAKQNRILLEHIQGHALYRSFDEIEREQFDDAMIEAHQLVSMTDLISQILQQLSASYNNILN 2hn2.1    ETVQRTHQLKRNLVELRKTIWPLREVLSSLYRDV-PPLIEKETVPYFRDVYDHTIQIADTVETFRDIVSGLLDVYLSSVS  target    NNLNDNLTTLTIISVLLAVLAVVTGFFGMNVP-LPLTDEPHAWLYISLASAGLWIVLSLLLRKIAKKS 2hn2.1    NKTNEVMKVLTIIATIFMPLTFIAGIYGMNFEYMPELRWKWGYPVVLAVMGVIAVIMVVYFKKK---- ``` | | | | | | | | | | | | | | | | | | | | | | | | | | | | | | | | | | | | | | | | | | | | | | | | | |
|  | 2iub.1.A | DIVALENT CATION TRANSPORT-RELATED PROTEIN  *CRYSTAL STRUCTURE OF A DIVALENT METAL ION TRANSPORTER CORA AT 2.9 A RESOLUTION.* | 0.64 | 0.29 | 15.33 | 0.95 | 8-298 | X-ray | 2.90 | homo-pentamer | 10 x MG | HHblits | 0.30 |
| ``` target    MVLEKQLGNGCTWIDLDLGKL-NKLEDLSEIYGLDKETIEYALDRNERAHMDYHRESETVTFIYNVLDVKKDKAYYETFP 2iub.1    -------SSTPTWINITGIHRTDVVQRVGEFFGIHPLVLEDILNVHQRPKVEFFEN--YVFIVLKMFTYDKNLHELESEQ  target    MTFIVEHRRLITISNTKNAYVIEQMTRYLENHD----TLSIYKFLFASLEIISNAYYPVIEQMDKSRDEVNDLLRQRTTK 2iub.1    VSLILTKNCVLMFQEKI-GDVFDPVRERIRYNRGIIRKKRADYLLYSLIDALVDDYFVLLEKIDDEIDVLEEEVLERPEK  target    KNLFVLSDLETGMVYLTAAAKQNRILLEHIQGHALYRSFDEIEREQFDDAMIEAHQLVSMTDLISQILQQLSASYNNILN 2iub.1    ETVQRTHQLKRNLVELRKTIWPLREVLSSLYRDV-PPLIEKETVPYFRDVYDHTIQIADTVETFRDIVSGLLDVYLSSVS  target    NNLNDNLTTLTIISVLLAVLAVVTGFFGMNVP-LPLTDEPHAWLYISLASAGLWIVLSLLLRKIAKKS 2iub.1    NKTNEVMKVLTIIATIFMPLTFIAGIYGMNFEYMPELRWKWGYPVVLAVMGVIAVIMVVYFKKK---- ``` | | | | | | | | | | | | | | | | | | | | | | | | | | | | | | | | | | | | | | | | | | | | | | | | | |
|  | 2iub.1.C | DIVALENT CATION TRANSPORT-RELATED PROTEIN  *CRYSTAL STRUCTURE OF A DIVALENT METAL ION TRANSPORTER CORA AT 2.9 A RESOLUTION.* | 0.64 | 0.30 | 15.33 | 0.95 | 8-298 | X-ray | 2.90 | homo-pentamer | 10 x MG | HHblits | 0.30 |
| ``` target    MVLEKQLGNGCTWIDLDLGKL-NKLEDLSEIYGLDKETIEYALDRNERAHMDYHRESETVTFIYNVLDVKKDKAYYETFP 2iub.1    -------SSTPTWINITGIHRTDVVQRVGEFFGIHPLVLEDILNVHQRPKVEFFEN--YVFIVLKMFTYDKNLHELESEQ  target    MTFIVEHRRLITISNTKNAYVIEQMTRYLENHD----TLSIYKFLFASLEIISNAYYPVIEQMDKSRDEVNDLLRQRTTK 2iub.1    VSLILTKNCVLMFQEKI-GDVFDPVRERIRYNRGIIRKKRADYLLYSLIDALVDDYFVLLEKIDDEIDVLEEEVLERPEK  target    KNLFVLSDLETGMVYLTAAAKQNRILLEHIQGHALYRSFDEIEREQFDDAMIEAHQLVSMTDLISQILQQLSASYNNILN 2iub.1    ETVQRTHQLKRNLVELRKTIWPLREVLSSLYRDV-PPLIEKETVPYFRDVYDHTIQIADTVETFRDIVSGLLDVYLSSVS  target    NNLNDNLTTLTIISVLLAVLAVVTGFFGMNVP-LPLTDEPHAWLYISLASAGLWIVLSLLLRKIAKKS 2iub.1    NKTNEVMKVLTIIATIFMPLTFIAGIYGMNFEYMPELRWKWGYPVVLAVMGVIAVIMVVYFKKK---- ``` | | | | | | | | | | | | | | | | | | | | | | | | | | | | | | | | | | | | | | | | | | | | | | | | | |
|  | 3jcf.1.E | Magnesium transport protein CorA  *Cryo-EM structure of the magnesium channel CorA in the closed symmetric magnesium-bound state* | 0.67 | 0.46 | 15.33 | 0.95 | 8-298 | EM | 0.00 | homo-pentamer | 11 x MG | HHblits | 0.30 |
| ``` target    MVLEKQLGNGCTWIDLDLGKL-NKLEDLSEIYGLDKETIEYALDRNERAHMDYHRESETVTFIYNVLDVKKDKAYYETFP 3jcf.1    -------SSTPTWINITGIHRTDVVQRVGEFFGIHPLVLEDILNVHQRPKVEFFENY--VFIVLKMFTYDKNLHELESEQ  target    MTFIVEHRRLITISNTKNAYVIEQMTRYLENHD----TLSIYKFLFASLEIISNAYYPVIEQMDKSRDEVNDLLRQRTTK 3jcf.1    VSLILTKNCVLMFQEKI-GDVFDPVRERIRYNRGIIRKKRADYLLYSLIDALVDDYFVLLEKIDDEIDVLEEEVLERPEK  target    KNLFVLSDLETGMVYLTAAAKQNRILLEHIQGHALYRSFDEIEREQFDDAMIEAHQLVSMTDLISQILQQLSASYNNILN 3jcf.1    ETVQRTHQLKRNLVELRKTIWPLREVLSSLYRDV-PPLIEKETVPYFRDVYDHTIQIADTVETFRDIVSGLLDVYLSSVS  target    NNLNDNLTTLTIISVLLAVLAVVTGFFGMNVP-LPLTDEPHAWLYISLASAGLWIVLSLLLRKIAKKS 3jcf.1    NKTNEVMKVLTIIATIFMPLTFIAGIYGMNFEYMPELRWKWGYPVVLAVMGVIAVIMVVYFKKK---- ``` | | | | | | | | | | | | | | | | | | | | | | | | | | | | | | | | | | | | | | | | | | | | | | | | | |
|  | 4i0u.1.D | Magnesium transport protein CorA  *Improved structure of Thermotoga maritima CorA at 2.7 A resolution* | 0.67 |  | 15.33 | 0.95 | 8-298 | X-ray | 2.70 | homo-pentamer | 11 x MG, 3 x LMT | HHblits | 0.30 |
| ``` target    MVLEKQLGNGCTWIDLDLGKL-NKLEDLSEIYGLDKETIEYALDRNERAHMDYHRESETVTFIYNVLDVKKDKAYYETFP 4i0u.1    -------SSTPTWINITGIHRTDVVQRVGEFFGIHPLVLEDILNVHQRPKVEFFENY--VFIVLKMFTYDKNLHELESEQ  target    MTFIVEHRRLITISNTKNAYVIEQMTRYLENHD----TLSIYKFLFASLEIISNAYYPVIEQMDKSRDEVNDLLRQRTTK 4i0u.1    VSLILTKNCVLMFQEKI-GDVFDPVRERIRYNRGIIRKKRADYLLYSLIDALVDDYFVLLEKIDDEIDVLEEEVLERPEK  target    KNLFVLSDLETGMVYLTAAAKQNRILLEHIQGHALYRSFDEIEREQFDDAMIEAHQLVSMTDLISQILQQLSASYNNILN 4i0u.1    ETVQRTHQLKRNLVELRKTIWPLREVLSSLYRDV-PPLIEKETVPYFRDVYDHTIQIADTVETFRDIVSGLLDVYLSSVS  target    NNLNDNLTTLTIISVLLAVLAVVTGFFGMNVP-LPLTDEPHAWLYISLASAGLWIVLSLLLRKIAKKS 4i0u.1    NKTNEVMKVLTIIATIFMPLTFIAGIYGMNFEYMPELRWKWGYPVVLAVMGVIAVIMVVYFKKK---- ``` | | | | | | | | | | | | | | | | | | | | | | | | | | | | | | | | | | | | | | | | | | | | | | | | | |
|  | 4i0u.1.E | Magnesium transport protein CorA  *Improved structure of Thermotoga maritima CorA at 2.7 A resolution* | 0.67 |  | 15.33 | 0.95 | 8-298 | X-ray | 2.70 | homo-pentamer | 11 x MG, 3 x LMT | HHblits | 0.30 |
| ``` target    MVLEKQLGNGCTWIDLDLGKL-NKLEDLSEIYGLDKETIEYALDRNERAHMDYHRESETVTFIYNVLDVKKDKAYYETFP 4i0u.1    -------SSTPTWINITGIHRTDVVQRVGEFFGIHPLVLEDILNVHQRPKVEFFENY--VFIVLKMFTYDKNLHELESEQ  target    MTFIVEHRRLITISNTKNAYVIEQMTRYLENHD----TLSIYKFLFASLEIISNAYYPVIEQMDKSRDEVNDLLRQRTTK 4i0u.1    VSLILTKNCVLMFQEKI-GDVFDPVRERIRYNRGIIRKKRADYLLYSLIDALVDDYFVLLEKIDDEIDVLEEEVLERPEK  target    KNLFVLSDLETGMVYLTAAAKQNRILLEHIQGHALYRSFDEIEREQFDDAMIEAHQLVSMTDLISQILQQLSASYNNILN 4i0u.1    ETVQRTHQLKRNLVELRKTIWPLREVLSSLYRDV-PPLIEKETVPYFRDVYDHTIQIADTVETFRDIVSGLLDVYLSSVS  target    NNLNDNLTTLTIISVLLAVLAVVTGFFGMNVP-LPLTDEPHAWLYISLASAGLWIVLSLLLRKIAKKS 4i0u.1    NKTNEVMKVLTIIATIFMPLTFIAGIYGMNFEYMPELRWKWGYPVVLAVMGVIAVIMVVYFKKK---- ``` | | | | | | | | | | | | | | | | | | | | | | | | | | | | | | | | | | | | | | | | | | | | | | | | | |
|  | 4i0u.2.A | Magnesium transport protein CorA  *Improved structure of Thermotoga maritima CorA at 2.7 A resolution* | 0.67 |  | 15.33 | 0.95 | 8-298 | X-ray | 2.70 | homo-pentamer | 13 x MG | HHblits | 0.30 |
| ``` target    MVLEKQLGNGCTWIDLDLGKL-NKLEDLSEIYGLDKETIEYALDRNERAHMDYHRESETVTFIYNVLDVKKDKAYYETFP 4i0u.2    -------SSTPTWINITGIHRTDVVQRVGEFFGIHPLVLEDILNVHQRPKVEFFENY--VFIVLKMFTYDKNLHELESEQ  target    MTFIVEHRRLITISNTKNAYVIEQMTRYLENHD----TLSIYKFLFASLEIISNAYYPVIEQMDKSRDEVNDLLRQRTTK 4i0u.2    VSLILTKNCVLMFQEKI-GDVFDPVRERIRYNRGIIRKKRADYLLYSLIDALVDDYFVLLEKIDDEIDVLEEEVLERPEK  target    KNLFVLSDLETGMVYLTAAAKQNRILLEHIQGHALYRSFDEIEREQFDDAMIEAHQLVSMTDLISQILQQLSASYNNILN 4i0u.2    ETVQRTHQLKRNLVELRKTIWPLREVLSSLYRDV-PPLIEKETVPYFRDVYDHTIQIADTVETFRDIVSGLLDVYLSSVS  target    NNLNDNLTTLTIISVLLAVLAVVTGFFGMNVP-LPLTDEPHAWLYISLASAGLWIVLSLLLRKIAKKS 4i0u.2    NKTNEVMKVLTIIATIFMPLTFIAGIYGMNFEYMPELRWKWGYPVVLAVMGVIAVIMVVYFKKK---- ``` | | | | | | | | | | | | | | | | | | | | | | | | | | | | | | | | | | | | | | | | | | | | | | | | | |
|  | 4i0u.2.B | Magnesium transport protein CorA  *Improved structure of Thermotoga maritima CorA at 2.7 A resolution* | 0.67 |  | 15.33 | 0.95 | 8-298 | X-ray | 2.70 | homo-pentamer | 13 x MG | HHblits | 0.30 |
| ``` target    MVLEKQLGNGCTWIDLDLGKL-NKLEDLSEIYGLDKETIEYALDRNERAHMDYHRESETVTFIYNVLDVKKDKAYYETFP 4i0u.2    -------SSTPTWINITGIHRTDVVQRVGEFFGIHPLVLEDILNVHQRPKVEFFENY--VFIVLKMFTYDKNLHELESEQ  target    MTFIVEHRRLITISNTKNAYVIEQMTRYLENHD----TLSIYKFLFASLEIISNAYYPVIEQMDKSRDEVNDLLRQRTTK 4i0u.2    VSLILTKNCVLMFQEKI-GDVFDPVRERIRYNRGIIRKKRADYLLYSLIDALVDDYFVLLEKIDDEIDVLEEEVLERPEK  target    KNLFVLSDLETGMVYLTAAAKQNRILLEHIQGHALYRSFDEIEREQFDDAMIEAHQLVSMTDLISQILQQLSASYNNILN 4i0u.2    ETVQRTHQLKRNLVELRKTIWPLREVLSSLYRDV-PPLIEKETVPYFRDVYDHTIQIADTVETFRDIVSGLLDVYLSSVS  target    NNLNDNLTTLTIISVLLAVLAVVTGFFGMNVP-LPLTDEPHAWLYISLASAGLWIVLSLLLRKIAKKS 4i0u.2    NKTNEVMKVLTIIATIFMPLTFIAGIYGMNFEYMPELRWKWGYPVVLAVMGVIAVIMVVYFKKK---- ``` | | | | | | | | | | | | | | | | | | | | | | | | | | | | | | | | | | | | | | | | | | | | | | | | | |
|  | 4i0u.2.C | Magnesium transport protein CorA  *Improved structure of Thermotoga maritima CorA at 2.7 A resolution* | 0.67 |  | 15.33 | 0.95 | 8-298 | X-ray | 2.70 | homo-pentamer | 13 x MG | HHblits | 0.30 |
| ``` target    MVLEKQLGNGCTWIDLDLGKL-NKLEDLSEIYGLDKETIEYALDRNERAHMDYHRESETVTFIYNVLDVKKDKAYYETFP 4i0u.2    -------SSTPTWINITGIHRTDVVQRVGEFFGIHPLVLEDILNVHQRPKVEFFENY--VFIVLKMFTYDKNLHELESEQ  target    MTFIVEHRRLITISNTKNAYVIEQMTRYLENHD----TLSIYKFLFASLEIISNAYYPVIEQMDKSRDEVNDLLRQRTTK 4i0u.2    VSLILTKNCVLMFQEKI-GDVFDPVRERIRYNRGIIRKKRADYLLYSLIDALVDDYFVLLEKIDDEIDVLEEEVLERPEK  target    KNLFVLSDLETGMVYLTAAAKQNRILLEHIQGHALYRSFDEIEREQFDDAMIEAHQLVSMTDLISQILQQLSASYNNILN 4i0u.2    ETVQRTHQLKRNLVELRKTIWPLREVLSSLYRDV-PPLIEKETVPYFRDVYDHTIQIADTVETFRDIVSGLLDVYLSSVS  target    NNLNDNLTTLTIISVLLAVLAVVTGFFGMNVP-LPLTDEPHAWLYISLASAGLWIVLSLLLRKIAKKS 4i0u.2    NKTNEVMKVLTIIATIFMPLTFIAGIYGMNFEYMPELRWKWGYPVVLAVMGVIAVIMVVYFKKK---- ``` | | | | | | | | | | | | | | | | | | | | | | | | | | | | | | | | | | | | | | | | | | | | | | | | | |
|  | 4i0u.2.D | Magnesium transport protein CorA  *Improved structure of Thermotoga maritima CorA at 2.7 A resolution* | 0.67 |  | 15.33 | 0.95 | 8-298 | X-ray | 2.70 | homo-pentamer | 13 x MG | HHblits | 0.30 |
| ``` target    MVLEKQLGNGCTWIDLDLGKL-NKLEDLSEIYGLDKETIEYALDRNERAHMDYHRESETVTFIYNVLDVKKDKAYYETFP 4i0u.2    -------SSTPTWINITGIHRTDVVQRVGEFFGIHPLVLEDILNVHQRPKVEFFENY--VFIVLKMFTYDKNLHELESEQ  target    MTFIVEHRRLITISNTKNAYVIEQMTRYLENHD----TLSIYKFLFASLEIISNAYYPVIEQMDKSRDEVNDLLRQRTTK 4i0u.2    VSLILTKNCVLMFQEKI-GDVFDPVRERIRYNRGIIRKKRADYLLYSLIDALVDDYFVLLEKIDDEIDVLEEEVLERPEK  target    KNLFVLSDLETGMVYLTAAAKQNRILLEHIQGHALYRSFDEIEREQFDDAMIEAHQLVSMTDLISQILQQLSASYNNILN 4i0u.2    ETVQRTHQLKRNLVELRKTIWPLREVLSSLYRDV-PPLIEKETVPYFRDVYDHTIQIADTVETFRDIVSGLLDVYLSSVS  target    NNLNDNLTTLTIISVLLAVLAVVTGFFGMNVP-LPLTDEPHAWLYISLASAGLWIVLSLLLRKIAKKS 4i0u.2    NKTNEVMKVLTIIATIFMPLTFIAGIYGMNFEYMPELRWKWGYPVVLAVMGVIAVIMVVYFKKK---- ``` | | | | | | | | | | | | | | | | | | | | | | | | | | | | | | | | | | | | | | | | | | | | | | | | | |
|  | 4i0u.2.E | Magnesium transport protein CorA  *Improved structure of Thermotoga maritima CorA at 2.7 A resolution* | 0.67 |  | 15.33 | 0.95 | 8-298 | X-ray | 2.70 | homo-pentamer | 13 x MG | HHblits | 0.30 |
| ``` target    MVLEKQLGNGCTWIDLDLGKL-NKLEDLSEIYGLDKETIEYALDRNERAHMDYHRESETVTFIYNVLDVKKDKAYYETFP 4i0u.2    -------SSTPTWINITGIHRTDVVQRVGEFFGIHPLVLEDILNVHQRPKVEFFENY--VFIVLKMFTYDKNLHELESEQ  target    MTFIVEHRRLITISNTKNAYVIEQMTRYLENHD----TLSIYKFLFASLEIISNAYYPVIEQMDKSRDEVNDLLRQRTTK 4i0u.2    VSLILTKNCVLMFQEKI-GDVFDPVRERIRYNRGIIRKKRADYLLYSLIDALVDDYFVLLEKIDDEIDVLEEEVLERPEK  target    KNLFVLSDLETGMVYLTAAAKQNRILLEHIQGHALYRSFDEIEREQFDDAMIEAHQLVSMTDLISQILQQLSASYNNILN 4i0u.2    ETVQRTHQLKRNLVELRKTIWPLREVLSSLYRDV-PPLIEKETVPYFRDVYDHTIQIADTVETFRDIVSGLLDVYLSSVS  target    NNLNDNLTTLTIISVLLAVLAVVTGFFGMNVP-LPLTDEPHAWLYISLASAGLWIVLSLLLRKIAKKS 4i0u.2    NKTNEVMKVLTIIATIFMPLTFIAGIYGMNFEYMPELRWKWGYPVVLAVMGVIAVIMVVYFKKK---- ``` | | | | | | | | | | | | | | | | | | | | | | | | | | | | | | | | | | | | | | | | | | | | | | | | | |
|  | 4i0u.1.A | Magnesium transport protein CorA  *Improved structure of Thermotoga maritima CorA at 2.7 A resolution* | 0.67 |  | 15.33 | 0.95 | 8-298 | X-ray | 2.70 | homo-pentamer | 11 x MG, 3 x LMT | HHblits | 0.30 |
| ``` target    MVLEKQLGNGCTWIDLDLGKL-NKLEDLSEIYGLDKETIEYALDRNERAHMDYHRESETVTFIYNVLDVKKDKAYYETFP 4i0u.1    -------SSTPTWINITGIHRTDVVQRVGEFFGIHPLVLEDILNVHQRPKVEFFENY--VFIVLKMFTYDKNLHELESEQ  target    MTFIVEHRRLITISNTKNAYVIEQMTRYLENHD----TLSIYKFLFASLEIISNAYYPVIEQMDKSRDEVNDLLRQRTTK 4i0u.1    VSLILTKNCVLMFQEKI-GDVFDPVRERIRYNRGIIRKKRADYLLYSLIDALVDDYFVLLEKIDDEIDVLEEEVLERPEK  target    KNLFVLSDLETGMVYLTAAAKQNRILLEHIQGHALYRSFDEIEREQFDDAMIEAHQLVSMTDLISQILQQLSASYNNILN 4i0u.1    ETVQRTHQLKRNLVELRKTIWPLREVLSSLYRDV-PPLIEKETVPYFRDVYDHTIQIADTVETFRDIVSGLLDVYLSSVS  target    NNLNDNLTTLTIISVLLAVLAVVTGFFGMNVP-LPLTDEPHAWLYISLASAGLWIVLSLLLRKIAKKS 4i0u.1    NKTNEVMKVLTIIATIFMPLTFIAGIYGMNFEYMPELRWKWGYPVVLAVMGVIAVIMVVYFKKK---- ``` | | | | | | | | | | | | | | | | | | | | | | | | | | | | | | | | | | | | | | | | | | | | | | | | | |
|  | 4i0u.1.B | Magnesium transport protein CorA  *Improved structure of Thermotoga maritima CorA at 2.7 A resolution* | 0.67 |  | 15.33 | 0.95 | 8-298 | X-ray | 2.70 | homo-pentamer | 11 x MG, 3 x LMT | HHblits | 0.30 |
| ``` target    MVLEKQLGNGCTWIDLDLGKL-NKLEDLSEIYGLDKETIEYALDRNERAHMDYHRESETVTFIYNVLDVKKDKAYYETFP 4i0u.1    -------SSTPTWINITGIHRTDVVQRVGEFFGIHPLVLEDILNVHQRPKVEFFENY--VFIVLKMFTYDKNLHELESEQ  target    MTFIVEHRRLITISNTKNAYVIEQMTRYLENHD----TLSIYKFLFASLEIISNAYYPVIEQMDKSRDEVNDLLRQRTTK 4i0u.1    VSLILTKNCVLMFQEKI-GDVFDPVRERIRYNRGIIRKKRADYLLYSLIDALVDDYFVLLEKIDDEIDVLEEEVLERPEK  target    KNLFVLSDLETGMVYLTAAAKQNRILLEHIQGHALYRSFDEIEREQFDDAMIEAHQLVSMTDLISQILQQLSASYNNILN 4i0u.1    ETVQRTHQLKRNLVELRKTIWPLREVLSSLYRDV-PPLIEKETVPYFRDVYDHTIQIADTVETFRDIVSGLLDVYLSSVS  target    NNLNDNLTTLTIISVLLAVLAVVTGFFGMNVP-LPLTDEPHAWLYISLASAGLWIVLSLLLRKIAKKS 4i0u.1    NKTNEVMKVLTIIATIFMPLTFIAGIYGMNFEYMPELRWKWGYPVVLAVMGVIAVIMVVYFKKK---- ``` | | | | | | | | | | | | | | | | | | | | | | | | | | | | | | | | | | | | | | | | | | | | | | | | | |
|  | 3jcg.1.D | Magnesium transport protein CorA  *Cryo-EM structure of the magnesium channel CorA in the magnesium-free, asymmetric open state I* | 0.63 |  | 15.33 | 0.95 | 8-298 | EM | 0.00 | homo-pentamer |  | HHblits | 0.30 |
| ``` target    MVLEKQLGNGCTWIDLDLGKL-NKLEDLSEIYGLDKETIEYALDRNERAHMDYHRESETVTFIYNVLDVKKDKAYYETFP 3jcg.1    -------SSTPTWINITGIHRTDVVQRVGEFFGIHPLVLEDILNVHQRPKVEFFENY--VFIVLKMFTYDKNLHELESEQ  target    MTFIVEHRRLITISNTKNAYVIEQMTRYLENHD----TLSIYKFLFASLEIISNAYYPVIEQMDKSRDEVNDLLRQRTTK 3jcg.1    VSLILTKNCVLMFQEKI-GDVFDPVRERIRYNRGIIRKKRADYLLYSLIDALVDDYFVLLEKIDDEIDVLEEEVLERPEK  target    KNLFVLSDLETGMVYLTAAAKQNRILLEHIQGHALYRSFDEIEREQFDDAMIEAHQLVSMTDLISQILQQLSASYNNILN 3jcg.1    ETVQRTHQLKRNLVELRKTIWPLREVLSSLYRDV-PPLIEKETVPYFRDVYDHTIQIADTVETFRDIVSGLLDVYLSSVS  target    NNLNDNLTTLTIISVLLAVLAVVTGFFGMNVP-LPLTDEPHAWLYISLASAGLWIVLSLLLRKIAKKS 3jcg.1    NKTNEVMKVLTIIATIFMPLTFIAGIYGMNFEYMPELRWKWGYPVVLAVMGVIAVIMVVYFKKK---- ``` | | | | | | | | | | | | | | | | | | | | | | | | | | | | | | | | | | | | | | | | | | | | | | | | | |
|  | 3jcg.1.E | Magnesium transport protein CorA  *Cryo-EM structure of the magnesium channel CorA in the magnesium-free, asymmetric open state I* | 0.63 |  | 15.33 | 0.95 | 8-298 | EM | 0.00 | homo-pentamer |  | HHblits | 0.30 |
| ``` target    MVLEKQLGNGCTWIDLDLGKL-NKLEDLSEIYGLDKETIEYALDRNERAHMDYHRESETVTFIYNVLDVKKDKAYYETFP 3jcg.1    -------SSTPTWINITGIHRTDVVQRVGEFFGIHPLVLEDILNVHQRPKVEFFENY--VFIVLKMFTYDKNLHELESEQ  target    MTFIVEHRRLITISNTKNAYVIEQMTRYLENHD----TLSIYKFLFASLEIISNAYYPVIEQMDKSRDEVNDLLRQRTTK 3jcg.1    VSLILTKNCVLMFQEKI-GDVFDPVRERIRYNRGIIRKKRADYLLYSLIDALVDDYFVLLEKIDDEIDVLEEEVLERPEK  target    KNLFVLSDLETGMVYLTAAAKQNRILLEHIQGHALYRSFDEIEREQFDDAMIEAHQLVSMTDLISQILQQLSASYNNILN 3jcg.1    ETVQRTHQLKRNLVELRKTIWPLREVLSSLYRDV-PPLIEKETVPYFRDVYDHTIQIADTVETFRDIVSGLLDVYLSSVS  target    NNLNDNLTTLTIISVLLAVLAVVTGFFGMNVP-LPLTDEPHAWLYISLASAGLWIVLSLLLRKIAKKS 3jcg.1    NKTNEVMKVLTIIATIFMPLTFIAGIYGMNFEYMPELRWKWGYPVVLAVMGVIAVIMVVYFKKK---- ``` | | | | | | | | | | | | | | | | | | | | | | | | | | | | | | | | | | | | | | | | | | | | | | | | | |
|  | 3jcg.1.A | Magnesium transport protein CorA  *Cryo-EM structure of the magnesium channel CorA in the magnesium-free, asymmetric open state I* | 0.62 |  | 15.33 | 0.95 | 8-298 | EM | 0.00 | homo-pentamer |  | HHblits | 0.30 |
| ``` target    MVLEKQLGNGCTWIDLDLGKL-NKLEDLSEIYGLDKETIEYALDRNERAHMDYHRESETVTFIYNVLDVKKDKAYYETFP 3jcg.1    -------SSTPTWINITGIHRTDVVQRVGEFFGIHPLVLEDILNVHQRPKVEFFENY--VFIVLKMFTYDKNLHELESEQ  target    MTFIVEHRRLITISNTKNAYVIEQMTRYLENHD----TLSIYKFLFASLEIISNAYYPVIEQMDKSRDEVNDLLRQRTTK 3jcg.1    VSLILTKNCVLMFQEKI-GDVFDPVRERIRYNRGIIRKKRADYLLYSLIDALVDDYFVLLEKIDDEIDVLEEEVLERPEK  target    KNLFVLSDLETGMVYLTAAAKQNRILLEHIQGHALYRSFDEIEREQFDDAMIEAHQLVSMTDLISQILQQLSASYNNILN 3jcg.1    ETVQRTHQLKRNLVELRKTIWPLREVLSSLYRDV-PPLIEKETVPYFRDVYDHTIQIADTVETFRDIVSGLLDVYLSSVS  target    NNLNDNLTTLTIISVLLAVLAVVTGFFGMNVP-LPLTDEPHAWLYISLASAGLWIVLSLLLRKIAKKS 3jcg.1    NKTNEVMKVLTIIATIFMPLTFIAGIYGMNFEYMPELRWKWGYPVVLAVMGVIAVIMVVYFKKK---- ``` | | | | | | | | | | | | | | | | | | | | | | | | | | | | | | | | | | | | | | | | | | | | | | | | | |
|  | 3jcg.1.B | Magnesium transport protein CorA  *Cryo-EM structure of the magnesium channel CorA in the magnesium-free, asymmetric open state I* | 0.63 |  | 15.33 | 0.95 | 8-298 | EM | 0.00 | homo-pentamer |  | HHblits | 0.30 |
| ``` target    MVLEKQLGNGCTWIDLDLGKL-NKLEDLSEIYGLDKETIEYALDRNERAHMDYHRESETVTFIYNVLDVKKDKAYYETFP 3jcg.1    -------SSTPTWINITGIHRTDVVQRVGEFFGIHPLVLEDILNVHQRPKVEFFENY--VFIVLKMFTYDKNLHELESEQ  target    MTFIVEHRRLITISNTKNAYVIEQMTRYLENHD----TLSIYKFLFASLEIISNAYYPVIEQMDKSRDEVNDLLRQRTTK 3jcg.1    VSLILTKNCVLMFQEKI-GDVFDPVRERIRYNRGIIRKKRADYLLYSLIDALVDDYFVLLEKIDDEIDVLEEEVLERPEK  target    KNLFVLSDLETGMVYLTAAAKQNRILLEHIQGHALYRSFDEIEREQFDDAMIEAHQLVSMTDLISQILQQLSASYNNILN 3jcg.1    ETVQRTHQLKRNLVELRKTIWPLREVLSSLYRDV-PPLIEKETVPYFRDVYDHTIQIADTVETFRDIVSGLLDVYLSSVS  target    NNLNDNLTTLTIISVLLAVLAVVTGFFGMNVP-LPLTDEPHAWLYISLASAGLWIVLSLLLRKIAKKS 3jcg.1    NKTNEVMKVLTIIATIFMPLTFIAGIYGMNFEYMPELRWKWGYPVVLAVMGVIAVIMVVYFKKK---- ``` | | | | | | | | | | | | | | | | | | | | | | | | | | | | | | | | | | | | | | | | | | | | | | | | | |
|  | 3jcg.1.C | Magnesium transport protein CorA  *Cryo-EM structure of the magnesium channel CorA in the magnesium-free, asymmetric open state I* | 0.62 |  | 15.33 | 0.95 | 8-298 | EM | 0.00 | homo-pentamer |  | HHblits | 0.30 |
| ``` target    MVLEKQLGNGCTWIDLDLGKL-NKLEDLSEIYGLDKETIEYALDRNERAHMDYHRESETVTFIYNVLDVKKDKAYYETFP 3jcg.1    -------SSTPTWINITGIHRTDVVQRVGEFFGIHPLVLEDILNVHQRPKVEFFENY--VFIVLKMFTYDKNLHELESEQ  target    MTFIVEHRRLITISNTKNAYVIEQMTRYLENHD----TLSIYKFLFASLEIISNAYYPVIEQMDKSRDEVNDLLRQRTTK 3jcg.1    VSLILTKNCVLMFQEKI-GDVFDPVRERIRYNRGIIRKKRADYLLYSLIDALVDDYFVLLEKIDDEIDVLEEEVLERPEK  target    KNLFVLSDLETGMVYLTAAAKQNRILLEHIQGHALYRSFDEIEREQFDDAMIEAHQLVSMTDLISQILQQLSASYNNILN 3jcg.1    ETVQRTHQLKRNLVELRKTIWPLREVLSSLYRDV-PPLIEKETVPYFRDVYDHTIQIADTVETFRDIVSGLLDVYLSSVS  target    NNLNDNLTTLTIISVLLAVLAVVTGFFGMNVP-LPLTDEPHAWLYISLASAGLWIVLSLLLRKIAKKS 3jcg.1    NKTNEVMKVLTIIATIFMPLTFIAGIYGMNFEYMPELRWKWGYPVVLAVMGVIAVIMVVYFKKK---- ``` | | | | | | | | | | | | | | | | | | | | | | | | | | | | | | | | | | | | | | | | | | | | | | | | | |
|  | 4i0u.1.C | Magnesium transport protein CorA  *Improved structure of Thermotoga maritima CorA at 2.7 A resolution* | 0.67 |  | 15.33 | 0.95 | 8-298 | X-ray | 2.70 | homo-pentamer | 11 x MG, 3 x LMT | HHblits | 0.30 |
| ``` target    MVLEKQLGNGCTWIDLDLGKL-NKLEDLSEIYGLDKETIEYALDRNERAHMDYHRESETVTFIYNVLDVKKDKAYYETFP 4i0u.1    -------SSTPTWINITGIHRTDVVQRVGEFFGIHPLVLEDILNVHQRPKVEFFENY--VFIVLKMFTYDKNLHELESEQ  target    MTFIVEHRRLITISNTKNAYVIEQMTRYLENHD----TLSIYKFLFASLEIISNAYYPVIEQMDKSRDEVNDLLRQRTTK 4i0u.1    VSLILTKNCVLMFQEKI-GDVFDPVRERIRYNRGIIRKKRADYLLYSLIDALVDDYFVLLEKIDDEIDVLEEEVLERPEK  target    KNLFVLSDLETGMVYLTAAAKQNRILLEHIQGHALYRSFDEIEREQFDDAMIEAHQLVSMTDLISQILQQLSASYNNILN 4i0u.1    ETVQRTHQLKRNLVELRKTIWPLREVLSSLYRDV-PPLIEKETVPYFRDVYDHTIQIADTVETFRDIVSGLLDVYLSSVS  target    NNLNDNLTTLTIISVLLAVLAVVTGFFGMNVP-LPLTDEPHAWLYISLASAGLWIVLSLLLRKIAKKS 4i0u.1    NKTNEVMKVLTIIATIFMPLTFIAGIYGMNFEYMPELRWKWGYPVVLAVMGVIAVIMVVYFKKK---- ``` | | | | | | | | | | | | | | | | | | | | | | | | | | | | | | | | | | | | | | | | | | | | | | | | | |
|  | 3jch.1.C | Magnesium transport protein CorA  *Cryo-EM structure of the magnesium channel CorA in the magnesium-free, asymmetric open state II* | 0.64 |  | 15.33 | 0.95 | 8-298 | EM | 0.00 | homo-pentamer |  | HHblits | 0.30 |
| ``` target    MVLEKQLGNGCTWIDLDLGKL-NKLEDLSEIYGLDKETIEYALDRNERAHMDYHRESETVTFIYNVLDVKKDKAYYETFP 3jch.1    -------SSTPTWINITGIHRTDVVQRVGEFFGIHPLVLEDILNVHQRPKVEFFENY--VFIVLKMFTYDKNLHELESEQ  target    MTFIVEHRRLITISNTKNAYVIEQMTRYLENHD----TLSIYKFLFASLEIISNAYYPVIEQMDKSRDEVNDLLRQRTTK 3jch.1    VSLILTKNCVLMFQEKI-GDVFDPVRERIRYNRGIIRKKRADYLLYSLIDALVDDYFVLLEKIDDEIDVLEEEVLERPEK  target    KNLFVLSDLETGMVYLTAAAKQNRILLEHIQGHALYRSFDEIEREQFDDAMIEAHQLVSMTDLISQILQQLSASYNNILN 3jch.1    ETVQRTHQLKRNLVELRKTIWPLREVLSSLYRDV-PPLIEKETVPYFRDVYDHTIQIADTVETFRDIVSGLLDVYLSSVS  target    NNLNDNLTTLTIISVLLAVLAVVTGFFGMNVP-LPLTDEPHAWLYISLASAGLWIVLSLLLRKIAKKS 3jch.1    NKTNEVMKVLTIIATIFMPLTFIAGIYGMNFEYMPELRWKWGYPVVLAVMGVIAVIMVVYFKKK---- ``` | | | | | | | | | | | | | | | | | | | | | | | | | | | | | | | | | | | | | | | | | | | | | | | | | |
|  | 3jch.1.B | Magnesium transport protein CorA  *Cryo-EM structure of the magnesium channel CorA in the magnesium-free, asymmetric open state II* | 0.64 |  | 15.33 | 0.95 | 8-298 | EM | 0.00 | homo-pentamer |  | HHblits | 0.30 |
| ``` target    MVLEKQLGNGCTWIDLDLGKL-NKLEDLSEIYGLDKETIEYALDRNERAHMDYHRESETVTFIYNVLDVKKDKAYYETFP 3jch.1    -------SSTPTWINITGIHRTDVVQRVGEFFGIHPLVLEDILNVHQRPKVEFFENY--VFIVLKMFTYDKNLHELESEQ  target    MTFIVEHRRLITISNTKNAYVIEQMTRYLENHD----TLSIYKFLFASLEIISNAYYPVIEQMDKSRDEVNDLLRQRTTK 3jch.1    VSLILTKNCVLMFQEKI-GDVFDPVRERIRYNRGIIRKKRADYLLYSLIDALVDDYFVLLEKIDDEIDVLEEEVLERPEK  target    KNLFVLSDLETGMVYLTAAAKQNRILLEHIQGHALYRSFDEIEREQFDDAMIEAHQLVSMTDLISQILQQLSASYNNILN 3jch.1    ETVQRTHQLKRNLVELRKTIWPLREVLSSLYRDV-PPLIEKETVPYFRDVYDHTIQIADTVETFRDIVSGLLDVYLSSVS  target    NNLNDNLTTLTIISVLLAVLAVVTGFFGMNVP-LPLTDEPHAWLYISLASAGLWIVLSLLLRKIAKKS 3jch.1    NKTNEVMKVLTIIATIFMPLTFIAGIYGMNFEYMPELRWKWGYPVVLAVMGVIAVIMVVYFKKK---- ``` | | | | | | | | | | | | | | | | | | | | | | | | | | | | | | | | | | | | | | | | | | | | | | | | | |
|  | 3jch.1.A | Magnesium transport protein CorA  *Cryo-EM structure of the magnesium channel CorA in the magnesium-free, asymmetric open state II* | 0.62 |  | 15.33 | 0.95 | 8-298 | EM | 0.00 | homo-pentamer |  | HHblits | 0.30 |
| ``` target    MVLEKQLGNGCTWIDLDLGKL-NKLEDLSEIYGLDKETIEYALDRNERAHMDYHRESETVTFIYNVLDVKKDKAYYETFP 3jch.1    -------SSTPTWINITGIHRTDVVQRVGEFFGIHPLVLEDILNVHQRPKVEFFENY--VFIVLKMFTYDKNLHELESEQ  target    MTFIVEHRRLITISNTKNAYVIEQMTRYLENHD----TLSIYKFLFASLEIISNAYYPVIEQMDKSRDEVNDLLRQRTTK 3jch.1    VSLILTKNCVLMFQEKI-GDVFDPVRERIRYNRGIIRKKRADYLLYSLIDALVDDYFVLLEKIDDEIDVLEEEVLERPEK  target    KNLFVLSDLETGMVYLTAAAKQNRILLEHIQGHALYRSFDEIEREQFDDAMIEAHQLVSMTDLISQILQQLSASYNNILN 3jch.1    ETVQRTHQLKRNLVELRKTIWPLREVLSSLYRDV-PPLIEKETVPYFRDVYDHTIQIADTVETFRDIVSGLLDVYLSSVS  target    NNLNDNLTTLTIISVLLAVLAVVTGFFGMNVP-LPLTDEPHAWLYISLASAGLWIVLSLLLRKIAKKS 3jch.1    NKTNEVMKVLTIIATIFMPLTFIAGIYGMNFEYMPELRWKWGYPVVLAVMGVIAVIMVVYFKKK---- ``` | | | | | | | | | | | | | | | | | | | | | | | | | | | | | | | | | | | | | | | | | | | | | | | | | |
|  | 3jch.1.D | Magnesium transport protein CorA  *Cryo-EM structure of the magnesium channel CorA in the magnesium-free, asymmetric open state II* | 0.63 |  | 15.33 | 0.95 | 8-298 | EM | 0.00 | homo-pentamer |  | HHblits | 0.30 |
| ``` target    MVLEKQLGNGCTWIDLDLGKL-NKLEDLSEIYGLDKETIEYALDRNERAHMDYHRESETVTFIYNVLDVKKDKAYYETFP 3jch.1    -------SSTPTWINITGIHRTDVVQRVGEFFGIHPLVLEDILNVHQRPKVEFFENY--VFIVLKMFTYDKNLHELESEQ  target    MTFIVEHRRLITISNTKNAYVIEQMTRYLENHD----TLSIYKFLFASLEIISNAYYPVIEQMDKSRDEVNDLLRQRTTK 3jch.1    VSLILTKNCVLMFQEKI-GDVFDPVRERIRYNRGIIRKKRADYLLYSLIDALVDDYFVLLEKIDDEIDVLEEEVLERPEK  target    KNLFVLSDLETGMVYLTAAAKQNRILLEHIQGHALYRSFDEIEREQFDDAMIEAHQLVSMTDLISQILQQLSASYNNILN 3jch.1    ETVQRTHQLKRNLVELRKTIWPLREVLSSLYRDV-PPLIEKETVPYFRDVYDHTIQIADTVETFRDIVSGLLDVYLSSVS  target    NNLNDNLTTLTIISVLLAVLAVVTGFFGMNVP-LPLTDEPHAWLYISLASAGLWIVLSLLLRKIAKKS 3jch.1    NKTNEVMKVLTIIATIFMPLTFIAGIYGMNFEYMPELRWKWGYPVVLAVMGVIAVIMVVYFKKK---- ``` | | | | | | | | | | | | | | | | | | | | | | | | | | | | | | | | | | | | | | | | | | | | | | | | | |
|  | 5jtg.1.B | Cobalt/magnesium transport protein CorA  *Crystal structure of Thermotoga maritima mutant D89K/D253K* | 0.64 |  | 14.98 | 0.95 | 8-298 | X-ray | 3.05 | homo-pentamer | 3 x MG | HHblits | 0.29 |
| ``` target    MVLEKQLGNGCTWIDLDLGKL-NKLEDLSEIYGLDKETIEYALDRNERAHMDYHRESETVTFIYNVLDVKKDKAYYETFP 5jtg.1    -------SSTPTWINITGIHRTDVVQRVGEFFGIHPLVLEKILNVHQRPKVEFFENY--VFIVLKMFTYDKNLHELESEQ  target    MTFIVEHRRLITISNTKNAYVIEQMTRYLENHD----TLSIYKFLFASLEIISNAYYPVIEQMDKSRDEVNDLLRQRTTK 5jtg.1    VSLILTKNCVLMFQEKI-GDVFDPVRERIRYNRGIIRKKRADYLLYSLIDALVDDYFVLLEKIDDEIDVLEEEVLERPEK  target    KNLFVLSDLETGMVYLTAAAKQNRILLEHIQGHALYRSFDEIEREQFDDAMIEAHQLVSMTDLISQILQQLSASYNNILN 5jtg.1    ETVQRTHQLKRNLVELRKTIWPLREVLSSLYRDV-PPLIEKETVPYFRKVYDHTIQIADTVETFRDIVSGLLDVYLSSVS  target    NNLNDNLTTLTIISVLLAVLAVVTGFFGMNVP-LPLTDEPHAWLYISLASAGLWIVLSLLLRKIAKKS 5jtg.1    NKTNEVMKVLTIIATIFMPLTFIAGIYGMNFEYMPELRWKWGYPVVLAVMGVIAVIMVVYFKKK---- ``` | | | | | | | | | | | | | | | | | | | | | | | | | | | | | | | | | | | | | | | | | | | | | | | | | |
|  | 5jtg.1.A | Cobalt/magnesium transport protein CorA  *Crystal structure of Thermotoga maritima mutant D89K/D253K* | 0.64 |  | 14.98 | 0.95 | 8-298 | X-ray | 3.05 | homo-pentamer | 3 x MG | HHblits | 0.29 |
| ``` target    MVLEKQLGNGCTWIDLDLGKL-NKLEDLSEIYGLDKETIEYALDRNERAHMDYHRESETVTFIYNVLDVKKDKAYYETFP 5jtg.1    -------SSTPTWINITGIHRTDVVQRVGEFFGIHPLVLEKILNVHQRPKVEFFENY--VFIVLKMFTYDKNLHELESEQ  target    MTFIVEHRRLITISNTKNAYVIEQMTRYLENHD----TLSIYKFLFASLEIISNAYYPVIEQMDKSRDEVNDLLRQRTTK 5jtg.1    VSLILTKNCVLMFQEKI-GDVFDPVRERIRYNRGIIRKKRADYLLYSLIDALVDDYFVLLEKIDDEIDVLEEEVLERPEK  target    KNLFVLSDLETGMVYLTAAAKQNRILLEHIQGHALYRSFDEIEREQFDDAMIEAHQLVSMTDLISQILQQLSASYNNILN 5jtg.1    ETVQRTHQLKRNLVELRKTIWPLREVLSSLYRDV-PPLIEKETVPYFRKVYDHTIQIADTVETFRDIVSGLLDVYLSSVS  target    NNLNDNLTTLTIISVLLAVLAVVTGFFGMNVP-LPLTDEPHAWLYISLASAGLWIVLSLLLRKIAKKS 5jtg.1    NKTNEVMKVLTIIATIFMPLTFIAGIYGMNFEYMPELRWKWGYPVVLAVMGVIAVIMVVYFKKK---- ``` | | | | | | | | | | | | | | | | | | | | | | | | | | | | | | | | | | | | | | | | | | | | | | | | | |
|  | 5jtg.1.C | Cobalt/magnesium transport protein CorA  *Crystal structure of Thermotoga maritima mutant D89K/D253K* | 0.64 |  | 14.98 | 0.95 | 8-298 | X-ray | 3.05 | homo-pentamer | 3 x MG | HHblits | 0.29 |
| ``` target    MVLEKQLGNGCTWIDLDLGKL-NKLEDLSEIYGLDKETIEYALDRNERAHMDYHRESETVTFIYNVLDVKKDKAYYETFP 5jtg.1    -------SSTPTWINITGIHRTDVVQRVGEFFGIHPLVLEKILNVHQRPKVEFFENY--VFIVLKMFTYDKNLHELESEQ  target    MTFIVEHRRLITISNTKNAYVIEQMTRYLENHD----TLSIYKFLFASLEIISNAYYPVIEQMDKSRDEVNDLLRQRTTK 5jtg.1    VSLILTKNCVLMFQEKI-GDVFDPVRERIRYNRGIIRKKRADYLLYSLIDALVDDYFVLLEKIDDEIDVLEEEVLERPEK  target    KNLFVLSDLETGMVYLTAAAKQNRILLEHIQGHALYRSFDEIEREQFDDAMIEAHQLVSMTDLISQILQQLSASYNNILN 5jtg.1    ETVQRTHQLKRNLVELRKTIWPLREVLSSLYRDV-PPLIEKETVPYFRKVYDHTIQIADTVETFRDIVSGLLDVYLSSVS  target    NNLNDNLTTLTIISVLLAVLAVVTGFFGMNVP-LPLTDEPHAWLYISLASAGLWIVLSLLLRKIAKKS 5jtg.1    NKTNEVMKVLTIIATIFMPLTFIAGIYGMNFEYMPELRWKWGYPVVLAVMGVIAVIMVVYFKKK---- ``` | | | | | | | | | | | | | | | | | | | | | | | | | | | | | | | | | | | | | | | | | | | | | | | | | |
|  | 5jtg.1.D | Cobalt/magnesium transport protein CorA  *Crystal structure of Thermotoga maritima mutant D89K/D253K* | 0.64 |  | 14.98 | 0.95 | 8-298 | X-ray | 3.05 | homo-pentamer | 3 x MG | HHblits | 0.29 |
| ``` target    MVLEKQLGNGCTWIDLDLGKL-NKLEDLSEIYGLDKETIEYALDRNERAHMDYHRESETVTFIYNVLDVKKDKAYYETFP 5jtg.1    -------SSTPTWINITGIHRTDVVQRVGEFFGIHPLVLEKILNVHQRPKVEFFENY--VFIVLKMFTYDKNLHELESEQ  target    MTFIVEHRRLITISNTKNAYVIEQMTRYLENHD----TLSIYKFLFASLEIISNAYYPVIEQMDKSRDEVNDLLRQRTTK 5jtg.1    VSLILTKNCVLMFQEKI-GDVFDPVRERIRYNRGIIRKKRADYLLYSLIDALVDDYFVLLEKIDDEIDVLEEEVLERPEK  target    KNLFVLSDLETGMVYLTAAAKQNRILLEHIQGHALYRSFDEIEREQFDDAMIEAHQLVSMTDLISQILQQLSASYNNILN 5jtg.1    ETVQRTHQLKRNLVELRKTIWPLREVLSSLYRDV-PPLIEKETVPYFRKVYDHTIQIADTVETFRDIVSGLLDVYLSSVS  target    NNLNDNLTTLTIISVLLAVLAVVTGFFGMNVP-LPLTDEPHAWLYISLASAGLWIVLSLLLRKIAKKS 5jtg.1    NKTNEVMKVLTIIATIFMPLTFIAGIYGMNFEYMPELRWKWGYPVVLAVMGVIAVIMVVYFKKK---- ``` | | | | | | | | | | | | | | | | | | | | | | | | | | | | | | | | | | | | | | | | | | | | | | | | | |
|  | 5jrw.1.B | Cobalt/magnesium transport protein CorA  *Crystal structure of Thermotoga maritima mutant D89R/D253R* | 0.67 |  | 14.98 | 0.95 | 8-298 | X-ray | 3.30 | homo-pentamer | 5 x MG | HHblits | 0.29 |
| ``` target    MVLEKQLGNGCTWIDLDLGKL-NKLEDLSEIYGLDKETIEYALDRNERAHMDYHRESETVTFIYNVLDVKKDKAYYETFP 5jrw.1    -------SSTPTWINITGIHRTDVVQRVGEFFGIHPLVLERILNVHQRPKVEFFENY--VFIVLKMFTYDKNLHELESEQ  target    MTFIVEHRRLITISNTKNAYVIEQMTRYLENHD----TLSIYKFLFASLEIISNAYYPVIEQMDKSRDEVNDLLRQRTTK 5jrw.1    VSLILTKNCVLMFQEKI-GDVFDPVRERIRYNRGIIRKKRADYLLYSLIDALVDDYFVLLEKIDDEIDVLEEEVLERPEK  target    KNLFVLSDLETGMVYLTAAAKQNRILLEHIQGHALYRSFDEIEREQFDDAMIEAHQLVSMTDLISQILQQLSASYNNILN 5jrw.1    ETVQRTHQLKRNLVELRKTIWPLREVLSSLYRDV-PPLIEKETVPYFRRVYDHTIQIADTVETFRDIVSGLLDVYLSSVS  target    NNLNDNLTTLTIISVLLAVLAVVTGFFGMNVP-LPLTDEPHAWLYISLASAGLWIVLSLLLRKIAKKS 5jrw.1    NKTNEVMKVLTIIATIFMPLTFIAGIYGMNFEYMPELRWKWGYPVVLAVMGVIAVIMVVYFKKK---- ``` | | | | | | | | | | | | | | | | | | | | | | | | | | | | | | | | | | | | | | | | | | | | | | | | | |
|  | 5jrw.1.A | Cobalt/magnesium transport protein CorA  *Crystal structure of Thermotoga maritima mutant D89R/D253R* | 0.67 |  | 14.98 | 0.95 | 8-298 | X-ray | 3.30 | homo-pentamer | 5 x MG | HHblits | 0.29 |
| ``` target    MVLEKQLGNGCTWIDLDLGKL-NKLEDLSEIYGLDKETIEYALDRNERAHMDYHRESETVTFIYNVLDVKKDKAYYETFP 5jrw.1    -------SSTPTWINITGIHRTDVVQRVGEFFGIHPLVLERILNVHQRPKVEFFENY--VFIVLKMFTYDKNLHELESEQ  target    MTFIVEHRRLITISNTKNAYVIEQMTRYLENHD----TLSIYKFLFASLEIISNAYYPVIEQMDKSRDEVNDLLRQRTTK 5jrw.1    VSLILTKNCVLMFQEKI-GDVFDPVRERIRYNRGIIRKKRADYLLYSLIDALVDDYFVLLEKIDDEIDVLEEEVLERPEK  target    KNLFVLSDLETGMVYLTAAAKQNRILLEHIQGHALYRSFDEIEREQFDDAMIEAHQLVSMTDLISQILQQLSASYNNILN 5jrw.1    ETVQRTHQLKRNLVELRKTIWPLREVLSSLYRDV-PPLIEKETVPYFRRVYDHTIQIADTVETFRDIVSGLLDVYLSSVS  target    NNLNDNLTTLTIISVLLAVLAVVTGFFGMNVP-LPLTDEPHAWLYISLASAGLWIVLSLLLRKIAKKS 5jrw.1    NKTNEVMKVLTIIATIFMPLTFIAGIYGMNFEYMPELRWKWGYPVVLAVMGVIAVIMVVYFKKK---- ``` | | | | | | | | | | | | | | | | | | | | | | | | | | | | | | | | | | | | | | | | | | | | | | | | | |
|  | 5jrw.1.C | Cobalt/magnesium transport protein CorA  *Crystal structure of Thermotoga maritima mutant D89R/D253R* | 0.67 |  | 14.98 | 0.95 | 8-298 | X-ray | 3.30 | homo-pentamer | 5 x MG | HHblits | 0.29 |
| ``` target    MVLEKQLGNGCTWIDLDLGKL-NKLEDLSEIYGLDKETIEYALDRNERAHMDYHRESETVTFIYNVLDVKKDKAYYETFP 5jrw.1    -------SSTPTWINITGIHRTDVVQRVGEFFGIHPLVLERILNVHQRPKVEFFENY--VFIVLKMFTYDKNLHELESEQ  target    MTFIVEHRRLITISNTKNAYVIEQMTRYLENHD----TLSIYKFLFASLEIISNAYYPVIEQMDKSRDEVNDLLRQRTTK 5jrw.1    VSLILTKNCVLMFQEKI-GDVFDPVRERIRYNRGIIRKKRADYLLYSLIDALVDDYFVLLEKIDDEIDVLEEEVLERPEK  target    KNLFVLSDLETGMVYLTAAAKQNRILLEHIQGHALYRSFDEIEREQFDDAMIEAHQLVSMTDLISQILQQLSASYNNILN 5jrw.1    ETVQRTHQLKRNLVELRKTIWPLREVLSSLYRDV-PPLIEKETVPYFRRVYDHTIQIADTVETFRDIVSGLLDVYLSSVS  target    NNLNDNLTTLTIISVLLAVLAVVTGFFGMNVP-LPLTDEPHAWLYISLASAGLWIVLSLLLRKIAKKS 5jrw.1    NKTNEVMKVLTIIATIFMPLTFIAGIYGMNFEYMPELRWKWGYPVVLAVMGVIAVIMVVYFKKK---- ``` | | | | | | | | | | | | | | | | | | | | | | | | | | | | | | | | | | | | | | | | | | | | | | | | | |
|  | 5jrw.1.D | Cobalt/magnesium transport protein CorA  *Crystal structure of Thermotoga maritima mutant D89R/D253R* | 0.68 |  | 14.98 | 0.95 | 8-298 | X-ray | 3.30 | homo-pentamer | 5 x MG | HHblits | 0.29 |
| ``` target    MVLEKQLGNGCTWIDLDLGKL-NKLEDLSEIYGLDKETIEYALDRNERAHMDYHRESETVTFIYNVLDVKKDKAYYETFP 5jrw.1    -------SSTPTWINITGIHRTDVVQRVGEFFGIHPLVLERILNVHQRPKVEFFENY--VFIVLKMFTYDKNLHELESEQ  target    MTFIVEHRRLITISNTKNAYVIEQMTRYLENHD----TLSIYKFLFASLEIISNAYYPVIEQMDKSRDEVNDLLRQRTTK 5jrw.1    VSLILTKNCVLMFQEKI-GDVFDPVRERIRYNRGIIRKKRADYLLYSLIDALVDDYFVLLEKIDDEIDVLEEEVLERPEK  target    KNLFVLSDLETGMVYLTAAAKQNRILLEHIQGHALYRSFDEIEREQFDDAMIEAHQLVSMTDLISQILQQLSASYNNILN 5jrw.1    ETVQRTHQLKRNLVELRKTIWPLREVLSSLYRDV-PPLIEKETVPYFRRVYDHTIQIADTVETFRDIVSGLLDVYLSSVS  target    NNLNDNLTTLTIISVLLAVLAVVTGFFGMNVP-LPLTDEPHAWLYISLASAGLWIVLSLLLRKIAKKS 5jrw.1    NKTNEVMKVLTIIATIFMPLTFIAGIYGMNFEYMPELRWKWGYPVVLAVMGVIAVIMVVYFKKK---- ``` | | | | | | | | | | | | | | | | | | | | | | | | | | | | | | | | | | | | | | | | | | | | | | | | | |
|  | 5n9y.1.A | Zinc transport protein ZntB  *The full-length structure of ZntB* | 0.59 | 0.33 | 13.24 | 0.95 | 6-298 | EM | 0.00 | homo-pentamer |  | HHblits | 0.27 |
| ``` target    MVLEKQLGNGCTWIDLDLGKLNKLEDLSEIYGLDKETIEYALD-RNERAHMDYHRESETVTFIYNVLDVKKDKAYYETFP 5n9y.1    -----IDEAHPCWLHLNYVHHDSAQWLAT-TPLLPNNVRDALAGESTRPRVSRLGEG--TLITLRCINGSTDERPDQLVA  target    MTFIVEHRRLITISNTKNAYVIEQMTRYLENH-DTLSIYKFLFASLEIISNAYYPVIEQMDKSRDEVNDLLRQRTTKKNL 5n9y.1    MRVYMDGRLIVSTRQRKVL-ALDDVVSDLEEGTGPTDCGGWLVDVCDALTDHSSEFIEQLHDKIIDLEDNLLDQQI-PPR  target    FVLSDLETGMVYLTAAAKQNRILLEHIQGHALYRSFDEIEREQFDDAMIEAHQLVSMTDLISQILQQLSASYNNILNNNL 5n9y.1    GFLALLRKQLIVMRRYMAPQRDVYARLASER-LPWMSDDQRRRMQDIADRLGRGLDEIDACIARTGVMADEIAQVMQENL  target    NDNLTTLTIISVLLAVLAVVTGFFGMNVP-LPLTDEPHAWLYISLASAGLWIVLSLLLRKIAKKS 5n9y.1    ARRTYTMSLMAMVFLPSTFLTGLFGVNLGGIPGGGWQFGFSIFCILLVVLIGGVALWLHRS---- ``` | | | | | | | | | | | | | | | | | | | | | | | | | | | | | | | | | | | | | | | | | | | | | | | | | |
|  | 5n9y.1.B | Zinc transport protein ZntB  *The full-length structure of ZntB* | 0.60 | 0.33 | 13.24 | 0.95 | 6-298 | EM | 0.00 | homo-pentamer |  | HHblits | 0.27 |
| ``` target    MVLEKQLGNGCTWIDLDLGKLNKLEDLSEIYGLDKETIEYALD-RNERAHMDYHRESETVTFIYNVLDVKKDKAYYETFP 5n9y.1    -----IDEAHPCWLHLNYVHHDSAQWLAT-TPLLPNNVRDALAGESTRPRVSRLGEG--TLITLRCINGSTDERPDQLVA  target    MTFIVEHRRLITISNTKNAYVIEQMTRYLENH-DTLSIYKFLFASLEIISNAYYPVIEQMDKSRDEVNDLLRQRTTKKNL 5n9y.1    MRVYMDGRLIVSTRQRKVL-ALDDVVSDLEEGTGPTDCGGWLVDVCDALTDHSSEFIEQLHDKIIDLEDNLLDQQI-PPR  target    FVLSDLETGMVYLTAAAKQNRILLEHIQGHALYRSFDEIEREQFDDAMIEAHQLVSMTDLISQILQQLSASYNNILNNNL 5n9y.1    GFLALLRKQLIVMRRYMAPQRDVYARLASER-LPWMSDDQRRRMQDIADRLGRGLDEIDACIARTGVMADEIAQVMQENL  target    NDNLTTLTIISVLLAVLAVVTGFFGMNVP-LPLTDEPHAWLYISLASAGLWIVLSLLLRKIAKKS 5n9y.1    ARRTYTMSLMAMVFLPSTFLTGLFGVNLGGIPGGGWQFGFSIFCILLVVLIGGVALWLHRS---- ``` | | | | | | | | | | | | | | | | | | | | | | | | | | | | | | | | | | | | | | | | | | | | | | | | | |
|  | 5n9y.1.C | Zinc transport protein ZntB  *The full-length structure of ZntB* | 0.59 | 0.33 | 13.24 | 0.95 | 6-298 | EM | 0.00 | homo-pentamer |  | HHblits | 0.27 |
| ``` target    MVLEKQLGNGCTWIDLDLGKLNKLEDLSEIYGLDKETIEYALD-RNERAHMDYHRESETVTFIYNVLDVKKDKAYYETFP 5n9y.1    -----IDEAHPCWLHLNYVHHDSAQWLAT-TPLLPNNVRDALAGESTRPRVSRLGEG--TLITLRCINGSTDERPDQLVA  target    MTFIVEHRRLITISNTKNAYVIEQMTRYLENH-DTLSIYKFLFASLEIISNAYYPVIEQMDKSRDEVNDLLRQRTTKKNL 5n9y.1    MRVYMDGRLIVSTRQRKVL-ALDDVVSDLEEGTGPTDCGGWLVDVCDALTDHSSEFIEQLHDKIIDLEDNLLDQQI-PPR  target    FVLSDLETGMVYLTAAAKQNRILLEHIQGHALYRSFDEIEREQFDDAMIEAHQLVSMTDLISQILQQLSASYNNILNNNL 5n9y.1    GFLALLRKQLIVMRRYMAPQRDVYARLASER-LPWMSDDQRRRMQDIADRLGRGLDEIDACIARTGVMADEIAQVMQENL  target    NDNLTTLTIISVLLAVLAVVTGFFGMNVP-LPLTDEPHAWLYISLASAGLWIVLSLLLRKIAKKS 5n9y.1    ARRTYTMSLMAMVFLPSTFLTGLFGVNLGGIPGGGWQFGFSIFCILLVVLIGGVALWLHRS---- ``` | | | | | | | | | | | | | | | | | | | | | | | | | | | | | | | | | | | | | | | | | | | | | | | | | |
|  | 5n9y.1.D | Zinc transport protein ZntB  *The full-length structure of ZntB* | 0.59 | 0.33 | 13.24 | 0.95 | 6-298 | EM | 0.00 | homo-pentamer |  | HHblits | 0.27 |
| ``` target    MVLEKQLGNGCTWIDLDLGKLNKLEDLSEIYGLDKETIEYALD-RNERAHMDYHRESETVTFIYNVLDVKKDKAYYETFP 5n9y.1    -----IDEAHPCWLHLNYVHHDSAQWLAT-TPLLPNNVRDALAGESTRPRVSRLGEG--TLITLRCINGSTDERPDQLVA  target    MTFIVEHRRLITISNTKNAYVIEQMTRYLENH-DTLSIYKFLFASLEIISNAYYPVIEQMDKSRDEVNDLLRQRTTKKNL 5n9y.1    MRVYMDGRLIVSTRQRKVL-ALDDVVSDLEEGTGPTDCGGWLVDVCDALTDHSSEFIEQLHDKIIDLEDNLLDQQI-PPR  target    FVLSDLETGMVYLTAAAKQNRILLEHIQGHALYRSFDEIEREQFDDAMIEAHQLVSMTDLISQILQQLSASYNNILNNNL 5n9y.1    GFLALLRKQLIVMRRYMAPQRDVYARLASER-LPWMSDDQRRRMQDIADRLGRGLDEIDACIARTGVMADEIAQVMQENL  target    NDNLTTLTIISVLLAVLAVVTGFFGMNVP-LPLTDEPHAWLYISLASAGLWIVLSLLLRKIAKKS 5n9y.1    ARRTYTMSLMAMVFLPSTFLTGLFGVNLGGIPGGGWQFGFSIFCILLVVLIGGVALWLHRS---- ``` | | | | | | | | | | | | | | | | | | | | | | | | | | | | | | | | | | | | | | | | | | | | | | | | | |
|  | 5n9y.1.E | Zinc transport protein ZntB  *The full-length structure of ZntB* | 0.59 | 0.33 | 13.24 | 0.95 | 6-298 | EM | 0.00 | homo-pentamer |  | HHblits | 0.27 |
| ``` target    MVLEKQLGNGCTWIDLDLGKLNKLEDLSEIYGLDKETIEYALD-RNERAHMDYHRESETVTFIYNVLDVKKDKAYYETFP 5n9y.1    -----IDEAHPCWLHLNYVHHDSAQWLAT-TPLLPNNVRDALAGESTRPRVSRLGEG--TLITLRCINGSTDERPDQLVA  target    MTFIVEHRRLITISNTKNAYVIEQMTRYLENH-DTLSIYKFLFASLEIISNAYYPVIEQMDKSRDEVNDLLRQRTTKKNL 5n9y.1    MRVYMDGRLIVSTRQRKVL-ALDDVVSDLEEGTGPTDCGGWLVDVCDALTDHSSEFIEQLHDKIIDLEDNLLDQQI-PPR  target    FVLSDLETGMVYLTAAAKQNRILLEHIQGHALYRSFDEIEREQFDDAMIEAHQLVSMTDLISQILQQLSASYNNILNNNL 5n9y.1    GFLALLRKQLIVMRRYMAPQRDVYARLASER-LPWMSDDQRRRMQDIADRLGRGLDEIDACIARTGVMADEIAQVMQENL  target    NDNLTTLTIISVLLAVLAVVTGFFGMNVP-LPLTDEPHAWLYISLASAGLWIVLSLLLRKIAKKS 5n9y.1    ARRTYTMSLMAMVFLPSTFLTGLFGVNLGGIPGGGWQFGFSIFCILLVVLIGGVALWLHRS---- ``` | | | | | | | | | | | | | | | | | | | | | | | | | | | | | | | | | | | | | | | | | | | | | | | | | |
|  | 4egw.1.A | Magnesium transport protein CorA  *The structure of the soluble domain of CorA from Methanocaldococcus jannaschii* | 0.54 | 0.03 | 18.70 | 0.76 | 7-243 | X-ray | 2.50 | homo-dimer | 13 x HEZ, 3 x MG, 7 x PGO | HHblits | 0.30 |
| ``` target    MVLEKQLGNGCTWIDLDLGKLNKLEDLSEIYGLDKETIEYALDRNERAHMDYHRESETVTFIYNVLDVKKDKAYYETFPM 4egw.1    ------EDYRLIWIDCYDPKDEELYKLSKKIGISVSDLQIGLDEQEIPRVEEDED--FYLIIYKAPLFEE---DITTTSL  target    TFIVEHRRLITISNTKNAYVIEQMTRYLENHD-----TLSIYKFLFASLEIISNAYYPVIEQMDKSRDEVNDLLRQRTTK 4egw.1    GIYIKNNLLLTIHSDKIK-AIGRLHKLISTKKPRIVFERGIGFLLYHILNEITRSYSRILMNLEDELEELEDKLLAGYDR  target    KNLFVLSDLETGMVYLTAAAKQNRILLEHIQGHALYRSFDEIEREQFDDAMIEAHQLVSMTDLISQILQQLSASYNNILN 4egw.1    EVMEKILGLRKTLVYFHKSLIANRDVLVLLKRKY-LPITTKEDRENFEDLYYDTLQLIDMSATYREVLTSMMDITLSLEN  target    NNLNDNLTTLTIISVLLAVLAVVTGFFGMNVPLPLTDEPHAWLYISLASAGLWIVLSLLLRKIAKKS 4egw.1    IKMNQIMK----------------------------------------------------------- ``` | | | | | | | | | | | | | | | | | | | | | | | | | | | | | | | | | | | | | | | | | | | | | | | | | |
|  | 4egw.1.B | Magnesium transport protein CorA  *The structure of the soluble domain of CorA from Methanocaldococcus jannaschii* | 0.54 | 0.03 | 18.70 | 0.76 | 7-243 | X-ray | 2.50 | homo-dimer | 13 x HEZ, 3 x MG, 7 x PGO | HHblits | 0.30 |
| ``` target    MVLEKQLGNGCTWIDLDLGKLNKLEDLSEIYGLDKETIEYALDRNERAHMDYHRESETVTFIYNVLDVKKDKAYYETFPM 4egw.1    ------EDYRLIWIDCYDPKDEELYKLSKKIGISVSDLQIGLDEQEIPRVEEDED--FYLIIYKAPLFEE---DITTTSL  target    TFIVEHRRLITISNTKNAYVIEQMTRYLENHD-----TLSIYKFLFASLEIISNAYYPVIEQMDKSRDEVNDLLRQRTTK 4egw.1    GIYIKNNLLLTIHSDKIK-AIGRLHKLISTKKPRIVFERGIGFLLYHILNEITRSYSRILMNLEDELEELEDKLLAGYDR  target    KNLFVLSDLETGMVYLTAAAKQNRILLEHIQGHALYRSFDEIEREQFDDAMIEAHQLVSMTDLISQILQQLSASYNNILN 4egw.1    EVMEKILGLRKTLVYFHKSLIANRDVLVLLKRKY-LPITTKEDRENFEDLYYDTLQLIDMSATYREVLTSMMDITLSLEN  target    NNLNDNLTTLTIISVLLAVLAVVTGFFGMNVPLPLTDEPHAWLYISLASAGLWIVLSLLLRKIAKKS 4egw.1    IKMNQIMK----------------------------------------------------------- ``` | | | | | | | | | | | | | | | | | | | | | | | | | | | | | | | | | | | | | | | | | | | | | | | | | |
|  | 5n77.1.A | Magnesium transport protein CorA  *Crystal structure of the cytosolic domain of the CorA magnesium channel from Escherichia coli in complex with magnesium* | 0.50 | 0.25 | 13.04 | 0.76 | 7-243 | X-ray | 2.80 | homo-pentamer | 6 x MG | HHblits | 0.26 |
| ``` target    MVLEKQLGNGCTWIDLDLGKLNKLEDLSEIYGLDKETIEYALDRNERAHMDYHRESETVTFIYNVLDVKKDKAYYETFPM 5n77.1    ------PLVNAVWIDLVEPDDDERLRVQSELGQSLATRPELEDIEASARFFEDDD--GLHIHSFFFFEDA-EDHAGNSTV  target    TFIVEHRRLITISNTKNAYVIEQMTRYLENH--DTLSIYKFLFASLEIISNAYYPVIEQMDKSRDEVNDLLRQRTT---- 5n77.1    AFTIRDGRLFTLRERELP-AFRLYRMRARSQSMVDGNAYELLLDLFETKIEQLADEIENIYSDLEQLSRVIMEGHQGDEY  target    KKNLFVLSDLETGMVYLTAAAKQNRILLEHIQGHALYRSFDEIEREQFDDAMIEAHQLVSMTDLISQILQQLSASYNNIL 5n77.1    DEALSTLAELEDIGWKVRLCLMDTQRALNFLVRKA---RLPGGQLEQAREILRDIESLLPHNESLFQKVNFLMQAAMGFI  target    NNNLNDNLTTLTIISVLLAVLAVVTGFFGMNVPLPLTDEPHAWLYISLASAGLWIVLSLLLRKIAKKS 5n77.1    NIEQNRIIK----------------------------------------------------------- ``` | | | | | | | | | | | | | | | | | | | | | | | | | | | | | | | | | | | | | | | | | | | | | | | | | |
|  | 3nwi.1.A | Zinc transport protein zntB  *The Soluble Domain Structure of the ZntB Zn2+ Efflux System* | 0.44 | 0.08 | 10.71 | 0.74 | 7-236 | X-ray | 3.13 | homo-pentamer | 15 x ZN | HHblits | 0.27 |
| ``` target    MVLEKQLGNGCTWIDLDLGKLNKLEDLSEIYGLDKETIEYALD-RNERAHMDYHRESETVTFIYNVLDVKKDKAYYETFP 3nwi.1    ------DSQHPCWLHLNYTHPDSARWLAS-TPLLPNNVRDALAGESSRPRVSRMGEG--TLITLRCINGSTDERPDQLVA  target    MTFIVEHRRLITISNTKNAYVIEQMTRYLENH-DTLSIYKFLFASLEIISNAYYPVIEQMDKSRDEVNDLLRQRTTKKNL 3nwi.1    MRLYMDERFIVSTRQRKVL-ALDDVVSDLQEGTGPVDCGGWLVDVCDALTDHASEFIEELHDKIIDLEDNLLDQQI-PPR  target    FVLSDLETGMVYLTAAAKQNRILLEHIQGHALYRSFDEIEREQFDDAMIEAHQLVSMTDLISQILQQLSASYNNILNNNL 3nwi.1    GFLALLRKQLIVMRRYMAPQRDVYARLASER-LPWMSDDHRRRMQDIADRLGRGLDEIDACIARTGIMADEIAQVMQE--  target    NDNLTTLTIISVLLAVLAVVTGFFGMNVPLPLTDEPHAWLYISLASAGLWIVLSLLLRKIAKKS 3nwi.1    ---------------------------------------------------------------- ``` | | | | | | | | | | | | | | | | | | | | | | | | | | | | | | | | | | | | | | | | | | | | | | | | | |
|  | 3nvo.1.A | Zinc transport protein zntB  *The Soluble Domain Structure of the ZntB Zn2+ Efflux System* | 0.47 | 0.00 | 10.71 | 0.74 | 7-236 | X-ray | 2.30 | homo-dimer | 8 x ZN | HHblits | 0.27 |
| ``` target    MVLEKQLGNGCTWIDLDLGKLNKLEDLSEIYGLDKETIEYALD-RNERAHMDYHRESETVTFIYNVLDVKKDKAYYETFP 3nvo.1    ------DSQHPCWLHLNYTHPDSARWLAS-TPLLPNNVRDALAGESSRPRVSRMGE--GTLITLRCINGSTDERPDQLVA  target    MTFIVEHRRLITISNTKNAYVIEQMTRYLENH-DTLSIYKFLFASLEIISNAYYPVIEQMDKSRDEVNDLLRQRTTKKNL 3nvo.1    MRLYMDERFIVSTRQRKVL-ALDDVVSDLQEGTGPVDCGGWLVDVCDALTDHASEFIEELHDKIIDLEDNLLDQQI-PPR  target    FVLSDLETGMVYLTAAAKQNRILLEHIQGHALYRSFDEIEREQFDDAMIEAHQLVSMTDLISQILQQLSASYNNILNNNL 3nvo.1    GFLALLRKQLIVMRRYMAPQRDVYARLASER-LPWMSDDHRRRMQDIADRLGRGLDEIDACIARTGIMADEIAQVMQE--  target    NDNLTTLTIISVLLAVLAVVTGFFGMNVPLPLTDEPHAWLYISLASAGLWIVLSLLLRKIAKKS 3nvo.1    ---------------------------------------------------------------- ``` | | | | | | | | | | | | | | | | | | | | | | | | | | | | | | | | | | | | | | | | | | | | | | | | | |
|  | 3ck6.1.A | Putative membrane transport protein  *Crystal structure of ZntB cytoplasmic domain from Vibrio parahaemolyticus RIMD 2210633* | 0.44 | 0.16 | 8.93 | 0.74 | 7-241 | X-ray | 1.90 | homo-pentamer |  | HHblits | 0.26 |
| ``` target    MVLEKQLGNGCTWIDLDLGKLNKLEDLSEIYGLDKETIEYALDRNERAHMDYHRESETVTFIYNVLDVKKDKAYYETFPM 3ck6.1    ------HIQPNHWYHCERLHPD-IRGWLEDNHVPRATVDHLLADESRPSFHPLDDD-NFMLILRGINMNENASPEDMLSI  target    TFIVEHRRLITISNTKNAYVIEQMTRYLENHDTLSIYKFLFASLEIISNAYYPVIEQMDKSRDEVNDLLRQRTTK-KNLF 3ck6.1    RILYFQGALISTRKIPSR-AIMEIRQALAEHK--GPKSLA-SLLNQIIEGLNGKIDLY---LDTIEETLNEFDVNDESTY  target    VLSDLETGMVYLTAAAKQNRILLEHIQGHALYRSFDEIEREQFDDAMIEAHQLVSMTDLISQILQQLSASYNNILNNNLN 3ck6.1    NHIAAQKALISIKRFIRPQQYAIRDLIESE-SELVTSRP-HQYRFAHNNITRINETIEFYLGEVALFQDEIKHNRDEKTN  target    DNLTTLTIISVLLAVLAVVTGFFGMNVPLPLTDEPHAWLYISLASAGLWIVLSLLLRKIAKKS 3ck6.1    KN------------------------------------------------------------- ``` | | | | | | | | | | | | | | | | | | | | | | | | | | | | | | | | | | | | | | | | | | | | | | | | | |
|  | 3ck6.1.B | Putative membrane transport protein  *Crystal structure of ZntB cytoplasmic domain from Vibrio parahaemolyticus RIMD 2210633* | 0.44 | 0.16 | 8.93 | 0.74 | 7-241 | X-ray | 1.90 | homo-pentamer |  | HHblits | 0.26 |
| ``` target    MVLEKQLGNGCTWIDLDLGKLNKLEDLSEIYGLDKETIEYALDRNERAHMDYHRESETVTFIYNVLDVKKDKAYYETFPM 3ck6.1    ------HIQPNHWYHCERLHPD-IRGWLEDNHVPRATVDHLLADESRPSFHPLDDD-NFMLILRGINMNENASPEDMLSI  target    TFIVEHRRLITISNTKNAYVIEQMTRYLENHDTLSIYKFLFASLEIISNAYYPVIEQMDKSRDEVNDLLRQRTTK-KNLF 3ck6.1    RILYFQGALISTRKIPSR-AIMEIRQALAEHK--GPKSLA-SLLNQIIEGLNGKIDLY---LDTIEETLNEFDVNDESTY  target    VLSDLETGMVYLTAAAKQNRILLEHIQGHALYRSFDEIEREQFDDAMIEAHQLVSMTDLISQILQQLSASYNNILNNNLN 3ck6.1    NHIAAQKALISIKRFIRPQQYAIRDLIESE-SELVTSRP-HQYRFAHNNITRINETIEFYLGEVALFQDEIKHNRDEKTN  target    DNLTTLTIISVLLAVLAVVTGFFGMNVPLPLTDEPHAWLYISLASAGLWIVLSLLLRKIAKKS 3ck6.1    KN------------------------------------------------------------- ``` | | | | | | | | | | | | | | | | | | | | | | | | | | | | | | | | | | | | | | | | | | | | | | | | | |
|  | 3ck6.1.E | Putative membrane transport protein  *Crystal structure of ZntB cytoplasmic domain from Vibrio parahaemolyticus RIMD 2210633* | 0.44 | 0.16 | 8.93 | 0.74 | 7-241 | X-ray | 1.90 | homo-pentamer |  | HHblits | 0.26 |
| ``` target    MVLEKQLGNGCTWIDLDLGKLNKLEDLSEIYGLDKETIEYALDRNERAHMDYHRESETVTFIYNVLDVKKDKAYYETFPM 3ck6.1    ------HIQPNHWYHCERLHPD-IRGWLEDNHVPRATVDHLLADESRPSFHPLDDD-NFMLILRGINMNENASPEDMLSI  target    TFIVEHRRLITISNTKNAYVIEQMTRYLENHDTLSIYKFLFASLEIISNAYYPVIEQMDKSRDEVNDLLRQRTTK-KNLF 3ck6.1    RILYFQGALISTRKIPSR-AIMEIRQALAEHK--GPKSLA-SLLNQIIEGLNGKIDLY---LDTIEETLNEFDVNDESTY  target    VLSDLETGMVYLTAAAKQNRILLEHIQGHALYRSFDEIEREQFDDAMIEAHQLVSMTDLISQILQQLSASYNNILNNNLN 3ck6.1    NHIAAQKALISIKRFIRPQQYAIRDLIESE-SELVTSRP-HQYRFAHNNITRINETIEFYLGEVALFQDEIKHNRDEKTN  target    DNLTTLTIISVLLAVLAVVTGFFGMNVPLPLTDEPHAWLYISLASAGLWIVLSLLLRKIAKKS 3ck6.1    KN------------------------------------------------------------- ``` | | | | | | | | | | | | | | | | | | | | | | | | | | | | | | | | | | | | | | | | | | | | | | | | | |
|  | 2bbh.1.A | divalent cation transport-related protein  *X-ray structure of T.maritima CorA soluble domain* | 0.39 |  | 13.59 | 0.68 | 8-217 | X-ray | 1.85 | monomer | 4 x DMU, 1 x MG | HHblits | 0.29 |
| ``` target    MVLEKQLGNGCTWIDLDLGKL-NKLEDLSEIYGLDKETIEYALDRNERAHMDYHRESETVTFIYNVLDVKKDKAYYETFP 2bbh.1    -------SSTPTWINITGIHRTDVVQRVGEFFGTHPLVLEDILNVHQRPKVEFFEN--YVFIVLKMFTYDKNLHELESEQ  target    MTFIVEHRRLITISNTKNAYVIEQMTRYLENHD----TLSIYKFLFASLEIISNAYYPVIEQMDKSRDEVNDLLRQRTTK 2bbh.1    VSLILTKNCVLMFQEKI-GDVFDPVRERIRYNRGIIRKKRADYLLYSLIDALVDDYFVLLEKIDDEIDVLEEEVLERPEK  target    KNLFVLSDLETGMVYLTAAAKQNRILLEHIQGHALYRSFDEIEREQFDDAMIEAHQLVSMTDLISQILQQLSASYNNILN 2bbh.1    ETVQRTHQLKRNLVELRKTIWPLREVLSSLYRDV-PPLIEKETVPYFRDVYDHTIQIADTVE------------------  target    NNLNDNLTTLTIISVLLAVLAVVTGFFGMNVPLPLTDEPHAWLYISLASAGLWIVLSLLLRKIAKKS 2bbh.1    ------------------------------------------------------------------- ``` | | | | | | | | | | | | | | | | | | | | | | | | | | | | | | | | | | | | | | | | | | | | | | | | | |
|  | 2hn1.1.A | Magnesium and cobalt transporter  *Crystal structure of a CorA soluble domain from A. fulgidus in complex with Co2+* | 0.42 | 0.18 | 12.50 | 0.66 | 7-214 | X-ray | 2.90 | homo-dimer | 2 x CO | HHblits | 0.28 |
| ``` target    MVLEKQLGNGCTWIDLDLGK-LNKLEDLSEIYGLDKETIEYALDRNERAHMDYHRESETVTFIYNVLDVKKDKAYYETFP 2hn1.1    ------ALNKKLWIDVVGVHDESLIAKICEFLGIHPLAAEDILNTAQRVKIEDYDD--HLFLVLKILLYN---ETLEIDQ  target    MTFIVEHRRLITISNTKNAYVIEQMTRYLENHD---TLSIYKFLFASLEIISNAYYPVIEQMDKSRDEVNDLLRQRTTKK 2hn1.1    LSLVLKKNLVATFEERE-YWILDSIRSRLKSGGRMRKLAGDYLAYTILDAVVDSYFEALLKISDEIEVLEDEVVSGD-ST  target    NLFVLSDLETGMVYLTAAAKQNRILLEHIQGHALYRSFDEIEREQFDDAMIEAHQLVSMTDLISQILQQLSASYNNILNN 2hn1.1    LIGKIHSLKREILAFRNAVWPLRDVLSFFTRVE-HELIGEEVKVYYRDVYDHAVRLME----------------------  target    NLNDNLTTLTIISVLLAVLAVVTGFFGMNVPLPLTDEPHAWLYISLASAGLWIVLSLLLRKIAKKS 2hn1.1    ------------------------------------------------------------------ ``` | | | | | | | | | | | | | | | | | | | | | | | | | | | | | | | | | | | | | | | | | | | | | | | | | |
|  | 3rkg.1.A | Magnesium transporter MRS2, mitochondrial  *Structural and Functional Characterization of the Yeast Mg2+ Channel Mrs2* | 0.26 | 0.00 | 8.54 | 0.54 | 9-188 | X-ray | 1.28 | monomer |  | HHblits | 0.25 |
| ``` target    MVLEKQLGNGCTWIDLDLGK-L----N--KLEDLSEIYGLDKETIEYALDR--NERAHMDYHRESETVTFIYNVLDVKKD 3rkg.1    --------LFISCTVFNSKGNIISMSEKFPKWSFLTEHSLFPRDLRKIDNSSIDIIPTIMCKPN----CIVINL------  target    KAYYETFPMTFIVEHRRLITISNTKNA--YVIEQ----MTRYLENH-DTLSIY--KFLFASLEIISNAYYPVIEQMDKSR 3rkg.1    ------LHIKALIERDKVYVFDTTNPSAAAKLSVLMYDLESKLSSTKNNSQFYEHRALESIFINVMSALETDFKLHSQIC  target    DEVNDLLRQRTTKKNLFVLSDLETGMVYLTAAAKQNRILLEHIQGHALYRSFDEIEREQFDDAMIEAHQLVSMTDLISQI 3rkg.1    IQILNDLENEVNRLKLRHLLIKSKDLTLFYQKTLLIRDLLDELLEN----------------------------------  target    LQQLSASYNNILNNNLNDNLTTLTIISVLLAVLAVVTGFFGMNVPLPLTDEPHAWLYISLASAGLWIVLSLLLRKIAKKS 3rkg.1    -------------------------------------------------------------------------------- ``` | | | | | | | | | | | | | | | | | | | | | | | | | | | | | | | | | | | | | | | | | | | | | | | | | |
|  | 3jc8.42.A | Type 4 fimbrial assembly protein PilC  *Architectural model of the type IVa pilus machine in a piliated state* | 0.04 | 0.00 | 8.93 | 0.19 | 201-256 | EM | 0.00 | monomer |  | HHblits | 0.27 |
| ``` target    MVLEKQLGNGCTWIDLDLGKLNKLEDLSEIYGLDKETIEYALDRNERAHMDYHRESETVTFIYNVLDVKKDKAYYETFPM 3jc8.42   --------------------------------------------------------------------------------  target    TFIVEHRRLITISNTKNAYVIEQMTRYLENHDTLSIYKFLFASLEIISNAYYPVIEQMDKSRDEVNDLLRQRTTKKNLFV 3jc8.42   --------------------------------------------------------------------------------  target    LSDLETGMVYLTAAAKQNRILLEHIQGHALYRSFDEIEREQFDDAMIEAHQLVSMTDLISQILQQLSASYNNILNNNLND 3jc8.42   ----------------------------------------VFPSMVVQMIGVGEATGAMDTMLNKIADFYDDEVDAAINS  target    NLTTLTIISVLLAVLAVVTGFFGMNVPLPLTDEPHAWLYISLASAGLWIVLSLLLRKIAKKS 3jc8.42   LTAMIEPVLMVFLGGV---------------------------------------------- ``` | | | | | | | | | | | | | | | | | | | | | | | | | | | | | | | | | | | | | | | | | | | | | | | | | |
|  | 3jc8.43.A | Type 4 fimbrial assembly protein PilC  *Architectural model of the type IVa pilus machine in a piliated state* | 0.04 | 0.00 | 8.93 | 0.19 | 201-256 | EM | 0.00 | monomer |  | HHblits | 0.27 |
| ``` target    MVLEKQLGNGCTWIDLDLGKLNKLEDLSEIYGLDKETIEYALDRNERAHMDYHRESETVTFIYNVLDVKKDKAYYETFPM 3jc8.43   --------------------------------------------------------------------------------  target    TFIVEHRRLITISNTKNAYVIEQMTRYLENHDTLSIYKFLFASLEIISNAYYPVIEQMDKSRDEVNDLLRQRTTKKNLFV 3jc8.43   --------------------------------------------------------------------------------  target    LSDLETGMVYLTAAAKQNRILLEHIQGHALYRSFDEIEREQFDDAMIEAHQLVSMTDLISQILQQLSASYNNILNNNLND 3jc8.43   ----------------------------------------VFPSMVVQMIGVGEATGAMDTMLNKIADFYDDEVDAAINS  target    NLTTLTIISVLLAVLAVVTGFFGMNVPLPLTDEPHAWLYISLASAGLWIVLSLLLRKIAKKS 3jc8.43   LTAMIEPVLMVFLGGV---------------------------------------------- ``` | | | | | | | | | | | | | | | | | | | | | | | | | | | | | | | | | | | | | | | | | | | | | | | | | |
|  | 6ysl.1.A | Motility protein A  *Structure of the flagellar MotAB stator complex from Bacillus subtilis* | 0.04 | 0.00 | 20.83 | 0.16 | 242-291 | EM | 0.00 | homo-pentamer |  | HHblits | 0.31 |
| ``` target    MVLEKQLGNGCTWIDLDLGKLNKLEDLSEIYGLDKETIEYALDRNERAHMDYHRESETVTFIYNVLDVKKDKAYYETFPM 6ysl.1    --------------------------------------------------------------------------------  target    TFIVEHRRLITISNTKNAYVIEQMTRYLENHDTLSIYKFLFASLEIISNAYYPVIEQMDKSRDEVNDLLRQRTTKKNLFV 6ysl.1    --------------------------------------------------------------------------------  target    LSDLETGMVYLTAAAKQNRILLEHIQGHALYRSFDEIEREQFDDAMIEAHQLVSMTDLISQILQQLSASYNNILNNNLND 6ysl.1    --------------------------------------------------------------------------------  target    NLTTLTIISVLLAVLAVVTGFFGM--NVPLPLTDEPHAWLYISLASAGLWIVLSLLLRKIAKKS 6ysl.1    -MDKTSLIGIILAFVALSVGMVLKGVSF-SALA-NPAAILIIIAGTISAVVIA----------- ``` | | | | | | | | | | | | | | | | | | | | | | | | | | | | | | | | | | | | | | | | | | | | | | | | | |
|  | 6ysl.1.D | Motility protein A  *Structure of the flagellar MotAB stator complex from Bacillus subtilis* | 0.04 | 0.00 | 20.83 | 0.16 | 242-291 | EM | 0.00 | homo-pentamer |  | HHblits | 0.31 |
| ``` target    MVLEKQLGNGCTWIDLDLGKLNKLEDLSEIYGLDKETIEYALDRNERAHMDYHRESETVTFIYNVLDVKKDKAYYETFPM 6ysl.1    --------------------------------------------------------------------------------  target    TFIVEHRRLITISNTKNAYVIEQMTRYLENHDTLSIYKFLFASLEIISNAYYPVIEQMDKSRDEVNDLLRQRTTKKNLFV 6ysl.1    --------------------------------------------------------------------------------  target    LSDLETGMVYLTAAAKQNRILLEHIQGHALYRSFDEIEREQFDDAMIEAHQLVSMTDLISQILQQLSASYNNILNNNLND 6ysl.1    --------------------------------------------------------------------------------  target    NLTTLTIISVLLAVLAVVTGFFGM--NVPLPLTDEPHAWLYISLASAGLWIVLSLLLRKIAKKS 6ysl.1    -MDKTSLIGIILAFVALSVGMVLKGVSF-SALA-NPAAILIIIAGTISAVVIA----------- ``` | | | | | | | | | | | | | | | | | | | | | | | | | | | | | | | | | | | | | | | | | | | | | | | | | |
|  | 6ysl.1.E | Motility protein A  *Structure of the flagellar MotAB stator complex from Bacillus subtilis* | 0.04 | 0.00 | 20.83 | 0.16 | 242-291 | EM | 0.00 | homo-pentamer |  | HHblits | 0.31 |
| ``` target    MVLEKQLGNGCTWIDLDLGKLNKLEDLSEIYGLDKETIEYALDRNERAHMDYHRESETVTFIYNVLDVKKDKAYYETFPM 6ysl.1    --------------------------------------------------------------------------------  target    TFIVEHRRLITISNTKNAYVIEQMTRYLENHDTLSIYKFLFASLEIISNAYYPVIEQMDKSRDEVNDLLRQRTTKKNLFV 6ysl.1    --------------------------------------------------------------------------------  target    LSDLETGMVYLTAAAKQNRILLEHIQGHALYRSFDEIEREQFDDAMIEAHQLVSMTDLISQILQQLSASYNNILNNNLND 6ysl.1    --------------------------------------------------------------------------------  target    NLTTLTIISVLLAVLAVVTGFFGM--NVPLPLTDEPHAWLYISLASAGLWIVLSLLLRKIAKKS 6ysl.1    -MDKTSLIGIILAFVALSVGMVLKGVSF-SALA-NPAAILIIIAGTISAVVIA----------- ``` | | | | | | | | | | | | | | | | | | | | | | | | | | | | | | | | | | | | | | | | | | | | | | | | | |
|  | 6ysl.1.F | Motility protein A  *Structure of the flagellar MotAB stator complex from Bacillus subtilis* | 0.04 | 0.00 | 20.83 | 0.16 | 242-291 | EM | 0.00 | homo-pentamer |  | HHblits | 0.31 |
| ``` target    MVLEKQLGNGCTWIDLDLGKLNKLEDLSEIYGLDKETIEYALDRNERAHMDYHRESETVTFIYNVLDVKKDKAYYETFPM 6ysl.1    --------------------------------------------------------------------------------  target    TFIVEHRRLITISNTKNAYVIEQMTRYLENHDTLSIYKFLFASLEIISNAYYPVIEQMDKSRDEVNDLLRQRTTKKNLFV 6ysl.1    --------------------------------------------------------------------------------  target    LSDLETGMVYLTAAAKQNRILLEHIQGHALYRSFDEIEREQFDDAMIEAHQLVSMTDLISQILQQLSASYNNILNNNLND 6ysl.1    --------------------------------------------------------------------------------  target    NLTTLTIISVLLAVLAVVTGFFGM--NVPLPLTDEPHAWLYISLASAGLWIVLSLLLRKIAKKS 6ysl.1    -MDKTSLIGIILAFVALSVGMVLKGVSF-SALA-NPAAILIIIAGTISAVVIA----------- ``` | | | | | | | | | | | | | | | | | | | | | | | | | | | | | | | | | | | | | | | | | | | | | | | | | |
|  | 6ysl.1.G | Motility protein A  *Structure of the flagellar MotAB stator complex from Bacillus subtilis* | 0.04 | 0.00 | 20.83 | 0.16 | 242-291 | EM | 0.00 | homo-pentamer |  | HHblits | 0.31 |
| ``` target    MVLEKQLGNGCTWIDLDLGKLNKLEDLSEIYGLDKETIEYALDRNERAHMDYHRESETVTFIYNVLDVKKDKAYYETFPM 6ysl.1    --------------------------------------------------------------------------------  target    TFIVEHRRLITISNTKNAYVIEQMTRYLENHDTLSIYKFLFASLEIISNAYYPVIEQMDKSRDEVNDLLRQRTTKKNLFV 6ysl.1    --------------------------------------------------------------------------------  target    LSDLETGMVYLTAAAKQNRILLEHIQGHALYRSFDEIEREQFDDAMIEAHQLVSMTDLISQILQQLSASYNNILNNNLND 6ysl.1    --------------------------------------------------------------------------------  target    NLTTLTIISVLLAVLAVVTGFFGM--NVPLPLTDEPHAWLYISLASAGLWIVLSLLLRKIAKKS 6ysl.1    -MDKTSLIGIILAFVALSVGMVLKGVSF-SALA-NPAAILIIIAGTISAVVIA----------- ``` | | | | | | | | | | | | | | | | | | | | | | | | | | | | | | | | | | | | | | | | | | | | | | | | | |
|  | 7kdp.1.A | Envelope glycoprotein B  *HCMV prefusion gB in complex with fusion inhibitor WAY-174865* | 0.03 | 0.00 | 17.02 | 0.16 | 251-297 | EM | 0.00 | homo-trimer | 30 x NAG, 3 x WCY | HHblits | 0.29 |
| ``` target    MVLEKQLGNGCTWIDLDLGKLNKLEDLSEIYGLDKETIEYALDRNERAHMDYHRESETVTFIYNVLDVKKDKAYYETFPM 7kdp.1    --------------------------------------------------------------------------------  target    TFIVEHRRLITISNTKNAYVIEQMTRYLENHDTLSIYKFLFASLEIISNAYYPVIEQMDKSRDEVNDLLRQRTTKKNLFV 7kdp.1    --------------------------------------------------------------------------------  target    LSDLETGMVYLTAAAKQNRILLEHIQGHALYRSFDEIEREQFDDAMIEAHQLVSMTDLISQILQQLSASYNNILNNNLND 7kdp.1    --------------------------------------------------------------------------------  target    NLTTLTIISVLLAVLAVVTGFFGMNVP--LPLTDEPHAWLYISLASAGLWIVLSLLLRKIAKKS 7kdp.1    ----------VGVAIGAVGGAVASVVEGVATFLKNPFGAFTIILVAIAVVIIIYLIYTR----- ``` | | | | | | | | | | | | | | | | | | | | | | | | | | | | | | | | | | | | | | | | | | | | | | | | | |
|  | 3zsu.1.A | TLL2057 PROTEIN  *Structure of the CyanoQ protein from Thermosynechococcus elongatus* | 0.04 | 0.00 | 12.00 | 0.17 | 126-175 | X-ray | 1.60 | monomer |  | HHblits | 0.24 |
| ``` target    MVLEKQLGNGCTWIDLDLGKLNKLEDLSEIYGLDKETIEYALDRNERAHMDYHRESETVTFIYNVLDVKKDKAYYETFPM 3zsu.1    --------------------------------------------------------------------------------  target    TFIVEHRRLITISNTKNAYVIEQMTRYLENHDTLSIYKFLFASLEIISNAYYPVIEQMDKSRDEVNDLLRQRT----TKK 3zsu.1    ---------------------------------------------TRIQDYLRDIEKNAERFADLEVSVAKGDWQEARNI  target    NLFVLSDLETGMVYLTAAAKQNRILLEHIQGHALYRSFDEIEREQFDDAMIEAHQLVSMTDLISQILQQLSASYNNILNN 3zsu.1    MRGPLGEMLMDMRALNRNL-------------------------------------------------------------  target    NLNDNLTTLTIISVLLAVLAVVTGFFGMNVPLPLTDEPHAWLYISLASAGLWIVLSLLLRKIAKKS 3zsu.1    ------------------------------------------------------------------ ``` | | | | | | | | | | | | | | | | | | | | | | | | | | | | | | | | | | | | | | | | | | | | | | | | | |
|  | 4eij.1.A | P protein  *Structure of the Mumps virus phosphoprotein oligomerization domain* | 0.03 | 0.00 | 6.52 | 0.15 | 128-175 | X-ray | 2.20 | homo-tetramer |  | HHblits | 0.27 |
| ``` target    MVLEKQLGNGCTWIDLDLGKLNKLEDLSEIYGLDKETIEYALDRNERAHMDYHRESETVTFIYNVLDVKKDKAYYETFPM 4eij.1    --------------------------------------------------------------------------------  target    TFIVEHRRLITISNTKNAYVIEQMTRYLENHDTLSIYKFLFASLEIISNAYYPVIEQMDKSRDEVNDLLRQRTTKKNLFV 4eij.1    -----------------------------------------------ANEIMDLLRGMDARLQHLEQKVDKVL--AQGSM  target    LSDLETGMVYLTAAAKQNRILLEHIQGHALYRSFDEIEREQFDDAMIEAHQLVSMTDLISQILQQLSASYNNILNNNLND 4eij.1    VTQIKNELSTVKTTL-----------------------------------------------------------------  target    NLTTLTIISVLLAVLAVVTGFFGMNVPLPLTDEPHAWLYISLASAGLWIVLSLLLRKIAKKS 4eij.1    -------------------------------------------------------------- ``` | | | | | | | | | | | | | | | | | | | | | | | | | | | | | | | | | | | | | | | | | | | | | | | | | |
|  | 4eij.1.B | P protein  *Structure of the Mumps virus phosphoprotein oligomerization domain* | 0.04 | 0.00 | 6.52 | 0.15 | 128-175 | X-ray | 2.20 | homo-tetramer |  | HHblits | 0.27 |
| ``` target    MVLEKQLGNGCTWIDLDLGKLNKLEDLSEIYGLDKETIEYALDRNERAHMDYHRESETVTFIYNVLDVKKDKAYYETFPM 4eij.1    --------------------------------------------------------------------------------  target    TFIVEHRRLITISNTKNAYVIEQMTRYLENHDTLSIYKFLFASLEIISNAYYPVIEQMDKSRDEVNDLLRQRTTKKNLFV 4eij.1    -----------------------------------------------ANEIMDLLRGMDARLQHLEQKVDKVL--AQGSM  target    LSDLETGMVYLTAAAKQNRILLEHIQGHALYRSFDEIEREQFDDAMIEAHQLVSMTDLISQILQQLSASYNNILNNNLND 4eij.1    VTQIKNELSTVKTTL-----------------------------------------------------------------  target    NLTTLTIISVLLAVLAVVTGFFGMNVPLPLTDEPHAWLYISLASAGLWIVLSLLLRKIAKKS 4eij.1    -------------------------------------------------------------- ``` | | | | | | | | | | | | | | | | | | | | | | | | | | | | | | | | | | | | | | | | | | | | | | | | | |
|  | 2wz7.1.A | UNCHARACTERIZED PROTEIN YBGF  *CRYSTAL STRUCTURE OF THE N-TERMINAL DOMAIN OF E.COLI YBGF* | 0.02 | 0.00 | 10.26 | 0.13 | 137-175 | X-ray | 2.48 | homo-trimer | 1 x AUC | HHblits | 0.26 |
| ``` target    MVLEKQLGNGCTWIDLDLGKLNKLEDLSEIYGLDKETIEYALDRNERAHMDYHRESETVTFIYNVLDVKKDKAYYETFPM 2wz7.1    --------------------------------------------------------------------------------  target    TFIVEHRRLITISNTKNAYVIEQMTRYLENHDTLSIYKFLFASLEIISNAYYPVIEQMDKSRDEVNDLLRQR--TTKKNL 2wz7.1    --------------------------------------------------------SVEDRVTQLERISNAHSQLLTQLQ  target    FVLSDLETGMVYLTAAAKQNRILLEHIQGHALYRSFDEIEREQFDDAMIEAHQLVSMTDLISQILQQLSASYNNILNNNL 2wz7.1    QQLSDNQSDIDSLRGQI---------------------------------------------------------------  target    NDNLTTLTIISVLLAVLAVVTGFFGMNVPLPLTDEPHAWLYISLASAGLWIVLSLLLRKIAKKS 2wz7.1    ---------------------------------------------------------------- ``` | | | | | | | | | | | | | | | | | | | | | | | | | | | | | | | | | | | | | | | | | | | | | | | | | |
|  | 2wz7.1.B | UNCHARACTERIZED PROTEIN YBGF  *CRYSTAL STRUCTURE OF THE N-TERMINAL DOMAIN OF E.COLI YBGF* | 0.02 | 0.00 | 10.26 | 0.13 | 137-175 | X-ray | 2.48 | homo-trimer | 1 x AUC | HHblits | 0.26 |
| ``` target    MVLEKQLGNGCTWIDLDLGKLNKLEDLSEIYGLDKETIEYALDRNERAHMDYHRESETVTFIYNVLDVKKDKAYYETFPM 2wz7.1    --------------------------------------------------------------------------------  target    TFIVEHRRLITISNTKNAYVIEQMTRYLENHDTLSIYKFLFASLEIISNAYYPVIEQMDKSRDEVNDLLRQR--TTKKNL 2wz7.1    --------------------------------------------------------SVEDRVTQLERISNAHSQLLTQLQ  target    FVLSDLETGMVYLTAAAKQNRILLEHIQGHALYRSFDEIEREQFDDAMIEAHQLVSMTDLISQILQQLSASYNNILNNNL 2wz7.1    QQLSDNQSDIDSLRGQI---------------------------------------------------------------  target    NDNLTTLTIISVLLAVLAVVTGFFGMNVPLPLTDEPHAWLYISLASAGLWIVLSLLLRKIAKKS 2wz7.1    ---------------------------------------------------------------- ``` | | | | | | | | | | | | | | | | | | | | | | | | | | | | | | | | | | | | | | | | | | | | | | | | | |
|  | 2wz7.1.C | UNCHARACTERIZED PROTEIN YBGF  *CRYSTAL STRUCTURE OF THE N-TERMINAL DOMAIN OF E.COLI YBGF* | 0.02 | 0.00 | 10.26 | 0.13 | 137-175 | X-ray | 2.48 | homo-trimer | 1 x AUC | HHblits | 0.26 |
| ``` target    MVLEKQLGNGCTWIDLDLGKLNKLEDLSEIYGLDKETIEYALDRNERAHMDYHRESETVTFIYNVLDVKKDKAYYETFPM 2wz7.1    --------------------------------------------------------------------------------  target    TFIVEHRRLITISNTKNAYVIEQMTRYLENHDTLSIYKFLFASLEIISNAYYPVIEQMDKSRDEVNDLLRQR--TTKKNL 2wz7.1    --------------------------------------------------------SVEDRVTQLERISNAHSQLLTQLQ  target    FVLSDLETGMVYLTAAAKQNRILLEHIQGHALYRSFDEIEREQFDDAMIEAHQLVSMTDLISQILQQLSASYNNILNNNL 2wz7.1    QQLSDNQSDIDSLRGQI---------------------------------------------------------------  target    NDNLTTLTIISVLLAVLAVVTGFFGMNVPLPLTDEPHAWLYISLASAGLWIVLSLLLRKIAKKS 2wz7.1    ---------------------------------------------------------------- ``` | | | | | | | | | | | | | | | | | | | | | | | | | | | | | | | | | | | | | | | | | | | | | | | | | |
|  | 2wz7.2.A | UNCHARACTERIZED PROTEIN YBGF  *CRYSTAL STRUCTURE OF THE N-TERMINAL DOMAIN OF E.COLI YBGF* | 0.02 | 0.00 | 10.26 | 0.13 | 137-175 | X-ray | 2.48 | homo-trimer | 2 x AU | HHblits | 0.26 |
| ``` target    MVLEKQLGNGCTWIDLDLGKLNKLEDLSEIYGLDKETIEYALDRNERAHMDYHRESETVTFIYNVLDVKKDKAYYETFPM 2wz7.2    --------------------------------------------------------------------------------  target    TFIVEHRRLITISNTKNAYVIEQMTRYLENHDTLSIYKFLFASLEIISNAYYPVIEQMDKSRDEVNDLLRQR--TTKKNL 2wz7.2    --------------------------------------------------------SVEDRVTQLERISNAHSQLLTQLQ  target    FVLSDLETGMVYLTAAAKQNRILLEHIQGHALYRSFDEIEREQFDDAMIEAHQLVSMTDLISQILQQLSASYNNILNNNL 2wz7.2    QQLSDNQSDIDSLRGQI---------------------------------------------------------------  target    NDNLTTLTIISVLLAVLAVVTGFFGMNVPLPLTDEPHAWLYISLASAGLWIVLSLLLRKIAKKS 2wz7.2    ---------------------------------------------------------------- ``` | | | | | | | | | | | | | | | | | | | | | | | | | | | | | | | | | | | | | | | | | | | | | | | | | |
|  | 2wz7.2.B | UNCHARACTERIZED PROTEIN YBGF  *CRYSTAL STRUCTURE OF THE N-TERMINAL DOMAIN OF E.COLI YBGF* | 0.02 | 0.00 | 10.26 | 0.13 | 137-175 | X-ray | 2.48 | homo-trimer | 2 x AU | HHblits | 0.26 |
| ``` target    MVLEKQLGNGCTWIDLDLGKLNKLEDLSEIYGLDKETIEYALDRNERAHMDYHRESETVTFIYNVLDVKKDKAYYETFPM 2wz7.2    --------------------------------------------------------------------------------  target    TFIVEHRRLITISNTKNAYVIEQMTRYLENHDTLSIYKFLFASLEIISNAYYPVIEQMDKSRDEVNDLLRQR--TTKKNL 2wz7.2    --------------------------------------------------------SVEDRVTQLERISNAHSQLLTQLQ  target    FVLSDLETGMVYLTAAAKQNRILLEHIQGHALYRSFDEIEREQFDDAMIEAHQLVSMTDLISQILQQLSASYNNILNNNL 2wz7.2    QQLSDNQSDIDSLRGQI---------------------------------------------------------------  target    NDNLTTLTIISVLLAVLAVVTGFFGMNVPLPLTDEPHAWLYISLASAGLWIVLSLLLRKIAKKS 2wz7.2    ---------------------------------------------------------------- ``` | | | | | | | | | | | | | | | | | | | | | | | | | | | | | | | | | | | | | | | | | | | | | | | | | |
|  | 2wz7.2.C | UNCHARACTERIZED PROTEIN YBGF  *CRYSTAL STRUCTURE OF THE N-TERMINAL DOMAIN OF E.COLI YBGF* | 0.02 | 0.00 | 10.26 | 0.13 | 137-175 | X-ray | 2.48 | homo-trimer | 2 x AU | HHblits | 0.26 |
| ``` target    MVLEKQLGNGCTWIDLDLGKLNKLEDLSEIYGLDKETIEYALDRNERAHMDYHRESETVTFIYNVLDVKKDKAYYETFPM 2wz7.2    --------------------------------------------------------------------------------  target    TFIVEHRRLITISNTKNAYVIEQMTRYLENHDTLSIYKFLFASLEIISNAYYPVIEQMDKSRDEVNDLLRQR--TTKKNL 2wz7.2    --------------------------------------------------------SVEDRVTQLERISNAHSQLLTQLQ  target    FVLSDLETGMVYLTAAAKQNRILLEHIQGHALYRSFDEIEREQFDDAMIEAHQLVSMTDLISQILQQLSASYNNILNNNL 2wz7.2    QQLSDNQSDIDSLRGQI---------------------------------------------------------------  target    NDNLTTLTIISVLLAVLAVVTGFFGMNVPLPLTDEPHAWLYISLASAGLWIVLSLLLRKIAKKS 2wz7.2    ---------------------------------------------------------------- ``` | | | | | | | | | | | | | | | | | | | | | | | | | | | | | | | | | | | | | | | | | | | | | | | | | |
|  | 6znl.1.O | Dynactin subunit 3  *Cryo-EM structure of the dynactin complex* | 0.02 | 0.00 | 7.69 | 0.13 | 133-171 | EM | 0.00 | monomer | 9 x ADP, 1 x ATP, 3 x ZN | HHblits | 0.26 |
| ``` target    MVLEKQLGNGCTWIDLDLGKLNKLEDLSEIYGLDKETIEYALDRNERAHMDYHRESETVTFIYNVLDVKKDKAYYETFPM 6znl.1    --------------------------------------------------------------------------------  target    TFIVEHRRLITISNTKNAYVIEQMTRYLENHDTLSIYKFLFASLEIISNAYYPVIEQMDKSRDEVNDLLRQRTT----KK 6znl.1    ----------------------------------------------------TDVQRLQARLEELERWVYGPGGSRGSRK  target    NLFVLSDLETGMVYLTAAAKQNRILLEHIQGHALYRSFDEIEREQFDDAMIEAHQLVSMTDLISQILQQLSASYNNILNN 6znl.1    VADGLVKVQVALGNI-----------------------------------------------------------------  target    NLNDNLTTLTIISVLLAVLAVVTGFFGMNVPLPLTDEPHAWLYISLASAGLWIVLSLLLRKIAKKS 6znl.1    ------------------------------------------------------------------ ``` | | | | | | | | | | | | | | | | | | | | | | | | | | | | | | | | | | | | | | | | | | | | | | | | | |
|  | 6znl.1.V | Dynactin subunit 3  *Cryo-EM structure of the dynactin complex* | 0.03 | 0.00 | 7.69 | 0.13 | 133-171 | EM | 0.00 | monomer | 9 x ADP, 1 x ATP, 3 x ZN | HHblits | 0.26 |
| ``` target    MVLEKQLGNGCTWIDLDLGKLNKLEDLSEIYGLDKETIEYALDRNERAHMDYHRESETVTFIYNVLDVKKDKAYYETFPM 6znl.1    --------------------------------------------------------------------------------  target    TFIVEHRRLITISNTKNAYVIEQMTRYLENHDTLSIYKFLFASLEIISNAYYPVIEQMDKSRDEVNDLLRQRTT----KK 6znl.1    ----------------------------------------------------TDVQRLQARLEELERWVYGPGGSRGSRK  target    NLFVLSDLETGMVYLTAAAKQNRILLEHIQGHALYRSFDEIEREQFDDAMIEAHQLVSMTDLISQILQQLSASYNNILNN 6znl.1    VADGLVKVQVALGNI-----------------------------------------------------------------  target    NLNDNLTTLTIISVLLAVLAVVTGFFGMNVPLPLTDEPHAWLYISLASAGLWIVLSLLLRKIAKKS 6znl.1    ------------------------------------------------------------------ ``` | | | | | | | | | | | | | | | | | | | | | | | | | | | | | | | | | | | | | | | | | | | | | | | | | |
|  | 6y07.1.A | sohair  *Designing a Granulopoietic Protein by Topological Rescaffolding 1: Sohair* | 0.03 | 0.00 | 17.14 | 0.12 | 115-150 | NMR | 0.00 | monomer |  | HHblits | 0.32 |
| ``` target    MVLEKQLGNGCTWIDLDLGKLNKLEDLSEIYGLDKETIEYALDRNERAHMDYHRESETVTFIYNVLDVKKDKAYYETFPM 6y07.1    --------------------------------------------------------------------------------  target    TFIVEHRRLITISNTKNAYVIEQMTRYLENHDTLSIYKFLFASLEIISNAYYPVIEQMDKSRDEVNDLLRQRTTKKNLFV 6y07.1    ----------------------------------SSDQLLNLILD-LADIITTLIQIIEESNEAIKELIK----------  target    LSDLETGMVYLTAAAKQNRILLEHIQGHALYRSFDEIEREQFDDAMIEAHQLVSMTDLISQILQQLSASYNNILNNNLND 6y07.1    --------------------------------------------------------------------------------  target    NLTTLTIISVLLAVLAVVTGFFGMNVPLPLTDEPHAWLYISLASAGLWIVLSLLLRKIAKKS 6y07.1    -------------------------------------------------------------- ``` | | | | | | | | | | | | | | | | | | | | | | | | | | | | | | | | | | | | | | | | | | | | | | | | | |
|  | 6btm.1.C | Alternative Complex III subunit C  *Structure of Alternative Complex III from Flavobacterium johnsoniae (Wild Type)* | 0.02 | 0.00 | 13.51 | 0.12 | 237-274 | EM | 3.40 | monomer | 6 x HEC, 1 x F3S, 1 x SF4, 2 x E87 | HHblits | 0.28 |
| ``` target    MVLEKQLGNGCTWIDLDLGKLNKLEDLSEIYGLDKETIEYALDRNERAHMDYHRESETVTFIYNVLDVKKDKAYYETFPM 6btm.1    --------------------------------------------------------------------------------  target    TFIVEHRRLITISNTKNAYVIEQMTRYLENHDTLSIYKFLFASLEIISNAYYPVIEQMDKSRDEVNDLLRQRTTKKNLFV 6btm.1    --------------------------------------------------------------------------------  target    LSDLETGMVYLTAAAKQNRILLEHIQGHALYRSFDEIEREQFDDAMIEAHQLVSMTDLISQILQQLSASYNNILNNNLND 6btm.1    ----------------------------------------------------------------------------RFEE  target    NLTTLTIISVLLAVL--AVVTGFFGMNVPLPLTDEPHAWLYISLASAGLWIVLSLLLRKIAKKS 6btm.1    VSLVLAGLATPLVLSVHTIVSMDFATS-VIPGWHTT---------------------------- ``` | | | | | | | | | | | | | | | | | | | | | | | | | | | | | | | | | | | | | | | | | | | | | | | | | |
|  | 6lod.1.C | Polysulphide reductase NrfD  *Cryo-EM structure of the air-oxidized photosynthetic alternative complex III from Roseiflexus castenholzii* | 0.02 | 0.00 | 16.22 | 0.12 | 237-274 | EM | 0.00 | monomer | 6 x HEC, 2 x EL6, 3 x SF4, 1 x F3S | HHblits | 0.27 |
| ``` target    MVLEKQLGNGCTWIDLDLGKLNKLEDLSEIYGLDKETIEYALDRNERAHMDYHRESETVTFIYNVLDVKKDKAYYETFPM 6lod.1    --------------------------------------------------------------------------------  target    TFIVEHRRLITISNTKNAYVIEQMTRYLENHDTLSIYKFLFASLEIISNAYYPVIEQMDKSRDEVNDLLRQRTTKKNLFV 6lod.1    --------------------------------------------------------------------------------  target    LSDLETGMVYLTAAAKQNRILLEHIQGHALYRSFDEIEREQFDDAMIEAHQLVSMTDLISQILQQLSASYNNILNNNLND 6lod.1    ----------------------------------------------------------------------------RYEV  target    NLTTLTIISVLLAV--LAVVTGFFGMNVPLPLTDEPHAWLYISLASAGLWIVLSLLLRKIAKKS 6lod.1    ASLILAGLSTPLVLSVHSIISLDFAIS-QLPGWHVT---------------------------- ``` | | | | | | | | | | | | | | | | | | | | | | | | | | | | | | | | | | | | | | | | | | | | | | | | | |
|  | 6f0k.1.C | Polysulphide reductase NrfD  *Alternative complex III* | 0.02 |  | 16.67 | 0.12 | 238-274 | EM | 0.00 | hetero-1-1-1-1-1-1-… | 6 x HEC, 1 x F3S, 3 x SF4 | HHblits | 0.29 |
| ``` target    MVLEKQLGNGCTWIDLDLGKLNKLEDLSEIYGLDKETIEYALDRNERAHMDYHRESETVTFIYNVLDVKKDKAYYETFPM 6f0k.1    --------------------------------------------------------------------------------  target    TFIVEHRRLITISNTKNAYVIEQMTRYLENHDTLSIYKFLFASLEIISNAYYPVIEQMDKSRDEVNDLLRQRTTKKNLFV 6f0k.1    --------------------------------------------------------------------------------  target    LSDLETGMVYLTAAAKQNRILLEHIQGHALYRSFDEIEREQFDDAMIEAHQLVSMTDLISQILQQLSASYNNILNNNLND 6f0k.1    -----------------------------------------------------------------------------YEK  target    NLTTLTIISVLLAVL--AVVTGFFGMNVPLPLTDEPHAWLYISLASAGLWIVLSLLLRKIAKKS 6f0k.1    VYMLLAGLATPLVLSVHSVVSFDFAVSI-IPGWHTT---------------------------- ``` | | | | | | | | | | | | | | | | | | | | | | | | | | | | | | | | | | | | | | | | | | | | | | | | | |
|  | 6xns.1.A | C3\_crown-05  *C3\_crown-05* | 0.03 |  | 17.14 | 0.12 | 130-164 | X-ray | 3.19 | homo-trimer |  | HHblits | 0.31 |
| ``` target    MVLEKQLGNGCTWIDLDLGKLNKLEDLSEIYGLDKETIEYALDRNERAHMDYHRESETVTFIYNVLDVKKDKAYYETFPM 6xns.1    --------------------------------------------------------------------------------  target    TFIVEHRRLITISNTKNAYVIEQMTRYLENHDTLSIYKFLFASLEIISNAYYPVIEQMDKSRDEVNDLLRQRTTKKNLFV 6xns.1    -------------------------------------------------NLRRHLDRLDKHIKQLRDILSENPEDERVKD  target    LSDLETGMVYLTAAAKQNRILLEHIQGHALYRSFDEIEREQFDDAMIEAHQLVSMTDLISQILQQLSASYNNILNNNLND 6xns.1    VIDL----------------------------------------------------------------------------  target    NLTTLTIISVLLAVLAVVTGFFGMNVPLPLTDEPHAWLYISLASAGLWIVLSLLLRKIAKKS 6xns.1    -------------------------------------------------------------- ``` | | | | | | | | | | | | | | | | | | | | | | | | | | | | | | | | | | | | | | | | | | | | | | | | | |
|  | 6xns.1.B | C3\_crown-05  *C3\_crown-05* | 0.03 |  | 17.14 | 0.12 | 130-164 | X-ray | 3.19 | homo-trimer |  | HHblits | 0.31 |
| ``` target    MVLEKQLGNGCTWIDLDLGKLNKLEDLSEIYGLDKETIEYALDRNERAHMDYHRESETVTFIYNVLDVKKDKAYYETFPM 6xns.1    --------------------------------------------------------------------------------  target    TFIVEHRRLITISNTKNAYVIEQMTRYLENHDTLSIYKFLFASLEIISNAYYPVIEQMDKSRDEVNDLLRQRTTKKNLFV 6xns.1    -------------------------------------------------NLRRHLDRLDKHIKQLRDILSENPEDERVKD  target    LSDLETGMVYLTAAAKQNRILLEHIQGHALYRSFDEIEREQFDDAMIEAHQLVSMTDLISQILQQLSASYNNILNNNLND 6xns.1    VIDL----------------------------------------------------------------------------  target    NLTTLTIISVLLAVLAVVTGFFGMNVPLPLTDEPHAWLYISLASAGLWIVLSLLLRKIAKKS 6xns.1    -------------------------------------------------------------- ``` | | | | | | | | | | | | | | | | | | | | | | | | | | | | | | | | | | | | | | | | | | | | | | | | | |
|  | 6xns.1.C | C3\_crown-05  *C3\_crown-05* | 0.03 |  | 17.14 | 0.12 | 130-164 | X-ray | 3.19 | homo-trimer |  | HHblits | 0.31 |
| ``` target    MVLEKQLGNGCTWIDLDLGKLNKLEDLSEIYGLDKETIEYALDRNERAHMDYHRESETVTFIYNVLDVKKDKAYYETFPM 6xns.1    --------------------------------------------------------------------------------  target    TFIVEHRRLITISNTKNAYVIEQMTRYLENHDTLSIYKFLFASLEIISNAYYPVIEQMDKSRDEVNDLLRQRTTKKNLFV 6xns.1    -------------------------------------------------NLRRHLDRLDKHIKQLRDILSENPEDERVKD  target    LSDLETGMVYLTAAAKQNRILLEHIQGHALYRSFDEIEREQFDDAMIEAHQLVSMTDLISQILQQLSASYNNILNNNLND 6xns.1    VIDL----------------------------------------------------------------------------  target    NLTTLTIISVLLAVLAVVTGFFGMNVPLPLTDEPHAWLYISLASAGLWIVLSLLLRKIAKKS 6xns.1    -------------------------------------------------------------- ``` | | | | | | | | | | | | | | | | | | | | | | | | | | | | | | | | | | | | | | | | | | | | | | | | | |
|  | 6xns.2.A | C3\_crown-05  *C3\_crown-05* | 0.03 |  | 17.14 | 0.12 | 130-164 | X-ray | 3.19 | homo-trimer |  | HHblits | 0.31 |
| ``` target    MVLEKQLGNGCTWIDLDLGKLNKLEDLSEIYGLDKETIEYALDRNERAHMDYHRESETVTFIYNVLDVKKDKAYYETFPM 6xns.2    --------------------------------------------------------------------------------  target    TFIVEHRRLITISNTKNAYVIEQMTRYLENHDTLSIYKFLFASLEIISNAYYPVIEQMDKSRDEVNDLLRQRTTKKNLFV 6xns.2    -------------------------------------------------NLRRHLDRLDKHIKQLRDILSENPEDERVKD  target    LSDLETGMVYLTAAAKQNRILLEHIQGHALYRSFDEIEREQFDDAMIEAHQLVSMTDLISQILQQLSASYNNILNNNLND 6xns.2    VIDL----------------------------------------------------------------------------  target    NLTTLTIISVLLAVLAVVTGFFGMNVPLPLTDEPHAWLYISLASAGLWIVLSLLLRKIAKKS 6xns.2    -------------------------------------------------------------- ``` | | | | | | | | | | | | | | | | | | | | | | | | | | | | | | | | | | | | | | | | | | | | | | | | | |
|  | 6xns.2.B | C3\_crown-05  *C3\_crown-05* | 0.02 |  | 17.14 | 0.12 | 130-164 | X-ray | 3.19 | homo-trimer |  | HHblits | 0.31 |
| ``` target    MVLEKQLGNGCTWIDLDLGKLNKLEDLSEIYGLDKETIEYALDRNERAHMDYHRESETVTFIYNVLDVKKDKAYYETFPM 6xns.2    --------------------------------------------------------------------------------  target    TFIVEHRRLITISNTKNAYVIEQMTRYLENHDTLSIYKFLFASLEIISNAYYPVIEQMDKSRDEVNDLLRQRTTKKNLFV 6xns.2    -------------------------------------------------NLRRHLDRLDKHIKQLRDILSENPEDERVKD  target    LSDLETGMVYLTAAAKQNRILLEHIQGHALYRSFDEIEREQFDDAMIEAHQLVSMTDLISQILQQLSASYNNILNNNLND 6xns.2    VIDL----------------------------------------------------------------------------  target    NLTTLTIISVLLAVLAVVTGFFGMNVPLPLTDEPHAWLYISLASAGLWIVLSLLLRKIAKKS 6xns.2    -------------------------------------------------------------- ``` | | | | | | | | | | | | | | | | | | | | | | | | | | | | | | | | | | | | | | | | | | | | | | | | | |
|  | 6xns.2.C | C3\_crown-05  *C3\_crown-05* | 0.03 |  | 17.14 | 0.12 | 130-164 | X-ray | 3.19 | homo-trimer |  | HHblits | 0.31 |
| ``` target    MVLEKQLGNGCTWIDLDLGKLNKLEDLSEIYGLDKETIEYALDRNERAHMDYHRESETVTFIYNVLDVKKDKAYYETFPM 6xns.2    --------------------------------------------------------------------------------  target    TFIVEHRRLITISNTKNAYVIEQMTRYLENHDTLSIYKFLFASLEIISNAYYPVIEQMDKSRDEVNDLLRQRTTKKNLFV 6xns.2    -------------------------------------------------NLRRHLDRLDKHIKQLRDILSENPEDERVKD  target    LSDLETGMVYLTAAAKQNRILLEHIQGHALYRSFDEIEREQFDDAMIEAHQLVSMTDLISQILQQLSASYNNILNNNLND 6xns.2    VIDL----------------------------------------------------------------------------  target    NLTTLTIISVLLAVLAVVTGFFGMNVPLPLTDEPHAWLYISLASAGLWIVLSLLLRKIAKKS 6xns.2    -------------------------------------------------------------- ``` | | | | | | | | | | | | | | | | | | | | | | | | | | | | | | | | | | | | | | | | | | | | | | | | | |
|  | 6ysl.1.A | Motility protein A  *Structure of the flagellar MotAB stator complex from Bacillus subtilis* | 0.02 |  | 11.11 | 0.12 | 232-267 | EM | 0.00 | hetero-5-2-mer |  | HHblits | 0.27 |
| ``` target    MVLEKQLGNGCTWIDLDLGKLNKLEDLSEIYGLDKETIEYALDRNERAHMDYHRESETVTFIYNVLDVKKDKAYYETFPM 6ysl.1    --------------------------------------------------------------------------------  target    TFIVEHRRLITISNTKNAYVIEQMTRYLENHDTLSIYKFLFASLEIISNAYYPVIEQMDKSRDEVNDLLRQRTTKKNLFV 6ysl.1    --------------------------------------------------------------------------------  target    LSDLETGMVYLTAAAKQNRILLEHIQGHALYRSFDEIEREQFDDAMIEAHQLVSMTDLISQILQQLSASYNNILNNNLND 6ysl.1    -----------------------------------------------------------------------EAMEDRHQA  target    NLTTLTIISVLLAVLAVVTGFFGMNVPLPLTDEPHAWLYISLASAGLWIVLSLLLRKIAKKS 6ysl.1    GAAIFTQAGTYAPTLGVLGAVIGLIAA----------------------------------- ``` | | | | | | | | | | | | | | | | | | | | | | | | | | | | | | | | | | | | | | | | | | | | | | | | | |
|  | 6ysl.1.D | Motility protein A  *Structure of the flagellar MotAB stator complex from Bacillus subtilis* | 0.02 |  | 11.11 | 0.12 | 232-267 | EM | 0.00 | hetero-5-2-mer |  | HHblits | 0.27 |
| ``` target    MVLEKQLGNGCTWIDLDLGKLNKLEDLSEIYGLDKETIEYALDRNERAHMDYHRESETVTFIYNVLDVKKDKAYYETFPM 6ysl.1    --------------------------------------------------------------------------------  target    TFIVEHRRLITISNTKNAYVIEQMTRYLENHDTLSIYKFLFASLEIISNAYYPVIEQMDKSRDEVNDLLRQRTTKKNLFV 6ysl.1    --------------------------------------------------------------------------------  target    LSDLETGMVYLTAAAKQNRILLEHIQGHALYRSFDEIEREQFDDAMIEAHQLVSMTDLISQILQQLSASYNNILNNNLND 6ysl.1    -----------------------------------------------------------------------EAMEDRHQA  target    NLTTLTIISVLLAVLAVVTGFFGMNVPLPLTDEPHAWLYISLASAGLWIVLSLLLRKIAKKS 6ysl.1    GAAIFTQAGTYAPTLGVLGAVIGLIAA----------------------------------- ``` | | | | | | | | | | | | | | | | | | | | | | | | | | | | | | | | | | | | | | | | | | | | | | | | | |
|  | 6ysl.1.E | Motility protein A  *Structure of the flagellar MotAB stator complex from Bacillus subtilis* | 0.02 |  | 11.11 | 0.12 | 232-267 | EM | 0.00 | hetero-5-2-mer |  | HHblits | 0.27 |
| ``` target    MVLEKQLGNGCTWIDLDLGKLNKLEDLSEIYGLDKETIEYALDRNERAHMDYHRESETVTFIYNVLDVKKDKAYYETFPM 6ysl.1    --------------------------------------------------------------------------------  target    TFIVEHRRLITISNTKNAYVIEQMTRYLENHDTLSIYKFLFASLEIISNAYYPVIEQMDKSRDEVNDLLRQRTTKKNLFV 6ysl.1    --------------------------------------------------------------------------------  target    LSDLETGMVYLTAAAKQNRILLEHIQGHALYRSFDEIEREQFDDAMIEAHQLVSMTDLISQILQQLSASYNNILNNNLND 6ysl.1    -----------------------------------------------------------------------EAMEDRHQA  target    NLTTLTIISVLLAVLAVVTGFFGMNVPLPLTDEPHAWLYISLASAGLWIVLSLLLRKIAKKS 6ysl.1    GAAIFTQAGTYAPTLGVLGAVIGLIAA----------------------------------- ``` | | | | | | | | | | | | | | | | | | | | | | | | | | | | | | | | | | | | | | | | | | | | | | | | | |
|  | 6ysl.1.F | Motility protein A  *Structure of the flagellar MotAB stator complex from Bacillus subtilis* | 0.02 |  | 11.11 | 0.12 | 232-267 | EM | 0.00 | hetero-5-2-mer |  | HHblits | 0.27 |
| ``` target    MVLEKQLGNGCTWIDLDLGKLNKLEDLSEIYGLDKETIEYALDRNERAHMDYHRESETVTFIYNVLDVKKDKAYYETFPM 6ysl.1    --------------------------------------------------------------------------------  target    TFIVEHRRLITISNTKNAYVIEQMTRYLENHDTLSIYKFLFASLEIISNAYYPVIEQMDKSRDEVNDLLRQRTTKKNLFV 6ysl.1    --------------------------------------------------------------------------------  target    LSDLETGMVYLTAAAKQNRILLEHIQGHALYRSFDEIEREQFDDAMIEAHQLVSMTDLISQILQQLSASYNNILNNNLND 6ysl.1    -----------------------------------------------------------------------EAMEDRHQA  target    NLTTLTIISVLLAVLAVVTGFFGMNVPLPLTDEPHAWLYISLASAGLWIVLSLLLRKIAKKS 6ysl.1    GAAIFTQAGTYAPTLGVLGAVIGLIAA----------------------------------- ``` | | | | | | | | | | | | | | | | | | | | | | | | | | | | | | | | | | | | | | | | | | | | | | | | | |
|  | 6ysl.1.G | Motility protein A  *Structure of the flagellar MotAB stator complex from Bacillus subtilis* | 0.02 |  | 11.11 | 0.12 | 232-267 | EM | 0.00 | hetero-5-2-mer |  | HHblits | 0.27 |
| ``` target    MVLEKQLGNGCTWIDLDLGKLNKLEDLSEIYGLDKETIEYALDRNERAHMDYHRESETVTFIYNVLDVKKDKAYYETFPM 6ysl.1    --------------------------------------------------------------------------------  target    TFIVEHRRLITISNTKNAYVIEQMTRYLENHDTLSIYKFLFASLEIISNAYYPVIEQMDKSRDEVNDLLRQRTTKKNLFV 6ysl.1    --------------------------------------------------------------------------------  target    LSDLETGMVYLTAAAKQNRILLEHIQGHALYRSFDEIEREQFDDAMIEAHQLVSMTDLISQILQQLSASYNNILNNNLND 6ysl.1    -----------------------------------------------------------------------EAMEDRHQA  target    NLTTLTIISVLLAVLAVVTGFFGMNVPLPLTDEPHAWLYISLASAGLWIVLSLLLRKIAKKS 6ysl.1    GAAIFTQAGTYAPTLGVLGAVIGLIAA----------------------------------- ``` | | | | | | | | | | | | | | | | | | | | | | | | | | | | | | | | | | | | | | | | | | | | | | | | | |
|  | 6hwh.1.K | Cytochrome c oxidase polypeptide 4  *Structure of a functional obligate respiratory supercomplex from Mycobacterium smegmatis* | 0.02 |  | 16.67 | 0.12 | 242-278 | EM | 0.00 | hetero-2-2-2-2-2-4-… | 2 x FES, 8 x CDL, 4 x MQ9, 6 x CU, 4 x HAS, 4 x HEC, 4 x HEM | HHblits | 0.26 |
| ``` target    MVLEKQLGNGCTWIDLDLGKLNKLEDLSEIYGLDKETIEYALDRNERAHMDYHRESETVTFIYNVLDVKKDKAYYETFPM 6hwh.1    --------------------------------------------------------------------------------  target    TFIVEHRRLITISNTKNAYVIEQMTRYLENHDTLSIYKFLFASLEIISNAYYPVIEQMDKSRDEVNDLLRQRTTKKNLFV 6hwh.1    --------------------------------------------------------------------------------  target    LSDLETGMVYLTAAAKQNRILLEHIQGHALYRSFDEIEREQFDDAMIEAHQLVSMTDLISQILQQLSASYNNILNNNLND 6hwh.1    --------------------------------------------------------------------------------  target    NLTTLTIISVLLAVLAVVTGFFGMNVPLPLTDEPHAWLYISLASAGLWIVLSLLLRKIAKKS 6hwh.1    -ARLFEILTAFFALAAVVYAVLTAMFA-TGGVEWAGTT------------------------ ``` | | | | | | | | | | | | | | | | | | | | | | | | | | | | | | | | | | | | | | | | | | | | | | | | | |
|  | 6adq.1.D | Cytochrome c oxidase polypeptide 4  *Respiratory Complex CIII2CIV2SOD2 from Mycobacterium smegmatis* | 0.02 |  | 16.67 | 0.12 | 242-278 | EM | 0.00 | hetero-2-2-2-2-2-2-… | 8 x CU, 4 x HEA, 18 x CDL, 8 x 9Y0, 4 x PLM, 4 x 9XX, 8 x 9YF, 4 x HEM, 10 x MQ9, 4 x HEC, 2 x FES | HHblits | 0.26 |
| ``` target    MVLEKQLGNGCTWIDLDLGKLNKLEDLSEIYGLDKETIEYALDRNERAHMDYHRESETVTFIYNVLDVKKDKAYYETFPM 6adq.1    --------------------------------------------------------------------------------  target    TFIVEHRRLITISNTKNAYVIEQMTRYLENHDTLSIYKFLFASLEIISNAYYPVIEQMDKSRDEVNDLLRQRTTKKNLFV 6adq.1    --------------------------------------------------------------------------------  target    LSDLETGMVYLTAAAKQNRILLEHIQGHALYRSFDEIEREQFDDAMIEAHQLVSMTDLISQILQQLSASYNNILNNNLND 6adq.1    --------------------------------------------------------------------------------  target    NLTTLTIISVLLAVLAVVTGFFGMNVPLPLTDEPHAWLYISLASAGLWIVLSLLLRKIAKKS 6adq.1    -ARLFEILTAFFALAAVVYAVLTAMFA-TGGVEWAGTT------------------------ ``` | | | | | | | | | | | | | | | | | | | | | | | | | | | | | | | | | | | | | | | | | | | | | | | | | |
|  | 6zyw.1.B | Outer arm dynein beta heavy chain  *Outer Dynein Arm-Shulin complex - overall structure (Tetrahymena thermophila)* | 0.03 |  | 5.71 | 0.12 | 119-153 | EM | 0.00 | hetero-1-1-1-2-2-1-… | 3 x ADP, 1 x ATP, 1 x GTP | HHblits | 0.27 |
| ``` target    MVLEKQLGNGCTWIDLDLGKLNKLEDLSEIYGLDKETIEYALDRNERAHMDYHRESETVTFIYNVLDVKKDKAYYETFPM 6zyw.1    --------------------------------------------------------------------------------  target    TFIVEHRRLITISNTKNAYVIEQMTRYLENHDTLSIYKFLFASLEIISNAYYPVIEQMDKSRDEVNDLLRQRTTKKNLFV 6zyw.1    --------------------------------------WIRVYTDFLVNQFRTTQKNLLDFIEKTKDGIKKNP-------  target    LSDLETGMVYLTAAAKQNRILLEHIQGHALYRSFDEIEREQFDDAMIEAHQLVSMTDLISQILQQLSASYNNILNNNLND 6zyw.1    --------------------------------------------------------------------------------  target    NLTTLTIISVLLAVLAVVTGFFGMNVPLPLTDEPHAWLYISLASAGLWIVLSLLLRKIAKKS 6zyw.1    -------------------------------------------------------------- ``` | | | | | | | | | | | | | | | | | | | | | | | | | | | | | | | | | | | | | | | | | | | | | | | | | |
|  | 6dlc.1.A | Designed protein DHD1:234\_A  *Designed protein DHD1:234\_A, Designed protein DHD1:234\_B* | 0.03 |  | 11.76 | 0.11 | 127-160 | X-ray | 3.26 | hetero-2-2-mer |  | HHblits | 0.29 |
| ``` target    MVLEKQLGNGCTWIDLDLGKLNKLEDLSEIYGLDKETIEYALDRNERAHMDYHRESETVTFIYNVLDVKKDKAYYETFPM 6dlc.1    --------------------------------------------------------------------------------  target    TFIVEHRRLITISNTKNAYVIEQMTRYLENHDTLSIYKFLFASLEIISNAYYPVIEQMDKSRDEVNDLLRQRTTKKNLFV 6dlc.1    ----------------------------------------------FLENLRRHLDRLDKHIKQLRDILSENPEDERVKD  target    LSDLETGMVYLTAAAKQNRILLEHIQGHALYRSFDEIEREQFDDAMIEAHQLVSMTDLISQILQQLSASYNNILNNNLND 6dlc.1    --------------------------------------------------------------------------------  target    NLTTLTIISVLLAVLAVVTGFFGMNVPLPLTDEPHAWLYISLASAGLWIVLSLLLRKIAKKS 6dlc.1    -------------------------------------------------------------- ``` | | | | | | | | | | | | | | | | | | | | | | | | | | | | | | | | | | | | | | | | | | | | | | | | | |
|  | 6ncn.1.A | Apolipoprotein E  *Fragment-based Discovery of an apoE4 Stabilizer* | 0.02 |  | 14.29 | 0.12 | 213-247 | X-ray | 1.82 | monomer | 1 x KJM | HHblits | 0.26 |
| ``` target    MVLEKQLGNGCTWIDLDLGKLNKLEDLSEIYGLDKETIEYALDRNERAHMDYHRESETVTFIYNVLDVKKDKAYYETFPM 6ncn.1    --------------------------------------------------------------------------------  target    TFIVEHRRLITISNTKNAYVIEQMTRYLENHDTLSIYKFLFASLEIISNAYYPVIEQMDKSRDEVNDLLRQRTTKKNLFV 6ncn.1    --------------------------------------------------------------------------------  target    LSDLETGMVYLTAAAKQNRILLEHIQGHALYRSFDEIEREQFDDAMIEAHQLVSMTDLISQILQQLSASYNNILNNNLND 6ncn.1    ----------------------------------------------------GQSTEELRVRLASHLRKLRKRLLRDADD  target    NLTTLTIISVLLAVLAVVTGFFGMNVPLPLTDEPHAWLYISLASAGLWIVLSLLLRKIAKKS 6ncn.1    LQKRLAV------------------------------------------------------- ``` | | | | | | | | | | | | | | | | | | | | | | | | | | | | | | | | | | | | | | | | | | | | | | | | | |
|  | 6zyw.1.C | Dynein heavy chain, outer arm protein  *Outer Dynein Arm-Shulin complex - overall structure (Tetrahymena thermophila)* | 0.03 |  | 2.86 | 0.12 | 119-153 | EM | 0.00 | hetero-1-1-1-2-2-1-… | 3 x ADP, 1 x ATP, 1 x GTP | HHblits | 0.25 |
| ``` target    MVLEKQLGNGCTWIDLDLGKLNKLEDLSEIYGLDKETIEYALDRNERAHMDYHRESETVTFIYNVLDVKKDKAYYETFPM 6zyw.1    --------------------------------------------------------------------------------  target    TFIVEHRRLITISNTKNAYVIEQMTRYLENHDTLSIYKFLFASLEIISNAYYPVIEQMDKSRDEVNDLLRQRTTKKNLFV 6zyw.1    --------------------------------------WKLQYSQDLHKRARQLLDSLTEQTKMLSTKLSKPV-------  target    LSDLETGMVYLTAAAKQNRILLEHIQGHALYRSFDEIEREQFDDAMIEAHQLVSMTDLISQILQQLSASYNNILNNNLND 6zyw.1    --------------------------------------------------------------------------------  target    NLTTLTIISVLLAVLAVVTGFFGMNVPLPLTDEPHAWLYISLASAGLWIVLSLLLRKIAKKS 6zyw.1    -------------------------------------------------------------- ``` | | | | | | | | | | | | | | | | | | | | | | | | | | | | | | | | | | | | | | | | | | | | | | | | | |
|  | 1gs9.1.A | APOLIPOPROTEIN E  *APOLIPOPROTEIN E4, 22K DOMAIN* | 0.02 |  | 14.71 | 0.11 | 214-247 | X-ray | 1.70 | monomer |  | HHblits | 0.27 |
| ``` target    MVLEKQLGNGCTWIDLDLGKLNKLEDLSEIYGLDKETIEYALDRNERAHMDYHRESETVTFIYNVLDVKKDKAYYETFPM 1gs9.1    --------------------------------------------------------------------------------  target    TFIVEHRRLITISNTKNAYVIEQMTRYLENHDTLSIYKFLFASLEIISNAYYPVIEQMDKSRDEVNDLLRQRTTKKNLFV 1gs9.1    --------------------------------------------------------------------------------  target    LSDLETGMVYLTAAAKQNRILLEHIQGHALYRSFDEIEREQFDDAMIEAHQLVSMTDLISQILQQLSASYNNILNNNLND 1gs9.1    -----------------------------------------------------QSTEELRVRLASHLRKLRKRLLRDADD  target    NLTTLTIISVLLAVLAVVTGFFGMNVPLPLTDEPHAWLYISLASAGLWIVLSLLLRKIAKKS 1gs9.1    LQKRLAV------------------------------------------------------- ``` | | | | | | | | | | | | | | | | | | | | | | | | | | | | | | | | | | | | | | | | | | | | | | | | | |
|  | 1le2.1.A | APOLIPOPROTEIN E2  *STRUCTURAL BASIS FOR ALTERED FUNCTION IN THE COMMON MUTANTS OF HUMAN APOLIPOPROTEIN-E* | 0.01 |  | 14.71 | 0.11 | 213-246 | X-ray | 3.00 | monomer |  | HHblits | 0.25 |
| ``` target    MVLEKQLGNGCTWIDLDLGKLNKLEDLSEIYGLDKETIEYALDRNERAHMDYHRESETVTFIYNVLDVKKDKAYYETFPM 1le2.1    --------------------------------------------------------------------------------  target    TFIVEHRRLITISNTKNAYVIEQMTRYLENHDTLSIYKFLFASLEIISNAYYPVIEQMDKSRDEVNDLLRQRTTKKNLFV 1le2.1    --------------------------------------------------------------------------------  target    LSDLETGMVYLTAAAKQNRILLEHIQGHALYRSFDEIEREQFDDAMIEAHQLVSMTDLISQILQQLSASYNNILNNNLND 1le2.1    ----------------------------------------------------GQSTEELRVRLASHLRKLRKRLLRDADD  target    NLTTLTIISVLLAVLAVVTGFFGMNVPLPLTDEPHAWLYISLASAGLWIVLSLLLRKIAKKS 1le2.1    LQKCLA-------------------------------------------------------- ``` | | | | | | | | | | | | | | | | | | | | | | | | | | | | | | | | | | | | | | | | | | | | | | | | | |
|  | 1lpe.1.A | APOLIPOPROTEIN E3  *THREE-DIMENSIONAL STRUCTURE OF THE LDL RECEPTOR-BINDING DOMAIN OF HUMAN APOLIPOPROTEIN E* | 0.01 |  | 14.71 | 0.11 | 213-246 | X-ray | 2.25 | monomer |  | HHblits | 0.25 |
| ``` target    MVLEKQLGNGCTWIDLDLGKLNKLEDLSEIYGLDKETIEYALDRNERAHMDYHRESETVTFIYNVLDVKKDKAYYETFPM 1lpe.1    --------------------------------------------------------------------------------  target    TFIVEHRRLITISNTKNAYVIEQMTRYLENHDTLSIYKFLFASLEIISNAYYPVIEQMDKSRDEVNDLLRQRTTKKNLFV 1lpe.1    --------------------------------------------------------------------------------  target    LSDLETGMVYLTAAAKQNRILLEHIQGHALYRSFDEIEREQFDDAMIEAHQLVSMTDLISQILQQLSASYNNILNNNLND 1lpe.1    ----------------------------------------------------GQSTEELRVRLASHLRKLRKRLLRDADD  target    NLTTLTIISVLLAVLAVVTGFFGMNVPLPLTDEPHAWLYISLASAGLWIVLSLLLRKIAKKS 1lpe.1    LQKRLA-------------------------------------------------------- ``` | | | | | | | | | | | | | | | | | | | | | | | | | | | | | | | | | | | | | | | | | | | | | | | | | |
|  | 6bu5.1.A | Divalent metal cation transporter MntH  *Crystal structure of the Deinococcus radiodurans Nramp/MntH divalent transition metal transporter in the outward-open, manganese-bound conformation* | 0.02 |  | 11.43 | 0.12 | 237-271 | X-ray | 2.40 | monomer | 2 x MN, 1 x SPD, 9 x OLC | HHblits | 0.23 |
| ``` target    MVLEKQLGNGCTWIDLDLGKLNKLEDLSEIYGLDKETIEYALDRNERAHMDYHRESETVTFIYNVLDVKKDKAYYETFPM 6bu5.1    --------------------------------------------------------------------------------  target    TFIVEHRRLITISNTKNAYVIEQMTRYLENHDTLSIYKFLFASLEIISNAYYPVIEQMDKSRDEVNDLLRQRTTKKNLFV 6bu5.1    --------------------------------------------------------------------------------  target    LSDLETGMVYLTAAAKQNRILLEHIQGHALYRSFDEIEREQFDDAMIEAHQLVSMTDLISQILQQLSASYNNILNNNLND 6bu5.1    ----------------------------------------------------------------------------DPSS  target    NLTTLTIISVLLAVLAVVTGFFGMNVP-LPLTDEPHAWLYISLASAGLWIVLSLLLRKIAKKS 6bu5.1    VLILSQVILCFGVPFALVPLLLFTARRDVMGA------------------------------- ``` | | | | | | | | | | | | | | | | | | | | | | | | | | | | | | | | | | | | | | | | | | | | | | | | | |
|  | 6d91.1.A | Divalent metal cation transporter MntH  *Crystal structure of the Deinococcus radiodurans Nramp/MntH divalent transition metal transporter in the outward-open, apo conformation* | 0.02 |  | 11.43 | 0.12 | 237-271 | X-ray | 2.36 | monomer | 6 x OLC | HHblits | 0.23 |
| ``` target    MVLEKQLGNGCTWIDLDLGKLNKLEDLSEIYGLDKETIEYALDRNERAHMDYHRESETVTFIYNVLDVKKDKAYYETFPM 6d91.1    --------------------------------------------------------------------------------  target    TFIVEHRRLITISNTKNAYVIEQMTRYLENHDTLSIYKFLFASLEIISNAYYPVIEQMDKSRDEVNDLLRQRTTKKNLFV 6d91.1    --------------------------------------------------------------------------------  target    LSDLETGMVYLTAAAKQNRILLEHIQGHALYRSFDEIEREQFDDAMIEAHQLVSMTDLISQILQQLSASYNNILNNNLND 6d91.1    ----------------------------------------------------------------------------DPSS  target    NLTTLTIISVLLAVLAVVTGFFGMNVP-LPLTDEPHAWLYISLASAGLWIVLSLLLRKIAKKS 6d91.1    VLILSQVILCFGVPFALVPLLLFTARRDVMGA------------------------------- ``` | | | | | | | | | | | | | | | | | | | | | | | | | | | | | | | | | | | | | | | | | | | | | | | | | |
|  | 6dlm.1.A | DHD127\_A  *DHD127* | 0.02 |  | 19.35 | 0.10 | 176-210 | X-ray | 1.75 | hetero-1-1-mer |  | HHblits | 0.32 |
| ``` target    MVLEKQLGNGCTWIDLDLGKLNKLEDLSEIYGLDKETIEYALDRNERAHMDYHRESETVTFIYNVLDVKKDKAYYETFPM 6dlm.1    --------------------------------------------------------------------------------  target    TFIVEHRRLITISNTKNAYVIEQMTRYLENHDTLSIYKFLFASLEIISNAYYPVIEQMDKSRDEVNDLLRQRTTKKNLFV 6dlm.1    --------------------------------------------------------------------------------  target    LSDLETGMVYLTAAAKQNRILLEHIQGHALYRSFDEIEREQFDDAMIEAHQLVSMTDLISQILQQLSASYNNILNNNLND 6dlm.1    ---------------RESERVVEVLERGE----VDEEELKRLEDLHRELE------------------------------  target    NLTTLTIISVLLAVLAVVTGFFGMNVPLPLTDEPHAWLYISLASAGLWIVLSLLLRKIAKKS 6dlm.1    -------------------------------------------------------------- ``` | | | | | | | | | | | | | | | | | | | | | | | | | | | | | | | | | | | | | | | | | | | | | | | | | |
|  | 5kte.1.A | Divalent metal cation transporter MntH  *Crystal structure of Deinococcus radiodurans MntH, an Nramp-family transition metal transporter* | 0.02 |  | 11.76 | 0.11 | 238-271 | X-ray | 3.94 | hetero-1-1-1-mer |  | HHblits | 0.23 |
| ``` target    MVLEKQLGNGCTWIDLDLGKLNKLEDLSEIYGLDKETIEYALDRNERAHMDYHRESETVTFIYNVLDVKKDKAYYETFPM 5kte.1    --------------------------------------------------------------------------------  target    TFIVEHRRLITISNTKNAYVIEQMTRYLENHDTLSIYKFLFASLEIISNAYYPVIEQMDKSRDEVNDLLRQRTTKKNLFV 5kte.1    --------------------------------------------------------------------------------  target    LSDLETGMVYLTAAAKQNRILLEHIQGHALYRSFDEIEREQFDDAMIEAHQLVSMTDLISQILQQLSASYNNILNNNLND 5kte.1    -----------------------------------------------------------------------------PSS  target    NLTTLTIISVLLAVLAVVTGFFGMNVP-LPLTDEPHAWLYISLASAGLWIVLSLLLRKIAKKS 5kte.1    VLILSQVILCFGVPFALVPLLLFTAHHDVMGA------------------------------- ``` | | | | | | | | | | | | | | | | | | | | | | | | | | | | | | | | | | | | | | | | | | | | | | | | | |
|  | 6d9w.1.A | Divalent metal cation transporter MntH  *Crystal structure of Deinococcus radiodurans MntH, an Nramp-family transition metal transporter, in the inward-open apo state* | 0.02 |  | 11.76 | 0.11 | 238-271 | X-ray | 3.94 | hetero-1-1-1-mer | 1 x OS | HHblits | 0.23 |
| ``` target    MVLEKQLGNGCTWIDLDLGKLNKLEDLSEIYGLDKETIEYALDRNERAHMDYHRESETVTFIYNVLDVKKDKAYYETFPM 6d9w.1    --------------------------------------------------------------------------------  target    TFIVEHRRLITISNTKNAYVIEQMTRYLENHDTLSIYKFLFASLEIISNAYYPVIEQMDKSRDEVNDLLRQRTTKKNLFV 6d9w.1    --------------------------------------------------------------------------------  target    LSDLETGMVYLTAAAKQNRILLEHIQGHALYRSFDEIEREQFDDAMIEAHQLVSMTDLISQILQQLSASYNNILNNNLND 6d9w.1    -----------------------------------------------------------------------------PSS  target    NLTTLTIISVLLAVLAVVTGFFGMNVP-LPLTDEPHAWLYISLASAGLWIVLSLLLRKIAKKS 6d9w.1    VLILSQVILCFGVPFALVPLLLFTAHHDVMGA------------------------------- ``` | | | | | | | | | | | | | | | | | | | | | | | | | | | | | | | | | | | | | | | | | | | | | | | | | |
|  | 3jc8.42.A | Type 4 fimbrial assembly protein PilC  *Architectural model of the type IVa pilus machine in a piliated state* | 0.02 |  | 20.00 | 0.10 | 202-231 | EM | 0.00 | monomer |  | HHblits | 0.32 |
| ``` target    MVLEKQLGNGCTWIDLDLGKLNKLEDLSEIYGLDKETIEYALDRNERAHMDYHRESETVTFIYNVLDVKKDKAYYETFPM 3jc8.42   --------------------------------------------------------------------------------  target    TFIVEHRRLITISNTKNAYVIEQMTRYLENHDTLSIYKFLFASLEIISNAYYPVIEQMDKSRDEVNDLLRQRTTKKNLFV 3jc8.42   --------------------------------------------------------------------------------  target    LSDLETGMVYLTAAAKQNRILLEHIQGHALYRSFDEIEREQFDDAMIEAHQLVSMTDLISQILQQLSASYNNILNNNLND 3jc8.42   -----------------------------------------FDELYVQLCAAGEVGGILDAILNRLAAYRE---------  target    NLTTLTIISVLLAVLAVVTGFFGMNVPLPLTDEPHAWLYISLASAGLWIVLSLLLRKIAKKS 3jc8.42   -------------------------------------------------------------- ``` | | | | | | | | | | | | | | | | | | | | | | | | | | | | | | | | | | | | | | | | | | | | | | | | | |
|  | 3jc8.43.A | Type 4 fimbrial assembly protein PilC  *Architectural model of the type IVa pilus machine in a piliated state* | 0.01 |  | 20.00 | 0.10 | 202-231 | EM | 0.00 | monomer |  | HHblits | 0.32 |
| ``` target    MVLEKQLGNGCTWIDLDLGKLNKLEDLSEIYGLDKETIEYALDRNERAHMDYHRESETVTFIYNVLDVKKDKAYYETFPM 3jc8.43   --------------------------------------------------------------------------------  target    TFIVEHRRLITISNTKNAYVIEQMTRYLENHDTLSIYKFLFASLEIISNAYYPVIEQMDKSRDEVNDLLRQRTTKKNLFV 3jc8.43   --------------------------------------------------------------------------------  target    LSDLETGMVYLTAAAKQNRILLEHIQGHALYRSFDEIEREQFDDAMIEAHQLVSMTDLISQILQQLSASYNNILNNNLND 3jc8.43   -----------------------------------------FDELYVQLCAAGEVGGILDAILNRLAAYRE---------  target    NLTTLTIISVLLAVLAVVTGFFGMNVPLPLTDEPHAWLYISLASAGLWIVLSLLLRKIAKKS 3jc8.43   -------------------------------------------------------------- ``` | | | | | | | | | | | | | | | | | | | | | | | | | | | | | | | | | | | | | | | | | | | | | | | | | |
|  | 6c3i.1.A | Divalent metal cation transporter MntH  *Crystal structure of the Deinococcus radiodurans Nramp/MntH divalent transition metal transporter G45R mutant in an inward occluded state* | 0.01 |  | 12.12 | 0.11 | 238-270 | X-ray | 2.95 | monomer | 4 x OLC | HHblits | 0.23 |
| ``` target    MVLEKQLGNGCTWIDLDLGKLNKLEDLSEIYGLDKETIEYALDRNERAHMDYHRESETVTFIYNVLDVKKDKAYYETFPM 6c3i.1    --------------------------------------------------------------------------------  target    TFIVEHRRLITISNTKNAYVIEQMTRYLENHDTLSIYKFLFASLEIISNAYYPVIEQMDKSRDEVNDLLRQRTTKKNLFV 6c3i.1    --------------------------------------------------------------------------------  target    LSDLETGMVYLTAAAKQNRILLEHIQGHALYRSFDEIEREQFDDAMIEAHQLVSMTDLISQILQQLSASYNNILNNNLND 6c3i.1    -----------------------------------------------------------------------------PSS  target    NLTTLTIISVLLAVLAVVTGFFGMNVP-LPLTDEPHAWLYISLASAGLWIVLSLLLRKIAKKS 6c3i.1    VLILSQVILCFGVPFALVPLLLFTARRDVMG-------------------------------- ``` | | | | | | | | | | | | | | | | | | | | | | | | | | | | | | | | | | | | | | | | | | | | | | | | | |
|  | 4n21.1.A | GP2 Ectodomain  *Crystal structure of the GP2 Core Domain from the California Academy of Science Virus* | 0.01 |  | 9.38 | 0.11 | 156-187 | X-ray | 1.99 | homo-trimer |  | HHblits | 0.25 |
| ``` target    MVLEKQLGNGCTWIDLDLGKLNKLEDLSEIYGLDKETIEYALDRNERAHMDYHRESETVTFIYNVLDVKKDKAYYETFPM 4n21.1    --------------------------------------------------------------------------------  target    TFIVEHRRLITISNTKNAYVIEQMTRYLENHDTLSIYKFLFASLEIISNAYYPVIEQMDKSRDEVNDLLRQRTTKKNLFV 4n21.1    ---------------------------------------------------------------------------YTTNA  target    LSDLETGMVYLTAAAKQNRILLEHIQGHALYRSFDEIEREQFDDAMIEAHQLVSMTDLISQILQQLSASYNNILNNNLND 4n21.1    LFLLNKEESEIRDHVVEHELALNYLLA-----------------------------------------------------  target    NLTTLTIISVLLAVLAVVTGFFGMNVPLPLTDEPHAWLYISLASAGLWIVLSLLLRKIAKKS 4n21.1    -------------------------------------------------------------- ``` | | | | | | | | | | | | | | | | | | | | | | | | | | | | | | | | | | | | | | | | | | | | | | | | | |
|  | 4n21.2.B | GP2 Ectodomain  *Crystal structure of the GP2 Core Domain from the California Academy of Science Virus* | 0.01 |  | 9.38 | 0.11 | 156-187 | X-ray | 1.99 | homo-trimer |  | HHblits | 0.25 |
| ``` target    MVLEKQLGNGCTWIDLDLGKLNKLEDLSEIYGLDKETIEYALDRNERAHMDYHRESETVTFIYNVLDVKKDKAYYETFPM 4n21.2    --------------------------------------------------------------------------------  target    TFIVEHRRLITISNTKNAYVIEQMTRYLENHDTLSIYKFLFASLEIISNAYYPVIEQMDKSRDEVNDLLRQRTTKKNLFV 4n21.2    ---------------------------------------------------------------------------YTTNA  target    LSDLETGMVYLTAAAKQNRILLEHIQGHALYRSFDEIEREQFDDAMIEAHQLVSMTDLISQILQQLSASYNNILNNNLND 4n21.2    LFLLNKEESEIRDHVVEHELALNYLLA-----------------------------------------------------  target    NLTTLTIISVLLAVLAVVTGFFGMNVPLPLTDEPHAWLYISLASAGLWIVLSLLLRKIAKKS 4n21.2    -------------------------------------------------------------- ``` | | | | | | | | | | | | | | | | | | | | | | | | | | | | | | | | | | | | | | | | | | | | | | | | | |
|  | 3dl8.1.B | Preprotein translocase subunit secY  *Structure of the complex of aquifex aeolicus SecYEG and bacillus subtilis SecA* | 0.02 |  | 16.67 | 0.10 | 236-265 | X-ray | 7.50 | hetero-oligomer |  | HHblits | 0.29 |
| ``` target    MVLEKQLGNGCTWIDLDLGKLNKLEDLSEIYGLDKETIEYALDRNERAHMDYHRESETVTFIYNVLDVKKDKAYYETFPM 3dl8.1    --------------------------------------------------------------------------------  target    TFIVEHRRLITISNTKNAYVIEQMTRYLENHDTLSIYKFLFASLEIISNAYYPVIEQMDKSRDEVNDLLRQRTTKKNLFV 3dl8.1    --------------------------------------------------------------------------------  target    LSDLETGMVYLTAAAKQNRILLEHIQGHALYRSFDEIEREQFDDAMIEAHQLVSMTDLISQILQQLSASYNNILNNNLND 3dl8.1    ---------------------------------------------------------------------------YKINE  target    NLTTLTIISVLLAVLAVVTGFFGMNVPLPLTDEPHAWLYISLASAGLWIVLSLLLRKIAKKS 3dl8.1    YTKYLTLFVATVQSLGIAFWIRGQV------------------------------------- ``` | | | | | | | | | | | | | | | | | | | | | | | | | | | | | | | | | | | | | | | | | | | | | | | | | |
|  | 5ijn.1.G | NUCLEAR PORE COMPLEX PROTEIN NUP58  *Composite structure of the inner ring of the human nuclear pore complex (32 copies of Nup205)* | 0.03 |  | 13.33 | 0.10 | 122-151 | EM | 0.00 | hetero-6-4-4-4-4-4-… |  | HHblits | 0.28 |
| ``` target    MVLEKQLGNGCTWIDLDLGKLNKLEDLSEIYGLDKETIEYALDRNERAHMDYHRESETVTFIYNVLDVKKDKAYYETFPM 5ijn.1    --------------------------------------------------------------------------------  target    TFIVEHRRLITISNTKNAYVIEQMTRYLENHDTLSIYKFLFASLEIISNAYYPVIEQMDKSRDEVNDLLRQRTTKKNLFV 5ijn.1    -----------------------------------------DYFRILVQQFEVQLQQYRQQIEELENHLAT---------  target    LSDLETGMVYLTAAAKQNRILLEHIQGHALYRSFDEIEREQFDDAMIEAHQLVSMTDLISQILQQLSASYNNILNNNLND 5ijn.1    --------------------------------------------------------------------------------  target    NLTTLTIISVLLAVLAVVTGFFGMNVPLPLTDEPHAWLYISLASAGLWIVLSLLLRKIAKKS 5ijn.1    -------------------------------------------------------------- ``` | | | | | | | | | | | | | | | | | | | | | | | | | | | | | | | | | | | | | | | | | | | | | | | | | |
|  | 6akg.1.A | Claudin-3  *Crystal structure of mouse claudin-3 P134G mutant in complex with C-terminal fragment of Clostridium perfringens enterotoxin* | 0.02 |  | 21.43 | 0.09 | 239-266 | X-ray | 4.30 | hetero-1-1-mer |  | HHblits | 0.33 |
| ``` target    MVLEKQLGNGCTWIDLDLGKLNKLEDLSEIYGLDKETIEYALDRNERAHMDYHRESETVTFIYNVLDVKKDKAYYETFPM 6akg.1    --------------------------------------------------------------------------------  target    TFIVEHRRLITISNTKNAYVIEQMTRYLENHDTLSIYKFLFASLEIISNAYYPVIEQMDKSRDEVNDLLRQRTTKKNLFV 6akg.1    --------------------------------------------------------------------------------  target    LSDLETGMVYLTAAAKQNRILLEHIQGHALYRSFDEIEREQFDDAMIEAHQLVSMTDLISQILQQLSASYNNILNNNLND 6akg.1    ------------------------------------------------------------------------------LQ  target    NLTTLTIISVLLAVLAVVTGFFGMNVPLPLTDEPHAWLYISLASAGLWIVLSLLLRKIAKKS 6akg.1    AARALIVVSILLAAFGLLVALVGAQA------------------------------------ ``` | | | | | | | | | | | | | | | | | | | | | | | | | | | | | | | | | | | | | | | | | | | | | | | | | |
|  | 6tl2.1.A | Divalent metal cation transporter MntH  *Crystal structure of Eremococcus coleocola manganese transporter in complex with an aromatic bis-isothiourea substituted compound* | 0.01 |  | 13.33 | 0.10 | 238-267 | X-ray | 3.80 | monomer | 1 x NJZ | HHblits | 0.28 |
| ``` target    MVLEKQLGNGCTWIDLDLGKLNKLEDLSEIYGLDKETIEYALDRNERAHMDYHRESETVTFIYNVLDVKKDKAYYETFPM 6tl2.1    --------------------------------------------------------------------------------  target    TFIVEHRRLITISNTKNAYVIEQMTRYLENHDTLSIYKFLFASLEIISNAYYPVIEQMDKSRDEVNDLLRQRTTKKNLFV 6tl2.1    --------------------------------------------------------------------------------  target    LSDLETGMVYLTAAAKQNRILLEHIQGHALYRSFDEIEREQFDDAMIEAHQLVSMTDLISQILQQLSASYNNILNNNLND 6tl2.1    -----------------------------------------------------------------------------LNN  target    NLTTLTIISVLLAVLAVVTGFFGMNVPLPLTDEPHAWLYISLASAGLWIVLSLLLRKIAKKS 6tl2.1    LMNNSQVFLALALPFSIVPLLMLTDSA----------------------------------- ``` | | | | | | | | | | | | | | | | | | | | | | | | | | | | | | | | | | | | | | | | | | | | | | | | | |
|  | 7kak.1.A | Protein transport channel Sec61 complex, alpha subunit (Sec61)  *Cryo-EM structure of the Sec complex from T. lanuginosus, wild-type, class without Sec62* | 0.02 |  | 16.67 | 0.10 | 235-264 | EM | 0.00 | hetero-1-1-1-1-1-1-… |  | HHblits | 0.27 |
| ``` target    MVLEKQLGNGCTWIDLDLGKLNKLEDLSEIYGLDKETIEYALDRNERAHMDYHRESETVTFIYNVLDVKKDKAYYETFPM 7kak.1    --------------------------------------------------------------------------------  target    TFIVEHRRLITISNTKNAYVIEQMTRYLENHDTLSIYKFLFASLEIISNAYYPVIEQMDKSRDEVNDLLRQRTTKKNLFV 7kak.1    --------------------------------------------------------------------------------  target    LSDLETGMVYLTAAAKQNRILLEHIQGHALYRSFDEIEREQFDDAMIEAHQLVSMTDLISQILQQLSASYNNILNNNLND 7kak.1    --------------------------------------------------------------------------RELYQT  target    NLTTLTIISVLLAVLAVVT-GFFGMNVPLPLTDEPHAWLYISLASAGLWIVLSLLLRKIAKKS 7kak.1    AQKLFAIILSFGQACVHVLTGLYGQ-------------------------------------- ``` | | | | | | | | | | | | | | | | | | | | | | | | | | | | | | | | | | | | | | | | | | | | | | | | | |
|  | 7kal.1.A | Protein transport channel Sec61 complex, alpha subunit (Sec61)  *Cryo-EM structure of the Sec complex from T. lanuginosus, wild-type, class with Sec62, plug-open conformation* | 0.01 |  | 16.67 | 0.10 | 235-264 | EM | 0.00 | hetero-1-1-1-1-1-1-… |  | HHblits | 0.27 |
| ``` target    MVLEKQLGNGCTWIDLDLGKLNKLEDLSEIYGLDKETIEYALDRNERAHMDYHRESETVTFIYNVLDVKKDKAYYETFPM 7kal.1    --------------------------------------------------------------------------------  target    TFIVEHRRLITISNTKNAYVIEQMTRYLENHDTLSIYKFLFASLEIISNAYYPVIEQMDKSRDEVNDLLRQRTTKKNLFV 7kal.1    --------------------------------------------------------------------------------  target    LSDLETGMVYLTAAAKQNRILLEHIQGHALYRSFDEIEREQFDDAMIEAHQLVSMTDLISQILQQLSASYNNILNNNLND 7kal.1    --------------------------------------------------------------------------RELYQT  target    NLTTLTIISVLLAVLAVVT-GFFGMNVPLPLTDEPHAWLYISLASAGLWIVLSLLLRKIAKKS 7kal.1    AQKLFAIILSFGQACVHVLTGLYGQ-------------------------------------- ``` | | | | | | | | | | | | | | | | | | | | | | | | | | | | | | | | | | | | | | | | | | | | | | | | | |
|  | 7kam.1.A | Protein transport channel Sec61 complex, alpha subunit (Sec61)  *Cryo-EM structure of the Sec complex from T. lanuginosus, wild-type, class with Sec62, plug-closed conformation* | 0.02 |  | 16.67 | 0.10 | 235-264 | EM | 0.00 | hetero-1-1-1-1-1-1-… |  | HHblits | 0.27 |
| ``` target    MVLEKQLGNGCTWIDLDLGKLNKLEDLSEIYGLDKETIEYALDRNERAHMDYHRESETVTFIYNVLDVKKDKAYYETFPM 7kam.1    --------------------------------------------------------------------------------  target    TFIVEHRRLITISNTKNAYVIEQMTRYLENHDTLSIYKFLFASLEIISNAYYPVIEQMDKSRDEVNDLLRQRTTKKNLFV 7kam.1    --------------------------------------------------------------------------------  target    LSDLETGMVYLTAAAKQNRILLEHIQGHALYRSFDEIEREQFDDAMIEAHQLVSMTDLISQILQQLSASYNNILNNNLND 7kam.1    --------------------------------------------------------------------------RELYQT  target    NLTTLTIISVLLAVLAVVT-GFFGMNVPLPLTDEPHAWLYISLASAGLWIVLSLLLRKIAKKS 7kam.1    AQKLFAIILSFGQACVHVLTGLYGQ-------------------------------------- ``` | | | | | | | | | | | | | | | | | | | | | | | | | | | | | | | | | | | | | | | | | | | | | | | | | |
|  | 7jr7.1.B | ATP-binding cassette sub-family G member 8  *Cryo-EM structure of ABCG5/G8 in complex with Fab 2E10 and 11F4* | 0.01 |  | 16.67 | 0.10 | 240-270 | EM | 0.00 | hetero-1-1-1-1-1-1-… |  | HHblits | 0.27 |
| ``` target    MVLEKQLGNGCTWIDLDLGKLNKLEDLSEIYGLDKETIEYALDRNERAHMDYHRESETVTFIYNVLDVKKDKAYYETFPM 7jr7.1    --------------------------------------------------------------------------------  target    TFIVEHRRLITISNTKNAYVIEQMTRYLENHDTLSIYKFLFASLEIISNAYYPVIEQMDKSRDEVNDLLRQRTTKKNLFV 7jr7.1    --------------------------------------------------------------------------------  target    LSDLETGMVYLTAAAKQNRILLEHIQGHALYRSFDEIEREQFDDAMIEAHQLVSMTDLISQILQQLSASYNNILNNNLND 7jr7.1    -------------------------------------------------------------------------------T  target    NLTTLTIISVLLAVLAVVTGFFGMNVP-LPLTDEPHAWLYISLASAGLWIVLSLLLRKIAKKS 7jr7.1    FHMASFFSNALYNSFYLAGGFM-INLSSLWT-------------------------------- ``` | | | | | | | | | | | | | | | | | | | | | | | | | | | | | | | | | | | | | | | | | | | | | | | | | |
|  | 4p79.1.A | Claudin-15  *Crystal structure of mouse claudin-15* | 0.02 |  | 25.93 | 0.09 | 240-266 | X-ray | 2.40 | monomer | 2 x OLC | HHblits | 0.34 |
| ``` target    MVLEKQLGNGCTWIDLDLGKLNKLEDLSEIYGLDKETIEYALDRNERAHMDYHRESETVTFIYNVLDVKKDKAYYETFPM 4p79.1    --------------------------------------------------------------------------------  target    TFIVEHRRLITISNTKNAYVIEQMTRYLENHDTLSIYKFLFASLEIISNAYYPVIEQMDKSRDEVNDLLRQRTTKKNLFV 4p79.1    --------------------------------------------------------------------------------  target    LSDLETGMVYLTAAAKQNRILLEHIQGHALYRSFDEIEREQFDDAMIEAHQLVSMTDLISQILQQLSASYNNILNNNLND 4p79.1    -------------------------------------------------------------------------------Q  target    NLTTLTIISVLLAVLAVVTGFFGMNVPLPLTDEPHAWLYISLASAGLWIVLSLLLRKIAKKS 4p79.1    GCRALMITAILLGFLGLFLGMVGLRA------------------------------------ ``` | | | | | | | | | | | | | | | | | | | | | | | | | | | | | | | | | | | | | | | | | | | | | | | | | |
|  | 7kp4.1.A | Claudin-4  *Crystal structure of human claudin-4 in complex with Clostridium perfringens enterotoxin C-terminal domain* | 0.02 |  | 29.63 | 0.09 | 240-266 | X-ray | 3.37 | hetero-1-1-mer |  | HHblits | 0.34 |
| ``` target    MVLEKQLGNGCTWIDLDLGKLNKLEDLSEIYGLDKETIEYALDRNERAHMDYHRESETVTFIYNVLDVKKDKAYYETFPM 7kp4.1    --------------------------------------------------------------------------------  target    TFIVEHRRLITISNTKNAYVIEQMTRYLENHDTLSIYKFLFASLEIISNAYYPVIEQMDKSRDEVNDLLRQRTTKKNLFV 7kp4.1    --------------------------------------------------------------------------------  target    LSDLETGMVYLTAAAKQNRILLEHIQGHALYRSFDEIEREQFDDAMIEAHQLVSMTDLISQILQQLSASYNNILNNNLND 7kp4.1    -------------------------------------------------------------------------------Q  target    NLTTLTIISVLLAVLAVVTGFFGMNVPLPLTDEPHAWLYISLASAGLWIVLSLLLRKIAKKS 7kp4.1    AARALVIISIIVAALGVLLSVVGGKC------------------------------------ ``` | | | | | | | | | | | | | | | | | | | | | | | | | | | | | | | | | | | | | | | | | | | | | | | | | |
|  | 5b2g.1.A | Endolysin,Claudin-4  *Crystal structure of human claudin-4 in complex with C-terminal fragment of Clostridium perfringens enterotoxin* | 0.01 |  | 29.63 | 0.09 | 240-266 | X-ray | 3.50 | hetero-1-1-mer |  | HHblits | 0.34 |
| ``` target    MVLEKQLGNGCTWIDLDLGKLNKLEDLSEIYGLDKETIEYALDRNERAHMDYHRESETVTFIYNVLDVKKDKAYYETFPM 5b2g.1    --------------------------------------------------------------------------------  target    TFIVEHRRLITISNTKNAYVIEQMTRYLENHDTLSIYKFLFASLEIISNAYYPVIEQMDKSRDEVNDLLRQRTTKKNLFV 5b2g.1    --------------------------------------------------------------------------------  target    LSDLETGMVYLTAAAKQNRILLEHIQGHALYRSFDEIEREQFDDAMIEAHQLVSMTDLISQILQQLSASYNNILNNNLND 5b2g.1    -------------------------------------------------------------------------------Q  target    NLTTLTIISVLLAVLAVVTGFFGMNVPLPLTDEPHAWLYISLASAGLWIVLSLLLRKIAKKS 5b2g.1    AARALVIISIIVAALGVLLSVVGGKC------------------------------------ ``` | | | | | | | | | | | | | | | | | | | | | | | | | | | | | | | | | | | | | | | | | | | | | | | | | |
|  | 5b2g.2.A | Endolysin,Claudin-4  *Crystal structure of human claudin-4 in complex with C-terminal fragment of Clostridium perfringens enterotoxin* | 0.02 |  | 29.63 | 0.09 | 240-266 | X-ray | 3.50 | hetero-1-1-mer |  | HHblits | 0.34 |
| ``` target    MVLEKQLGNGCTWIDLDLGKLNKLEDLSEIYGLDKETIEYALDRNERAHMDYHRESETVTFIYNVLDVKKDKAYYETFPM 5b2g.2    --------------------------------------------------------------------------------  target    TFIVEHRRLITISNTKNAYVIEQMTRYLENHDTLSIYKFLFASLEIISNAYYPVIEQMDKSRDEVNDLLRQRTTKKNLFV 5b2g.2    --------------------------------------------------------------------------------  target    LSDLETGMVYLTAAAKQNRILLEHIQGHALYRSFDEIEREQFDDAMIEAHQLVSMTDLISQILQQLSASYNNILNNNLND 5b2g.2    -------------------------------------------------------------------------------Q  target    NLTTLTIISVLLAVLAVVTGFFGMNVPLPLTDEPHAWLYISLASAGLWIVLSLLLRKIAKKS 5b2g.2    AARALVIISIIVAALGVLLSVVGGKC------------------------------------ ``` | | | | | | | | | | | | | | | | | | | | | | | | | | | | | | | | | | | | | | | | | | | | | | | | | |
|  | 5b2g.3.A | Endolysin,Claudin-4  *Crystal structure of human claudin-4 in complex with C-terminal fragment of Clostridium perfringens enterotoxin* | 0.01 |  | 29.63 | 0.09 | 240-266 | X-ray | 3.50 | hetero-1-1-mer |  | HHblits | 0.34 |
| ``` target    MVLEKQLGNGCTWIDLDLGKLNKLEDLSEIYGLDKETIEYALDRNERAHMDYHRESETVTFIYNVLDVKKDKAYYETFPM 5b2g.3    --------------------------------------------------------------------------------  target    TFIVEHRRLITISNTKNAYVIEQMTRYLENHDTLSIYKFLFASLEIISNAYYPVIEQMDKSRDEVNDLLRQRTTKKNLFV 5b2g.3    --------------------------------------------------------------------------------  target    LSDLETGMVYLTAAAKQNRILLEHIQGHALYRSFDEIEREQFDDAMIEAHQLVSMTDLISQILQQLSASYNNILNNNLND 5b2g.3    -------------------------------------------------------------------------------Q  target    NLTTLTIISVLLAVLAVVTGFFGMNVPLPLTDEPHAWLYISLASAGLWIVLSLLLRKIAKKS 5b2g.3    AARALVIISIIVAALGVLLSVVGGKC------------------------------------ ``` | | | | | | | | | | | | | | | | | | | | | | | | | | | | | | | | | | | | | | | | | | | | | | | | | |
|  | 5b2g.4.A | Endolysin,Claudin-4  *Crystal structure of human claudin-4 in complex with C-terminal fragment of Clostridium perfringens enterotoxin* | 0.01 |  | 29.63 | 0.09 | 240-266 | X-ray | 3.50 | hetero-1-1-mer |  | HHblits | 0.34 |
| ``` target    MVLEKQLGNGCTWIDLDLGKLNKLEDLSEIYGLDKETIEYALDRNERAHMDYHRESETVTFIYNVLDVKKDKAYYETFPM 5b2g.4    --------------------------------------------------------------------------------  target    TFIVEHRRLITISNTKNAYVIEQMTRYLENHDTLSIYKFLFASLEIISNAYYPVIEQMDKSRDEVNDLLRQRTTKKNLFV 5b2g.4    --------------------------------------------------------------------------------  target    LSDLETGMVYLTAAAKQNRILLEHIQGHALYRSFDEIEREQFDDAMIEAHQLVSMTDLISQILQQLSASYNNILNNNLND 5b2g.4    -------------------------------------------------------------------------------Q  target    NLTTLTIISVLLAVLAVVTGFFGMNVPLPLTDEPHAWLYISLASAGLWIVLSLLLRKIAKKS 5b2g.4    AARALVIISIIVAALGVLLSVVGGKC------------------------------------ ``` | | | | | | | | | | | | | | | | | | | | | | | | | | | | | | | | | | | | | | | | | | | | | | | | | |
|  | 6ake.1.A | Claudin-3  *Crystal structure of mouse claudin-3 in complex with C-terminal fragment of Clostridium perfringens enterotoxin* | 0.01 |  | 22.22 | 0.09 | 240-266 | X-ray | 3.60 | hetero-1-1-mer |  | HHblits | 0.34 |
| ``` target    MVLEKQLGNGCTWIDLDLGKLNKLEDLSEIYGLDKETIEYALDRNERAHMDYHRESETVTFIYNVLDVKKDKAYYETFPM 6ake.1    --------------------------------------------------------------------------------  target    TFIVEHRRLITISNTKNAYVIEQMTRYLENHDTLSIYKFLFASLEIISNAYYPVIEQMDKSRDEVNDLLRQRTTKKNLFV 6ake.1    --------------------------------------------------------------------------------  target    LSDLETGMVYLTAAAKQNRILLEHIQGHALYRSFDEIEREQFDDAMIEAHQLVSMTDLISQILQQLSASYNNILNNNLND 6ake.1    -------------------------------------------------------------------------------Q  target    NLTTLTIISVLLAVLAVVTGFFGMNVPLPLTDEPHAWLYISLASAGLWIVLSLLLRKIAKKS 6ake.1    AARALIVVSILLAAFGLLVALVGAQA------------------------------------ ``` | | | | | | | | | | | | | | | | | | | | | | | | | | | | | | | | | | | | | | | | | | | | | | | | | |
|  | 6ake.2.A | Claudin-3  *Crystal structure of mouse claudin-3 in complex with C-terminal fragment of Clostridium perfringens enterotoxin* | 0.01 |  | 22.22 | 0.09 | 240-266 | X-ray | 3.60 | hetero-1-1-mer |  | HHblits | 0.34 |
| ``` target    MVLEKQLGNGCTWIDLDLGKLNKLEDLSEIYGLDKETIEYALDRNERAHMDYHRESETVTFIYNVLDVKKDKAYYETFPM 6ake.2    --------------------------------------------------------------------------------  target    TFIVEHRRLITISNTKNAYVIEQMTRYLENHDTLSIYKFLFASLEIISNAYYPVIEQMDKSRDEVNDLLRQRTTKKNLFV 6ake.2    --------------------------------------------------------------------------------  target    LSDLETGMVYLTAAAKQNRILLEHIQGHALYRSFDEIEREQFDDAMIEAHQLVSMTDLISQILQQLSASYNNILNNNLND 6ake.2    -------------------------------------------------------------------------------Q  target    NLTTLTIISVLLAVLAVVTGFFGMNVPLPLTDEPHAWLYISLASAGLWIVLSLLLRKIAKKS 6ake.2    AARALIVVSILLAAFGLLVALVGAQA------------------------------------ ``` | | | | | | | | | | | | | | | | | | | | | | | | | | | | | | | | | | | | | | | | | | | | | | | | | |
|  | 1qle.1.A | CYTOCHROME C OXIDASE POLYPEPTIDE I-BETA  *CRYO-STRUCTURE OF THE PARACOCCUS DENITRIFICANS FOUR-SUBUNIT CYTOCHROME C OXIDASE IN THE COMPLETELY OXIDIZED STATE COMPLEXED WITH AN ANTIBODY FV FRAGMENT* | 0.00 |  | 17.24 | 0.10 | 233-261 | X-ray | 3.00 | hetero-oligomer | 2 x HEA, 1 x CU, 1 x CA, 1 x CUA, 1 x MN, 2 x PC1 | HHblits | 0.28 |
| ``` target    MVLEKQLGNGCTWIDLDLGKLNKLEDLSEIYGLDKETIEYALDRNERAHMDYHRESETVTFIYNVLDVKKDKAYYETFPM 1qle.1    --------------------------------------------------------------------------------  target    TFIVEHRRLITISNTKNAYVIEQMTRYLENHDTLSIYKFLFASLEIISNAYYPVIEQMDKSRDEVNDLLRQRTTKKNLFV 1qle.1    --------------------------------------------------------------------------------  target    LSDLETGMVYLTAAAKQNRILLEHIQGHALYRSFDEIEREQFDDAMIEAHQLVSMTDLISQILQQLSASYNNILNNNLND 1qle.1    ------------------------------------------------------------------------MAFPRLNN  target    NLTTLTIISVLLAVLAVVTGFFGMNVPLPLTDEPHAWLYISLASAGLWIVLSLLLRKIAKKS 1qle.1    LSYWMYVCGVALGVASLLAPG----------------------------------------- ``` | | | | | | | | | | | | | | | | | | | | | | | | | | | | | | | | | | | | | | | | | | | | | | | | | |
|  | 3x29.1.A | Claudin-19  *CRYSTAL STRUCTURE of MOUSE CLAUDIN-19 IN COMPLEX with C-TERMINAL FRAGMENT OF CLOSTRIDIUM PERFRINGENS ENTEROTOXIN* | 0.01 |  | 30.77 | 0.09 | 241-266 | X-ray | 3.70 | hetero-oligomer |  | HHblits | 0.36 |
| ``` target    MVLEKQLGNGCTWIDLDLGKLNKLEDLSEIYGLDKETIEYALDRNERAHMDYHRESETVTFIYNVLDVKKDKAYYETFPM 3x29.1    --------------------------------------------------------------------------------  target    TFIVEHRRLITISNTKNAYVIEQMTRYLENHDTLSIYKFLFASLEIISNAYYPVIEQMDKSRDEVNDLLRQRTTKKNLFV 3x29.1    --------------------------------------------------------------------------------  target    LSDLETGMVYLTAAAKQNRILLEHIQGHALYRSFDEIEREQFDDAMIEAHQLVSMTDLISQILQQLSASYNNILNNNLND 3x29.1    --------------------------------------------------------------------------------  target    NLTTLTIISVLLAVLAVVTGFFGMNVPLPLTDEPHAWLYISLASAGLWIVLSLLLRKIAKKS 3x29.1    SARALMVVAVLLGFVAMVLSVVGMKA------------------------------------ ``` | | | | | | | | | | | | | | | | | | | | | | | | | | | | | | | | | | | | | | | | | | | | | | | | | |
|  | 3omi.1.A | Cytochrome c oxidase, aa3 type, subunit I  *Catalytic core subunits (I and II) of cytochrome C oxidase from Rhodobacter sphaeroides with D132A mutation* | 0.00 |  | 17.24 | 0.10 | 233-261 | X-ray | 2.15 | hetero-oligomer | 1 x OH, 5 x DMU, 6 x TRD, 2 x HEA, 2 x CU, 1 x MG, 1 x CA, 1 x HTH, 1 x CU1, 2 x CD | HHblits | 0.28 |
| ``` target    MVLEKQLGNGCTWIDLDLGKLNKLEDLSEIYGLDKETIEYALDRNERAHMDYHRESETVTFIYNVLDVKKDKAYYETFPM 3omi.1    --------------------------------------------------------------------------------  target    TFIVEHRRLITISNTKNAYVIEQMTRYLENHDTLSIYKFLFASLEIISNAYYPVIEQMDKSRDEVNDLLRQRTTKKNLFV 3omi.1    --------------------------------------------------------------------------------  target    LSDLETGMVYLTAAAKQNRILLEHIQGHALYRSFDEIEREQFDDAMIEAHQLVSMTDLISQILQQLSASYNNILNNNLND 3omi.1    ------------------------------------------------------------------------MAFPRMNN  target    NLTTLTIISVLLAVLAVVTGFFGMNVPLPLTDEPHAWLYISLASAGLWIVLSLLLRKIAKKS 3omi.1    LSYWLYVAGTSLAVASLFAPG----------------------------------------- ``` | | | | | | | | | | | | | | | | | | | | | | | | | | | | | | | | | | | | | | | | | | | | | | | | | |
|  | 6pw0.1.A | Cytochrome c oxidase subunit 1  *Cytochrome C oxidase delta 6 mutant* | 0.00 |  | 17.24 | 0.10 | 233-261 | X-ray | 2.50 | hetero-1-1-mer | 1 x OH, 2 x HEA, 6 x DMU, 10 x TRD, 2 x HTH, 3 x CU, 1 x MG, 1 x CA, 2 x CD, 1 x GLC-GLC | HHblits | 0.28 |
| ``` target    MVLEKQLGNGCTWIDLDLGKLNKLEDLSEIYGLDKETIEYALDRNERAHMDYHRESETVTFIYNVLDVKKDKAYYETFPM 6pw0.1    --------------------------------------------------------------------------------  target    TFIVEHRRLITISNTKNAYVIEQMTRYLENHDTLSIYKFLFASLEIISNAYYPVIEQMDKSRDEVNDLLRQRTTKKNLFV 6pw0.1    --------------------------------------------------------------------------------  target    LSDLETGMVYLTAAAKQNRILLEHIQGHALYRSFDEIEREQFDDAMIEAHQLVSMTDLISQILQQLSASYNNILNNNLND 6pw0.1    ------------------------------------------------------------------------MAFPRMNN  target    NLTTLTIISVLLAVLAVVTGFFGMNVPLPLTDEPHAWLYISLASAGLWIVLSLLLRKIAKKS 6pw0.1    LSYWLYVAGTSLAVASLFAPG----------------------------------------- ``` | | | | | | | | | | | | | | | | | | | | | | | | | | | | | | | | | | | | | | | | | | | | | | | | | |
|  | 6ci0.1.A | Cytochrome c oxidase subunit 1  *Catalytic core subunits (I and II) of cytochrome C oxidase from Rhodobacter sphaeroides with E101A (II) mutation* | 0.00 |  | 17.24 | 0.10 | 233-261 | X-ray | 2.40 | hetero-1-1-mer | 5 x DMU, 11 x TRD, 3 x CU, 1 x MG, 1 x CA, 2 x HEA, 1 x CD, 3 x HTH, 1 x GLC-GLC | HHblits | 0.28 |
| ``` target    MVLEKQLGNGCTWIDLDLGKLNKLEDLSEIYGLDKETIEYALDRNERAHMDYHRESETVTFIYNVLDVKKDKAYYETFPM 6ci0.1    --------------------------------------------------------------------------------  target    TFIVEHRRLITISNTKNAYVIEQMTRYLENHDTLSIYKFLFASLEIISNAYYPVIEQMDKSRDEVNDLLRQRTTKKNLFV 6ci0.1    --------------------------------------------------------------------------------  target    LSDLETGMVYLTAAAKQNRILLEHIQGHALYRSFDEIEREQFDDAMIEAHQLVSMTDLISQILQQLSASYNNILNNNLND 6ci0.1    ------------------------------------------------------------------------MAFPRMNN  target    NLTTLTIISVLLAVLAVVTGFFGMNVPLPLTDEPHAWLYISLASAGLWIVLSLLLRKIAKKS 6ci0.1    LSYWLYVAGTSLAVASLFAPG----------------------------------------- ``` | | | | | | | | | | | | | | | | | | | | | | | | | | | | | | | | | | | | | | | | | | | | | | | | | |
|  | 3om3.1.A | Cytochrome c oxidase, aa3 type, subunit I  *Catalytic core subunits (I and II) of cytochrome C oxidase from Rhodobacter sphaeroides with K362M mutation in the reduced state* | 0.00 |  | 17.24 | 0.10 | 233-261 | X-ray | 2.60 | hetero-oligomer | 5 x DMU, 5 x TRD, 2 x HEA, 3 x CU1, 1 x MG, 1 x CA, 1 x HTH, 2 x CD | HHblits | 0.28 |
| ``` target    MVLEKQLGNGCTWIDLDLGKLNKLEDLSEIYGLDKETIEYALDRNERAHMDYHRESETVTFIYNVLDVKKDKAYYETFPM 3om3.1    --------------------------------------------------------------------------------  target    TFIVEHRRLITISNTKNAYVIEQMTRYLENHDTLSIYKFLFASLEIISNAYYPVIEQMDKSRDEVNDLLRQRTTKKNLFV 3om3.1    --------------------------------------------------------------------------------  target    LSDLETGMVYLTAAAKQNRILLEHIQGHALYRSFDEIEREQFDDAMIEAHQLVSMTDLISQILQQLSASYNNILNNNLND 3om3.1    ------------------------------------------------------------------------MAFPRMNN  target    NLTTLTIISVLLAVLAVVTGFFGMNVPLPLTDEPHAWLYISLASAGLWIVLSLLLRKIAKKS 3om3.1    LSYWLYVAGTSLAVASLFAPG----------------------------------------- ``` | | | | | | | | | | | | | | | | | | | | | | | | | | | | | | | | | | | | | | | | | | | | | | | | | |
|  | 6pw1.1.A | Cytochrome c oxidase subunit 1  *Cytochrome c Oxidase delta 16* | 0.00 |  | 17.24 | 0.10 | 233-261 | X-ray | 2.10 | hetero-1-1-mer | 11 x TRD, 5 x DMU, 2 x HEA, 3 x CU, 1 x MG, 1 x CA, 2 x HTH, 2 x CD, 1 x GLC-GLC | HHblits | 0.28 |
| ``` target    MVLEKQLGNGCTWIDLDLGKLNKLEDLSEIYGLDKETIEYALDRNERAHMDYHRESETVTFIYNVLDVKKDKAYYETFPM 6pw1.1    --------------------------------------------------------------------------------  target    TFIVEHRRLITISNTKNAYVIEQMTRYLENHDTLSIYKFLFASLEIISNAYYPVIEQMDKSRDEVNDLLRQRTTKKNLFV 6pw1.1    --------------------------------------------------------------------------------  target    LSDLETGMVYLTAAAKQNRILLEHIQGHALYRSFDEIEREQFDDAMIEAHQLVSMTDLISQILQQLSASYNNILNNNLND 6pw1.1    ------------------------------------------------------------------------MAFPRMNN  target    NLTTLTIISVLLAVLAVVTGFFGMNVPLPLTDEPHAWLYISLASAGLWIVLSLLLRKIAKKS 6pw1.1    LSYWLYVAGTSLAVASLFAPG----------------------------------------- ``` | | | | | | | | | | | | | | | | | | | | | | | | | | | | | | | | | | | | | | | | | | | | | | | | | |
|  | 5ch4.1.A | Protein translocase subunit SecY  *Peptide-Bound State of Thermus thermophilus SecYEG* | 0.02 |  | 17.24 | 0.10 | 236-264 | X-ray | 3.64 | hetero-1-1-1-mer |  | HHblits | 0.27 |
| ``` target    MVLEKQLGNGCTWIDLDLGKLNKLEDLSEIYGLDKETIEYALDRNERAHMDYHRESETVTFIYNVLDVKKDKAYYETFPM 5ch4.1    --------------------------------------------------------------------------------  target    TFIVEHRRLITISNTKNAYVIEQMTRYLENHDTLSIYKFLFASLEIISNAYYPVIEQMDKSRDEVNDLLRQRTTKKNLFV 5ch4.1    --------------------------------------------------------------------------------  target    LSDLETGMVYLTAAAKQNRILLEHIQGHALYRSFDEIEREQFDDAMIEAHQLVSMTDLISQILQQLSASYNNILNNNLND 5ch4.1    ---------------------------------------------------------------------------RIINQ  target    NLTTLTIISVLLAVLAVVTGFFGMNVPLPLTDEPHAWLYISLASAGLWIVLSLLLRKIAKKS 5ch4.1    YTRIGGIALGAFQGFFLATAFLGA-------------------------------------- ``` | | | | | | | | | | | | | | | | | | | | | | | | | | | | | | | | | | | | | | | | | | | | | | | | | |
|  | 5aww.1.A | Protein translocase subunit SecY  *Precise Resting State of Thermus thermophilus SecYEG* | 0.02 |  | 17.24 | 0.10 | 236-264 | X-ray | 2.72 | hetero-1-1-1-mer | 9 x OLC | HHblits | 0.27 |
| ``` target    MVLEKQLGNGCTWIDLDLGKLNKLEDLSEIYGLDKETIEYALDRNERAHMDYHRESETVTFIYNVLDVKKDKAYYETFPM 5aww.1    --------------------------------------------------------------------------------  target    TFIVEHRRLITISNTKNAYVIEQMTRYLENHDTLSIYKFLFASLEIISNAYYPVIEQMDKSRDEVNDLLRQRTTKKNLFV 5aww.1    --------------------------------------------------------------------------------  target    LSDLETGMVYLTAAAKQNRILLEHIQGHALYRSFDEIEREQFDDAMIEAHQLVSMTDLISQILQQLSASYNNILNNNLND 5aww.1    ---------------------------------------------------------------------------RIINQ  target    NLTTLTIISVLLAVLAVVTGFFGMNVPLPLTDEPHAWLYISLASAGLWIVLSLLLRKIAKKS 5aww.1    YTRIGGIALGAFQGFFLATAFLGA-------------------------------------- ``` | | | | | | | | | | | | | | | | | | | | | | | | | | | | | | | | | | | | | | | | | | | | | | | | | |
|  | 2zjs.1.A | Preprotein translocase SecY subunit  *Crystal Structure of SecYE translocon from Thermus thermophilus with a Fab fragment* | 0.02 |  | 17.24 | 0.10 | 236-264 | X-ray | 3.20 | hetero-oligomer | 1 x ZN | HHblits | 0.27 |
| ``` target    MVLEKQLGNGCTWIDLDLGKLNKLEDLSEIYGLDKETIEYALDRNERAHMDYHRESETVTFIYNVLDVKKDKAYYETFPM 2zjs.1    --------------------------------------------------------------------------------  target    TFIVEHRRLITISNTKNAYVIEQMTRYLENHDTLSIYKFLFASLEIISNAYYPVIEQMDKSRDEVNDLLRQRTTKKNLFV 2zjs.1    --------------------------------------------------------------------------------  target    LSDLETGMVYLTAAAKQNRILLEHIQGHALYRSFDEIEREQFDDAMIEAHQLVSMTDLISQILQQLSASYNNILNNNLND 2zjs.1    ---------------------------------------------------------------------------RIINQ  target    NLTTLTIISVLLAVLAVVTGFFGMNVPLPLTDEPHAWLYISLASAGLWIVLSLLLRKIAKKS 2zjs.1    YTRIGGIALGAFQGFFLATAFLGA-------------------------------------- ``` | | | | | | | | | | | | | | | | | | | | | | | | | | | | | | | | | | | | | | | | | | | | | | | | | |
|  | 2zqp.1.A | Preprotein translocase SecY subunit  *Crystal Structure of SecYE translocon from Thermus thermophilus* | 0.01 |  | 17.24 | 0.10 | 236-264 | X-ray | 6.00 | hetero-oligomer |  | HHblits | 0.27 |
| ``` target    MVLEKQLGNGCTWIDLDLGKLNKLEDLSEIYGLDKETIEYALDRNERAHMDYHRESETVTFIYNVLDVKKDKAYYETFPM 2zqp.1    --------------------------------------------------------------------------------  target    TFIVEHRRLITISNTKNAYVIEQMTRYLENHDTLSIYKFLFASLEIISNAYYPVIEQMDKSRDEVNDLLRQRTTKKNLFV 2zqp.1    --------------------------------------------------------------------------------  target    LSDLETGMVYLTAAAKQNRILLEHIQGHALYRSFDEIEREQFDDAMIEAHQLVSMTDLISQILQQLSASYNNILNNNLND 2zqp.1    ---------------------------------------------------------------------------RIINQ  target    NLTTLTIISVLLAVLAVVTGFFGMNVPLPLTDEPHAWLYISLASAGLWIVLSLLLRKIAKKS 2zqp.1    YTRIGGIALGAFQGFFLATAFLGA-------------------------------------- ``` | | | | | | | | | | | | | | | | | | | | | | | | | | | | | | | | | | | | | | | | | | | | | | | | | |
|  | 2osz.1.C | Nucleoporin p58/p45  *Structure of Nup58/45 suggests flexible nuclear pore diameter by intermolecular sliding* | 0.02 |  | 10.71 | 0.09 | 124-151 | X-ray | 2.85 | homo-tetramer |  | HHblits | 0.29 |
| ``` target    MVLEKQLGNGCTWIDLDLGKLNKLEDLSEIYGLDKETIEYALDRNERAHMDYHRESETVTFIYNVLDVKKDKAYYETFPM 2osz.1    --------------------------------------------------------------------------------  target    TFIVEHRRLITISNTKNAYVIEQMTRYLENHDTLSIYKFLFASLEIISNAYYPVIEQMDKSRDEVNDLLRQRTTKKNLFV 2osz.1    -------------------------------------------FRVLVQQFEVQLQQYRQQIEELENHLAT---------  target    LSDLETGMVYLTAAAKQNRILLEHIQGHALYRSFDEIEREQFDDAMIEAHQLVSMTDLISQILQQLSASYNNILNNNLND 2osz.1    --------------------------------------------------------------------------------  target    NLTTLTIISVLLAVLAVVTGFFGMNVPLPLTDEPHAWLYISLASAGLWIVLSLLLRKIAKKS 2osz.1    -------------------------------------------------------------- ``` | | | | | | | | | | | | | | | | | | | | | | | | | | | | | | | | | | | | | | | | | | | | | | | | | |
|  | 3t98.1.B | Nucleoporin Nup58/Nup45  *Molecular Architecture of the Transport Channel of the Nuclear Pore Complex: Nup54/Nup58* | 0.02 |  | 10.71 | 0.09 | 124-151 | X-ray | 2.50 | hetero-oligomer |  | HHblits | 0.29 |
| ``` target    MVLEKQLGNGCTWIDLDLGKLNKLEDLSEIYGLDKETIEYALDRNERAHMDYHRESETVTFIYNVLDVKKDKAYYETFPM 3t98.1    --------------------------------------------------------------------------------  target    TFIVEHRRLITISNTKNAYVIEQMTRYLENHDTLSIYKFLFASLEIISNAYYPVIEQMDKSRDEVNDLLRQRTTKKNLFV 3t98.1    -------------------------------------------FRVLVQQFEVQLQQYRQQIEELENHLAT---------  target    LSDLETGMVYLTAAAKQNRILLEHIQGHALYRSFDEIEREQFDDAMIEAHQLVSMTDLISQILQQLSASYNNILNNNLND 3t98.1    --------------------------------------------------------------------------------  target    NLTTLTIISVLLAVLAVVTGFFGMNVPLPLTDEPHAWLYISLASAGLWIVLSLLLRKIAKKS 3t98.1    -------------------------------------------------------------- ``` | | | | | | | | | | | | | | | | | | | | | | | | | | | | | | | | | | | | | | | | | | | | | | | | | |
|  | 2osz.1.D | Nucleoporin p58/p45  *Structure of Nup58/45 suggests flexible nuclear pore diameter by intermolecular sliding* | 0.02 |  | 10.71 | 0.09 | 124-151 | X-ray | 2.85 | homo-tetramer |  | HHblits | 0.29 |
| ``` target    MVLEKQLGNGCTWIDLDLGKLNKLEDLSEIYGLDKETIEYALDRNERAHMDYHRESETVTFIYNVLDVKKDKAYYETFPM 2osz.1    --------------------------------------------------------------------------------  target    TFIVEHRRLITISNTKNAYVIEQMTRYLENHDTLSIYKFLFASLEIISNAYYPVIEQMDKSRDEVNDLLRQRTTKKNLFV 2osz.1    -------------------------------------------FRVLVQQFEVQLQQYRQQIEELENHLAT---------  target    LSDLETGMVYLTAAAKQNRILLEHIQGHALYRSFDEIEREQFDDAMIEAHQLVSMTDLISQILQQLSASYNNILNNNLND 2osz.1    --------------------------------------------------------------------------------  target    NLTTLTIISVLLAVLAVVTGFFGMNVPLPLTDEPHAWLYISLASAGLWIVLSLLLRKIAKKS 2osz.1    -------------------------------------------------------------- ``` | | | | | | | | | | | | | | | | | | | | | | | | | | | | | | | | | | | | | | | | | | | | | | | | | |
|  | 6akf.1.A | Claudin-3  *Crystal structure of mouse claudin-3 P134A mutant in complex with C-terminal fragment of Clostridium perfringens enterotoxin* | 0.01 |  | 23.08 | 0.09 | 241-266 | X-ray | 3.90 | hetero-1-1-mer |  | HHblits | 0.34 |
| ``` target    MVLEKQLGNGCTWIDLDLGKLNKLEDLSEIYGLDKETIEYALDRNERAHMDYHRESETVTFIYNVLDVKKDKAYYETFPM 6akf.1    --------------------------------------------------------------------------------  target    TFIVEHRRLITISNTKNAYVIEQMTRYLENHDTLSIYKFLFASLEIISNAYYPVIEQMDKSRDEVNDLLRQRTTKKNLFV 6akf.1    --------------------------------------------------------------------------------  target    LSDLETGMVYLTAAAKQNRILLEHIQGHALYRSFDEIEREQFDDAMIEAHQLVSMTDLISQILQQLSASYNNILNNNLND 6akf.1    --------------------------------------------------------------------------------  target    NLTTLTIISVLLAVLAVVTGFFGMNVPLPLTDEPHAWLYISLASAGLWIVLSLLLRKIAKKS 6akf.1    AARALIVVSILLAAFGLLVALVGAQA------------------------------------ ``` | | | | | | | | | | | | | | | | | | | | | | | | | | | | | | | | | | | | | | | | | | | | | | | | | |
|  | 6akf.2.A | Claudin-3  *Crystal structure of mouse claudin-3 P134A mutant in complex with C-terminal fragment of Clostridium perfringens enterotoxin* | 0.01 |  | 23.08 | 0.09 | 241-266 | X-ray | 3.90 | hetero-1-1-mer |  | HHblits | 0.34 |
| ``` target    MVLEKQLGNGCTWIDLDLGKLNKLEDLSEIYGLDKETIEYALDRNERAHMDYHRESETVTFIYNVLDVKKDKAYYETFPM 6akf.2    --------------------------------------------------------------------------------  target    TFIVEHRRLITISNTKNAYVIEQMTRYLENHDTLSIYKFLFASLEIISNAYYPVIEQMDKSRDEVNDLLRQRTTKKNLFV 6akf.2    --------------------------------------------------------------------------------  target    LSDLETGMVYLTAAAKQNRILLEHIQGHALYRSFDEIEREQFDDAMIEAHQLVSMTDLISQILQQLSASYNNILNNNLND 6akf.2    --------------------------------------------------------------------------------  target    NLTTLTIISVLLAVLAVVTGFFGMNVPLPLTDEPHAWLYISLASAGLWIVLSLLLRKIAKKS 6akf.2    AARALIVVSILLAAFGLLVALVGAQA------------------------------------ ``` | | | | | | | | | | | | | | | | | | | | | | | | | | | | | | | | | | | | | | | | | | | | | | | | | |
|  | 6akf.3.A | Claudin-3  *Crystal structure of mouse claudin-3 P134A mutant in complex with C-terminal fragment of Clostridium perfringens enterotoxin* | 0.01 |  | 23.08 | 0.09 | 241-266 | X-ray | 3.90 | hetero-1-1-mer |  | HHblits | 0.34 |
| ``` target    MVLEKQLGNGCTWIDLDLGKLNKLEDLSEIYGLDKETIEYALDRNERAHMDYHRESETVTFIYNVLDVKKDKAYYETFPM 6akf.3    --------------------------------------------------------------------------------  target    TFIVEHRRLITISNTKNAYVIEQMTRYLENHDTLSIYKFLFASLEIISNAYYPVIEQMDKSRDEVNDLLRQRTTKKNLFV 6akf.3    --------------------------------------------------------------------------------  target    LSDLETGMVYLTAAAKQNRILLEHIQGHALYRSFDEIEREQFDDAMIEAHQLVSMTDLISQILQQLSASYNNILNNNLND 6akf.3    --------------------------------------------------------------------------------  target    NLTTLTIISVLLAVLAVVTGFFGMNVPLPLTDEPHAWLYISLASAGLWIVLSLLLRKIAKKS 6akf.3    AARALIVVSILLAAFGLLVALVGAQA------------------------------------ ``` | | | | | | | | | | | | | | | | | | | | | | | | | | | | | | | | | | | | | | | | | | | | | | | | | |
|  | 6akf.4.A | Claudin-3  *Crystal structure of mouse claudin-3 P134A mutant in complex with C-terminal fragment of Clostridium perfringens enterotoxin* | 0.01 |  | 23.08 | 0.09 | 241-266 | X-ray | 3.90 | hetero-1-1-mer |  | HHblits | 0.34 |
| ``` target    MVLEKQLGNGCTWIDLDLGKLNKLEDLSEIYGLDKETIEYALDRNERAHMDYHRESETVTFIYNVLDVKKDKAYYETFPM 6akf.4    --------------------------------------------------------------------------------  target    TFIVEHRRLITISNTKNAYVIEQMTRYLENHDTLSIYKFLFASLEIISNAYYPVIEQMDKSRDEVNDLLRQRTTKKNLFV 6akf.4    --------------------------------------------------------------------------------  target    LSDLETGMVYLTAAAKQNRILLEHIQGHALYRSFDEIEREQFDDAMIEAHQLVSMTDLISQILQQLSASYNNILNNNLND 6akf.4    --------------------------------------------------------------------------------  target    NLTTLTIISVLLAVLAVVTGFFGMNVPLPLTDEPHAWLYISLASAGLWIVLSLLLRKIAKKS 6akf.4    AARALIVVSILLAAFGLLVALVGAQA------------------------------------ ``` | | | | | | | | | | | | | | | | | | | | | | | | | | | | | | | | | | | | | | | | | | | | | | | | | |
|  | 3hb3.1.A | Cytochrome c oxidase subunit 1-beta  *High resolution crystal structure of Paracoccus denitrificans cytochrome c oxidase* | 0.00 |  | 17.86 | 0.09 | 234-261 | X-ray | 2.25 | hetero-oligomer | 2 x HEA, 3 x CU1, 1 x MN, 1 x CA, 9 x LDA, 14 x LMT | HHblits | 0.28 |
| ``` target    MVLEKQLGNGCTWIDLDLGKLNKLEDLSEIYGLDKETIEYALDRNERAHMDYHRESETVTFIYNVLDVKKDKAYYETFPM 3hb3.1    --------------------------------------------------------------------------------  target    TFIVEHRRLITISNTKNAYVIEQMTRYLENHDTLSIYKFLFASLEIISNAYYPVIEQMDKSRDEVNDLLRQRTTKKNLFV 3hb3.1    --------------------------------------------------------------------------------  target    LSDLETGMVYLTAAAKQNRILLEHIQGHALYRSFDEIEREQFDDAMIEAHQLVSMTDLISQILQQLSASYNNILNNNLND 3hb3.1    -------------------------------------------------------------------------AFPRLNN  target    NLTTLTIISVLLAVLAVVTGFFGMNVPLPLTDEPHAWLYISLASAGLWIVLSLLLRKIAKKS 3hb3.1    LSYWMYVCGVALGVASLLAPG----------------------------------------- ``` | | | | | | | | | | | | | | | | | | | | | | | | | | | | | | | | | | | | | | | | | | | | | | | | | |
|  | 1m56.1.A | CYTOCHROME C OXIDASE  *Structure of cytochrome c oxidase from Rhodobactor sphaeroides (Wild Type)* | 0.00 |  | 17.86 | 0.09 | 234-261 | X-ray | 2.30 | hetero-oligomer | 3 x CU, 1 x MG, 1 x CA, 2 x HEA, 6 x PEH | HHblits | 0.27 |
| ``` target    MVLEKQLGNGCTWIDLDLGKLNKLEDLSEIYGLDKETIEYALDRNERAHMDYHRESETVTFIYNVLDVKKDKAYYETFPM 1m56.1    --------------------------------------------------------------------------------  target    TFIVEHRRLITISNTKNAYVIEQMTRYLENHDTLSIYKFLFASLEIISNAYYPVIEQMDKSRDEVNDLLRQRTTKKNLFV 1m56.1    --------------------------------------------------------------------------------  target    LSDLETGMVYLTAAAKQNRILLEHIQGHALYRSFDEIEREQFDDAMIEAHQLVSMTDLISQILQQLSASYNNILNNNLND 1m56.1    -------------------------------------------------------------------------AFPRMNN  target    NLTTLTIISVLLAVLAVVTGFFGMNVPLPLTDEPHAWLYISLASAGLWIVLSLLLRKIAKKS 1m56.1    LSYWLYVAGTSLAVASLFAPG----------------------------------------- ``` | | | | | | | | | | | | | | | | | | | | | | | | | | | | | | | | | | | | | | | | | | | | | | | | | |
|  | 3fyi.1.A | Cytochrome c oxidase subunit 1  *Catalytic core subunits (I and II) of cytochrome C oxidase from Rhodobacter sphaeroides in the reduced state bound with cyanide* | 0.00 |  | 17.86 | 0.09 | 234-261 | X-ray | 2.20 | hetero-1-1-mer | 2 x HEA, 3 x CU1, 1 x MG, 1 x CA, 1 x CYN, 6 x DMU, 5 x TRD, 1 x HTO, 2 x CD | HHblits | 0.27 |
| ``` target    MVLEKQLGNGCTWIDLDLGKLNKLEDLSEIYGLDKETIEYALDRNERAHMDYHRESETVTFIYNVLDVKKDKAYYETFPM 3fyi.1    --------------------------------------------------------------------------------  target    TFIVEHRRLITISNTKNAYVIEQMTRYLENHDTLSIYKFLFASLEIISNAYYPVIEQMDKSRDEVNDLLRQRTTKKNLFV 3fyi.1    --------------------------------------------------------------------------------  target    LSDLETGMVYLTAAAKQNRILLEHIQGHALYRSFDEIEREQFDDAMIEAHQLVSMTDLISQILQQLSASYNNILNNNLND 3fyi.1    -------------------------------------------------------------------------AFPRMNN  target    NLTTLTIISVLLAVLAVVTGFFGMNVPLPLTDEPHAWLYISLASAGLWIVLSLLLRKIAKKS 3fyi.1    LSYWLYVAGTSLAVASLFAPG----------------------------------------- ``` | | | | | | | | | | | | | | | | | | | | | | | | | | | | | | | | | | | | | | | | | | | | | | | | | |
|  | 1m57.1.A | CYTOCHROME C OXIDASE  *Structure of cytochrome c oxidase from Rhodobacter sphaeroides (EQ(I-286) mutant))* | 0.00 |  | 17.86 | 0.09 | 234-261 | X-ray | 3.00 | hetero-oligomer | 3 x CU, 1 x MG, 1 x CA, 2 x HEA, 6 x PEH | HHblits | 0.27 |
| ``` target    MVLEKQLGNGCTWIDLDLGKLNKLEDLSEIYGLDKETIEYALDRNERAHMDYHRESETVTFIYNVLDVKKDKAYYETFPM 1m57.1    --------------------------------------------------------------------------------  target    TFIVEHRRLITISNTKNAYVIEQMTRYLENHDTLSIYKFLFASLEIISNAYYPVIEQMDKSRDEVNDLLRQRTTKKNLFV 1m57.1    --------------------------------------------------------------------------------  target    LSDLETGMVYLTAAAKQNRILLEHIQGHALYRSFDEIEREQFDDAMIEAHQLVSMTDLISQILQQLSASYNNILNNNLND 1m57.1    -------------------------------------------------------------------------AFPRMNN  target    NLTTLTIISVLLAVLAVVTGFFGMNVPLPLTDEPHAWLYISLASAGLWIVLSLLLRKIAKKS 1m57.1    LSYWLYVAGTSLAVASLFAPG----------------------------------------- ``` | | | | | | | | | | | | | | | | | | | | | | | | | | | | | | | | | | | | | | | | | | | | | | | | | |
|  | 3ehb.1.A | Cytochrome c oxidase subunit 1-beta  *A D-Pathway Mutation Decouples the Paracoccus Denitrificans Cytochrome c Oxidase by Altering the side chain orientation of a distant, conserved Glutamate* | 0.00 |  | 14.29 | 0.09 | 234-261 | X-ray | 2.32 | hetero-1-1-1-1-mer | 2 x HEA, 3 x CU, 1 x MG, 1 x CA, 9 x LDA, 12 x LMT, 1 x PER | HHblits | 0.27 |
| ``` target    MVLEKQLGNGCTWIDLDLGKLNKLEDLSEIYGLDKETIEYALDRNERAHMDYHRESETVTFIYNVLDVKKDKAYYETFPM 3ehb.1    --------------------------------------------------------------------------------  target    TFIVEHRRLITISNTKNAYVIEQMTRYLENHDTLSIYKFLFASLEIISNAYYPVIEQMDKSRDEVNDLLRQRTTKKNLFV 3ehb.1    --------------------------------------------------------------------------------  target    LSDLETGMVYLTAAAKQNRILLEHIQGHALYRSFDEIEREQFDDAMIEAHQLVSMTDLISQILQQLSASYNNILNNNLND 3ehb.1    -------------------------------------------------------------------------AFPRLDN  target    NLTTLTIISVLLAVLAVVTGFFGMNVPLPLTDEPHAWLYISLASAGLWIVLSLLLRKIAKKS 3ehb.1    LSYWMYVCGVALGVASLLAPG----------------------------------------- ``` | | | | | | | | | | | | | | | | | | | | | | | | | | | | | | | | | | | | | | | | | | | | | | | | | |
|  | 4jo7.1.C | Nucleoporin p58/p45  *Crystal structure of the human Nup49CCS2+3\* Nup57CCS3\* complex with 2:2 stoichiometry* | 0.02 |  | 14.81 | 0.09 | 125-151 | X-ray | 1.75 | hetero-oligomer |  | HHblits | 0.30 |
| ``` target    MVLEKQLGNGCTWIDLDLGKLNKLEDLSEIYGLDKETIEYALDRNERAHMDYHRESETVTFIYNVLDVKKDKAYYETFPM 4jo7.1    --------------------------------------------------------------------------------  target    TFIVEHRRLITISNTKNAYVIEQMTRYLENHDTLSIYKFLFASLEIISNAYYPVIEQMDKSRDEVNDLLRQRTTKKNLFV 4jo7.1    --------------------------------------------RILVQQFEVQLQQYRQQIEELENHLAT---------  target    LSDLETGMVYLTAAAKQNRILLEHIQGHALYRSFDEIEREQFDDAMIEAHQLVSMTDLISQILQQLSASYNNILNNNLND 4jo7.1    --------------------------------------------------------------------------------  target    NLTTLTIISVLLAVLAVVTGFFGMNVPLPLTDEPHAWLYISLASAGLWIVLSLLLRKIAKKS 4jo7.1    -------------------------------------------------------------- ``` | | | | | | | | | | | | | | | | | | | | | | | | | | | | | | | | | | | | | | | | | | | | | | | | | |
|  | 4jo7.1.D | Nucleoporin p58/p45  *Crystal structure of the human Nup49CCS2+3\* Nup57CCS3\* complex with 2:2 stoichiometry* | 0.02 |  | 14.81 | 0.09 | 125-151 | X-ray | 1.75 | hetero-oligomer |  | HHblits | 0.30 |
| ``` target    MVLEKQLGNGCTWIDLDLGKLNKLEDLSEIYGLDKETIEYALDRNERAHMDYHRESETVTFIYNVLDVKKDKAYYETFPM 4jo7.1    --------------------------------------------------------------------------------  target    TFIVEHRRLITISNTKNAYVIEQMTRYLENHDTLSIYKFLFASLEIISNAYYPVIEQMDKSRDEVNDLLRQRTTKKNLFV 4jo7.1    --------------------------------------------RILVQQFEVQLQQYRQQIEELENHLAT---------  target    LSDLETGMVYLTAAAKQNRILLEHIQGHALYRSFDEIEREQFDDAMIEAHQLVSMTDLISQILQQLSASYNNILNNNLND 4jo7.1    --------------------------------------------------------------------------------  target    NLTTLTIISVLLAVLAVVTGFFGMNVPLPLTDEPHAWLYISLASAGLWIVLSLLLRKIAKKS 4jo7.1    -------------------------------------------------------------- ``` | | | | | | | | | | | | | | | | | | | | | | | | | | | | | | | | | | | | | | | | | | | | | | | | | |
|  | 4jo7.2.A | Nucleoporin p58/p45  *Crystal structure of the human Nup49CCS2+3\* Nup57CCS3\* complex with 2:2 stoichiometry* | 0.02 |  | 14.81 | 0.09 | 125-151 | X-ray | 1.75 | hetero-oligomer |  | HHblits | 0.30 |
| ``` target    MVLEKQLGNGCTWIDLDLGKLNKLEDLSEIYGLDKETIEYALDRNERAHMDYHRESETVTFIYNVLDVKKDKAYYETFPM 4jo7.2    --------------------------------------------------------------------------------  target    TFIVEHRRLITISNTKNAYVIEQMTRYLENHDTLSIYKFLFASLEIISNAYYPVIEQMDKSRDEVNDLLRQRTTKKNLFV 4jo7.2    --------------------------------------------RILVQQFEVQLQQYRQQIEELENHLAT---------  target    LSDLETGMVYLTAAAKQNRILLEHIQGHALYRSFDEIEREQFDDAMIEAHQLVSMTDLISQILQQLSASYNNILNNNLND 4jo7.2    --------------------------------------------------------------------------------  target    NLTTLTIISVLLAVLAVVTGFFGMNVPLPLTDEPHAWLYISLASAGLWIVLSLLLRKIAKKS 4jo7.2    -------------------------------------------------------------- ``` | | | | | | | | | | | | | | | | | | | | | | | | | | | | | | | | | | | | | | | | | | | | | | | | | |
|  | 4jo7.2.C | Nucleoporin p58/p45  *Crystal structure of the human Nup49CCS2+3\* Nup57CCS3\* complex with 2:2 stoichiometry* | 0.02 |  | 14.81 | 0.09 | 125-151 | X-ray | 1.75 | hetero-oligomer |  | HHblits | 0.30 |
| ``` target    MVLEKQLGNGCTWIDLDLGKLNKLEDLSEIYGLDKETIEYALDRNERAHMDYHRESETVTFIYNVLDVKKDKAYYETFPM 4jo7.2    --------------------------------------------------------------------------------  target    TFIVEHRRLITISNTKNAYVIEQMTRYLENHDTLSIYKFLFASLEIISNAYYPVIEQMDKSRDEVNDLLRQRTTKKNLFV 4jo7.2    --------------------------------------------RILVQQFEVQLQQYRQQIEELENHLAT---------  target    LSDLETGMVYLTAAAKQNRILLEHIQGHALYRSFDEIEREQFDDAMIEAHQLVSMTDLISQILQQLSASYNNILNNNLND 4jo7.2    --------------------------------------------------------------------------------  target    NLTTLTIISVLLAVLAVVTGFFGMNVPLPLTDEPHAWLYISLASAGLWIVLSLLLRKIAKKS 4jo7.2    -------------------------------------------------------------- ``` | | | | | | | | | | | | | | | | | | | | | | | | | | | | | | | | | | | | | | | | | | | | | | | | | |
|  | 4jq5.1.B | Nucleoporin p58/p45  *Crystal structure of the human Nup49CCS2+3\* coiled-coil segment* | 0.02 |  | 14.81 | 0.09 | 125-151 | X-ray | 2.19 | homo-tetramer |  | HHblits | 0.30 |
| ``` target    MVLEKQLGNGCTWIDLDLGKLNKLEDLSEIYGLDKETIEYALDRNERAHMDYHRESETVTFIYNVLDVKKDKAYYETFPM 4jq5.1    --------------------------------------------------------------------------------  target    TFIVEHRRLITISNTKNAYVIEQMTRYLENHDTLSIYKFLFASLEIISNAYYPVIEQMDKSRDEVNDLLRQRTTKKNLFV 4jq5.1    --------------------------------------------RILVQQFEVQLQQYRQQIEELENHLAT---------  target    LSDLETGMVYLTAAAKQNRILLEHIQGHALYRSFDEIEREQFDDAMIEAHQLVSMTDLISQILQQLSASYNNILNNNLND 4jq5.1    --------------------------------------------------------------------------------  target    NLTTLTIISVLLAVLAVVTGFFGMNVPLPLTDEPHAWLYISLASAGLWIVLSLLLRKIAKKS 4jq5.1    -------------------------------------------------------------- ``` | | | | | | | | | | | | | | | | | | | | | | | | | | | | | | | | | | | | | | | | | | | | | | | | | |
|  | 4jq5.1.A | Nucleoporin p58/p45  *Crystal structure of the human Nup49CCS2+3\* coiled-coil segment* | 0.02 |  | 14.81 | 0.09 | 125-151 | X-ray | 2.19 | homo-tetramer |  | HHblits | 0.30 |
| ``` target    MVLEKQLGNGCTWIDLDLGKLNKLEDLSEIYGLDKETIEYALDRNERAHMDYHRESETVTFIYNVLDVKKDKAYYETFPM 4jq5.1    --------------------------------------------------------------------------------  target    TFIVEHRRLITISNTKNAYVIEQMTRYLENHDTLSIYKFLFASLEIISNAYYPVIEQMDKSRDEVNDLLRQRTTKKNLFV 4jq5.1    --------------------------------------------RILVQQFEVQLQQYRQQIEELENHLAT---------  target    LSDLETGMVYLTAAAKQNRILLEHIQGHALYRSFDEIEREQFDDAMIEAHQLVSMTDLISQILQQLSASYNNILNNNLND 4jq5.1    --------------------------------------------------------------------------------  target    NLTTLTIISVLLAVLAVVTGFFGMNVPLPLTDEPHAWLYISLASAGLWIVLSLLLRKIAKKS 4jq5.1    -------------------------------------------------------------- ``` | | | | | | | | | | | | | | | | | | | | | | | | | | | | | | | | | | | | | | | | | | | | | | | | | |
|  | 4jq5.1.C | Nucleoporin p58/p45  *Crystal structure of the human Nup49CCS2+3\* coiled-coil segment* | 0.02 |  | 14.81 | 0.09 | 125-151 | X-ray | 2.19 | homo-tetramer |  | HHblits | 0.30 |
| ``` target    MVLEKQLGNGCTWIDLDLGKLNKLEDLSEIYGLDKETIEYALDRNERAHMDYHRESETVTFIYNVLDVKKDKAYYETFPM 4jq5.1    --------------------------------------------------------------------------------  target    TFIVEHRRLITISNTKNAYVIEQMTRYLENHDTLSIYKFLFASLEIISNAYYPVIEQMDKSRDEVNDLLRQRTTKKNLFV 4jq5.1    --------------------------------------------RILVQQFEVQLQQYRQQIEELENHLAT---------  target    LSDLETGMVYLTAAAKQNRILLEHIQGHALYRSFDEIEREQFDDAMIEAHQLVSMTDLISQILQQLSASYNNILNNNLND 4jq5.1    --------------------------------------------------------------------------------  target    NLTTLTIISVLLAVLAVVTGFFGMNVPLPLTDEPHAWLYISLASAGLWIVLSLLLRKIAKKS 4jq5.1    -------------------------------------------------------------- ``` | | | | | | | | | | | | | | | | | | | | | | | | | | | | | | | | | | | | | | | | | | | | | | | | | |
|  | 4jq5.2.A | Nucleoporin p58/p45  *Crystal structure of the human Nup49CCS2+3\* coiled-coil segment* | 0.02 |  | 14.81 | 0.09 | 125-151 | X-ray | 2.19 | homo-tetramer |  | HHblits | 0.30 |
| ``` target    MVLEKQLGNGCTWIDLDLGKLNKLEDLSEIYGLDKETIEYALDRNERAHMDYHRESETVTFIYNVLDVKKDKAYYETFPM 4jq5.2    --------------------------------------------------------------------------------  target    TFIVEHRRLITISNTKNAYVIEQMTRYLENHDTLSIYKFLFASLEIISNAYYPVIEQMDKSRDEVNDLLRQRTTKKNLFV 4jq5.2    --------------------------------------------RILVQQFEVQLQQYRQQIEELENHLAT---------  target    LSDLETGMVYLTAAAKQNRILLEHIQGHALYRSFDEIEREQFDDAMIEAHQLVSMTDLISQILQQLSASYNNILNNNLND 4jq5.2    --------------------------------------------------------------------------------  target    NLTTLTIISVLLAVLAVVTGFFGMNVPLPLTDEPHAWLYISLASAGLWIVLSLLLRKIAKKS 4jq5.2    -------------------------------------------------------------- ``` | | | | | | | | | | | | | | | | | | | | | | | | | | | | | | | | | | | | | | | | | | | | | | | | | |
|  | 4jq5.2.B | Nucleoporin p58/p45  *Crystal structure of the human Nup49CCS2+3\* coiled-coil segment* | 0.02 |  | 14.81 | 0.09 | 125-151 | X-ray | 2.19 | homo-tetramer |  | HHblits | 0.30 |
| ``` target    MVLEKQLGNGCTWIDLDLGKLNKLEDLSEIYGLDKETIEYALDRNERAHMDYHRESETVTFIYNVLDVKKDKAYYETFPM 4jq5.2    --------------------------------------------------------------------------------  target    TFIVEHRRLITISNTKNAYVIEQMTRYLENHDTLSIYKFLFASLEIISNAYYPVIEQMDKSRDEVNDLLRQRTTKKNLFV 4jq5.2    --------------------------------------------RILVQQFEVQLQQYRQQIEELENHLAT---------  target    LSDLETGMVYLTAAAKQNRILLEHIQGHALYRSFDEIEREQFDDAMIEAHQLVSMTDLISQILQQLSASYNNILNNNLND 4jq5.2    --------------------------------------------------------------------------------  target    NLTTLTIISVLLAVLAVVTGFFGMNVPLPLTDEPHAWLYISLASAGLWIVLSLLLRKIAKKS 4jq5.2    -------------------------------------------------------------- ``` | | | | | | | | | | | | | | | | | | | | | | | | | | | | | | | | | | | | | | | | | | | | | | | | | |
|  | 4jq5.2.C | Nucleoporin p58/p45  *Crystal structure of the human Nup49CCS2+3\* coiled-coil segment* | 0.02 |  | 14.81 | 0.09 | 125-151 | X-ray | 2.19 | homo-tetramer |  | HHblits | 0.30 |
| ``` target    MVLEKQLGNGCTWIDLDLGKLNKLEDLSEIYGLDKETIEYALDRNERAHMDYHRESETVTFIYNVLDVKKDKAYYETFPM 4jq5.2    --------------------------------------------------------------------------------  target    TFIVEHRRLITISNTKNAYVIEQMTRYLENHDTLSIYKFLFASLEIISNAYYPVIEQMDKSRDEVNDLLRQRTTKKNLFV 4jq5.2    --------------------------------------------RILVQQFEVQLQQYRQQIEELENHLAT---------  target    LSDLETGMVYLTAAAKQNRILLEHIQGHALYRSFDEIEREQFDDAMIEAHQLVSMTDLISQILQQLSASYNNILNNNLND 4jq5.2    --------------------------------------------------------------------------------  target    NLTTLTIISVLLAVLAVVTGFFGMNVPLPLTDEPHAWLYISLASAGLWIVLSLLLRKIAKKS 4jq5.2    -------------------------------------------------------------- ``` | | | | | | | | | | | | | | | | | | | | | | | | | | | | | | | | | | | | | | | | | | | | | | | | | |
|  | 4jq5.3.A | Nucleoporin p58/p45  *Crystal structure of the human Nup49CCS2+3\* coiled-coil segment* | 0.02 |  | 14.81 | 0.09 | 125-151 | X-ray | 2.19 | homo-tetramer |  | HHblits | 0.30 |
| ``` target    MVLEKQLGNGCTWIDLDLGKLNKLEDLSEIYGLDKETIEYALDRNERAHMDYHRESETVTFIYNVLDVKKDKAYYETFPM 4jq5.3    --------------------------------------------------------------------------------  target    TFIVEHRRLITISNTKNAYVIEQMTRYLENHDTLSIYKFLFASLEIISNAYYPVIEQMDKSRDEVNDLLRQRTTKKNLFV 4jq5.3    --------------------------------------------RILVQQFEVQLQQYRQQIEELENHLAT---------  target    LSDLETGMVYLTAAAKQNRILLEHIQGHALYRSFDEIEREQFDDAMIEAHQLVSMTDLISQILQQLSASYNNILNNNLND 4jq5.3    --------------------------------------------------------------------------------  target    NLTTLTIISVLLAVLAVVTGFFGMNVPLPLTDEPHAWLYISLASAGLWIVLSLLLRKIAKKS 4jq5.3    -------------------------------------------------------------- ``` | | | | | | | | | | | | | | | | | | | | | | | | | | | | | | | | | | | | | | | | | | | | | | | | | |
|  | 4jo9.1.B | Nucleoporin p58/p45  *Crystal structure of the human Nup49CCS2+3\* Nup57CCS3\* complex 1:2 stoichiometry* | 0.02 |  | 14.81 | 0.09 | 125-151 | X-ray | 2.50 | hetero-oligomer |  | HHblits | 0.30 |
| ``` target    MVLEKQLGNGCTWIDLDLGKLNKLEDLSEIYGLDKETIEYALDRNERAHMDYHRESETVTFIYNVLDVKKDKAYYETFPM 4jo9.1    --------------------------------------------------------------------------------  target    TFIVEHRRLITISNTKNAYVIEQMTRYLENHDTLSIYKFLFASLEIISNAYYPVIEQMDKSRDEVNDLLRQRTTKKNLFV 4jo9.1    --------------------------------------------RILVQQFEVQLQQYRQQIEELENHLAT---------  target    LSDLETGMVYLTAAAKQNRILLEHIQGHALYRSFDEIEREQFDDAMIEAHQLVSMTDLISQILQQLSASYNNILNNNLND 4jo9.1    --------------------------------------------------------------------------------  target    NLTTLTIISVLLAVLAVVTGFFGMNVPLPLTDEPHAWLYISLASAGLWIVLSLLLRKIAKKS 4jo9.1    -------------------------------------------------------------- ``` | | | | | | | | | | | | | | | | | | | | | | | | | | | | | | | | | | | | | | | | | | | | | | | | | |
|  | 6yar.1.C | Bacterial cellulose secretion regulator BcsR  *Crystal structure of a Selenium-derivatized complex of the bacterial cellulose secretion regulators BcsR and BcsQ, crystallized in the presence of AppCp* | 0.00 |  | 14.29 | 0.09 | 19-46 | X-ray | 1.90 | hetero-2-2-mer | 2 x MG, 2 x ATP | HHblits | 0.26 |
| ``` target    MVLEKQLGNGCTWIDLDLGKLNKLEDLSEIYGLDKETIEYALDRNERAHMDYHRESETVTFIYNVLDVKKDKAYYETFPM 6yar.1    ------------------IFQNDIVALKQAFSLPDIDYADISQREQ----------------------------------  target    TFIVEHRRLITISNTKNAYVIEQMTRYLENHDTLSIYKFLFASLEIISNAYYPVIEQMDKSRDEVNDLLRQRTTKKNLFV 6yar.1    --------------------------------------------------------------------------------  target    LSDLETGMVYLTAAAKQNRILLEHIQGHALYRSFDEIEREQFDDAMIEAHQLVSMTDLISQILQQLSASYNNILNNNLND 6yar.1    --------------------------------------------------------------------------------  target    NLTTLTIISVLLAVLAVVTGFFGMNVPLPLTDEPHAWLYISLASAGLWIVLSLLLRKIAKKS 6yar.1    -------------------------------------------------------------- ``` | | | | | | | | | | | | | | | | | | | | | | | | | | | | | | | | | | | | | | | | | | | | | | | | | |
|  | 6yay.1.C | Bacterial cellulose secretion regulator BcsR  *Crystal structure of a Selenium-derivatized complex of the bacterial cellulose secretion regulators BcsR and BcsQ, crystallized in the presence of ADP* | 0.00 |  | 14.29 | 0.09 | 19-46 | X-ray | 2.09 | hetero-2-2-mer | 2 x MG, 2 x ATP | HHblits | 0.26 |
| ``` target    MVLEKQLGNGCTWIDLDLGKLNKLEDLSEIYGLDKETIEYALDRNERAHMDYHRESETVTFIYNVLDVKKDKAYYETFPM 6yay.1    ------------------IFQNDIVALKQAFSLPDIDYADISQREQ----------------------------------  target    TFIVEHRRLITISNTKNAYVIEQMTRYLENHDTLSIYKFLFASLEIISNAYYPVIEQMDKSRDEVNDLLRQRTTKKNLFV 6yay.1    --------------------------------------------------------------------------------  target    LSDLETGMVYLTAAAKQNRILLEHIQGHALYRSFDEIEREQFDDAMIEAHQLVSMTDLISQILQQLSASYNNILNNNLND 6yay.1    --------------------------------------------------------------------------------  target    NLTTLTIISVLLAVLAVVTGFFGMNVPLPLTDEPHAWLYISLASAGLWIVLSLLLRKIAKKS 6yay.1    -------------------------------------------------------------- ``` | | | | | | | | | | | | | | | | | | | | | | | | | | | | | | | | | | | | | | | | | | | | | | | | | |
|  | 6yb3.1.C | Bacterial cellulose secretion regulator BcsR  *Crystal structure of a native BcsRQ complex purified and crystallized in the absence of nucleotide* | 0.00 |  | 14.29 | 0.09 | 19-46 | X-ray | 1.59 | hetero-2-2-mer | 2 x MG, 2 x ATP | HHblits | 0.26 |
| ``` target    MVLEKQLGNGCTWIDLDLGKLNKLEDLSEIYGLDKETIEYALDRNERAHMDYHRESETVTFIYNVLDVKKDKAYYETFPM 6yb3.1    ------------------IFQNDIVALKQAFSLPDIDYADISQREQ----------------------------------  target    TFIVEHRRLITISNTKNAYVIEQMTRYLENHDTLSIYKFLFASLEIISNAYYPVIEQMDKSRDEVNDLLRQRTTKKNLFV 6yb3.1    --------------------------------------------------------------------------------  target    LSDLETGMVYLTAAAKQNRILLEHIQGHALYRSFDEIEREQFDDAMIEAHQLVSMTDLISQILQQLSASYNNILNNNLND 6yb3.1    --------------------------------------------------------------------------------  target    NLTTLTIISVLLAVLAVVTGFFGMNVPLPLTDEPHAWLYISLASAGLWIVLSLLLRKIAKKS 6yb3.1    -------------------------------------------------------------- ``` | | | | | | | | | | | | | | | | | | | | | | | | | | | | | | | | | | | | | | | | | | | | | | | | | |
|  | 6yb3.1.D | Bacterial cellulose secretion regulator BcsR  *Crystal structure of a native BcsRQ complex purified and crystallized in the absence of nucleotide* | 0.00 |  | 14.29 | 0.09 | 19-46 | X-ray | 1.59 | hetero-2-2-mer | 2 x MG, 2 x ATP | HHblits | 0.26 |
| ``` target    MVLEKQLGNGCTWIDLDLGKLNKLEDLSEIYGLDKETIEYALDRNERAHMDYHRESETVTFIYNVLDVKKDKAYYETFPM 6yb3.1    ------------------IFQNDIVALKQAFSLPDIDYADISQREQ----------------------------------  target    TFIVEHRRLITISNTKNAYVIEQMTRYLENHDTLSIYKFLFASLEIISNAYYPVIEQMDKSRDEVNDLLRQRTTKKNLFV 6yb3.1    --------------------------------------------------------------------------------  target    LSDLETGMVYLTAAAKQNRILLEHIQGHALYRSFDEIEREQFDDAMIEAHQLVSMTDLISQILQQLSASYNNILNNNLND 6yb3.1    --------------------------------------------------------------------------------  target    NLTTLTIISVLLAVLAVVTGFFGMNVPLPLTDEPHAWLYISLASAGLWIVLSLLLRKIAKKS 6yb3.1    -------------------------------------------------------------- ``` | | | | | | | | | | | | | | | | | | | | | | | | | | | | | | | | | | | | | | | | | | | | | | | | | |
|  | 6yb5.1.D | Bacterial cellulose secretion regulator BcsR  *Orthorhombic crystal structure of a native BcsRQ complex crystallized in the presence of ADP* | 0.00 |  | 14.29 | 0.09 | 19-46 | X-ray | 1.59 | hetero-3-2-mer | 2 x MG, 2 x ATP | HHblits | 0.26 |
| ``` target    MVLEKQLGNGCTWIDLDLGKLNKLEDLSEIYGLDKETIEYALDRNERAHMDYHRESETVTFIYNVLDVKKDKAYYETFPM 6yb5.1    ------------------IFQNDIVALKQAFSLPDIDYADISQREQ----------------------------------  target    TFIVEHRRLITISNTKNAYVIEQMTRYLENHDTLSIYKFLFASLEIISNAYYPVIEQMDKSRDEVNDLLRQRTTKKNLFV 6yb5.1    --------------------------------------------------------------------------------  target    LSDLETGMVYLTAAAKQNRILLEHIQGHALYRSFDEIEREQFDDAMIEAHQLVSMTDLISQILQQLSASYNNILNNNLND 6yb5.1    --------------------------------------------------------------------------------  target    NLTTLTIISVLLAVLAVVTGFFGMNVPLPLTDEPHAWLYISLASAGLWIVLSLLLRKIAKKS 6yb5.1    -------------------------------------------------------------- ``` | | | | | | | | | | | | | | | | | | | | | | | | | | | | | | | | | | | | | | | | | | | | | | | | | |
|  | 2rt6.1.A | Primosomal replication protein N''  *Backbone 1H, 13C, and 15N Chemical Shift Assignments for PriC N-terminal domain* | 0.00 |  | 0.00 | 0.10 | 121-149 | NMR | 0.00 | monomer |  | HHblits | 0.24 |
| ``` target    MVLEKQLGNGCTWIDLDLGKLNKLEDLSEIYGLDKETIEYALDRNERAHMDYHRESETVTFIYNVLDVKKDKAYYETFPM 2rt6.1    --------------------------------------------------------------------------------  target    TFIVEHRRLITISNTKNAYVIEQMTRYLENHDTLSIYKFLFASLEIISNAYYPVIEQMDKSRDEVNDLLRQRTTKKNLFV 2rt6.1    ----------------------------------------RHLFQTRATTLQACLDEAGDNLAALRHAV-----------  target    LSDLETGMVYLTAAAKQNRILLEHIQGHALYRSFDEIEREQFDDAMIEAHQLVSMTDLISQILQQLSASYNNILNNNLND 2rt6.1    --------------------------------------------------------------------------------  target    NLTTLTIISVLLAVLAVVTGFFGMNVPLPLTDEPHAWLYISLASAGLWIVLSLLLRKIAKKS 2rt6.1    -------------------------------------------------------------- ``` | | | | | | | | | | | | | | | | | | | | | | | | | | | | | | | | | | | | | | | | | | | | | | | | | |
|  | 6ov2.1.A | Claudin-9  *Crystal structure of human claudin-9 in complex with Clostridium perfringens entertoxin C-terminal domain in closed form* | 0.01 |  | 28.00 | 0.08 | 241-265 | X-ray | 3.20 | hetero-1-1-mer |  | HHblits | 0.35 |
| ``` target    MVLEKQLGNGCTWIDLDLGKLNKLEDLSEIYGLDKETIEYALDRNERAHMDYHRESETVTFIYNVLDVKKDKAYYETFPM 6ov2.1    --------------------------------------------------------------------------------  target    TFIVEHRRLITISNTKNAYVIEQMTRYLENHDTLSIYKFLFASLEIISNAYYPVIEQMDKSRDEVNDLLRQRTTKKNLFV 6ov2.1    --------------------------------------------------------------------------------  target    LSDLETGMVYLTAAAKQNRILLEHIQGHALYRSFDEIEREQFDDAMIEAHQLVSMTDLISQILQQLSASYNNILNNNLND 6ov2.1    --------------------------------------------------------------------------------  target    NLTTLTIISVLLAVLAVVTGFFGMNVPLPLTDEPHAWLYISLASAGLWIVLSLLLRKIAKKS 6ov2.1    AARALCVIALLLALLGLLVAITGAQ------------------------------------- ``` | | | | | | | | | | | | | | | | | | | | | | | | | | | | | | | | | | | | | | | | | | | | | | | | | |
|  | 6ov3.1.A | Claudin-9  *Crystal structure of human claudin-9 in complex with Clostridium perfringens entertoxin C-terminal domain in open form* | 0.01 |  | 28.00 | 0.08 | 241-265 | X-ray | 3.25 | hetero-1-1-mer |  | HHblits | 0.35 |
| ``` target    MVLEKQLGNGCTWIDLDLGKLNKLEDLSEIYGLDKETIEYALDRNERAHMDYHRESETVTFIYNVLDVKKDKAYYETFPM 6ov3.1    --------------------------------------------------------------------------------  target    TFIVEHRRLITISNTKNAYVIEQMTRYLENHDTLSIYKFLFASLEIISNAYYPVIEQMDKSRDEVNDLLRQRTTKKNLFV 6ov3.1    --------------------------------------------------------------------------------  target    LSDLETGMVYLTAAAKQNRILLEHIQGHALYRSFDEIEREQFDDAMIEAHQLVSMTDLISQILQQLSASYNNILNNNLND 6ov3.1    --------------------------------------------------------------------------------  target    NLTTLTIISVLLAVLAVVTGFFGMNVPLPLTDEPHAWLYISLASAGLWIVLSLLLRKIAKKS 6ov3.1    AARALCVIALLLALLGLLVAITGAQ------------------------------------- ``` | | | | | | | | | | | | | | | | | | | | | | | | | | | | | | | | | | | | | | | | | | | | | | | | | |
|  | 7jr7.1.A | ATP-binding cassette sub-family G member 5  *Cryo-EM structure of ABCG5/G8 in complex with Fab 2E10 and 11F4* | 0.00 |  | 14.81 | 0.09 | 235-261 | EM | 0.00 | hetero-1-1-1-1-1-1-… |  | HHblits | 0.29 |
| ``` target    MVLEKQLGNGCTWIDLDLGKLNKLEDLSEIYGLDKETIEYALDRNERAHMDYHRESETVTFIYNVLDVKKDKAYYETFPM 7jr7.1    --------------------------------------------------------------------------------  target    TFIVEHRRLITISNTKNAYVIEQMTRYLENHDTLSIYKFLFASLEIISNAYYPVIEQMDKSRDEVNDLLRQRTTKKNLFV 7jr7.1    --------------------------------------------------------------------------------  target    LSDLETGMVYLTAAAKQNRILLEHIQGHALYRSFDEIEREQFDDAMIEAHQLVSMTDLISQILQQLSASYNNILNNNLND 7jr7.1    --------------------------------------------------------------------------LGIVQN  target    NLTTLTIISVLLAVLAVVT-GFFGMNVPLPLTDEPHAWLYISLASAGLWIVLSLLLRKIAKKS 7jr7.1    PNIVNSVVALLSIAGVLVGSGF----------------------------------------- ``` | | | | | | | | | | | | | | | | | | | | | | | | | | | | | | | | | | | | | | | | | | | | | | | | | |
|  | 5do7.1.B | ATP-binding cassette sub-family G member 8  *Crystal Structure of the Human Sterol Transporter ABCG5/ABCG8* | 0.01 |  | 18.52 | 0.09 | 243-270 | X-ray | 3.93 | hetero-1-1-mer |  | HHblits | 0.29 |
| ``` target    MVLEKQLGNGCTWIDLDLGKLNKLEDLSEIYGLDKETIEYALDRNERAHMDYHRESETVTFIYNVLDVKKDKAYYETFPM 5do7.1    --------------------------------------------------------------------------------  target    TFIVEHRRLITISNTKNAYVIEQMTRYLENHDTLSIYKFLFASLEIISNAYYPVIEQMDKSRDEVNDLLRQRTTKKNLFV 5do7.1    --------------------------------------------------------------------------------  target    LSDLETGMVYLTAAAKQNRILLEHIQGHALYRSFDEIEREQFDDAMIEAHQLVSMTDLISQILQQLSASYNNILNNNLND 5do7.1    --------------------------------------------------------------------------------  target    NLTTLTIISVLLAVLAVVTGFFGMNVP-LPLTDEPHAWLYISLASAGLWIVLSLLLRKIAKKS 5do7.1    --MASFFSNALYNSFYLAGGFM-INLSSLWT-------------------------------- ``` | | | | | | | | | | | | | | | | | | | | | | | | | | | | | | | | | | | | | | | | | | | | | | | | | |
|  | 5do7.2.A | ATP-binding cassette sub-family G member 8  *Crystal Structure of the Human Sterol Transporter ABCG5/ABCG8* | 0.01 |  | 18.52 | 0.09 | 243-270 | X-ray | 3.93 | hetero-1-1-mer |  | HHblits | 0.29 |
| ``` target    MVLEKQLGNGCTWIDLDLGKLNKLEDLSEIYGLDKETIEYALDRNERAHMDYHRESETVTFIYNVLDVKKDKAYYETFPM 5do7.2    --------------------------------------------------------------------------------  target    TFIVEHRRLITISNTKNAYVIEQMTRYLENHDTLSIYKFLFASLEIISNAYYPVIEQMDKSRDEVNDLLRQRTTKKNLFV 5do7.2    --------------------------------------------------------------------------------  target    LSDLETGMVYLTAAAKQNRILLEHIQGHALYRSFDEIEREQFDDAMIEAHQLVSMTDLISQILQQLSASYNNILNNNLND 5do7.2    --------------------------------------------------------------------------------  target    NLTTLTIISVLLAVLAVVTGFFGMNVP-LPLTDEPHAWLYISLASAGLWIVLSLLLRKIAKKS 5do7.2    --MASFFSNALYNSFYLAGGFM-INLSSLWT-------------------------------- ``` | | | | | | | | | | | | | | | | | | | | | | | | | | | | | | | | | | | | | | | | | | | | | | | | | |
|  | 6oih.1.B | Transport permease protein  *Crystal structure of O-antigen polysaccharide ABC-transporter* | 0.01 |  | 26.92 | 0.09 | 276-301 | X-ray | 3.85 | hetero-1-1-mer | 3 x LDA | HHblits | 0.31 |
| ``` target    MVLEKQLGNGCTWIDLDLGKLNKLEDLSEIYGLDKETIEYALDRNERAHMDYHRESETVTFIYNVLDVKKDKAYYETFPM 6oih.1    --------------------------------------------------------------------------------  target    TFIVEHRRLITISNTKNAYVIEQMTRYLENHDTLSIYKFLFASLEIISNAYYPVIEQMDKSRDEVNDLLRQRTTKKNLFV 6oih.1    --------------------------------------------------------------------------------  target    LSDLETGMVYLTAAAKQNRILLEHIQGHALYRSFDEIEREQFDDAMIEAHQLVSMTDLISQILQQLSASYNNILNNNLND 6oih.1    --------------------------------------------------------------------------------  target    NLTTLTIISVLLAVLAVVTGFFGMNVPLPLTDEPHAWLYISLASAGLWIVLSLLLRKIAKKS 6oih.1    -----------------------------------SLLGFLLASPLVFFVSYYFFKKLEKD- ``` | | | | | | | | | | | | | | | | | | | | | | | | | | | | | | | | | | | | | | | | | | | | | | | | | |
|  | 6oih.2.B | Transport permease protein  *Crystal structure of O-antigen polysaccharide ABC-transporter* | 0.01 |  | 26.92 | 0.09 | 276-301 | X-ray | 3.85 | hetero-1-1-mer | 5 x LDA | HHblits | 0.31 |
| ``` target    MVLEKQLGNGCTWIDLDLGKLNKLEDLSEIYGLDKETIEYALDRNERAHMDYHRESETVTFIYNVLDVKKDKAYYETFPM 6oih.2    --------------------------------------------------------------------------------  target    TFIVEHRRLITISNTKNAYVIEQMTRYLENHDTLSIYKFLFASLEIISNAYYPVIEQMDKSRDEVNDLLRQRTTKKNLFV 6oih.2    --------------------------------------------------------------------------------  target    LSDLETGMVYLTAAAKQNRILLEHIQGHALYRSFDEIEREQFDDAMIEAHQLVSMTDLISQILQQLSASYNNILNNNLND 6oih.2    --------------------------------------------------------------------------------  target    NLTTLTIISVLLAVLAVVTGFFGMNVPLPLTDEPHAWLYISLASAGLWIVLSLLLRKIAKKS 6oih.2    -----------------------------------SLLGFLLASPLVFFVSYYFFKKLEKD- ``` | | | | | | | | | | | | | | | | | | | | | | | | | | | | | | | | | | | | | | | | | | | | | | | | | |
|  | 6lod.1.F | Uncharacterized protein ActF  *Cryo-EM structure of the air-oxidized photosynthetic alternative complex III from Roseiflexus castenholzii* | 0.01 |  | 10.71 | 0.09 | 237-264 | EM | 0.00 | hetero-1-1-1-1-1-1-… | 6 x HEC, 2 x EL6, 3 x SF4, 1 x F3S | HHblits | 0.25 |
| ``` target    MVLEKQLGNGCTWIDLDLGKLNKLEDLSEIYGLDKETIEYALDRNERAHMDYHRESETVTFIYNVLDVKKDKAYYETFPM 6lod.1    --------------------------------------------------------------------------------  target    TFIVEHRRLITISNTKNAYVIEQMTRYLENHDTLSIYKFLFASLEIISNAYYPVIEQMDKSRDEVNDLLRQRTTKKNLFV 6lod.1    --------------------------------------------------------------------------------  target    LSDLETGMVYLTAAAKQNRILLEHIQGHALYRSFDEIEREQFDDAMIEAHQLVSMTDLISQILQQLSASYNNILNNNLND 6lod.1    ----------------------------------------------------------------------------DLGK  target    NLTTLTIISVLLAVLAVVTGFFGMNVPLPLTDEPHAWLYISLASAGLWIVLSLLLRKIAKKS 6lod.1    FLFAFVAVWAYVNFSEYLIIWSGN-------------------------------------- ``` | | | | | | | | | | | | | | | | | | | | | | | | | | | | | | | | | | | | | | | | | | | | | | | | | |
|  | 6nbx.1.E | NAD(P)H-quinone oxidoreductase subunit 4L  *T.elongatus NDH (data-set 2)* | 0.01 |  | 18.52 | 0.09 | 240-266 | EM | 0.00 | hetero-1-1-1-1-1-1-… | 3 x SF4 | HHblits | 0.27 |
| ``` target    MVLEKQLGNGCTWIDLDLGKLNKLEDLSEIYGLDKETIEYALDRNERAHMDYHRESETVTFIYNVLDVKKDKAYYETFPM 6nbx.1    --------------------------------------------------------------------------------  target    TFIVEHRRLITISNTKNAYVIEQMTRYLENHDTLSIYKFLFASLEIISNAYYPVIEQMDKSRDEVNDLLRQRTTKKNLFV 6nbx.1    --------------------------------------------------------------------------------  target    LSDLETGMVYLTAAAKQNRILLEHIQGHALYRSFDEIEREQFDDAMIEAHQLVSMTDLISQILQQLSASYNNILNNNLND 6nbx.1    -------------------------------------------------------------------------------N  target    NLTTLTIISVLLAVLAVVTGFFGMNVPLPLTDEPHAWLYISLASAGLWIVLSLLLRKIAKKS 6nbx.1    AVRVLMSIELLLNAVNLNLIGFANYL------------------------------------ ``` | | | | | | | | | | | | | | | | | | | | | | | | | | | | | | | | | | | | | | | | | | | | | | | | | |
|  | 6nbq.1.L | NAD(P)H-quinone oxidoreductase subunit 4L  *T.elongatus NDH (data-set 1)* | 0.01 |  | 18.52 | 0.09 | 240-266 | EM | 0.00 | hetero-1-1-1-1-1-1-… | 3 x SF4 | HHblits | 0.27 |
| ``` target    MVLEKQLGNGCTWIDLDLGKLNKLEDLSEIYGLDKETIEYALDRNERAHMDYHRESETVTFIYNVLDVKKDKAYYETFPM 6nbq.1    --------------------------------------------------------------------------------  target    TFIVEHRRLITISNTKNAYVIEQMTRYLENHDTLSIYKFLFASLEIISNAYYPVIEQMDKSRDEVNDLLRQRTTKKNLFV 6nbq.1    --------------------------------------------------------------------------------  target    LSDLETGMVYLTAAAKQNRILLEHIQGHALYRSFDEIEREQFDDAMIEAHQLVSMTDLISQILQQLSASYNNILNNNLND 6nbq.1    -------------------------------------------------------------------------------N  target    NLTTLTIISVLLAVLAVVTGFFGMNVPLPLTDEPHAWLYISLASAGLWIVLSLLLRKIAKKS 6nbq.1    AVRVLMSIELLLNAVNLNLIGFANYL------------------------------------ ``` | | | | | | | | | | | | | | | | | | | | | | | | | | | | | | | | | | | | | | | | | | | | | | | | | |
|  | 6hum.1.D | NAD(P)H-quinone oxidoreductase subunit 4L  *Structure of the photosynthetic complex I from Thermosynechococcus elongatus* | 0.01 |  | 18.52 | 0.09 | 240-266 | EM | 0.00 | hetero-1-1-1-1-1-1-… | 1 x BCR, 1 x LMG, 3 x SF4 | HHblits | 0.27 |
| ``` target    MVLEKQLGNGCTWIDLDLGKLNKLEDLSEIYGLDKETIEYALDRNERAHMDYHRESETVTFIYNVLDVKKDKAYYETFPM 6hum.1    --------------------------------------------------------------------------------  target    TFIVEHRRLITISNTKNAYVIEQMTRYLENHDTLSIYKFLFASLEIISNAYYPVIEQMDKSRDEVNDLLRQRTTKKNLFV 6hum.1    --------------------------------------------------------------------------------  target    LSDLETGMVYLTAAAKQNRILLEHIQGHALYRSFDEIEREQFDDAMIEAHQLVSMTDLISQILQQLSASYNNILNNNLND 6hum.1    -------------------------------------------------------------------------------N  target    NLTTLTIISVLLAVLAVVTGFFGMNVPLPLTDEPHAWLYISLASAGLWIVLSLLLRKIAKKS 6hum.1    AVRVLMSIELLLNAVNLNLIGFANYL------------------------------------ ``` | | | | | | | | | | | | | | | | | | | | | | | | | | | | | | | | | | | | | | | | | | | | | | | | | |
|  | 6khi.1.E | NAD(P)H-quinone oxidoreductase subunit 4L  *Supercomplex for cylic electron transport in cyanobacteria* | 0.01 |  | 18.52 | 0.09 | 240-266 | EM | 0.00 | hetero-1-1-1-1-1-1-… | 2 x DGD, 6 x LHG, 3 x SQD, 2 x BCR, 2 x LMG, 3 x SF4, 1 x FES | HHblits | 0.27 |
| ``` target    MVLEKQLGNGCTWIDLDLGKLNKLEDLSEIYGLDKETIEYALDRNERAHMDYHRESETVTFIYNVLDVKKDKAYYETFPM 6khi.1    --------------------------------------------------------------------------------  target    TFIVEHRRLITISNTKNAYVIEQMTRYLENHDTLSIYKFLFASLEIISNAYYPVIEQMDKSRDEVNDLLRQRTTKKNLFV 6khi.1    --------------------------------------------------------------------------------  target    LSDLETGMVYLTAAAKQNRILLEHIQGHALYRSFDEIEREQFDDAMIEAHQLVSMTDLISQILQQLSASYNNILNNNLND 6khi.1    -------------------------------------------------------------------------------N  target    NLTTLTIISVLLAVLAVVTGFFGMNVPLPLTDEPHAWLYISLASAGLWIVLSLLLRKIAKKS 6khi.1    AVRVLMSIELLLNAVNLNLIGFANYL------------------------------------ ``` | | | | | | | | | | | | | | | | | | | | | | | | | | | | | | | | | | | | | | | | | | | | | | | | | |
|  | 6tjv.1.E | NAD(P)H-quinone oxidoreductase subunit 4L  *Structure of the NDH-1MS complex from Thermosynechococcus elongatus* | 0.01 |  | 18.52 | 0.09 | 240-266 | EM | 0.00 | hetero-1-1-1-1-1-1-… | 2 x DGD, 2 x PGT, 5 x SQD, 1 x BCR, 1 x CLA, 3 x SF4, 1 x ZN | HHblits | 0.27 |
| ``` target    MVLEKQLGNGCTWIDLDLGKLNKLEDLSEIYGLDKETIEYALDRNERAHMDYHRESETVTFIYNVLDVKKDKAYYETFPM 6tjv.1    --------------------------------------------------------------------------------  target    TFIVEHRRLITISNTKNAYVIEQMTRYLENHDTLSIYKFLFASLEIISNAYYPVIEQMDKSRDEVNDLLRQRTTKKNLFV 6tjv.1    --------------------------------------------------------------------------------  target    LSDLETGMVYLTAAAKQNRILLEHIQGHALYRSFDEIEREQFDDAMIEAHQLVSMTDLISQILQQLSASYNNILNNNLND 6tjv.1    -------------------------------------------------------------------------------N  target    NLTTLTIISVLLAVLAVVTGFFGMNVPLPLTDEPHAWLYISLASAGLWIVLSLLLRKIAKKS 6tjv.1    AVRVLMSIELLLNAVNLNLIGFANYL------------------------------------ ``` | | | | | | | | | | | | | | | | | | | | | | | | | | | | | | | | | | | | | | | | | | | | | | | | | |
|  | 6l7o.1.E | NAD(P)H-quinone oxidoreductase subunit 4L  *cryo-EM structure of cyanobacteria Fd-NDH-1L complex* | 0.01 |  | 18.52 | 0.09 | 240-266 | EM | 0.00 | hetero-1-1-1-1-1-1-… | 3 x BCR, 9 x LHG, 2 x DGD, 58 x E7U, 1 x PQN, 4 x SQD, 3 x SF4, 1 x FES | HHblits | 0.27 |
| ``` target    MVLEKQLGNGCTWIDLDLGKLNKLEDLSEIYGLDKETIEYALDRNERAHMDYHRESETVTFIYNVLDVKKDKAYYETFPM 6l7o.1    --------------------------------------------------------------------------------  target    TFIVEHRRLITISNTKNAYVIEQMTRYLENHDTLSIYKFLFASLEIISNAYYPVIEQMDKSRDEVNDLLRQRTTKKNLFV 6l7o.1    --------------------------------------------------------------------------------  target    LSDLETGMVYLTAAAKQNRILLEHIQGHALYRSFDEIEREQFDDAMIEAHQLVSMTDLISQILQQLSASYNNILNNNLND 6l7o.1    -------------------------------------------------------------------------------N  target    NLTTLTIISVLLAVLAVVTGFFGMNVPLPLTDEPHAWLYISLASAGLWIVLSLLLRKIAKKS 6l7o.1    AVRVLMSIELLLNAVNLNLIGFANYL------------------------------------ ``` | | | | | | | | | | | | | | | | | | | | | | | | | | | | | | | | | | | | | | | | | | | | | | | | | |
|  | 6eti.1.A | ATP-binding cassette sub-family G member 2  *Structure of inhibitor-bound ABCG2* | 0.00 |  | 3.70 | 0.09 | 236-262 | EM | 0.00 | hetero-2-2-2-mer | 2 x BWQ, 2 x NAG-NAG | HHblits | 0.27 |
| ``` target    MVLEKQLGNGCTWIDLDLGKLNKLEDLSEIYGLDKETIEYALDRNERAHMDYHRESETVTFIYNVLDVKKDKAYYETFPM 6eti.1    --------------------------------------------------------------------------------  target    TFIVEHRRLITISNTKNAYVIEQMTRYLENHDTLSIYKFLFASLEIISNAYYPVIEQMDKSRDEVNDLLRQRTTKKNLFV 6eti.1    --------------------------------------------------------------------------------  target    LSDLETGMVYLTAAAKQNRILLEHIQGHALYRSFDEIEREQFDDAMIEAHQLVSMTDLISQILQQLSASYNNILNNNLND 6eti.1    ---------------------------------------------------------------------------AGQSV  target    NLTTLTIISVLLAVLAVVTGFFGMNVPLPLTDEPHAWLYISLASAGLWIVLSLLLRKIAKKS 6eti.1    VSVATLLMTICFVFMMIFSGLL---------------------------------------- ``` | | | | | | | | | | | | | | | | | | | | | | | | | | | | | | | | | | | | | | | | | | | | | | | | | |
|  | 6hij.1.A | ATP-binding cassette sub-family G member 2  *Cryo-EM structure of the human ABCG2-MZ29-Fab complex with cholesterol and PE lipids docked* | 0.00 |  | 3.70 | 0.09 | 236-262 | EM | 0.00 | homo-dimer | 8 x PEE, 10 x CLR, 2 x BWQ | HHblits | 0.27 |
| ``` target    MVLEKQLGNGCTWIDLDLGKLNKLEDLSEIYGLDKETIEYALDRNERAHMDYHRESETVTFIYNVLDVKKDKAYYETFPM 6hij.1    --------------------------------------------------------------------------------  target    TFIVEHRRLITISNTKNAYVIEQMTRYLENHDTLSIYKFLFASLEIISNAYYPVIEQMDKSRDEVNDLLRQRTTKKNLFV 6hij.1    --------------------------------------------------------------------------------  target    LSDLETGMVYLTAAAKQNRILLEHIQGHALYRSFDEIEREQFDDAMIEAHQLVSMTDLISQILQQLSASYNNILNNNLND 6hij.1    ---------------------------------------------------------------------------AGQSV  target    NLTTLTIISVLLAVLAVVTGFFGMNVPLPLTDEPHAWLYISLASAGLWIVLSLLLRKIAKKS 6hij.1    VSVATLLMTICFVFMMIFSGLL---------------------------------------- ``` | | | | | | | | | | | | | | | | | | | | | | | | | | | | | | | | | | | | | | | | | | | | | | | | | |
|  | 6vxi.1.A | Broad substrate specificity ATP-binding cassette transporter ABCG2  *Structure of ABCG2 bound to mitoxantrone* | 0.00 |  | 3.70 | 0.09 | 236-262 | EM | 0.00 | homo-dimer | 2 x CLR, 1 x MIX | HHblits | 0.27 |
| ``` target    MVLEKQLGNGCTWIDLDLGKLNKLEDLSEIYGLDKETIEYALDRNERAHMDYHRESETVTFIYNVLDVKKDKAYYETFPM 6vxi.1    --------------------------------------------------------------------------------  target    TFIVEHRRLITISNTKNAYVIEQMTRYLENHDTLSIYKFLFASLEIISNAYYPVIEQMDKSRDEVNDLLRQRTTKKNLFV 6vxi.1    --------------------------------------------------------------------------------  target    LSDLETGMVYLTAAAKQNRILLEHIQGHALYRSFDEIEREQFDDAMIEAHQLVSMTDLISQILQQLSASYNNILNNNLND 6vxi.1    ---------------------------------------------------------------------------AGQSV  target    NLTTLTIISVLLAVLAVVTGFFGMNVPLPLTDEPHAWLYISLASAGLWIVLSLLLRKIAKKS 6vxi.1    VSVATLLMTICFVFMMIFSGLL---------------------------------------- ``` | | | | | | | | | | | | | | | | | | | | | | | | | | | | | | | | | | | | | | | | | | | | | | | | | |
|  | 6vxh.1.B | Broad substrate specificity ATP-binding cassette transporter ABCG2  *Structure of ABCG2 bound to imatinib* | 0.00 |  | 3.70 | 0.09 | 236-262 | EM | 0.00 | homo-dimer | 2 x CLR, 1 x STI | HHblits | 0.27 |
| ``` target    MVLEKQLGNGCTWIDLDLGKLNKLEDLSEIYGLDKETIEYALDRNERAHMDYHRESETVTFIYNVLDVKKDKAYYETFPM 6vxh.1    --------------------------------------------------------------------------------  target    TFIVEHRRLITISNTKNAYVIEQMTRYLENHDTLSIYKFLFASLEIISNAYYPVIEQMDKSRDEVNDLLRQRTTKKNLFV 6vxh.1    --------------------------------------------------------------------------------  target    LSDLETGMVYLTAAAKQNRILLEHIQGHALYRSFDEIEREQFDDAMIEAHQLVSMTDLISQILQQLSASYNNILNNNLND 6vxh.1    ---------------------------------------------------------------------------AGQSV  target    NLTTLTIISVLLAVLAVVTGFFGMNVPLPLTDEPHAWLYISLASAGLWIVLSLLLRKIAKKS 6vxh.1    VSVATLLMTICFVFMMIFSGLL---------------------------------------- ``` | | | | | | | | | | | | | | | | | | | | | | | | | | | | | | | | | | | | | | | | | | | | | | | | | |
|  | 6vxf.1.B | Broad substrate specificity ATP-binding cassette transporter ABCG2  *Structure of apo-closed ABCG2* | 0.00 |  | 3.70 | 0.09 | 236-262 | EM | 0.00 | homo-dimer |  | HHblits | 0.27 |
| ``` target    MVLEKQLGNGCTWIDLDLGKLNKLEDLSEIYGLDKETIEYALDRNERAHMDYHRESETVTFIYNVLDVKKDKAYYETFPM 6vxf.1    --------------------------------------------------------------------------------  target    TFIVEHRRLITISNTKNAYVIEQMTRYLENHDTLSIYKFLFASLEIISNAYYPVIEQMDKSRDEVNDLLRQRTTKKNLFV 6vxf.1    --------------------------------------------------------------------------------  target    LSDLETGMVYLTAAAKQNRILLEHIQGHALYRSFDEIEREQFDDAMIEAHQLVSMTDLISQILQQLSASYNNILNNNLND 6vxf.1    ---------------------------------------------------------------------------AGQSV  target    NLTTLTIISVLLAVLAVVTGFFGMNVPLPLTDEPHAWLYISLASAGLWIVLSLLLRKIAKKS 6vxf.1    VSVATLLMTICFVFMMIFSGLL---------------------------------------- ``` | | | | | | | | | | | | | | | | | | | | | | | | | | | | | | | | | | | | | | | | | | | | | | | | | |
|  | 7nez.1.A | ATP-binding cassette sub-family G member 2  *Structure of topotecan-bound ABCG2* | 0.00 |  | 3.70 | 0.09 | 236-262 | EM | 0.00 | hetero-2-2-2-mer | 1 x TTC, 2 x NAG | HHblits | 0.27 |
| ``` target    MVLEKQLGNGCTWIDLDLGKLNKLEDLSEIYGLDKETIEYALDRNERAHMDYHRESETVTFIYNVLDVKKDKAYYETFPM 7nez.1    --------------------------------------------------------------------------------  target    TFIVEHRRLITISNTKNAYVIEQMTRYLENHDTLSIYKFLFASLEIISNAYYPVIEQMDKSRDEVNDLLRQRTTKKNLFV 7nez.1    --------------------------------------------------------------------------------  target    LSDLETGMVYLTAAAKQNRILLEHIQGHALYRSFDEIEREQFDDAMIEAHQLVSMTDLISQILQQLSASYNNILNNNLND 7nez.1    ---------------------------------------------------------------------------AGQSV  target    NLTTLTIISVLLAVLAVVTGFFGMNVPLPLTDEPHAWLYISLASAGLWIVLSLLLRKIAKKS 7nez.1    VSVATLLMTICFVFMMIFSGLL---------------------------------------- ``` | | | | | | | | | | | | | | | | | | | | | | | | | | | | | | | | | | | | | | | | | | | | | | | | | |
|  | 7nfd.1.F | ATP-binding cassette sub-family G member 2  *Structure of mitoxantrone-bound ABCG2* | 0.00 |  | 3.70 | 0.09 | 236-262 | EM | 0.00 | hetero-2-2-2-mer | 1 x MIX, 2 x NAG-NAG | HHblits | 0.27 |
| ``` target    MVLEKQLGNGCTWIDLDLGKLNKLEDLSEIYGLDKETIEYALDRNERAHMDYHRESETVTFIYNVLDVKKDKAYYETFPM 7nfd.1    --------------------------------------------------------------------------------  target    TFIVEHRRLITISNTKNAYVIEQMTRYLENHDTLSIYKFLFASLEIISNAYYPVIEQMDKSRDEVNDLLRQRTTKKNLFV 7nfd.1    --------------------------------------------------------------------------------  target    LSDLETGMVYLTAAAKQNRILLEHIQGHALYRSFDEIEREQFDDAMIEAHQLVSMTDLISQILQQLSASYNNILNNNLND 7nfd.1    ---------------------------------------------------------------------------AGQSV  target    NLTTLTIISVLLAVLAVVTGFFGMNVPLPLTDEPHAWLYISLASAGLWIVLSLLLRKIAKKS 7nfd.1    VSVATLLMTICFVFMMIFSGLL---------------------------------------- ``` | | | | | | | | | | | | | | | | | | | | | | | | | | | | | | | | | | | | | | | | | | | | | | | | | |
|  | 7neq.1.F | ATP-binding cassette sub-family G member 2  *Structure of tariquidar-bound ABCG2* | 0.00 |  | 3.70 | 0.09 | 236-262 | EM | 0.00 | hetero-2-2-2-mer | 2 x NAG, 1 x U9N, 3 x CLR, 1 x R1H | HHblits | 0.27 |
| ``` target    MVLEKQLGNGCTWIDLDLGKLNKLEDLSEIYGLDKETIEYALDRNERAHMDYHRESETVTFIYNVLDVKKDKAYYETFPM 7neq.1    --------------------------------------------------------------------------------  target    TFIVEHRRLITISNTKNAYVIEQMTRYLENHDTLSIYKFLFASLEIISNAYYPVIEQMDKSRDEVNDLLRQRTTKKNLFV 7neq.1    --------------------------------------------------------------------------------  target    LSDLETGMVYLTAAAKQNRILLEHIQGHALYRSFDEIEREQFDDAMIEAHQLVSMTDLISQILQQLSASYNNILNNNLND 7neq.1    ---------------------------------------------------------------------------AGQSV  target    NLTTLTIISVLLAVLAVVTGFFGMNVPLPLTDEPHAWLYISLASAGLWIVLSLLLRKIAKKS 7neq.1    VSVATLLMTICFVFMMIFSGLL---------------------------------------- ``` | | | | | | | | | | | | | | | | | | | | | | | | | | | | | | | | | | | | | | | | | | | | | | | | | |
|  | 7neq.1.A | ATP-binding cassette sub-family G member 2  *Structure of tariquidar-bound ABCG2* | 0.00 |  | 3.70 | 0.09 | 236-262 | EM | 0.00 | hetero-2-2-2-mer | 2 x NAG, 1 x U9N, 3 x CLR, 1 x R1H | HHblits | 0.27 |
| ``` target    MVLEKQLGNGCTWIDLDLGKLNKLEDLSEIYGLDKETIEYALDRNERAHMDYHRESETVTFIYNVLDVKKDKAYYETFPM 7neq.1    --------------------------------------------------------------------------------  target    TFIVEHRRLITISNTKNAYVIEQMTRYLENHDTLSIYKFLFASLEIISNAYYPVIEQMDKSRDEVNDLLRQRTTKKNLFV 7neq.1    --------------------------------------------------------------------------------  target    LSDLETGMVYLTAAAKQNRILLEHIQGHALYRSFDEIEREQFDDAMIEAHQLVSMTDLISQILQQLSASYNNILNNNLND 7neq.1    ---------------------------------------------------------------------------AGQSV  target    NLTTLTIISVLLAVLAVVTGFFGMNVPLPLTDEPHAWLYISLASAGLWIVLSLLLRKIAKKS 7neq.1    VSVATLLMTICFVFMMIFSGLL---------------------------------------- ``` | | | | | | | | | | | | | | | | | | | | | | | | | | | | | | | | | | | | | | | | | | | | | | | | | |
|  | 6rlb.1.A | O6-alkylguanine-DNA alkyltransferase mutant,DYNC2H1 variant protein  *Structure of the dynein-2 complex; tail domain* | 0.02 |  | 11.54 | 0.09 | 124-149 | EM | 0.00 | hetero-2-1-1-2-2-6-… |  | HHblits | 0.28 |
| ``` target    MVLEKQLGNGCTWIDLDLGKLNKLEDLSEIYGLDKETIEYALDRNERAHMDYHRESETVTFIYNVLDVKKDKAYYETFPM 6rlb.1    --------------------------------------------------------------------------------  target    TFIVEHRRLITISNTKNAYVIEQMTRYLENHDTLSIYKFLFASLEIISNAYYPVIEQMDKSRDEVNDLLRQRTTKKNLFV 6rlb.1    -------------------------------------------VLSLKKSIQAHLHEIDTFVTEAMEVL-----------  target    LSDLETGMVYLTAAAKQNRILLEHIQGHALYRSFDEIEREQFDDAMIEAHQLVSMTDLISQILQQLSASYNNILNNNLND 6rlb.1    --------------------------------------------------------------------------------  target    NLTTLTIISVLLAVLAVVTGFFGMNVPLPLTDEPHAWLYISLASAGLWIVLSLLLRKIAKKS 6rlb.1    -------------------------------------------------------------- ``` | | | | | | | | | | | | | | | | | | | | | | | | | | | | | | | | | | | | | | | | | | | | | | | | | |
|  | 2c5i.1.B | T-SNARE AFFECTING A LATE GOLGI COMPARTMENT PROTEIN 1  *N-TERMINAL DOMAIN OF TLG1 COMPLEXED WITH N-TERMINUS OF VPS51 IN DISTORTED CONFORMATION* | 0.01 |  | 0.00 | 0.09 | 125-151 | X-ray | 2.30 | hetero-oligomer |  | HHblits | 0.25 |
| ``` target    MVLEKQLGNGCTWIDLDLGKLNKLEDLSEIYGLDKETIEYALDRNERAHMDYHRESETVTFIYNVLDVKKDKAYYETFPM 2c5i.1    --------------------------------------------------------------------------------  target    TFIVEHRRLITISNTKNAYVIEQMTRYLENHDTLSIYKFLFASLEIISNAYYPVIEQMDKSRDEVNDLLRQRTTKKNLFV 2c5i.1    --------------------------------------------DDQEEEIQDILKDVEETIVDLDRSIIV---------  target    LSDLETGMVYLTAAAKQNRILLEHIQGHALYRSFDEIEREQFDDAMIEAHQLVSMTDLISQILQQLSASYNNILNNNLND 2c5i.1    --------------------------------------------------------------------------------  target    NLTTLTIISVLLAVLAVVTGFFGMNVPLPLTDEPHAWLYISLASAGLWIVLSLLLRKIAKKS 2c5i.1    -------------------------------------------------------------- ``` | | | | | | | | | | | | | | | | | | | | | | | | | | | | | | | | | | | | | | | | | | | | | | | | | |
|  | 6tfj.1.B | Vegetative insecticidal protein  *Vip3Aa protoxin structure* | 0.00 |  | 24.00 | 0.08 | 124-149 | EM | 0.00 | homo-tetramer |  | HHblits | 0.31 |
| ``` target    MVLEKQLGNGCTWIDLDLGKLNKLEDLSEIYGLDKETIEYALDRNERAHMDYHRESETVTFIYNVLDVKKDKAYYETFPM 6tfj.1    --------------------------------------------------------------------------------  target    TFIVEHRRLITISNTKNAYVIEQMTRYLENHDTLSIYKFLFASLEIISNAYYPVIEQMDKSRDEVNDLLRQRTTKKNLFV 6tfj.1    -------------------------------------------LDEILKNQQ-LLNDISGKLDGVNGSL-----------  target    LSDLETGMVYLTAAAKQNRILLEHIQGHALYRSFDEIEREQFDDAMIEAHQLVSMTDLISQILQQLSASYNNILNNNLND 6tfj.1    --------------------------------------------------------------------------------  target    NLTTLTIISVLLAVLAVVTGFFGMNVPLPLTDEPHAWLYISLASAGLWIVLSLLLRKIAKKS 6tfj.1    -------------------------------------------------------------- ``` | | | | | | | | | | | | | | | | | | | | | | | | | | | | | | | | | | | | | | | | | | | | | | | | | |
|  | 6tfj.1.A | Vegetative insecticidal protein  *Vip3Aa protoxin structure* | 0.02 |  | 24.00 | 0.08 | 124-149 | EM | 0.00 | homo-tetramer |  | HHblits | 0.31 |
| ``` target    MVLEKQLGNGCTWIDLDLGKLNKLEDLSEIYGLDKETIEYALDRNERAHMDYHRESETVTFIYNVLDVKKDKAYYETFPM 6tfj.1    --------------------------------------------------------------------------------  target    TFIVEHRRLITISNTKNAYVIEQMTRYLENHDTLSIYKFLFASLEIISNAYYPVIEQMDKSRDEVNDLLRQRTTKKNLFV 6tfj.1    -------------------------------------------LDEILKNQQ-LLNDISGKLDGVNGSL-----------  target    LSDLETGMVYLTAAAKQNRILLEHIQGHALYRSFDEIEREQFDDAMIEAHQLVSMTDLISQILQQLSASYNNILNNNLND 6tfj.1    --------------------------------------------------------------------------------  target    NLTTLTIISVLLAVLAVVTGFFGMNVPLPLTDEPHAWLYISLASAGLWIVLSLLLRKIAKKS 6tfj.1    -------------------------------------------------------------- ``` | | | | | | | | | | | | | | | | | | | | | | | | | | | | | | | | | | | | | | | | | | | | | | | | | |
|  | 6yrf.1.B | Vegetative insecticidal protein  *Vip3Bc1 tetramer* | 0.00 |  | 20.00 | 0.08 | 124-149 | EM | 0.00 | homo-tetramer |  | HHblits | 0.29 |
| ``` target    MVLEKQLGNGCTWIDLDLGKLNKLEDLSEIYGLDKETIEYALDRNERAHMDYHRESETVTFIYNVLDVKKDKAYYETFPM 6yrf.1    --------------------------------------------------------------------------------  target    TFIVEHRRLITISNTKNAYVIEQMTRYLENHDTLSIYKFLFASLEIISNAYYPVIEQMDKSRDEVNDLLRQRTTKKNLFV 6yrf.1    -------------------------------------------LDEILKNQNL-LNDISGKLDGINGDL-----------  target    LSDLETGMVYLTAAAKQNRILLEHIQGHALYRSFDEIEREQFDDAMIEAHQLVSMTDLISQILQQLSASYNNILNNNLND 6yrf.1    --------------------------------------------------------------------------------  target    NLTTLTIISVLLAVLAVVTGFFGMNVPLPLTDEPHAWLYISLASAGLWIVLSLLLRKIAKKS 6yrf.1    -------------------------------------------------------------- ``` | | | | | | | | | | | | | | | | | | | | | | | | | | | | | | | | | | | | | | | | | | | | | | | | | |
|  | 6yrf.1.A | Vegetative insecticidal protein  *Vip3Bc1 tetramer* | 0.01 |  | 20.00 | 0.08 | 124-149 | EM | 0.00 | homo-tetramer |  | HHblits | 0.29 |
| ``` target    MVLEKQLGNGCTWIDLDLGKLNKLEDLSEIYGLDKETIEYALDRNERAHMDYHRESETVTFIYNVLDVKKDKAYYETFPM 6yrf.1    --------------------------------------------------------------------------------  target    TFIVEHRRLITISNTKNAYVIEQMTRYLENHDTLSIYKFLFASLEIISNAYYPVIEQMDKSRDEVNDLLRQRTTKKNLFV 6yrf.1    -------------------------------------------LDEILKNQNL-LNDISGKLDGINGDL-----------  target    LSDLETGMVYLTAAAKQNRILLEHIQGHALYRSFDEIEREQFDDAMIEAHQLVSMTDLISQILQQLSASYNNILNNNLND 6yrf.1    --------------------------------------------------------------------------------  target    NLTTLTIISVLLAVLAVVTGFFGMNVPLPLTDEPHAWLYISLASAGLWIVLSLLLRKIAKKS 6yrf.1    -------------------------------------------------------------- ``` | | | | | | | | | | | | | | | | | | | | | | | | | | | | | | | | | | | | | | | | | | | | | | | | | |
|  | 1orj.1.A | flagellar protein FliS  *FLAGELLAR EXPORT CHAPERONE* | 0.01 |  | 11.54 | 0.09 | 114-139 | X-ray | 2.25 | monomer |  | HHblits | 0.25 |
| ``` target    MVLEKQLGNGCTWIDLDLGKLNKLEDLSEIYGLDKETIEYALDRNERAHMDYHRESETVTFIYNVLDVKKDKAYYETFPM 1orj.1    --------------------------------------------------------------------------------  target    TFIVEHRRLITISNTKNAYVIEQMTRYLENHDTLSIYKFLFASLEIISNAYYPVIEQMDKSRDEVNDLLRQRTTKKNLFV 1orj.1    ---------------------------------ATPLEQIILLYDKAIECLERAIEIYD---------------------  target    LSDLETGMVYLTAAAKQNRILLEHIQGHALYRSFDEIEREQFDDAMIEAHQLVSMTDLISQILQQLSASYNNILNNNLND 1orj.1    --------------------------------------------------------------------------------  target    NLTTLTIISVLLAVLAVVTGFFGMNVPLPLTDEPHAWLYISLASAGLWIVLSLLLRKIAKKS 1orj.1    -------------------------------------------------------------- ``` | | | | | | | | | | | | | | | | | | | | | | | | | | | | | | | | | | | | | | | | | | | | | | | | | |
|  | 1orj.2.A | flagellar protein FliS  *FLAGELLAR EXPORT CHAPERONE* | 0.01 |  | 11.54 | 0.09 | 114-139 | X-ray | 2.25 | monomer |  | HHblits | 0.25 |
| ``` target    MVLEKQLGNGCTWIDLDLGKLNKLEDLSEIYGLDKETIEYALDRNERAHMDYHRESETVTFIYNVLDVKKDKAYYETFPM 1orj.2    --------------------------------------------------------------------------------  target    TFIVEHRRLITISNTKNAYVIEQMTRYLENHDTLSIYKFLFASLEIISNAYYPVIEQMDKSRDEVNDLLRQRTTKKNLFV 1orj.2    ---------------------------------ATPLEQIILLYDKAIECLERAIEIYD---------------------  target    LSDLETGMVYLTAAAKQNRILLEHIQGHALYRSFDEIEREQFDDAMIEAHQLVSMTDLISQILQQLSASYNNILNNNLND 1orj.2    --------------------------------------------------------------------------------  target    NLTTLTIISVLLAVLAVVTGFFGMNVPLPLTDEPHAWLYISLASAGLWIVLSLLLRKIAKKS 1orj.2    -------------------------------------------------------------- ``` | | | | | | | | | | | | | | | | | | | | | | | | | | | | | | | | | | | | | | | | | | | | | | | | | |
|  | 6hbu.1.A | ATP-binding cassette sub-family G member 2  *Cryo-EM structure of the ABCG2 E211Q mutant bound to ATP and Magnesium* | 0.00 |  | 4.00 | 0.08 | 238-262 | EM | 0.00 | homo-dimer | 2 x ATP, 2 x MG | HHblits | 0.28 |
| ``` target    MVLEKQLGNGCTWIDLDLGKLNKLEDLSEIYGLDKETIEYALDRNERAHMDYHRESETVTFIYNVLDVKKDKAYYETFPM 6hbu.1    --------------------------------------------------------------------------------  target    TFIVEHRRLITISNTKNAYVIEQMTRYLENHDTLSIYKFLFASLEIISNAYYPVIEQMDKSRDEVNDLLRQRTTKKNLFV 6hbu.1    --------------------------------------------------------------------------------  target    LSDLETGMVYLTAAAKQNRILLEHIQGHALYRSFDEIEREQFDDAMIEAHQLVSMTDLISQILQQLSASYNNILNNNLND 6hbu.1    -----------------------------------------------------------------------------QSV  target    NLTTLTIISVLLAVLAVVTGFFGMNVPLPLTDEPHAWLYISLASAGLWIVLSLLLRKIAKKS 6hbu.1    VSVATLLMTICFVFMMIFSGLL---------------------------------------- ``` | | | | | | | | | | | | | | | | | | | | | | | | | | | | | | | | | | | | | | | | | | | | | | | | | |
|  | 6w4s.1.A | Solute carrier family 40 member 1  *Structure of apo human ferroportin in lipid nanodisc* | 0.01 |  | 11.54 | 0.09 | 236-261 | EM | 0.00 | hetero-1-1-1-mer |  | HHblits | 0.25 |
| ``` target    MVLEKQLGNGCTWIDLDLGKLNKLEDLSEIYGLDKETIEYALDRNERAHMDYHRESETVTFIYNVLDVKKDKAYYETFPM 6w4s.1    --------------------------------------------------------------------------------  target    TFIVEHRRLITISNTKNAYVIEQMTRYLENHDTLSIYKFLFASLEIISNAYYPVIEQMDKSRDEVNDLLRQRTTKKNLFV 6w4s.1    --------------------------------------------------------------------------------  target    LSDLETGMVYLTAAAKQNRILLEHIQGHALYRSFDEIEREQFDDAMIEAHQLVSMTDLISQILQQLSASYNNILNNNLND 6w4s.1    ---------------------------------------------------------------------------SERGI  target    NLTTLTIISVLLAVLAVVTGFFGMNVPLPLTDEPHAWLYISLASAGLWIVLSLLLRKIAKKS 6w4s.1    INGVQNSMNYLLDLLHFIMVI----------------------------------------- ``` | | | | | | | | | | | | | | | | | | | | | | | | | | | | | | | | | | | | | | | | | | | | | | | | | |
|  | 6wbv.1.B | Solute carrier family 40 member 1  *Structure of human ferroportin bound to hepcidin and cobalt in lipid nanodisc* | 0.01 |  | 11.54 | 0.09 | 236-261 | EM | 0.00 | hetero-1-1-1-1-mer | 2 x AGA, 2 x CO | HHblits | 0.25 |
| ``` target    MVLEKQLGNGCTWIDLDLGKLNKLEDLSEIYGLDKETIEYALDRNERAHMDYHRESETVTFIYNVLDVKKDKAYYETFPM 6wbv.1    --------------------------------------------------------------------------------  target    TFIVEHRRLITISNTKNAYVIEQMTRYLENHDTLSIYKFLFASLEIISNAYYPVIEQMDKSRDEVNDLLRQRTTKKNLFV 6wbv.1    --------------------------------------------------------------------------------  target    LSDLETGMVYLTAAAKQNRILLEHIQGHALYRSFDEIEREQFDDAMIEAHQLVSMTDLISQILQQLSASYNNILNNNLND 6wbv.1    ---------------------------------------------------------------------------SERGI  target    NLTTLTIISVLLAVLAVVTGFFGMNVPLPLTDEPHAWLYISLASAGLWIVLSLLLRKIAKKS 6wbv.1    INGVQNSMNYLLDLLHFIMVI----------------------------------------- ``` | | | | | | | | | | | | | | | | | | | | | | | | | | | | | | | | | | | | | | | | | | | | | | | | | |
|  | 6vyh.1.A | Solute carrier family 40 protein  *Cryo-EM structure of SLC40/ferroportin in complex with Fab* | 0.01 |  | 11.54 | 0.09 | 236-261 | EM | 0.00 | hetero-1-1-1-mer | 2 x CO | HHblits | 0.25 |
| ``` target    MVLEKQLGNGCTWIDLDLGKLNKLEDLSEIYGLDKETIEYALDRNERAHMDYHRESETVTFIYNVLDVKKDKAYYETFPM 6vyh.1    --------------------------------------------------------------------------------  target    TFIVEHRRLITISNTKNAYVIEQMTRYLENHDTLSIYKFLFASLEIISNAYYPVIEQMDKSRDEVNDLLRQRTTKKNLFV 6vyh.1    --------------------------------------------------------------------------------  target    LSDLETGMVYLTAAAKQNRILLEHIQGHALYRSFDEIEREQFDDAMIEAHQLVSMTDLISQILQQLSASYNNILNNNLND 6vyh.1    ---------------------------------------------------------------------------SERGI  target    NLTTLTIISVLLAVLAVVTGFFGMNVPLPLTDEPHAWLYISLASAGLWIVLSLLLRKIAKKS 6vyh.1    INGVQNSMNYLLDLLHFIMVI----------------------------------------- ``` | | | | | | | | | | | | | | | | | | | | | | | | | | | | | | | | | | | | | | | | | | | | | | | | | |
|  | 6wik.1.C | Solute carrier family 40 protein  *Cryo-EM structure of SLC40/ferroportin with Fab in the presence of hepcidin* | 0.01 |  | 11.54 | 0.09 | 236-261 | EM | 0.00 | hetero-1-1-1-mer |  | HHblits | 0.25 |
| ``` target    MVLEKQLGNGCTWIDLDLGKLNKLEDLSEIYGLDKETIEYALDRNERAHMDYHRESETVTFIYNVLDVKKDKAYYETFPM 6wik.1    --------------------------------------------------------------------------------  target    TFIVEHRRLITISNTKNAYVIEQMTRYLENHDTLSIYKFLFASLEIISNAYYPVIEQMDKSRDEVNDLLRQRTTKKNLFV 6wik.1    --------------------------------------------------------------------------------  target    LSDLETGMVYLTAAAKQNRILLEHIQGHALYRSFDEIEREQFDDAMIEAHQLVSMTDLISQILQQLSASYNNILNNNLND 6wik.1    ---------------------------------------------------------------------------SERGI  target    NLTTLTIISVLLAVLAVVTGFFGMNVPLPLTDEPHAWLYISLASAGLWIVLSLLLRKIAKKS 6wik.1    INGVQNSMNYLLDLLHFIMVI----------------------------------------- ``` | | | | | | | | | | | | | | | | | | | | | | | | | | | | | | | | | | | | | | | | | | | | | | | | | |
|  | 5sva.1.U | Mediator of RNA polymerase II transcription subunit 7  *Mediator-RNA Polymerase II Pre-Initiation Complex* | 0.00 |  | 20.00 | 0.08 | 119-143 | EM | 0.00 | hetero-1-1-1-1-1-1-… | 8 x ZN, 1 x MG | HHblits | 0.27 |
| ``` target    MVLEKQLGNGCTWIDLDLGKLNKLEDLSEIYGLDKETIEYALDRNERAHMDYHRESETVTFIYNVLDVKKDKAYYETFPM 5sva.1    --------------------------------------------------------------------------------  target    TFIVEHRRLITISNTKNAYVIEQMTRYLENHDTLSIYKFLFASLEIISNAYYPVIEQMDKSRDEVNDLLRQRTTKKNLFV 5sva.1    --------------------------------------ELRKLLKSLLLNYLELIGVLSINPD-----------------  target    LSDLETGMVYLTAAAKQNRILLEHIQGHALYRSFDEIEREQFDDAMIEAHQLVSMTDLISQILQQLSASYNNILNNNLND 5sva.1    --------------------------------------------------------------------------------  target    NLTTLTIISVLLAVLAVVTGFFGMNVPLPLTDEPHAWLYISLASAGLWIVLSLLLRKIAKKS 5sva.1    -------------------------------------------------------------- ``` | | | | | | | | | | | | | | | | | | | | | | | | | | | | | | | | | | | | | | | | | | | | | | | | | |
|  | 5do7.1.A | ATP-binding cassette sub-family G member 5  *Crystal Structure of the Human Sterol Transporter ABCG5/ABCG8* | 0.00 |  | 16.67 | 0.08 | 238-261 | X-ray | 3.93 | hetero-1-1-mer |  | HHblits | 0.31 |
| ``` target    MVLEKQLGNGCTWIDLDLGKLNKLEDLSEIYGLDKETIEYALDRNERAHMDYHRESETVTFIYNVLDVKKDKAYYETFPM 5do7.1    --------------------------------------------------------------------------------  target    TFIVEHRRLITISNTKNAYVIEQMTRYLENHDTLSIYKFLFASLEIISNAYYPVIEQMDKSRDEVNDLLRQRTTKKNLFV 5do7.1    --------------------------------------------------------------------------------  target    LSDLETGMVYLTAAAKQNRILLEHIQGHALYRSFDEIEREQFDDAMIEAHQLVSMTDLISQILQQLSASYNNILNNNLND 5do7.1    -----------------------------------------------------------------------------VQN  target    NLTTLTIISVLLAVLAVVT-GFFGMNVPLPLTDEPHAWLYISLASAGLWIVLSLLLRKIAKKS 5do7.1    PNIVNSVVALLSIAGVLVGSGF----------------------------------------- ``` | | | | | | | | | | | | | | | | | | | | | | | | | | | | | | | | | | | | | | | | | | | | | | | | | |
|  | 5do7.2.B | ATP-binding cassette sub-family G member 5  *Crystal Structure of the Human Sterol Transporter ABCG5/ABCG8* | 0.00 |  | 16.67 | 0.08 | 238-261 | X-ray | 3.93 | hetero-1-1-mer |  | HHblits | 0.31 |
| ``` target    MVLEKQLGNGCTWIDLDLGKLNKLEDLSEIYGLDKETIEYALDRNERAHMDYHRESETVTFIYNVLDVKKDKAYYETFPM 5do7.2    --------------------------------------------------------------------------------  target    TFIVEHRRLITISNTKNAYVIEQMTRYLENHDTLSIYKFLFASLEIISNAYYPVIEQMDKSRDEVNDLLRQRTTKKNLFV 5do7.2    --------------------------------------------------------------------------------  target    LSDLETGMVYLTAAAKQNRILLEHIQGHALYRSFDEIEREQFDDAMIEAHQLVSMTDLISQILQQLSASYNNILNNNLND 5do7.2    -----------------------------------------------------------------------------VQN  target    NLTTLTIISVLLAVLAVVT-GFFGMNVPLPLTDEPHAWLYISLASAGLWIVLSLLLRKIAKKS 5do7.2    PNIVNSVVALLSIAGVLVGSGF----------------------------------------- ``` | | | | | | | | | | | | | | | | | | | | | | | | | | | | | | | | | | | | | | | | | | | | | | | | | |
|  | 3tlm.1.A | Sarcoplasmic/endoplasmic reticulum calcium ATPase 1  *Crystal Structure of Endoplasmic Reticulum Ca2+-ATPase (SERCA) From Bovine Muscle* | 0.01 |  | 20.00 | 0.08 | 277-301 | X-ray | 2.95 | monomer | 2 x CA, 1 x MG, 1 x K, 1 x ACP | HHblits | 0.27 |
| ``` target    MVLEKQLGNGCTWIDLDLGKLNKLEDLSEIYGLDKETIEYALDRNERAHMDYHRESETVTFIYNVLDVKKDKAYYETFPM 3tlm.1    --------------------------------------------------------------------------------  target    TFIVEHRRLITISNTKNAYVIEQMTRYLENHDTLSIYKFLFASLEIISNAYYPVIEQMDKSRDEVNDLLRQRTTKKNLFV 3tlm.1    --------------------------------------------------------------------------------  target    LSDLETGMVYLTAAAKQNRILLEHIQGHALYRSFDEIEREQFDDAMIEAHQLVSMTDLISQILQQLSASYNNILNNNLND 3tlm.1    --------------------------------------------------------------------------------  target    NLTTLTIISVLLAVLAVVTGFFGMNVPLPLTDEPHAWLYISLASAGLWIVLSLLLRKIAKKS 3tlm.1    ------------------------------------HWLMVLKISLPVIGLDEILKFVARN- ``` | | | | | | | | | | | | | | | | | | | | | | | | | | | | | | | | | | | | | | | | | | | | | | | | | |
|  | 6gy8.1.A | XaxA  *Crystal structure of XaxA from Xenorhabdus nematophila* | 0.01 |  | 26.09 | 0.08 | 210-232 | X-ray | 2.50 | monomer |  | HHblits | 0.33 |
| ``` target    MVLEKQLGNGCTWIDLDLGKLNKLEDLSEIYGLDKETIEYALDRNERAHMDYHRESETVTFIYNVLDVKKDKAYYETFPM 6gy8.1    --------------------------------------------------------------------------------  target    TFIVEHRRLITISNTKNAYVIEQMTRYLENHDTLSIYKFLFASLEIISNAYYPVIEQMDKSRDEVNDLLRQRTTKKNLFV 6gy8.1    --------------------------------------------------------------------------------  target    LSDLETGMVYLTAAAKQNRILLEHIQGHALYRSFDEIEREQFDDAMIEAHQLVSMTDLISQILQQLSASYNNILNNNLND 6gy8.1    -------------------------------------------------KDLQSKIDEKNKEIDQFQKDYNK--------  target    NLTTLTIISVLLAVLAVVTGFFGMNVPLPLTDEPHAWLYISLASAGLWIVLSLLLRKIAKKS 6gy8.1    -------------------------------------------------------------- ``` | | | | | | | | | | | | | | | | | | | | | | | | | | | | | | | | | | | | | | | | | | | | | | | | | |
|  | 6gy8.2.A | XaxA  *Crystal structure of XaxA from Xenorhabdus nematophila* | 0.01 |  | 26.09 | 0.08 | 210-232 | X-ray | 2.50 | monomer |  | HHblits | 0.33 |
| ``` target    MVLEKQLGNGCTWIDLDLGKLNKLEDLSEIYGLDKETIEYALDRNERAHMDYHRESETVTFIYNVLDVKKDKAYYETFPM 6gy8.2    --------------------------------------------------------------------------------  target    TFIVEHRRLITISNTKNAYVIEQMTRYLENHDTLSIYKFLFASLEIISNAYYPVIEQMDKSRDEVNDLLRQRTTKKNLFV 6gy8.2    --------------------------------------------------------------------------------  target    LSDLETGMVYLTAAAKQNRILLEHIQGHALYRSFDEIEREQFDDAMIEAHQLVSMTDLISQILQQLSASYNNILNNNLND 6gy8.2    -------------------------------------------------KDLQSKIDEKNKEIDQFQKDYNK--------  target    NLTTLTIISVLLAVLAVVTGFFGMNVPLPLTDEPHAWLYISLASAGLWIVLSLLLRKIAKKS 6gy8.2    -------------------------------------------------------------- ``` | | | | | | | | | | | | | | | | | | | | | | | | | | | | | | | | | | | | | | | | | | | | | | | | | |
|  | 6gy6.1.A | XaxA  *XaxAB pore complex from Xenorhabdus nematophila* | 0.01 |  | 26.09 | 0.08 | 210-232 | EM | 0.00 | hetero-13-13-mer |  | HHblits | 0.33 |
| ``` target    MVLEKQLGNGCTWIDLDLGKLNKLEDLSEIYGLDKETIEYALDRNERAHMDYHRESETVTFIYNVLDVKKDKAYYETFPM 6gy6.1    --------------------------------------------------------------------------------  target    TFIVEHRRLITISNTKNAYVIEQMTRYLENHDTLSIYKFLFASLEIISNAYYPVIEQMDKSRDEVNDLLRQRTTKKNLFV 6gy6.1    --------------------------------------------------------------------------------  target    LSDLETGMVYLTAAAKQNRILLEHIQGHALYRSFDEIEREQFDDAMIEAHQLVSMTDLISQILQQLSASYNNILNNNLND 6gy6.1    -------------------------------------------------KDLQSKIDEKNKEIDQFQKDYNK--------  target    NLTTLTIISVLLAVLAVVTGFFGMNVPLPLTDEPHAWLYISLASAGLWIVLSLLLRKIAKKS 6gy6.1    -------------------------------------------------------------- ``` | | | | | | | | | | | | | | | | | | | | | | | | | | | | | | | | | | | | | | | | | | | | | | | | | |
|  | 4ycm.1.A | Sarcoplasmic/endoplasmic reticulum calcium ATPase 1  *Crystal structure of the calcium pump with bound marine macrolide BLS* | 0.01 |  | 25.00 | 0.08 | 277-300 | X-ray | 3.20 | monomer | 1 x 7BS, 2 x PTY | HHblits | 0.28 |
| ``` target    MVLEKQLGNGCTWIDLDLGKLNKLEDLSEIYGLDKETIEYALDRNERAHMDYHRESETVTFIYNVLDVKKDKAYYETFPM 4ycm.1    --------------------------------------------------------------------------------  target    TFIVEHRRLITISNTKNAYVIEQMTRYLENHDTLSIYKFLFASLEIISNAYYPVIEQMDKSRDEVNDLLRQRTTKKNLFV 4ycm.1    --------------------------------------------------------------------------------  target    LSDLETGMVYLTAAAKQNRILLEHIQGHALYRSFDEIEREQFDDAMIEAHQLVSMTDLISQILQQLSASYNNILNNNLND 4ycm.1    --------------------------------------------------------------------------------  target    NLTTLTIISVLLAVLAVVTGFFGMNVPLPLTDEPHAWLYISLASAGLWIVLSLLLRKIAKKS 4ycm.1    ------------------------------------QWLMVLKISLPVIGLDEILKFIAR-- ``` | | | | | | | | | | | | | | | | | | | | | | | | | | | | | | | | | | | | | | | | | | | | | | | | | |
|  | 1t5t.1.A | Sarcoplasmic/endoplasmic reticulum calcium ATPase 1 isoform SERCA1A  *Structure of the (SR)Ca2+-ATPase Ca2-E1-ADP:AlF4- form* | 0.01 |  | 25.00 | 0.08 | 277-300 | X-ray | 2.90 | monomer | 1 x ALF, 2 x CA, 1 x MG, 1 x K, 1 x ADP | HHblits | 0.28 |
| ``` target    MVLEKQLGNGCTWIDLDLGKLNKLEDLSEIYGLDKETIEYALDRNERAHMDYHRESETVTFIYNVLDVKKDKAYYETFPM 1t5t.1    --------------------------------------------------------------------------------  target    TFIVEHRRLITISNTKNAYVIEQMTRYLENHDTLSIYKFLFASLEIISNAYYPVIEQMDKSRDEVNDLLRQRTTKKNLFV 1t5t.1    --------------------------------------------------------------------------------  target    LSDLETGMVYLTAAAKQNRILLEHIQGHALYRSFDEIEREQFDDAMIEAHQLVSMTDLISQILQQLSASYNNILNNNLND 1t5t.1    --------------------------------------------------------------------------------  target    NLTTLTIISVLLAVLAVVTGFFGMNVPLPLTDEPHAWLYISLASAGLWIVLSLLLRKIAKKS 1t5t.1    ------------------------------------QWLMVLKISLPVIGLDEILKFIAR-- ``` | | | | | | | | | | | | | | | | | | | | | | | | | | | | | | | | | | | | | | | | | | | | | | | | | |
|  | 3fps.1.A | Sarcoplasmic/endoplasmic reticulum calcium ATPase 1  *The Structure of Sarcoplasmic Reticulum Ca2+-ATPase Bound To Cyclopiazonic and ADP* | 0.00 |  | 25.00 | 0.08 | 277-300 | X-ray | 3.20 | monomer | 2 x MG, 1 x CZA, 1 x ADP | HHblits | 0.28 |
| ``` target    MVLEKQLGNGCTWIDLDLGKLNKLEDLSEIYGLDKETIEYALDRNERAHMDYHRESETVTFIYNVLDVKKDKAYYETFPM 3fps.1    --------------------------------------------------------------------------------  target    TFIVEHRRLITISNTKNAYVIEQMTRYLENHDTLSIYKFLFASLEIISNAYYPVIEQMDKSRDEVNDLLRQRTTKKNLFV 3fps.1    --------------------------------------------------------------------------------  target    LSDLETGMVYLTAAAKQNRILLEHIQGHALYRSFDEIEREQFDDAMIEAHQLVSMTDLISQILQQLSASYNNILNNNLND 3fps.1    --------------------------------------------------------------------------------  target    NLTTLTIISVLLAVLAVVTGFFGMNVPLPLTDEPHAWLYISLASAGLWIVLSLLLRKIAKKS 3fps.1    ------------------------------------QWLMVLKISLPVIGLDEILKFIAR-- ``` | | | | | | | | | | | | | | | | | | | | | | | | | | | | | | | | | | | | | | | | | | | | | | | | | |
|  | 3fgo.1.A | Sarcoplasmic/endoplasmic reticulum calcium ATPase 1  *Crystal Structure of the E2 magnesium fluoride complex of the (SR) Ca2+-ATPase with bound CPA and AMPPCP* | 0.00 |  | 25.00 | 0.08 | 277-300 | X-ray | 2.50 | monomer | 1 x MG, 1 x MF4, 1 x K, 1 x CZA, 1 x MN, 1 x ACP | HHblits | 0.28 |
| ``` target    MVLEKQLGNGCTWIDLDLGKLNKLEDLSEIYGLDKETIEYALDRNERAHMDYHRESETVTFIYNVLDVKKDKAYYETFPM 3fgo.1    --------------------------------------------------------------------------------  target    TFIVEHRRLITISNTKNAYVIEQMTRYLENHDTLSIYKFLFASLEIISNAYYPVIEQMDKSRDEVNDLLRQRTTKKNLFV 3fgo.1    --------------------------------------------------------------------------------  target    LSDLETGMVYLTAAAKQNRILLEHIQGHALYRSFDEIEREQFDDAMIEAHQLVSMTDLISQILQQLSASYNNILNNNLND 3fgo.1    --------------------------------------------------------------------------------  target    NLTTLTIISVLLAVLAVVTGFFGMNVPLPLTDEPHAWLYISLASAGLWIVLSLLLRKIAKKS 3fgo.1    ------------------------------------QWLMVLKISLPVIGLDEILKFIAR-- ``` | | | | | | | | | | | | | | | | | | | | | | | | | | | | | | | | | | | | | | | | | | | | | | | | | |
|  | 3fgo.2.A | Sarcoplasmic/endoplasmic reticulum calcium ATPase 1  *Crystal Structure of the E2 magnesium fluoride complex of the (SR) Ca2+-ATPase with bound CPA and AMPPCP* | 0.00 |  | 25.00 | 0.08 | 277-300 | X-ray | 2.50 | monomer | 1 x MG, 1 x MF4, 1 x K, 1 x CZA, 1 x MN, 1 x ACP | HHblits | 0.28 |
| ``` target    MVLEKQLGNGCTWIDLDLGKLNKLEDLSEIYGLDKETIEYALDRNERAHMDYHRESETVTFIYNVLDVKKDKAYYETFPM 3fgo.2    --------------------------------------------------------------------------------  target    TFIVEHRRLITISNTKNAYVIEQMTRYLENHDTLSIYKFLFASLEIISNAYYPVIEQMDKSRDEVNDLLRQRTTKKNLFV 3fgo.2    --------------------------------------------------------------------------------  target    LSDLETGMVYLTAAAKQNRILLEHIQGHALYRSFDEIEREQFDDAMIEAHQLVSMTDLISQILQQLSASYNNILNNNLND 3fgo.2    --------------------------------------------------------------------------------  target    NLTTLTIISVLLAVLAVVTGFFGMNVPLPLTDEPHAWLYISLASAGLWIVLSLLLRKIAKKS 3fgo.2    ------------------------------------QWLMVLKISLPVIGLDEILKFIAR-- ``` | | | | | | | | | | | | | | | | | | | | | | | | | | | | | | | | | | | | | | | | | | | | | | | | | |
|  | 3fpb.1.A | Sarcoplasmic/endoplasmic reticulum calcium ATPase 1  *The Structure of Sarcoplasmic Reticulum Ca2+-ATPase Bound To Cyclopiazonic acid with ATP* | 0.01 |  | 25.00 | 0.08 | 277-300 | X-ray | 2.55 | monomer | 2 x MG, 1 x MF4, 1 x K, 1 x CZA, 1 x ATP | HHblits | 0.28 |
| ``` target    MVLEKQLGNGCTWIDLDLGKLNKLEDLSEIYGLDKETIEYALDRNERAHMDYHRESETVTFIYNVLDVKKDKAYYETFPM 3fpb.1    --------------------------------------------------------------------------------  target    TFIVEHRRLITISNTKNAYVIEQMTRYLENHDTLSIYKFLFASLEIISNAYYPVIEQMDKSRDEVNDLLRQRTTKKNLFV 3fpb.1    --------------------------------------------------------------------------------  target    LSDLETGMVYLTAAAKQNRILLEHIQGHALYRSFDEIEREQFDDAMIEAHQLVSMTDLISQILQQLSASYNNILNNNLND 3fpb.1    --------------------------------------------------------------------------------  target    NLTTLTIISVLLAVLAVVTGFFGMNVPLPLTDEPHAWLYISLASAGLWIVLSLLLRKIAKKS 3fpb.1    ------------------------------------QWLMVLKISLPVIGLDEILKFIAR-- ``` | | | | | | | | | | | | | | | | | | | | | | | | | | | | | | | | | | | | | | | | | | | | | | | | | |
|  | 1xp5.1.A | Sarcoplasmic/endoplasmic reticulum calcium ATPase 1  *Structure Of The (Sr)Ca2+-ATPase E2-AlF4- Form* | 0.01 |  | 25.00 | 0.08 | 277-300 | X-ray | 3.00 | monomer | 1 x MG, 1 x ALF, 1 x K, 1 x TG1 | HHblits | 0.28 |
| ``` target    MVLEKQLGNGCTWIDLDLGKLNKLEDLSEIYGLDKETIEYALDRNERAHMDYHRESETVTFIYNVLDVKKDKAYYETFPM 1xp5.1    --------------------------------------------------------------------------------  target    TFIVEHRRLITISNTKNAYVIEQMTRYLENHDTLSIYKFLFASLEIISNAYYPVIEQMDKSRDEVNDLLRQRTTKKNLFV 1xp5.1    --------------------------------------------------------------------------------  target    LSDLETGMVYLTAAAKQNRILLEHIQGHALYRSFDEIEREQFDDAMIEAHQLVSMTDLISQILQQLSASYNNILNNNLND 1xp5.1    --------------------------------------------------------------------------------  target    NLTTLTIISVLLAVLAVVTGFFGMNVPLPLTDEPHAWLYISLASAGLWIVLSLLLRKIAKKS 1xp5.1    ------------------------------------QWLMVLKISLPVIGLDEILKFIAR-- ``` | | | | | | | | | | | | | | | | | | | | | | | | | | | | | | | | | | | | | | | | | | | | | | | | | |
|  | 1wpg.1.A | Sarcoplasmic/endoplasmic reticulum calcium ATPase 1  *Crystal structure of the SR CA2+-ATPase with MGF4* | 0.01 |  | 25.00 | 0.08 | 277-300 | X-ray | 2.30 | homo-tetramer | 6 x MG, 4 x MF4, 4 x ADP, 4 x TG1 | HHblits | 0.28 |
| ``` target    MVLEKQLGNGCTWIDLDLGKLNKLEDLSEIYGLDKETIEYALDRNERAHMDYHRESETVTFIYNVLDVKKDKAYYETFPM 1wpg.1    --------------------------------------------------------------------------------  target    TFIVEHRRLITISNTKNAYVIEQMTRYLENHDTLSIYKFLFASLEIISNAYYPVIEQMDKSRDEVNDLLRQRTTKKNLFV 1wpg.1    --------------------------------------------------------------------------------  target    LSDLETGMVYLTAAAKQNRILLEHIQGHALYRSFDEIEREQFDDAMIEAHQLVSMTDLISQILQQLSASYNNILNNNLND 1wpg.1    --------------------------------------------------------------------------------  target    NLTTLTIISVLLAVLAVVTGFFGMNVPLPLTDEPHAWLYISLASAGLWIVLSLLLRKIAKKS 1wpg.1    ------------------------------------QWLMVLKISLPVIGLDEILKFIAR-- ``` | | | | | | | | | | | | | | | | | | | | | | | | | | | | | | | | | | | | | | | | | | | | | | | | | |
|  | 1wpg.1.B | Sarcoplasmic/endoplasmic reticulum calcium ATPase 1  *Crystal structure of the SR CA2+-ATPase with MGF4* | 0.00 |  | 25.00 | 0.08 | 277-300 | X-ray | 2.30 | homo-tetramer | 6 x MG, 4 x MF4, 4 x ADP, 4 x TG1 | HHblits | 0.28 |
| ``` target    MVLEKQLGNGCTWIDLDLGKLNKLEDLSEIYGLDKETIEYALDRNERAHMDYHRESETVTFIYNVLDVKKDKAYYETFPM 1wpg.1    --------------------------------------------------------------------------------  target    TFIVEHRRLITISNTKNAYVIEQMTRYLENHDTLSIYKFLFASLEIISNAYYPVIEQMDKSRDEVNDLLRQRTTKKNLFV 1wpg.1    --------------------------------------------------------------------------------  target    LSDLETGMVYLTAAAKQNRILLEHIQGHALYRSFDEIEREQFDDAMIEAHQLVSMTDLISQILQQLSASYNNILNNNLND 1wpg.1    --------------------------------------------------------------------------------  target    NLTTLTIISVLLAVLAVVTGFFGMNVPLPLTDEPHAWLYISLASAGLWIVLSLLLRKIAKKS 1wpg.1    ------------------------------------QWLMVLKISLPVIGLDEILKFIAR-- ``` | | | | | | | | | | | | | | | | | | | | | | | | | | | | | | | | | | | | | | | | | | | | | | | | | |
|  | 3b9b.1.A | Sarcoplasmic/endoplasmic reticulum calcium ATPase 1  *Structure of the E2 beryllium fluoride complex of the SERCA Ca2+-ATPase* | 0.01 |  | 25.00 | 0.08 | 277-300 | X-ray | 2.65 | monomer | 2 x MG, 1 x BEF | HHblits | 0.28 |
| ``` target    MVLEKQLGNGCTWIDLDLGKLNKLEDLSEIYGLDKETIEYALDRNERAHMDYHRESETVTFIYNVLDVKKDKAYYETFPM 3b9b.1    --------------------------------------------------------------------------------  target    TFIVEHRRLITISNTKNAYVIEQMTRYLENHDTLSIYKFLFASLEIISNAYYPVIEQMDKSRDEVNDLLRQRTTKKNLFV 3b9b.1    --------------------------------------------------------------------------------  target    LSDLETGMVYLTAAAKQNRILLEHIQGHALYRSFDEIEREQFDDAMIEAHQLVSMTDLISQILQQLSASYNNILNNNLND 3b9b.1    --------------------------------------------------------------------------------  target    NLTTLTIISVLLAVLAVVTGFFGMNVPLPLTDEPHAWLYISLASAGLWIVLSLLLRKIAKKS 3b9b.1    ------------------------------------QWLMVLKISLPVIGLDEILKFIAR-- ``` | | | | | | | | | | | | | | | | | | | | | | | | | | | | | | | | | | | | | | | | | | | | | | | | | |
|  | 2agv.1.A | Sarcoplasmic/endoplasmic reticulum calcium ATPase 1  *Crystal structure of the SR CA2+-ATPASE with BHQ and TG* | 0.01 |  | 25.00 | 0.08 | 277-300 | X-ray | 2.40 | homo-dimer | 2 x TG1, 2 x BHQ, 6 x PTY | HHblits | 0.28 |
| ``` target    MVLEKQLGNGCTWIDLDLGKLNKLEDLSEIYGLDKETIEYALDRNERAHMDYHRESETVTFIYNVLDVKKDKAYYETFPM 2agv.1    --------------------------------------------------------------------------------  target    TFIVEHRRLITISNTKNAYVIEQMTRYLENHDTLSIYKFLFASLEIISNAYYPVIEQMDKSRDEVNDLLRQRTTKKNLFV 2agv.1    --------------------------------------------------------------------------------  target    LSDLETGMVYLTAAAKQNRILLEHIQGHALYRSFDEIEREQFDDAMIEAHQLVSMTDLISQILQQLSASYNNILNNNLND 2agv.1    --------------------------------------------------------------------------------  target    NLTTLTIISVLLAVLAVVTGFFGMNVPLPLTDEPHAWLYISLASAGLWIVLSLLLRKIAKKS 2agv.1    ------------------------------------QWLMVLKISLPVIGLDEILKFIAR-- ``` | | | | | | | | | | | | | | | | | | | | | | | | | | | | | | | | | | | | | | | | | | | | | | | | | |
|  | 1vfp.1.A | Sarcoplasmic/endoplasmic reticulum calcium ATPase 1  *Crystal structure of the SR CA2+-ATPase with bound AMPPCP* | 0.00 |  | 25.00 | 0.08 | 277-300 | X-ray | 2.90 | homo-dimer | 4 x CA, 2 x MG, 2 x ACP | HHblits | 0.28 |
| ``` target    MVLEKQLGNGCTWIDLDLGKLNKLEDLSEIYGLDKETIEYALDRNERAHMDYHRESETVTFIYNVLDVKKDKAYYETFPM 1vfp.1    --------------------------------------------------------------------------------  target    TFIVEHRRLITISNTKNAYVIEQMTRYLENHDTLSIYKFLFASLEIISNAYYPVIEQMDKSRDEVNDLLRQRTTKKNLFV 1vfp.1    --------------------------------------------------------------------------------  target    LSDLETGMVYLTAAAKQNRILLEHIQGHALYRSFDEIEREQFDDAMIEAHQLVSMTDLISQILQQLSASYNNILNNNLND 1vfp.1    --------------------------------------------------------------------------------  target    NLTTLTIISVLLAVLAVVTGFFGMNVPLPLTDEPHAWLYISLASAGLWIVLSLLLRKIAKKS 1vfp.1    ------------------------------------QWLMVLKISLPVIGLDEILKFIAR-- ``` | | | | | | | | | | | | | | | | | | | | | | | | | | | | | | | | | | | | | | | | | | | | | | | | | |
|  | 2yfy.1.A | SARCOPLASMIC/ENDOPLASMIC RETICULUM CALCIUM ATPASE 1  *SERCA IN THE HNE2 STATE COMPLEXED WITH DEBUTANOYL THAPSIGARGIN* | 0.01 |  | 25.00 | 0.08 | 277-300 | X-ray | 3.10 | monomer | 1 x 9TN, 1 x K, 1 x MG | HHblits | 0.28 |
| ``` target    MVLEKQLGNGCTWIDLDLGKLNKLEDLSEIYGLDKETIEYALDRNERAHMDYHRESETVTFIYNVLDVKKDKAYYETFPM 2yfy.1    --------------------------------------------------------------------------------  target    TFIVEHRRLITISNTKNAYVIEQMTRYLENHDTLSIYKFLFASLEIISNAYYPVIEQMDKSRDEVNDLLRQRTTKKNLFV 2yfy.1    --------------------------------------------------------------------------------  target    LSDLETGMVYLTAAAKQNRILLEHIQGHALYRSFDEIEREQFDDAMIEAHQLVSMTDLISQILQQLSASYNNILNNNLND 2yfy.1    --------------------------------------------------------------------------------  target    NLTTLTIISVLLAVLAVVTGFFGMNVPLPLTDEPHAWLYISLASAGLWIVLSLLLRKIAKKS 2yfy.1    ------------------------------------QWLMVLKISLPVIGLDEILKFIAR-- ``` | | | | | | | | | | | | | | | | | | | | | | | | | | | | | | | | | | | | | | | | | | | | | | | | | |
|  | 1iwo.1.A | Sarcoplasmic/endoplasmic reticulum calcium ATPase 1  *Crystal structure of the SR Ca2+-ATPase in the absence of Ca2+* | 0.00 |  | 25.00 | 0.08 | 277-300 | X-ray | 3.10 | homo-dimer | 2 x TG1 | HHblits | 0.28 |
| ``` target    MVLEKQLGNGCTWIDLDLGKLNKLEDLSEIYGLDKETIEYALDRNERAHMDYHRESETVTFIYNVLDVKKDKAYYETFPM 1iwo.1    --------------------------------------------------------------------------------  target    TFIVEHRRLITISNTKNAYVIEQMTRYLENHDTLSIYKFLFASLEIISNAYYPVIEQMDKSRDEVNDLLRQRTTKKNLFV 1iwo.1    --------------------------------------------------------------------------------  target    LSDLETGMVYLTAAAKQNRILLEHIQGHALYRSFDEIEREQFDDAMIEAHQLVSMTDLISQILQQLSASYNNILNNNLND 1iwo.1    --------------------------------------------------------------------------------  target    NLTTLTIISVLLAVLAVVTGFFGMNVPLPLTDEPHAWLYISLASAGLWIVLSLLLRKIAKKS 1iwo.1    ------------------------------------QWLMVLKISLPVIGLDEILKFIAR-- ``` | | | | | | | | | | | | | | | | | | | | | | | | | | | | | | | | | | | | | | | | | | | | | | | | | |
|  | 3nal.1.A | SERCA1a  *SR Ca(2+)-ATPase in the HnE2 state complexed with the Thapsigargin derivative DTB* | 0.01 |  | 25.00 | 0.08 | 277-300 | X-ray | 2.65 | monomer | 1 x K, 1 x MG, 1 x DBK | HHblits | 0.28 |
| ``` target    MVLEKQLGNGCTWIDLDLGKLNKLEDLSEIYGLDKETIEYALDRNERAHMDYHRESETVTFIYNVLDVKKDKAYYETFPM 3nal.1    --------------------------------------------------------------------------------  target    TFIVEHRRLITISNTKNAYVIEQMTRYLENHDTLSIYKFLFASLEIISNAYYPVIEQMDKSRDEVNDLLRQRTTKKNLFV 3nal.1    --------------------------------------------------------------------------------  target    LSDLETGMVYLTAAAKQNRILLEHIQGHALYRSFDEIEREQFDDAMIEAHQLVSMTDLISQILQQLSASYNNILNNNLND 3nal.1    --------------------------------------------------------------------------------  target    NLTTLTIISVLLAVLAVVTGFFGMNVPLPLTDEPHAWLYISLASAGLWIVLSLLLRKIAKKS 3nal.1    ------------------------------------QWLMVLKISLPVIGLDEILKFIAR-- ``` | | | | | | | | | | | | | | | | | | | | | | | | | | | | | | | | | | | | | | | | | | | | | | | | | |
|  | 2c8l.1.A | SARCOPLASMIC/ENDOPLASMIC RETICULUM CALCIUM ATPASE 1  *CRYSTAL STRUCTURE OF (SR) CALCIUM-ATPASE E2(TG) FORM* | 0.00 |  | 25.00 | 0.08 | 277-300 | X-ray | 3.10 | monomer | 1 x TG1 | HHblits | 0.28 |
| ``` target    MVLEKQLGNGCTWIDLDLGKLNKLEDLSEIYGLDKETIEYALDRNERAHMDYHRESETVTFIYNVLDVKKDKAYYETFPM 2c8l.1    --------------------------------------------------------------------------------  target    TFIVEHRRLITISNTKNAYVIEQMTRYLENHDTLSIYKFLFASLEIISNAYYPVIEQMDKSRDEVNDLLRQRTTKKNLFV 2c8l.1    --------------------------------------------------------------------------------  target    LSDLETGMVYLTAAAKQNRILLEHIQGHALYRSFDEIEREQFDDAMIEAHQLVSMTDLISQILQQLSASYNNILNNNLND 2c8l.1    --------------------------------------------------------------------------------  target    NLTTLTIISVLLAVLAVVTGFFGMNVPLPLTDEPHAWLYISLASAGLWIVLSLLLRKIAKKS 2c8l.1    ------------------------------------QWLMVLKISLPVIGLDEILKFIAR-- ``` | | | | | | | | | | | | | | | | | | | | | | | | | | | | | | | | | | | | | | | | | | | | | | | | | |
|  | 2c9m.1.A | SARCOPLASMIC/ENDOPLASMIC RETICULUM CALCIUM ATPASE 1  *STRUCTURE OF (SR) CALCIUM-ATPASE IN THE CA2E1 STATE SOLVED IN A P1 CRYSTAL FORM.* | 0.01 |  | 25.00 | 0.08 | 277-300 | X-ray | 3.00 | monomer | 4 x CA, 1 x K | HHblits | 0.28 |
| ``` target    MVLEKQLGNGCTWIDLDLGKLNKLEDLSEIYGLDKETIEYALDRNERAHMDYHRESETVTFIYNVLDVKKDKAYYETFPM 2c9m.1    --------------------------------------------------------------------------------  target    TFIVEHRRLITISNTKNAYVIEQMTRYLENHDTLSIYKFLFASLEIISNAYYPVIEQMDKSRDEVNDLLRQRTTKKNLFV 2c9m.1    --------------------------------------------------------------------------------  target    LSDLETGMVYLTAAAKQNRILLEHIQGHALYRSFDEIEREQFDDAMIEAHQLVSMTDLISQILQQLSASYNNILNNNLND 2c9m.1    --------------------------------------------------------------------------------  target    NLTTLTIISVLLAVLAVVTGFFGMNVPLPLTDEPHAWLYISLASAGLWIVLSLLLRKIAKKS 2c9m.1    ------------------------------------QWLMVLKISLPVIGLDEILKFIAR-- ``` | | | | | | | | | | | | | | | | | | | | | | | | | | | | | | | | | | | | | | | | | | | | | | | | | |
|  | 2c9m.2.A | SARCOPLASMIC/ENDOPLASMIC RETICULUM CALCIUM ATPASE 1  *STRUCTURE OF (SR) CALCIUM-ATPASE IN THE CA2E1 STATE SOLVED IN A P1 CRYSTAL FORM.* | 0.01 |  | 25.00 | 0.08 | 277-300 | X-ray | 3.00 | monomer | 3 x CA, 1 x K | HHblits | 0.28 |
| ``` target    MVLEKQLGNGCTWIDLDLGKLNKLEDLSEIYGLDKETIEYALDRNERAHMDYHRESETVTFIYNVLDVKKDKAYYETFPM 2c9m.2    --------------------------------------------------------------------------------  target    TFIVEHRRLITISNTKNAYVIEQMTRYLENHDTLSIYKFLFASLEIISNAYYPVIEQMDKSRDEVNDLLRQRTTKKNLFV 2c9m.2    --------------------------------------------------------------------------------  target    LSDLETGMVYLTAAAKQNRILLEHIQGHALYRSFDEIEREQFDDAMIEAHQLVSMTDLISQILQQLSASYNNILNNNLND 2c9m.2    --------------------------------------------------------------------------------  target    NLTTLTIISVLLAVLAVVTGFFGMNVPLPLTDEPHAWLYISLASAGLWIVLSLLLRKIAKKS 2c9m.2    ------------------------------------QWLMVLKISLPVIGLDEILKFIAR-- ``` | | | | | | | | | | | | | | | | | | | | | | | | | | | | | | | | | | | | | | | | | | | | | | | | | |
|  | 3b9r.2.A | Sarcoplasmic/endoplasmic reticulum calcium ATPase 1  *SERCA Ca2+-ATPase E2 aluminium fluoride complex without thapsigargin* | 0.00 |  | 25.00 | 0.08 | 277-300 | X-ray | 3.00 | monomer | 1 x ALF, 1 x MG, 1 x K, 1 x ACP | HHblits | 0.28 |
| ``` target    MVLEKQLGNGCTWIDLDLGKLNKLEDLSEIYGLDKETIEYALDRNERAHMDYHRESETVTFIYNVLDVKKDKAYYETFPM 3b9r.2    --------------------------------------------------------------------------------  target    TFIVEHRRLITISNTKNAYVIEQMTRYLENHDTLSIYKFLFASLEIISNAYYPVIEQMDKSRDEVNDLLRQRTTKKNLFV 3b9r.2    --------------------------------------------------------------------------------  target    LSDLETGMVYLTAAAKQNRILLEHIQGHALYRSFDEIEREQFDDAMIEAHQLVSMTDLISQILQQLSASYNNILNNNLND 3b9r.2    --------------------------------------------------------------------------------  target    NLTTLTIISVLLAVLAVVTGFFGMNVPLPLTDEPHAWLYISLASAGLWIVLSLLLRKIAKKS 3b9r.2    ------------------------------------QWLMVLKISLPVIGLDEILKFIAR-- ``` | | | | | | | | | | | | | | | | | | | | | | | | | | | | | | | | | | | | | | | | | | | | | | | | | |
|  | 3b9r.3.A | Sarcoplasmic/endoplasmic reticulum calcium ATPase 1  *SERCA Ca2+-ATPase E2 aluminium fluoride complex without thapsigargin* | 0.00 |  | 25.00 | 0.08 | 277-300 | X-ray | 3.00 | homo-dimer | 2 x ALF, 2 x MG, 2 x K, 2 x ACP | HHblits | 0.28 |
| ``` target    MVLEKQLGNGCTWIDLDLGKLNKLEDLSEIYGLDKETIEYALDRNERAHMDYHRESETVTFIYNVLDVKKDKAYYETFPM 3b9r.3    --------------------------------------------------------------------------------  target    TFIVEHRRLITISNTKNAYVIEQMTRYLENHDTLSIYKFLFASLEIISNAYYPVIEQMDKSRDEVNDLLRQRTTKKNLFV 3b9r.3    --------------------------------------------------------------------------------  target    LSDLETGMVYLTAAAKQNRILLEHIQGHALYRSFDEIEREQFDDAMIEAHQLVSMTDLISQILQQLSASYNNILNNNLND 3b9r.3    --------------------------------------------------------------------------------  target    NLTTLTIISVLLAVLAVVTGFFGMNVPLPLTDEPHAWLYISLASAGLWIVLSLLLRKIAKKS 3b9r.3    ------------------------------------QWLMVLKISLPVIGLDEILKFIAR-- ``` | | | | | | | | | | | | | | | | | | | | | | | | | | | | | | | | | | | | | | | | | | | | | | | | | |
|  | 3j7t.1.B | Sarcoplasmic/endoplasmic reticulum calcium ATPase 1  *Calcium atpase structure with two bound calcium ions determined by electron crystallography of thin 3D crystals* | 0.01 |  | 25.00 | 0.08 | 277-300 | 2DX | 3.40 | homo-dimer | 4 x CA | HHblits | 0.28 |
| ``` target    MVLEKQLGNGCTWIDLDLGKLNKLEDLSEIYGLDKETIEYALDRNERAHMDYHRESETVTFIYNVLDVKKDKAYYETFPM 3j7t.1    --------------------------------------------------------------------------------  target    TFIVEHRRLITISNTKNAYVIEQMTRYLENHDTLSIYKFLFASLEIISNAYYPVIEQMDKSRDEVNDLLRQRTTKKNLFV 3j7t.1    --------------------------------------------------------------------------------  target    LSDLETGMVYLTAAAKQNRILLEHIQGHALYRSFDEIEREQFDDAMIEAHQLVSMTDLISQILQQLSASYNNILNNNLND 3j7t.1    --------------------------------------------------------------------------------  target    NLTTLTIISVLLAVLAVVTGFFGMNVPLPLTDEPHAWLYISLASAGLWIVLSLLLRKIAKKS 3j7t.1    ------------------------------------QWLMVLKISLPVIGLDEILKFIAR-- ``` | | | | | | | | | | | | | | | | | | | | | | | | | | | | | | | | | | | | | | | | | | | | | | | | | |
|  | 4y3u.1.A | Sarcoplasmic/endoplasmic reticulum calcium ATPase 1  *The structure of phospholamban bound to the calcium pump SERCA1a* | 0.01 |  | 25.00 | 0.08 | 277-300 | X-ray | 3.51 | hetero-1-1-1-mer | 1 x K | HHblits | 0.28 |
| ``` target    MVLEKQLGNGCTWIDLDLGKLNKLEDLSEIYGLDKETIEYALDRNERAHMDYHRESETVTFIYNVLDVKKDKAYYETFPM 4y3u.1    --------------------------------------------------------------------------------  target    TFIVEHRRLITISNTKNAYVIEQMTRYLENHDTLSIYKFLFASLEIISNAYYPVIEQMDKSRDEVNDLLRQRTTKKNLFV 4y3u.1    --------------------------------------------------------------------------------  target    LSDLETGMVYLTAAAKQNRILLEHIQGHALYRSFDEIEREQFDDAMIEAHQLVSMTDLISQILQQLSASYNNILNNNLND 4y3u.1    --------------------------------------------------------------------------------  target    NLTTLTIISVLLAVLAVVTGFFGMNVPLPLTDEPHAWLYISLASAGLWIVLSLLLRKIAKKS 4y3u.1    ------------------------------------QWLMVLKISLPVIGLDEILKFIAR-- ``` | | | | | | | | | | | | | | | | | | | | | | | | | | | | | | | | | | | | | | | | | | | | | | | | | |
|  | 3n5k.2.A | Sarcoplasmic/endoplasmic reticulum calcium ATPase 1  *Structure Of The (Sr)Ca2+-ATPase E2-AlF4- Form* | 0.01 |  | 25.00 | 0.08 | 277-300 | X-ray | 2.20 | monomer | 1 x TG1, 1 x MG, 1 x ALF, 1 x K | HHblits | 0.28 |
| ``` target    MVLEKQLGNGCTWIDLDLGKLNKLEDLSEIYGLDKETIEYALDRNERAHMDYHRESETVTFIYNVLDVKKDKAYYETFPM 3n5k.2    --------------------------------------------------------------------------------  target    TFIVEHRRLITISNTKNAYVIEQMTRYLENHDTLSIYKFLFASLEIISNAYYPVIEQMDKSRDEVNDLLRQRTTKKNLFV 3n5k.2    --------------------------------------------------------------------------------  target    LSDLETGMVYLTAAAKQNRILLEHIQGHALYRSFDEIEREQFDDAMIEAHQLVSMTDLISQILQQLSASYNNILNNNLND 3n5k.2    --------------------------------------------------------------------------------  target    NLTTLTIISVLLAVLAVVTGFFGMNVPLPLTDEPHAWLYISLASAGLWIVLSLLLRKIAKKS 3n5k.2    ------------------------------------QWLMVLKISLPVIGLDEILKFIAR-- ``` | | | | | | | | | | | | | | | | | | | | | | | | | | | | | | | | | | | | | | | | | | | | | | | | | |
|  | 3n5k.1.A | Sarcoplasmic/endoplasmic reticulum calcium ATPase 1  *Structure Of The (Sr)Ca2+-ATPase E2-AlF4- Form* | 0.01 |  | 25.00 | 0.08 | 277-300 | X-ray | 2.20 | monomer | 1 x TG1, 1 x MG, 1 x ALF, 1 x K | HHblits | 0.28 |
| ``` target    MVLEKQLGNGCTWIDLDLGKLNKLEDLSEIYGLDKETIEYALDRNERAHMDYHRESETVTFIYNVLDVKKDKAYYETFPM 3n5k.1    --------------------------------------------------------------------------------  target    TFIVEHRRLITISNTKNAYVIEQMTRYLENHDTLSIYKFLFASLEIISNAYYPVIEQMDKSRDEVNDLLRQRTTKKNLFV 3n5k.1    --------------------------------------------------------------------------------  target    LSDLETGMVYLTAAAKQNRILLEHIQGHALYRSFDEIEREQFDDAMIEAHQLVSMTDLISQILQQLSASYNNILNNNLND 3n5k.1    --------------------------------------------------------------------------------  target    NLTTLTIISVLLAVLAVVTGFFGMNVPLPLTDEPHAWLYISLASAGLWIVLSLLLRKIAKKS 3n5k.1    ------------------------------------QWLMVLKISLPVIGLDEILKFIAR-- ``` | | | | | | | | | | | | | | | | | | | | | | | | | | | | | | | | | | | | | | | | | | | | | | | | | |
|  | 4h1w.1.A | SERCA1a  *E1 structure of the (SR) Ca2+-ATPase in complex with Sarcolipin* | 0.01 |  | 25.00 | 0.08 | 277-300 | X-ray | 3.10 | hetero-oligomer | 1 x K, 2 x MG, 1 x ACP | HHblits | 0.28 |
| ``` target    MVLEKQLGNGCTWIDLDLGKLNKLEDLSEIYGLDKETIEYALDRNERAHMDYHRESETVTFIYNVLDVKKDKAYYETFPM 4h1w.1    --------------------------------------------------------------------------------  target    TFIVEHRRLITISNTKNAYVIEQMTRYLENHDTLSIYKFLFASLEIISNAYYPVIEQMDKSRDEVNDLLRQRTTKKNLFV 4h1w.1    --------------------------------------------------------------------------------  target    LSDLETGMVYLTAAAKQNRILLEHIQGHALYRSFDEIEREQFDDAMIEAHQLVSMTDLISQILQQLSASYNNILNNNLND 4h1w.1    --------------------------------------------------------------------------------  target    NLTTLTIISVLLAVLAVVTGFFGMNVPLPLTDEPHAWLYISLASAGLWIVLSLLLRKIAKKS 4h1w.1    ------------------------------------QWLMVLKISLPVIGLDEILKFIAR-- ``` | | | | | | | | | | | | | | | | | | | | | | | | | | | | | | | | | | | | | | | | | | | | | | | | | |
|  | 5ncq.1.A | Sarcoplasmic/endoplasmic reticulum calcium ATPase 1  *Structure of the (SR) Ca2+-ATPase bound to a Tetrahydrocarbazole and TNP-ATP* | 0.01 |  | 25.00 | 0.08 | 277-300 | X-ray | 3.00 | monomer | 1 x 128, 1 x K, 2 x PCW, 1 x 8T8 | HHblits | 0.28 |
| ``` target    MVLEKQLGNGCTWIDLDLGKLNKLEDLSEIYGLDKETIEYALDRNERAHMDYHRESETVTFIYNVLDVKKDKAYYETFPM 5ncq.1    --------------------------------------------------------------------------------  target    TFIVEHRRLITISNTKNAYVIEQMTRYLENHDTLSIYKFLFASLEIISNAYYPVIEQMDKSRDEVNDLLRQRTTKKNLFV 5ncq.1    --------------------------------------------------------------------------------  target    LSDLETGMVYLTAAAKQNRILLEHIQGHALYRSFDEIEREQFDDAMIEAHQLVSMTDLISQILQQLSASYNNILNNNLND 5ncq.1    --------------------------------------------------------------------------------  target    NLTTLTIISVLLAVLAVVTGFFGMNVPLPLTDEPHAWLYISLASAGLWIVLSLLLRKIAKKS 5ncq.1    ------------------------------------QWLMVLKISLPVIGLDEILKFIAR-- ``` | | | | | | | | | | | | | | | | | | | | | | | | | | | | | | | | | | | | | | | | | | | | | | | | | |
|  | 1kju.1.A | Sarcoplasmic/endoplasmic reticulum calcium ATPase 1a  *Ca2+-ATPase in the E2 State* | 0.00 |  | 25.00 | 0.08 | 277-300 | EM | 6.00 | monomer |  | HHblits | 0.28 |
| ``` target    MVLEKQLGNGCTWIDLDLGKLNKLEDLSEIYGLDKETIEYALDRNERAHMDYHRESETVTFIYNVLDVKKDKAYYETFPM 1kju.1    --------------------------------------------------------------------------------  target    TFIVEHRRLITISNTKNAYVIEQMTRYLENHDTLSIYKFLFASLEIISNAYYPVIEQMDKSRDEVNDLLRQRTTKKNLFV 1kju.1    --------------------------------------------------------------------------------  target    LSDLETGMVYLTAAAKQNRILLEHIQGHALYRSFDEIEREQFDDAMIEAHQLVSMTDLISQILQQLSASYNNILNNNLND 1kju.1    --------------------------------------------------------------------------------  target    NLTTLTIISVLLAVLAVVTGFFGMNVPLPLTDEPHAWLYISLASAGLWIVLSLLLRKIAKKS 1kju.1    ------------------------------------QWLMVLKISLPVIGLDEILKFIAR-- ``` | | | | | | | | | | | | | | | | | | | | | | | | | | | | | | | | | | | | | | | | | | | | | | | | | |
|  | 4xou.1.A | Sarcoplasmic/endoplasmic reticulum calcium ATPase 1  *Crystal structure of the SR Ca2+-ATPase in the Ca2-E1-MgAMPPCP form determined by serial femtosecond crystallography using an X-ray free-electron laser.* | 0.01 |  | 25.00 | 0.08 | 277-300 | X-ray | 2.80 | monomer | 3 x CA, 1 x K, 1 x ACP | HHblits | 0.28 |
| ``` target    MVLEKQLGNGCTWIDLDLGKLNKLEDLSEIYGLDKETIEYALDRNERAHMDYHRESETVTFIYNVLDVKKDKAYYETFPM 4xou.1    --------------------------------------------------------------------------------  target    TFIVEHRRLITISNTKNAYVIEQMTRYLENHDTLSIYKFLFASLEIISNAYYPVIEQMDKSRDEVNDLLRQRTTKKNLFV 4xou.1    --------------------------------------------------------------------------------  target    LSDLETGMVYLTAAAKQNRILLEHIQGHALYRSFDEIEREQFDDAMIEAHQLVSMTDLISQILQQLSASYNNILNNNLND 4xou.1    --------------------------------------------------------------------------------  target    NLTTLTIISVLLAVLAVVTGFFGMNVPLPLTDEPHAWLYISLASAGLWIVLSLLLRKIAKKS 4xou.1    ------------------------------------QWLMVLKISLPVIGLDEILKFIAR-- ``` | | | | | | | | | | | | | | | | | | | | | | | | | | | | | | | | | | | | | | | | | | | | | | | | | |
|  | 6hef.1.A | Sarcoplasmic/endoplasmic reticulum calcium ATPase 1  *Room temperature structure of the (SR)Ca2+-ATPase Ca2-E1-CaAMPPCP form* | 0.01 |  | 25.00 | 0.08 | 277-300 | X-ray | 3.54 | monomer | 1 x ACP, 3 x CA, 1 x K, 1 x PCW | HHblits | 0.28 |
| ``` target    MVLEKQLGNGCTWIDLDLGKLNKLEDLSEIYGLDKETIEYALDRNERAHMDYHRESETVTFIYNVLDVKKDKAYYETFPM 6hef.1    --------------------------------------------------------------------------------  target    TFIVEHRRLITISNTKNAYVIEQMTRYLENHDTLSIYKFLFASLEIISNAYYPVIEQMDKSRDEVNDLLRQRTTKKNLFV 6hef.1    --------------------------------------------------------------------------------  target    LSDLETGMVYLTAAAKQNRILLEHIQGHALYRSFDEIEREQFDDAMIEAHQLVSMTDLISQILQQLSASYNNILNNNLND 6hef.1    --------------------------------------------------------------------------------  target    NLTTLTIISVLLAVLAVVTGFFGMNVPLPLTDEPHAWLYISLASAGLWIVLSLLLRKIAKKS 6hef.1    ------------------------------------QWLMVLKISLPVIGLDEILKFIAR-- ``` | | | | | | | | | | | | | | | | | | | | | | | | | | | | | | | | | | | | | | | | | | | | | | | | | |
|  | 5a3r.1.A | SARCOPLASMIC/ENDOPLASMIC RETICULUM CALCIUM ATPASE 1  *Crystal structure of the (SR) Calcium ATPase E2.BeF3- complex bound to TNP-AMPPCP* | 0.01 |  | 25.00 | 0.08 | 277-300 | X-ray | 3.05 | monomer | 1 x DL5, 1 x K, 2 x MG | HHblits | 0.28 |
| ``` target    MVLEKQLGNGCTWIDLDLGKLNKLEDLSEIYGLDKETIEYALDRNERAHMDYHRESETVTFIYNVLDVKKDKAYYETFPM 5a3r.1    --------------------------------------------------------------------------------  target    TFIVEHRRLITISNTKNAYVIEQMTRYLENHDTLSIYKFLFASLEIISNAYYPVIEQMDKSRDEVNDLLRQRTTKKNLFV 5a3r.1    --------------------------------------------------------------------------------  target    LSDLETGMVYLTAAAKQNRILLEHIQGHALYRSFDEIEREQFDDAMIEAHQLVSMTDLISQILQQLSASYNNILNNNLND 5a3r.1    --------------------------------------------------------------------------------  target    NLTTLTIISVLLAVLAVVTGFFGMNVPLPLTDEPHAWLYISLASAGLWIVLSLLLRKIAKKS 5a3r.1    ------------------------------------QWLMVLKISLPVIGLDEILKFIAR-- ``` | | | | | | | | | | | | | | | | | | | | | | | | | | | | | | | | | | | | | | | | | | | | | | | | | |
|  | 6yaa.1.A | Sarcoplasmic/endoplasmic reticulum calcium ATPase 1  *Structure of the (SR) Ca2+-ATPase bound to the inhibitor compound CAD204520 and TNP-ATP* | 0.00 |  | 25.00 | 0.08 | 277-300 | X-ray | 3.40 | monomer | 1 x 128, 1 x OHW, 1 x K | HHblits | 0.28 |
| ``` target    MVLEKQLGNGCTWIDLDLGKLNKLEDLSEIYGLDKETIEYALDRNERAHMDYHRESETVTFIYNVLDVKKDKAYYETFPM 6yaa.1    --------------------------------------------------------------------------------  target    TFIVEHRRLITISNTKNAYVIEQMTRYLENHDTLSIYKFLFASLEIISNAYYPVIEQMDKSRDEVNDLLRQRTTKKNLFV 6yaa.1    --------------------------------------------------------------------------------  target    LSDLETGMVYLTAAAKQNRILLEHIQGHALYRSFDEIEREQFDDAMIEAHQLVSMTDLISQILQQLSASYNNILNNNLND 6yaa.1    --------------------------------------------------------------------------------  target    NLTTLTIISVLLAVLAVVTGFFGMNVPLPLTDEPHAWLYISLASAGLWIVLSLLLRKIAKKS 6yaa.1    ------------------------------------QWLMVLKISLPVIGLDEILKFIAR-- ``` | | | | | | | | | | | | | | | | | | | | | | | | | | | | | | | | | | | | | | | | | | | | | | | | | |
|  | 6yso.2.A | Sarcoplasmic/endoplasmic reticulum calcium ATPase 1  *Crystal structure of the (SR) Ca2+-ATPase solved by vanadium SAD phasing* | 0.01 |  | 25.00 | 0.08 | 277-300 | X-ray | 3.13 | monomer | 1 x 128, 1 x TG1, 1 x VN4, 2 x MG, 1 x K | HHblits | 0.28 |
| ``` target    MVLEKQLGNGCTWIDLDLGKLNKLEDLSEIYGLDKETIEYALDRNERAHMDYHRESETVTFIYNVLDVKKDKAYYETFPM 6yso.2    --------------------------------------------------------------------------------  target    TFIVEHRRLITISNTKNAYVIEQMTRYLENHDTLSIYKFLFASLEIISNAYYPVIEQMDKSRDEVNDLLRQRTTKKNLFV 6yso.2    --------------------------------------------------------------------------------  target    LSDLETGMVYLTAAAKQNRILLEHIQGHALYRSFDEIEREQFDDAMIEAHQLVSMTDLISQILQQLSASYNNILNNNLND 6yso.2    --------------------------------------------------------------------------------  target    NLTTLTIISVLLAVLAVVTGFFGMNVPLPLTDEPHAWLYISLASAGLWIVLSLLLRKIAKKS 6yso.2    ------------------------------------QWLMVLKISLPVIGLDEILKFIAR-- ``` | | | | | | | | | | | | | | | | | | | | | | | | | | | | | | | | | | | | | | | | | | | | | | | | | |
|  | 6yso.1.A | Sarcoplasmic/endoplasmic reticulum calcium ATPase 1  *Crystal structure of the (SR) Ca2+-ATPase solved by vanadium SAD phasing* | 0.01 |  | 25.00 | 0.08 | 277-300 | X-ray | 3.13 | monomer | 1 x 128, 1 x TG1, 1 x VN4, 2 x MG, 1 x K | HHblits | 0.28 |
| ``` target    MVLEKQLGNGCTWIDLDLGKLNKLEDLSEIYGLDKETIEYALDRNERAHMDYHRESETVTFIYNVLDVKKDKAYYETFPM 6yso.1    --------------------------------------------------------------------------------  target    TFIVEHRRLITISNTKNAYVIEQMTRYLENHDTLSIYKFLFASLEIISNAYYPVIEQMDKSRDEVNDLLRQRTTKKNLFV 6yso.1    --------------------------------------------------------------------------------  target    LSDLETGMVYLTAAAKQNRILLEHIQGHALYRSFDEIEREQFDDAMIEAHQLVSMTDLISQILQQLSASYNNILNNNLND 6yso.1    --------------------------------------------------------------------------------  target    NLTTLTIISVLLAVLAVVTGFFGMNVPLPLTDEPHAWLYISLASAGLWIVLSLLLRKIAKKS 6yso.1    ------------------------------------QWLMVLKISLPVIGLDEILKFIAR-- ``` | | | | | | | | | | | | | | | | | | | | | | | | | | | | | | | | | | | | | | | | | | | | | | | | | |
|  | 6vja.1.A | B-lymphocyte antigen CD20  *Structure of CD20 in complex with rituximab Fab* | 0.01 |  | 17.39 | 0.08 | 242-264 | EM | 0.00 | hetero-2-2-2-mer | 6 x Y01 | HHblits | 0.30 |
| ``` target    MVLEKQLGNGCTWIDLDLGKLNKLEDLSEIYGLDKETIEYALDRNERAHMDYHRESETVTFIYNVLDVKKDKAYYETFPM 6vja.1    --------------------------------------------------------------------------------  target    TFIVEHRRLITISNTKNAYVIEQMTRYLENHDTLSIYKFLFASLEIISNAYYPVIEQMDKSRDEVNDLLRQRTTKKNLFV 6vja.1    --------------------------------------------------------------------------------  target    LSDLETGMVYLTAAAKQNRILLEHIQGHALYRSFDEIEREQFDDAMIEAHQLVSMTDLISQILQQLSASYNNILNNNLND 6vja.1    --------------------------------------------------------------------------------  target    NLTTLTIISVLLAVLAVVTGFFGMNVPLPLTDEPHAWLYISLASAGLWIVLSLLLRKIAKKS 6vja.1    -SKTLGAVQIMNGLFHIALGGLLM-------------------------------------- ``` | | | | | | | | | | | | | | | | | | | | | | | | | | | | | | | | | | | | | | | | | | | | | | | | | |
|  | 5tcx.1.A | CD81 antigen  *Crystal structure of human tetraspanin CD81* | 0.01 |  | 12.50 | 0.08 | 244-267 | X-ray | 2.96 | monomer | 1 x CLR | HHblits | 0.26 |
| ``` target    MVLEKQLGNGCTWIDLDLGKLNKLEDLSEIYGLDKETIEYALDRNERAHMDYHRESETVTFIYNVLDVKKDKAYYETFPM 5tcx.1    --------------------------------------------------------------------------------  target    TFIVEHRRLITISNTKNAYVIEQMTRYLENHDTLSIYKFLFASLEIISNAYYPVIEQMDKSRDEVNDLLRQRTTKKNLFV 5tcx.1    --------------------------------------------------------------------------------  target    LSDLETGMVYLTAAAKQNRILLEHIQGHALYRSFDEIEREQFDDAMIEAHQLVSMTDLISQILQQLSASYNNILNNNLND 5tcx.1    --------------------------------------------------------------------------------  target    NLTTLTIISVLLAVLAVVTGFFGMNVPLPLTDEPHAWLYISLASAGLWIVLSLLLRKIAKKS 5tcx.1    ---YILIAVGAVMMFVGFLGCYGAIQE----------------------------------- ``` | | | | | | | | | | | | | | | | | | | | | | | | | | | | | | | | | | | | | | | | | | | | | | | | | |
|  | 1zbt.1.A | Peptide chain release factor 1  *Crystal structure of Peptide chain release factor 1 (RF-1) (SMU.1085) from Streptococcus mutans at 2.34 A resolution* | 0.00 |  | 18.18 | 0.07 | 130-151 | X-ray | 2.34 | monomer |  | HHblits | 0.33 |
| ``` target    MVLEKQLGNGCTWIDLDLGKLNKLEDLSEIYGLDKETIEYALDRNERAHMDYHRESETVTFIYNVLDVKKDKAYYETFPM 1zbt.1    --------------------------------------------------------------------------------  target    TFIVEHRRLITISNTKNAYVIEQMTRYLENHDTLSIYKFLFASLEIISNAYYPVIEQMDKSRDEVNDLLRQRTTKKNLFV 1zbt.1    -------------------------------------------------NIYDQLQAVEDRYEELGELLSD---------  target    LSDLETGMVYLTAAAKQNRILLEHIQGHALYRSFDEIEREQFDDAMIEAHQLVSMTDLISQILQQLSASYNNILNNNLND 1zbt.1    --------------------------------------------------------------------------------  target    NLTTLTIISVLLAVLAVVTGFFGMNVPLPLTDEPHAWLYISLASAGLWIVLSLLLRKIAKKS 1zbt.1    -------------------------------------------------------------- ``` | | | | | | | | | | | | | | | | | | | | | | | | | | | | | | | | | | | | | | | | | | | | | | | | | |
|  | 6qkz.1.E | Voltage-dependent calcium channel gamma-8 subunit  *Full length GluA1/2-gamma8 complex* | 0.01 |  | 8.33 | 0.08 | 272-295 | EM | 0.00 | hetero-2-2-2-mer | 4 x E2Q, 2 x NAG, 2 x NAG-NAG-BMA, 6 x NAG-NAG | HHblits | 0.26 |
| ``` target    MVLEKQLGNGCTWIDLDLGKLNKLEDLSEIYGLDKETIEYALDRNERAHMDYHRESETVTFIYNVLDVKKDKAYYETFPM 6qkz.1    --------------------------------------------------------------------------------  target    TFIVEHRRLITISNTKNAYVIEQMTRYLENHDTLSIYKFLFASLEIISNAYYPVIEQMDKSRDEVNDLLRQRTTKKNLFV 6qkz.1    --------------------------------------------------------------------------------  target    LSDLETGMVYLTAAAKQNRILLEHIQGHALYRSFDEIEREQFDDAMIEAHQLVSMTDLISQILQQLSASYNNILNNNLND 6qkz.1    --------------------------------------------------------------------------------  target    NLTTLTIISVLLAVLAVVTGFFGMNVPLPLTDEPHAWLYISLASAGLWIVLSLLLRKIAKKS 6qkz.1    -------------------------------HYSYGWSFYFGGLSFILAEVIGVL------- ``` | | | | | | | | | | | | | | | | | | | | | | | | | | | | | | | | | | | | | | | | | | | | | | | | | |
|  | 6tqe.1.A | ABC transporter ATP-binding protein/permease  *The structure of ABC transporter Rv1819c without addition of substrate* | 0.00 |  | 4.35 | 0.08 | 245-267 | EM | 0.00 | homo-dimer | 2 x ATP, 2 x MG | HHblits | 0.28 |
| ``` target    MVLEKQLGNGCTWIDLDLGKLNKLEDLSEIYGLDKETIEYALDRNERAHMDYHRESETVTFIYNVLDVKKDKAYYETFPM 6tqe.1    --------------------------------------------------------------------------------  target    TFIVEHRRLITISNTKNAYVIEQMTRYLENHDTLSIYKFLFASLEIISNAYYPVIEQMDKSRDEVNDLLRQRTTKKNLFV 6tqe.1    --------------------------------------------------------------------------------  target    LSDLETGMVYLTAAAKQNRILLEHIQGHALYRSFDEIEREQFDDAMIEAHQLVSMTDLISQILQQLSASYNNILNNNLND 6tqe.1    --------------------------------------------------------------------------------  target    NLTTLTIISVLLAVLAVVTGFFGMNVPLPLTDEPHAWLYISLASAGLWIVLSLLLRKIAKKS 6tqe.1    ----FGAVQSIISVISFTAILWNLSGT----------------------------------- ``` | | | | | | | | | | | | | | | | | | | | | | | | | | | | | | | | | | | | | | | | | | | | | | | | | |
|  | 6tqf.1.A | ABC transporter ATP-binding protein/permease  *The structure of ABC transporter Rv1819c in AMP-PNP bound state* | 0.00 |  | 4.35 | 0.08 | 245-267 | EM | 0.00 | homo-dimer | 10 x LMT, 2 x MG, 2 x ANP | HHblits | 0.28 |
| ``` target    MVLEKQLGNGCTWIDLDLGKLNKLEDLSEIYGLDKETIEYALDRNERAHMDYHRESETVTFIYNVLDVKKDKAYYETFPM 6tqf.1    --------------------------------------------------------------------------------  target    TFIVEHRRLITISNTKNAYVIEQMTRYLENHDTLSIYKFLFASLEIISNAYYPVIEQMDKSRDEVNDLLRQRTTKKNLFV 6tqf.1    --------------------------------------------------------------------------------  target    LSDLETGMVYLTAAAKQNRILLEHIQGHALYRSFDEIEREQFDDAMIEAHQLVSMTDLISQILQQLSASYNNILNNNLND 6tqf.1    --------------------------------------------------------------------------------  target    NLTTLTIISVLLAVLAVVTGFFGMNVPLPLTDEPHAWLYISLASAGLWIVLSLLLRKIAKKS 6tqf.1    ----FGAVQSIISVISFTAILWNLSGT----------------------------------- ``` | | | | | | | | | | | | | | | | | | | | | | | | | | | | | | | | | | | | | | | | | | | | | | | | | |
|  | 2dqs.1.A | Sarcoplasmic/endoplasmic reticulum calcium ATPase 1  *Crystal structure of the calcium pump with amppcp in the absence of calcium* | 0.01 |  | 26.09 | 0.08 | 278-300 | X-ray | 2.50 | monomer | 1 x MG, 1 x ACP, 1 x TG1, 2 x PTY | HHblits | 0.28 |
| ``` target    MVLEKQLGNGCTWIDLDLGKLNKLEDLSEIYGLDKETIEYALDRNERAHMDYHRESETVTFIYNVLDVKKDKAYYETFPM 2dqs.1    --------------------------------------------------------------------------------  target    TFIVEHRRLITISNTKNAYVIEQMTRYLENHDTLSIYKFLFASLEIISNAYYPVIEQMDKSRDEVNDLLRQRTTKKNLFV 2dqs.1    --------------------------------------------------------------------------------  target    LSDLETGMVYLTAAAKQNRILLEHIQGHALYRSFDEIEREQFDDAMIEAHQLVSMTDLISQILQQLSASYNNILNNNLND 2dqs.1    --------------------------------------------------------------------------------  target    NLTTLTIISVLLAVLAVVTGFFGMNVPLPLTDEPHAWLYISLASAGLWIVLSLLLRKIAKKS 2dqs.1    -------------------------------------WLMVLKISLPVIGLDEILKFIAR-- ``` | | | | | | | | | | | | | | | | | | | | | | | | | | | | | | | | | | | | | | | | | | | | | | | | | |
|  | 2zbg.1.A | Sarcoplasmic/endoplasmic reticulum calcium ATPase 1  *Calcium pump crystal structure with bound AlF4 and TG in the absence of calcium* | 0.00 |  | 26.09 | 0.08 | 278-300 | X-ray | 2.55 | monomer | 1 x MG, 1 x ALF, 1 x TG1 | HHblits | 0.28 |
| ``` target    MVLEKQLGNGCTWIDLDLGKLNKLEDLSEIYGLDKETIEYALDRNERAHMDYHRESETVTFIYNVLDVKKDKAYYETFPM 2zbg.1    --------------------------------------------------------------------------------  target    TFIVEHRRLITISNTKNAYVIEQMTRYLENHDTLSIYKFLFASLEIISNAYYPVIEQMDKSRDEVNDLLRQRTTKKNLFV 2zbg.1    --------------------------------------------------------------------------------  target    LSDLETGMVYLTAAAKQNRILLEHIQGHALYRSFDEIEREQFDDAMIEAHQLVSMTDLISQILQQLSASYNNILNNNLND 2zbg.1    --------------------------------------------------------------------------------  target    NLTTLTIISVLLAVLAVVTGFFGMNVPLPLTDEPHAWLYISLASAGLWIVLSLLLRKIAKKS 2zbg.1    -------------------------------------WLMVLKISLPVIGLDEILKFIAR-- ``` | | | | | | | | | | | | | | | | | | | | | | | | | | | | | | | | | | | | | | | | | | | | | | | | | |
|  | 2zbf.1.A | Sarcoplasmic/endoplasmic reticulum calcium ATPase 1  *Calcium pump crystal structure with bound BeF3 and TG in the absence of calcium* | 0.01 |  | 26.09 | 0.08 | 278-300 | X-ray | 2.40 | monomer | 1 x MG, 1 x BEF, 1 x TG1 | HHblits | 0.28 |
| ``` target    MVLEKQLGNGCTWIDLDLGKLNKLEDLSEIYGLDKETIEYALDRNERAHMDYHRESETVTFIYNVLDVKKDKAYYETFPM 2zbf.1    --------------------------------------------------------------------------------  target    TFIVEHRRLITISNTKNAYVIEQMTRYLENHDTLSIYKFLFASLEIISNAYYPVIEQMDKSRDEVNDLLRQRTTKKNLFV 2zbf.1    --------------------------------------------------------------------------------  target    LSDLETGMVYLTAAAKQNRILLEHIQGHALYRSFDEIEREQFDDAMIEAHQLVSMTDLISQILQQLSASYNNILNNNLND 2zbf.1    --------------------------------------------------------------------------------  target    NLTTLTIISVLLAVLAVVTGFFGMNVPLPLTDEPHAWLYISLASAGLWIVLSLLLRKIAKKS 2zbf.1    -------------------------------------WLMVLKISLPVIGLDEILKFIAR-- ``` | | | | | | | | | | | | | | | | | | | | | | | | | | | | | | | | | | | | | | | | | | | | | | | | | |
|  | 2zbd.1.A | Sarcoplasmic/endoplasmic reticulum calcium ATPase 1  *Crystal Structure of the SR Calcium Pump with Bound Aluminium Fluoride, ADP and Calcium* | 0.01 |  | 26.09 | 0.08 | 278-300 | X-ray | 2.40 | monomer | 2 x CA, 1 x ALF, 1 x MG, 1 x ADP, 2 x PC1 | HHblits | 0.28 |
| ``` target    MVLEKQLGNGCTWIDLDLGKLNKLEDLSEIYGLDKETIEYALDRNERAHMDYHRESETVTFIYNVLDVKKDKAYYETFPM 2zbd.1    --------------------------------------------------------------------------------  target    TFIVEHRRLITISNTKNAYVIEQMTRYLENHDTLSIYKFLFASLEIISNAYYPVIEQMDKSRDEVNDLLRQRTTKKNLFV 2zbd.1    --------------------------------------------------------------------------------  target    LSDLETGMVYLTAAAKQNRILLEHIQGHALYRSFDEIEREQFDDAMIEAHQLVSMTDLISQILQQLSASYNNILNNNLND 2zbd.1    --------------------------------------------------------------------------------  target    NLTTLTIISVLLAVLAVVTGFFGMNVPLPLTDEPHAWLYISLASAGLWIVLSLLLRKIAKKS 2zbd.1    -------------------------------------WLMVLKISLPVIGLDEILKFIAR-- ``` | | | | | | | | | | | | | | | | | | | | | | | | | | | | | | | | | | | | | | | | | | | | | | | | | |
|  | 2zbe.1.A | Sarcoplasmic/endoplasmic reticulum calcium ATPase 1  *Calcium pump crystal structure with bound BeF3 in the absence of calcium and TG* | 0.01 |  | 26.09 | 0.08 | 278-300 | X-ray | 3.80 | monomer | 1 x MG, 1 x BEF | HHblits | 0.28 |
| ``` target    MVLEKQLGNGCTWIDLDLGKLNKLEDLSEIYGLDKETIEYALDRNERAHMDYHRESETVTFIYNVLDVKKDKAYYETFPM 2zbe.1    --------------------------------------------------------------------------------  target    TFIVEHRRLITISNTKNAYVIEQMTRYLENHDTLSIYKFLFASLEIISNAYYPVIEQMDKSRDEVNDLLRQRTTKKNLFV 2zbe.1    --------------------------------------------------------------------------------  target    LSDLETGMVYLTAAAKQNRILLEHIQGHALYRSFDEIEREQFDDAMIEAHQLVSMTDLISQILQQLSASYNNILNNNLND 2zbe.1    --------------------------------------------------------------------------------  target    NLTTLTIISVLLAVLAVVTGFFGMNVPLPLTDEPHAWLYISLASAGLWIVLSLLLRKIAKKS 2zbe.1    -------------------------------------WLMVLKISLPVIGLDEILKFIAR-- ``` | | | | | | | | | | | | | | | | | | | | | | | | | | | | | | | | | | | | | | | | | | | | | | | | | |
|  | 2eat.1.A | Sarcoplasmic/endoplasmic reticulum calcium ATPase 1  *Crystal structure of the SR CA2+-ATPASE with bound CPA and TG* | 0.00 |  | 26.09 | 0.08 | 278-300 | X-ray | 2.90 | monomer | 1 x TG1, 1 x CZA | HHblits | 0.28 |
| ``` target    MVLEKQLGNGCTWIDLDLGKLNKLEDLSEIYGLDKETIEYALDRNERAHMDYHRESETVTFIYNVLDVKKDKAYYETFPM 2eat.1    --------------------------------------------------------------------------------  target    TFIVEHRRLITISNTKNAYVIEQMTRYLENHDTLSIYKFLFASLEIISNAYYPVIEQMDKSRDEVNDLLRQRTTKKNLFV 2eat.1    --------------------------------------------------------------------------------  target    LSDLETGMVYLTAAAKQNRILLEHIQGHALYRSFDEIEREQFDDAMIEAHQLVSMTDLISQILQQLSASYNNILNNNLND 2eat.1    --------------------------------------------------------------------------------  target    NLTTLTIISVLLAVLAVVTGFFGMNVPLPLTDEPHAWLYISLASAGLWIVLSLLLRKIAKKS 2eat.1    -------------------------------------WLMVLKISLPVIGLDEILKFIAR-- ``` | | | | | | | | | | | | | | | | | | | | | | | | | | | | | | | | | | | | | | | | | | | | | | | | | |
|  | 2ear.1.A | Sarcoplasmic/endoplasmic reticulum calcium ATPase 1  *P21 crystal of the SR CA2+-ATPase with bound TG* | 0.00 |  | 26.09 | 0.08 | 278-300 | X-ray | 3.10 | monomer | 1 x TG1 | HHblits | 0.28 |
| ``` target    MVLEKQLGNGCTWIDLDLGKLNKLEDLSEIYGLDKETIEYALDRNERAHMDYHRESETVTFIYNVLDVKKDKAYYETFPM 2ear.1    --------------------------------------------------------------------------------  target    TFIVEHRRLITISNTKNAYVIEQMTRYLENHDTLSIYKFLFASLEIISNAYYPVIEQMDKSRDEVNDLLRQRTTKKNLFV 2ear.1    --------------------------------------------------------------------------------  target    LSDLETGMVYLTAAAKQNRILLEHIQGHALYRSFDEIEREQFDDAMIEAHQLVSMTDLISQILQQLSASYNNILNNNLND 2ear.1    --------------------------------------------------------------------------------  target    NLTTLTIISVLLAVLAVVTGFFGMNVPLPLTDEPHAWLYISLASAGLWIVLSLLLRKIAKKS 2ear.1    -------------------------------------WLMVLKISLPVIGLDEILKFIAR-- ``` | | | | | | | | | | | | | | | | | | | | | | | | | | | | | | | | | | | | | | | | | | | | | | | | | |
|  | 2eau.1.A | Sarcoplasmic/endoplasmic reticulum calcium ATPase 1  *Crystal structure of the SR CA2+-ATPASE with bound CPA in the presence of curcumin* | 0.00 |  | 26.09 | 0.08 | 278-300 | X-ray | 2.80 | monomer | 1 x CZA, 3 x PTY | HHblits | 0.28 |
| ``` target    MVLEKQLGNGCTWIDLDLGKLNKLEDLSEIYGLDKETIEYALDRNERAHMDYHRESETVTFIYNVLDVKKDKAYYETFPM 2eau.1    --------------------------------------------------------------------------------  target    TFIVEHRRLITISNTKNAYVIEQMTRYLENHDTLSIYKFLFASLEIISNAYYPVIEQMDKSRDEVNDLLRQRTTKKNLFV 2eau.1    --------------------------------------------------------------------------------  target    LSDLETGMVYLTAAAKQNRILLEHIQGHALYRSFDEIEREQFDDAMIEAHQLVSMTDLISQILQQLSASYNNILNNNLND 2eau.1    --------------------------------------------------------------------------------  target    NLTTLTIISVLLAVLAVVTGFFGMNVPLPLTDEPHAWLYISLASAGLWIVLSLLLRKIAKKS 2eau.1    -------------------------------------WLMVLKISLPVIGLDEILKFIAR-- ``` | | | | | | | | | | | | | | | | | | | | | | | | | | | | | | | | | | | | | | | | | | | | | | | | | |
|  | 3w5a.1.A | SERCA1a  *Crystal structure of the calcium pump and sarcolipin from rabbit fast twitch skeletal muscle in the E1.Mg2+ state* | 0.01 |  | 26.09 | 0.08 | 278-300 | X-ray | 3.01 | hetero-oligomer | 2 x TM1, 5 x PTY, 2 x MG | HHblits | 0.28 |
| ``` target    MVLEKQLGNGCTWIDLDLGKLNKLEDLSEIYGLDKETIEYALDRNERAHMDYHRESETVTFIYNVLDVKKDKAYYETFPM 3w5a.1    --------------------------------------------------------------------------------  target    TFIVEHRRLITISNTKNAYVIEQMTRYLENHDTLSIYKFLFASLEIISNAYYPVIEQMDKSRDEVNDLLRQRTTKKNLFV 3w5a.1    --------------------------------------------------------------------------------  target    LSDLETGMVYLTAAAKQNRILLEHIQGHALYRSFDEIEREQFDDAMIEAHQLVSMTDLISQILQQLSASYNNILNNNLND 3w5a.1    --------------------------------------------------------------------------------  target    NLTTLTIISVLLAVLAVVTGFFGMNVPLPLTDEPHAWLYISLASAGLWIVLSLLLRKIAKKS 3w5a.1    -------------------------------------WLMVLKISLPVIGLDEILKFIAR-- ``` | | | | | | | | | | | | | | | | | | | | | | | | | | | | | | | | | | | | | | | | | | | | | | | | | |
|  | 3ar2.1.A | Sarcoplasmic/endoplasmic reticulum calcium ATPase 1  *Calcium pump crystal structure with bound AMPPCP and Ca2+* | 0.01 |  | 26.09 | 0.08 | 278-300 | X-ray | 2.50 | monomer | 3 x CA, 1 x ACP, 1 x PC1 | HHblits | 0.28 |
| ``` target    MVLEKQLGNGCTWIDLDLGKLNKLEDLSEIYGLDKETIEYALDRNERAHMDYHRESETVTFIYNVLDVKKDKAYYETFPM 3ar2.1    --------------------------------------------------------------------------------  target    TFIVEHRRLITISNTKNAYVIEQMTRYLENHDTLSIYKFLFASLEIISNAYYPVIEQMDKSRDEVNDLLRQRTTKKNLFV 3ar2.1    --------------------------------------------------------------------------------  target    LSDLETGMVYLTAAAKQNRILLEHIQGHALYRSFDEIEREQFDDAMIEAHQLVSMTDLISQILQQLSASYNNILNNNLND 3ar2.1    --------------------------------------------------------------------------------  target    NLTTLTIISVLLAVLAVVTGFFGMNVPLPLTDEPHAWLYISLASAGLWIVLSLLLRKIAKKS 3ar2.1    -------------------------------------WLMVLKISLPVIGLDEILKFIAR-- ``` | | | | | | | | | | | | | | | | | | | | | | | | | | | | | | | | | | | | | | | | | | | | | | | | | |
|  | 4uu0.1.A | SERCA1A  *CRYSTAL STRUCTURE OF (SR) CALCIUM-ATPASE E2(TG) IN THE PRESENCE OF 14:1 PC* | 0.00 |  | 26.09 | 0.08 | 278-300 | X-ray | 2.50 | monomer | 1 x TG1, 1 x K, 1 x MG | HHblits | 0.28 |
| ``` target    MVLEKQLGNGCTWIDLDLGKLNKLEDLSEIYGLDKETIEYALDRNERAHMDYHRESETVTFIYNVLDVKKDKAYYETFPM 4uu0.1    --------------------------------------------------------------------------------  target    TFIVEHRRLITISNTKNAYVIEQMTRYLENHDTLSIYKFLFASLEIISNAYYPVIEQMDKSRDEVNDLLRQRTTKKNLFV 4uu0.1    --------------------------------------------------------------------------------  target    LSDLETGMVYLTAAAKQNRILLEHIQGHALYRSFDEIEREQFDDAMIEAHQLVSMTDLISQILQQLSASYNNILNNNLND 4uu0.1    --------------------------------------------------------------------------------  target    NLTTLTIISVLLAVLAVVTGFFGMNVPLPLTDEPHAWLYISLASAGLWIVLSLLLRKIAKKS 4uu0.1    -------------------------------------WLMVLKISLPVIGLDEILKFIAR-- ``` | | | | | | | | | | | | | | | | | | | | | | | | | | | | | | | | | | | | | | | | | | | | | | | | | |
|  | 4uu1.1.A | SARCOPLASMIC ENDOPLASMIC RETICULUM CALCIUM ATPASE  *CRYSTAL STRUCTURE OF (SR) CALCIUM-ATPASE E2(TG) IN THE PRESENCE OF DOPC* | 0.01 |  | 26.09 | 0.08 | 278-300 | X-ray | 2.80 | monomer | 1 x TG1, 3 x PCW, 1 x ACP, 1 x K, 1 x MG | HHblits | 0.28 |
| ``` target    MVLEKQLGNGCTWIDLDLGKLNKLEDLSEIYGLDKETIEYALDRNERAHMDYHRESETVTFIYNVLDVKKDKAYYETFPM 4uu1.1    --------------------------------------------------------------------------------  target    TFIVEHRRLITISNTKNAYVIEQMTRYLENHDTLSIYKFLFASLEIISNAYYPVIEQMDKSRDEVNDLLRQRTTKKNLFV 4uu1.1    --------------------------------------------------------------------------------  target    LSDLETGMVYLTAAAKQNRILLEHIQGHALYRSFDEIEREQFDDAMIEAHQLVSMTDLISQILQQLSASYNNILNNNLND 4uu1.1    --------------------------------------------------------------------------------  target    NLTTLTIISVLLAVLAVVTGFFGMNVPLPLTDEPHAWLYISLASAGLWIVLSLLLRKIAKKS 4uu1.1    -------------------------------------WLMVLKISLPVIGLDEILKFIAR-- ``` | | | | | | | | | | | | | | | | | | | | | | | | | | | | | | | | | | | | | | | | | | | | | | | | | |
|  | 5xaa.1.A | Sarcoplasmic/endoplasmic reticulum calcium ATPase 1  *Complete structure factors and an atomic model of the calcium pump (SERCA1A) and associated phospholipids in the E2-ALF-(TG) crystals of P21212 symmetry* | 0.00 |  | 26.09 | 0.08 | 278-300 | X-ray | 3.20 | monomer | 1 x MG, 1 x ALF, 1 x TG1, 26 x PCW | HHblits | 0.28 |
| ``` target    MVLEKQLGNGCTWIDLDLGKLNKLEDLSEIYGLDKETIEYALDRNERAHMDYHRESETVTFIYNVLDVKKDKAYYETFPM 5xaa.1    --------------------------------------------------------------------------------  target    TFIVEHRRLITISNTKNAYVIEQMTRYLENHDTLSIYKFLFASLEIISNAYYPVIEQMDKSRDEVNDLLRQRTTKKNLFV 5xaa.1    --------------------------------------------------------------------------------  target    LSDLETGMVYLTAAAKQNRILLEHIQGHALYRSFDEIEREQFDDAMIEAHQLVSMTDLISQILQQLSASYNNILNNNLND 5xaa.1    --------------------------------------------------------------------------------  target    NLTTLTIISVLLAVLAVVTGFFGMNVPLPLTDEPHAWLYISLASAGLWIVLSLLLRKIAKKS 5xaa.1    -------------------------------------WLMVLKISLPVIGLDEILKFIAR-- ``` | | | | | | | | | | | | | | | | | | | | | | | | | | | | | | | | | | | | | | | | | | | | | | | | | |
|  | 5xab.1.A | Sarcoplasmic/endoplasmic reticulum calcium ATPase 1  *Complete structure factors and an atomic model of the calcium pump (SERCA1A) and associated phospholipids in the E2(TG) crystals* | 0.01 |  | 26.09 | 0.08 | 278-300 | X-ray | 3.20 | monomer | 1 x TG1, 25 x PCW | HHblits | 0.28 |
| ``` target    MVLEKQLGNGCTWIDLDLGKLNKLEDLSEIYGLDKETIEYALDRNERAHMDYHRESETVTFIYNVLDVKKDKAYYETFPM 5xab.1    --------------------------------------------------------------------------------  target    TFIVEHRRLITISNTKNAYVIEQMTRYLENHDTLSIYKFLFASLEIISNAYYPVIEQMDKSRDEVNDLLRQRTTKKNLFV 5xab.1    --------------------------------------------------------------------------------  target    LSDLETGMVYLTAAAKQNRILLEHIQGHALYRSFDEIEREQFDDAMIEAHQLVSMTDLISQILQQLSASYNNILNNNLND 5xab.1    --------------------------------------------------------------------------------  target    NLTTLTIISVLLAVLAVVTGFFGMNVPLPLTDEPHAWLYISLASAGLWIVLSLLLRKIAKKS 5xab.1    -------------------------------------WLMVLKISLPVIGLDEILKFIAR-- ``` | | | | | | | | | | | | | | | | | | | | | | | | | | | | | | | | | | | | | | | | | | | | | | | | | |
|  | 5xa7.1.A | Sarcoplasmic/endoplasmic reticulum calcium ATPase 1  *Complete structure factors and an atomic model of the calcium pump (SERCA1A) and associated phospholipids in the E1-2CA2+ crystals* | 0.01 |  | 26.09 | 0.08 | 278-300 | X-ray | 3.20 | monomer | 2 x CA, 19 x PCW | HHblits | 0.28 |
| ``` target    MVLEKQLGNGCTWIDLDLGKLNKLEDLSEIYGLDKETIEYALDRNERAHMDYHRESETVTFIYNVLDVKKDKAYYETFPM 5xa7.1    --------------------------------------------------------------------------------  target    TFIVEHRRLITISNTKNAYVIEQMTRYLENHDTLSIYKFLFASLEIISNAYYPVIEQMDKSRDEVNDLLRQRTTKKNLFV 5xa7.1    --------------------------------------------------------------------------------  target    LSDLETGMVYLTAAAKQNRILLEHIQGHALYRSFDEIEREQFDDAMIEAHQLVSMTDLISQILQQLSASYNNILNNNLND 5xa7.1    --------------------------------------------------------------------------------  target    NLTTLTIISVLLAVLAVVTGFFGMNVPLPLTDEPHAWLYISLASAGLWIVLSLLLRKIAKKS 5xa7.1    -------------------------------------WLMVLKISLPVIGLDEILKFIAR-- ``` | | | | | | | | | | | | | | | | | | | | | | | | | | | | | | | | | | | | | | | | | | | | | | | | | |
|  | 5a3s.2.A | SARCOPLASMIC RETICULUM CALCIUM ATPASE 1 MOLECULE SARCOPLASMIC/ENDOPLASMIC RETICULUM CALCIUM ATPASE 1  *Crystal structure of the (SR) Calcium ATPase E2-vanadate complex bound to thapsigargin and TNP-ATP* | 0.00 |  | 26.09 | 0.08 | 278-300 | X-ray | 3.30 | monomer | 1 x TG1, 1 x VN4, 1 x 128, 2 x MG, 1 x K | HHblits | 0.28 |
| ``` target    MVLEKQLGNGCTWIDLDLGKLNKLEDLSEIYGLDKETIEYALDRNERAHMDYHRESETVTFIYNVLDVKKDKAYYETFPM 5a3s.2    --------------------------------------------------------------------------------  target    TFIVEHRRLITISNTKNAYVIEQMTRYLENHDTLSIYKFLFASLEIISNAYYPVIEQMDKSRDEVNDLLRQRTTKKNLFV 5a3s.2    --------------------------------------------------------------------------------  target    LSDLETGMVYLTAAAKQNRILLEHIQGHALYRSFDEIEREQFDDAMIEAHQLVSMTDLISQILQQLSASYNNILNNNLND 5a3s.2    --------------------------------------------------------------------------------  target    NLTTLTIISVLLAVLAVVTGFFGMNVPLPLTDEPHAWLYISLASAGLWIVLSLLLRKIAKKS 5a3s.2    -------------------------------------WLMVLKISLPVIGLDEILKFIAR-- ``` | | | | | | | | | | | | | | | | | | | | | | | | | | | | | | | | | | | | | | | | | | | | | | | | | |
|  | 5a3s.1.A | SARCOPLASMIC RETICULUM CALCIUM ATPASE 1 MOLECULE SARCOPLASMIC/ENDOPLASMIC RETICULUM CALCIUM ATPASE 1  *Crystal structure of the (SR) Calcium ATPase E2-vanadate complex bound to thapsigargin and TNP-ATP* | 0.00 |  | 26.09 | 0.08 | 278-300 | X-ray | 3.30 | monomer | 1 x TG1, 1 x VN4, 1 x 128, 2 x MG, 1 x K | HHblits | 0.28 |
| ``` target    MVLEKQLGNGCTWIDLDLGKLNKLEDLSEIYGLDKETIEYALDRNERAHMDYHRESETVTFIYNVLDVKKDKAYYETFPM 5a3s.1    --------------------------------------------------------------------------------  target    TFIVEHRRLITISNTKNAYVIEQMTRYLENHDTLSIYKFLFASLEIISNAYYPVIEQMDKSRDEVNDLLRQRTTKKNLFV 5a3s.1    --------------------------------------------------------------------------------  target    LSDLETGMVYLTAAAKQNRILLEHIQGHALYRSFDEIEREQFDDAMIEAHQLVSMTDLISQILQQLSASYNNILNNNLND 5a3s.1    --------------------------------------------------------------------------------  target    NLTTLTIISVLLAVLAVVTGFFGMNVPLPLTDEPHAWLYISLASAGLWIVLSLLLRKIAKKS 5a3s.1    -------------------------------------WLMVLKISLPVIGLDEILKFIAR-- ``` | | | | | | | | | | | | | | | | | | | | | | | | | | | | | | | | | | | | | | | | | | | | | | | | | |
|  | 5a3q.1.A | SARCOPLASMIC/ENDOPLASMIC RETICULUM CALCIUM ATPASE 1  *Crystal structure of the (SR) Calcium ATPase E2-vanadate complex bound to thapsigargin and TNP-AMPPCP* | 0.00 |  | 26.09 | 0.08 | 278-300 | X-ray | 3.05 | monomer | 1 x TG1, 1 x VN4, 1 x DL5, 2 x MG, 1 x K | HHblits | 0.28 |
| ``` target    MVLEKQLGNGCTWIDLDLGKLNKLEDLSEIYGLDKETIEYALDRNERAHMDYHRESETVTFIYNVLDVKKDKAYYETFPM 5a3q.1    --------------------------------------------------------------------------------  target    TFIVEHRRLITISNTKNAYVIEQMTRYLENHDTLSIYKFLFASLEIISNAYYPVIEQMDKSRDEVNDLLRQRTTKKNLFV 5a3q.1    --------------------------------------------------------------------------------  target    LSDLETGMVYLTAAAKQNRILLEHIQGHALYRSFDEIEREQFDDAMIEAHQLVSMTDLISQILQQLSASYNNILNNNLND 5a3q.1    --------------------------------------------------------------------------------  target    NLTTLTIISVLLAVLAVVTGFFGMNVPLPLTDEPHAWLYISLASAGLWIVLSLLLRKIAKKS 5a3q.1    -------------------------------------WLMVLKISLPVIGLDEILKFIAR-- ``` | | | | | | | | | | | | | | | | | | | | | | | | | | | | | | | | | | | | | | | | | | | | | | | | | |
|  | 4ycl.1.A | Sarcoplasmic/endoplasmic reticulum calcium ATPase 1  *Crystal structure of the SR CA2+-ATPASE with bound CPA* | 0.01 |  | 26.09 | 0.08 | 278-300 | X-ray | 3.25 | monomer | 1 x K, 1 x MG, 1 x CZA | HHblits | 0.28 |
| ``` target    MVLEKQLGNGCTWIDLDLGKLNKLEDLSEIYGLDKETIEYALDRNERAHMDYHRESETVTFIYNVLDVKKDKAYYETFPM 4ycl.1    --------------------------------------------------------------------------------  target    TFIVEHRRLITISNTKNAYVIEQMTRYLENHDTLSIYKFLFASLEIISNAYYPVIEQMDKSRDEVNDLLRQRTTKKNLFV 4ycl.1    --------------------------------------------------------------------------------  target    LSDLETGMVYLTAAAKQNRILLEHIQGHALYRSFDEIEREQFDDAMIEAHQLVSMTDLISQILQQLSASYNNILNNNLND 4ycl.1    --------------------------------------------------------------------------------  target    NLTTLTIISVLLAVLAVVTGFFGMNVPLPLTDEPHAWLYISLASAGLWIVLSLLLRKIAKKS 4ycl.1    -------------------------------------WLMVLKISLPVIGLDEILKFIAR-- ``` | | | | | | | | | | | | | | | | | | | | | | | | | | | | | | | | | | | | | | | | | | | | | | | | | |
|  | 1quu.1.A | HUMAN SKELETAL MUSCLE ALPHA-ACTININ 2  *CRYSTAL STRUCTURE OF TWO CENTRAL SPECTRIN-LIKE REPEATS FROM ALPHA-ACTININ* | 0.01 |  | 8.33 | 0.08 | 128-151 | X-ray | 2.50 | homo-dimer |  | HHblits | 0.25 |
| ``` target    MVLEKQLGNGCTWIDLDLGKLNKLEDLSEIYGLDKETIEYALDRNERAHMDYHRESETVTFIYNVLDVKKDKAYYETFPM 1quu.1    --------------------------------------------------------------------------------  target    TFIVEHRRLITISNTKNAYVIEQMTRYLENHDTLSIYKFLFASLEIISNAYYPVIEQMDKSRDEVNDLLRQRTTKKNLFV 1quu.1    -----------------------------------------------AEKFRQKASTHETWAYGKEQILLQ---------  target    LSDLETGMVYLTAAAKQNRILLEHIQGHALYRSFDEIEREQFDDAMIEAHQLVSMTDLISQILQQLSASYNNILNNNLND 1quu.1    --------------------------------------------------------------------------------  target    NLTTLTIISVLLAVLAVVTGFFGMNVPLPLTDEPHAWLYISLASAGLWIVLSLLLRKIAKKS 1quu.1    -------------------------------------------------------------- ``` | | | | | | | | | | | | | | | | | | | | | | | | | | | | | | | | | | | | | | | | | | | | | | | | | |
|  | 7lep.1.G | Voltage-dependent calcium channel gamma-8 subunit  *The composite LBD-TMD structure combined from all hippocampal AMPAR subtypes at 3.25 Angstrom resolution* | 0.00 |  | 4.17 | 0.08 | 271-294 | EM | 0.00 | hetero-1-2-1-2-2-mer | 4 x ZK1, 30 x POV, 1 x C14, 1 x D10, 2 x XVD, 2 x OCT, 2 x D12 | HHblits | 0.24 |
| ``` target    MVLEKQLGNGCTWIDLDLGKLNKLEDLSEIYGLDKETIEYALDRNERAHMDYHRESETVTFIYNVLDVKKDKAYYETFPM 7lep.1    --------------------------------------------------------------------------------  target    TFIVEHRRLITISNTKNAYVIEQMTRYLENHDTLSIYKFLFASLEIISNAYYPVIEQMDKSRDEVNDLLRQRTTKKNLFV 7lep.1    --------------------------------------------------------------------------------  target    LSDLETGMVYLTAAAKQNRILLEHIQGHALYRSFDEIEREQFDDAMIEAHQLVSMTDLISQILQQLSASYNNILNNNLND 7lep.1    --------------------------------------------------------------------------------  target    NLTTLTIISVLLAVLAVVTGFFGMNVPLPLTDEPHAWLYISLASAGLWIVLSLLLRKIAKKS 7lep.1    ------------------------------NHYSYGWSFYFGGLSFILAEVIGV-------- ``` | | | | | | | | | | | | | | | | | | | | | | | | | | | | | | | | | | | | | | | | | | | | | | | | | |
|  | 5oqm.1.d | Mediator of RNA polymerase II transcription subunit 7  *STRUCTURE OF YEAST TRANSCRIPTION PRE-INITIATION COMPLEX WITH TFIIH AND CORE MEDIATOR* | 0.00 |  | 17.39 | 0.08 | 120-142 | EM | 0.00 | hetero-1-1-1-1-1-1-… | 16 x ZN, 1 x MG, 1 x SF4 | HHblits | 0.27 |
| ``` target    MVLEKQLGNGCTWIDLDLGKLNKLEDLSEIYGLDKETIEYALDRNERAHMDYHRESETVTFIYNVLDVKKDKAYYETFPM 5oqm.1    --------------------------------------------------------------------------------  target    TFIVEHRRLITISNTKNAYVIEQMTRYLENHDTLSIYKFLFASLEIISNAYYPVIEQMDKSRDEVNDLLRQRTTKKNLFV 5oqm.1    ---------------------------------------LRKLLKSLLLNYLELIGVLSINP------------------  target    LSDLETGMVYLTAAAKQNRILLEHIQGHALYRSFDEIEREQFDDAMIEAHQLVSMTDLISQILQQLSASYNNILNNNLND 5oqm.1    --------------------------------------------------------------------------------  target    NLTTLTIISVLLAVLAVVTGFFGMNVPLPLTDEPHAWLYISLASAGLWIVLSLLLRKIAKKS 5oqm.1    -------------------------------------------------------------- ``` | | | | | | | | | | | | | | | | | | | | | | | | | | | | | | | | | | | | | | | | | | | | | | | | | |
|  | 6s7o.1.F | Dolichyl-diphosphooligosaccharide--protein glycosyltransferase subunit 2  *Cryo-EM structure of human oligosaccharyltransferase complex OST-A* | 0.00 |  | 23.81 | 0.07 | 251-271 | EM | 0.00 | hetero-1-1-1-1-1-1-… | 9 x KZB, 7 x EGY, 2 x MG, 1 x KZE, 1 x NAG-NAG-BMA, 2 x NAG-NAG-BMA-MAN-MAN-MAN-MAN-MAN | HHblits | 0.34 |
| ``` target    MVLEKQLGNGCTWIDLDLGKLNKLEDLSEIYGLDKETIEYALDRNERAHMDYHRESETVTFIYNVLDVKKDKAYYETFPM 6s7o.1    --------------------------------------------------------------------------------  target    TFIVEHRRLITISNTKNAYVIEQMTRYLENHDTLSIYKFLFASLEIISNAYYPVIEQMDKSRDEVNDLLRQRTTKKNLFV 6s7o.1    --------------------------------------------------------------------------------  target    LSDLETGMVYLTAAAKQNRILLEHIQGHALYRSFDEIEREQFDDAMIEAHQLVSMTDLISQILQQLSASYNNILNNNLND 6s7o.1    --------------------------------------------------------------------------------  target    NLTTLTIISVLLAVLAVVTGFF---GMNVP-LPLTDEPHAWLYISLASAGLWIVLSLLLRKIAKKS 6s7o.1    ----------ILSPLLLLFALWIRIGANVSNFTFA------------------------------- ``` | | | | | | | | | | | | | | | | | | | | | | | | | | | | | | | | | | | | | | | | | | | | | | | | | |
|  | 6s7t.1.F | Dolichyl-diphosphooligosaccharide--protein glycosyltransferase subunit 2  *Cryo-EM structure of human oligosaccharyltransferase complex OST-B* | 0.00 |  | 23.81 | 0.07 | 251-271 | EM | 0.00 | hetero-1-1-1-1-1-1-… | 10 x EGY, 13 x KZB, 2 x MG, 1 x 0K3, 1 x ALA-ALA-ASN-ALA-THR-ALA-ALA, 2 x NAG-NAG, 2 x NAG-NAG-BMA-MAN-MAN-MAN-MAN-MAN | HHblits | 0.34 |
| ``` target    MVLEKQLGNGCTWIDLDLGKLNKLEDLSEIYGLDKETIEYALDRNERAHMDYHRESETVTFIYNVLDVKKDKAYYETFPM 6s7t.1    --------------------------------------------------------------------------------  target    TFIVEHRRLITISNTKNAYVIEQMTRYLENHDTLSIYKFLFASLEIISNAYYPVIEQMDKSRDEVNDLLRQRTTKKNLFV 6s7t.1    --------------------------------------------------------------------------------  target    LSDLETGMVYLTAAAKQNRILLEHIQGHALYRSFDEIEREQFDDAMIEAHQLVSMTDLISQILQQLSASYNNILNNNLND 6s7t.1    --------------------------------------------------------------------------------  target    NLTTLTIISVLLAVLAVVTGFF---GMNVP-LPLTDEPHAWLYISLASAGLWIVLSLLLRKIAKKS 6s7t.1    ----------ILSPLLLLFALWIRIGANVSNFTFA------------------------------- ``` | | | | | | | | | | | | | | | | | | | | | | | | | | | | | | | | | | | | | | | | | | | | | | | | | |
|  | 6qkc.1.E | Voltage-dependent calcium channel gamma-8 subunit  *GluA1/2 In complex with auxiliary subunit gamma-8* | 0.00 |  | 8.70 | 0.08 | 273-295 | EM | 4.10 | hetero-2-2-2-mer | 4 x E2Q, 7 x OLC | HHblits | 0.26 |
| ``` target    MVLEKQLGNGCTWIDLDLGKLNKLEDLSEIYGLDKETIEYALDRNERAHMDYHRESETVTFIYNVLDVKKDKAYYETFPM 6qkc.1    --------------------------------------------------------------------------------  target    TFIVEHRRLITISNTKNAYVIEQMTRYLENHDTLSIYKFLFASLEIISNAYYPVIEQMDKSRDEVNDLLRQRTTKKNLFV 6qkc.1    --------------------------------------------------------------------------------  target    LSDLETGMVYLTAAAKQNRILLEHIQGHALYRSFDEIEREQFDDAMIEAHQLVSMTDLISQILQQLSASYNNILNNNLND 6qkc.1    --------------------------------------------------------------------------------  target    NLTTLTIISVLLAVLAVVTGFFGMNVPLPLTDEPHAWLYISLASAGLWIVLSLLLRKIAKKS 6qkc.1    --------------------------------YSYGWSFYFGGLSFILAEVIGVL------- ``` | | | | | | | | | | | | | | | | | | | | | | | | | | | | | | | | | | | | | | | | | | | | | | | | | |
|  | 6r7q.79.A | Protein transport protein Sec61 subunit alpha isoform 1  *Structure of XBP1u-paused ribosome nascent chain complex with Sec61.* | 0.01 |  | 13.04 | 0.08 | 236-258 | EM | 0.00 | monomer |  | HHblits | 0.25 |
| ``` target    MVLEKQLGNGCTWIDLDLGKLNKLEDLSEIYGLDKETIEYALDRNERAHMDYHRESETVTFIYNVLDVKKDKAYYETFPM 6r7q.79   --------------------------------------------------------------------------------  target    TFIVEHRRLITISNTKNAYVIEQMTRYLENHDTLSIYKFLFASLEIISNAYYPVIEQMDKSRDEVNDLLRQRTTKKNLFV 6r7q.79   --------------------------------------------------------------------------------  target    LSDLETGMVYLTAAAKQNRILLEHIQGHALYRSFDEIEREQFDDAMIEAHQLVSMTDLISQILQQLSASYNNILNNNLND 6r7q.79   ---------------------------------------------------------------------------ALFNG  target    NLTTLTIISVLLAVLAVVTGFFGMNVPLPLTDEPHAWLYISLASAGLWIVLSLLLRKIAKKS 6r7q.79   AQKLFGMIITIGQSIVYV-------------------------------------------- ``` | | | | | | | | | | | | | | | | | | | | | | | | | | | | | | | | | | | | | | | | | | | | | | | | | |
|  | 1vh6.1.A | Flagellar protein fliS  *Crystal structure of a flagellar protein* | 0.00 |  | 0.00 | 0.08 | 115-138 | X-ray | 2.50 | homo-dimer |  | HHblits | 0.20 |
| ``` target    MVLEKQLGNGCTWIDLDLGKLNKLEDLSEIYGLDKETIEYALDRNERAHMDYHRESETVTFIYNVLDVKKDKAYYETFPM 1vh6.1    --------------------------------------------------------------------------------  target    TFIVEHRRLITISNTKNAYVIEQMTRYLENHDTLSIYKFLFASLEIISNAYYPVIEQMDKSRDEVNDLLRQRTTKKNLFV 1vh6.1    ----------------------------------TPGELTLMLYNGCLKFIRLAAQAI----------------------  target    LSDLETGMVYLTAAAKQNRILLEHIQGHALYRSFDEIEREQFDDAMIEAHQLVSMTDLISQILQQLSASYNNILNNNLND 1vh6.1    --------------------------------------------------------------------------------  target    NLTTLTIISVLLAVLAVVTGFFGMNVPLPLTDEPHAWLYISLASAGLWIVLSLLLRKIAKKS 1vh6.1    -------------------------------------------------------------- ``` | | | | | | | | | | | | | | | | | | | | | | | | | | | | | | | | | | | | | | | | | | | | | | | | | |
|  | 7kzm.1.Q | Dynein gamma chain, flagellar outer arm  *Outer dynein arm bound to doublet microtubules from C. reinhardtii* | 0.00 |  | 0.00 | 0.08 | 164-187 | EM | 0.00 | hetero-8-6-1-1-1-1-… | 7 x GTP, 7 x MG, 8 x GDP | HHblits | 0.20 |
| ``` target    MVLEKQLGNGCTWIDLDLGKLNKLEDLSEIYGLDKETIEYALDRNERAHMDYHRESETVTFIYNVLDVKKDKAYYETFPM 7kzm.1    --------------------------------------------------------------------------------  target    TFIVEHRRLITISNTKNAYVIEQMTRYLENHDTLSIYKFLFASLEIISNAYYPVIEQMDKSRDEVNDLLRQRTTKKNLFV 7kzm.1    --------------------------------------------------------------------------------  target    LSDLETGMVYLTAAAKQNRILLEHIQGHALYRSFDEIEREQFDDAMIEAHQLVSMTDLISQILQQLSASYNNILNNNLND 7kzm.1    ---VREKESEIDNLIGPIEEMYGLLMR-----------------------------------------------------  target    NLTTLTIISVLLAVLAVVTGFFGMNVPLPLTDEPHAWLYISLASAGLWIVLSLLLRKIAKKS 7kzm.1    -------------------------------------------------------------- ``` | | | | | | | | | | | | | | | | | | | | | | | | | | | | | | | | | | | | | | | | | | | | | | | | | |
|  | 5ijn.1.G | NUCLEAR PORE COMPLEX PROTEIN NUP58  *Composite structure of the inner ring of the human nuclear pore complex (32 copies of Nup205)* | 0.00 |  | 0.00 | 0.07 | 131-152 | EM | 0.00 | hetero-6-4-4-4-4-4-… |  | HHblits | 0.24 |
| ``` target    MVLEKQLGNGCTWIDLDLGKLNKLEDLSEIYGLDKETIEYALDRNERAHMDYHRESETVTFIYNVLDVKKDKAYYETFPM 5ijn.1    --------------------------------------------------------------------------------  target    TFIVEHRRLITISNTKNAYVIEQMTRYLENHDTLSIYKFLFASLEIISNAYYPVIEQMDKSRDEVNDLLRQRTTKKNLFV 5ijn.1    --------------------------------------------------DENLPPVICQDVENLQKFVKEQ--------  target    LSDLETGMVYLTAAAKQNRILLEHIQGHALYRSFDEIEREQFDDAMIEAHQLVSMTDLISQILQQLSASYNNILNNNLND 5ijn.1    --------------------------------------------------------------------------------  target    NLTTLTIISVLLAVLAVVTGFFGMNVPLPLTDEPHAWLYISLASAGLWIVLSLLLRKIAKKS 5ijn.1    -------------------------------------------------------------- ``` | | | | | | | | | | | | | | | | | | | | | | | | | | | | | | | | | | | | | | | | | | | | | | | | | |
|  | 3pdy.1.A | Plectin  *Structure of the third and fourth spectrin repeats of the plakin domain of plectin* | 0.00 |  | 4.55 | 0.07 | 129-150 | X-ray | 2.22 | monomer |  | HHblits | 0.24 |
| ``` target    MVLEKQLGNGCTWIDLDLGKLNKLEDLSEIYGLDKETIEYALDRNERAHMDYHRESETVTFIYNVLDVKKDKAYYETFPM 3pdy.1    --------------------------------------------------------------------------------  target    TFIVEHRRLITISNTKNAYVIEQMTRYLENHDTLSIYKFLFASLEIISNAYYPVIEQMDKSRDEVNDLLRQRTTKKNLFV 3pdy.1    ------------------------------------------------DSTLRYLQDLLAWVEENQHRVD----------  target    LSDLETGMVYLTAAAKQNRILLEHIQGHALYRSFDEIEREQFDDAMIEAHQLVSMTDLISQILQQLSASYNNILNNNLND 3pdy.1    --------------------------------------------------------------------------------  target    NLTTLTIISVLLAVLAVVTGFFGMNVPLPLTDEPHAWLYISLASAGLWIVLSLLLRKIAKKS 3pdy.1    -------------------------------------------------------------- ``` | | | | | | | | | | | | | | | | | | | | | | | | | | | | | | | | | | | | | | | | | | | | | | | | | |
|  | 3pdy.2.A | Plectin  *Structure of the third and fourth spectrin repeats of the plakin domain of plectin* | 0.00 |  | 4.55 | 0.07 | 129-150 | X-ray | 2.22 | monomer |  | HHblits | 0.24 |
| ``` target    MVLEKQLGNGCTWIDLDLGKLNKLEDLSEIYGLDKETIEYALDRNERAHMDYHRESETVTFIYNVLDVKKDKAYYETFPM 3pdy.2    --------------------------------------------------------------------------------  target    TFIVEHRRLITISNTKNAYVIEQMTRYLENHDTLSIYKFLFASLEIISNAYYPVIEQMDKSRDEVNDLLRQRTTKKNLFV 3pdy.2    ------------------------------------------------DSTLRYLQDLLAWVEENQHRVD----------  target    LSDLETGMVYLTAAAKQNRILLEHIQGHALYRSFDEIEREQFDDAMIEAHQLVSMTDLISQILQQLSASYNNILNNNLND 3pdy.2    --------------------------------------------------------------------------------  target    NLTTLTIISVLLAVLAVVTGFFGMNVPLPLTDEPHAWLYISLASAGLWIVLSLLLRKIAKKS 3pdy.2    -------------------------------------------------------------- ``` | | | | | | | | | | | | | | | | | | | | | | | | | | | | | | | | | | | | | | | | | | | | | | | | | |
|  | 6ys8.1.C | GldL  *Structure of GldLM, the proton-powered motor that drives protein transport and gliding motility* | 0.01 |  | 0.00 | 0.07 | 237-258 | EM | 0.00 | hetero-2-5-mer |  | HHblits | 0.24 |
| ``` target    MVLEKQLGNGCTWIDLDLGKLNKLEDLSEIYGLDKETIEYALDRNERAHMDYHRESETVTFIYNVLDVKKDKAYYETFPM 6ys8.1    --------------------------------------------------------------------------------  target    TFIVEHRRLITISNTKNAYVIEQMTRYLENHDTLSIYKFLFASLEIISNAYYPVIEQMDKSRDEVNDLLRQRTTKKNLFV 6ys8.1    --------------------------------------------------------------------------------  target    LSDLETGMVYLTAAAKQNRILLEHIQGHALYRSFDEIEREQFDDAMIEAHQLVSMTDLISQILQQLSASYNNILNNNLND 6ys8.1    ----------------------------------------------------------------------------LSKK  target    NLTTLTIISVLLAVLAVVTGFFGMNVPLPLTDEPHAWLYISLASAGLWIVLSLLLRKIAKKS 6ys8.1    VMNFAYGMGAAVVIVGAL-------------------------------------------- ``` | | | | | | | | | | | | | | | | | | | | | | | | | | | | | | | | | | | | | | | | | | | | | | | | | |
|  | 6ys8.1.D | GldL  *Structure of GldLM, the proton-powered motor that drives protein transport and gliding motility* | 0.01 |  | 0.00 | 0.07 | 237-258 | EM | 0.00 | hetero-2-5-mer |  | HHblits | 0.24 |
| ``` target    MVLEKQLGNGCTWIDLDLGKLNKLEDLSEIYGLDKETIEYALDRNERAHMDYHRESETVTFIYNVLDVKKDKAYYETFPM 6ys8.1    --------------------------------------------------------------------------------  target    TFIVEHRRLITISNTKNAYVIEQMTRYLENHDTLSIYKFLFASLEIISNAYYPVIEQMDKSRDEVNDLLRQRTTKKNLFV 6ys8.1    --------------------------------------------------------------------------------  target    LSDLETGMVYLTAAAKQNRILLEHIQGHALYRSFDEIEREQFDDAMIEAHQLVSMTDLISQILQQLSASYNNILNNNLND 6ys8.1    ----------------------------------------------------------------------------LSKK  target    NLTTLTIISVLLAVLAVVTGFFGMNVPLPLTDEPHAWLYISLASAGLWIVLSLLLRKIAKKS 6ys8.1    VMNFAYGMGAAVVIVGAL-------------------------------------------- ``` | | | | | | | | | | | | | | | | | | | | | | | | | | | | | | | | | | | | | | | | | | | | | | | | | |
|  | 6ys8.1.E | GldL  *Structure of GldLM, the proton-powered motor that drives protein transport and gliding motility* | 0.01 |  | 0.00 | 0.07 | 237-258 | EM | 0.00 | hetero-2-5-mer |  | HHblits | 0.24 |
| ``` target    MVLEKQLGNGCTWIDLDLGKLNKLEDLSEIYGLDKETIEYALDRNERAHMDYHRESETVTFIYNVLDVKKDKAYYETFPM 6ys8.1    --------------------------------------------------------------------------------  target    TFIVEHRRLITISNTKNAYVIEQMTRYLENHDTLSIYKFLFASLEIISNAYYPVIEQMDKSRDEVNDLLRQRTTKKNLFV 6ys8.1    --------------------------------------------------------------------------------  target    LSDLETGMVYLTAAAKQNRILLEHIQGHALYRSFDEIEREQFDDAMIEAHQLVSMTDLISQILQQLSASYNNILNNNLND 6ys8.1    ----------------------------------------------------------------------------LSKK  target    NLTTLTIISVLLAVLAVVTGFFGMNVPLPLTDEPHAWLYISLASAGLWIVLSLLLRKIAKKS 6ys8.1    VMNFAYGMGAAVVIVGAL-------------------------------------------- ``` | | | | | | | | | | | | | | | | | | | | | | | | | | | | | | | | | | | | | | | | | | | | | | | | | |
|  | 6ys8.1.F | GldL  *Structure of GldLM, the proton-powered motor that drives protein transport and gliding motility* | 0.01 |  | 0.00 | 0.07 | 237-258 | EM | 0.00 | hetero-2-5-mer |  | HHblits | 0.24 |
| ``` target    MVLEKQLGNGCTWIDLDLGKLNKLEDLSEIYGLDKETIEYALDRNERAHMDYHRESETVTFIYNVLDVKKDKAYYETFPM 6ys8.1    --------------------------------------------------------------------------------  target    TFIVEHRRLITISNTKNAYVIEQMTRYLENHDTLSIYKFLFASLEIISNAYYPVIEQMDKSRDEVNDLLRQRTTKKNLFV 6ys8.1    --------------------------------------------------------------------------------  target    LSDLETGMVYLTAAAKQNRILLEHIQGHALYRSFDEIEREQFDDAMIEAHQLVSMTDLISQILQQLSASYNNILNNNLND 6ys8.1    ----------------------------------------------------------------------------LSKK  target    NLTTLTIISVLLAVLAVVTGFFGMNVPLPLTDEPHAWLYISLASAGLWIVLSLLLRKIAKKS 6ys8.1    VMNFAYGMGAAVVIVGAL-------------------------------------------- ``` | | | | | | | | | | | | | | | | | | | | | | | | | | | | | | | | | | | | | | | | | | | | | | | | | |
|  | 6ys8.1.G | GldL  *Structure of GldLM, the proton-powered motor that drives protein transport and gliding motility* | 0.01 |  | 0.00 | 0.07 | 237-258 | EM | 0.00 | hetero-2-5-mer |  | HHblits | 0.24 |
| ``` target    MVLEKQLGNGCTWIDLDLGKLNKLEDLSEIYGLDKETIEYALDRNERAHMDYHRESETVTFIYNVLDVKKDKAYYETFPM 6ys8.1    --------------------------------------------------------------------------------  target    TFIVEHRRLITISNTKNAYVIEQMTRYLENHDTLSIYKFLFASLEIISNAYYPVIEQMDKSRDEVNDLLRQRTTKKNLFV 6ys8.1    --------------------------------------------------------------------------------  target    LSDLETGMVYLTAAAKQNRILLEHIQGHALYRSFDEIEREQFDDAMIEAHQLVSMTDLISQILQQLSASYNNILNNNLND 6ys8.1    ----------------------------------------------------------------------------LSKK  target    NLTTLTIISVLLAVLAVVTGFFGMNVPLPLTDEPHAWLYISLASAGLWIVLSLLLRKIAKKS 6ys8.1    VMNFAYGMGAAVVIVGAL-------------------------------------------- ``` | | | | | | | | | | | | | | | | | | | | | | | | | | | | | | | | | | | | | | | | | | | | | | | | | |
|  | 5nj3.1.A | ATP-binding cassette sub-family G member 2  *Structure of an ABC transporter: complete structure* | 0.00 |  | 5.00 | 0.07 | 243-262 | EM | 0.00 | hetero-2-2-2-mer | 2 x NAG-NAG | HHblits | 0.31 |
| ``` target    MVLEKQLGNGCTWIDLDLGKLNKLEDLSEIYGLDKETIEYALDRNERAHMDYHRESETVTFIYNVLDVKKDKAYYETFPM 5nj3.1    --------------------------------------------------------------------------------  target    TFIVEHRRLITISNTKNAYVIEQMTRYLENHDTLSIYKFLFASLEIISNAYYPVIEQMDKSRDEVNDLLRQRTTKKNLFV 5nj3.1    --------------------------------------------------------------------------------  target    LSDLETGMVYLTAAAKQNRILLEHIQGHALYRSFDEIEREQFDDAMIEAHQLVSMTDLISQILQQLSASYNNILNNNLND 5nj3.1    --------------------------------------------------------------------------------  target    NLTTLTIISVLLAVLAVVTGFFGMNVPLPLTDEPHAWLYISLASAGLWIVLSLLLRKIAKKS 5nj3.1    --VATLLMTICFVFMMIFSGLL---------------------------------------- ``` | | | | | | | | | | | | | | | | | | | | | | | | | | | | | | | | | | | | | | | | | | | | | | | | | |
|  | 6ffc.1.A | ATP-binding cassette sub-family G member 2  *Structure of an inhibitor-bound ABC transporter* | 0.00 |  | 5.00 | 0.07 | 243-262 | EM | 0.00 | homo-dimer | 2 x BWQ | HHblits | 0.31 |
| ``` target    MVLEKQLGNGCTWIDLDLGKLNKLEDLSEIYGLDKETIEYALDRNERAHMDYHRESETVTFIYNVLDVKKDKAYYETFPM 6ffc.1    --------------------------------------------------------------------------------  target    TFIVEHRRLITISNTKNAYVIEQMTRYLENHDTLSIYKFLFASLEIISNAYYPVIEQMDKSRDEVNDLLRQRTTKKNLFV 6ffc.1    --------------------------------------------------------------------------------  target    LSDLETGMVYLTAAAKQNRILLEHIQGHALYRSFDEIEREQFDDAMIEAHQLVSMTDLISQILQQLSASYNNILNNNLND 6ffc.1    --------------------------------------------------------------------------------  target    NLTTLTIISVLLAVLAVVTGFFGMNVPLPLTDEPHAWLYISLASAGLWIVLSLLLRKIAKKS 6ffc.1    --VATLLMTICFVFMMIFSGLL---------------------------------------- ``` | | | | | | | | | | | | | | | | | | | | | | | | | | | | | | | | | | | | | | | | | | | | | | | | | |
|  | 3w5b.1.A | SERCA1a  *Crystal structure of the recombinant SERCA1a (calcium pump of fast twitch skeletal muscle) in the E1.Mg2+ state* | 0.00 |  | 23.81 | 0.07 | 278-298 | X-ray | 3.20 | monomer | 1 x MG, 1 x TM1, 3 x PTY | HHblits | 0.26 |
| ``` target    MVLEKQLGNGCTWIDLDLGKLNKLEDLSEIYGLDKETIEYALDRNERAHMDYHRESETVTFIYNVLDVKKDKAYYETFPM 3w5b.1    --------------------------------------------------------------------------------  target    TFIVEHRRLITISNTKNAYVIEQMTRYLENHDTLSIYKFLFASLEIISNAYYPVIEQMDKSRDEVNDLLRQRTTKKNLFV 3w5b.1    --------------------------------------------------------------------------------  target    LSDLETGMVYLTAAAKQNRILLEHIQGHALYRSFDEIEREQFDDAMIEAHQLVSMTDLISQILQQLSASYNNILNNNLND 3w5b.1    --------------------------------------------------------------------------------  target    NLTTLTIISVLLAVLAVVTGFFGMNVPLPLTDEPHAWLYISLASAGLWIVLSLLLRKIAKKS 3w5b.1    -------------------------------------WLMVLKISLPVIGLDEILKFI---- ``` | | | | | | | | | | | | | | | | | | | | | | | | | | | | | | | | | | | | | | | | | | | | | | | | | |
|  | 5szs.1.A | Spike glycoprotein  *Glycan shield and epitope masking of a coronavirus spike protein observed by cryo-electron microscopy* | 0.00 |  | 15.00 | 0.07 | 168-187 | EM | 3.40 | homo-trimer | 21 x NAG, 3 x NAG-NAG-BMA-MAN-MAN-MAN, 33 x NAG-NAG, 9 x NAG-NAG-BMA-MAN, 3 x NAG-NAG-BMA-MAN-MAN-MAN-MAN-MAN, 9 x NAG-NAG-BMA, 6 x NAG-NAG-BMA-MAN-MAN | HHblits | 0.30 |
| ``` target    MVLEKQLGNGCTWIDLDLGKLNKLEDLSEIYGLDKETIEYALDRNERAHMDYHRESETVTFIYNVLDVKKDKAYYETFPM 5szs.1    --------------------------------------------------------------------------------  target    TFIVEHRRLITISNTKNAYVIEQMTRYLENHDTLSIYKFLFASLEIISNAYYPVIEQMDKSRDEVNDLLRQRTTKKNLFV 5szs.1    --------------------------------------------------------------------------------  target    LSDLETGMVYLTAAAKQNRILLEHIQGHALYRSFDEIEREQFDDAMIEAHQLVSMTDLISQILQQLSASYNNILNNNLND 5szs.1    -------LNKIQDVVNQQGSALNHLTS-----------------------------------------------------  target    NLTTLTIISVLLAVLAVVTGFFGMNVPLPLTDEPHAWLYISLASAGLWIVLSLLLRKIAKKS 5szs.1    -------------------------------------------------------------- ``` | | | | | | | | | | | | | | | | | | | | | | | | | | | | | | | | | | | | | | | | | | | | | | | | | |
|  | 1kmi.1.B | Chemotaxis protein cheZ  *CRYSTAL STRUCTURE OF AN E.COLI CHEMOTAXIS PROTEIN, CHEZ* | 0.00 |  | 5.00 | 0.07 | 230-249 | X-ray | 2.90 | hetero-oligomer | 2 x MG, 2 x BEF, 2 x BCN | HHblits | 0.28 |
| ``` target    MVLEKQLGNGCTWIDLDLGKLNKLEDLSEIYGLDKETIEYALDRNERAHMDYHRESETVTFIYNVLDVKKDKAYYETFPM 1kmi.1    --------------------------------------------------------------------------------  target    TFIVEHRRLITISNTKNAYVIEQMTRYLENHDTLSIYKFLFASLEIISNAYYPVIEQMDKSRDEVNDLLRQRTTKKNLFV 1kmi.1    --------------------------------------------------------------------------------  target    LSDLETGMVYLTAAAKQNRILLEHIQGHALYRSFDEIEREQFDDAMIEAHQLVSMTDLISQILQQLSASYNNILNNNLND 1kmi.1    ---------------------------------------------------------------------FQDLTGQVIKR  target    NLTTLTIISVLLAVLAVVTGFFGMNVPLPLTDEPHAWLYISLASAGLWIVLSLLLRKIAKKS 1kmi.1    MMDVIQEIE----------------------------------------------------- ``` | | | | | | | | | | | | | | | | | | | | | | | | | | | | | | | | | | | | | | | | | | | | | | | | | |
|  | 1mhs.1.A | Plasma Membrane ATPase  *Model of Neurospora crassa proton ATPase* | 0.00 |  | 9.52 | 0.07 | 277-297 | 2DX | 8.00 | homo-dimer |  | HHblits | 0.23 |
| ``` target    MVLEKQLGNGCTWIDLDLGKLNKLEDLSEIYGLDKETIEYALDRNERAHMDYHRESETVTFIYNVLDVKKDKAYYETFPM 1mhs.1    --------------------------------------------------------------------------------  target    TFIVEHRRLITISNTKNAYVIEQMTRYLENHDTLSIYKFLFASLEIISNAYYPVIEQMDKSRDEVNDLLRQRTTKKNLFV 1mhs.1    --------------------------------------------------------------------------------  target    LSDLETGMVYLTAAAKQNRILLEHIQGHALYRSFDEIEREQFDDAMIEAHQLVSMTDLISQILQQLSASYNNILNNNLND 1mhs.1    --------------------------------------------------------------------------------  target    NLTTLTIISVLLAVLAVVTGFFGMNVPLPLTDEPHAWLYISLASAGLWIVLSLLLRKIAKKS 1mhs.1    ------------------------------------IWIFSFGIFCIMGGVYYILQD----- ``` | | | | | | | | | | | | | | | | | | | | | | | | | | | | | | | | | | | | | | | | | | | | | | | | | |
|  | 5j1g.1.A | Plectin  *Structure of the spectrin repeats 7 and 8 of the plakin domain of plectin* | 0.00 |  | 0.00 | 0.07 | 130-150 | X-ray | 1.80 | monomer |  | HHblits | 0.22 |
| ``` target    MVLEKQLGNGCTWIDLDLGKLNKLEDLSEIYGLDKETIEYALDRNERAHMDYHRESETVTFIYNVLDVKKDKAYYETFPM 5j1g.1    --------------------------------------------------------------------------------  target    TFIVEHRRLITISNTKNAYVIEQMTRYLENHDTLSIYKFLFASLEIISNAYYPVIEQMDKSRDEVNDLLRQRTTKKNLFV 5j1g.1    -------------------------------------------------RCISELKDIRLQLEACETRTV----------  target    LSDLETGMVYLTAAAKQNRILLEHIQGHALYRSFDEIEREQFDDAMIEAHQLVSMTDLISQILQQLSASYNNILNNNLND 5j1g.1    --------------------------------------------------------------------------------  target    NLTTLTIISVLLAVLAVVTGFFGMNVPLPLTDEPHAWLYISLASAGLWIVLSLLLRKIAKKS 5j1g.1    -------------------------------------------------------------- ``` | | | | | | | | | | | | | | | | | | | | | | | | | | | | | | | | | | | | | | | | | | | | | | | | | |
|  | 7cyc.1.A | Spike glycoprotein  *Cryo-EM structures of Alphacoronavirus spike glycoprotein* | 0.01 |  | 15.79 | 0.06 | 169-187 | EM | 0.00 | homo-trimer | 48 x NAG, 3 x NAG-NAG-BMA-MAN-MAN-MAN-MAN, 6 x NAG-NAG-BMA, 3 x NAG-NAG | HHblits | 0.28 |
| ``` target    MVLEKQLGNGCTWIDLDLGKLNKLEDLSEIYGLDKETIEYALDRNERAHMDYHRESETVTFIYNVLDVKKDKAYYETFPM 7cyc.1    --------------------------------------------------------------------------------  target    TFIVEHRRLITISNTKNAYVIEQMTRYLENHDTLSIYKFLFASLEIISNAYYPVIEQMDKSRDEVNDLLRQRTTKKNLFV 7cyc.1    --------------------------------------------------------------------------------  target    LSDLETGMVYLTAAAKQNRILLEHIQGHALYRSFDEIEREQFDDAMIEAHQLVSMTDLISQILQQLSASYNNILNNNLND 7cyc.1    --------NKIQDVVNQQGNSLNHLTS-----------------------------------------------------  target    NLTTLTIISVLLAVLAVVTGFFGMNVPLPLTDEPHAWLYISLASAGLWIVLSLLLRKIAKKS 7cyc.1    -------------------------------------------------------------- ``` | | | | | | | | | | | | | | | | | | | | | | | | | | | | | | | | | | | | | | | | | | | | | | | | | |
|  | 7cyd.1.A | Spike glycoprotein  *Cryo-EM structures of Alphacoronavirus spike glycoprotein* | 0.01 |  | 15.79 | 0.06 | 169-187 | EM | 0.00 | homo-trimer | 42 x NAG, 3 x NAG-NAG-BMA-MAN-MAN-MAN-MAN, 3 x NAG-NAG-BMA | HHblits | 0.28 |
| ``` target    MVLEKQLGNGCTWIDLDLGKLNKLEDLSEIYGLDKETIEYALDRNERAHMDYHRESETVTFIYNVLDVKKDKAYYETFPM 7cyd.1    --------------------------------------------------------------------------------  target    TFIVEHRRLITISNTKNAYVIEQMTRYLENHDTLSIYKFLFASLEIISNAYYPVIEQMDKSRDEVNDLLRQRTTKKNLFV 7cyd.1    --------------------------------------------------------------------------------  target    LSDLETGMVYLTAAAKQNRILLEHIQGHALYRSFDEIEREQFDDAMIEAHQLVSMTDLISQILQQLSASYNNILNNNLND 7cyd.1    --------NKIQDVVNQQGNSLNHLTS-----------------------------------------------------  target    NLTTLTIISVLLAVLAVVTGFFGMNVPLPLTDEPHAWLYISLASAGLWIVLSLLLRKIAKKS 7cyd.1    -------------------------------------------------------------- ``` | | | | | | | | | | | | | | | | | | | | | | | | | | | | | | | | | | | | | | | | | | | | | | | | | |
|  | 5c3l.1.B | Nucleoporin Nup58  *Structure of the metazoan Nup62.Nup58.Nup54 nucleoporin complex.* | 0.00 |  | 10.53 | 0.06 | 132-150 | X-ray | 2.90 | hetero-oligomer |  | HHblits | 0.27 |
| ``` target    MVLEKQLGNGCTWIDLDLGKLNKLEDLSEIYGLDKETIEYALDRNERAHMDYHRESETVTFIYNVLDVKKDKAYYETFPM 5c3l.1    --------------------------------------------------------------------------------  target    TFIVEHRRLITISNTKNAYVIEQMTRYLENHDTLSIYKFLFASLEIISNAYYPVIEQMDKSRDEVNDLLRQRTTKKNLFV 5c3l.1    ---------------------------------------------------SKAMLKVQEDIKALKQLLS----------  target    LSDLETGMVYLTAAAKQNRILLEHIQGHALYRSFDEIEREQFDDAMIEAHQLVSMTDLISQILQQLSASYNNILNNNLND 5c3l.1    --------------------------------------------------------------------------------  target    NLTTLTIISVLLAVLAVVTGFFGMNVPLPLTDEPHAWLYISLASAGLWIVLSLLLRKIAKKS 5c3l.1    -------------------------------------------------------------- ``` | | | | | | | | | | | | | | | | | | | | | | | | | | | | | | | | | | | | | | | | | | | | | | | | | |
|  | 6jx7.1.A | Feline Infectious Peritonitis Virus Spike Protein  *Cryo-EM structure of spike protein of feline infectious peritonitis virus strain UU4* | 0.00 |  | 16.67 | 0.06 | 170-187 | EM | 0.00 | homo-trimer | 27 x NAG, 21 x NAG-NAG, 9 x NAG-NAG-BMA, 6 x NAG-NAG-BMA-MAN-MAN-MAN-MAN, 12 x NAG-NAG-BMA-MAN, 3 x NAG-NAG-BMA-FUC, 3 x NAG-NAG-BMA-MAN-MAN | HHblits | 0.30 |
| ``` target    MVLEKQLGNGCTWIDLDLGKLNKLEDLSEIYGLDKETIEYALDRNERAHMDYHRESETVTFIYNVLDVKKDKAYYETFPM 6jx7.1    --------------------------------------------------------------------------------  target    TFIVEHRRLITISNTKNAYVIEQMTRYLENHDTLSIYKFLFASLEIISNAYYPVIEQMDKSRDEVNDLLRQRTTKKNLFV 6jx7.1    --------------------------------------------------------------------------------  target    LSDLETGMVYLTAAAKQNRILLEHIQGHALYRSFDEIEREQFDDAMIEAHQLVSMTDLISQILQQLSASYNNILNNNLND 6jx7.1    ---------KIQSVVNQQGEALSHLIS-----------------------------------------------------  target    NLTTLTIISVLLAVLAVVTGFFGMNVPLPLTDEPHAWLYISLASAGLWIVLSLLLRKIAKKS 6jx7.1    -------------------------------------------------------------- ``` | | | | | | | | | | | | | | | | | | | | | | | | | | | | | | | | | | | | | | | | | | | | | | | | | |
|  | 3okq.1.A | Bud site selection protein 6  *Crystal structure of a core domain of yeast actin nucleation cofactor Bud6* | 0.00 |  | 0.00 | 0.06 | 133-151 | X-ray | 2.04 | homo-dimer |  | HHblits | 0.25 |
| ``` target    MVLEKQLGNGCTWIDLDLGKLNKLEDLSEIYGLDKETIEYALDRNERAHMDYHRESETVTFIYNVLDVKKDKAYYETFPM 3okq.1    --------------------------------------------------------------------------------  target    TFIVEHRRLITISNTKNAYVIEQMTRYLENHDTLSIYKFLFASLEIISNAYYPVIEQMDKSRDEVNDLLRQRTTKKNLFV 3okq.1    ----------------------------------------------------SKVDDLQDVIEIMRKDVAE---------  target    LSDLETGMVYLTAAAKQNRILLEHIQGHALYRSFDEIEREQFDDAMIEAHQLVSMTDLISQILQQLSASYNNILNNNLND 3okq.1    --------------------------------------------------------------------------------  target    NLTTLTIISVLLAVLAVVTGFFGMNVPLPLTDEPHAWLYISLASAGLWIVLSLLLRKIAKKS 3okq.1    -------------------------------------------------------------- ``` | | | | | | | | | | | | | | | | | | | | | | | | | | | | | | | | | | | | | | | | | | | | | | | | | |
|  | 3onx.1.A | Bud site selection protein 6  *Crystal structure of a domain of a protein involved in formation of actin cytoskeleton* | 0.00 |  | 0.00 | 0.06 | 133-151 | X-ray | 2.90 | homo-dimer |  | HHblits | 0.25 |
| ``` target    MVLEKQLGNGCTWIDLDLGKLNKLEDLSEIYGLDKETIEYALDRNERAHMDYHRESETVTFIYNVLDVKKDKAYYETFPM 3onx.1    --------------------------------------------------------------------------------  target    TFIVEHRRLITISNTKNAYVIEQMTRYLENHDTLSIYKFLFASLEIISNAYYPVIEQMDKSRDEVNDLLRQRTTKKNLFV 3onx.1    ----------------------------------------------------SKVDDLQDVIEIMRKDVAE---------  target    LSDLETGMVYLTAAAKQNRILLEHIQGHALYRSFDEIEREQFDDAMIEAHQLVSMTDLISQILQQLSASYNNILNNNLND 3onx.1    --------------------------------------------------------------------------------  target    NLTTLTIISVLLAVLAVVTGFFGMNVPLPLTDEPHAWLYISLASAGLWIVLSLLLRKIAKKS 3onx.1    -------------------------------------------------------------- ``` | | | | | | | | | | | | | | | | | | | | | | | | | | | | | | | | | | | | | | | | | | | | | | | | | |
|  | 3onx.1.B | Bud site selection protein 6  *Crystal structure of a domain of a protein involved in formation of actin cytoskeleton* | 0.00 |  | 0.00 | 0.06 | 133-151 | X-ray | 2.90 | homo-dimer |  | HHblits | 0.25 |
| ``` target    MVLEKQLGNGCTWIDLDLGKLNKLEDLSEIYGLDKETIEYALDRNERAHMDYHRESETVTFIYNVLDVKKDKAYYETFPM 3onx.1    --------------------------------------------------------------------------------  target    TFIVEHRRLITISNTKNAYVIEQMTRYLENHDTLSIYKFLFASLEIISNAYYPVIEQMDKSRDEVNDLLRQRTTKKNLFV 3onx.1    ----------------------------------------------------SKVDDLQDVIEIMRKDVAE---------  target    LSDLETGMVYLTAAAKQNRILLEHIQGHALYRSFDEIEREQFDDAMIEAHQLVSMTDLISQILQQLSASYNNILNNNLND 3onx.1    --------------------------------------------------------------------------------  target    NLTTLTIISVLLAVLAVVTGFFGMNVPLPLTDEPHAWLYISLASAGLWIVLSLLLRKIAKKS 3onx.1    -------------------------------------------------------------- ``` | | | | | | | | | | | | | | | | | | | | | | | | | | | | | | | | | | | | | | | | | | | | | | | | | |
|  | 5h5u.1.D | Peptide chain release factor 2  *Mechanistic insights into the alternative translation termination by ArfA and RF2* | 0.00 |  | 29.41 | 0.06 | 135-151 | EM | 3.00 | hetero-1-1-1-1-1-1-… | 1 x A-C-U-A-U-G | HHblits | 0.33 |
| ``` target    MVLEKQLGNGCTWIDLDLGKLNKLEDLSEIYGLDKETIEYALDRNERAHMDYHRESETVTFIYNVLDVKKDKAYYETFPM 5h5u.1    --------------------------------------------------------------------------------  target    TFIVEHRRLITISNTKNAYVIEQMTRYLENHDTLSIYKFLFASLEIISNAYYPVIEQMDKSRDEVNDLLRQRTTKKNLFV 5h5u.1    ------------------------------------------------------YDAKKERLEEVNAELEQ---------  target    LSDLETGMVYLTAAAKQNRILLEHIQGHALYRSFDEIEREQFDDAMIEAHQLVSMTDLISQILQQLSASYNNILNNNLND 5h5u.1    --------------------------------------------------------------------------------  target    NLTTLTIISVLLAVLAVVTGFFGMNVPLPLTDEPHAWLYISLASAGLWIVLSLLLRKIAKKS 5h5u.1    -------------------------------------------------------------- ``` | | | | | | | | | | | | | | | | | | | | | | | | | | | | | | | | | | | | | | | | | | | | | | | | | |
|  | 5mdv.1.G | Peptide chain release factor 2  *Structure of ArfA and RF2 bound to the 70S ribosome (accommodated state)* | 0.00 |  | 29.41 | 0.06 | 135-151 | EM | 2.97 | hetero-1-1-1-1-1-1-… | 25 x MG, 1 x FME, 2 x ZN | HHblits | 0.33 |
| ``` target    MVLEKQLGNGCTWIDLDLGKLNKLEDLSEIYGLDKETIEYALDRNERAHMDYHRESETVTFIYNVLDVKKDKAYYETFPM 5mdv.1    --------------------------------------------------------------------------------  target    TFIVEHRRLITISNTKNAYVIEQMTRYLENHDTLSIYKFLFASLEIISNAYYPVIEQMDKSRDEVNDLLRQRTTKKNLFV 5mdv.1    ------------------------------------------------------YDAKKERLEEVNAELEQ---------  target    LSDLETGMVYLTAAAKQNRILLEHIQGHALYRSFDEIEREQFDDAMIEAHQLVSMTDLISQILQQLSASYNNILNNNLND 5mdv.1    --------------------------------------------------------------------------------  target    NLTTLTIISVLLAVLAVVTGFFGMNVPLPLTDEPHAWLYISLASAGLWIVLSLLLRKIAKKS 5mdv.1    -------------------------------------------------------------- ``` | | | | | | | | | | | | | | | | | | | | | | | | | | | | | | | | | | | | | | | | | | | | | | | | | |
|  | 5mdw.1.G | Peptide chain release factor 2  *Structure of ArfA(A18T) and RF2 bound to the 70S ribosome (pre-accommodated state)* | 0.00 |  | 29.41 | 0.06 | 135-151 | EM | 3.06 | hetero-1-1-1-1-1-1-… | 25 x MG, 2 x ZN | HHblits | 0.33 |
| ``` target    MVLEKQLGNGCTWIDLDLGKLNKLEDLSEIYGLDKETIEYALDRNERAHMDYHRESETVTFIYNVLDVKKDKAYYETFPM 5mdw.1    --------------------------------------------------------------------------------  target    TFIVEHRRLITISNTKNAYVIEQMTRYLENHDTLSIYKFLFASLEIISNAYYPVIEQMDKSRDEVNDLLRQRTTKKNLFV 5mdw.1    ------------------------------------------------------YDAKKERLEEVNAELEQ---------  target    LSDLETGMVYLTAAAKQNRILLEHIQGHALYRSFDEIEREQFDDAMIEAHQLVSMTDLISQILQQLSASYNNILNNNLND 5mdw.1    --------------------------------------------------------------------------------  target    NLTTLTIISVLLAVLAVVTGFFGMNVPLPLTDEPHAWLYISLASAGLWIVLSLLLRKIAKKS 5mdw.1    -------------------------------------------------------------- ``` | | | | | | | | | | | | | | | | | | | | | | | | | | | | | | | | | | | | | | | | | | | | | | | | | |
|  | 5u9f.1.d | Peptide chain release factor RF2  *3.2 A cryo-EM ArfA-RF2 ribosome rescue complex (Structure II)* | 0.00 |  | 29.41 | 0.06 | 135-151 | EM | 0.00 | hetero-1-1-1-1-1-1-… | 21 x MG, 1 x ZN | HHblits | 0.33 |
| ``` target    MVLEKQLGNGCTWIDLDLGKLNKLEDLSEIYGLDKETIEYALDRNERAHMDYHRESETVTFIYNVLDVKKDKAYYETFPM 5u9f.1    --------------------------------------------------------------------------------  target    TFIVEHRRLITISNTKNAYVIEQMTRYLENHDTLSIYKFLFASLEIISNAYYPVIEQMDKSRDEVNDLLRQRTTKKNLFV 5u9f.1    ------------------------------------------------------YDAKKERLEEVNAELEQ---------  target    LSDLETGMVYLTAAAKQNRILLEHIQGHALYRSFDEIEREQFDDAMIEAHQLVSMTDLISQILQQLSASYNNILNNNLND 5u9f.1    --------------------------------------------------------------------------------  target    NLTTLTIISVLLAVLAVVTGFFGMNVPLPLTDEPHAWLYISLASAGLWIVLSLLLRKIAKKS 5u9f.1    -------------------------------------------------------------- ``` | | | | | | | | | | | | | | | | | | | | | | | | | | | | | | | | | | | | | | | | | | | | | | | | | |
|  | 5u9g.1.d | Peptide chain release factor RF2  *3.2 A cryo-EM ArfA-RF2 ribosome rescue complex (Structure I)* | 0.00 |  | 29.41 | 0.06 | 135-151 | EM | 0.00 | hetero-1-1-1-1-1-1-… | 20 x MG, 1 x ZN | HHblits | 0.33 |
| ``` target    MVLEKQLGNGCTWIDLDLGKLNKLEDLSEIYGLDKETIEYALDRNERAHMDYHRESETVTFIYNVLDVKKDKAYYETFPM 5u9g.1    --------------------------------------------------------------------------------  target    TFIVEHRRLITISNTKNAYVIEQMTRYLENHDTLSIYKFLFASLEIISNAYYPVIEQMDKSRDEVNDLLRQRTTKKNLFV 5u9g.1    ------------------------------------------------------YDAKKERLEEVNAELEQ---------  target    LSDLETGMVYLTAAAKQNRILLEHIQGHALYRSFDEIEREQFDDAMIEAHQLVSMTDLISQILQQLSASYNNILNNNLND 5u9g.1    --------------------------------------------------------------------------------  target    NLTTLTIISVLLAVLAVVTGFFGMNVPLPLTDEPHAWLYISLASAGLWIVLSLLLRKIAKKS 5u9g.1    -------------------------------------------------------------- ``` | | | | | | | | | | | | | | | | | | | | | | | | | | | | | | | | | | | | | | | | | | | | | | | | | |
|  | 3ghg.1.B | Fibrinogen beta chain  *Crystal Structure of Human Fibrinogen* | 0.00 |  | 23.53 | 0.06 | 133-149 | X-ray | 2.90 | hetero-2-2-2-mer | 4 x CA, 2 x GLY-PRO-ARG-PRO, 2 x GLY-HIS-ARG-PRO, 2 x NAG-NDG-BMA-MAN-NDG-GAL-SIA-MAN-NDG-GAL-SIA | HHblits | 0.31 |
| ``` target    MVLEKQLGNGCTWIDLDLGKLNKLEDLSEIYGLDKETIEYALDRNERAHMDYHRESETVTFIYNVLDVKKDKAYYETFPM 3ghg.1    --------------------------------------------------------------------------------  target    TFIVEHRRLITISNTKNAYVIEQMTRYLENHDTLSIYKFLFASLEIISNAYYPVIEQMDKSRDEVNDLLRQRTTKKNLFV 3ghg.1    ----------------------------------------------------QQERPIRNSVDELNNNV-----------  target    LSDLETGMVYLTAAAKQNRILLEHIQGHALYRSFDEIEREQFDDAMIEAHQLVSMTDLISQILQQLSASYNNILNNNLND 3ghg.1    --------------------------------------------------------------------------------  target    NLTTLTIISVLLAVLAVVTGFFGMNVPLPLTDEPHAWLYISLASAGLWIVLSLLLRKIAKKS 3ghg.1    -------------------------------------------------------------- ``` | | | | | | | | | | | | | | | | | | | | | | | | | | | | | | | | | | | | | | | | | | | | | | | | | |
|  | 3ghg.1.E | Fibrinogen beta chain  *Crystal Structure of Human Fibrinogen* | 0.00 |  | 23.53 | 0.06 | 133-149 | X-ray | 2.90 | hetero-2-2-2-mer | 4 x CA, 2 x GLY-PRO-ARG-PRO, 2 x GLY-HIS-ARG-PRO, 2 x NAG-NDG-BMA-MAN-NDG-GAL-SIA-MAN-NDG-GAL-SIA | HHblits | 0.31 |
| ``` target    MVLEKQLGNGCTWIDLDLGKLNKLEDLSEIYGLDKETIEYALDRNERAHMDYHRESETVTFIYNVLDVKKDKAYYETFPM 3ghg.1    --------------------------------------------------------------------------------  target    TFIVEHRRLITISNTKNAYVIEQMTRYLENHDTLSIYKFLFASLEIISNAYYPVIEQMDKSRDEVNDLLRQRTTKKNLFV 3ghg.1    ----------------------------------------------------QQERPIRNSVDELNNNV-----------  target    LSDLETGMVYLTAAAKQNRILLEHIQGHALYRSFDEIEREQFDDAMIEAHQLVSMTDLISQILQQLSASYNNILNNNLND 3ghg.1    --------------------------------------------------------------------------------  target    NLTTLTIISVLLAVLAVVTGFFGMNVPLPLTDEPHAWLYISLASAGLWIVLSLLLRKIAKKS 3ghg.1    -------------------------------------------------------------- ``` | | | | | | | | | | | | | | | | | | | | | | | | | | | | | | | | | | | | | | | | | | | | | | | | | |
|  | 3ghg.2.B | Fibrinogen beta chain  *Crystal Structure of Human Fibrinogen* | 0.00 |  | 23.53 | 0.06 | 133-149 | X-ray | 2.90 | hetero-2-2-2-mer | 4 x CA, 2 x GLY-PRO-ARG-PRO, 2 x GLY-HIS-ARG-PRO, 1 x NAG-NDG-BMA-MAN-MAN, 1 x NAG-NDG-BMA-MAN-NDG-GAL-SIA-MAN-NDG-GAL-SIA, 1 x NAG-NAG | HHblits | 0.31 |
| ``` target    MVLEKQLGNGCTWIDLDLGKLNKLEDLSEIYGLDKETIEYALDRNERAHMDYHRESETVTFIYNVLDVKKDKAYYETFPM 3ghg.2    --------------------------------------------------------------------------------  target    TFIVEHRRLITISNTKNAYVIEQMTRYLENHDTLSIYKFLFASLEIISNAYYPVIEQMDKSRDEVNDLLRQRTTKKNLFV 3ghg.2    ----------------------------------------------------QQERPIRNSVDELNNNV-----------  target    LSDLETGMVYLTAAAKQNRILLEHIQGHALYRSFDEIEREQFDDAMIEAHQLVSMTDLISQILQQLSASYNNILNNNLND 3ghg.2    --------------------------------------------------------------------------------  target    NLTTLTIISVLLAVLAVVTGFFGMNVPLPLTDEPHAWLYISLASAGLWIVLSLLLRKIAKKS 3ghg.2    -------------------------------------------------------------- ``` | | | | | | | | | | | | | | | | | | | | | | | | | | | | | | | | | | | | | | | | | | | | | | | | | |
|  | 3ghg.2.E | Fibrinogen beta chain  *Crystal Structure of Human Fibrinogen* | 0.00 |  | 23.53 | 0.06 | 133-149 | X-ray | 2.90 | hetero-2-2-2-mer | 4 x CA, 2 x GLY-PRO-ARG-PRO, 2 x GLY-HIS-ARG-PRO, 1 x NAG-NDG-BMA-MAN-MAN, 1 x NAG-NDG-BMA-MAN-NDG-GAL-SIA-MAN-NDG-GAL-SIA, 1 x NAG-NAG | HHblits | 0.31 |
| ``` target    MVLEKQLGNGCTWIDLDLGKLNKLEDLSEIYGLDKETIEYALDRNERAHMDYHRESETVTFIYNVLDVKKDKAYYETFPM 3ghg.2    --------------------------------------------------------------------------------  target    TFIVEHRRLITISNTKNAYVIEQMTRYLENHDTLSIYKFLFASLEIISNAYYPVIEQMDKSRDEVNDLLRQRTTKKNLFV 3ghg.2    ----------------------------------------------------QQERPIRNSVDELNNNV-----------  target    LSDLETGMVYLTAAAKQNRILLEHIQGHALYRSFDEIEREQFDDAMIEAHQLVSMTDLISQILQQLSASYNNILNNNLND 3ghg.2    --------------------------------------------------------------------------------  target    NLTTLTIISVLLAVLAVVTGFFGMNVPLPLTDEPHAWLYISLASAGLWIVLSLLLRKIAKKS 3ghg.2    -------------------------------------------------------------- ``` | | | | | | | | | | | | | | | | | | | | | | | | | | | | | | | | | | | | | | | | | | | | | | | | | |
|  | 6c5l.1.Y | Peptide chain release factor 2  *Conformation of methylated GGQ in the Peptidyl Transferase Center during translation termination (T. thermophilus)* | 0.00 |  | 23.53 | 0.06 | 135-151 | X-ray | 3.20 | hetero-1-1-1-1-1-1-… | 26 x MG, 3 x ZN | HHblits | 0.31 |
| ``` target    MVLEKQLGNGCTWIDLDLGKLNKLEDLSEIYGLDKETIEYALDRNERAHMDYHRESETVTFIYNVLDVKKDKAYYETFPM 6c5l.1    --------------------------------------------------------------------------------  target    TFIVEHRRLITISNTKNAYVIEQMTRYLENHDTLSIYKFLFASLEIISNAYYPVIEQMDKSRDEVNDLLRQRTTKKNLFV 6c5l.1    ------------------------------------------------------IPQKETRLKELERRLED---------  target    LSDLETGMVYLTAAAKQNRILLEHIQGHALYRSFDEIEREQFDDAMIEAHQLVSMTDLISQILQQLSASYNNILNNNLND 6c5l.1    --------------------------------------------------------------------------------  target    NLTTLTIISVLLAVLAVVTGFFGMNVPLPLTDEPHAWLYISLASAGLWIVLSLLLRKIAKKS 6c5l.1    -------------------------------------------------------------- ``` | | | | | | | | | | | | | | | | | | | | | | | | | | | | | | | | | | | | | | | | | | | | | | | | | |
|  | 6c5l.2.Y | Peptide chain release factor 2  *Conformation of methylated GGQ in the Peptidyl Transferase Center during translation termination (T. thermophilus)* | 0.00 |  | 23.53 | 0.06 | 135-151 | X-ray | 3.20 | hetero-1-1-1-1-1-1-… | 27 x MG, 3 x ZN | HHblits | 0.31 |
| ``` target    MVLEKQLGNGCTWIDLDLGKLNKLEDLSEIYGLDKETIEYALDRNERAHMDYHRESETVTFIYNVLDVKKDKAYYETFPM 6c5l.2    --------------------------------------------------------------------------------  target    TFIVEHRRLITISNTKNAYVIEQMTRYLENHDTLSIYKFLFASLEIISNAYYPVIEQMDKSRDEVNDLLRQRTTKKNLFV 6c5l.2    ------------------------------------------------------IPQKETRLKELERRLED---------  target    LSDLETGMVYLTAAAKQNRILLEHIQGHALYRSFDEIEREQFDDAMIEAHQLVSMTDLISQILQQLSASYNNILNNNLND 6c5l.2    --------------------------------------------------------------------------------  target    NLTTLTIISVLLAVLAVVTGFFGMNVPLPLTDEPHAWLYISLASAGLWIVLSLLLRKIAKKS 6c5l.2    -------------------------------------------------------------- ``` | | | | | | | | | | | | | | | | | | | | | | | | | | | | | | | | | | | | | | | | | | | | | | | | | |
|  | 4v5j.1.X | PEPTIDE CHAIN RELEASE FACTOR 2  *Structure of the 70S ribosome bound to Release factor 2 and a substrate analog provides insights into catalysis of peptide release* | 0.00 |  | 23.53 | 0.06 | 135-151 | X-ray | 3.10 | hetero-1-1-1-1-1-1-… | 49 x MG, 3 x ZN, 1 x A-A-U-U-C-U-A-A | HHblits | 0.31 |
| ``` target    MVLEKQLGNGCTWIDLDLGKLNKLEDLSEIYGLDKETIEYALDRNERAHMDYHRESETVTFIYNVLDVKKDKAYYETFPM 4v5j.1    --------------------------------------------------------------------------------  target    TFIVEHRRLITISNTKNAYVIEQMTRYLENHDTLSIYKFLFASLEIISNAYYPVIEQMDKSRDEVNDLLRQRTTKKNLFV 4v5j.1    ------------------------------------------------------IPQKETRLKELERRLED---------  target    LSDLETGMVYLTAAAKQNRILLEHIQGHALYRSFDEIEREQFDDAMIEAHQLVSMTDLISQILQQLSASYNNILNNNLND 4v5j.1    --------------------------------------------------------------------------------  target    NLTTLTIISVLLAVLAVVTGFFGMNVPLPLTDEPHAWLYISLASAGLWIVLSLLLRKIAKKS 4v5j.1    -------------------------------------------------------------- ``` | | | | | | | | | | | | | | | | | | | | | | | | | | | | | | | | | | | | | | | | | | | | | | | | | |
|  | 4v5j.2.X | PEPTIDE CHAIN RELEASE FACTOR 2  *Structure of the 70S ribosome bound to Release factor 2 and a substrate analog provides insights into catalysis of peptide release* | 0.00 |  | 23.53 | 0.06 | 135-151 | X-ray | 3.10 | hetero-1-1-1-1-1-1-… | 46 x MG, 3 x ZN, 1 x A-A-U-U-C-U-A-A | HHblits | 0.31 |
| ``` target    MVLEKQLGNGCTWIDLDLGKLNKLEDLSEIYGLDKETIEYALDRNERAHMDYHRESETVTFIYNVLDVKKDKAYYETFPM 4v5j.2    --------------------------------------------------------------------------------  target    TFIVEHRRLITISNTKNAYVIEQMTRYLENHDTLSIYKFLFASLEIISNAYYPVIEQMDKSRDEVNDLLRQRTTKKNLFV 4v5j.2    ------------------------------------------------------IPQKETRLKELERRLED---------  target    LSDLETGMVYLTAAAKQNRILLEHIQGHALYRSFDEIEREQFDDAMIEAHQLVSMTDLISQILQQLSASYNNILNNNLND 4v5j.2    --------------------------------------------------------------------------------  target    NLTTLTIISVLLAVLAVVTGFFGMNVPLPLTDEPHAWLYISLASAGLWIVLSLLLRKIAKKS 4v5j.2    -------------------------------------------------------------- ``` | | | | | | | | | | | | | | | | | | | | | | | | | | | | | | | | | | | | | | | | | | | | | | | | | |
|  | 4v67.1.Y | Bacterial peptide chain release factor 2 (RF-2)  *Crystal structure of a translation termination complex formed with release factor RF2.* | 0.00 |  | 23.53 | 0.06 | 135-151 | X-ray | 3.00 | hetero-oligomer | 115 x MG, 2 x ZN | HHblits | 0.31 |
| ``` target    MVLEKQLGNGCTWIDLDLGKLNKLEDLSEIYGLDKETIEYALDRNERAHMDYHRESETVTFIYNVLDVKKDKAYYETFPM 4v67.1    --------------------------------------------------------------------------------  target    TFIVEHRRLITISNTKNAYVIEQMTRYLENHDTLSIYKFLFASLEIISNAYYPVIEQMDKSRDEVNDLLRQRTTKKNLFV 4v67.1    ------------------------------------------------------IPQKETRLKELERRLED---------  target    LSDLETGMVYLTAAAKQNRILLEHIQGHALYRSFDEIEREQFDDAMIEAHQLVSMTDLISQILQQLSASYNNILNNNLND 4v67.1    --------------------------------------------------------------------------------  target    NLTTLTIISVLLAVLAVVTGFFGMNVPLPLTDEPHAWLYISLASAGLWIVLSLLLRKIAKKS 4v67.1    -------------------------------------------------------------- ``` | | | | | | | | | | | | | | | | | | | | | | | | | | | | | | | | | | | | | | | | | | | | | | | | | |
|  | 5mdy.1.G | Peptide chain release factor 2  *Structure of ArfA and TtRF2 bound to the 70S ribosome (pre-accommodated state)* | 0.00 |  | 23.53 | 0.06 | 135-151 | EM | 3.35 | hetero-1-1-1-1-1-1-… | 19 x MG, 2 x ZN | HHblits | 0.31 |
| ``` target    MVLEKQLGNGCTWIDLDLGKLNKLEDLSEIYGLDKETIEYALDRNERAHMDYHRESETVTFIYNVLDVKKDKAYYETFPM 5mdy.1    --------------------------------------------------------------------------------  target    TFIVEHRRLITISNTKNAYVIEQMTRYLENHDTLSIYKFLFASLEIISNAYYPVIEQMDKSRDEVNDLLRQRTTKKNLFV 5mdy.1    ------------------------------------------------------IPQKETRLKELERRLED---------  target    LSDLETGMVYLTAAAKQNRILLEHIQGHALYRSFDEIEREQFDDAMIEAHQLVSMTDLISQILQQLSASYNNILNNNLND 5mdy.1    --------------------------------------------------------------------------------  target    NLTTLTIISVLLAVLAVVTGFFGMNVPLPLTDEPHAWLYISLASAGLWIVLSLLLRKIAKKS 5mdy.1    -------------------------------------------------------------- ``` | | | | | | | | | | | | | | | | | | | | | | | | | | | | | | | | | | | | | | | | | | | | | | | | | |
|  | 6m3p.1.B | Spectrin beta chain, non-erythrocytic 1  *Crystal structure of AnkG/beta2-spectrin complex* | 0.00 |  | 11.11 | 0.06 | 132-149 | X-ray | 3.31 | hetero-1-1-mer |  | HHblits | 0.25 |
| ``` target    MVLEKQLGNGCTWIDLDLGKLNKLEDLSEIYGLDKETIEYALDRNERAHMDYHRESETVTFIYNVLDVKKDKAYYETFPM 6m3p.1    --------------------------------------------------------------------------------  target    TFIVEHRRLITISNTKNAYVIEQMTRYLENHDTLSIYKFLFASLEIISNAYYPVIEQMDKSRDEVNDLLRQRTTKKNLFV 6m3p.1    ---------------------------------------------------YFDAAEAEAWMSEQELYM-----------  target    LSDLETGMVYLTAAAKQNRILLEHIQGHALYRSFDEIEREQFDDAMIEAHQLVSMTDLISQILQQLSASYNNILNNNLND 6m3p.1    --------------------------------------------------------------------------------  target    NLTTLTIISVLLAVLAVVTGFFGMNVPLPLTDEPHAWLYISLASAGLWIVLSLLLRKIAKKS 6m3p.1    -------------------------------------------------------------- ``` | | | | | | | | | | | | | | | | | | | | | | | | | | | | | | | | | | | | | | | | | | | | | | | | | |
|  | 6vv5.1.A | Spike glycoprotein  *Cryo-EM structure of porcine epidemic diarrhea virus (PEDV) spike protein* | 0.00 |  | 5.56 | 0.06 | 170-187 | EM | 0.00 | homo-trimer | 15 x NAG, 6 x PAM, 3 x NAG-NAG-BMA-MAN-MAN-MAN-NAG-MAN, 24 x NAG-NAG, 3 x NAG-NAG-BMA | HHblits | 0.24 |
| ``` target    MVLEKQLGNGCTWIDLDLGKLNKLEDLSEIYGLDKETIEYALDRNERAHMDYHRESETVTFIYNVLDVKKDKAYYETFPM 6vv5.1    --------------------------------------------------------------------------------  target    TFIVEHRRLITISNTKNAYVIEQMTRYLENHDTLSIYKFLFASLEIISNAYYPVIEQMDKSRDEVNDLLRQRTTKKNLFV 6vv5.1    --------------------------------------------------------------------------------  target    LSDLETGMVYLTAAAKQNRILLEHIQGHALYRSFDEIEREQFDDAMIEAHQLVSMTDLISQILQQLSASYNNILNNNLND 6vv5.1    ---------KVQEVVNSQGAALTQLTV-----------------------------------------------------  target    NLTTLTIISVLLAVLAVVTGFFGMNVPLPLTDEPHAWLYISLASAGLWIVLSLLLRKIAKKS 6vv5.1    -------------------------------------------------------------- ``` | | | | | | | | | | | | | | | | | | | | | | | | | | | | | | | | | | | | | | | | | | | | | | | | | |
|  | 5ijn.1.G | NUCLEAR PORE COMPLEX PROTEIN NUP58  *Composite structure of the inner ring of the human nuclear pore complex (32 copies of Nup205)* | 0.00 |  | 11.76 | 0.06 | 133-149 | EM | 0.00 | hetero-6-4-4-4-4-4-… |  | HHblits | 0.28 |
| ``` target    MVLEKQLGNGCTWIDLDLGKLNKLEDLSEIYGLDKETIEYALDRNERAHMDYHRESETVTFIYNVLDVKKDKAYYETFPM 5ijn.1    --------------------------------------------------------------------------------  target    TFIVEHRRLITISNTKNAYVIEQMTRYLENHDTLSIYKFLFASLEIISNAYYPVIEQMDKSRDEVNDLLRQRTTKKNLFV 5ijn.1    ----------------------------------------------------KAMLKVQEDIKALKQLL-----------  target    LSDLETGMVYLTAAAKQNRILLEHIQGHALYRSFDEIEREQFDDAMIEAHQLVSMTDLISQILQQLSASYNNILNNNLND 5ijn.1    --------------------------------------------------------------------------------  target    NLTTLTIISVLLAVLAVVTGFFGMNVPLPLTDEPHAWLYISLASAGLWIVLSLLLRKIAKKS 5ijn.1    -------------------------------------------------------------- ``` | | | | | | | | | | | | | | | | | | | | | | | | | | | | | | | | | | | | | | | | | | | | | | | | | |
|  | 6wvg.1.A | Green fluorescent protein, Leukocyte surface antigen CD53 chimera  *human CD53* | 0.00 |  | 11.76 | 0.06 | 251-267 | X-ray | 2.90 | monomer | 4 x OLC, 1 x NAG | HHblits | 0.27 |
| ``` target    MVLEKQLGNGCTWIDLDLGKLNKLEDLSEIYGLDKETIEYALDRNERAHMDYHRESETVTFIYNVLDVKKDKAYYETFPM 6wvg.1    --------------------------------------------------------------------------------  target    TFIVEHRRLITISNTKNAYVIEQMTRYLENHDTLSIYKFLFASLEIISNAYYPVIEQMDKSRDEVNDLLRQRTTKKNLFV 6wvg.1    --------------------------------------------------------------------------------  target    LSDLETGMVYLTAAAKQNRILLEHIQGHALYRSFDEIEREQFDDAMIEAHQLVSMTDLISQILQQLSASYNNILNNNLND 6wvg.1    --------------------------------------------------------------------------------  target    NLTTLTIISVLLAVLAVVTGFFGMNVPLPLTDEPHAWLYISLASAGLWIVLSLLLRKIAKKS 6wvg.1    ----------SIIMVVAFLGCMGSIKE----------------------------------- ``` | | | | | | | | | | | | | | | | | | | | | | | | | | | | | | | | | | | | | | | | | | | | | | | | | |
|  | 5j4z.76.A | COMPLEX IV COX4  *Architecture of tight respirasome* | 0.00 |  | 0.00 | 0.06 | 261-278 | EM | 5.80 | monomer |  | HHblits | 0.22 |
| ``` target    MVLEKQLGNGCTWIDLDLGKLNKLEDLSEIYGLDKETIEYALDRNERAHMDYHRESETVTFIYNVLDVKKDKAYYETFPM 5j4z.76   --------------------------------------------------------------------------------  target    TFIVEHRRLITISNTKNAYVIEQMTRYLENHDTLSIYKFLFASLEIISNAYYPVIEQMDKSRDEVNDLLRQRTTKKNLFV 5j4z.76   --------------------------------------------------------------------------------  target    LSDLETGMVYLTAAAKQNRILLEHIQGHALYRSFDEIEREQFDDAMIEAHQLVSMTDLISQILQQLSASYNNILNNNLND 5j4z.76   --------------------------------------------------------------------------------  target    NLTTLTIISVLLAVLAVVTGFFGMNVP--LPLTDEPHAWLYISLASAGLWIVLSLLLRKIAKKS 5j4z.76   --------------------LYRLKFKESFAEMNRSTNEW------------------------ ``` | | | | | | | | | | | | | | | | | | | | | | | | | | | | | | | | | | | | | | | | | | | | | | | | | |
|  | 4v5e.1.X | PEPTIDE CHAIN RELEASE FACTOR 2  *Insights into translational termination from the structure of RF2 bound to the ribosome* | 0.00 |  | 25.00 | 0.05 | 135-150 | X-ray | 3.45 | hetero-1-1-1-1-1-1-… | 50 x MG, 3 x ZN, 1 x A-A-U-U-C-U-G-A | HHblits | 0.31 |
| ``` target    MVLEKQLGNGCTWIDLDLGKLNKLEDLSEIYGLDKETIEYALDRNERAHMDYHRESETVTFIYNVLDVKKDKAYYETFPM 4v5e.1    --------------------------------------------------------------------------------  target    TFIVEHRRLITISNTKNAYVIEQMTRYLENHDTLSIYKFLFASLEIISNAYYPVIEQMDKSRDEVNDLLRQRTTKKNLFV 4v5e.1    ------------------------------------------------------IPQKETRLKELERRLE----------  target    LSDLETGMVYLTAAAKQNRILLEHIQGHALYRSFDEIEREQFDDAMIEAHQLVSMTDLISQILQQLSASYNNILNNNLND 4v5e.1    --------------------------------------------------------------------------------  target    NLTTLTIISVLLAVLAVVTGFFGMNVPLPLTDEPHAWLYISLASAGLWIVLSLLLRKIAKKS 4v5e.1    -------------------------------------------------------------- ``` | | | | | | | | | | | | | | | | | | | | | | | | | | | | | | | | | | | | | | | | | | | | | | | | | |
|  | 4v5e.2.X | PEPTIDE CHAIN RELEASE FACTOR 2  *Insights into translational termination from the structure of RF2 bound to the ribosome* | 0.00 |  | 25.00 | 0.05 | 135-150 | X-ray | 3.45 | hetero-1-1-1-1-1-1-… | 56 x MG, 3 x ZN, 1 x A-A-U-U-C-U-G-A | HHblits | 0.31 |
| ``` target    MVLEKQLGNGCTWIDLDLGKLNKLEDLSEIYGLDKETIEYALDRNERAHMDYHRESETVTFIYNVLDVKKDKAYYETFPM 4v5e.2    --------------------------------------------------------------------------------  target    TFIVEHRRLITISNTKNAYVIEQMTRYLENHDTLSIYKFLFASLEIISNAYYPVIEQMDKSRDEVNDLLRQRTTKKNLFV 4v5e.2    ------------------------------------------------------IPQKETRLKELERRLE----------  target    LSDLETGMVYLTAAAKQNRILLEHIQGHALYRSFDEIEREQFDDAMIEAHQLVSMTDLISQILQQLSASYNNILNNNLND 4v5e.2    --------------------------------------------------------------------------------  target    NLTTLTIISVLLAVLAVVTGFFGMNVPLPLTDEPHAWLYISLASAGLWIVLSLLLRKIAKKS 4v5e.2    -------------------------------------------------------------- ``` | | | | | | | | | | | | | | | | | | | | | | | | | | | | | | | | | | | | | | | | | | | | | | | | | |
|  | 4v9n.1.D | Bacterial peptide chain release factor 2 (RF-2)  *Crystal structure of the 70S ribosome bound with the Q253P mutant of release factor RF2.* | 0.00 |  | 25.00 | 0.05 | 135-150 | X-ray | 3.40 | hetero-1-1-1-1-1-1-… | 71 x MG, 2 x ZN | HHblits | 0.31 |
| ``` target    MVLEKQLGNGCTWIDLDLGKLNKLEDLSEIYGLDKETIEYALDRNERAHMDYHRESETVTFIYNVLDVKKDKAYYETFPM 4v9n.1    --------------------------------------------------------------------------------  target    TFIVEHRRLITISNTKNAYVIEQMTRYLENHDTLSIYKFLFASLEIISNAYYPVIEQMDKSRDEVNDLLRQRTTKKNLFV 4v9n.1    ------------------------------------------------------IPQKETRLKELERRLE----------  target    LSDLETGMVYLTAAAKQNRILLEHIQGHALYRSFDEIEREQFDDAMIEAHQLVSMTDLISQILQQLSASYNNILNNNLND 4v9n.1    --------------------------------------------------------------------------------  target    NLTTLTIISVLLAVLAVVTGFFGMNVPLPLTDEPHAWLYISLASAGLWIVLSLLLRKIAKKS 4v9n.1    -------------------------------------------------------------- ``` | | | | | | | | | | | | | | | | | | | | | | | | | | | | | | | | | | | | | | | | | | | | | | | | | |
|  | 5ijn.1.H | Nuclear pore glycoprotein p62  *Composite structure of the inner ring of the human nuclear pore complex (32 copies of Nup205)* | 0.00 |  | 18.75 | 0.05 | 231-246 | EM | 0.00 | hetero-6-4-4-4-4-4-… |  | HHblits | 0.29 |
| ``` target    MVLEKQLGNGCTWIDLDLGKLNKLEDLSEIYGLDKETIEYALDRNERAHMDYHRESETVTFIYNVLDVKKDKAYYETFPM 5ijn.1    --------------------------------------------------------------------------------  target    TFIVEHRRLITISNTKNAYVIEQMTRYLENHDTLSIYKFLFASLEIISNAYYPVIEQMDKSRDEVNDLLRQRTTKKNLFV 5ijn.1    --------------------------------------------------------------------------------  target    LSDLETGMVYLTAAAKQNRILLEHIQGHALYRSFDEIEREQFDDAMIEAHQLVSMTDLISQILQQLSASYNNILNNNLND 5ijn.1    ----------------------------------------------------------------------LKRMAQDLKD  target    NLTTLTIISVLLAVLAVVTGFFGMNVPLPLTDEPHAWLYISLASAGLWIVLSLLLRKIAKKS 5ijn.1    IIEHLN-------------------------------------------------------- ``` | | | | | | | | | | | | | | | | | | | | | | | | | | | | | | | | | | | | | | | | | | | | | | | | | |
|  | 3abm.1.D | Cytochrome c oxidase subunit 4 isoform 1  *Bovine heart cytochrome c oxidase at the fully oxidized state (200-s X-ray exposure dataset)* | 0.00 |  | 0.00 | 0.06 | 261-277 | X-ray | 1.95 | hetero-oligomer | 1 x CU, 1 x PER, 1 x MG, 2 x HEA, 3 x TGL, 4 x PGV, 1 x CUA, 4 x CHD, 3 x PEK, 2 x CDL, 1 x PSC, 1 x ZN, 2 x DMU | HHblits | 0.22 |
| ``` target    MVLEKQLGNGCTWIDLDLGKLNKLEDLSEIYGLDKETIEYALDRNERAHMDYHRESETVTFIYNVLDVKKDKAYYETFPM 3abm.1    --------------------------------------------------------------------------------  target    TFIVEHRRLITISNTKNAYVIEQMTRYLENHDTLSIYKFLFASLEIISNAYYPVIEQMDKSRDEVNDLLRQRTTKKNLFV 3abm.1    --------------------------------------------------------------------------------  target    LSDLETGMVYLTAAAKQNRILLEHIQGHALYRSFDEIEREQFDDAMIEAHQLVSMTDLISQILQQLSASYNNILNNNLND 3abm.1    --------------------------------------------------------------------------------  target    NLTTLTIISVLLAVLAVVTGFFGMNVP--LPLTDEPHAWLYISLASAGLWIVLSLLLRKIAKKS 3abm.1    --------------------LYRLKFKESFAEMNRSTNE------------------------- ``` | | | | | | | | | | | | | | | | | | | | | | | | | | | | | | | | | | | | | | | | | | | | | | | | | |
|  | 5xdq.2.D | Cytochrome c oxidase subunit 4 isoform 1, mitochondrial  *Bovine heart cytochrome c oxidase in the fully oxidized state with pH 7.3 at 1.77 angstrom resolution* | 0.00 |  | 0.00 | 0.06 | 261-277 | X-ray | 1.77 | hetero-1-1-1-1-1-1-… | 2 x HEA, 1 x CU, 1 x MG, 3 x TGL, 4 x PGV, 1 x PER, 1 x CUA, 1 x PSC, 3 x CHD, 3 x PEK, 2 x CDL, 3 x DMU, 1 x ZN | HHblits | 0.22 |
| ``` target    MVLEKQLGNGCTWIDLDLGKLNKLEDLSEIYGLDKETIEYALDRNERAHMDYHRESETVTFIYNVLDVKKDKAYYETFPM 5xdq.2    --------------------------------------------------------------------------------  target    TFIVEHRRLITISNTKNAYVIEQMTRYLENHDTLSIYKFLFASLEIISNAYYPVIEQMDKSRDEVNDLLRQRTTKKNLFV 5xdq.2    --------------------------------------------------------------------------------  target    LSDLETGMVYLTAAAKQNRILLEHIQGHALYRSFDEIEREQFDDAMIEAHQLVSMTDLISQILQQLSASYNNILNNNLND 5xdq.2    --------------------------------------------------------------------------------  target    NLTTLTIISVLLAVLAVVTGFFGMNVP--LPLTDEPHAWLYISLASAGLWIVLSLLLRKIAKKS 5xdq.2    --------------------LYRLKFKESFAEMNRSTNE------------------------- ``` | | | | | | | | | | | | | | | | | | | | | | | | | | | | | | | | | | | | | | | | | | | | | | | | | |
|  | 5wau.1.Q | Cytochrome c oxidase subunit 4 isoform 1, mitochondrial  *Crystal Structure of CO-bound Cytochrome c Oxidase determined by Synchrotron X-Ray Crystallography at 100 K* | 0.00 |  | 0.00 | 0.06 | 261-277 | X-ray | 1.95 | hetero-oligomer | 4 x HEA, 2 x CU, 2 x MG, 8 x PGV, 6 x TGL, 1 x FME, 2 x CUA, 8 x CHD, 6 x PEK, 4 x CDL, 4 x DMU, 2 x PSC, 2 x ZN, 2 x SAC | HHblits | 0.22 |
| ``` target    MVLEKQLGNGCTWIDLDLGKLNKLEDLSEIYGLDKETIEYALDRNERAHMDYHRESETVTFIYNVLDVKKDKAYYETFPM 5wau.1    --------------------------------------------------------------------------------  target    TFIVEHRRLITISNTKNAYVIEQMTRYLENHDTLSIYKFLFASLEIISNAYYPVIEQMDKSRDEVNDLLRQRTTKKNLFV 5wau.1    --------------------------------------------------------------------------------  target    LSDLETGMVYLTAAAKQNRILLEHIQGHALYRSFDEIEREQFDDAMIEAHQLVSMTDLISQILQQLSASYNNILNNNLND 5wau.1    --------------------------------------------------------------------------------  target    NLTTLTIISVLLAVLAVVTGFFGMNVP--LPLTDEPHAWLYISLASAGLWIVLSLLLRKIAKKS 5wau.1    --------------------LYRLKFKESFAEMNRSTNE------------------------- ``` | | | | | | | | | | | | | | | | | | | | | | | | | | | | | | | | | | | | | | | | | | | | | | | | | |
|  | 5x19.2.D | Cytochrome c oxidase subunit 4 isoform 1, mitochondrial  *CO bound cytochrome c oxidase at 100 micro sec after pump laser irradiation to release CO from O2 reduction center* | 0.00 |  | 0.00 | 0.06 | 261-277 | X-ray | 2.20 | hetero-1-1-1-1-1-1-… | 2 x HEA, 1 x CU, 1 x MG, 4 x PGV, 1 x CUA, 3 x TGL, 1 x PSC, 2 x CDL, 3 x CHD, 1 x ZN, 3 x PEK, 1 x DMU | HHblits | 0.22 |
| ``` target    MVLEKQLGNGCTWIDLDLGKLNKLEDLSEIYGLDKETIEYALDRNERAHMDYHRESETVTFIYNVLDVKKDKAYYETFPM 5x19.2    --------------------------------------------------------------------------------  target    TFIVEHRRLITISNTKNAYVIEQMTRYLENHDTLSIYKFLFASLEIISNAYYPVIEQMDKSRDEVNDLLRQRTTKKNLFV 5x19.2    --------------------------------------------------------------------------------  target    LSDLETGMVYLTAAAKQNRILLEHIQGHALYRSFDEIEREQFDDAMIEAHQLVSMTDLISQILQQLSASYNNILNNNLND 5x19.2    --------------------------------------------------------------------------------  target    NLTTLTIISVLLAVLAVVTGFFGMNVP--LPLTDEPHAWLYISLASAGLWIVLSLLLRKIAKKS 5x19.2    --------------------LYRLKFKESFAEMNRSTNE------------------------- ``` | | | | | | | | | | | | | | | | | | | | | | | | | | | | | | | | | | | | | | | | | | | | | | | | | |
|  | 5x1b.2.D | Cytochrome c oxidase subunit 4 isoform 1, mitochondrial  *CO bound cytochrome c oxidase at 20 nsec after pump laser irradiation to release CO from O2 reduction center* | 0.00 |  | 0.00 | 0.06 | 261-277 | X-ray | 2.40 | hetero-1-1-1-1-1-1-… | 2 x HEA, 1 x CU, 1 x MG, 3 x PGV, 3 x TGL, 1 x CUA, 4 x CHD, 3 x PEK, 2 x CDL, 1 x PSC, 1 x ZN, 1 x DMU | HHblits | 0.22 |
| ``` target    MVLEKQLGNGCTWIDLDLGKLNKLEDLSEIYGLDKETIEYALDRNERAHMDYHRESETVTFIYNVLDVKKDKAYYETFPM 5x1b.2    --------------------------------------------------------------------------------  target    TFIVEHRRLITISNTKNAYVIEQMTRYLENHDTLSIYKFLFASLEIISNAYYPVIEQMDKSRDEVNDLLRQRTTKKNLFV 5x1b.2    --------------------------------------------------------------------------------  target    LSDLETGMVYLTAAAKQNRILLEHIQGHALYRSFDEIEREQFDDAMIEAHQLVSMTDLISQILQQLSASYNNILNNNLND 5x1b.2    --------------------------------------------------------------------------------  target    NLTTLTIISVLLAVLAVVTGFFGMNVP--LPLTDEPHAWLYISLASAGLWIVLSLLLRKIAKKS 5x1b.2    --------------------LYRLKFKESFAEMNRSTNE------------------------- ``` | | | | | | | | | | | | | | | | | | | | | | | | | | | | | | | | | | | | | | | | | | | | | | | | | |
|  | 5x1f.2.D | Cytochrome c oxidase subunit 4 isoform 1, mitochondrial  *CO bound cytochrome c oxidase without pump laser irradiation at 278K* | 0.00 |  | 0.00 | 0.06 | 261-277 | X-ray | 2.20 | hetero-1-1-1-1-1-1-… | 2 x HEA, 1 x CU, 1 x MG, 4 x PGV, 3 x TGL, 1 x CUA, 1 x PSC, 2 x CDL, 3 x CHD, 1 x ZN, 1 x PEK, 1 x DMU | HHblits | 0.22 |
| ``` target    MVLEKQLGNGCTWIDLDLGKLNKLEDLSEIYGLDKETIEYALDRNERAHMDYHRESETVTFIYNVLDVKKDKAYYETFPM 5x1f.2    --------------------------------------------------------------------------------  target    TFIVEHRRLITISNTKNAYVIEQMTRYLENHDTLSIYKFLFASLEIISNAYYPVIEQMDKSRDEVNDLLRQRTTKKNLFV 5x1f.2    --------------------------------------------------------------------------------  target    LSDLETGMVYLTAAAKQNRILLEHIQGHALYRSFDEIEREQFDDAMIEAHQLVSMTDLISQILQQLSASYNNILNNNLND 5x1f.2    --------------------------------------------------------------------------------  target    NLTTLTIISVLLAVLAVVTGFFGMNVP--LPLTDEPHAWLYISLASAGLWIVLSLLLRKIAKKS 5x1f.2    --------------------LYRLKFKESFAEMNRSTNE------------------------- ``` | | | | | | | | | | | | | | | | | | | | | | | | | | | | | | | | | | | | | | | | | | | | | | | | | |
|  | 5zcp.2.D | Cytochrome c oxidase subunit 4 isoform 1, mitochondrial  *azide-bound cytochrome c oxidase structure determined using the crystals exposed to 20 mM azide solution for 2 days* | 0.00 |  | 0.00 | 0.06 | 261-277 | X-ray | 1.65 | hetero-1-1-1-1-1-1-… | 3 x CDL, 2 x HEA, 1 x CU, 1 x MG, 2 x AZI, 4 x PGV, 3 x TGL, 1 x PSC, 1 x CUA, 3 x CHD, 4 x DMU, 2 x PEK, 1 x ZN | HHblits | 0.22 |
| ``` target    MVLEKQLGNGCTWIDLDLGKLNKLEDLSEIYGLDKETIEYALDRNERAHMDYHRESETVTFIYNVLDVKKDKAYYETFPM 5zcp.2    --------------------------------------------------------------------------------  target    TFIVEHRRLITISNTKNAYVIEQMTRYLENHDTLSIYKFLFASLEIISNAYYPVIEQMDKSRDEVNDLLRQRTTKKNLFV 5zcp.2    --------------------------------------------------------------------------------  target    LSDLETGMVYLTAAAKQNRILLEHIQGHALYRSFDEIEREQFDDAMIEAHQLVSMTDLISQILQQLSASYNNILNNNLND 5zcp.2    --------------------------------------------------------------------------------  target    NLTTLTIISVLLAVLAVVTGFFGMNVP--LPLTDEPHAWLYISLASAGLWIVLSLLLRKIAKKS 5zcp.2    --------------------LYRLKFKESFAEMNRSTNE------------------------- ``` | | | | | | | | | | | | | | | | | | | | | | | | | | | | | | | | | | | | | | | | | | | | | | | | | |
|  | 5zcq.2.D | Cytochrome c oxidase subunit 4 isoform 1, mitochondrial  *Azide-bound cytochrome c oxidase structure determined using the crystals exposed to 10 mM azide solution for 2 days* | 0.00 |  | 0.00 | 0.06 | 261-277 | X-ray | 1.65 | hetero-1-1-1-1-1-1-… | 2 x HEA, 1 x CU, 1 x MG, 2 x AZI, 4 x PGV, 3 x TGL, 1 x CUA, 1 x PSC, 3 x CHD, 2 x CDL, 4 x DMU, 3 x PEK, 1 x ZN | HHblits | 0.22 |
| ``` target    MVLEKQLGNGCTWIDLDLGKLNKLEDLSEIYGLDKETIEYALDRNERAHMDYHRESETVTFIYNVLDVKKDKAYYETFPM 5zcq.2    --------------------------------------------------------------------------------  target    TFIVEHRRLITISNTKNAYVIEQMTRYLENHDTLSIYKFLFASLEIISNAYYPVIEQMDKSRDEVNDLLRQRTTKKNLFV 5zcq.2    --------------------------------------------------------------------------------  target    LSDLETGMVYLTAAAKQNRILLEHIQGHALYRSFDEIEREQFDDAMIEAHQLVSMTDLISQILQQLSASYNNILNNNLND 5zcq.2    --------------------------------------------------------------------------------  target    NLTTLTIISVLLAVLAVVTGFFGMNVP--LPLTDEPHAWLYISLASAGLWIVLSLLLRKIAKKS 5zcq.2    --------------------LYRLKFKESFAEMNRSTNE------------------------- ``` | | | | | | | | | | | | | | | | | | | | | | | | | | | | | | | | | | | | | | | | | | | | | | | | | |
|  | 5z86.2.D | Cytochrome c oxidase subunit 4 isoform 1, mitochondrial  *azide-bound cytochrome c oxidase structure determined using the crystals exposed to 20 mM azide solution for 3 days* | 0.00 |  | 0.00 | 0.06 | 261-277 | X-ray | 1.85 | hetero-1-1-1-1-1-1-… | 2 x HEA, 1 x CU, 1 x MG, 2 x AZI, 4 x PGV, 3 x TGL, 1 x CUA, 1 x PSC, 3 x CHD, 3 x PEK, 2 x CDL, 4 x DMU, 1 x ZN | HHblits | 0.22 |
| ``` target    MVLEKQLGNGCTWIDLDLGKLNKLEDLSEIYGLDKETIEYALDRNERAHMDYHRESETVTFIYNVLDVKKDKAYYETFPM 5z86.2    --------------------------------------------------------------------------------  target    TFIVEHRRLITISNTKNAYVIEQMTRYLENHDTLSIYKFLFASLEIISNAYYPVIEQMDKSRDEVNDLLRQRTTKKNLFV 5z86.2    --------------------------------------------------------------------------------  target    LSDLETGMVYLTAAAKQNRILLEHIQGHALYRSFDEIEREQFDDAMIEAHQLVSMTDLISQILQQLSASYNNILNNNLND 5z86.2    --------------------------------------------------------------------------------  target    NLTTLTIISVLLAVLAVVTGFFGMNVP--LPLTDEPHAWLYISLASAGLWIVLSLLLRKIAKKS 5z86.2    --------------------LYRLKFKESFAEMNRSTNE------------------------- ``` | | | | | | | | | | | | | | | | | | | | | | | | | | | | | | | | | | | | | | | | | | | | | | | | | |
|  | 5z85.2.D | Cytochrome c oxidase subunit 4 isoform 1, mitochondrial  *The structure of azide-bound cytochrome c oxidase determined using the another batch crystals exposed to 20 mM azide solution for 2 days* | 0.00 |  | 0.00 | 0.06 | 261-277 | X-ray | 1.85 | hetero-1-1-1-1-1-1-… | 2 x HEA, 1 x CU, 1 x MG, 2 x AZI, 4 x PGV, 3 x TGL, 1 x CUA, 1 x PSC, 3 x CHD, 3 x PEK, 2 x CDL, 4 x DMU, 1 x ZN | HHblits | 0.22 |
| ``` target    MVLEKQLGNGCTWIDLDLGKLNKLEDLSEIYGLDKETIEYALDRNERAHMDYHRESETVTFIYNVLDVKKDKAYYETFPM 5z85.2    --------------------------------------------------------------------------------  target    TFIVEHRRLITISNTKNAYVIEQMTRYLENHDTLSIYKFLFASLEIISNAYYPVIEQMDKSRDEVNDLLRQRTTKKNLFV 5z85.2    --------------------------------------------------------------------------------  target    LSDLETGMVYLTAAAKQNRILLEHIQGHALYRSFDEIEREQFDDAMIEAHQLVSMTDLISQILQQLSASYNNILNNNLND 5z85.2    --------------------------------------------------------------------------------  target    NLTTLTIISVLLAVLAVVTGFFGMNVP--LPLTDEPHAWLYISLASAGLWIVLSLLLRKIAKKS 5z85.2    --------------------LYRLKFKESFAEMNRSTNE------------------------- ``` | | | | | | | | | | | | | | | | | | | | | | | | | | | | | | | | | | | | | | | | | | | | | | | | | |
|  | 5z84.2.D | Cytochrome c oxidase subunit 4 isoform 1, mitochondrial  *The structure of azide-bound cytochrome c oxidase determined using the crystals exposed to 20 mM azide solution for 4 days* | 0.00 |  | 0.00 | 0.06 | 261-277 | X-ray | 1.85 | hetero-1-1-1-1-1-1-… | 3 x CDL, 2 x HEA, 1 x CU, 1 x MG, 2 x AZI, 3 x PGV, 3 x TGL, 1 x CUA, 1 x PSC, 3 x CHD, 4 x DMU, 3 x PEK, 1 x ZN | HHblits | 0.22 |
| ``` target    MVLEKQLGNGCTWIDLDLGKLNKLEDLSEIYGLDKETIEYALDRNERAHMDYHRESETVTFIYNVLDVKKDKAYYETFPM 5z84.2    --------------------------------------------------------------------------------  target    TFIVEHRRLITISNTKNAYVIEQMTRYLENHDTLSIYKFLFASLEIISNAYYPVIEQMDKSRDEVNDLLRQRTTKKNLFV 5z84.2    --------------------------------------------------------------------------------  target    LSDLETGMVYLTAAAKQNRILLEHIQGHALYRSFDEIEREQFDDAMIEAHQLVSMTDLISQILQQLSASYNNILNNNLND 5z84.2    --------------------------------------------------------------------------------  target    NLTTLTIISVLLAVLAVVTGFFGMNVP--LPLTDEPHAWLYISLASAGLWIVLSLLLRKIAKKS 5z84.2    --------------------LYRLKFKESFAEMNRSTNE------------------------- ``` | | | | | | | | | | | | | | | | | | | | | | | | | | | | | | | | | | | | | | | | | | | | | | | | | |
|  | 5w97.1.Q | Cytochrome c oxidase subunit 4 isoform 1, mitochondrial  *Crystal Structure of CO-bound Cytochrome c Oxidase determined by Serial Femtosecond X-Ray Crystallography at Room Temperature* | 0.00 |  | 0.00 | 0.06 | 261-277 | X-ray | 2.30 | hetero-2-2-2-2-2-2-… | 4 x HEA, 2 x CU, 2 x MG, 8 x PGV, 6 x TGL, 2 x CUA, 2 x PSC, 8 x CHD, 6 x PEK, 4 x CDL, 4 x DMU, 2 x ZN | HHblits | 0.22 |
| ``` target    MVLEKQLGNGCTWIDLDLGKLNKLEDLSEIYGLDKETIEYALDRNERAHMDYHRESETVTFIYNVLDVKKDKAYYETFPM 5w97.1    --------------------------------------------------------------------------------  target    TFIVEHRRLITISNTKNAYVIEQMTRYLENHDTLSIYKFLFASLEIISNAYYPVIEQMDKSRDEVNDLLRQRTTKKNLFV 5w97.1    --------------------------------------------------------------------------------  target    LSDLETGMVYLTAAAKQNRILLEHIQGHALYRSFDEIEREQFDDAMIEAHQLVSMTDLISQILQQLSASYNNILNNNLND 5w97.1    --------------------------------------------------------------------------------  target    NLTTLTIISVLLAVLAVVTGFFGMNVP--LPLTDEPHAWLYISLASAGLWIVLSLLLRKIAKKS 5w97.1    --------------------LYRLKFKESFAEMNRSTNE------------------------- ``` | | | | | | | | | | | | | | | | | | | | | | | | | | | | | | | | | | | | | | | | | | | | | | | | | |
|  | 6nmf.1.Q | Cytochrome c oxidase subunit 4 isoform 1, mitochondrial  *SFX structure of reduced cytochrome c oxidase at room temperature* | 0.00 |  | 0.00 | 0.06 | 261-277 | X-ray | 2.80 | hetero-2-2-2-2-2-2-… | 4 x HEA, 2 x CU, 2 x MG, 8 x PGV, 6 x TGL, 4 x CDL, 2 x CUA, 8 x CHD, 2 x PSC, 4 x DMU, 6 x PEK, 2 x ZN | HHblits | 0.22 |
| ``` target    MVLEKQLGNGCTWIDLDLGKLNKLEDLSEIYGLDKETIEYALDRNERAHMDYHRESETVTFIYNVLDVKKDKAYYETFPM 6nmf.1    --------------------------------------------------------------------------------  target    TFIVEHRRLITISNTKNAYVIEQMTRYLENHDTLSIYKFLFASLEIISNAYYPVIEQMDKSRDEVNDLLRQRTTKKNLFV 6nmf.1    --------------------------------------------------------------------------------  target    LSDLETGMVYLTAAAKQNRILLEHIQGHALYRSFDEIEREQFDDAMIEAHQLVSMTDLISQILQQLSASYNNILNNNLND 6nmf.1    --------------------------------------------------------------------------------  target    NLTTLTIISVLLAVLAVVTGFFGMNVP--LPLTDEPHAWLYISLASAGLWIVLSLLLRKIAKKS 6nmf.1    --------------------LYRLKFKESFAEMNRSTNE------------------------- ``` | | | | | | | | | | | | | | | | | | | | | | | | | | | | | | | | | | | | | | | | | | | | | | | | | |
|  | 1cun.1.B | PROTEIN (ALPHA SPECTRIN)  *CRYSTAL STRUCTURE OF REPEATS 16 AND 17 OF CHICKEN BRAIN ALPHA SPECTRIN* | 0.00 |  | 13.33 | 0.05 | 135-149 | X-ray | 2.00 | homo-trimer |  | HHblits | 0.27 |
| ``` target    MVLEKQLGNGCTWIDLDLGKLNKLEDLSEIYGLDKETIEYALDRNERAHMDYHRESETVTFIYNVLDVKKDKAYYETFPM 1cun.1    --------------------------------------------------------------------------------  target    TFIVEHRRLITISNTKNAYVIEQMTRYLENHDTLSIYKFLFASLEIISNAYYPVIEQMDKSRDEVNDLLRQRTTKKNLFV 1cun.1    ------------------------------------------------------MDDEESWIKEKKLLV-----------  target    LSDLETGMVYLTAAAKQNRILLEHIQGHALYRSFDEIEREQFDDAMIEAHQLVSMTDLISQILQQLSASYNNILNNNLND 1cun.1    --------------------------------------------------------------------------------  target    NLTTLTIISVLLAVLAVVTGFFGMNVPLPLTDEPHAWLYISLASAGLWIVLSLLLRKIAKKS 1cun.1    -------------------------------------------------------------- ``` | | | | | | | | | | | | | | | | | | | | | | | | | | | | | | | | | | | | | | | | | | | | | | | | | |
|  | 1cun.1.A | PROTEIN (ALPHA SPECTRIN)  *CRYSTAL STRUCTURE OF REPEATS 16 AND 17 OF CHICKEN BRAIN ALPHA SPECTRIN* | 0.00 |  | 13.33 | 0.05 | 135-149 | X-ray | 2.00 | homo-trimer |  | HHblits | 0.27 |
| ``` target    MVLEKQLGNGCTWIDLDLGKLNKLEDLSEIYGLDKETIEYALDRNERAHMDYHRESETVTFIYNVLDVKKDKAYYETFPM 1cun.1    --------------------------------------------------------------------------------  target    TFIVEHRRLITISNTKNAYVIEQMTRYLENHDTLSIYKFLFASLEIISNAYYPVIEQMDKSRDEVNDLLRQRTTKKNLFV 1cun.1    ------------------------------------------------------MDDEESWIKEKKLLV-----------  target    LSDLETGMVYLTAAAKQNRILLEHIQGHALYRSFDEIEREQFDDAMIEAHQLVSMTDLISQILQQLSASYNNILNNNLND 1cun.1    --------------------------------------------------------------------------------  target    NLTTLTIISVLLAVLAVVTGFFGMNVPLPLTDEPHAWLYISLASAGLWIVLSLLLRKIAKKS 1cun.1    -------------------------------------------------------------- ``` | | | | | | | | | | | | | | | | | | | | | | | | | | | | | | | | | | | | | | | | | | | | | | | | | |

**Export Alignment**
  
FASTA format
Clustal Format
PNG Image

**Secondary Structure**
  
None
DSSP
PSIPRED
SSpro

**Colour Scheme** 


Fade Mismatches
Enhance Mismatches

Clustal
Hydrophobic
Size
Charged
Polar
Proline
Ser/Thr
Cysteine
Aliphatic
Aromatic
QMEAN
Indels
Chain
Unique Chain
Rainbow
Structure
No Colour

 Use QMEANBrane values

|  |  |  |  |
| --- | --- | --- | --- |
| Background |  |  |  |

**3D Viewer**  
NGL
PV

FASTA
Multi FASTA
ClustalW
PNG
