# Supplementary material for: A bacterial pan-genome makes gene essentiality strain-dependent and evolvable
Source: Nat Microbiol. 2022 Sep 12;7(10):1580–92. doi: 10.1038/s41564-022-01208-7 (PMC9519441; doi:10.1038/s41564-022-01208-7)
Supplement: Source Data Fig. 4 — Swiss models data, genetic interactions (a subset of Supplementary Data 11), growth curves data and RNA-seq data for the two transporters (taken from Supplementary Data 6). [file 41564_2022_1208_MOESM8_ESM.zip › Fig_4a_models/SP_1751_swissmodel/SP_1751/models.html]

Untitled Project | Models


SWISS-MODEL

### Untitled Project

### Created: May 16, 2021, 3:29 a.m. at 03:29

- Templates
- Models

Order by:
GMQE
QMEAN4
Oligo State
Ligands
Seq Identity
Similarity
Coverage

Model 01

- PDB Format *(Display)*
- JSON Format *(Display)*
- Model Report *(Display)*

| Oligo-State | Ligands | GMQE | QMEAN |
| --- | --- | --- | --- |
| Homo-pentamer (matching prediction) | 1 x MG | 0.64 | -2.82 |
|  | 1 x MAGNESIUM ION  |  |  | | --- | --- | | MG.34: | 3 residues within 4Å:   - Chain D:   N.231,- Chain E:     N.232,     N.235  No protein-ligand interaction detected (PLIP) |  --- | | |

| Global Quality Estimate | Local Quality Estimate | Comparison |
| --- | --- | --- |
|  |  | |

Membrane annotation has been transferred from template. 
  


Show / Hide

| Template | Seq Identity | Coverage | Description |  |
| --- | --- | --- | --- | --- |
| 4ev6.1.A | 20.07% |  | Magnesium transport protein CorA The complete structure of CorA magnesium transporter from Methanocaldococcus jannaschii |  |

|  |  |
| --- | --- |
| **Biounit Oligo State** | Homo-pentamer |
| **QSQE** | 0.49 |
| **Method** | X-ray, 3.20 Å |
| **Seq Similarity** | 0.31 |
| **Coverage** | 0.94 |
| **Range** | 7-297 |
|

| Ligand | Added to Model | Description |
| --- | --- | --- |
| MG | ✓ | MAGNESIUM ION |
| MG | ✕ - Not in contact with model. | MAGNESIUM ION |
| MG | ✕ - Not in contact with model. | MAGNESIUM ION |
| MG | ✕ - Binding site not conserved. | MAGNESIUM ION |
| MG | ✕ - Not in contact with model. | MAGNESIUM ION |
| MG | ✕ - Not in contact with model. | MAGNESIUM ION |
| MG | ✕ - Not in contact with model. | MAGNESIUM ION |
| MG | ✕ - Binding site not conserved. | MAGNESIUM ION |
| MG | ✕ - Not in contact with model. | MAGNESIUM ION |
| MG | ✕ - Binding site not conserved. | MAGNESIUM ION |
| MG | ✕ - Not in contact with model. | MAGNESIUM ION |
| MG | ✕ - Not in contact with model. | MAGNESIUM ION |
| MG | ✕ - Binding site not conserved. | MAGNESIUM ION |
| MG | ✕ - Not in contact with model. | MAGNESIUM ION |
| MG | ✕ - Binding site not conserved. | MAGNESIUM ION |
| MG | ✕ - Binding site not conserved. | MAGNESIUM ION |
| MG | ✕ - Binding site not conserved. | MAGNESIUM ION |
| MG | ✕ - Binding site not conserved. | MAGNESIUM ION |
| MG | ✕ - Not in contact with model. | MAGNESIUM ION |
| MG | ✕ - Binding site not conserved. | MAGNESIUM ION |
| MG | ✕ - Not in contact with model. | MAGNESIUM ION |
| MG | ✕ - Binding site not conserved. | MAGNESIUM ION |
| MG | ✕ - Binding site not conserved. | MAGNESIUM ION |
| MG | ✕ - Binding site not conserved. | MAGNESIUM ION |
| MG | ✕ - Not in contact with model. | MAGNESIUM ION |
| MG | ✕ - Binding site not conserved. | MAGNESIUM ION |
| MG | ✕ - Binding site not conserved. | MAGNESIUM ION |
| MG | ✕ - Binding site not conserved. | MAGNESIUM ION |
| MG | ✕ - Not in contact with model. | MAGNESIUM ION |
| MG | ✕ - Binding site not conserved. | MAGNESIUM ION |
| MG | ✕ - Binding site not conserved. | MAGNESIUM ION |
| MG | ✕ - Binding site not conserved. | MAGNESIUM ION |
| UMQ | ✕ - Binding site not conserved. | UNDECYL-MALTOSIDE |
| UMQ | ✕ - Binding site not conserved. | UNDECYL-MALTOSIDE |
| UMQ | ✕ - Binding site not conserved. | UNDECYL-MALTOSIDE |
| UMQ | ✕ - Binding site not conserved. | UNDECYL-MALTOSIDE |
| UMQ | ✕ - Binding site not conserved. | UNDECYL-MALTOSIDE |
| UMQ | ✕ - Binding site not conserved. | UNDECYL-MALTOSIDE |
| UMQ | ✕ - Binding site not conserved. | UNDECYL-MALTOSIDE |
| UMQ | ✕ - Binding site not conserved. | UNDECYL-MALTOSIDE |

Model-Template Alignment

|  |  |  |
| --- | --- | --- |
|  |  |  |

Cartoon

- Cartoon
- Tube
- Trace
- Lines
- Ball+Stick
- Licorice
- Hyperball
- Rope
- Surface
- Spacefill
- Outline
- Fog

###### Background

- Transparent

###### Resolution

- Low
- Medium
- High
- Extreme

##### Click model image to view in 3D

##### Click model image to view in 3D

×

### Delete Model - ""

Are you sure you want to delete this model?  
(This really can't be undone!)

Close
Delete Model

**Export Alignment**
  
FASTA format
Clustal Format
PNG Image

**Secondary Structure**
  
None
DSSP
PSIPRED
SSpro

**Colour Scheme** 


Fade Mismatches
Enhance Mismatches

Clustal
Hydrophobic
Size
Charged
Polar
Proline
Ser/Thr
Cysteine
Aliphatic
Aromatic
QMEAN
Indels
Chain
Unique Chain
Rainbow
Structure
No Colour

 Use QMEANBrane values

|  |  |  |  |
| --- | --- | --- | --- |
| Background |  |  |  |

**3D Viewer**  
NGL
PV

FASTA
Multi FASTA
ClustalW
PNG
