# Supplementary material for: A bacterial pan-genome makes gene essentiality strain-dependent and evolvable
Source: Nat Microbiol. 2022 Sep 12;7(10):1580–92. doi: 10.1038/s41564-022-01208-7 (PMC9519441; doi:10.1038/s41564-022-01208-7)
Supplement: Source Data Fig. 4 — Swiss models data, genetic interactions (a subset of Supplementary Data 11), growth curves data and RNA-seq data for the two transporters (taken from Supplementary Data 6). [file 41564_2022_1208_MOESM8_ESM.zip › Fig_4a_models/SP_0185_swissmodel/SP_0185/templates.html]

SP\_0185 | Templates


SWISS-MODEL

### SP\_0185

### Created: May 24, 2021, 4:25 a.m. at 04:25

- Templates
- Models

Models | Name | Description | GMQE | QSQE | Seq Id | Coverage | Range | Method | Resolution | Oligo-state | Ligands | Found by | Seq Similarity ||  | 4ev6.1.A | Magnesium transport protein CorA  *The complete structure of CorA magnesium transporter from Methanocaldococcus jannaschii* | 0.71 | 0.54 | 26.55 | 0.92 | 17-314 | X-ray | 3.20 | homo-pentamer | 8 x UMQ, 28 x MG | HHblits | 0.34 |
| ``` target    MKQVFLSTTTEFKEIDTLEPGTWINLVNPTQNESLEIANTFDIDIADLRAPLDAEEMSRITIEDEYTLIIVDVPVTEERN 4ev6.1    ----------------EDYRLIWIDCYDPKDEELYKLSKKIGISVSDLQIGLDEQEIPRVEEDEDFYLIIYKAPLFEE--  target    NRTYYVTIPLGIIITEETIITTCLEPLPVLDVFINRRLRNFYT----FMRSRFIFQILYRNAELYLTALRSIDRKSEQIE 4ev6.1    ---DITTTSLGIYIKNNLLLTIHSDKIKAIGRLHKLISTKKPRIVFERGIGFLLYHILNEITRSYSRILMNLEDELEELE  target    SQLHQSTRNEELIELMELEKTIVYFKASLKTNERVIKKLTSSTSNIKKYLEDEDLLEDTLIETQQAIEMADIYGNVLHSM 4ev6.1    DKLLAGYDREVMEKILGLRKTLVYFHKSLIANRDVLVLLKRKYLPI-TTKEDRENFEDLYYDTLQLIDMSATYREVLTSM  target    TETFASIISNNQNNIMKTLALVTIVMSIPTMVFSAYGMNFKDNEIPLNGEPNAFWLIVFIAFAMSVSLTLYLIHKKWF 4ev6.1    MDITLSLENIKMNQIMKILTMVTTIFAVPMWITGIYGMNFSY--LPLANNPQGFWLVMALMVVIIMIFVYIFRRSGWI ``` | | | | | | | | | | | | | | | | | | | | | | | | | | | | | | | | | | | | | | | | | | | | | | | | | |
|  | 4ev6.1.B | Magnesium transport protein CorA  *The complete structure of CorA magnesium transporter from Methanocaldococcus jannaschii* | 0.70 | 0.54 | 26.55 | 0.92 | 17-314 | X-ray | 3.20 | homo-pentamer | 8 x UMQ, 28 x MG | HHblits | 0.34 |
| ``` target    MKQVFLSTTTEFKEIDTLEPGTWINLVNPTQNESLEIANTFDIDIADLRAPLDAEEMSRITIEDEYTLIIVDVPVTEERN 4ev6.1    ----------------EDYRLIWIDCYDPKDEELYKLSKKIGISVSDLQIGLDEQEIPRVEEDEDFYLIIYKAPLFEE--  target    NRTYYVTIPLGIIITEETIITTCLEPLPVLDVFINRRLRNFYT----FMRSRFIFQILYRNAELYLTALRSIDRKSEQIE 4ev6.1    ---DITTTSLGIYIKNNLLLTIHSDKIKAIGRLHKLISTKKPRIVFERGIGFLLYHILNEITRSYSRILMNLEDELEELE  target    SQLHQSTRNEELIELMELEKTIVYFKASLKTNERVIKKLTSSTSNIKKYLEDEDLLEDTLIETQQAIEMADIYGNVLHSM 4ev6.1    DKLLAGYDREVMEKILGLRKTLVYFHKSLIANRDVLVLLKRKYLPI-TTKEDRENFEDLYYDTLQLIDMSATYREVLTSM  target    TETFASIISNNQNNIMKTLALVTIVMSIPTMVFSAYGMNFKDNEIPLNGEPNAFWLIVFIAFAMSVSLTLYLIHKKWF 4ev6.1    MDITLSLENIKMNQIMKILTMVTTIFAVPMWITGIYGMNFSY--LPLANNPQGFWLVMALMVVIIMIFVYIFRRSGWI ``` | | | | | | | | | | | | | | | | | | | | | | | | | | | | | | | | | | | | | | | | | | | | | | | | | |
|  | 4ev6.1.C | Magnesium transport protein CorA  *The complete structure of CorA magnesium transporter from Methanocaldococcus jannaschii* | 0.71 | 0.54 | 26.55 | 0.92 | 17-314 | X-ray | 3.20 | homo-pentamer | 8 x UMQ, 28 x MG | HHblits | 0.34 |
| ``` target    MKQVFLSTTTEFKEIDTLEPGTWINLVNPTQNESLEIANTFDIDIADLRAPLDAEEMSRITIEDEYTLIIVDVPVTEERN 4ev6.1    ----------------EDYRLIWIDCYDPKDEELYKLSKKIGISVSDLQIGLDEQEIPRVEEDEDFYLIIYKAPLFEE--  target    NRTYYVTIPLGIIITEETIITTCLEPLPVLDVFINRRLRNFYT----FMRSRFIFQILYRNAELYLTALRSIDRKSEQIE 4ev6.1    ---DITTTSLGIYIKNNLLLTIHSDKIKAIGRLHKLISTKKPRIVFERGIGFLLYHILNEITRSYSRILMNLEDELEELE  target    SQLHQSTRNEELIELMELEKTIVYFKASLKTNERVIKKLTSSTSNIKKYLEDEDLLEDTLIETQQAIEMADIYGNVLHSM 4ev6.1    DKLLAGYDREVMEKILGLRKTLVYFHKSLIANRDVLVLLKRKYLPI-TTKEDRENFEDLYYDTLQLIDMSATYREVLTSM  target    TETFASIISNNQNNIMKTLALVTIVMSIPTMVFSAYGMNFKDNEIPLNGEPNAFWLIVFIAFAMSVSLTLYLIHKKWF 4ev6.1    MDITLSLENIKMNQIMKILTMVTTIFAVPMWITGIYGMNFSY--LPLANNPQGFWLVMALMVVIIMIFVYIFRRSGWI ``` | | | | | | | | | | | | | | | | | | | | | | | | | | | | | | | | | | | | | | | | | | | | | | | | | |
|  | 4ev6.1.D | Magnesium transport protein CorA  *The complete structure of CorA magnesium transporter from Methanocaldococcus jannaschii* | 0.71 | 0.54 | 26.55 | 0.92 | 17-314 | X-ray | 3.20 | homo-pentamer | 8 x UMQ, 28 x MG | HHblits | 0.34 |
| ``` target    MKQVFLSTTTEFKEIDTLEPGTWINLVNPTQNESLEIANTFDIDIADLRAPLDAEEMSRITIEDEYTLIIVDVPVTEERN 4ev6.1    ----------------EDYRLIWIDCYDPKDEELYKLSKKIGISVSDLQIGLDEQEIPRVEEDEDFYLIIYKAPLFEE--  target    NRTYYVTIPLGIIITEETIITTCLEPLPVLDVFINRRLRNFYT----FMRSRFIFQILYRNAELYLTALRSIDRKSEQIE 4ev6.1    ---DITTTSLGIYIKNNLLLTIHSDKIKAIGRLHKLISTKKPRIVFERGIGFLLYHILNEITRSYSRILMNLEDELEELE  target    SQLHQSTRNEELIELMELEKTIVYFKASLKTNERVIKKLTSSTSNIKKYLEDEDLLEDTLIETQQAIEMADIYGNVLHSM 4ev6.1    DKLLAGYDREVMEKILGLRKTLVYFHKSLIANRDVLVLLKRKYLPI-TTKEDRENFEDLYYDTLQLIDMSATYREVLTSM  target    TETFASIISNNQNNIMKTLALVTIVMSIPTMVFSAYGMNFKDNEIPLNGEPNAFWLIVFIAFAMSVSLTLYLIHKKWF 4ev6.1    MDITLSLENIKMNQIMKILTMVTTIFAVPMWITGIYGMNFSY--LPLANNPQGFWLVMALMVVIIMIFVYIFRRSGWI ``` | | | | | | | | | | | | | | | | | | | | | | | | | | | | | | | | | | | | | | | | | | | | | | | | | |
| ✓ | 4ev6.1.E | Magnesium transport protein CorA  *The complete structure of CorA magnesium transporter from Methanocaldococcus jannaschii* | 0.71 | 0.54 | 26.55 | 0.92 | 17-314 | X-ray | 3.20 | homo-pentamer | 8 x UMQ, 28 x MG | HHblits | 0.34 |
| ``` target    MKQVFLSTTTEFKEIDTLEPGTWINLVNPTQNESLEIANTFDIDIADLRAPLDAEEMSRITIEDEYTLIIVDVPVTEERN 4ev6.1    ----------------EDYRLIWIDCYDPKDEELYKLSKKIGISVSDLQIGLDEQEIPRVEEDEDFYLIIYKAPLFEE--  target    NRTYYVTIPLGIIITEETIITTCLEPLPVLDVFINRRLRNFYT----FMRSRFIFQILYRNAELYLTALRSIDRKSEQIE 4ev6.1    ---DITTTSLGIYIKNNLLLTIHSDKIKAIGRLHKLISTKKPRIVFERGIGFLLYHILNEITRSYSRILMNLEDELEELE  target    SQLHQSTRNEELIELMELEKTIVYFKASLKTNERVIKKLTSSTSNIKKYLEDEDLLEDTLIETQQAIEMADIYGNVLHSM 4ev6.1    DKLLAGYDREVMEKILGLRKTLVYFHKSLIANRDVLVLLKRKYLPI-TTKEDRENFEDLYYDTLQLIDMSATYREVLTSM  target    TETFASIISNNQNNIMKTLALVTIVMSIPTMVFSAYGMNFKDNEIPLNGEPNAFWLIVFIAFAMSVSLTLYLIHKKWF 4ev6.1    MDITLSLENIKMNQIMKILTMVTTIFAVPMWITGIYGMNFSY--LPLANNPQGFWLVMALMVVIIMIFVYIFRRSGWI ``` | | | | | | | | | | | | | | | | | | | | | | | | | | | | | | | | | | | | | | | | | | | | | | | | | |
|  | 4eed.1.C | Magnesium transport protein CorA  *CorA coiled-coil mutant under Mg2+ presence* | 0.61 | 0.54 | 16.84 | 0.95 | 13-314 | X-ray | 3.92 | homo-pentamer | 14 x MG | HHblits | 0.30 |
| ``` target    MKQVFLSTTTEFKEIDTLEPGTWINLVNPTQNE-SLEIANTFDIDIADLRAPLDAEEMSRITIEDEYTLIIVDVPVTEER 4eed.1    ------------LPFRDSSTPTWINITGIHRTDVVQRVGEFFGIHPLVLEDILNVHQRPKVEFFENYVFIVLKMFTYDKN  target    NNRTYYVTIPLGIIITEETIITTCLEPLPVLDVFINRRLRNFY---TFMRSRFIFQILYRNAELYLTALRSIDRKSEQIE 4eed.1    L--HELESEQVSLILTKNCVLMFQEKIGDVFDPVRERIRYNRGIIRKKRADYLLYSLIDALVDDYFVLLEKIDDEIDVLE  target    SQLHQSTRNEELIELMELEKTIVYFKASLKTNERVIKKLTSSTSNIKKYLEDEDLLEDTLIETQQAIEMADIYGNVLHSM 4eed.1    EEVLERPEKETVQRTHQLKRNLVELAATIWPLREVLSSLYRDVPPL-IEKETVPYFRDVYDHTIQIADTVETFRDIVSGL  target    TETFASIISNNQNNIMKTLALVTIVMSIPTMVFSAYGMNFKDNEIPLNGEPNAFWLIVFIAFAMSVSLTLYLIHKKWF 4eed.1    LDVYLSSVSNKTNEVMKVLTIIATIFMPLTFIAGIYGMNFEY--MPELRWKWGYPVVLAVMGVIAVIMVVYFKKKKWL ``` | | | | | | | | | | | | | | | | | | | | | | | | | | | | | | | | | | | | | | | | | | | | | | | | | |
|  | 4eeb.1.A | Magnesium transport protein CorA  *CorA coiled-coil mutant under Mg2+ absence* | 0.62 | 0.42 | 16.84 | 0.95 | 13-314 | X-ray | 3.80 | homo-pentamer | 7 x CS | HHblits | 0.30 |
| ``` target    MKQVFLSTTTEFKEIDTLEPGTWINLVNPTQNE-SLEIANTFDIDIADLRAPLDAEEMSRITIEDEYTLIIVDVPVTEER 4eeb.1    ------------LPFRDSSTPTWINITGIHRTDVVQRVGEFFGIHPLVLEDILNVHQRPKVEFFENYVFIVLKMFTYDKN  target    NNRTYYVTIPLGIIITEETIITTCLEPLPVLDVFINRRLRNFY---TFMRSRFIFQILYRNAELYLTALRSIDRKSEQIE 4eeb.1    L--HELESEQVSLILTKNCVLMFQEKIGDVFDPVRERIRYNRGIIRKKRADYLLYSLIDALVDDYFVLLEKIDDEIDVLE  target    SQLHQSTRNEELIELMELEKTIVYFKASLKTNERVIKKLTSSTSNIKKYLEDEDLLEDTLIETQQAIEMADIYGNVLHSM 4eeb.1    EEVLERPEKETVQRTHQLKRNLVELAATIWPLREVLSSLYRDVPPL-IEKETVPYFRDVYDHTIQIADTVETFRDIVSGL  target    TETFASIISNNQNNIMKTLALVTIVMSIPTMVFSAYGMNFKDNEIPLNGEPNAFWLIVFIAFAMSVSLTLYLIHKKWF 4eeb.1    LDVYLSSVSNKTNEVMKVLTIIATIFMPLTFIAGIYGMNFEY--MPELRWKWGYPVVLAVMGVIAVIMVVYFKKKKWL ``` | | | | | | | | | | | | | | | | | | | | | | | | | | | | | | | | | | | | | | | | | | | | | | | | | |
|  | 4eeb.1.B | Magnesium transport protein CorA  *CorA coiled-coil mutant under Mg2+ absence* | 0.62 | 0.42 | 16.84 | 0.95 | 13-314 | X-ray | 3.80 | homo-pentamer | 7 x CS | HHblits | 0.30 |
| ``` target    MKQVFLSTTTEFKEIDTLEPGTWINLVNPTQNE-SLEIANTFDIDIADLRAPLDAEEMSRITIEDEYTLIIVDVPVTEER 4eeb.1    ------------LPFRDSSTPTWINITGIHRTDVVQRVGEFFGIHPLVLEDILNVHQRPKVEFFENYVFIVLKMFTYDKN  target    NNRTYYVTIPLGIIITEETIITTCLEPLPVLDVFINRRLRNFY---TFMRSRFIFQILYRNAELYLTALRSIDRKSEQIE 4eeb.1    L--HELESEQVSLILTKNCVLMFQEKIGDVFDPVRERIRYNRGIIRKKRADYLLYSLIDALVDDYFVLLEKIDDEIDVLE  target    SQLHQSTRNEELIELMELEKTIVYFKASLKTNERVIKKLTSSTSNIKKYLEDEDLLEDTLIETQQAIEMADIYGNVLHSM 4eeb.1    EEVLERPEKETVQRTHQLKRNLVELAATIWPLREVLSSLYRDVPPL-IEKETVPYFRDVYDHTIQIADTVETFRDIVSGL  target    TETFASIISNNQNNIMKTLALVTIVMSIPTMVFSAYGMNFKDNEIPLNGEPNAFWLIVFIAFAMSVSLTLYLIHKKWF 4eeb.1    LDVYLSSVSNKTNEVMKVLTIIATIFMPLTFIAGIYGMNFEY--MPELRWKWGYPVVLAVMGVIAVIMVVYFKKKKWL ``` | | | | | | | | | | | | | | | | | | | | | | | | | | | | | | | | | | | | | | | | | | | | | | | | | |
|  | 2bbj.1.A | divalent cation transport-related protein  *Crystal structure of the CorA Mg2+ transporter* | 0.60 | 0.44 | 16.78 | 0.93 | 18-314 | X-ray | 3.90 | homo-pentamer |  | HHblits | 0.30 |
| ``` target    MKQVFLSTTTEFKEIDTLEPGTWINLVNPTQ-NESLEIANTFDIDIADLRAPLDAEEMSRITIEDEYTLIIVDVPVTEER 2bbj.1    -----------------SSTPTWINITGIHRTDVVQRVGEFFGIHPLVLEDILNVHQRPKVEFFENYVFIVLKMFTYDKN  target    NNRTYYVTIPLGIIITEETIITTCLEPLPVLDVFINRRLRNFY---TFMRSRFIFQILYRNAELYLTALRSIDRKSEQIE 2bbj.1    L--HELESEQVSLILTKNCVLMFQEKIGDVFDPVRERIRYNRGIIRKKRADYLLYSLIDALVDDYFVLLEKIDDEIDVLE  target    SQLHQSTRNEELIELMELEKTIVYFKASLKTNERVIKKLTSSTSNIKKYLEDEDLLEDTLIETQQAIEMADIYGNVLHSM 2bbj.1    EEVLERPEKETVQRTHQLKRNLVELRKTIWPLREVLSSLYRDVPPL-IEKETVPYFRDVYDHTIQIADTVETFRDIVSGL  target    TETFASIISNNQNNIMKTLALVTIVMSIPTMVFSAYGMNFKDNEIPLNGEPNAFWLIVFIAFAMSVSLTLYLIHKKWF 2bbj.1    LDVYLSSVSNKTNEVMKVLTIIATIFMPLTFIAGIYGMNFEY--MPELRWKWGYPVVLAVMGVIAVIMVVYFKKKKWL ``` | | | | | | | | | | | | | | | | | | | | | | | | | | | | | | | | | | | | | | | | | | | | | | | | | |
|  | 2hn2.1.C | Magnesium transport protein corA  *Crystal structure of the CorA Mg2+ transporter homologue from T. maritima in complex with divalent cations* | 0.61 | 0.48 | 16.78 | 0.93 | 18-314 | X-ray | 3.70 | homo-pentamer | 12 x CA | HHblits | 0.30 |
| ``` target    MKQVFLSTTTEFKEIDTLEPGTWINLVNPTQ-NESLEIANTFDIDIADLRAPLDAEEMSRITIEDEYTLIIVDVPVTEER 2hn2.1    -----------------SSTPTWINITGIHRTDVVQRVGEFFGIHPLVLEDILNVHQRPKVEFFENYVFIVLKMFTYDKN  target    NNRTYYVTIPLGIIITEETIITTCLEPLPVLDVFINRRLRNFY---TFMRSRFIFQILYRNAELYLTALRSIDRKSEQIE 2hn2.1    L--HELESEQVSLILTKNCVLMFQEKIGDVFDPVRERIRYNRGIIRKKRADYLLYSLIDALVDDYFVLLEKIDDEIDVLE  target    SQLHQSTRNEELIELMELEKTIVYFKASLKTNERVIKKLTSSTSNIKKYLEDEDLLEDTLIETQQAIEMADIYGNVLHSM 2hn2.1    EEVLERPEKETVQRTHQLKRNLVELRKTIWPLREVLSSLYRDVPPL-IEKETVPYFRDVYDHTIQIADTVETFRDIVSGL  target    TETFASIISNNQNNIMKTLALVTIVMSIPTMVFSAYGMNFKDNEIPLNGEPNAFWLIVFIAFAMSVSLTLYLIHKKWF 2hn2.1    LDVYLSSVSNKTNEVMKVLTIIATIFMPLTFIAGIYGMNFEY--MPELRWKWGYPVVLAVMGVIAVIMVVYFKKKKWL ``` | | | | | | | | | | | | | | | | | | | | | | | | | | | | | | | | | | | | | | | | | | | | | | | | | |
|  | 2iub.1.A | DIVALENT CATION TRANSPORT-RELATED PROTEIN  *CRYSTAL STRUCTURE OF A DIVALENT METAL ION TRANSPORTER CORA AT 2.9 A RESOLUTION.* | 0.61 | 0.32 | 16.78 | 0.93 | 18-314 | X-ray | 2.90 | homo-pentamer | 10 x MG | HHblits | 0.30 |
| ``` target    MKQVFLSTTTEFKEIDTLEPGTWINLVNPTQ-NESLEIANTFDIDIADLRAPLDAEEMSRITIEDEYTLIIVDVPVTEER 2iub.1    -----------------SSTPTWINITGIHRTDVVQRVGEFFGIHPLVLEDILNVHQRPKVEFFENYVFIVLKMFTYDKN  target    NNRTYYVTIPLGIIITEETIITTCLEPLPVLDVFINRRLRNFY---TFMRSRFIFQILYRNAELYLTALRSIDRKSEQIE 2iub.1    L--HELESEQVSLILTKNCVLMFQEKIGDVFDPVRERIRYNRGIIRKKRADYLLYSLIDALVDDYFVLLEKIDDEIDVLE  target    SQLHQSTRNEELIELMELEKTIVYFKASLKTNERVIKKLTSSTSNIKKYLEDEDLLEDTLIETQQAIEMADIYGNVLHSM 2iub.1    EEVLERPEKETVQRTHQLKRNLVELRKTIWPLREVLSSLYRDVPPL-IEKETVPYFRDVYDHTIQIADTVETFRDIVSGL  target    TETFASIISNNQNNIMKTLALVTIVMSIPTMVFSAYGMNFKDNEIPLNGEPNAFWLIVFIAFAMSVSLTLYLIHKKWF 2iub.1    LDVYLSSVSNKTNEVMKVLTIIATIFMPLTFIAGIYGMNFEY--MPELRWKWGYPVVLAVMGVIAVIMVVYFKKKKWL ``` | | | | | | | | | | | | | | | | | | | | | | | | | | | | | | | | | | | | | | | | | | | | | | | | | |
|  | 2iub.1.C | DIVALENT CATION TRANSPORT-RELATED PROTEIN  *CRYSTAL STRUCTURE OF A DIVALENT METAL ION TRANSPORTER CORA AT 2.9 A RESOLUTION.* | 0.61 | 0.32 | 16.78 | 0.93 | 18-314 | X-ray | 2.90 | homo-pentamer | 10 x MG | HHblits | 0.30 |
| ``` target    MKQVFLSTTTEFKEIDTLEPGTWINLVNPTQ-NESLEIANTFDIDIADLRAPLDAEEMSRITIEDEYTLIIVDVPVTEER 2iub.1    -----------------SSTPTWINITGIHRTDVVQRVGEFFGIHPLVLEDILNVHQRPKVEFFENYVFIVLKMFTYDKN  target    NNRTYYVTIPLGIIITEETIITTCLEPLPVLDVFINRRLRNFY---TFMRSRFIFQILYRNAELYLTALRSIDRKSEQIE 2iub.1    L--HELESEQVSLILTKNCVLMFQEKIGDVFDPVRERIRYNRGIIRKKRADYLLYSLIDALVDDYFVLLEKIDDEIDVLE  target    SQLHQSTRNEELIELMELEKTIVYFKASLKTNERVIKKLTSSTSNIKKYLEDEDLLEDTLIETQQAIEMADIYGNVLHSM 2iub.1    EEVLERPEKETVQRTHQLKRNLVELRKTIWPLREVLSSLYRDVPPL-IEKETVPYFRDVYDHTIQIADTVETFRDIVSGL  target    TETFASIISNNQNNIMKTLALVTIVMSIPTMVFSAYGMNFKDNEIPLNGEPNAFWLIVFIAFAMSVSLTLYLIHKKWF 2iub.1    LDVYLSSVSNKTNEVMKVLTIIATIFMPLTFIAGIYGMNFEY--MPELRWKWGYPVVLAVMGVIAVIMVVYFKKKKWL ``` | | | | | | | | | | | | | | | | | | | | | | | | | | | | | | | | | | | | | | | | | | | | | | | | | |
|  | 3jcf.1.E | Magnesium transport protein CorA  *Cryo-EM structure of the magnesium channel CorA in the closed symmetric magnesium-bound state* | 0.63 | 0.50 | 16.78 | 0.93 | 18-314 | EM | 0.00 | homo-pentamer | 11 x MG | HHblits | 0.30 |
| ``` target    MKQVFLSTTTEFKEIDTLEPGTWINLVNPTQ-NESLEIANTFDIDIADLRAPLDAEEMSRITIEDEYTLIIVDVPVTEER 3jcf.1    -----------------SSTPTWINITGIHRTDVVQRVGEFFGIHPLVLEDILNVHQRPKVEFFENYVFIVLKMFTYDKN  target    NNRTYYVTIPLGIIITEETIITTCLEPLPVLDVFINRRLRNFY---TFMRSRFIFQILYRNAELYLTALRSIDRKSEQIE 3jcf.1    L--HELESEQVSLILTKNCVLMFQEKIGDVFDPVRERIRYNRGIIRKKRADYLLYSLIDALVDDYFVLLEKIDDEIDVLE  target    SQLHQSTRNEELIELMELEKTIVYFKASLKTNERVIKKLTSSTSNIKKYLEDEDLLEDTLIETQQAIEMADIYGNVLHSM 3jcf.1    EEVLERPEKETVQRTHQLKRNLVELRKTIWPLREVLSSLYRDVPPL-IEKETVPYFRDVYDHTIQIADTVETFRDIVSGL  target    TETFASIISNNQNNIMKTLALVTIVMSIPTMVFSAYGMNFKDNEIPLNGEPNAFWLIVFIAFAMSVSLTLYLIHKKWF 3jcf.1    LDVYLSSVSNKTNEVMKVLTIIATIFMPLTFIAGIYGMNFEY--MPELRWKWGYPVVLAVMGVIAVIMVVYFKKKKWL ``` | | | | | | | | | | | | | | | | | | | | | | | | | | | | | | | | | | | | | | | | | | | | | | | | | |
|  | 4i0u.1.D | Magnesium transport protein CorA  *Improved structure of Thermotoga maritima CorA at 2.7 A resolution* | 0.63 |  | 16.78 | 0.93 | 18-314 | X-ray | 2.70 | homo-pentamer | 11 x MG, 3 x LMT | HHblits | 0.30 |
| ``` target    MKQVFLSTTTEFKEIDTLEPGTWINLVNPTQ-NESLEIANTFDIDIADLRAPLDAEEMSRITIEDEYTLIIVDVPVTEER 4i0u.1    -----------------SSTPTWINITGIHRTDVVQRVGEFFGIHPLVLEDILNVHQRPKVEFFENYVFIVLKMFTYDKN  target    NNRTYYVTIPLGIIITEETIITTCLEPLPVLDVFINRRLRNFY---TFMRSRFIFQILYRNAELYLTALRSIDRKSEQIE 4i0u.1    L--HELESEQVSLILTKNCVLMFQEKIGDVFDPVRERIRYNRGIIRKKRADYLLYSLIDALVDDYFVLLEKIDDEIDVLE  target    SQLHQSTRNEELIELMELEKTIVYFKASLKTNERVIKKLTSSTSNIKKYLEDEDLLEDTLIETQQAIEMADIYGNVLHSM 4i0u.1    EEVLERPEKETVQRTHQLKRNLVELRKTIWPLREVLSSLYRDVPPL-IEKETVPYFRDVYDHTIQIADTVETFRDIVSGL  target    TETFASIISNNQNNIMKTLALVTIVMSIPTMVFSAYGMNFKDNEIPLNGEPNAFWLIVFIAFAMSVSLTLYLIHKKWF 4i0u.1    LDVYLSSVSNKTNEVMKVLTIIATIFMPLTFIAGIYGMNFEY--MPELRWKWGYPVVLAVMGVIAVIMVVYFKKKKWL ``` | | | | | | | | | | | | | | | | | | | | | | | | | | | | | | | | | | | | | | | | | | | | | | | | | |
|  | 4i0u.1.E | Magnesium transport protein CorA  *Improved structure of Thermotoga maritima CorA at 2.7 A resolution* | 0.63 |  | 16.78 | 0.93 | 18-314 | X-ray | 2.70 | homo-pentamer | 11 x MG, 3 x LMT | HHblits | 0.30 |
| ``` target    MKQVFLSTTTEFKEIDTLEPGTWINLVNPTQ-NESLEIANTFDIDIADLRAPLDAEEMSRITIEDEYTLIIVDVPVTEER 4i0u.1    -----------------SSTPTWINITGIHRTDVVQRVGEFFGIHPLVLEDILNVHQRPKVEFFENYVFIVLKMFTYDKN  target    NNRTYYVTIPLGIIITEETIITTCLEPLPVLDVFINRRLRNFY---TFMRSRFIFQILYRNAELYLTALRSIDRKSEQIE 4i0u.1    L--HELESEQVSLILTKNCVLMFQEKIGDVFDPVRERIRYNRGIIRKKRADYLLYSLIDALVDDYFVLLEKIDDEIDVLE  target    SQLHQSTRNEELIELMELEKTIVYFKASLKTNERVIKKLTSSTSNIKKYLEDEDLLEDTLIETQQAIEMADIYGNVLHSM 4i0u.1    EEVLERPEKETVQRTHQLKRNLVELRKTIWPLREVLSSLYRDVPPL-IEKETVPYFRDVYDHTIQIADTVETFRDIVSGL  target    TETFASIISNNQNNIMKTLALVTIVMSIPTMVFSAYGMNFKDNEIPLNGEPNAFWLIVFIAFAMSVSLTLYLIHKKWF 4i0u.1    LDVYLSSVSNKTNEVMKVLTIIATIFMPLTFIAGIYGMNFEY--MPELRWKWGYPVVLAVMGVIAVIMVVYFKKKKWL ``` | | | | | | | | | | | | | | | | | | | | | | | | | | | | | | | | | | | | | | | | | | | | | | | | | |
|  | 4i0u.2.A | Magnesium transport protein CorA  *Improved structure of Thermotoga maritima CorA at 2.7 A resolution* | 0.62 |  | 16.78 | 0.93 | 18-314 | X-ray | 2.70 | homo-pentamer | 13 x MG | HHblits | 0.30 |
| ``` target    MKQVFLSTTTEFKEIDTLEPGTWINLVNPTQ-NESLEIANTFDIDIADLRAPLDAEEMSRITIEDEYTLIIVDVPVTEER 4i0u.2    -----------------SSTPTWINITGIHRTDVVQRVGEFFGIHPLVLEDILNVHQRPKVEFFENYVFIVLKMFTYDKN  target    NNRTYYVTIPLGIIITEETIITTCLEPLPVLDVFINRRLRNFY---TFMRSRFIFQILYRNAELYLTALRSIDRKSEQIE 4i0u.2    L--HELESEQVSLILTKNCVLMFQEKIGDVFDPVRERIRYNRGIIRKKRADYLLYSLIDALVDDYFVLLEKIDDEIDVLE  target    SQLHQSTRNEELIELMELEKTIVYFKASLKTNERVIKKLTSSTSNIKKYLEDEDLLEDTLIETQQAIEMADIYGNVLHSM 4i0u.2    EEVLERPEKETVQRTHQLKRNLVELRKTIWPLREVLSSLYRDVPPL-IEKETVPYFRDVYDHTIQIADTVETFRDIVSGL  target    TETFASIISNNQNNIMKTLALVTIVMSIPTMVFSAYGMNFKDNEIPLNGEPNAFWLIVFIAFAMSVSLTLYLIHKKWF 4i0u.2    LDVYLSSVSNKTNEVMKVLTIIATIFMPLTFIAGIYGMNFEY--MPELRWKWGYPVVLAVMGVIAVIMVVYFKKKKWL ``` | | | | | | | | | | | | | | | | | | | | | | | | | | | | | | | | | | | | | | | | | | | | | | | | | |
|  | 4i0u.2.B | Magnesium transport protein CorA  *Improved structure of Thermotoga maritima CorA at 2.7 A resolution* | 0.63 |  | 16.78 | 0.93 | 18-314 | X-ray | 2.70 | homo-pentamer | 13 x MG | HHblits | 0.30 |
| ``` target    MKQVFLSTTTEFKEIDTLEPGTWINLVNPTQ-NESLEIANTFDIDIADLRAPLDAEEMSRITIEDEYTLIIVDVPVTEER 4i0u.2    -----------------SSTPTWINITGIHRTDVVQRVGEFFGIHPLVLEDILNVHQRPKVEFFENYVFIVLKMFTYDKN  target    NNRTYYVTIPLGIIITEETIITTCLEPLPVLDVFINRRLRNFY---TFMRSRFIFQILYRNAELYLTALRSIDRKSEQIE 4i0u.2    L--HELESEQVSLILTKNCVLMFQEKIGDVFDPVRERIRYNRGIIRKKRADYLLYSLIDALVDDYFVLLEKIDDEIDVLE  target    SQLHQSTRNEELIELMELEKTIVYFKASLKTNERVIKKLTSSTSNIKKYLEDEDLLEDTLIETQQAIEMADIYGNVLHSM 4i0u.2    EEVLERPEKETVQRTHQLKRNLVELRKTIWPLREVLSSLYRDVPPL-IEKETVPYFRDVYDHTIQIADTVETFRDIVSGL  target    TETFASIISNNQNNIMKTLALVTIVMSIPTMVFSAYGMNFKDNEIPLNGEPNAFWLIVFIAFAMSVSLTLYLIHKKWF 4i0u.2    LDVYLSSVSNKTNEVMKVLTIIATIFMPLTFIAGIYGMNFEY--MPELRWKWGYPVVLAVMGVIAVIMVVYFKKKKWL ``` | | | | | | | | | | | | | | | | | | | | | | | | | | | | | | | | | | | | | | | | | | | | | | | | | |
|  | 4i0u.2.C | Magnesium transport protein CorA  *Improved structure of Thermotoga maritima CorA at 2.7 A resolution* | 0.62 |  | 16.78 | 0.93 | 18-314 | X-ray | 2.70 | homo-pentamer | 13 x MG | HHblits | 0.30 |
| ``` target    MKQVFLSTTTEFKEIDTLEPGTWINLVNPTQ-NESLEIANTFDIDIADLRAPLDAEEMSRITIEDEYTLIIVDVPVTEER 4i0u.2    -----------------SSTPTWINITGIHRTDVVQRVGEFFGIHPLVLEDILNVHQRPKVEFFENYVFIVLKMFTYDKN  target    NNRTYYVTIPLGIIITEETIITTCLEPLPVLDVFINRRLRNFY---TFMRSRFIFQILYRNAELYLTALRSIDRKSEQIE 4i0u.2    L--HELESEQVSLILTKNCVLMFQEKIGDVFDPVRERIRYNRGIIRKKRADYLLYSLIDALVDDYFVLLEKIDDEIDVLE  target    SQLHQSTRNEELIELMELEKTIVYFKASLKTNERVIKKLTSSTSNIKKYLEDEDLLEDTLIETQQAIEMADIYGNVLHSM 4i0u.2    EEVLERPEKETVQRTHQLKRNLVELRKTIWPLREVLSSLYRDVPPL-IEKETVPYFRDVYDHTIQIADTVETFRDIVSGL  target    TETFASIISNNQNNIMKTLALVTIVMSIPTMVFSAYGMNFKDNEIPLNGEPNAFWLIVFIAFAMSVSLTLYLIHKKWF 4i0u.2    LDVYLSSVSNKTNEVMKVLTIIATIFMPLTFIAGIYGMNFEY--MPELRWKWGYPVVLAVMGVIAVIMVVYFKKKKWL ``` | | | | | | | | | | | | | | | | | | | | | | | | | | | | | | | | | | | | | | | | | | | | | | | | | |
|  | 4i0u.2.D | Magnesium transport protein CorA  *Improved structure of Thermotoga maritima CorA at 2.7 A resolution* | 0.63 |  | 16.78 | 0.93 | 18-314 | X-ray | 2.70 | homo-pentamer | 13 x MG | HHblits | 0.30 |
| ``` target    MKQVFLSTTTEFKEIDTLEPGTWINLVNPTQ-NESLEIANTFDIDIADLRAPLDAEEMSRITIEDEYTLIIVDVPVTEER 4i0u.2    -----------------SSTPTWINITGIHRTDVVQRVGEFFGIHPLVLEDILNVHQRPKVEFFENYVFIVLKMFTYDKN  target    NNRTYYVTIPLGIIITEETIITTCLEPLPVLDVFINRRLRNFY---TFMRSRFIFQILYRNAELYLTALRSIDRKSEQIE 4i0u.2    L--HELESEQVSLILTKNCVLMFQEKIGDVFDPVRERIRYNRGIIRKKRADYLLYSLIDALVDDYFVLLEKIDDEIDVLE  target    SQLHQSTRNEELIELMELEKTIVYFKASLKTNERVIKKLTSSTSNIKKYLEDEDLLEDTLIETQQAIEMADIYGNVLHSM 4i0u.2    EEVLERPEKETVQRTHQLKRNLVELRKTIWPLREVLSSLYRDVPPL-IEKETVPYFRDVYDHTIQIADTVETFRDIVSGL  target    TETFASIISNNQNNIMKTLALVTIVMSIPTMVFSAYGMNFKDNEIPLNGEPNAFWLIVFIAFAMSVSLTLYLIHKKWF 4i0u.2    LDVYLSSVSNKTNEVMKVLTIIATIFMPLTFIAGIYGMNFEY--MPELRWKWGYPVVLAVMGVIAVIMVVYFKKKKWL ``` | | | | | | | | | | | | | | | | | | | | | | | | | | | | | | | | | | | | | | | | | | | | | | | | | |
|  | 4i0u.2.E | Magnesium transport protein CorA  *Improved structure of Thermotoga maritima CorA at 2.7 A resolution* | 0.63 |  | 16.78 | 0.93 | 18-314 | X-ray | 2.70 | homo-pentamer | 13 x MG | HHblits | 0.30 |
| ``` target    MKQVFLSTTTEFKEIDTLEPGTWINLVNPTQ-NESLEIANTFDIDIADLRAPLDAEEMSRITIEDEYTLIIVDVPVTEER 4i0u.2    -----------------SSTPTWINITGIHRTDVVQRVGEFFGIHPLVLEDILNVHQRPKVEFFENYVFIVLKMFTYDKN  target    NNRTYYVTIPLGIIITEETIITTCLEPLPVLDVFINRRLRNFY---TFMRSRFIFQILYRNAELYLTALRSIDRKSEQIE 4i0u.2    L--HELESEQVSLILTKNCVLMFQEKIGDVFDPVRERIRYNRGIIRKKRADYLLYSLIDALVDDYFVLLEKIDDEIDVLE  target    SQLHQSTRNEELIELMELEKTIVYFKASLKTNERVIKKLTSSTSNIKKYLEDEDLLEDTLIETQQAIEMADIYGNVLHSM 4i0u.2    EEVLERPEKETVQRTHQLKRNLVELRKTIWPLREVLSSLYRDVPPL-IEKETVPYFRDVYDHTIQIADTVETFRDIVSGL  target    TETFASIISNNQNNIMKTLALVTIVMSIPTMVFSAYGMNFKDNEIPLNGEPNAFWLIVFIAFAMSVSLTLYLIHKKWF 4i0u.2    LDVYLSSVSNKTNEVMKVLTIIATIFMPLTFIAGIYGMNFEY--MPELRWKWGYPVVLAVMGVIAVIMVVYFKKKKWL ``` | | | | | | | | | | | | | | | | | | | | | | | | | | | | | | | | | | | | | | | | | | | | | | | | | |
|  | 4i0u.1.A | Magnesium transport protein CorA  *Improved structure of Thermotoga maritima CorA at 2.7 A resolution* | 0.63 |  | 16.78 | 0.93 | 18-314 | X-ray | 2.70 | homo-pentamer | 11 x MG, 3 x LMT | HHblits | 0.30 |
| ``` target    MKQVFLSTTTEFKEIDTLEPGTWINLVNPTQ-NESLEIANTFDIDIADLRAPLDAEEMSRITIEDEYTLIIVDVPVTEER 4i0u.1    -----------------SSTPTWINITGIHRTDVVQRVGEFFGIHPLVLEDILNVHQRPKVEFFENYVFIVLKMFTYDKN  target    NNRTYYVTIPLGIIITEETIITTCLEPLPVLDVFINRRLRNFY---TFMRSRFIFQILYRNAELYLTALRSIDRKSEQIE 4i0u.1    L--HELESEQVSLILTKNCVLMFQEKIGDVFDPVRERIRYNRGIIRKKRADYLLYSLIDALVDDYFVLLEKIDDEIDVLE  target    SQLHQSTRNEELIELMELEKTIVYFKASLKTNERVIKKLTSSTSNIKKYLEDEDLLEDTLIETQQAIEMADIYGNVLHSM 4i0u.1    EEVLERPEKETVQRTHQLKRNLVELRKTIWPLREVLSSLYRDVPPL-IEKETVPYFRDVYDHTIQIADTVETFRDIVSGL  target    TETFASIISNNQNNIMKTLALVTIVMSIPTMVFSAYGMNFKDNEIPLNGEPNAFWLIVFIAFAMSVSLTLYLIHKKWF 4i0u.1    LDVYLSSVSNKTNEVMKVLTIIATIFMPLTFIAGIYGMNFEY--MPELRWKWGYPVVLAVMGVIAVIMVVYFKKKKWL ``` | | | | | | | | | | | | | | | | | | | | | | | | | | | | | | | | | | | | | | | | | | | | | | | | | |
|  | 4i0u.1.B | Magnesium transport protein CorA  *Improved structure of Thermotoga maritima CorA at 2.7 A resolution* | 0.63 |  | 16.78 | 0.93 | 18-314 | X-ray | 2.70 | homo-pentamer | 11 x MG, 3 x LMT | HHblits | 0.30 |
| ``` target    MKQVFLSTTTEFKEIDTLEPGTWINLVNPTQ-NESLEIANTFDIDIADLRAPLDAEEMSRITIEDEYTLIIVDVPVTEER 4i0u.1    -----------------SSTPTWINITGIHRTDVVQRVGEFFGIHPLVLEDILNVHQRPKVEFFENYVFIVLKMFTYDKN  target    NNRTYYVTIPLGIIITEETIITTCLEPLPVLDVFINRRLRNFY---TFMRSRFIFQILYRNAELYLTALRSIDRKSEQIE 4i0u.1    L--HELESEQVSLILTKNCVLMFQEKIGDVFDPVRERIRYNRGIIRKKRADYLLYSLIDALVDDYFVLLEKIDDEIDVLE  target    SQLHQSTRNEELIELMELEKTIVYFKASLKTNERVIKKLTSSTSNIKKYLEDEDLLEDTLIETQQAIEMADIYGNVLHSM 4i0u.1    EEVLERPEKETVQRTHQLKRNLVELRKTIWPLREVLSSLYRDVPPL-IEKETVPYFRDVYDHTIQIADTVETFRDIVSGL  target    TETFASIISNNQNNIMKTLALVTIVMSIPTMVFSAYGMNFKDNEIPLNGEPNAFWLIVFIAFAMSVSLTLYLIHKKWF 4i0u.1    LDVYLSSVSNKTNEVMKVLTIIATIFMPLTFIAGIYGMNFEY--MPELRWKWGYPVVLAVMGVIAVIMVVYFKKKKWL ``` | | | | | | | | | | | | | | | | | | | | | | | | | | | | | | | | | | | | | | | | | | | | | | | | | |
|  | 3jcg.1.D | Magnesium transport protein CorA  *Cryo-EM structure of the magnesium channel CorA in the magnesium-free, asymmetric open state I* | 0.59 |  | 16.78 | 0.93 | 18-314 | EM | 0.00 | homo-pentamer |  | HHblits | 0.30 |
| ``` target    MKQVFLSTTTEFKEIDTLEPGTWINLVNPTQ-NESLEIANTFDIDIADLRAPLDAEEMSRITIEDEYTLIIVDVPVTEER 3jcg.1    -----------------SSTPTWINITGIHRTDVVQRVGEFFGIHPLVLEDILNVHQRPKVEFFENYVFIVLKMFTYDKN  target    NNRTYYVTIPLGIIITEETIITTCLEPLPVLDVFINRRLRNFY---TFMRSRFIFQILYRNAELYLTALRSIDRKSEQIE 3jcg.1    L--HELESEQVSLILTKNCVLMFQEKIGDVFDPVRERIRYNRGIIRKKRADYLLYSLIDALVDDYFVLLEKIDDEIDVLE  target    SQLHQSTRNEELIELMELEKTIVYFKASLKTNERVIKKLTSSTSNIKKYLEDEDLLEDTLIETQQAIEMADIYGNVLHSM 3jcg.1    EEVLERPEKETVQRTHQLKRNLVELRKTIWPLREVLSSLYRDVPPL-IEKETVPYFRDVYDHTIQIADTVETFRDIVSGL  target    TETFASIISNNQNNIMKTLALVTIVMSIPTMVFSAYGMNFKDNEIPLNGEPNAFWLIVFIAFAMSVSLTLYLIHKKWF 3jcg.1    LDVYLSSVSNKTNEVMKVLTIIATIFMPLTFIAGIYGMNFEY--MPELRWKWGYPVVLAVMGVIAVIMVVYFKKKKWL ``` | | | | | | | | | | | | | | | | | | | | | | | | | | | | | | | | | | | | | | | | | | | | | | | | | |
|  | 3jcg.1.E | Magnesium transport protein CorA  *Cryo-EM structure of the magnesium channel CorA in the magnesium-free, asymmetric open state I* | 0.59 |  | 16.78 | 0.93 | 18-314 | EM | 0.00 | homo-pentamer |  | HHblits | 0.30 |
| ``` target    MKQVFLSTTTEFKEIDTLEPGTWINLVNPTQ-NESLEIANTFDIDIADLRAPLDAEEMSRITIEDEYTLIIVDVPVTEER 3jcg.1    -----------------SSTPTWINITGIHRTDVVQRVGEFFGIHPLVLEDILNVHQRPKVEFFENYVFIVLKMFTYDKN  target    NNRTYYVTIPLGIIITEETIITTCLEPLPVLDVFINRRLRNFY---TFMRSRFIFQILYRNAELYLTALRSIDRKSEQIE 3jcg.1    L--HELESEQVSLILTKNCVLMFQEKIGDVFDPVRERIRYNRGIIRKKRADYLLYSLIDALVDDYFVLLEKIDDEIDVLE  target    SQLHQSTRNEELIELMELEKTIVYFKASLKTNERVIKKLTSSTSNIKKYLEDEDLLEDTLIETQQAIEMADIYGNVLHSM 3jcg.1    EEVLERPEKETVQRTHQLKRNLVELRKTIWPLREVLSSLYRDVPPL-IEKETVPYFRDVYDHTIQIADTVETFRDIVSGL  target    TETFASIISNNQNNIMKTLALVTIVMSIPTMVFSAYGMNFKDNEIPLNGEPNAFWLIVFIAFAMSVSLTLYLIHKKWF 3jcg.1    LDVYLSSVSNKTNEVMKVLTIIATIFMPLTFIAGIYGMNFEY--MPELRWKWGYPVVLAVMGVIAVIMVVYFKKKKWL ``` | | | | | | | | | | | | | | | | | | | | | | | | | | | | | | | | | | | | | | | | | | | | | | | | | |
|  | 3jcg.1.A | Magnesium transport protein CorA  *Cryo-EM structure of the magnesium channel CorA in the magnesium-free, asymmetric open state I* | 0.58 |  | 16.78 | 0.93 | 18-314 | EM | 0.00 | homo-pentamer |  | HHblits | 0.30 |
| ``` target    MKQVFLSTTTEFKEIDTLEPGTWINLVNPTQ-NESLEIANTFDIDIADLRAPLDAEEMSRITIEDEYTLIIVDVPVTEER 3jcg.1    -----------------SSTPTWINITGIHRTDVVQRVGEFFGIHPLVLEDILNVHQRPKVEFFENYVFIVLKMFTYDKN  target    NNRTYYVTIPLGIIITEETIITTCLEPLPVLDVFINRRLRNFY---TFMRSRFIFQILYRNAELYLTALRSIDRKSEQIE 3jcg.1    L--HELESEQVSLILTKNCVLMFQEKIGDVFDPVRERIRYNRGIIRKKRADYLLYSLIDALVDDYFVLLEKIDDEIDVLE  target    SQLHQSTRNEELIELMELEKTIVYFKASLKTNERVIKKLTSSTSNIKKYLEDEDLLEDTLIETQQAIEMADIYGNVLHSM 3jcg.1    EEVLERPEKETVQRTHQLKRNLVELRKTIWPLREVLSSLYRDVPPL-IEKETVPYFRDVYDHTIQIADTVETFRDIVSGL  target    TETFASIISNNQNNIMKTLALVTIVMSIPTMVFSAYGMNFKDNEIPLNGEPNAFWLIVFIAFAMSVSLTLYLIHKKWF 3jcg.1    LDVYLSSVSNKTNEVMKVLTIIATIFMPLTFIAGIYGMNFEY--MPELRWKWGYPVVLAVMGVIAVIMVVYFKKKKWL ``` | | | | | | | | | | | | | | | | | | | | | | | | | | | | | | | | | | | | | | | | | | | | | | | | | |
|  | 3jcg.1.B | Magnesium transport protein CorA  *Cryo-EM structure of the magnesium channel CorA in the magnesium-free, asymmetric open state I* | 0.59 |  | 16.78 | 0.93 | 18-314 | EM | 0.00 | homo-pentamer |  | HHblits | 0.30 |
| ``` target    MKQVFLSTTTEFKEIDTLEPGTWINLVNPTQ-NESLEIANTFDIDIADLRAPLDAEEMSRITIEDEYTLIIVDVPVTEER 3jcg.1    -----------------SSTPTWINITGIHRTDVVQRVGEFFGIHPLVLEDILNVHQRPKVEFFENYVFIVLKMFTYDKN  target    NNRTYYVTIPLGIIITEETIITTCLEPLPVLDVFINRRLRNFY---TFMRSRFIFQILYRNAELYLTALRSIDRKSEQIE 3jcg.1    L--HELESEQVSLILTKNCVLMFQEKIGDVFDPVRERIRYNRGIIRKKRADYLLYSLIDALVDDYFVLLEKIDDEIDVLE  target    SQLHQSTRNEELIELMELEKTIVYFKASLKTNERVIKKLTSSTSNIKKYLEDEDLLEDTLIETQQAIEMADIYGNVLHSM 3jcg.1    EEVLERPEKETVQRTHQLKRNLVELRKTIWPLREVLSSLYRDVPPL-IEKETVPYFRDVYDHTIQIADTVETFRDIVSGL  target    TETFASIISNNQNNIMKTLALVTIVMSIPTMVFSAYGMNFKDNEIPLNGEPNAFWLIVFIAFAMSVSLTLYLIHKKWF 3jcg.1    LDVYLSSVSNKTNEVMKVLTIIATIFMPLTFIAGIYGMNFEY--MPELRWKWGYPVVLAVMGVIAVIMVVYFKKKKWL ``` | | | | | | | | | | | | | | | | | | | | | | | | | | | | | | | | | | | | | | | | | | | | | | | | | |
|  | 3jcg.1.C | Magnesium transport protein CorA  *Cryo-EM structure of the magnesium channel CorA in the magnesium-free, asymmetric open state I* | 0.59 |  | 16.78 | 0.93 | 18-314 | EM | 0.00 | homo-pentamer |  | HHblits | 0.30 |
| ``` target    MKQVFLSTTTEFKEIDTLEPGTWINLVNPTQ-NESLEIANTFDIDIADLRAPLDAEEMSRITIEDEYTLIIVDVPVTEER 3jcg.1    -----------------SSTPTWINITGIHRTDVVQRVGEFFGIHPLVLEDILNVHQRPKVEFFENYVFIVLKMFTYDKN  target    NNRTYYVTIPLGIIITEETIITTCLEPLPVLDVFINRRLRNFY---TFMRSRFIFQILYRNAELYLTALRSIDRKSEQIE 3jcg.1    L--HELESEQVSLILTKNCVLMFQEKIGDVFDPVRERIRYNRGIIRKKRADYLLYSLIDALVDDYFVLLEKIDDEIDVLE  target    SQLHQSTRNEELIELMELEKTIVYFKASLKTNERVIKKLTSSTSNIKKYLEDEDLLEDTLIETQQAIEMADIYGNVLHSM 3jcg.1    EEVLERPEKETVQRTHQLKRNLVELRKTIWPLREVLSSLYRDVPPL-IEKETVPYFRDVYDHTIQIADTVETFRDIVSGL  target    TETFASIISNNQNNIMKTLALVTIVMSIPTMVFSAYGMNFKDNEIPLNGEPNAFWLIVFIAFAMSVSLTLYLIHKKWF 3jcg.1    LDVYLSSVSNKTNEVMKVLTIIATIFMPLTFIAGIYGMNFEY--MPELRWKWGYPVVLAVMGVIAVIMVVYFKKKKWL ``` | | | | | | | | | | | | | | | | | | | | | | | | | | | | | | | | | | | | | | | | | | | | | | | | | |
|  | 4i0u.1.C | Magnesium transport protein CorA  *Improved structure of Thermotoga maritima CorA at 2.7 A resolution* | 0.63 |  | 16.78 | 0.93 | 18-314 | X-ray | 2.70 | homo-pentamer | 11 x MG, 3 x LMT | HHblits | 0.30 |
| ``` target    MKQVFLSTTTEFKEIDTLEPGTWINLVNPTQ-NESLEIANTFDIDIADLRAPLDAEEMSRITIEDEYTLIIVDVPVTEER 4i0u.1    -----------------SSTPTWINITGIHRTDVVQRVGEFFGIHPLVLEDILNVHQRPKVEFFENYVFIVLKMFTYDKN  target    NNRTYYVTIPLGIIITEETIITTCLEPLPVLDVFINRRLRNFY---TFMRSRFIFQILYRNAELYLTALRSIDRKSEQIE 4i0u.1    L--HELESEQVSLILTKNCVLMFQEKIGDVFDPVRERIRYNRGIIRKKRADYLLYSLIDALVDDYFVLLEKIDDEIDVLE  target    SQLHQSTRNEELIELMELEKTIVYFKASLKTNERVIKKLTSSTSNIKKYLEDEDLLEDTLIETQQAIEMADIYGNVLHSM 4i0u.1    EEVLERPEKETVQRTHQLKRNLVELRKTIWPLREVLSSLYRDVPPL-IEKETVPYFRDVYDHTIQIADTVETFRDIVSGL  target    TETFASIISNNQNNIMKTLALVTIVMSIPTMVFSAYGMNFKDNEIPLNGEPNAFWLIVFIAFAMSVSLTLYLIHKKWF 4i0u.1    LDVYLSSVSNKTNEVMKVLTIIATIFMPLTFIAGIYGMNFEY--MPELRWKWGYPVVLAVMGVIAVIMVVYFKKKKWL ``` | | | | | | | | | | | | | | | | | | | | | | | | | | | | | | | | | | | | | | | | | | | | | | | | | |
|  | 3jch.1.C | Magnesium transport protein CorA  *Cryo-EM structure of the magnesium channel CorA in the magnesium-free, asymmetric open state II* | 0.60 |  | 16.78 | 0.93 | 18-314 | EM | 0.00 | homo-pentamer |  | HHblits | 0.30 |
| ``` target    MKQVFLSTTTEFKEIDTLEPGTWINLVNPTQ-NESLEIANTFDIDIADLRAPLDAEEMSRITIEDEYTLIIVDVPVTEER 3jch.1    -----------------SSTPTWINITGIHRTDVVQRVGEFFGIHPLVLEDILNVHQRPKVEFFENYVFIVLKMFTYDKN  target    NNRTYYVTIPLGIIITEETIITTCLEPLPVLDVFINRRLRNFY---TFMRSRFIFQILYRNAELYLTALRSIDRKSEQIE 3jch.1    L--HELESEQVSLILTKNCVLMFQEKIGDVFDPVRERIRYNRGIIRKKRADYLLYSLIDALVDDYFVLLEKIDDEIDVLE  target    SQLHQSTRNEELIELMELEKTIVYFKASLKTNERVIKKLTSSTSNIKKYLEDEDLLEDTLIETQQAIEMADIYGNVLHSM 3jch.1    EEVLERPEKETVQRTHQLKRNLVELRKTIWPLREVLSSLYRDVPPL-IEKETVPYFRDVYDHTIQIADTVETFRDIVSGL  target    TETFASIISNNQNNIMKTLALVTIVMSIPTMVFSAYGMNFKDNEIPLNGEPNAFWLIVFIAFAMSVSLTLYLIHKKWF 3jch.1    LDVYLSSVSNKTNEVMKVLTIIATIFMPLTFIAGIYGMNFEY--MPELRWKWGYPVVLAVMGVIAVIMVVYFKKKKWL ``` | | | | | | | | | | | | | | | | | | | | | | | | | | | | | | | | | | | | | | | | | | | | | | | | | |
|  | 3jch.1.B | Magnesium transport protein CorA  *Cryo-EM structure of the magnesium channel CorA in the magnesium-free, asymmetric open state II* | 0.60 |  | 16.78 | 0.93 | 18-314 | EM | 0.00 | homo-pentamer |  | HHblits | 0.30 |
| ``` target    MKQVFLSTTTEFKEIDTLEPGTWINLVNPTQ-NESLEIANTFDIDIADLRAPLDAEEMSRITIEDEYTLIIVDVPVTEER 3jch.1    -----------------SSTPTWINITGIHRTDVVQRVGEFFGIHPLVLEDILNVHQRPKVEFFENYVFIVLKMFTYDKN  target    NNRTYYVTIPLGIIITEETIITTCLEPLPVLDVFINRRLRNFY---TFMRSRFIFQILYRNAELYLTALRSIDRKSEQIE 3jch.1    L--HELESEQVSLILTKNCVLMFQEKIGDVFDPVRERIRYNRGIIRKKRADYLLYSLIDALVDDYFVLLEKIDDEIDVLE  target    SQLHQSTRNEELIELMELEKTIVYFKASLKTNERVIKKLTSSTSNIKKYLEDEDLLEDTLIETQQAIEMADIYGNVLHSM 3jch.1    EEVLERPEKETVQRTHQLKRNLVELRKTIWPLREVLSSLYRDVPPL-IEKETVPYFRDVYDHTIQIADTVETFRDIVSGL  target    TETFASIISNNQNNIMKTLALVTIVMSIPTMVFSAYGMNFKDNEIPLNGEPNAFWLIVFIAFAMSVSLTLYLIHKKWF 3jch.1    LDVYLSSVSNKTNEVMKVLTIIATIFMPLTFIAGIYGMNFEY--MPELRWKWGYPVVLAVMGVIAVIMVVYFKKKKWL ``` | | | | | | | | | | | | | | | | | | | | | | | | | | | | | | | | | | | | | | | | | | | | | | | | | |
|  | 3jch.1.A | Magnesium transport protein CorA  *Cryo-EM structure of the magnesium channel CorA in the magnesium-free, asymmetric open state II* | 0.58 |  | 16.78 | 0.93 | 18-314 | EM | 0.00 | homo-pentamer |  | HHblits | 0.30 |
| ``` target    MKQVFLSTTTEFKEIDTLEPGTWINLVNPTQ-NESLEIANTFDIDIADLRAPLDAEEMSRITIEDEYTLIIVDVPVTEER 3jch.1    -----------------SSTPTWINITGIHRTDVVQRVGEFFGIHPLVLEDILNVHQRPKVEFFENYVFIVLKMFTYDKN  target    NNRTYYVTIPLGIIITEETIITTCLEPLPVLDVFINRRLRNFY---TFMRSRFIFQILYRNAELYLTALRSIDRKSEQIE 3jch.1    L--HELESEQVSLILTKNCVLMFQEKIGDVFDPVRERIRYNRGIIRKKRADYLLYSLIDALVDDYFVLLEKIDDEIDVLE  target    SQLHQSTRNEELIELMELEKTIVYFKASLKTNERVIKKLTSSTSNIKKYLEDEDLLEDTLIETQQAIEMADIYGNVLHSM 3jch.1    EEVLERPEKETVQRTHQLKRNLVELRKTIWPLREVLSSLYRDVPPL-IEKETVPYFRDVYDHTIQIADTVETFRDIVSGL  target    TETFASIISNNQNNIMKTLALVTIVMSIPTMVFSAYGMNFKDNEIPLNGEPNAFWLIVFIAFAMSVSLTLYLIHKKWF 3jch.1    LDVYLSSVSNKTNEVMKVLTIIATIFMPLTFIAGIYGMNFEY--MPELRWKWGYPVVLAVMGVIAVIMVVYFKKKKWL ``` | | | | | | | | | | | | | | | | | | | | | | | | | | | | | | | | | | | | | | | | | | | | | | | | | |
|  | 3jch.1.D | Magnesium transport protein CorA  *Cryo-EM structure of the magnesium channel CorA in the magnesium-free, asymmetric open state II* | 0.59 |  | 16.78 | 0.93 | 18-314 | EM | 0.00 | homo-pentamer |  | HHblits | 0.30 |
| ``` target    MKQVFLSTTTEFKEIDTLEPGTWINLVNPTQ-NESLEIANTFDIDIADLRAPLDAEEMSRITIEDEYTLIIVDVPVTEER 3jch.1    -----------------SSTPTWINITGIHRTDVVQRVGEFFGIHPLVLEDILNVHQRPKVEFFENYVFIVLKMFTYDKN  target    NNRTYYVTIPLGIIITEETIITTCLEPLPVLDVFINRRLRNFY---TFMRSRFIFQILYRNAELYLTALRSIDRKSEQIE 3jch.1    L--HELESEQVSLILTKNCVLMFQEKIGDVFDPVRERIRYNRGIIRKKRADYLLYSLIDALVDDYFVLLEKIDDEIDVLE  target    SQLHQSTRNEELIELMELEKTIVYFKASLKTNERVIKKLTSSTSNIKKYLEDEDLLEDTLIETQQAIEMADIYGNVLHSM 3jch.1    EEVLERPEKETVQRTHQLKRNLVELRKTIWPLREVLSSLYRDVPPL-IEKETVPYFRDVYDHTIQIADTVETFRDIVSGL  target    TETFASIISNNQNNIMKTLALVTIVMSIPTMVFSAYGMNFKDNEIPLNGEPNAFWLIVFIAFAMSVSLTLYLIHKKWF 3jch.1    LDVYLSSVSNKTNEVMKVLTIIATIFMPLTFIAGIYGMNFEY--MPELRWKWGYPVVLAVMGVIAVIMVVYFKKKKWL ``` | | | | | | | | | | | | | | | | | | | | | | | | | | | | | | | | | | | | | | | | | | | | | | | | | |
|  | 5jtg.1.B | Cobalt/magnesium transport protein CorA  *Crystal structure of Thermotoga maritima mutant D89K/D253K* | 0.61 |  | 16.44 | 0.93 | 18-314 | X-ray | 3.05 | homo-pentamer | 3 x MG | HHblits | 0.30 |
| ``` target    MKQVFLSTTTEFKEIDTLEPGTWINLVNPTQ-NESLEIANTFDIDIADLRAPLDAEEMSRITIEDEYTLIIVDVPVTEER 5jtg.1    -----------------SSTPTWINITGIHRTDVVQRVGEFFGIHPLVLEKILNVHQRPKVEFFENYVFIVLKMFTYDKN  target    NNRTYYVTIPLGIIITEETIITTCLEPLPVLDVFINRRLRNFY---TFMRSRFIFQILYRNAELYLTALRSIDRKSEQIE 5jtg.1    L--HELESEQVSLILTKNCVLMFQEKIGDVFDPVRERIRYNRGIIRKKRADYLLYSLIDALVDDYFVLLEKIDDEIDVLE  target    SQLHQSTRNEELIELMELEKTIVYFKASLKTNERVIKKLTSSTSNIKKYLEDEDLLEDTLIETQQAIEMADIYGNVLHSM 5jtg.1    EEVLERPEKETVQRTHQLKRNLVELRKTIWPLREVLSSLYRDVPPL-IEKETVPYFRKVYDHTIQIADTVETFRDIVSGL  target    TETFASIISNNQNNIMKTLALVTIVMSIPTMVFSAYGMNFKDNEIPLNGEPNAFWLIVFIAFAMSVSLTLYLIHKKWF 5jtg.1    LDVYLSSVSNKTNEVMKVLTIIATIFMPLTFIAGIYGMNFEY--MPELRWKWGYPVVLAVMGVIAVIMVVYFKKKKWL ``` | | | | | | | | | | | | | | | | | | | | | | | | | | | | | | | | | | | | | | | | | | | | | | | | | |
|  | 5jtg.1.A | Cobalt/magnesium transport protein CorA  *Crystal structure of Thermotoga maritima mutant D89K/D253K* | 0.61 |  | 16.44 | 0.93 | 18-314 | X-ray | 3.05 | homo-pentamer | 3 x MG | HHblits | 0.30 |
| ``` target    MKQVFLSTTTEFKEIDTLEPGTWINLVNPTQ-NESLEIANTFDIDIADLRAPLDAEEMSRITIEDEYTLIIVDVPVTEER 5jtg.1    -----------------SSTPTWINITGIHRTDVVQRVGEFFGIHPLVLEKILNVHQRPKVEFFENYVFIVLKMFTYDKN  target    NNRTYYVTIPLGIIITEETIITTCLEPLPVLDVFINRRLRNFY---TFMRSRFIFQILYRNAELYLTALRSIDRKSEQIE 5jtg.1    L--HELESEQVSLILTKNCVLMFQEKIGDVFDPVRERIRYNRGIIRKKRADYLLYSLIDALVDDYFVLLEKIDDEIDVLE  target    SQLHQSTRNEELIELMELEKTIVYFKASLKTNERVIKKLTSSTSNIKKYLEDEDLLEDTLIETQQAIEMADIYGNVLHSM 5jtg.1    EEVLERPEKETVQRTHQLKRNLVELRKTIWPLREVLSSLYRDVPPL-IEKETVPYFRKVYDHTIQIADTVETFRDIVSGL  target    TETFASIISNNQNNIMKTLALVTIVMSIPTMVFSAYGMNFKDNEIPLNGEPNAFWLIVFIAFAMSVSLTLYLIHKKWF 5jtg.1    LDVYLSSVSNKTNEVMKVLTIIATIFMPLTFIAGIYGMNFEY--MPELRWKWGYPVVLAVMGVIAVIMVVYFKKKKWL ``` | | | | | | | | | | | | | | | | | | | | | | | | | | | | | | | | | | | | | | | | | | | | | | | | | |
|  | 5jtg.1.C | Cobalt/magnesium transport protein CorA  *Crystal structure of Thermotoga maritima mutant D89K/D253K* | 0.61 |  | 16.44 | 0.93 | 18-314 | X-ray | 3.05 | homo-pentamer | 3 x MG | HHblits | 0.30 |
| ``` target    MKQVFLSTTTEFKEIDTLEPGTWINLVNPTQ-NESLEIANTFDIDIADLRAPLDAEEMSRITIEDEYTLIIVDVPVTEER 5jtg.1    -----------------SSTPTWINITGIHRTDVVQRVGEFFGIHPLVLEKILNVHQRPKVEFFENYVFIVLKMFTYDKN  target    NNRTYYVTIPLGIIITEETIITTCLEPLPVLDVFINRRLRNFY---TFMRSRFIFQILYRNAELYLTALRSIDRKSEQIE 5jtg.1    L--HELESEQVSLILTKNCVLMFQEKIGDVFDPVRERIRYNRGIIRKKRADYLLYSLIDALVDDYFVLLEKIDDEIDVLE  target    SQLHQSTRNEELIELMELEKTIVYFKASLKTNERVIKKLTSSTSNIKKYLEDEDLLEDTLIETQQAIEMADIYGNVLHSM 5jtg.1    EEVLERPEKETVQRTHQLKRNLVELRKTIWPLREVLSSLYRDVPPL-IEKETVPYFRKVYDHTIQIADTVETFRDIVSGL  target    TETFASIISNNQNNIMKTLALVTIVMSIPTMVFSAYGMNFKDNEIPLNGEPNAFWLIVFIAFAMSVSLTLYLIHKKWF 5jtg.1    LDVYLSSVSNKTNEVMKVLTIIATIFMPLTFIAGIYGMNFEY--MPELRWKWGYPVVLAVMGVIAVIMVVYFKKKKWL ``` | | | | | | | | | | | | | | | | | | | | | | | | | | | | | | | | | | | | | | | | | | | | | | | | | |
|  | 5jtg.1.D | Cobalt/magnesium transport protein CorA  *Crystal structure of Thermotoga maritima mutant D89K/D253K* | 0.61 |  | 16.44 | 0.93 | 18-314 | X-ray | 3.05 | homo-pentamer | 3 x MG | HHblits | 0.30 |
| ``` target    MKQVFLSTTTEFKEIDTLEPGTWINLVNPTQ-NESLEIANTFDIDIADLRAPLDAEEMSRITIEDEYTLIIVDVPVTEER 5jtg.1    -----------------SSTPTWINITGIHRTDVVQRVGEFFGIHPLVLEKILNVHQRPKVEFFENYVFIVLKMFTYDKN  target    NNRTYYVTIPLGIIITEETIITTCLEPLPVLDVFINRRLRNFY---TFMRSRFIFQILYRNAELYLTALRSIDRKSEQIE 5jtg.1    L--HELESEQVSLILTKNCVLMFQEKIGDVFDPVRERIRYNRGIIRKKRADYLLYSLIDALVDDYFVLLEKIDDEIDVLE  target    SQLHQSTRNEELIELMELEKTIVYFKASLKTNERVIKKLTSSTSNIKKYLEDEDLLEDTLIETQQAIEMADIYGNVLHSM 5jtg.1    EEVLERPEKETVQRTHQLKRNLVELRKTIWPLREVLSSLYRDVPPL-IEKETVPYFRKVYDHTIQIADTVETFRDIVSGL  target    TETFASIISNNQNNIMKTLALVTIVMSIPTMVFSAYGMNFKDNEIPLNGEPNAFWLIVFIAFAMSVSLTLYLIHKKWF 5jtg.1    LDVYLSSVSNKTNEVMKVLTIIATIFMPLTFIAGIYGMNFEY--MPELRWKWGYPVVLAVMGVIAVIMVVYFKKKKWL ``` | | | | | | | | | | | | | | | | | | | | | | | | | | | | | | | | | | | | | | | | | | | | | | | | | |
|  | 5jrw.1.B | Cobalt/magnesium transport protein CorA  *Crystal structure of Thermotoga maritima mutant D89R/D253R* | 0.63 |  | 16.44 | 0.93 | 18-314 | X-ray | 3.30 | homo-pentamer | 5 x MG | HHblits | 0.30 |
| ``` target    MKQVFLSTTTEFKEIDTLEPGTWINLVNPTQN-ESLEIANTFDIDIADLRAPLDAEEMSRITIEDEYTLIIVDVPVTEER 5jrw.1    -----------------SSTPTWINITGIHRTDVVQRVGEFFGIHPLVLERILNVHQRPKVEFFENYVFIVLKMFTYDKN  target    NNRTYYVTIPLGIIITEETIITTCLEPLPVLDVFINRRLRNFY---TFMRSRFIFQILYRNAELYLTALRSIDRKSEQIE 5jrw.1    L--HELESEQVSLILTKNCVLMFQEKIGDVFDPVRERIRYNRGIIRKKRADYLLYSLIDALVDDYFVLLEKIDDEIDVLE  target    SQLHQSTRNEELIELMELEKTIVYFKASLKTNERVIKKLTSSTSNIKKYLEDEDLLEDTLIETQQAIEMADIYGNVLHSM 5jrw.1    EEVLERPEKETVQRTHQLKRNLVELRKTIWPLREVLSSLYRDVPPL-IEKETVPYFRRVYDHTIQIADTVETFRDIVSGL  target    TETFASIISNNQNNIMKTLALVTIVMSIPTMVFSAYGMNFKDNEIPLNGEPNAFWLIVFIAFAMSVSLTLYLIHKKWF 5jrw.1    LDVYLSSVSNKTNEVMKVLTIIATIFMPLTFIAGIYGMNFEY--MPELRWKWGYPVVLAVMGVIAVIMVVYFKKKKWL ``` | | | | | | | | | | | | | | | | | | | | | | | | | | | | | | | | | | | | | | | | | | | | | | | | | |
|  | 5jrw.1.A | Cobalt/magnesium transport protein CorA  *Crystal structure of Thermotoga maritima mutant D89R/D253R* | 0.63 |  | 16.44 | 0.93 | 18-314 | X-ray | 3.30 | homo-pentamer | 5 x MG | HHblits | 0.30 |
| ``` target    MKQVFLSTTTEFKEIDTLEPGTWINLVNPTQN-ESLEIANTFDIDIADLRAPLDAEEMSRITIEDEYTLIIVDVPVTEER 5jrw.1    -----------------SSTPTWINITGIHRTDVVQRVGEFFGIHPLVLERILNVHQRPKVEFFENYVFIVLKMFTYDKN  target    NNRTYYVTIPLGIIITEETIITTCLEPLPVLDVFINRRLRNFY---TFMRSRFIFQILYRNAELYLTALRSIDRKSEQIE 5jrw.1    L--HELESEQVSLILTKNCVLMFQEKIGDVFDPVRERIRYNRGIIRKKRADYLLYSLIDALVDDYFVLLEKIDDEIDVLE  target    SQLHQSTRNEELIELMELEKTIVYFKASLKTNERVIKKLTSSTSNIKKYLEDEDLLEDTLIETQQAIEMADIYGNVLHSM 5jrw.1    EEVLERPEKETVQRTHQLKRNLVELRKTIWPLREVLSSLYRDVPPL-IEKETVPYFRRVYDHTIQIADTVETFRDIVSGL  target    TETFASIISNNQNNIMKTLALVTIVMSIPTMVFSAYGMNFKDNEIPLNGEPNAFWLIVFIAFAMSVSLTLYLIHKKWF 5jrw.1    LDVYLSSVSNKTNEVMKVLTIIATIFMPLTFIAGIYGMNFEY--MPELRWKWGYPVVLAVMGVIAVIMVVYFKKKKWL ``` | | | | | | | | | | | | | | | | | | | | | | | | | | | | | | | | | | | | | | | | | | | | | | | | | |
|  | 5jrw.1.C | Cobalt/magnesium transport protein CorA  *Crystal structure of Thermotoga maritima mutant D89R/D253R* | 0.63 |  | 16.44 | 0.93 | 18-314 | X-ray | 3.30 | homo-pentamer | 5 x MG | HHblits | 0.30 |
| ``` target    MKQVFLSTTTEFKEIDTLEPGTWINLVNPTQN-ESLEIANTFDIDIADLRAPLDAEEMSRITIEDEYTLIIVDVPVTEER 5jrw.1    -----------------SSTPTWINITGIHRTDVVQRVGEFFGIHPLVLERILNVHQRPKVEFFENYVFIVLKMFTYDKN  target    NNRTYYVTIPLGIIITEETIITTCLEPLPVLDVFINRRLRNFY---TFMRSRFIFQILYRNAELYLTALRSIDRKSEQIE 5jrw.1    L--HELESEQVSLILTKNCVLMFQEKIGDVFDPVRERIRYNRGIIRKKRADYLLYSLIDALVDDYFVLLEKIDDEIDVLE  target    SQLHQSTRNEELIELMELEKTIVYFKASLKTNERVIKKLTSSTSNIKKYLEDEDLLEDTLIETQQAIEMADIYGNVLHSM 5jrw.1    EEVLERPEKETVQRTHQLKRNLVELRKTIWPLREVLSSLYRDVPPL-IEKETVPYFRRVYDHTIQIADTVETFRDIVSGL  target    TETFASIISNNQNNIMKTLALVTIVMSIPTMVFSAYGMNFKDNEIPLNGEPNAFWLIVFIAFAMSVSLTLYLIHKKWF 5jrw.1    LDVYLSSVSNKTNEVMKVLTIIATIFMPLTFIAGIYGMNFEY--MPELRWKWGYPVVLAVMGVIAVIMVVYFKKKKWL ``` | | | | | | | | | | | | | | | | | | | | | | | | | | | | | | | | | | | | | | | | | | | | | | | | | |
|  | 5jrw.1.D | Cobalt/magnesium transport protein CorA  *Crystal structure of Thermotoga maritima mutant D89R/D253R* | 0.64 |  | 16.44 | 0.93 | 18-314 | X-ray | 3.30 | homo-pentamer | 5 x MG | HHblits | 0.30 |
| ``` target    MKQVFLSTTTEFKEIDTLEPGTWINLVNPTQN-ESLEIANTFDIDIADLRAPLDAEEMSRITIEDEYTLIIVDVPVTEER 5jrw.1    -----------------SSTPTWINITGIHRTDVVQRVGEFFGIHPLVLERILNVHQRPKVEFFENYVFIVLKMFTYDKN  target    NNRTYYVTIPLGIIITEETIITTCLEPLPVLDVFINRRLRNFY---TFMRSRFIFQILYRNAELYLTALRSIDRKSEQIE 5jrw.1    L--HELESEQVSLILTKNCVLMFQEKIGDVFDPVRERIRYNRGIIRKKRADYLLYSLIDALVDDYFVLLEKIDDEIDVLE  target    SQLHQSTRNEELIELMELEKTIVYFKASLKTNERVIKKLTSSTSNIKKYLEDEDLLEDTLIETQQAIEMADIYGNVLHSM 5jrw.1    EEVLERPEKETVQRTHQLKRNLVELRKTIWPLREVLSSLYRDVPPL-IEKETVPYFRRVYDHTIQIADTVETFRDIVSGL  target    TETFASIISNNQNNIMKTLALVTIVMSIPTMVFSAYGMNFKDNEIPLNGEPNAFWLIVFIAFAMSVSLTLYLIHKKWF 5jrw.1    LDVYLSSVSNKTNEVMKVLTIIATIFMPLTFIAGIYGMNFEY--MPELRWKWGYPVVLAVMGVIAVIMVVYFKKKKWL ``` | | | | | | | | | | | | | | | | | | | | | | | | | | | | | | | | | | | | | | | | | | | | | | | | | |
|  | 5n9y.1.A | Zinc transport protein ZntB  *The full-length structure of ZntB* | 0.57 | 0.30 | 14.43 | 0.93 | 17-314 | EM | 0.00 | homo-pentamer |  | HHblits | 0.27 |
| ``` target    MKQVFLSTTTEFKEIDTLEPGTWINLVNPTQNESLEIANTFDIDIADLRAPLD-AEEMSRITIEDEYTLIIVDVPVTEER 5n9y.1    ----------------DEAHPCWLHLNYVHHDSAQWLAT-TPLLPNNVRDALAGESTRPRVSRLGEGTLITLRCINGSTD  target    NNRTYYVTIPLGIIITEETIITTCLEPLPVLDVFINRRLRNFYTFMRSRFIFQILYRNAELYLTALRSIDRKSEQIESQL 5n9y.1    E--RPDQLVAMRVYMDGRLIVSTRQRKVLALDDVVSDLEEGTGPTDCGGWLVDVCDALTDHSSEFIEQLHDKIIDLEDNL  target    HQSTRNEELIELMELEKTIVYFKASLKTNERVIKKLTSSTSNIKKYLEDEDLLEDTLIETQQAIEMADIYGNVLHSMTET 5n9y.1    LDQQI-PPRGFLALLRKQLIVMRRYMAPQRDVYARLASERLPW-MSDDQRRRMQDIADRLGRGLDEIDACIARTGVMADE  target    FASIISNNQNNIMKTLALVTIVMSIPTMVFSAYGMNFKDNEIPLNGEPNAFWLIVFIAFAMSVSLTLYLIHKKWF 5n9y.1    IAQVMQENLARRTYTMSLMAMVFLPSTFLTGLFGVNLGG--IPGGGWQFGFSIFCILLVVLIGGVALWLHRSKWL ``` | | | | | | | | | | | | | | | | | | | | | | | | | | | | | | | | | | | | | | | | | | | | | | | | | |
|  | 5n9y.1.B | Zinc transport protein ZntB  *The full-length structure of ZntB* | 0.58 | 0.30 | 14.43 | 0.93 | 17-314 | EM | 0.00 | homo-pentamer |  | HHblits | 0.27 |
| ``` target    MKQVFLSTTTEFKEIDTLEPGTWINLVNPTQNESLEIANTFDIDIADLRAPLD-AEEMSRITIEDEYTLIIVDVPVTEER 5n9y.1    ----------------DEAHPCWLHLNYVHHDSAQWLAT-TPLLPNNVRDALAGESTRPRVSRLGEGTLITLRCINGSTD  target    NNRTYYVTIPLGIIITEETIITTCLEPLPVLDVFINRRLRNFYTFMRSRFIFQILYRNAELYLTALRSIDRKSEQIESQL 5n9y.1    E--RPDQLVAMRVYMDGRLIVSTRQRKVLALDDVVSDLEEGTGPTDCGGWLVDVCDALTDHSSEFIEQLHDKIIDLEDNL  target    HQSTRNEELIELMELEKTIVYFKASLKTNERVIKKLTSSTSNIKKYLEDEDLLEDTLIETQQAIEMADIYGNVLHSMTET 5n9y.1    LDQQI-PPRGFLALLRKQLIVMRRYMAPQRDVYARLASERLPW-MSDDQRRRMQDIADRLGRGLDEIDACIARTGVMADE  target    FASIISNNQNNIMKTLALVTIVMSIPTMVFSAYGMNFKDNEIPLNGEPNAFWLIVFIAFAMSVSLTLYLIHKKWF 5n9y.1    IAQVMQENLARRTYTMSLMAMVFLPSTFLTGLFGVNLGG--IPGGGWQFGFSIFCILLVVLIGGVALWLHRSKWL ``` | | | | | | | | | | | | | | | | | | | | | | | | | | | | | | | | | | | | | | | | | | | | | | | | | |
|  | 5n9y.1.C | Zinc transport protein ZntB  *The full-length structure of ZntB* | 0.58 | 0.30 | 14.43 | 0.93 | 17-314 | EM | 0.00 | homo-pentamer |  | HHblits | 0.27 |
| ``` target    MKQVFLSTTTEFKEIDTLEPGTWINLVNPTQNESLEIANTFDIDIADLRAPLD-AEEMSRITIEDEYTLIIVDVPVTEER 5n9y.1    ----------------DEAHPCWLHLNYVHHDSAQWLAT-TPLLPNNVRDALAGESTRPRVSRLGEGTLITLRCINGSTD  target    NNRTYYVTIPLGIIITEETIITTCLEPLPVLDVFINRRLRNFYTFMRSRFIFQILYRNAELYLTALRSIDRKSEQIESQL 5n9y.1    E--RPDQLVAMRVYMDGRLIVSTRQRKVLALDDVVSDLEEGTGPTDCGGWLVDVCDALTDHSSEFIEQLHDKIIDLEDNL  target    HQSTRNEELIELMELEKTIVYFKASLKTNERVIKKLTSSTSNIKKYLEDEDLLEDTLIETQQAIEMADIYGNVLHSMTET 5n9y.1    LDQQI-PPRGFLALLRKQLIVMRRYMAPQRDVYARLASERLPW-MSDDQRRRMQDIADRLGRGLDEIDACIARTGVMADE  target    FASIISNNQNNIMKTLALVTIVMSIPTMVFSAYGMNFKDNEIPLNGEPNAFWLIVFIAFAMSVSLTLYLIHKKWF 5n9y.1    IAQVMQENLARRTYTMSLMAMVFLPSTFLTGLFGVNLGG--IPGGGWQFGFSIFCILLVVLIGGVALWLHRSKWL ``` | | | | | | | | | | | | | | | | | | | | | | | | | | | | | | | | | | | | | | | | | | | | | | | | | |
|  | 5n9y.1.D | Zinc transport protein ZntB  *The full-length structure of ZntB* | 0.58 | 0.30 | 14.43 | 0.93 | 17-314 | EM | 0.00 | homo-pentamer |  | HHblits | 0.27 |
| ``` target    MKQVFLSTTTEFKEIDTLEPGTWINLVNPTQNESLEIANTFDIDIADLRAPLD-AEEMSRITIEDEYTLIIVDVPVTEER 5n9y.1    ----------------DEAHPCWLHLNYVHHDSAQWLAT-TPLLPNNVRDALAGESTRPRVSRLGEGTLITLRCINGSTD  target    NNRTYYVTIPLGIIITEETIITTCLEPLPVLDVFINRRLRNFYTFMRSRFIFQILYRNAELYLTALRSIDRKSEQIESQL 5n9y.1    E--RPDQLVAMRVYMDGRLIVSTRQRKVLALDDVVSDLEEGTGPTDCGGWLVDVCDALTDHSSEFIEQLHDKIIDLEDNL  target    HQSTRNEELIELMELEKTIVYFKASLKTNERVIKKLTSSTSNIKKYLEDEDLLEDTLIETQQAIEMADIYGNVLHSMTET 5n9y.1    LDQQI-PPRGFLALLRKQLIVMRRYMAPQRDVYARLASERLPW-MSDDQRRRMQDIADRLGRGLDEIDACIARTGVMADE  target    FASIISNNQNNIMKTLALVTIVMSIPTMVFSAYGMNFKDNEIPLNGEPNAFWLIVFIAFAMSVSLTLYLIHKKWF 5n9y.1    IAQVMQENLARRTYTMSLMAMVFLPSTFLTGLFGVNLGG--IPGGGWQFGFSIFCILLVVLIGGVALWLHRSKWL ``` | | | | | | | | | | | | | | | | | | | | | | | | | | | | | | | | | | | | | | | | | | | | | | | | | |
|  | 5n9y.1.E | Zinc transport protein ZntB  *The full-length structure of ZntB* | 0.57 | 0.30 | 14.43 | 0.93 | 17-314 | EM | 0.00 | homo-pentamer |  | HHblits | 0.27 |
| ``` target    MKQVFLSTTTEFKEIDTLEPGTWINLVNPTQNESLEIANTFDIDIADLRAPLD-AEEMSRITIEDEYTLIIVDVPVTEER 5n9y.1    ----------------DEAHPCWLHLNYVHHDSAQWLAT-TPLLPNNVRDALAGESTRPRVSRLGEGTLITLRCINGSTD  target    NNRTYYVTIPLGIIITEETIITTCLEPLPVLDVFINRRLRNFYTFMRSRFIFQILYRNAELYLTALRSIDRKSEQIESQL 5n9y.1    E--RPDQLVAMRVYMDGRLIVSTRQRKVLALDDVVSDLEEGTGPTDCGGWLVDVCDALTDHSSEFIEQLHDKIIDLEDNL  target    HQSTRNEELIELMELEKTIVYFKASLKTNERVIKKLTSSTSNIKKYLEDEDLLEDTLIETQQAIEMADIYGNVLHSMTET 5n9y.1    LDQQI-PPRGFLALLRKQLIVMRRYMAPQRDVYARLASERLPW-MSDDQRRRMQDIADRLGRGLDEIDACIARTGVMADE  target    FASIISNNQNNIMKTLALVTIVMSIPTMVFSAYGMNFKDNEIPLNGEPNAFWLIVFIAFAMSVSLTLYLIHKKWF 5n9y.1    IAQVMQENLARRTYTMSLMAMVFLPSTFLTGLFGVNLGG--IPGGGWQFGFSIFCILLVVLIGGVALWLHRSKWL ``` | | | | | | | | | | | | | | | | | | | | | | | | | | | | | | | | | | | | | | | | | | | | | | | | | |
|  | 4ev6.1.A | Magnesium transport protein CorA  *The complete structure of CorA magnesium transporter from Methanocaldococcus jannaschii* | 0.53 | 0.41 | 31.47 | 0.80 | 23-294 | X-ray | 3.20 | homo-pentamer | 8 x UMQ, 28 x MG | BLAST | 0.36 |
| ``` target    MKQVFLSTTTEFKEIDTLEPGTWINLVNPTQNESLEIANTFDIDIADLRAPLDAEEMSRITIEDEYTLIIVDVPVTEERN 4ev6.1    ----------------------WIDCYDPKDEELYKLSKKIGISVSDLQIGLDEQEIPRVEEDEDFYLIIYKAPLFEED-  target    NRTYYVTIPLGIIITEETIITTCLEPLPVLDVFINRRLRNFYTFMRSRFIFQILYRNAELYL-TALRSIDRKSEQI---- 4ev6.1    ----ITTTSLGIYIKNNLLLTIHSDKIKAIG-----RLHKLISTKKPRIVFE---RGIGFLLYHILNEITRSYSRILMNL  target    ------ESQLHQSTRNEELIE-LMELEKTIVYFKASLKTNERVIKKLTSSTSNIKKYLEDEDLLEDTLIE-----TQQAI 4ev6.1    EDELEELEDKLLAGYDREVMEKILGLRKTLVYFHKSLIANRDVLVLLK------RKYLPITTKEDRENFEDLYYDTLQLI  target    EMADIYGNVLHSMTETFASIISNNQNNIMKTLALVTIVMSIPTMVFSAYGMNFKDNEIPLNGEPNAFWLIVFIAFAMSVS 4ev6.1    DMSATYREVLTSMMDITLSLENIKMNQIMKILTMVTTIFAVPMWITGIYGMNF--SYLPLANNPQGFWLVM---------  target    LTLYLIHKKWF 4ev6.1    ----------- ``` | | | | | | | | | | | | | | | | | | | | | | | | | | | | | | | | | | | | | | | | | | | | | | | | | |
|  | 4ev6.1.B | Magnesium transport protein CorA  *The complete structure of CorA magnesium transporter from Methanocaldococcus jannaschii* | 0.52 | 0.41 | 31.47 | 0.80 | 23-294 | X-ray | 3.20 | homo-pentamer | 8 x UMQ, 28 x MG | BLAST | 0.36 |
| ``` target    MKQVFLSTTTEFKEIDTLEPGTWINLVNPTQNESLEIANTFDIDIADLRAPLDAEEMSRITIEDEYTLIIVDVPVTEERN 4ev6.1    ----------------------WIDCYDPKDEELYKLSKKIGISVSDLQIGLDEQEIPRVEEDEDFYLIIYKAPLFEED-  target    NRTYYVTIPLGIIITEETIITTCLEPLPVLDVFINRRLRNFYTFMRSRFIFQILYRNAELYL-TALRSIDRKSEQI---- 4ev6.1    ----ITTTSLGIYIKNNLLLTIHSDKIKAIG-----RLHKLISTKKPRIVFE---RGIGFLLYHILNEITRSYSRILMNL  target    ------ESQLHQSTRNEELIE-LMELEKTIVYFKASLKTNERVIKKLTSSTSNIKKYLEDEDLLEDTLIE-----TQQAI 4ev6.1    EDELEELEDKLLAGYDREVMEKILGLRKTLVYFHKSLIANRDVLVLLK------RKYLPITTKEDRENFEDLYYDTLQLI  target    EMADIYGNVLHSMTETFASIISNNQNNIMKTLALVTIVMSIPTMVFSAYGMNFKDNEIPLNGEPNAFWLIVFIAFAMSVS 4ev6.1    DMSATYREVLTSMMDITLSLENIKMNQIMKILTMVTTIFAVPMWITGIYGMNF--SYLPLANNPQGFWLVM---------  target    LTLYLIHKKWF 4ev6.1    ----------- ``` | | | | | | | | | | | | | | | | | | | | | | | | | | | | | | | | | | | | | | | | | | | | | | | | | |
|  | 4ev6.1.C | Magnesium transport protein CorA  *The complete structure of CorA magnesium transporter from Methanocaldococcus jannaschii* | 0.53 | 0.41 | 31.47 | 0.80 | 23-294 | X-ray | 3.20 | homo-pentamer | 8 x UMQ, 28 x MG | BLAST | 0.36 |
| ``` target    MKQVFLSTTTEFKEIDTLEPGTWINLVNPTQNESLEIANTFDIDIADLRAPLDAEEMSRITIEDEYTLIIVDVPVTEERN 4ev6.1    ----------------------WIDCYDPKDEELYKLSKKIGISVSDLQIGLDEQEIPRVEEDEDFYLIIYKAPLFEED-  target    NRTYYVTIPLGIIITEETIITTCLEPLPVLDVFINRRLRNFYTFMRSRFIFQILYRNAELYL-TALRSIDRKSEQI---- 4ev6.1    ----ITTTSLGIYIKNNLLLTIHSDKIKAIG-----RLHKLISTKKPRIVFE---RGIGFLLYHILNEITRSYSRILMNL  target    ------ESQLHQSTRNEELIE-LMELEKTIVYFKASLKTNERVIKKLTSSTSNIKKYLEDEDLLEDTLIE-----TQQAI 4ev6.1    EDELEELEDKLLAGYDREVMEKILGLRKTLVYFHKSLIANRDVLVLLK------RKYLPITTKEDRENFEDLYYDTLQLI  target    EMADIYGNVLHSMTETFASIISNNQNNIMKTLALVTIVMSIPTMVFSAYGMNFKDNEIPLNGEPNAFWLIVFIAFAMSVS 4ev6.1    DMSATYREVLTSMMDITLSLENIKMNQIMKILTMVTTIFAVPMWITGIYGMNF--SYLPLANNPQGFWLVM---------  target    LTLYLIHKKWF 4ev6.1    ----------- ``` | | | | | | | | | | | | | | | | | | | | | | | | | | | | | | | | | | | | | | | | | | | | | | | | | |
|  | 4ev6.1.D | Magnesium transport protein CorA  *The complete structure of CorA magnesium transporter from Methanocaldococcus jannaschii* | 0.52 | 0.41 | 31.47 | 0.80 | 23-294 | X-ray | 3.20 | homo-pentamer | 8 x UMQ, 28 x MG | BLAST | 0.36 |
| ``` target    MKQVFLSTTTEFKEIDTLEPGTWINLVNPTQNESLEIANTFDIDIADLRAPLDAEEMSRITIEDEYTLIIVDVPVTEERN 4ev6.1    ----------------------WIDCYDPKDEELYKLSKKIGISVSDLQIGLDEQEIPRVEEDEDFYLIIYKAPLFEED-  target    NRTYYVTIPLGIIITEETIITTCLEPLPVLDVFINRRLRNFYTFMRSRFIFQILYRNAELYL-TALRSIDRKSEQI---- 4ev6.1    ----ITTTSLGIYIKNNLLLTIHSDKIKAIG-----RLHKLISTKKPRIVFE---RGIGFLLYHILNEITRSYSRILMNL  target    ------ESQLHQSTRNEELIE-LMELEKTIVYFKASLKTNERVIKKLTSSTSNIKKYLEDEDLLEDTLIE-----TQQAI 4ev6.1    EDELEELEDKLLAGYDREVMEKILGLRKTLVYFHKSLIANRDVLVLLK------RKYLPITTKEDRENFEDLYYDTLQLI  target    EMADIYGNVLHSMTETFASIISNNQNNIMKTLALVTIVMSIPTMVFSAYGMNFKDNEIPLNGEPNAFWLIVFIAFAMSVS 4ev6.1    DMSATYREVLTSMMDITLSLENIKMNQIMKILTMVTTIFAVPMWITGIYGMNF--SYLPLANNPQGFWLVM---------  target    LTLYLIHKKWF 4ev6.1    ----------- ``` | | | | | | | | | | | | | | | | | | | | | | | | | | | | | | | | | | | | | | | | | | | | | | | | | |
|  | 4ev6.1.E | Magnesium transport protein CorA  *The complete structure of CorA magnesium transporter from Methanocaldococcus jannaschii* | 0.53 | 0.41 | 31.47 | 0.80 | 23-294 | X-ray | 3.20 | homo-pentamer | 8 x UMQ, 28 x MG | BLAST | 0.36 |
| ``` target    MKQVFLSTTTEFKEIDTLEPGTWINLVNPTQNESLEIANTFDIDIADLRAPLDAEEMSRITIEDEYTLIIVDVPVTEERN 4ev6.1    ----------------------WIDCYDPKDEELYKLSKKIGISVSDLQIGLDEQEIPRVEEDEDFYLIIYKAPLFEED-  target    NRTYYVTIPLGIIITEETIITTCLEPLPVLDVFINRRLRNFYTFMRSRFIFQILYRNAELYL-TALRSIDRKSEQI---- 4ev6.1    ----ITTTSLGIYIKNNLLLTIHSDKIKAIG-----RLHKLISTKKPRIVFE---RGIGFLLYHILNEITRSYSRILMNL  target    ------ESQLHQSTRNEELIE-LMELEKTIVYFKASLKTNERVIKKLTSSTSNIKKYLEDEDLLEDTLIE-----TQQAI 4ev6.1    EDELEELEDKLLAGYDREVMEKILGLRKTLVYFHKSLIANRDVLVLLK------RKYLPITTKEDRENFEDLYYDTLQLI  target    EMADIYGNVLHSMTETFASIISNNQNNIMKTLALVTIVMSIPTMVFSAYGMNFKDNEIPLNGEPNAFWLIVFIAFAMSVS 4ev6.1    DMSATYREVLTSMMDITLSLENIKMNQIMKILTMVTTIFAVPMWITGIYGMNF--SYLPLANNPQGFWLVM---------  target    LTLYLIHKKWF 4ev6.1    ----------- ``` | | | | | | | | | | | | | | | | | | | | | | | | | | | | | | | | | | | | | | | | | | | | | | | | | |
|  | 5n77.1.A | Magnesium transport protein CorA  *Crystal structure of the cytosolic domain of the CorA magnesium channel from Escherichia coli in complex with magnesium* | 0.53 | 0.30 | 16.19 | 0.79 | 1-253 | X-ray | 2.80 | homo-pentamer | 6 x MG | HHblits | 0.28 |
| ``` target    MKQVFLSTTTEFKEI-----DTLEPGTWINLVNPTQNESLEIANTFDIDIADLRAPLDAEEMSRITIEDEYTLIIVDVPV 5n77.1    MLSAFQLENNRLTRLEVEESQPLVNAVWIDLVEPDDDERLRVQSELGQSLATRPELEDIEASARFFEDDDGLHIHSFFFF  target    TEERNNRTYYVTIPLGIIITEETIITTCLEPLPVLDVFINRRLRNF-YTFMRSRFIFQILYRNAELYLTALRSIDRKSEQ 5n77.1    EDAE---DHAGNSTVAFTIRDGRLFTLRERELPAFRLYRMRARSQSMVDGNAYELLLDLFETKIEQLADEIENIYSDLEQ  target    IESQLHQSTR----NEELIELMELEKTIVYFKASLKTNERVIKKLTSSTSNIKKYLEDEDLLEDTLIETQQAIEMADIYG 5n77.1    LSRVIMEGHQGDEYDEALSTLAELEDIGWKVRLCLMDTQRALNFLVRKAR-L--PGGQLEQAREILRDIESLLPHNESLF  target    NVLHSMTETFASIISNNQNNIMKTLALVTIVMSIPTMVFSAYGMNFKDNEIPLNGEPNAFWLIVFIAFAMSVSLTLYLIH 5n77.1    QKVNFLMQAAMGFINIEQNRIIK---------------------------------------------------------  target    KKWF 5n77.1    ---- ``` | | | | | | | | | | | | | | | | | | | | | | | | | | | | | | | | | | | | | | | | | | | | | | | | | |
|  | 4egw.1.A | Magnesium transport protein CorA  *The structure of the soluble domain of CorA from Methanocaldococcus jannaschii* | 0.55 | 0.06 | 26.41 | 0.74 | 17-253 | X-ray | 2.50 | homo-dimer | 13 x HEZ, 3 x MG, 7 x PGO | HHblits | 0.33 |
| ``` target    MKQVFLSTTTEFKEIDTLEPGTWINLVNPTQNESLEIANTFDIDIADLRAPLDAEEMSRITIEDEYTLIIVDVPVTEERN 4egw.1    ----------------EDYRLIWIDCYDPKDEELYKLSKKIGISVSDLQIGLDEQEIPRVEEDEDFYLIIYKAPLFEE--  target    NRTYYVTIPLGIIITEETIITTCLEPLPVLDVFINRRLRNFY----TFMRSRFIFQILYRNAELYLTALRSIDRKSEQIE 4egw.1    ---DITTTSLGIYIKNNLLLTIHSDKIKAIGRLHKLISTKKPRIVFERGIGFLLYHILNEITRSYSRILMNLEDELEELE  target    SQLHQSTRNEELIELMELEKTIVYFKASLKTNERVIKKLTSSTSNIKKYLEDEDLLEDTLIETQQAIEMADIYGNVLHSM 4egw.1    DKLLAGYDREVMEKILGLRKTLVYFHKSLIANRDVLVLLKRKYLPI-TTKEDRENFEDLYYDTLQLIDMSATYREVLTSM  target    TETFASIISNNQNNIMKTLALVTIVMSIPTMVFSAYGMNFKDNEIPLNGEPNAFWLIVFIAFAMSVSLTLYLIHKKWF 4egw.1    MDITLSLENIKMNQIMK------------------------------------------------------------- ``` | | | | | | | | | | | | | | | | | | | | | | | | | | | | | | | | | | | | | | | | | | | | | | | | | |
|  | 4egw.1.B | Magnesium transport protein CorA  *The structure of the soluble domain of CorA from Methanocaldococcus jannaschii* | 0.55 | 0.06 | 26.41 | 0.74 | 17-253 | X-ray | 2.50 | homo-dimer | 13 x HEZ, 3 x MG, 7 x PGO | HHblits | 0.33 |
| ``` target    MKQVFLSTTTEFKEIDTLEPGTWINLVNPTQNESLEIANTFDIDIADLRAPLDAEEMSRITIEDEYTLIIVDVPVTEERN 4egw.1    ----------------EDYRLIWIDCYDPKDEELYKLSKKIGISVSDLQIGLDEQEIPRVEEDEDFYLIIYKAPLFEE--  target    NRTYYVTIPLGIIITEETIITTCLEPLPVLDVFINRRLRNFY----TFMRSRFIFQILYRNAELYLTALRSIDRKSEQIE 4egw.1    ---DITTTSLGIYIKNNLLLTIHSDKIKAIGRLHKLISTKKPRIVFERGIGFLLYHILNEITRSYSRILMNLEDELEELE  target    SQLHQSTRNEELIELMELEKTIVYFKASLKTNERVIKKLTSSTSNIKKYLEDEDLLEDTLIETQQAIEMADIYGNVLHSM 4egw.1    DKLLAGYDREVMEKILGLRKTLVYFHKSLIANRDVLVLLKRKYLPI-TTKEDRENFEDLYYDTLQLIDMSATYREVLTSM  target    TETFASIISNNQNNIMKTLALVTIVMSIPTMVFSAYGMNFKDNEIPLNGEPNAFWLIVFIAFAMSVSLTLYLIHKKWF 4egw.1    MDITLSLENIKMNQIMK------------------------------------------------------------- ``` | | | | | | | | | | | | | | | | | | | | | | | | | | | | | | | | | | | | | | | | | | | | | | | | | |
|  | 3ck6.1.A | Putative membrane transport protein  *Crystal structure of ZntB cytoplasmic domain from Vibrio parahaemolyticus RIMD 2210633* | 0.45 | 0.14 | 12.55 | 0.76 | 1-251 | X-ray | 1.90 | homo-pentamer |  | HHblits | 0.26 |
| ``` target    MKQVFLSTTT---EFKEID-TLEPGTWINLVNPTQNESLEIANTFDIDIADLRAPLDAEEMSRITIEDE-YTLIIVDVPV 3ck6.1    MIEHWDFSTPMATQETTTAEHIQPNHWYHCERLHP-DIRGWLEDNHVPRATVDHLLADESRPSFHPLDDDNFMLILRGIN  target    TEERNNRTYYVTIPLGIIITEETIITTCLEPLPVLDVFINRRLRNFYTFMRSRFIFQILYRNAELYLTALRSIDRKSEQI 3ck6.1    MNENA--SPEDMLSIRILYFQGALISTRKIPSRAIMEIRQALAEHK---GPKS-LASLLNQIIEGLNGKIDLY---LDTI  target    ESQLHQSTRN-EELIELMELEKTIVYFKASLKTNERVIKKLTSSTSNIKKYLEDEDLLEDTLIETQQAIEMADIYGNVLH 3ck6.1    EETLNEFDVNDESTYNHIAAQKALISIKRFIRPQQYAIRDLIESESELV--TSRPHQYRFAHNNITRINETIEFYLGEVA  target    SMTETFASIISNNQNNIMKTLALVTIVMSIPTMVFSAYGMNFKDNEIPLNGEPNAFWLIVFIAFAMSVSLTLYLIHKKWF 3ck6.1    LFQDEIKHNRDEKTNKN--------------------------------------------------------------- ``` | | | | | | | | | | | | | | | | | | | | | | | | | | | | | | | | | | | | | | | | | | | | | | | | | |
|  | 3ck6.1.B | Putative membrane transport protein  *Crystal structure of ZntB cytoplasmic domain from Vibrio parahaemolyticus RIMD 2210633* | 0.45 | 0.14 | 12.55 | 0.76 | 1-251 | X-ray | 1.90 | homo-pentamer |  | HHblits | 0.26 |
| ``` target    MKQVFLSTTT---EFKEID-TLEPGTWINLVNPTQNESLEIANTFDIDIADLRAPLDAEEMSRITIEDE-YTLIIVDVPV 3ck6.1    MIEHWDFSTPMATQETTTAEHIQPNHWYHCERLHP-DIRGWLEDNHVPRATVDHLLADESRPSFHPLDDDNFMLILRGIN  target    TEERNNRTYYVTIPLGIIITEETIITTCLEPLPVLDVFINRRLRNFYTFMRSRFIFQILYRNAELYLTALRSIDRKSEQI 3ck6.1    MNENA--SPEDMLSIRILYFQGALISTRKIPSRAIMEIRQALAEHK---GPKS-LASLLNQIIEGLNGKIDLY---LDTI  target    ESQLHQSTRN-EELIELMELEKTIVYFKASLKTNERVIKKLTSSTSNIKKYLEDEDLLEDTLIETQQAIEMADIYGNVLH 3ck6.1    EETLNEFDVNDESTYNHIAAQKALISIKRFIRPQQYAIRDLIESESELV--TSRPHQYRFAHNNITRINETIEFYLGEVA  target    SMTETFASIISNNQNNIMKTLALVTIVMSIPTMVFSAYGMNFKDNEIPLNGEPNAFWLIVFIAFAMSVSLTLYLIHKKWF 3ck6.1    LFQDEIKHNRDEKTNKN--------------------------------------------------------------- ``` | | | | | | | | | | | | | | | | | | | | | | | | | | | | | | | | | | | | | | | | | | | | | | | | | |
|  | 3ck6.1.E | Putative membrane transport protein  *Crystal structure of ZntB cytoplasmic domain from Vibrio parahaemolyticus RIMD 2210633* | 0.45 | 0.14 | 12.55 | 0.76 | 1-251 | X-ray | 1.90 | homo-pentamer |  | HHblits | 0.26 |
| ``` target    MKQVFLSTTT---EFKEID-TLEPGTWINLVNPTQNESLEIANTFDIDIADLRAPLDAEEMSRITIEDE-YTLIIVDVPV 3ck6.1    MIEHWDFSTPMATQETTTAEHIQPNHWYHCERLHP-DIRGWLEDNHVPRATVDHLLADESRPSFHPLDDDNFMLILRGIN  target    TEERNNRTYYVTIPLGIIITEETIITTCLEPLPVLDVFINRRLRNFYTFMRSRFIFQILYRNAELYLTALRSIDRKSEQI 3ck6.1    MNENA--SPEDMLSIRILYFQGALISTRKIPSRAIMEIRQALAEHK---GPKS-LASLLNQIIEGLNGKIDLY---LDTI  target    ESQLHQSTRN-EELIELMELEKTIVYFKASLKTNERVIKKLTSSTSNIKKYLEDEDLLEDTLIETQQAIEMADIYGNVLH 3ck6.1    EETLNEFDVNDESTYNHIAAQKALISIKRFIRPQQYAIRDLIESESELV--TSRPHQYRFAHNNITRINETIEFYLGEVA  target    SMTETFASIISNNQNNIMKTLALVTIVMSIPTMVFSAYGMNFKDNEIPLNGEPNAFWLIVFIAFAMSVSLTLYLIHKKWF 3ck6.1    LFQDEIKHNRDEKTNKN--------------------------------------------------------------- ``` | | | | | | | | | | | | | | | | | | | | | | | | | | | | | | | | | | | | | | | | | | | | | | | | | |
|  | 3nvo.1.A | Zinc transport protein zntB  *The Soluble Domain Structure of the ZntB Zn2+ Efflux System* | 0.45 | 0.02 | 12.44 | 0.72 | 17-246 | X-ray | 2.30 | homo-dimer | 8 x ZN | HHblits | 0.26 |
| ``` target    MKQVFLSTTTEFKEIDTLEPGTWINLVNPTQNESLEIANTFDIDIADLRAPLD-AEEMSRITIEDEYTLIIVDVPVTEER 3nvo.1    ----------------DSQHPCWLHLNYTHPDSARWLAS-TPLLPNNVRDALAGESSRPRVSRMGEGTLITLRCINGSTD  target    NNRTYYVTIPLGIIITEETIITTCLEPLPVLDVFINRRLRNFYTFMRSRFIFQILYRNAELYLTALRSIDRKSEQIESQL 3nvo.1    E--RPDQLVAMRLYMDERFIVSTRQRKVLALDDVVSDLQEGTGPVDCGGWLVDVCDALTDHASEFIEELHDKIIDLEDNL  target    HQSTRNEELIELMELEKTIVYFKASLKTNERVIKKLTSSTSNIKKYLEDEDLLEDTLIETQQAIEMADIYGNVLHSMTET 3nvo.1    LDQQI-PPRGFLALLRKQLIVMRRYMAPQRDVYARLASERLPW-MSDDHRRRMQDIADRLGRGLDEIDACIARTGIMADE  target    FASIISNNQNNIMKTLALVTIVMSIPTMVFSAYGMNFKDNEIPLNGEPNAFWLIVFIAFAMSVSLTLYLIHKKWF 3nvo.1    IAQVMQE-------------------------------------------------------------------- ``` | | | | | | | | | | | | | | | | | | | | | | | | | | | | | | | | | | | | | | | | | | | | | | | | | |
|  | 3nwi.1.A | Zinc transport protein zntB  *The Soluble Domain Structure of the ZntB Zn2+ Efflux System* | 0.42 | 0.10 | 12.50 | 0.71 | 18-246 | X-ray | 3.13 | homo-pentamer | 15 x ZN | HHblits | 0.26 |
| ``` target    MKQVFLSTTTEFKEIDTLEPGTWINLVNPTQNESLEIANTFDIDIADLRAPLD-AEEMSRITIEDEYTLIIVDVPVTEER 3nwi.1    -----------------SQHPCWLHLNYTHPDSARWLAS-TPLLPNNVRDALAGESSRPRVSRMGEGTLITLRCINGSTD  target    NNRTYYVTIPLGIIITEETIITTCLEPLPVLDVFINRRLRNFYTFMRSRFIFQILYRNAELYLTALRSIDRKSEQIESQL 3nwi.1    E--RPDQLVAMRLYMDERFIVSTRQRKVLALDDVVSDLQEGTGPVDCGGWLVDVCDALTDHASEFIEELHDKIIDLEDNL  target    HQSTRNEELIELMELEKTIVYFKASLKTNERVIKKLTSSTSNIKKYLEDEDLLEDTLIETQQAIEMADIYGNVLHSMTET 3nwi.1    LDQQI-PPRGFLALLRKQLIVMRRYMAPQRDVYARLASERLPW-MSDDHRRRMQDIADRLGRGLDEIDACIARTGIMADE  target    FASIISNNQNNIMKTLALVTIVMSIPTMVFSAYGMNFKDNEIPLNGEPNAFWLIVFIAFAMSVSLTLYLIHKKWF 3nwi.1    IAQVMQE-------------------------------------------------------------------- ``` | | | | | | | | | | | | | | | | | | | | | | | | | | | | | | | | | | | | | | | | | | | | | | | | | |
|  | 2bbh.1.A | divalent cation transport-related protein  *X-ray structure of T.maritima CorA soluble domain* | 0.35 |  | 14.01 | 0.66 | 18-227 | X-ray | 1.85 | monomer | 4 x DMU, 1 x MG | HHblits | 0.28 |
| ``` target    MKQVFLSTTTEFKEIDTLEPGTWINLVNPTQ-NESLEIANTFDIDIADLRAPLDAEEMSRITIEDEYTLIIVDVPVTEER 2bbh.1    -----------------SSTPTWINITGIHRTDVVQRVGEFFGTHPLVLEDILNVHQRPKVEFFENYVFIVLKMFTYDKN  target    NNRTYYVTIPLGIIITEETIITTCLEPLPVLDVFINRRLRNFY---TFMRSRFIFQILYRNAELYLTALRSIDRKSEQIE 2bbh.1    L--HELESEQVSLILTKNCVLMFQEKIGDVFDPVRERIRYNRGIIRKKRADYLLYSLIDALVDDYFVLLEKIDDEIDVLE  target    SQLHQSTRNEELIELMELEKTIVYFKASLKTNERVIKKLTSSTSNIKKYLEDEDLLEDTLIETQQAIEMADIYGNVLHSM 2bbh.1    EEVLERPEKETVQRTHQLKRNLVELRKTIWPLREVLSSLYRDVPPL-IEKETVPYFRDVYDHTIQIADTVE---------  target    TETFASIISNNQNNIMKTLALVTIVMSIPTMVFSAYGMNFKDNEIPLNGEPNAFWLIVFIAFAMSVSLTLYLIHKKWF 2bbh.1    ------------------------------------------------------------------------------ ``` | | | | | | | | | | | | | | | | | | | | | | | | | | | | | | | | | | | | | | | | | | | | | | | | | |
|  | 2hn1.1.A | Magnesium and cobalt transporter  *Crystal structure of a CorA soluble domain from A. fulgidus in complex with Co2+* | 0.38 | 0.25 | 15.50 | 0.64 | 18-224 | X-ray | 2.90 | homo-dimer | 2 x CO | HHblits | 0.28 |
| ``` target    MKQVFLSTTTEFKEIDTLEPGTWINLVNPTQN-ESLEIANTFDIDIADLRAPLDAEEMSRITIEDEYTLIIVDVPVTEER 2hn1.1    -----------------LNKKLWIDVVGVHDESLIAKICEFLGIHPLAAEDILNTAQRVKIEDYDDHLFLVLKILLYNE-  target    NNRTYYVTIPLGIIITEETIITTCLEPLPVLDVFINRRLRNFY--TFMRSRFIFQILYRNAELYLTALRSIDRKSEQIES 2hn1.1    ----TLEIDQLSLVLKKNLVATFEEREYWILDSIRSRLKSGGRMRKLAGDYLAYTILDAVVDSYFEALLKISDEIEVLED  target    QLHQSTRNEELIELMELEKTIVYFKASLKTNERVIKKLTSSTSNIKKYLEDEDLLEDTLIETQQAIEMADIYGNVLHSMT 2hn1.1    EVVSGD-STLIGKIHSLKREILAFRNAVWPLRDVLSFFTRVEHEL-IGEEVKVYYRDVYDHAVRLME-------------  target    ETFASIISNNQNNIMKTLALVTIVMSIPTMVFSAYGMNFKDNEIPLNGEPNAFWLIVFIAFAMSVSLTLYLIHKKWF 2hn1.1    ----------------------------------------------------------------------------- ``` | | | | | | | | | | | | | | | | | | | | | | | | | | | | | | | | | | | | | | | | | | | | | | | | | |
|  | 3rkg.1.A | Magnesium transporter MRS2, mitochondrial  *Structural and Functional Characterization of the Yeast Mg2+ Channel Mrs2* | 0.26 |  | 11.45 | 0.53 | 19-200 | X-ray | 1.28 | monomer |  | HHblits | 0.25 |
| ``` target    MKQVFLSTTTEFKEIDTLEPGTWINLVNPTQN-------ESLEIANTFDIDIADLRAPLDA--EEMSRITIEDEYTLIIV 3rkg.1    ------------------LFISCTVFNSKGNIISMSEKFPKWSFLTEHSLFPRDLRKIDNSSIDIIPTIMCKPNCIV--I  target    DVPVTEERNNRTYYVTIPLGIIITEETIITTCLEPL---PVLDVFINRR---LRNF-YTFMRS--RFIFQILYRNAELYL 3rkg.1    NLL--------------HIKALIERDKVYVFDTTNPSAAAKLSVLMYDLESKLSSTKNNSQFYEHRALESIFINVMSALE  target    TALRSIDRKSEQIESQLHQSTRNEELIELMELEKTIVYFKASLKTNERVIKKLTSSTSNIKKYLEDEDLLEDTLIETQQA 3rkg.1    TDFKLHSQICIQILNDLENEVNRLKLRHLLIKSKDLTLFYQKTLLIRDLLDELLENDD----------------------  target    IEMADIYGNVLHSMTETFASIISNNQNNIMKTLALVTIVMSIPTMVFSAYGMNFKDNEIPLNGEPNAFWLIVFIAFAMSV 3rkg.1    --------------------------------------------------------------------------------  target    SLTLYLIHKKWF 3rkg.1    ------------ ``` | | | | | | | | | | | | | | | | | | | | | | | | | | | | | | | | | | | | | | | | | | | | | | | | | |
|  | 6yrg.1.A | Vegetative insecticidal protein  *Vip3Bc1 tetramer in processed, activated state* | 0.05 | 0.00 | 15.56 | 0.29 | 168-260 | EM | 7.00 | homo-tetramer |  | HHblits | 0.28 |
| ``` target    MKQVFLSTTTEFKEIDTLEPGTWINLVNPTQNESLEIANTFDIDIADLRAPLDAEEMSRITIEDEYTLIIVDVPVTEERN 6yrg.1    --------------------------------------------------------------------------------  target    NRTYYVTIPLGIIITEETIITTCLEPLPVLDVFINRRLRNFYTFMRSRFIFQILYRNAELYLTALRSIDRKSEQIESQLH 6yrg.1    --------------------------------------------------------------------------------  target    QSTRNEELIELMELEKTIVYFKASLKTNERVIKKLTSSTSNIKKYLEDEDLLEDTLIETQQAIEMADIYGNVLHSMTETF 6yrg.1    -------LDEILKNQNLLNDISGKLDGINGDLGDLIAQGN-L--NSELAKELLKISNEQNQMLNHVNAQLNAINSTLNIY  target    ASIISNNQNNIMKTLALVTIVMSIPTMVFSAYGMNFKDNEIPLNGEPNAFWLIVFIAFAMSVSLTLYLIHKKWF 6yrg.1    LPKITSMLNEVMKQNHVLSL------------------------------------------------------ ``` | | | | | | | | | | | | | | | | | | | | | | | | | | | | | | | | | | | | | | | | | | | | | | | | | |
|  | 6yrf.1.B | Vegetative insecticidal protein  *Vip3Bc1 tetramer* | 0.10 | 0.00 | 15.56 | 0.29 | 168-260 | EM | 0.00 | homo-tetramer |  | HHblits | 0.28 |
| ``` target    MKQVFLSTTTEFKEIDTLEPGTWINLVNPTQNESLEIANTFDIDIADLRAPLDAEEMSRITIEDEYTLIIVDVPVTEERN 6yrf.1    --------------------------------------------------------------------------------  target    NRTYYVTIPLGIIITEETIITTCLEPLPVLDVFINRRLRNFYTFMRSRFIFQILYRNAELYLTALRSIDRKSEQIESQLH 6yrf.1    --------------------------------------------------------------------------------  target    QSTRNEELIELMELEKTIVYFKASLKTNERVIKKLTSSTSNIKKYLEDEDLLEDTLIETQQAIEMADIYGNVLHSMTETF 6yrf.1    -------LDEILKNQNLLNDISGKLDGINGDLGDLIAQGN-L--NSELAKELLKISNEQNQMLNHVNAQLNAINSTLNIY  target    ASIISNNQNNIMKTLALVTIVMSIPTMVFSAYGMNFKDNEIPLNGEPNAFWLIVFIAFAMSVSLTLYLIHKKWF 6yrf.1    LPKITSMLNEVMKQNHVLSL------------------------------------------------------ ``` | | | | | | | | | | | | | | | | | | | | | | | | | | | | | | | | | | | | | | | | | | | | | | | | | |
|  | 6yrg.1.B | Vegetative insecticidal protein  *Vip3Bc1 tetramer in processed, activated state* | 0.05 | 0.00 | 15.56 | 0.29 | 168-260 | EM | 7.00 | homo-tetramer |  | HHblits | 0.28 |
| ``` target    MKQVFLSTTTEFKEIDTLEPGTWINLVNPTQNESLEIANTFDIDIADLRAPLDAEEMSRITIEDEYTLIIVDVPVTEERN 6yrg.1    --------------------------------------------------------------------------------  target    NRTYYVTIPLGIIITEETIITTCLEPLPVLDVFINRRLRNFYTFMRSRFIFQILYRNAELYLTALRSIDRKSEQIESQLH 6yrg.1    --------------------------------------------------------------------------------  target    QSTRNEELIELMELEKTIVYFKASLKTNERVIKKLTSSTSNIKKYLEDEDLLEDTLIETQQAIEMADIYGNVLHSMTETF 6yrg.1    -------LDEILKNQNLLNDISGKLDGINGDLGDLIAQGN-L--NSELAKELLKISNEQNQMLNHVNAQLNAINSTLNIY  target    ASIISNNQNNIMKTLALVTIVMSIPTMVFSAYGMNFKDNEIPLNGEPNAFWLIVFIAFAMSVSLTLYLIHKKWF 6yrg.1    LPKITSMLNEVMKQNHVLSL------------------------------------------------------ ``` | | | | | | | | | | | | | | | | | | | | | | | | | | | | | | | | | | | | | | | | | | | | | | | | | |
|  | 6yrf.1.A | Vegetative insecticidal protein  *Vip3Bc1 tetramer* | 0.11 | 0.00 | 15.56 | 0.29 | 168-260 | EM | 0.00 | homo-tetramer |  | HHblits | 0.28 |
| ``` target    MKQVFLSTTTEFKEIDTLEPGTWINLVNPTQNESLEIANTFDIDIADLRAPLDAEEMSRITIEDEYTLIIVDVPVTEERN 6yrf.1    --------------------------------------------------------------------------------  target    NRTYYVTIPLGIIITEETIITTCLEPLPVLDVFINRRLRNFYTFMRSRFIFQILYRNAELYLTALRSIDRKSEQIESQLH 6yrf.1    --------------------------------------------------------------------------------  target    QSTRNEELIELMELEKTIVYFKASLKTNERVIKKLTSSTSNIKKYLEDEDLLEDTLIETQQAIEMADIYGNVLHSMTETF 6yrf.1    -------LDEILKNQNLLNDISGKLDGINGDLGDLIAQGN-L--NSELAKELLKISNEQNQMLNHVNAQLNAINSTLNIY  target    ASIISNNQNNIMKTLALVTIVMSIPTMVFSAYGMNFKDNEIPLNGEPNAFWLIVFIAFAMSVSLTLYLIHKKWF 6yrf.1    LPKITSMLNEVMKQNHVLSL------------------------------------------------------ ``` | | | | | | | | | | | | | | | | | | | | | | | | | | | | | | | | | | | | | | | | | | | | | | | | | |
|  | 6v1v.1.A | Vegetative insecticidal protein  *VIP3B (VIP3B\_2160) adapted for crystallization* | 0.11 | 0.00 | 15.56 | 0.29 | 168-260 | X-ray | 3.19 | homo-tetramer |  | HHblits | 0.28 |
| ``` target    MKQVFLSTTTEFKEIDTLEPGTWINLVNPTQNESLEIANTFDIDIADLRAPLDAEEMSRITIEDEYTLIIVDVPVTEERN 6v1v.1    --------------------------------------------------------------------------------  target    NRTYYVTIPLGIIITEETIITTCLEPLPVLDVFINRRLRNFYTFMRSRFIFQILYRNAELYLTALRSIDRKSEQIESQLH 6v1v.1    --------------------------------------------------------------------------------  target    QSTRNEELIELMELEKTIVYFKASLKTNERVIKKLTSSTSNIKKYLEDEDLLEDTLIETQQAIEMADIYGNVLHSMTETF 6v1v.1    -------LDEILKNQNLLNDISGKLDGINGDLGDLIAQGN-L--NSELAKELLKISNEQNQMLNHVNAQLNAINSTLNIY  target    ASIISNNQNNIMKTLALVTIVMSIPTMVFSAYGMNFKDNEIPLNGEPNAFWLIVFIAFAMSVSLTLYLIHKKWF 6v1v.1    LPKITSMLNEVMKQNHVLSL------------------------------------------------------ ``` | | | | | | | | | | | | | | | | | | | | | | | | | | | | | | | | | | | | | | | | | | | | | | | | | |
|  | 6v1v.1.C | Vegetative insecticidal protein  *VIP3B (VIP3B\_2160) adapted for crystallization* | 0.12 | 0.00 | 15.56 | 0.29 | 168-260 | X-ray | 3.19 | homo-tetramer |  | HHblits | 0.28 |
| ``` target    MKQVFLSTTTEFKEIDTLEPGTWINLVNPTQNESLEIANTFDIDIADLRAPLDAEEMSRITIEDEYTLIIVDVPVTEERN 6v1v.1    --------------------------------------------------------------------------------  target    NRTYYVTIPLGIIITEETIITTCLEPLPVLDVFINRRLRNFYTFMRSRFIFQILYRNAELYLTALRSIDRKSEQIESQLH 6v1v.1    --------------------------------------------------------------------------------  target    QSTRNEELIELMELEKTIVYFKASLKTNERVIKKLTSSTSNIKKYLEDEDLLEDTLIETQQAIEMADIYGNVLHSMTETF 6v1v.1    -------LDEILKNQNLLNDISGKLDGINGDLGDLIAQGN-L--NSELAKELLKISNEQNQMLNHVNAQLNAINSTLNIY  target    ASIISNNQNNIMKTLALVTIVMSIPTMVFSAYGMNFKDNEIPLNGEPNAFWLIVFIAFAMSVSLTLYLIHKKWF 6v1v.1    LPKITSMLNEVMKQNHVLSL------------------------------------------------------ ``` | | | | | | | | | | | | | | | | | | | | | | | | | | | | | | | | | | | | | | | | | | | | | | | | | |
|  | 6v1v.1.D | Vegetative insecticidal protein  *VIP3B (VIP3B\_2160) adapted for crystallization* | 0.12 | 0.00 | 15.56 | 0.29 | 168-260 | X-ray | 3.19 | homo-tetramer |  | HHblits | 0.28 |
| ``` target    MKQVFLSTTTEFKEIDTLEPGTWINLVNPTQNESLEIANTFDIDIADLRAPLDAEEMSRITIEDEYTLIIVDVPVTEERN 6v1v.1    --------------------------------------------------------------------------------  target    NRTYYVTIPLGIIITEETIITTCLEPLPVLDVFINRRLRNFYTFMRSRFIFQILYRNAELYLTALRSIDRKSEQIESQLH 6v1v.1    --------------------------------------------------------------------------------  target    QSTRNEELIELMELEKTIVYFKASLKTNERVIKKLTSSTSNIKKYLEDEDLLEDTLIETQQAIEMADIYGNVLHSMTETF 6v1v.1    -------LDEILKNQNLLNDISGKLDGINGDLGDLIAQGN-L--NSELAKELLKISNEQNQMLNHVNAQLNAINSTLNIY  target    ASIISNNQNNIMKTLALVTIVMSIPTMVFSAYGMNFKDNEIPLNGEPNAFWLIVFIAFAMSVSLTLYLIHKKWF 6v1v.1    LPKITSMLNEVMKQNHVLSL------------------------------------------------------ ``` | | | | | | | | | | | | | | | | | | | | | | | | | | | | | | | | | | | | | | | | | | | | | | | | | |
| ✓ | 6b7n.1.A | Spike protein  *Cryo-electron microscopy structure of porcine delta coronavirus spike protein in the pre-fusion state* | 0.09 | 0.00 | 14.29 | 0.27 | 137-227 | EM | 0.00 | homo-trimer | 24 x NAG, 12 x NAG-NAG, 3 x NAG-NAG-NAG | HHblits | 0.28 |
| ``` target    MKQVFLSTTTEFKEIDTLEPGTWINLVNPTQNESLEIANTFDIDIADLRAPLDAEEMSRITIEDEYTLIIVDVPVTEERN 6b7n.1    --------------------------------------------------------------------------------  target    NRTYYVTIPLGIIITEETIITTCLEPLPVLDVFINRRLRNFYTFMRSRFIFQILYRNAELYLTALRSIDRKSEQIESQLH 6b7n.1    --------------------------------------------------------LAESFNQAVGNISLALSSVNDAIQ  target    QSTRNEELIELMELEKTIVYFKASLKTNERVIKKLTSSTSNIKKYLEDEDLLEDTLIETQQAIEMADIYGNVLHSMTETF 6b7n.1    QTS-----EALNTVAIAIKKIQTVVNQQGEALSHLTAQLSN--NFQAISTSIQDIYNRLEEVEANQQ-------------  target    ASIISNNQNNIMKTLALVTIVMSIPTMVFSAYGMNFKDNEIPLNGEPNAFWLIVFIAFAMSVSLTLYLIHKKWF 6b7n.1    -------------------------------------------------------------------------- ``` | | | | | | | | | | | | | | | | | | | | | | | | | | | | | | | | | | | | | | | | | | | | | | | | | |
|  | 1t7s.2.A | BAG-1 cochaperone  *Structural Genomics of Caenorhabditis elegans: Structure of BAG-1 protein* | 0.06 | 0.00 | 13.64 | 0.21 | 171-236 | X-ray | 2.80 | monomer |  | HHblits | 0.28 |
| ``` target    MKQVFLSTTTEFKEIDTLEPGTWINLVNPTQNESLEIANTFDIDIADLRAPLDAEEMSRITIEDEYTLIIVDVPVTEERN 1t7s.2    --------------------------------------------------------------------------------  target    NRTYYVTIPLGIIITEETIITTCLEPLPVLDVFINRRLRNFYTFMRSRFIFQILYRNAELYLTALRSIDRKSEQIESQLH 1t7s.2    --------------------------------------------------------------------------------  target    QSTRNEELIELMELEKTIVYFKASLKTNERVIKKLTSSTSN-IK-KYLEDEDLLEDTLIETQQAIEMADIYGNVLHSMTE 1t7s.2    ----------GKKLEKKVKYFNEEAERHLETLDGMNIITETTPENQAKRNREKRKTLVNGIQTLLNQNDALLRRLQEY--  target    TFASIISNNQNNIMKTLALVTIVMSIPTMVFSAYGMNFKDNEIPLNGEPNAFWLIVFIAFAMSVSLTLYLIHKKWF 1t7s.2    ---------------------------------------------------------------------------- ``` | | | | | | | | | | | | | | | | | | | | | | | | | | | | | | | | | | | | | | | | | | | | | | | | | |
|  | 1t7s.1.A | BAG-1 cochaperone  *Structural Genomics of Caenorhabditis elegans: Structure of BAG-1 protein* | 0.06 | 0.00 | 13.64 | 0.21 | 171-236 | X-ray | 2.80 | monomer |  | HHblits | 0.28 |
| ``` target    MKQVFLSTTTEFKEIDTLEPGTWINLVNPTQNESLEIANTFDIDIADLRAPLDAEEMSRITIEDEYTLIIVDVPVTEERN 1t7s.1    --------------------------------------------------------------------------------  target    NRTYYVTIPLGIIITEETIITTCLEPLPVLDVFINRRLRNFYTFMRSRFIFQILYRNAELYLTALRSIDRKSEQIESQLH 1t7s.1    --------------------------------------------------------------------------------  target    QSTRNEELIELMELEKTIVYFKASLKTNERVIKKLTSSTSN-IK-KYLEDEDLLEDTLIETQQAIEMADIYGNVLHSMTE 1t7s.1    ----------GKKLEKKVKYFNEEAERHLETLDGMNIITETTPENQAKRNREKRKTLVNGIQTLLNQNDALLRRLQEY--  target    TFASIISNNQNNIMKTLALVTIVMSIPTMVFSAYGMNFKDNEIPLNGEPNAFWLIVFIAFAMSVSLTLYLIHKKWF 1t7s.1    ---------------------------------------------------------------------------- ``` | | | | | | | | | | | | | | | | | | | | | | | | | | | | | | | | | | | | | | | | | | | | | | | | | |
|  | 6zyw.1.B | Outer arm dynein beta heavy chain  *Outer Dynein Arm-Shulin complex - overall structure (Tetrahymena thermophila)* | 0.06 |  | 7.46 | 0.21 | 131-197 | EM | 0.00 | hetero-1-1-1-2-2-1-… | 3 x ADP, 1 x ATP, 1 x GTP | HHblits | 0.26 |
| ``` target    MKQVFLSTTTEFKEIDTLEPGTWINLVNPTQNESLEIANTFDIDIADLRAPLDAEEMSRITIEDEYTLIIVDVPVTEERN 6zyw.1    --------------------------------------------------------------------------------  target    NRTYYVTIPLGIIITEETIITTCLEPLPVLDVFINRRLRNFYTFMRSRFIFQILYRNAELYLTALRSIDRKSEQIESQLH 6zyw.1    --------------------------------------------------RVYTDFLVNQFRTTQKNLLDFIEKTKDGIK  target    QSTRN-E----------ELIELMELEKTIVYFKASLKTNERVIKKLTSSTSNIKKYLEDEDLLEDTLIETQQAIEMADIY 6zyw.1    KNPADHENLHDKKLLMSVMKVISDVKDVEPRREGIITRMKEMVTKLKK--------------------------------  target    GNVLHSMTETFASIISNNQNNIMKTLALVTIVMSIPTMVFSAYGMNFKDNEIPLNGEPNAFWLIVFIAFAMSVSLTLYLI 6zyw.1    --------------------------------------------------------------------------------  target    HKKWF 6zyw.1    ----- ``` | | | | | | | | | | | | | | | | | | | | | | | | | | | | | | | | | | | | | | | | | | | | | | | | | |
|  | 1i6z.1.A | BAG-FAMILY MOLECULAR CHAPERONE REGULATOR-1  *BAG DOMAIN OF BAG1 COCHAPERONE* | 0.06 |  | 8.06 | 0.20 | 172-237 | NMR | 0.00 | monomer |  | HHblits | 0.25 |
| ``` target    MKQVFLSTTTEFKEIDTLEPGTWINLVNPTQNESLEIANTFDIDIADLRAPLDAEEMSRITIEDEYTLIIVDVPVTEERN 1i6z.1    --------------------------------------------------------------------------------  target    NRTYYVTIPLGIIITEETIITTCLEPLPVLDVFINRRLRNFYTFMRSRFIFQILYRNAELYLTALRSIDRKSEQIESQLH 1i6z.1    --------------------------------------------------------------------------------  target    QSTRNEELIELMELEKTIVYFKASLKTNERVIKKLTSSTSNIKKYLEDEDLLEDTLIETQQAIEMADIYGNVLHSMTETF 1i6z.1    -----------CKLDRKVKATIEQFMKILEEIDTMVLPE----QFKDSRLKRKNLVKKVQVFLAECDTVEQYICQET---  target    ASIISNNQNNIMKTLALVTIVMSIPTMVFSAYGMNFKDNEIPLNGEPNAFWLIVFIAFAMSVSLTLYLIHKKWF 1i6z.1    -------------------------------------------------------------------------- ``` | | | | | | | | | | | | | | | | | | | | | | | | | | | | | | | | | | | | | | | | | | | | | | | | | |
|  | 1quu.1.A | HUMAN SKELETAL MUSCLE ALPHA-ACTININ 2  *CRYSTAL STRUCTURE OF TWO CENTRAL SPECTRIN-LIKE REPEATS FROM ALPHA-ACTININ* | 0.05 | 0.00 | 16.95 | 0.19 | 138-196 | X-ray | 2.50 | homo-dimer |  | HHblits | 0.28 |
| ``` target    MKQVFLSTTTEFKEIDTLEPGTWINLVNPTQNESLEIANTFDIDIADLRAPLDAEEMSRITIEDEYTLIIVDVPVTEERN 1quu.1    --------------------------------------------------------------------------------  target    NRTYYVTIPLGIIITEETIITTCLEPLPVLDVFINRRLRNFYTFMRSRFIFQILYRNAELYLTALRSIDRKSEQIESQLH 1quu.1    ---------------------------------------------------------AEKFRQKASTHETWAYGKEQILL  target    QSTRN-EELIELMELEKTIVYFKASLKTNERVIKKLTSSTSNIKKYLEDEDLLEDTLIETQQAIEMADIYGNVLHSMTET 1quu.1    QKDYESASLTEVRALLRKHEAFESDLAAHQDRVEQIA-------------------------------------------  target    FASIISNNQNNIMKTLALVTIVMSIPTMVFSAYGMNFKDNEIPLNGEPNAFWLIVFIAFAMSVSLTLYLIHKKWF 1quu.1    --------------------------------------------------------------------------- ``` | | | | | | | | | | | | | | | | | | | | | | | | | | | | | | | | | | | | | | | | | | | | | | | | | |
|  | 2ncj.1.A | Uncharacterized protein  *Solution Structure of the PriC DNA replication restart protein* | 0.06 | 0.00 | 11.86 | 0.19 | 135-193 | NMR | 0.00 | monomer |  | HHblits | 0.28 |
| ``` target    MKQVFLSTTTEFKEIDTLEPGTWINLVNPTQNESLEIANTFDIDIADLRAPLDAEEMSRITIEDEYTLIIVDVPVTEERN 2ncj.1    --------------------------------------------------------------------------------  target    NRTYYVTIPLGIIITEETIITTCLEPLPVLDVFINRRLRNFYTFMRSRFIFQILYRNAELYLTALRSIDRKSEQIESQLH 2ncj.1    ------------------------------------------------------HARLAEYQEYERRLLAMKNEREQRYA  target    QSTRNEELIELMELEKTIVYFKASLKTNERVIKKLTSSTSNIKKYLEDEDLLEDTLIETQQAIEMADIYGNVLHSMTETF 2ncj.1    ERHDPQLAREITALDERLTRCRTAIARTERALE-----------------------------------------------  target    ASIISNNQNNIMKTLALVTIVMSIPTMVFSAYGMNFKDNEIPLNGEPNAFWLIVFIAFAMSVSLTLYLIHKKWF 2ncj.1    -------------------------------------------------------------------------- ``` | | | | | | | | | | | | | | | | | | | | | | | | | | | | | | | | | | | | | | | | | | | | | | | | | |
|  | 5aqf.1.B | BAG FAMILY MOLECULAR CHAPERONE REGULATOR 1  *Fragment-based screening of HSP70 sheds light on the functional role of ATP-binding site residues* | 0.06 | 0.00 | 9.84 | 0.19 | 171-235 | X-ray | 1.88 | monomer | 1 x ADN | HHblits | 0.25 |
| ``` target    MKQVFLSTTTEFKEIDTLEPGTWINLVNPTQNESLEIANTFDIDIADLRAPLDAEEMSRITIEDEYTLIIVDVPVTEERN 5aqf.1    --------------------------------------------------------------------------------  target    NRTYYVTIPLGIIITEETIITTCLEPLPVLDVFINRRLRNFYTFMRSRFIFQILYRNAELYLTALRSIDRKSEQIESQLH 5aqf.1    --------------------------------------------------------------------------------  target    QSTRNEELIELMELEKTIVYFKASLKTNERVIKKLTSSTSNIKKYLEDEDLLEDTLIETQQAIEMADIYGNVLHSMTETF 5aqf.1    ----------LCKLDRRVKATIEQFMKILEEIDTLILPE----NFKDSRLKRKGLVKKVQAFLAECDTVEQNICQ-----  target    ASIISNNQNNIMKTLALVTIVMSIPTMVFSAYGMNFKDNEIPLNGEPNAFWLIVFIAFAMSVSLTLYLIHKKWF 5aqf.1    -------------------------------------------------------------------------- ``` | | | | | | | | | | | | | | | | | | | | | | | | | | | | | | | | | | | | | | | | | | | | | | | | | |
|  | 5aqf.2.B | BAG FAMILY MOLECULAR CHAPERONE REGULATOR 1  *Fragment-based screening of HSP70 sheds light on the functional role of ATP-binding site residues* | 0.06 | 0.00 | 9.84 | 0.19 | 171-235 | X-ray | 1.88 | monomer | 1 x ADN | HHblits | 0.25 |
| ``` target    MKQVFLSTTTEFKEIDTLEPGTWINLVNPTQNESLEIANTFDIDIADLRAPLDAEEMSRITIEDEYTLIIVDVPVTEERN 5aqf.2    --------------------------------------------------------------------------------  target    NRTYYVTIPLGIIITEETIITTCLEPLPVLDVFINRRLRNFYTFMRSRFIFQILYRNAELYLTALRSIDRKSEQIESQLH 5aqf.2    --------------------------------------------------------------------------------  target    QSTRNEELIELMELEKTIVYFKASLKTNERVIKKLTSSTSNIKKYLEDEDLLEDTLIETQQAIEMADIYGNVLHSMTETF 5aqf.2    ----------LCKLDRRVKATIEQFMKILEEIDTLILPE----NFKDSRLKRKGLVKKVQAFLAECDTVEQNICQ-----  target    ASIISNNQNNIMKTLALVTIVMSIPTMVFSAYGMNFKDNEIPLNGEPNAFWLIVFIAFAMSVSLTLYLIHKKWF 5aqf.2    -------------------------------------------------------------------------- ``` | | | | | | | | | | | | | | | | | | | | | | | | | | | | | | | | | | | | | | | | | | | | | | | | | |
|  | 5aqg.2.B | BAG FAMILY MOLECULAR CHAPERONE REGULATOR 1  *Fragment-based screening of HSP70 sheds light on the functional role of ATP-binding site residues* | 0.05 | 0.00 | 9.84 | 0.19 | 171-235 | X-ray | 2.24 | monomer | 1 x ZJB | HHblits | 0.25 |
| ``` target    MKQVFLSTTTEFKEIDTLEPGTWINLVNPTQNESLEIANTFDIDIADLRAPLDAEEMSRITIEDEYTLIIVDVPVTEERN 5aqg.2    --------------------------------------------------------------------------------  target    NRTYYVTIPLGIIITEETIITTCLEPLPVLDVFINRRLRNFYTFMRSRFIFQILYRNAELYLTALRSIDRKSEQIESQLH 5aqg.2    --------------------------------------------------------------------------------  target    QSTRNEELIELMELEKTIVYFKASLKTNERVIKKLTSSTSNIKKYLEDEDLLEDTLIETQQAIEMADIYGNVLHSMTETF 5aqg.2    ----------LCKLDRRVKATIEQFMKILEEIDTLILPE----NFKDSRLKRKGLVKKVQAFLAECDTVEQNICQ-----  target    ASIISNNQNNIMKTLALVTIVMSIPTMVFSAYGMNFKDNEIPLNGEPNAFWLIVFIAFAMSVSLTLYLIHKKWF 5aqg.2    -------------------------------------------------------------------------- ``` | | | | | | | | | | | | | | | | | | | | | | | | | | | | | | | | | | | | | | | | | | | | | | | | | |
|  | 5aqt.1.B | BAG FAMILY MOLECULAR CHAPERONE REGULATOR 1  *Fragment-based screening of HSP70 sheds light on the functional role of ATP-binding site residues* | 0.06 | 0.00 | 9.84 | 0.19 | 171-235 | X-ray | 1.90 | monomer | 1 x 5P7 | HHblits | 0.25 |
| ``` target    MKQVFLSTTTEFKEIDTLEPGTWINLVNPTQNESLEIANTFDIDIADLRAPLDAEEMSRITIEDEYTLIIVDVPVTEERN 5aqt.1    --------------------------------------------------------------------------------  target    NRTYYVTIPLGIIITEETIITTCLEPLPVLDVFINRRLRNFYTFMRSRFIFQILYRNAELYLTALRSIDRKSEQIESQLH 5aqt.1    --------------------------------------------------------------------------------  target    QSTRNEELIELMELEKTIVYFKASLKTNERVIKKLTSSTSNIKKYLEDEDLLEDTLIETQQAIEMADIYGNVLHSMTETF 5aqt.1    ----------LCKLDRRVKATIEQFMKILEEIDTLILPE----NFKDSRLKRKGLVKKVQAFLAECDTVEQNICQ-----  target    ASIISNNQNNIMKTLALVTIVMSIPTMVFSAYGMNFKDNEIPLNGEPNAFWLIVFIAFAMSVSLTLYLIHKKWF 5aqt.1    -------------------------------------------------------------------------- ``` | | | | | | | | | | | | | | | | | | | | | | | | | | | | | | | | | | | | | | | | | | | | | | | | | |
|  | 3fzh.2.A | BAG family molecular chaperone regulator 1  *Crystal Structures of Hsc70/Bag1 in Complex with Small Molecule Inhibitors* | 0.05 | 0.00 | 10.00 | 0.19 | 171-234 | X-ray | 2.00 | monomer |  | HHblits | 0.25 |
| ``` target    MKQVFLSTTTEFKEIDTLEPGTWINLVNPTQNESLEIANTFDIDIADLRAPLDAEEMSRITIEDEYTLIIVDVPVTEERN 3fzh.2    --------------------------------------------------------------------------------  target    NRTYYVTIPLGIIITEETIITTCLEPLPVLDVFINRRLRNFYTFMRSRFIFQILYRNAELYLTALRSIDRKSEQIESQLH 3fzh.2    --------------------------------------------------------------------------------  target    QSTRNEELIELMELEKTIVYFKASLKTNERVIKKLTSSTSNIKKYLEDEDLLEDTLIETQQAIEMADIYGNVLHSMTETF 3fzh.2    ----------LCKLDRRVKATIEQFMKILEEIDTLILPE----NFKDSRLKRKGLVKKVQAFLAECDTVEQNIC------  target    ASIISNNQNNIMKTLALVTIVMSIPTMVFSAYGMNFKDNEIPLNGEPNAFWLIVFIAFAMSVSLTLYLIHKKWF 3fzh.2    -------------------------------------------------------------------------- ``` | | | | | | | | | | | | | | | | | | | | | | | | | | | | | | | | | | | | | | | | | | | | | | | | | |
|  | 1hx1.1.B | BAG family molecular chaperone regulator 1  *CRYSTAL STRUCTURE OF A BAG DOMAIN IN COMPLEX WITH THE HSC70 ATPASE DOMAIN* | 0.05 | 0.00 | 10.00 | 0.19 | 171-234 | X-ray | 1.90 | monomer |  | HHblits | 0.25 |
| ``` target    MKQVFLSTTTEFKEIDTLEPGTWINLVNPTQNESLEIANTFDIDIADLRAPLDAEEMSRITIEDEYTLIIVDVPVTEERN 1hx1.1    --------------------------------------------------------------------------------  target    NRTYYVTIPLGIIITEETIITTCLEPLPVLDVFINRRLRNFYTFMRSRFIFQILYRNAELYLTALRSIDRKSEQIESQLH 1hx1.1    --------------------------------------------------------------------------------  target    QSTRNEELIELMELEKTIVYFKASLKTNERVIKKLTSSTSNIKKYLEDEDLLEDTLIETQQAIEMADIYGNVLHSMTETF 1hx1.1    ----------LCKLDRRVKATIEQFMKILEEIDTLILPE----NFKDSRLKRKGLVKKVQAFLAECDTVEQNIC------  target    ASIISNNQNNIMKTLALVTIVMSIPTMVFSAYGMNFKDNEIPLNGEPNAFWLIVFIAFAMSVSLTLYLIHKKWF 1hx1.1    -------------------------------------------------------------------------- ``` | | | | | | | | | | | | | | | | | | | | | | | | | | | | | | | | | | | | | | | | | | | | | | | | | |
|  | 3ghg.1.B | Fibrinogen beta chain  *Crystal Structure of Human Fibrinogen* | 0.05 |  | 15.79 | 0.18 | 139-195 | X-ray | 2.90 | hetero-2-2-2-mer | 4 x CA, 2 x GLY-PRO-ARG-PRO, 2 x GLY-HIS-ARG-PRO, 2 x NAG-NDG-BMA-MAN-NDG-GAL-SIA-MAN-NDG-GAL-SIA | HHblits | 0.29 |
| ``` target    MKQVFLSTTTEFKEIDTLEPGTWINLVNPTQNESLEIANTFDIDIADLRAPLDAEEMSRITIEDEYTLIIVDVPVTEERN 3ghg.1    --------------------------------------------------------------------------------  target    NRTYYVTIPLGIIITEETIITTCLEPLPVLDVFINRRLRNFYTFMRSRFIFQILYRNAELYLTALRSIDRKSEQIESQLH 3ghg.1    ----------------------------------------------------------EALLQQERPIRNSVDELNNNVE  target    QST--RNEELIELMELEKTIVYFKASLKTNERVIKKLTSSTSNIKKYLEDEDLLEDTLIETQQAIEMADIYGNVLHSMTE 3ghg.1    AVSQTSSSSFQYMYLLKDLWQKRQKQVKDNENVVNEY-------------------------------------------  target    TFASIISNNQNNIMKTLALVTIVMSIPTMVFSAYGMNFKDNEIPLNGEPNAFWLIVFIAFAMSVSLTLYLIHKKWF 3ghg.1    ---------------------------------------------------------------------------- ``` | | | | | | | | | | | | | | | | | | | | | | | | | | | | | | | | | | | | | | | | | | | | | | | | | |
|  | 3ghg.1.E | Fibrinogen beta chain  *Crystal Structure of Human Fibrinogen* | 0.04 |  | 15.79 | 0.18 | 139-195 | X-ray | 2.90 | hetero-2-2-2-mer | 4 x CA, 2 x GLY-PRO-ARG-PRO, 2 x GLY-HIS-ARG-PRO, 2 x NAG-NDG-BMA-MAN-NDG-GAL-SIA-MAN-NDG-GAL-SIA | HHblits | 0.29 |
| ``` target    MKQVFLSTTTEFKEIDTLEPGTWINLVNPTQNESLEIANTFDIDIADLRAPLDAEEMSRITIEDEYTLIIVDVPVTEERN 3ghg.1    --------------------------------------------------------------------------------  target    NRTYYVTIPLGIIITEETIITTCLEPLPVLDVFINRRLRNFYTFMRSRFIFQILYRNAELYLTALRSIDRKSEQIESQLH 3ghg.1    ----------------------------------------------------------EALLQQERPIRNSVDELNNNVE  target    QST--RNEELIELMELEKTIVYFKASLKTNERVIKKLTSSTSNIKKYLEDEDLLEDTLIETQQAIEMADIYGNVLHSMTE 3ghg.1    AVSQTSSSSFQYMYLLKDLWQKRQKQVKDNENVVNEY-------------------------------------------  target    TFASIISNNQNNIMKTLALVTIVMSIPTMVFSAYGMNFKDNEIPLNGEPNAFWLIVFIAFAMSVSLTLYLIHKKWF 3ghg.1    ---------------------------------------------------------------------------- ``` | | | | | | | | | | | | | | | | | | | | | | | | | | | | | | | | | | | | | | | | | | | | | | | | | |
|  | 3ghg.2.B | Fibrinogen beta chain  *Crystal Structure of Human Fibrinogen* | 0.05 |  | 15.79 | 0.18 | 139-195 | X-ray | 2.90 | hetero-2-2-2-mer | 4 x CA, 2 x GLY-PRO-ARG-PRO, 2 x GLY-HIS-ARG-PRO, 1 x NAG-NDG-BMA-MAN-MAN, 1 x NAG-NDG-BMA-MAN-NDG-GAL-SIA-MAN-NDG-GAL-SIA, 1 x NAG-NAG | HHblits | 0.29 |
| ``` target    MKQVFLSTTTEFKEIDTLEPGTWINLVNPTQNESLEIANTFDIDIADLRAPLDAEEMSRITIEDEYTLIIVDVPVTEERN 3ghg.2    --------------------------------------------------------------------------------  target    NRTYYVTIPLGIIITEETIITTCLEPLPVLDVFINRRLRNFYTFMRSRFIFQILYRNAELYLTALRSIDRKSEQIESQLH 3ghg.2    ----------------------------------------------------------EALLQQERPIRNSVDELNNNVE  target    QST--RNEELIELMELEKTIVYFKASLKTNERVIKKLTSSTSNIKKYLEDEDLLEDTLIETQQAIEMADIYGNVLHSMTE 3ghg.2    AVSQTSSSSFQYMYLLKDLWQKRQKQVKDNENVVNEY-------------------------------------------  target    TFASIISNNQNNIMKTLALVTIVMSIPTMVFSAYGMNFKDNEIPLNGEPNAFWLIVFIAFAMSVSLTLYLIHKKWF 3ghg.2    ---------------------------------------------------------------------------- ``` | | | | | | | | | | | | | | | | | | | | | | | | | | | | | | | | | | | | | | | | | | | | | | | | | |
|  | 3ghg.2.E | Fibrinogen beta chain  *Crystal Structure of Human Fibrinogen* | 0.05 |  | 15.79 | 0.18 | 139-195 | X-ray | 2.90 | hetero-2-2-2-mer | 4 x CA, 2 x GLY-PRO-ARG-PRO, 2 x GLY-HIS-ARG-PRO, 1 x NAG-NDG-BMA-MAN-MAN, 1 x NAG-NDG-BMA-MAN-NDG-GAL-SIA-MAN-NDG-GAL-SIA, 1 x NAG-NAG | HHblits | 0.29 |
| ``` target    MKQVFLSTTTEFKEIDTLEPGTWINLVNPTQNESLEIANTFDIDIADLRAPLDAEEMSRITIEDEYTLIIVDVPVTEERN 3ghg.2    --------------------------------------------------------------------------------  target    NRTYYVTIPLGIIITEETIITTCLEPLPVLDVFINRRLRNFYTFMRSRFIFQILYRNAELYLTALRSIDRKSEQIESQLH 3ghg.2    ----------------------------------------------------------EALLQQERPIRNSVDELNNNVE  target    QST--RNEELIELMELEKTIVYFKASLKTNERVIKKLTSSTSNIKKYLEDEDLLEDTLIETQQAIEMADIYGNVLHSMTE 3ghg.2    AVSQTSSSSFQYMYLLKDLWQKRQKQVKDNENVVNEY-------------------------------------------  target    TFASIISNNQNNIMKTLALVTIVMSIPTMVFSAYGMNFKDNEIPLNGEPNAFWLIVFIAFAMSVSLTLYLIHKKWF 3ghg.2    ---------------------------------------------------------------------------- ``` | | | | | | | | | | | | | | | | | | | | | | | | | | | | | | | | | | | | | | | | | | | | | | | | | |
|  | 3vpv.1.B | Tse2 specific immunity protein 2  *Crystal Structure of Pseudomonas aeruginosa Tsi2* | 0.06 |  | 14.55 | 0.18 | 144-202 | X-ray | 1.80 | homo-dimer |  | HHblits | 0.31 |
| ``` target    MKQVFLSTTTEFKEIDTLEPGTWINLVNPTQNESLEIANTFDIDIADLRAPLDAEEMSRITIEDEYTLIIVDVPVTEERN 3vpv.1    --------------------------------------------------------------------------------  target    NRTYYVTIPLGIIITEETIITTCLEPLPVLDVFINRRLRNFYTFMRSRFIFQILYRNAELYLTALRSIDRKSEQIESQLH 3vpv.1    ---------------------------------------------------------------AIQCVAARTRELDAQLQ  target    QSTRNEELIELMELEKTIVYFKASLKTNERVIKKLTSSTSNIKKYLEDEDLLEDTLIETQQAIEMADIYGNVLHSMTETF 3vpv.1    NDD----PQNAAELEQLLVGYDLAADDLKNAYEQALGQYSGL--------------------------------------  target    ASIISNNQNNIMKTLALVTIVMSIPTMVFSAYGMNFKDNEIPLNGEPNAFWLIVFIAFAMSVSLTLYLIHKKWF 3vpv.1    -------------------------------------------------------------------------- ``` | | | | | | | | | | | | | | | | | | | | | | | | | | | | | | | | | | | | | | | | | | | | | | | | | |
|  | 3vpv.1.A | Tse2 specific immunity protein 2  *Crystal Structure of Pseudomonas aeruginosa Tsi2* | 0.05 |  | 14.55 | 0.18 | 144-202 | X-ray | 1.80 | homo-dimer |  | HHblits | 0.31 |
| ``` target    MKQVFLSTTTEFKEIDTLEPGTWINLVNPTQNESLEIANTFDIDIADLRAPLDAEEMSRITIEDEYTLIIVDVPVTEERN 3vpv.1    --------------------------------------------------------------------------------  target    NRTYYVTIPLGIIITEETIITTCLEPLPVLDVFINRRLRNFYTFMRSRFIFQILYRNAELYLTALRSIDRKSEQIESQLH 3vpv.1    ---------------------------------------------------------------AIQCVAARTRELDAQLQ  target    QSTRNEELIELMELEKTIVYFKASLKTNERVIKKLTSSTSNIKKYLEDEDLLEDTLIETQQAIEMADIYGNVLHSMTETF 3vpv.1    NDD----PQNAAELEQLLVGYDLAADDLKNAYEQALGQYSGL--------------------------------------  target    ASIISNNQNNIMKTLALVTIVMSIPTMVFSAYGMNFKDNEIPLNGEPNAFWLIVFIAFAMSVSLTLYLIHKKWF 3vpv.1    -------------------------------------------------------------------------- ``` | | | | | | | | | | | | | | | | | | | | | | | | | | | | | | | | | | | | | | | | | | | | | | | | | |
|  | 2iak.1.A | Bullous pemphigoid antigen 1, isoform 5  *Crystal Structure of a protease resistant fragment of the plakin domain of Bullous Pemphigoid Antigen1 (BPAG1)* | 0.05 |  | 8.62 | 0.18 | 140-197 | X-ray | 3.00 | monomer |  | HHblits | 0.26 |
| ``` target    MKQVFLSTTTEFKEIDTLEPGTWINLVNPTQNESLEIANTFDIDIADLRAPLDAEEMSRITIEDEYTLIIVDVPVTEERN 2iak.1    --------------------------------------------------------------------------------  target    NRTYYVTIPLGIIITEETIITTCLEPLPVLDVFINRRLRNFYTFMRSRFIFQILYRNAELYLTALRSIDRKSEQIESQLH 2iak.1    -----------------------------------------------------------VNMKFVQDLLNWVDEMQVQLD  target    QSTRNEELIELMELEKTIVYFKASLKTNERVIKKLTSSTSNIKKYLEDEDLLEDTLIETQQAIEMADIYGNVLHSMTETF 2iak.1    RTEWGSDLPSVESHLENHKNVHRAIEEFESSLKEAKI-------------------------------------------  target    ASIISNNQNNIMKTLALVTIVMSIPTMVFSAYGMNFKDNEIPLNGEPNAFWLIVFIAFAMSVSLTLYLIHKKWF 2iak.1    -------------------------------------------------------------------------- ``` | | | | | | | | | | | | | | | | | | | | | | | | | | | | | | | | | | | | | | | | | | | | | | | | | |
|  | 6vja.1.A | B-lymphocyte antigen CD20  *Structure of CD20 in complex with rituximab Fab* | 0.04 |  | 21.05 | 0.18 | 250-307 | EM | 0.00 | hetero-2-2-2-mer | 6 x Y01 | HHblits | 0.27 |
| ``` target    MKQVFLSTTTEFKEIDTLEPGTWINLVNPTQNESLEIANTFDIDIADLRAPLDAEEMSRITIEDEYTLIIVDVPVTEERN 6vja.1    --------------------------------------------------------------------------------  target    NRTYYVTIPLGIIITEETIITTCLEPLPVLDVFINRRLRNFYTFMRSRFIFQILYRNAELYLTALRSIDRKSEQIESQLH 6vja.1    --------------------------------------------------------------------------------  target    QSTRNEELIELMELEKTIVYFKASLKTNERVIKKLTSSTSNIKKYLEDEDLLEDTLIETQQAIEMADIYGNVLHSMTETF 6vja.1    --------------------------------------------------------------------------------  target    ASIISNNQNNIMKTLALVTIVMSIPTMVFSAYGMNFKDNEIPLNGEPNAFWLIVFIAFAMSVSLTLYLIHKKWF 6vja.1    ---------RESKTLGAVQIMNGLFHIALGGLLMIPAGIYAPIC-VTVWYPLWGGIMYIISGSLLAA------- ``` | | | | | | | | | | | | | | | | | | | | | | | | | | | | | | | | | | | | | | | | | | | | | | | | | |
|  | 4xng.1.A | Uncharacterized protein MG218.1  *Central Domain of Mycoplasma Genitalium Terminal Organelle protein MG491* | 0.05 |  | 24.53 | 0.17 | 107-159 | X-ray | 3.00 | homo-tetramer |  | HHblits | 0.32 |
| ``` target    MKQVFLSTTTEFKEIDTLEPGTWINLVNPTQNESLEIANTFDIDIADLRAPLDAEEMSRITIEDEYTLIIVDVPVTEERN 4xng.1    --------------------------------------------------------------------------------  target    NRTYYVTIPLGIIITEETIITTCLEPLPVLDVFINRRLRNFYTFMRSRFIFQILYRNAELYLTALRSIDRKSEQIESQLH 4xng.1    --------------------------TPVLNSFFNKLLSDPDPMQREIGLRQFIITLRQRFKKLSQKIDSSLKQIETEA-  target    QSTRNEELIELMELEKTIVYFKASLKTNERVIKKLTSSTSNIKKYLEDEDLLEDTLIETQQAIEMADIYGNVLHSMTETF 4xng.1    --------------------------------------------------------------------------------  target    ASIISNNQNNIMKTLALVTIVMSIPTMVFSAYGMNFKDNEIPLNGEPNAFWLIVFIAFAMSVSLTLYLIHKKWF 4xng.1    -------------------------------------------------------------------------- ``` | | | | | | | | | | | | | | | | | | | | | | | | | | | | | | | | | | | | | | | | | | | | | | | | | |
|  | 4xng.1.B | Uncharacterized protein MG218.1  *Central Domain of Mycoplasma Genitalium Terminal Organelle protein MG491* | 0.05 |  | 24.53 | 0.17 | 107-159 | X-ray | 3.00 | homo-tetramer |  | HHblits | 0.32 |
| ``` target    MKQVFLSTTTEFKEIDTLEPGTWINLVNPTQNESLEIANTFDIDIADLRAPLDAEEMSRITIEDEYTLIIVDVPVTEERN 4xng.1    --------------------------------------------------------------------------------  target    NRTYYVTIPLGIIITEETIITTCLEPLPVLDVFINRRLRNFYTFMRSRFIFQILYRNAELYLTALRSIDRKSEQIESQLH 4xng.1    --------------------------TPVLNSFFNKLLSDPDPMQREIGLRQFIITLRQRFKKLSQKIDSSLKQIETEA-  target    QSTRNEELIELMELEKTIVYFKASLKTNERVIKKLTSSTSNIKKYLEDEDLLEDTLIETQQAIEMADIYGNVLHSMTETF 4xng.1    --------------------------------------------------------------------------------  target    ASIISNNQNNIMKTLALVTIVMSIPTMVFSAYGMNFKDNEIPLNGEPNAFWLIVFIAFAMSVSLTLYLIHKKWF 4xng.1    -------------------------------------------------------------------------- ``` | | | | | | | | | | | | | | | | | | | | | | | | | | | | | | | | | | | | | | | | | | | | | | | | | |
|  | 3jc8.42.A | Type 4 fimbrial assembly protein PilC  *Architectural model of the type IVa pilus machine in a piliated state* | 0.04 |  | 8.93 | 0.18 | 211-266 | EM | 0.00 | monomer |  | HHblits | 0.27 |
| ``` target    MKQVFLSTTTEFKEIDTLEPGTWINLVNPTQNESLEIANTFDIDIADLRAPLDAEEMSRITIEDEYTLIIVDVPVTEERN 3jc8.42   --------------------------------------------------------------------------------  target    NRTYYVTIPLGIIITEETIITTCLEPLPVLDVFINRRLRNFYTFMRSRFIFQILYRNAELYLTALRSIDRKSEQIESQLH 3jc8.42   --------------------------------------------------------------------------------  target    QSTRNEELIELMELEKTIVYFKASLKTNERVIKKLTSSTSNIKKYLEDEDLLEDTLIETQQAIEMADIYGNVLHSMTETF 3jc8.42   --------------------------------------------------VFPSMVVQMIGVGEATGAMDTMLNKIADFY  target    ASIISNNQNNIMKTLALVTIVMSIPTMVFSAYGMNFKDNEIPLNGEPNAFWLIVFIAFAMSVSLTLYLIHKKWF 3jc8.42   DDEVDAAINSLTAMIEPVLMVFLGGV------------------------------------------------ ``` | | | | | | | | | | | | | | | | | | | | | | | | | | | | | | | | | | | | | | | | | | | | | | | | | |
|  | 3jc8.43.A | Type 4 fimbrial assembly protein PilC  *Architectural model of the type IVa pilus machine in a piliated state* | 0.04 |  | 8.93 | 0.18 | 211-266 | EM | 0.00 | monomer |  | HHblits | 0.27 |
| ``` target    MKQVFLSTTTEFKEIDTLEPGTWINLVNPTQNESLEIANTFDIDIADLRAPLDAEEMSRITIEDEYTLIIVDVPVTEERN 3jc8.43   --------------------------------------------------------------------------------  target    NRTYYVTIPLGIIITEETIITTCLEPLPVLDVFINRRLRNFYTFMRSRFIFQILYRNAELYLTALRSIDRKSEQIESQLH 3jc8.43   --------------------------------------------------------------------------------  target    QSTRNEELIELMELEKTIVYFKASLKTNERVIKKLTSSTSNIKKYLEDEDLLEDTLIETQQAIEMADIYGNVLHSMTETF 3jc8.43   --------------------------------------------------VFPSMVVQMIGVGEATGAMDTMLNKIADFY  target    ASIISNNQNNIMKTLALVTIVMSIPTMVFSAYGMNFKDNEIPLNGEPNAFWLIVFIAFAMSVSLTLYLIHKKWF 3jc8.43   DDEVDAAINSLTAMIEPVLMVFLGGV------------------------------------------------ ``` | | | | | | | | | | | | | | | | | | | | | | | | | | | | | | | | | | | | | | | | | | | | | | | | | |
|  | 6nyk.1.A | Design construct XAX\_GGDQ  *Crystal structure of computationally designed protein XAX\_GGDQ* | 0.06 |  | 16.98 | 0.17 | 182-238 | X-ray | 2.80 | homo-trimer |  | HHblits | 0.30 |
| ``` target    MKQVFLSTTTEFKEIDTLEPGTWINLVNPTQNESLEIANTFDIDIADLRAPLDAEEMSRITIEDEYTLIIVDVPVTEERN 6nyk.1    --------------------------------------------------------------------------------  target    NRTYYVTIPLGIIITEETIITTCLEPLPVLDVFINRRLRNFYTFMRSRFIFQILYRNAELYLTALRSIDRKSEQIESQLH 6nyk.1    --------------------------------------------------------------------------------  target    QSTRNEELIELMELEKTIVYFKASLKTNERVIKKLTSSTSNIKKYLEDEDLLEDTLIETQQAIEMADIYGNVLHSMTETF 6nyk.1    ---------------------KYSLERLREILERLEENPSE----KQIVEAIRAIVENNAQIVEAIRAIVEILAQIVE--  target    ASIISNNQNNIMKTLALVTIVMSIPTMVFSAYGMNFKDNEIPLNGEPNAFWLIVFIAFAMSVSLTLYLIHKKWF 6nyk.1    -------------------------------------------------------------------------- ``` | | | | | | | | | | | | | | | | | | | | | | | | | | | | | | | | | | | | | | | | | | | | | | | | | |
|  | 3stq.3.A | Putative uncharacterized protein  *Hypothetical protein PA2703 Pseudomonas aeruginosa PAO1* | 0.05 |  | 13.21 | 0.17 | 145-201 | X-ray | 2.28 | homo-dimer |  | HHblits | 0.30 |
| ``` target    MKQVFLSTTTEFKEIDTLEPGTWINLVNPTQNESLEIANTFDIDIADLRAPLDAEEMSRITIEDEYTLIIVDVPVTEERN 3stq.3    --------------------------------------------------------------------------------  target    NRTYYVTIPLGIIITEETIITTCLEPLPVLDVFINRRLRNFYTFMRSRFIFQILYRNAELYLTALRSIDRKSEQIESQLH 3stq.3    ----------------------------------------------------------------IQCVAARTRELDAQLQ  target    QSTRNEELIELMELEKTIVYFKASLKTNERVIKKLTSSTSNIKKYLEDEDLLEDTLIETQQAIEMADIYGNVLHSMTETF 3stq.3    NDD----PQNAAELEQLLVGYDLAADDLKNAYEQALGQYSG---------------------------------------  target    ASIISNNQNNIMKTLALVTIVMSIPTMVFSAYGMNFKDNEIPLNGEPNAFWLIVFIAFAMSVSLTLYLIHKKWF 3stq.3    -------------------------------------------------------------------------- ``` | | | | | | | | | | | | | | | | | | | | | | | | | | | | | | | | | | | | | | | | | | | | | | | | | |
|  | 3stq.1.B | Putative uncharacterized protein  *Hypothetical protein PA2703 Pseudomonas aeruginosa PAO1* | 0.06 |  | 13.21 | 0.17 | 145-201 | X-ray | 2.28 | homo-dimer |  | HHblits | 0.30 |
| ``` target    MKQVFLSTTTEFKEIDTLEPGTWINLVNPTQNESLEIANTFDIDIADLRAPLDAEEMSRITIEDEYTLIIVDVPVTEERN 3stq.1    --------------------------------------------------------------------------------  target    NRTYYVTIPLGIIITEETIITTCLEPLPVLDVFINRRLRNFYTFMRSRFIFQILYRNAELYLTALRSIDRKSEQIESQLH 3stq.1    ----------------------------------------------------------------IQCVAARTRELDAQLQ  target    QSTRNEELIELMELEKTIVYFKASLKTNERVIKKLTSSTSNIKKYLEDEDLLEDTLIETQQAIEMADIYGNVLHSMTETF 3stq.1    NDD----PQNAAELEQLLVGYDLAADDLKNAYEQALGQYSG---------------------------------------  target    ASIISNNQNNIMKTLALVTIVMSIPTMVFSAYGMNFKDNEIPLNGEPNAFWLIVFIAFAMSVSLTLYLIHKKWF 3stq.1    -------------------------------------------------------------------------- ``` | | | | | | | | | | | | | | | | | | | | | | | | | | | | | | | | | | | | | | | | | | | | | | | | | |
|  | 3stq.2.A | Putative uncharacterized protein  *Hypothetical protein PA2703 Pseudomonas aeruginosa PAO1* | 0.06 |  | 13.21 | 0.17 | 145-201 | X-ray | 2.28 | homo-dimer |  | HHblits | 0.30 |
| ``` target    MKQVFLSTTTEFKEIDTLEPGTWINLVNPTQNESLEIANTFDIDIADLRAPLDAEEMSRITIEDEYTLIIVDVPVTEERN 3stq.2    --------------------------------------------------------------------------------  target    NRTYYVTIPLGIIITEETIITTCLEPLPVLDVFINRRLRNFYTFMRSRFIFQILYRNAELYLTALRSIDRKSEQIESQLH 3stq.2    ----------------------------------------------------------------IQCVAARTRELDAQLQ  target    QSTRNEELIELMELEKTIVYFKASLKTNERVIKKLTSSTSNIKKYLEDEDLLEDTLIETQQAIEMADIYGNVLHSMTETF 3stq.2    NDD----PQNAAELEQLLVGYDLAADDLKNAYEQALGQYSG---------------------------------------  target    ASIISNNQNNIMKTLALVTIVMSIPTMVFSAYGMNFKDNEIPLNGEPNAFWLIVFIAFAMSVSLTLYLIHKKWF 3stq.2    -------------------------------------------------------------------------- ``` | | | | | | | | | | | | | | | | | | | | | | | | | | | | | | | | | | | | | | | | | | | | | | | | | |
|  | 3stq.2.B | Putative uncharacterized protein  *Hypothetical protein PA2703 Pseudomonas aeruginosa PAO1* | 0.06 |  | 13.21 | 0.17 | 145-201 | X-ray | 2.28 | homo-dimer |  | HHblits | 0.30 |
| ``` target    MKQVFLSTTTEFKEIDTLEPGTWINLVNPTQNESLEIANTFDIDIADLRAPLDAEEMSRITIEDEYTLIIVDVPVTEERN 3stq.2    --------------------------------------------------------------------------------  target    NRTYYVTIPLGIIITEETIITTCLEPLPVLDVFINRRLRNFYTFMRSRFIFQILYRNAELYLTALRSIDRKSEQIESQLH 3stq.2    ----------------------------------------------------------------IQCVAARTRELDAQLQ  target    QSTRNEELIELMELEKTIVYFKASLKTNERVIKKLTSSTSNIKKYLEDEDLLEDTLIETQQAIEMADIYGNVLHSMTETF 3stq.2    NDD----PQNAAELEQLLVGYDLAADDLKNAYEQALGQYSG---------------------------------------  target    ASIISNNQNNIMKTLALVTIVMSIPTMVFSAYGMNFKDNEIPLNGEPNAFWLIVFIAFAMSVSLTLYLIHKKWF 3stq.2    -------------------------------------------------------------------------- ``` | | | | | | | | | | | | | | | | | | | | | | | | | | | | | | | | | | | | | | | | | | | | | | | | | |
|  | 3stq.1.A | Putative uncharacterized protein  *Hypothetical protein PA2703 Pseudomonas aeruginosa PAO1* | 0.06 |  | 13.21 | 0.17 | 145-201 | X-ray | 2.28 | homo-dimer |  | HHblits | 0.30 |
| ``` target    MKQVFLSTTTEFKEIDTLEPGTWINLVNPTQNESLEIANTFDIDIADLRAPLDAEEMSRITIEDEYTLIIVDVPVTEERN 3stq.1    --------------------------------------------------------------------------------  target    NRTYYVTIPLGIIITEETIITTCLEPLPVLDVFINRRLRNFYTFMRSRFIFQILYRNAELYLTALRSIDRKSEQIESQLH 3stq.1    ----------------------------------------------------------------IQCVAARTRELDAQLQ  target    QSTRNEELIELMELEKTIVYFKASLKTNERVIKKLTSSTSNIKKYLEDEDLLEDTLIETQQAIEMADIYGNVLHSMTETF 3stq.1    NDD----PQNAAELEQLLVGYDLAADDLKNAYEQALGQYSG---------------------------------------  target    ASIISNNQNNIMKTLALVTIVMSIPTMVFSAYGMNFKDNEIPLNGEPNAFWLIVFIAFAMSVSLTLYLIHKKWF 3stq.1    -------------------------------------------------------------------------- ``` | | | | | | | | | | | | | | | | | | | | | | | | | | | | | | | | | | | | | | | | | | | | | | | | | |
|  | 3stq.4.A | Putative uncharacterized protein  *Hypothetical protein PA2703 Pseudomonas aeruginosa PAO1* | 0.05 |  | 13.21 | 0.17 | 145-201 | X-ray | 2.28 | homo-dimer |  | HHblits | 0.30 |
| ``` target    MKQVFLSTTTEFKEIDTLEPGTWINLVNPTQNESLEIANTFDIDIADLRAPLDAEEMSRITIEDEYTLIIVDVPVTEERN 3stq.4    --------------------------------------------------------------------------------  target    NRTYYVTIPLGIIITEETIITTCLEPLPVLDVFINRRLRNFYTFMRSRFIFQILYRNAELYLTALRSIDRKSEQIESQLH 3stq.4    ----------------------------------------------------------------IQCVAARTRELDAQLQ  target    QSTRNEELIELMELEKTIVYFKASLKTNERVIKKLTSSTSNIKKYLEDEDLLEDTLIETQQAIEMADIYGNVLHSMTETF 3stq.4    NDD----PQNAAELEQLLVGYDLAADDLKNAYEQALGQYSG---------------------------------------  target    ASIISNNQNNIMKTLALVTIVMSIPTMVFSAYGMNFKDNEIPLNGEPNAFWLIVFIAFAMSVSLTLYLIHKKWF 3stq.4    -------------------------------------------------------------------------- ``` | | | | | | | | | | | | | | | | | | | | | | | | | | | | | | | | | | | | | | | | | | | | | | | | | |
|  | 6y97.1.A | B-lymphocyte antigen CD20  *Structure of full-length CD20 in complex with Obinutuzumab Fab* | 0.04 |  | 22.64 | 0.17 | 253-306 | EM | 0.00 | hetero-2-1-1-mer |  | HHblits | 0.27 |
| ``` target    MKQVFLSTTTEFKEIDTLEPGTWINLVNPTQNESLEIANTFDIDIADLRAPLDAEEMSRITIEDEYTLIIVDVPVTEERN 6y97.1    --------------------------------------------------------------------------------  target    NRTYYVTIPLGIIITEETIITTCLEPLPVLDVFINRRLRNFYTFMRSRFIFQILYRNAELYLTALRSIDRKSEQIESQLH 6y97.1    --------------------------------------------------------------------------------  target    QSTRNEELIELMELEKTIVYFKASLKTNERVIKKLTSSTSNIKKYLEDEDLLEDTLIETQQAIEMADIYGNVLHSMTETF 6y97.1    --------------------------------------------------------------------------------  target    ASIISNNQNNIMKTLALVTIVMSIPTMVFSAYGMNFKDNEIPLNGEPNAFWLIVFIAFAMSVSLTLYLIHKKWF 6y97.1    ------------KTLGAVQIMNGLFHIALGGLLMIPAGIYAPIC-VTVWYPLWGGIMYIISGSLLA-------- ``` | | | | | | | | | | | | | | | | | | | | | | | | | | | | | | | | | | | | | | | | | | | | | | | | | |
|  | 6y97.1.B | B-lymphocyte antigen CD20  *Structure of full-length CD20 in complex with Obinutuzumab Fab* | 0.04 |  | 22.64 | 0.17 | 253-306 | EM | 0.00 | hetero-2-1-1-mer |  | HHblits | 0.27 |
| ``` target    MKQVFLSTTTEFKEIDTLEPGTWINLVNPTQNESLEIANTFDIDIADLRAPLDAEEMSRITIEDEYTLIIVDVPVTEERN 6y97.1    --------------------------------------------------------------------------------  target    NRTYYVTIPLGIIITEETIITTCLEPLPVLDVFINRRLRNFYTFMRSRFIFQILYRNAELYLTALRSIDRKSEQIESQLH 6y97.1    --------------------------------------------------------------------------------  target    QSTRNEELIELMELEKTIVYFKASLKTNERVIKKLTSSTSNIKKYLEDEDLLEDTLIETQQAIEMADIYGNVLHSMTETF 6y97.1    --------------------------------------------------------------------------------  target    ASIISNNQNNIMKTLALVTIVMSIPTMVFSAYGMNFKDNEIPLNGEPNAFWLIVFIAFAMSVSLTLYLIHKKWF 6y97.1    ------------KTLGAVQIMNGLFHIALGGLLMIPAGIYAPIC-VTVWYPLWGGIMYIISGSLLA-------- ``` | | | | | | | | | | | | | | | | | | | | | | | | | | | | | | | | | | | | | | | | | | | | | | | | | |
|  | 6y9a.1.A | B-lymphocyte antigen CD20  *Structure of full-length CD20 in complex with Obinutuzumab Fab* | 0.04 |  | 22.64 | 0.17 | 253-306 | EM | 0.00 | hetero-2-1-1-mer |  | HHblits | 0.27 |
| ``` target    MKQVFLSTTTEFKEIDTLEPGTWINLVNPTQNESLEIANTFDIDIADLRAPLDAEEMSRITIEDEYTLIIVDVPVTEERN 6y9a.1    --------------------------------------------------------------------------------  target    NRTYYVTIPLGIIITEETIITTCLEPLPVLDVFINRRLRNFYTFMRSRFIFQILYRNAELYLTALRSIDRKSEQIESQLH 6y9a.1    --------------------------------------------------------------------------------  target    QSTRNEELIELMELEKTIVYFKASLKTNERVIKKLTSSTSNIKKYLEDEDLLEDTLIETQQAIEMADIYGNVLHSMTETF 6y9a.1    --------------------------------------------------------------------------------  target    ASIISNNQNNIMKTLALVTIVMSIPTMVFSAYGMNFKDNEIPLNGEPNAFWLIVFIAFAMSVSLTLYLIHKKWF 6y9a.1    ------------KTLGAVQIMNGLFHIALGGLLMIPAGIYAPIC-VTVWYPLWGGIMYIISGSLLA-------- ``` | | | | | | | | | | | | | | | | | | | | | | | | | | | | | | | | | | | | | | | | | | | | | | | | | |
|  | 6y9a.1.B | B-lymphocyte antigen CD20  *Structure of full-length CD20 in complex with Obinutuzumab Fab* | 0.03 |  | 22.64 | 0.17 | 253-306 | EM | 0.00 | hetero-2-1-1-mer |  | HHblits | 0.27 |
| ``` target    MKQVFLSTTTEFKEIDTLEPGTWINLVNPTQNESLEIANTFDIDIADLRAPLDAEEMSRITIEDEYTLIIVDVPVTEERN 6y9a.1    --------------------------------------------------------------------------------  target    NRTYYVTIPLGIIITEETIITTCLEPLPVLDVFINRRLRNFYTFMRSRFIFQILYRNAELYLTALRSIDRKSEQIESQLH 6y9a.1    --------------------------------------------------------------------------------  target    QSTRNEELIELMELEKTIVYFKASLKTNERVIKKLTSSTSNIKKYLEDEDLLEDTLIETQQAIEMADIYGNVLHSMTETF 6y9a.1    --------------------------------------------------------------------------------  target    ASIISNNQNNIMKTLALVTIVMSIPTMVFSAYGMNFKDNEIPLNGEPNAFWLIVFIAFAMSVSLTLYLIHKKWF 6y9a.1    ------------KTLGAVQIMNGLFHIALGGLLMIPAGIYAPIC-VTVWYPLWGGIMYIISGSLLA-------- ``` | | | | | | | | | | | | | | | | | | | | | | | | | | | | | | | | | | | | | | | | | | | | | | | | | |
|  | 5kuc.1.A | Pesticidal crystal protein Cry6Aa  *Crystal structure of trypsin activated Cry6Aa* | 0.06 |  | 17.31 | 0.17 | 146-197 | X-ray | 2.00 | monomer |  | HHblits | 0.28 |
| ``` target    MKQVFLSTTTEFKEIDTLEPGTWINLVNPTQNESLEIANTFDIDIADLRAPLDAEEMSRITIEDEYTLIIVDVPVTEERN 5kuc.1    --------------------------------------------------------------------------------  target    NRTYYVTIPLGIIITEETIITTCLEPLPVLDVFINRRLRNFYTFMRSRFIFQILYRNAELYLTALRSIDRKSEQIESQLH 5kuc.1    -----------------------------------------------------------------QHIKNQIDEIKKQLD  target    QST-----RNEELIELMELEKTIVYFKASLKTNERVIKKLTSSTSNIKKYLEDEDLLEDTLIETQQAIEMADIYGNVLHS 5kuc.1    SAQHDLDRDVKIIGMLNSINTDIDNLYSQGQEAIKVFQKLQG--------------------------------------  target    MTETFASIISNNQNNIMKTLALVTIVMSIPTMVFSAYGMNFKDNEIPLNGEPNAFWLIVFIAFAMSVSLTLYLIHKKWF 5kuc.1    ------------------------------------------------------------------------------- ``` | | | | | | | | | | | | | | | | | | | | | | | | | | | | | | | | | | | | | | | | | | | | | | | | | |
|  | 3okq.1.A | Bud site selection protein 6  *Crystal structure of a core domain of yeast actin nucleation cofactor Bud6* | 0.05 |  | 9.43 | 0.17 | 138-190 | X-ray | 2.04 | homo-dimer |  | HHblits | 0.27 |
| ``` target    MKQVFLSTTTEFKEIDTLEPGTWINLVNPTQNESLEIANTFDIDIADLRAPLDAEEMSRITIEDEYTLIIVDVPVTEERN 3okq.1    --------------------------------------------------------------------------------  target    NRTYYVTIPLGIIITEETIITTCLEPLPVLDVFINRRLRNFYTFMRSRFIFQILYRNAELYLTALRSIDRKSEQIESQLH 3okq.1    ---------------------------------------------------------SDTLLSKVDDLQDVIEIMRKDVA  target    QSTRNEELIELMELEKTIVYFKASLKTNERVIKKLTSSTSNIKKYLEDEDLLEDTLIETQQAIEMADIYGNVLHSMTETF 3okq.1    ERRSQPAKKKLETVSKDLENAQADVLKLQE--------------------------------------------------  target    ASIISNNQNNIMKTLALVTIVMSIPTMVFSAYGMNFKDNEIPLNGEPNAFWLIVFIAFAMSVSLTLYLIHKKWF 3okq.1    -------------------------------------------------------------------------- ``` | | | | | | | | | | | | | | | | | | | | | | | | | | | | | | | | | | | | | | | | | | | | | | | | | |
|  | 3onx.1.A | Bud site selection protein 6  *Crystal structure of a domain of a protein involved in formation of actin cytoskeleton* | 0.05 |  | 9.43 | 0.17 | 138-190 | X-ray | 2.90 | homo-dimer |  | HHblits | 0.27 |
| ``` target    MKQVFLSTTTEFKEIDTLEPGTWINLVNPTQNESLEIANTFDIDIADLRAPLDAEEMSRITIEDEYTLIIVDVPVTEERN 3onx.1    --------------------------------------------------------------------------------  target    NRTYYVTIPLGIIITEETIITTCLEPLPVLDVFINRRLRNFYTFMRSRFIFQILYRNAELYLTALRSIDRKSEQIESQLH 3onx.1    ---------------------------------------------------------SDTLLSKVDDLQDVIEIMRKDVA  target    QSTRNEELIELMELEKTIVYFKASLKTNERVIKKLTSSTSNIKKYLEDEDLLEDTLIETQQAIEMADIYGNVLHSMTETF 3onx.1    ERRSQPAKKKLETVSKDLENAQADVLKLQE--------------------------------------------------  target    ASIISNNQNNIMKTLALVTIVMSIPTMVFSAYGMNFKDNEIPLNGEPNAFWLIVFIAFAMSVSLTLYLIHKKWF 3onx.1    -------------------------------------------------------------------------- ``` | | | | | | | | | | | | | | | | | | | | | | | | | | | | | | | | | | | | | | | | | | | | | | | | | |
|  | 3onx.1.B | Bud site selection protein 6  *Crystal structure of a domain of a protein involved in formation of actin cytoskeleton* | 0.05 |  | 9.43 | 0.17 | 138-190 | X-ray | 2.90 | homo-dimer |  | HHblits | 0.27 |
| ``` target    MKQVFLSTTTEFKEIDTLEPGTWINLVNPTQNESLEIANTFDIDIADLRAPLDAEEMSRITIEDEYTLIIVDVPVTEERN 3onx.1    --------------------------------------------------------------------------------  target    NRTYYVTIPLGIIITEETIITTCLEPLPVLDVFINRRLRNFYTFMRSRFIFQILYRNAELYLTALRSIDRKSEQIESQLH 3onx.1    ---------------------------------------------------------SDTLLSKVDDLQDVIEIMRKDVA  target    QSTRNEELIELMELEKTIVYFKASLKTNERVIKKLTSSTSNIKKYLEDEDLLEDTLIETQQAIEMADIYGNVLHSMTETF 3onx.1    ERRSQPAKKKLETVSKDLENAQADVLKLQE--------------------------------------------------  target    ASIISNNQNNIMKTLALVTIVMSIPTMVFSAYGMNFKDNEIPLNGEPNAFWLIVFIAFAMSVSLTLYLIHKKWF 3onx.1    -------------------------------------------------------------------------- ``` | | | | | | | | | | | | | | | | | | | | | | | | | | | | | | | | | | | | | | | | | | | | | | | | | |
|  | 4eij.1.A | P protein  *Structure of the Mumps virus phosphoprotein oligomerization domain* | 0.05 |  | 18.00 | 0.16 | 138-189 | X-ray | 2.20 | homo-tetramer |  | HHblits | 0.31 |
| ``` target    MKQVFLSTTTEFKEIDTLEPGTWINLVNPTQNESLEIANTFDIDIADLRAPLDAEEMSRITIEDEYTLIIVDVPVTEERN 4eij.1    --------------------------------------------------------------------------------  target    NRTYYVTIPLGIIITEETIITTCLEPLPVLDVFINRRLRNFYTFMRSRFIFQILYRNAELYLTALRSIDRKSEQIESQLH 4eij.1    ---------------------------------------------------------ANEIMDLLRGMDARLQHLEQKVD  target    QSTRNEELIELMELEKTIVYFKASLKTNERVIKKLTSSTSNIKKYLEDEDLLEDTLIETQQAIEMADIYGNVLHSMTETF 4eij.1    KVL--AQGSMVTQIKNELSTVKTTLATIE---------------------------------------------------  target    ASIISNNQNNIMKTLALVTIVMSIPTMVFSAYGMNFKDNEIPLNGEPNAFWLIVFIAFAMSVSLTLYLIHKKWF 4eij.1    -------------------------------------------------------------------------- ``` | | | | | | | | | | | | | | | | | | | | | | | | | | | | | | | | | | | | | | | | | | | | | | | | | |
|  | 4eij.1.B | P protein  *Structure of the Mumps virus phosphoprotein oligomerization domain* | 0.05 |  | 18.00 | 0.16 | 138-189 | X-ray | 2.20 | homo-tetramer |  | HHblits | 0.31 |
| ``` target    MKQVFLSTTTEFKEIDTLEPGTWINLVNPTQNESLEIANTFDIDIADLRAPLDAEEMSRITIEDEYTLIIVDVPVTEERN 4eij.1    --------------------------------------------------------------------------------  target    NRTYYVTIPLGIIITEETIITTCLEPLPVLDVFINRRLRNFYTFMRSRFIFQILYRNAELYLTALRSIDRKSEQIESQLH 4eij.1    ---------------------------------------------------------ANEIMDLLRGMDARLQHLEQKVD  target    QSTRNEELIELMELEKTIVYFKASLKTNERVIKKLTSSTSNIKKYLEDEDLLEDTLIETQQAIEMADIYGNVLHSMTETF 4eij.1    KVL--AQGSMVTQIKNELSTVKTTLATIE---------------------------------------------------  target    ASIISNNQNNIMKTLALVTIVMSIPTMVFSAYGMNFKDNEIPLNGEPNAFWLIVFIAFAMSVSLTLYLIHKKWF 4eij.1    -------------------------------------------------------------------------- ``` | | | | | | | | | | | | | | | | | | | | | | | | | | | | | | | | | | | | | | | | | | | | | | | | | |
|  | 2xdj.1.A | UNCHARACTERIZED PROTEIN YBGF  *CRYSTAL STRUCTURE OF THE N-TERMINAL DOMAIN OF E.COLI YBGF* | 0.04 |  | 12.00 | 0.16 | 147-196 | X-ray | 1.82 | homo-trimer |  | HHblits | 0.29 |
| ``` target    MKQVFLSTTTEFKEIDTLEPGTWINLVNPTQNESLEIANTFDIDIADLRAPLDAEEMSRITIEDEYTLIIVDVPVTEERN 2xdj.1    --------------------------------------------------------------------------------  target    NRTYYVTIPLGIIITEETIITTCLEPLPVLDVFINRRLRNFYTFMRSRFIFQILYRNAELYLTALRSIDRKSEQIESQLH 2xdj.1    ------------------------------------------------------------------SVEDRVTQLERISN  target    QS--TRNEELIELMELEKTIVYFKASLKTNERVIKKLTSSTSNIKKYLEDEDLLEDTLIETQQAIEMADIYGNVLHSMTE 2xdj.1    AHSQLLTQLQQQLSDNQSDIDSLRGQIQENQYQLNQVV------------------------------------------  target    TFASIISNNQNNIMKTLALVTIVMSIPTMVFSAYGMNFKDNEIPLNGEPNAFWLIVFIAFAMSVSLTLYLIHKKWF 2xdj.1    ---------------------------------------------------------------------------- ``` | | | | | | | | | | | | | | | | | | | | | | | | | | | | | | | | | | | | | | | | | | | | | | | | | |
|  | 2xdj.1.B | UNCHARACTERIZED PROTEIN YBGF  *CRYSTAL STRUCTURE OF THE N-TERMINAL DOMAIN OF E.COLI YBGF* | 0.04 |  | 12.00 | 0.16 | 147-196 | X-ray | 1.82 | homo-trimer |  | HHblits | 0.29 |
| ``` target    MKQVFLSTTTEFKEIDTLEPGTWINLVNPTQNESLEIANTFDIDIADLRAPLDAEEMSRITIEDEYTLIIVDVPVTEERN 2xdj.1    --------------------------------------------------------------------------------  target    NRTYYVTIPLGIIITEETIITTCLEPLPVLDVFINRRLRNFYTFMRSRFIFQILYRNAELYLTALRSIDRKSEQIESQLH 2xdj.1    ------------------------------------------------------------------SVEDRVTQLERISN  target    QS--TRNEELIELMELEKTIVYFKASLKTNERVIKKLTSSTSNIKKYLEDEDLLEDTLIETQQAIEMADIYGNVLHSMTE 2xdj.1    AHSQLLTQLQQQLSDNQSDIDSLRGQIQENQYQLNQVV------------------------------------------  target    TFASIISNNQNNIMKTLALVTIVMSIPTMVFSAYGMNFKDNEIPLNGEPNAFWLIVFIAFAMSVSLTLYLIHKKWF 2xdj.1    ---------------------------------------------------------------------------- ``` | | | | | | | | | | | | | | | | | | | | | | | | | | | | | | | | | | | | | | | | | | | | | | | | | |
|  | 2xdj.1.C | UNCHARACTERIZED PROTEIN YBGF  *CRYSTAL STRUCTURE OF THE N-TERMINAL DOMAIN OF E.COLI YBGF* | 0.04 |  | 12.00 | 0.16 | 147-196 | X-ray | 1.82 | homo-trimer |  | HHblits | 0.29 |
| ``` target    MKQVFLSTTTEFKEIDTLEPGTWINLVNPTQNESLEIANTFDIDIADLRAPLDAEEMSRITIEDEYTLIIVDVPVTEERN 2xdj.1    --------------------------------------------------------------------------------  target    NRTYYVTIPLGIIITEETIITTCLEPLPVLDVFINRRLRNFYTFMRSRFIFQILYRNAELYLTALRSIDRKSEQIESQLH 2xdj.1    ------------------------------------------------------------------SVEDRVTQLERISN  target    QS--TRNEELIELMELEKTIVYFKASLKTNERVIKKLTSSTSNIKKYLEDEDLLEDTLIETQQAIEMADIYGNVLHSMTE 2xdj.1    AHSQLLTQLQQQLSDNQSDIDSLRGQIQENQYQLNQVV------------------------------------------  target    TFASIISNNQNNIMKTLALVTIVMSIPTMVFSAYGMNFKDNEIPLNGEPNAFWLIVFIAFAMSVSLTLYLIHKKWF 2xdj.1    ---------------------------------------------------------------------------- ``` | | | | | | | | | | | | | | | | | | | | | | | | | | | | | | | | | | | | | | | | | | | | | | | | | |
|  | 2xdj.2.A | UNCHARACTERIZED PROTEIN YBGF  *CRYSTAL STRUCTURE OF THE N-TERMINAL DOMAIN OF E.COLI YBGF* | 0.04 |  | 12.00 | 0.16 | 147-196 | X-ray | 1.82 | homo-trimer |  | HHblits | 0.29 |
| ``` target    MKQVFLSTTTEFKEIDTLEPGTWINLVNPTQNESLEIANTFDIDIADLRAPLDAEEMSRITIEDEYTLIIVDVPVTEERN 2xdj.2    --------------------------------------------------------------------------------  target    NRTYYVTIPLGIIITEETIITTCLEPLPVLDVFINRRLRNFYTFMRSRFIFQILYRNAELYLTALRSIDRKSEQIESQLH 2xdj.2    ------------------------------------------------------------------SVEDRVTQLERISN  target    QS--TRNEELIELMELEKTIVYFKASLKTNERVIKKLTSSTSNIKKYLEDEDLLEDTLIETQQAIEMADIYGNVLHSMTE 2xdj.2    AHSQLLTQLQQQLSDNQSDIDSLRGQIQENQYQLNQVV------------------------------------------  target    TFASIISNNQNNIMKTLALVTIVMSIPTMVFSAYGMNFKDNEIPLNGEPNAFWLIVFIAFAMSVSLTLYLIHKKWF 2xdj.2    ---------------------------------------------------------------------------- ``` | | | | | | | | | | | | | | | | | | | | | | | | | | | | | | | | | | | | | | | | | | | | | | | | | |
|  | 2xdj.2.B | UNCHARACTERIZED PROTEIN YBGF  *CRYSTAL STRUCTURE OF THE N-TERMINAL DOMAIN OF E.COLI YBGF* | 0.04 |  | 12.00 | 0.16 | 147-196 | X-ray | 1.82 | homo-trimer |  | HHblits | 0.29 |
| ``` target    MKQVFLSTTTEFKEIDTLEPGTWINLVNPTQNESLEIANTFDIDIADLRAPLDAEEMSRITIEDEYTLIIVDVPVTEERN 2xdj.2    --------------------------------------------------------------------------------  target    NRTYYVTIPLGIIITEETIITTCLEPLPVLDVFINRRLRNFYTFMRSRFIFQILYRNAELYLTALRSIDRKSEQIESQLH 2xdj.2    ------------------------------------------------------------------SVEDRVTQLERISN  target    QS--TRNEELIELMELEKTIVYFKASLKTNERVIKKLTSSTSNIKKYLEDEDLLEDTLIETQQAIEMADIYGNVLHSMTE 2xdj.2    AHSQLLTQLQQQLSDNQSDIDSLRGQIQENQYQLNQVV------------------------------------------  target    TFASIISNNQNNIMKTLALVTIVMSIPTMVFSAYGMNFKDNEIPLNGEPNAFWLIVFIAFAMSVSLTLYLIHKKWF 2xdj.2    ---------------------------------------------------------------------------- ``` | | | | | | | | | | | | | | | | | | | | | | | | | | | | | | | | | | | | | | | | | | | | | | | | | |
|  | 2xdj.2.C | UNCHARACTERIZED PROTEIN YBGF  *CRYSTAL STRUCTURE OF THE N-TERMINAL DOMAIN OF E.COLI YBGF* | 0.04 |  | 12.00 | 0.16 | 147-196 | X-ray | 1.82 | homo-trimer |  | HHblits | 0.29 |
| ``` target    MKQVFLSTTTEFKEIDTLEPGTWINLVNPTQNESLEIANTFDIDIADLRAPLDAEEMSRITIEDEYTLIIVDVPVTEERN 2xdj.2    --------------------------------------------------------------------------------  target    NRTYYVTIPLGIIITEETIITTCLEPLPVLDVFINRRLRNFYTFMRSRFIFQILYRNAELYLTALRSIDRKSEQIESQLH 2xdj.2    ------------------------------------------------------------------SVEDRVTQLERISN  target    QS--TRNEELIELMELEKTIVYFKASLKTNERVIKKLTSSTSNIKKYLEDEDLLEDTLIETQQAIEMADIYGNVLHSMTE 2xdj.2    AHSQLLTQLQQQLSDNQSDIDSLRGQIQENQYQLNQVV------------------------------------------  target    TFASIISNNQNNIMKTLALVTIVMSIPTMVFSAYGMNFKDNEIPLNGEPNAFWLIVFIAFAMSVSLTLYLIHKKWF 2xdj.2    ---------------------------------------------------------------------------- ``` | | | | | | | | | | | | | | | | | | | | | | | | | | | | | | | | | | | | | | | | | | | | | | | | | |
|  | 2wz7.1.A | UNCHARACTERIZED PROTEIN YBGF  *CRYSTAL STRUCTURE OF THE N-TERMINAL DOMAIN OF E.COLI YBGF* | 0.04 |  | 12.24 | 0.16 | 147-195 | X-ray | 2.48 | homo-trimer | 1 x AUC | HHblits | 0.29 |
| ``` target    MKQVFLSTTTEFKEIDTLEPGTWINLVNPTQNESLEIANTFDIDIADLRAPLDAEEMSRITIEDEYTLIIVDVPVTEERN 2wz7.1    --------------------------------------------------------------------------------  target    NRTYYVTIPLGIIITEETIITTCLEPLPVLDVFINRRLRNFYTFMRSRFIFQILYRNAELYLTALRSIDRKSEQIESQLH 2wz7.1    ------------------------------------------------------------------SVEDRVTQLERISN  target    QS--TRNEELIELMELEKTIVYFKASLKTNERVIKKLTSSTSNIKKYLEDEDLLEDTLIETQQAIEMADIYGNVLHSMTE 2wz7.1    AHSQLLTQLQQQLSDNQSDIDSLRGQIQENQYQLNQV-------------------------------------------  target    TFASIISNNQNNIMKTLALVTIVMSIPTMVFSAYGMNFKDNEIPLNGEPNAFWLIVFIAFAMSVSLTLYLIHKKWF 2wz7.1    ---------------------------------------------------------------------------- ``` | | | | | | | | | | | | | | | | | | | | | | | | | | | | | | | | | | | | | | | | | | | | | | | | | |
|  | 2wz7.1.B | UNCHARACTERIZED PROTEIN YBGF  *CRYSTAL STRUCTURE OF THE N-TERMINAL DOMAIN OF E.COLI YBGF* | 0.04 |  | 12.24 | 0.16 | 147-195 | X-ray | 2.48 | homo-trimer | 1 x AUC | HHblits | 0.29 |
| ``` target    MKQVFLSTTTEFKEIDTLEPGTWINLVNPTQNESLEIANTFDIDIADLRAPLDAEEMSRITIEDEYTLIIVDVPVTEERN 2wz7.1    --------------------------------------------------------------------------------  target    NRTYYVTIPLGIIITEETIITTCLEPLPVLDVFINRRLRNFYTFMRSRFIFQILYRNAELYLTALRSIDRKSEQIESQLH 2wz7.1    ------------------------------------------------------------------SVEDRVTQLERISN  target    QS--TRNEELIELMELEKTIVYFKASLKTNERVIKKLTSSTSNIKKYLEDEDLLEDTLIETQQAIEMADIYGNVLHSMTE 2wz7.1    AHSQLLTQLQQQLSDNQSDIDSLRGQIQENQYQLNQV-------------------------------------------  target    TFASIISNNQNNIMKTLALVTIVMSIPTMVFSAYGMNFKDNEIPLNGEPNAFWLIVFIAFAMSVSLTLYLIHKKWF 2wz7.1    ---------------------------------------------------------------------------- ``` | | | | | | | | | | | | | | | | | | | | | | | | | | | | | | | | | | | | | | | | | | | | | | | | | |
|  | 2wz7.1.C | UNCHARACTERIZED PROTEIN YBGF  *CRYSTAL STRUCTURE OF THE N-TERMINAL DOMAIN OF E.COLI YBGF* | 0.04 |  | 12.24 | 0.16 | 147-195 | X-ray | 2.48 | homo-trimer | 1 x AUC | HHblits | 0.29 |
| ``` target    MKQVFLSTTTEFKEIDTLEPGTWINLVNPTQNESLEIANTFDIDIADLRAPLDAEEMSRITIEDEYTLIIVDVPVTEERN 2wz7.1    --------------------------------------------------------------------------------  target    NRTYYVTIPLGIIITEETIITTCLEPLPVLDVFINRRLRNFYTFMRSRFIFQILYRNAELYLTALRSIDRKSEQIESQLH 2wz7.1    ------------------------------------------------------------------SVEDRVTQLERISN  target    QS--TRNEELIELMELEKTIVYFKASLKTNERVIKKLTSSTSNIKKYLEDEDLLEDTLIETQQAIEMADIYGNVLHSMTE 2wz7.1    AHSQLLTQLQQQLSDNQSDIDSLRGQIQENQYQLNQV-------------------------------------------  target    TFASIISNNQNNIMKTLALVTIVMSIPTMVFSAYGMNFKDNEIPLNGEPNAFWLIVFIAFAMSVSLTLYLIHKKWF 2wz7.1    ---------------------------------------------------------------------------- ``` | | | | | | | | | | | | | | | | | | | | | | | | | | | | | | | | | | | | | | | | | | | | | | | | | |
|  | 2wz7.2.A | UNCHARACTERIZED PROTEIN YBGF  *CRYSTAL STRUCTURE OF THE N-TERMINAL DOMAIN OF E.COLI YBGF* | 0.04 |  | 12.24 | 0.16 | 147-195 | X-ray | 2.48 | homo-trimer | 2 x AU | HHblits | 0.29 |
| ``` target    MKQVFLSTTTEFKEIDTLEPGTWINLVNPTQNESLEIANTFDIDIADLRAPLDAEEMSRITIEDEYTLIIVDVPVTEERN 2wz7.2    --------------------------------------------------------------------------------  target    NRTYYVTIPLGIIITEETIITTCLEPLPVLDVFINRRLRNFYTFMRSRFIFQILYRNAELYLTALRSIDRKSEQIESQLH 2wz7.2    ------------------------------------------------------------------SVEDRVTQLERISN  target    QS--TRNEELIELMELEKTIVYFKASLKTNERVIKKLTSSTSNIKKYLEDEDLLEDTLIETQQAIEMADIYGNVLHSMTE 2wz7.2    AHSQLLTQLQQQLSDNQSDIDSLRGQIQENQYQLNQV-------------------------------------------  target    TFASIISNNQNNIMKTLALVTIVMSIPTMVFSAYGMNFKDNEIPLNGEPNAFWLIVFIAFAMSVSLTLYLIHKKWF 2wz7.2    ---------------------------------------------------------------------------- ``` | | | | | | | | | | | | | | | | | | | | | | | | | | | | | | | | | | | | | | | | | | | | | | | | | |
|  | 2wz7.2.B | UNCHARACTERIZED PROTEIN YBGF  *CRYSTAL STRUCTURE OF THE N-TERMINAL DOMAIN OF E.COLI YBGF* | 0.04 |  | 12.24 | 0.16 | 147-195 | X-ray | 2.48 | homo-trimer | 2 x AU | HHblits | 0.29 |
| ``` target    MKQVFLSTTTEFKEIDTLEPGTWINLVNPTQNESLEIANTFDIDIADLRAPLDAEEMSRITIEDEYTLIIVDVPVTEERN 2wz7.2    --------------------------------------------------------------------------------  target    NRTYYVTIPLGIIITEETIITTCLEPLPVLDVFINRRLRNFYTFMRSRFIFQILYRNAELYLTALRSIDRKSEQIESQLH 2wz7.2    ------------------------------------------------------------------SVEDRVTQLERISN  target    QS--TRNEELIELMELEKTIVYFKASLKTNERVIKKLTSSTSNIKKYLEDEDLLEDTLIETQQAIEMADIYGNVLHSMTE 2wz7.2    AHSQLLTQLQQQLSDNQSDIDSLRGQIQENQYQLNQV-------------------------------------------  target    TFASIISNNQNNIMKTLALVTIVMSIPTMVFSAYGMNFKDNEIPLNGEPNAFWLIVFIAFAMSVSLTLYLIHKKWF 2wz7.2    ---------------------------------------------------------------------------- ``` | | | | | | | | | | | | | | | | | | | | | | | | | | | | | | | | | | | | | | | | | | | | | | | | | |
|  | 2wz7.2.C | UNCHARACTERIZED PROTEIN YBGF  *CRYSTAL STRUCTURE OF THE N-TERMINAL DOMAIN OF E.COLI YBGF* | 0.04 |  | 12.24 | 0.16 | 147-195 | X-ray | 2.48 | homo-trimer | 2 x AU | HHblits | 0.29 |
| ``` target    MKQVFLSTTTEFKEIDTLEPGTWINLVNPTQNESLEIANTFDIDIADLRAPLDAEEMSRITIEDEYTLIIVDVPVTEERN 2wz7.2    --------------------------------------------------------------------------------  target    NRTYYVTIPLGIIITEETIITTCLEPLPVLDVFINRRLRNFYTFMRSRFIFQILYRNAELYLTALRSIDRKSEQIESQLH 2wz7.2    ------------------------------------------------------------------SVEDRVTQLERISN  target    QS--TRNEELIELMELEKTIVYFKASLKTNERVIKKLTSSTSNIKKYLEDEDLLEDTLIETQQAIEMADIYGNVLHSMTE 2wz7.2    AHSQLLTQLQQQLSDNQSDIDSLRGQIQENQYQLNQV-------------------------------------------  target    TFASIISNNQNNIMKTLALVTIVMSIPTMVFSAYGMNFKDNEIPLNGEPNAFWLIVFIAFAMSVSLTLYLIHKKWF 2wz7.2    ---------------------------------------------------------------------------- ``` | | | | | | | | | | | | | | | | | | | | | | | | | | | | | | | | | | | | | | | | | | | | | | | | | |
|  | 3zsu.1.A | TLL2057 PROTEIN  *Structure of the CyanoQ protein from Thermosynechococcus elongatus* | 0.05 |  | 16.33 | 0.16 | 137-185 | X-ray | 1.60 | monomer |  | HHblits | 0.28 |
| ``` target    MKQVFLSTTTEFKEIDTLEPGTWINLVNPTQNESLEIANTFDIDIADLRAPLDAEEMSRITIEDEYTLIIVDVPVTEERN 3zsu.1    --------------------------------------------------------------------------------  target    NRTYYVTIPLGIIITEETIITTCLEPLPVLDVFINRRLRNFYTFMRSRFIFQILYRNAELYLTALRSIDRKSEQIESQLH 3zsu.1    --------------------------------------------------------RIQDYLRDIEKNAERFADLEVSVA  target    QST----RNEELIELMELEKTIVYFKASLKTNERVIKKLTSSTSNIKKYLEDEDLLEDTLIETQQAIEMADIYGNVLHSM 3zsu.1    KGDWQEARNIMRGPLGEMLMDMRALNRNL---------------------------------------------------  target    TETFASIISNNQNNIMKTLALVTIVMSIPTMVFSAYGMNFKDNEIPLNGEPNAFWLIVFIAFAMSVSLTLYLIHKKWF 3zsu.1    ------------------------------------------------------------------------------ ``` | | | | | | | | | | | | | | | | | | | | | | | | | | | | | | | | | | | | | | | | | | | | | | | | | |
|  | 6ysl.1.A | Motility protein A  *Structure of the flagellar MotAB stator complex from Bacillus subtilis* | 0.03 |  | 16.67 | 0.15 | 252-302 | EM | 0.00 | hetero-5-2-mer |  | HHblits | 0.29 |
| ``` target    MKQVFLSTTTEFKEIDTLEPGTWINLVNPTQNESLEIANTFDIDIADLRAPLDAEEMSRITIEDEYTLIIVDVPVTEERN 6ysl.1    --------------------------------------------------------------------------------  target    NRTYYVTIPLGIIITEETIITTCLEPLPVLDVFINRRLRNFYTFMRSRFIFQILYRNAELYLTALRSIDRKSEQIESQLH 6ysl.1    --------------------------------------------------------------------------------  target    QSTRNEELIELMELEKTIVYFKASLKTNERVIKKLTSSTSNIKKYLEDEDLLEDTLIETQQAIEMADIYGNVLHSMTETF 6ysl.1    --------------------------------------------------------------------------------  target    ASIISNNQNNIMKTLALVTIVMSIPTMVFSAYGMNFKDNEIPLNGEPNAFWLIVFIAFAMSVSLTLYLIHKKWF 6ysl.1    -----------MDKTSLIGIILAFVALSVGMV-LKGVSF-SALA-NPAAILIIIAGTISAVV------------ ``` | | | | | | | | | | | | | | | | | | | | | | | | | | | | | | | | | | | | | | | | | | | | | | | | | |
|  | 6ysl.1.D | Motility protein A  *Structure of the flagellar MotAB stator complex from Bacillus subtilis* | 0.04 |  | 16.67 | 0.15 | 252-302 | EM | 0.00 | hetero-5-2-mer |  | HHblits | 0.29 |
| ``` target    MKQVFLSTTTEFKEIDTLEPGTWINLVNPTQNESLEIANTFDIDIADLRAPLDAEEMSRITIEDEYTLIIVDVPVTEERN 6ysl.1    --------------------------------------------------------------------------------  target    NRTYYVTIPLGIIITEETIITTCLEPLPVLDVFINRRLRNFYTFMRSRFIFQILYRNAELYLTALRSIDRKSEQIESQLH 6ysl.1    --------------------------------------------------------------------------------  target    QSTRNEELIELMELEKTIVYFKASLKTNERVIKKLTSSTSNIKKYLEDEDLLEDTLIETQQAIEMADIYGNVLHSMTETF 6ysl.1    --------------------------------------------------------------------------------  target    ASIISNNQNNIMKTLALVTIVMSIPTMVFSAYGMNFKDNEIPLNGEPNAFWLIVFIAFAMSVSLTLYLIHKKWF 6ysl.1    -----------MDKTSLIGIILAFVALSVGMV-LKGVSF-SALA-NPAAILIIIAGTISAVV------------ ``` | | | | | | | | | | | | | | | | | | | | | | | | | | | | | | | | | | | | | | | | | | | | | | | | | |
|  | 6ysl.1.E | Motility protein A  *Structure of the flagellar MotAB stator complex from Bacillus subtilis* | 0.04 |  | 16.67 | 0.15 | 252-302 | EM | 0.00 | hetero-5-2-mer |  | HHblits | 0.29 |
| ``` target    MKQVFLSTTTEFKEIDTLEPGTWINLVNPTQNESLEIANTFDIDIADLRAPLDAEEMSRITIEDEYTLIIVDVPVTEERN 6ysl.1    --------------------------------------------------------------------------------  target    NRTYYVTIPLGIIITEETIITTCLEPLPVLDVFINRRLRNFYTFMRSRFIFQILYRNAELYLTALRSIDRKSEQIESQLH 6ysl.1    --------------------------------------------------------------------------------  target    QSTRNEELIELMELEKTIVYFKASLKTNERVIKKLTSSTSNIKKYLEDEDLLEDTLIETQQAIEMADIYGNVLHSMTETF 6ysl.1    --------------------------------------------------------------------------------  target    ASIISNNQNNIMKTLALVTIVMSIPTMVFSAYGMNFKDNEIPLNGEPNAFWLIVFIAFAMSVSLTLYLIHKKWF 6ysl.1    -----------MDKTSLIGIILAFVALSVGMV-LKGVSF-SALA-NPAAILIIIAGTISAVV------------ ``` | | | | | | | | | | | | | | | | | | | | | | | | | | | | | | | | | | | | | | | | | | | | | | | | | |
|  | 6ysl.1.F | Motility protein A  *Structure of the flagellar MotAB stator complex from Bacillus subtilis* | 0.04 |  | 16.67 | 0.15 | 252-302 | EM | 0.00 | hetero-5-2-mer |  | HHblits | 0.29 |
| ``` target    MKQVFLSTTTEFKEIDTLEPGTWINLVNPTQNESLEIANTFDIDIADLRAPLDAEEMSRITIEDEYTLIIVDVPVTEERN 6ysl.1    --------------------------------------------------------------------------------  target    NRTYYVTIPLGIIITEETIITTCLEPLPVLDVFINRRLRNFYTFMRSRFIFQILYRNAELYLTALRSIDRKSEQIESQLH 6ysl.1    --------------------------------------------------------------------------------  target    QSTRNEELIELMELEKTIVYFKASLKTNERVIKKLTSSTSNIKKYLEDEDLLEDTLIETQQAIEMADIYGNVLHSMTETF 6ysl.1    --------------------------------------------------------------------------------  target    ASIISNNQNNIMKTLALVTIVMSIPTMVFSAYGMNFKDNEIPLNGEPNAFWLIVFIAFAMSVSLTLYLIHKKWF 6ysl.1    -----------MDKTSLIGIILAFVALSVGMV-LKGVSF-SALA-NPAAILIIIAGTISAVV------------ ``` | | | | | | | | | | | | | | | | | | | | | | | | | | | | | | | | | | | | | | | | | | | | | | | | | |
|  | 6ysl.1.G | Motility protein A  *Structure of the flagellar MotAB stator complex from Bacillus subtilis* | 0.04 |  | 16.67 | 0.15 | 252-302 | EM | 0.00 | hetero-5-2-mer |  | HHblits | 0.29 |
| ``` target    MKQVFLSTTTEFKEIDTLEPGTWINLVNPTQNESLEIANTFDIDIADLRAPLDAEEMSRITIEDEYTLIIVDVPVTEERN 6ysl.1    --------------------------------------------------------------------------------  target    NRTYYVTIPLGIIITEETIITTCLEPLPVLDVFINRRLRNFYTFMRSRFIFQILYRNAELYLTALRSIDRKSEQIESQLH 6ysl.1    --------------------------------------------------------------------------------  target    QSTRNEELIELMELEKTIVYFKASLKTNERVIKKLTSSTSNIKKYLEDEDLLEDTLIETQQAIEMADIYGNVLHSMTETF 6ysl.1    --------------------------------------------------------------------------------  target    ASIISNNQNNIMKTLALVTIVMSIPTMVFSAYGMNFKDNEIPLNGEPNAFWLIVFIAFAMSVSLTLYLIHKKWF 6ysl.1    -----------MDKTSLIGIILAFVALSVGMV-LKGVSF-SALA-NPAAILIIIAGTISAVV------------ ``` | | | | | | | | | | | | | | | | | | | | | | | | | | | | | | | | | | | | | | | | | | | | | | | | | |
|  | 2xzr.1.A | IMMUNOGLOBULIN-BINDING PROTEIN EIBD  *ESCHERICHIA COLI IMMUNOGLOBULIN-BINDING PROTEIN EIBD 391-438 FUSED TO GCN4 ADAPTORS* | 0.04 |  | 19.15 | 0.15 | 139-185 | X-ray | 2.80 | homo-trimer |  | HHblits | 0.30 |
| ``` target    MKQVFLSTTTEFKEIDTLEPGTWINLVNPTQNESLEIANTFDIDIADLRAPLDAEEMSRITIEDEYTLIIVDVPVTEERN 2xzr.1    --------------------------------------------------------------------------------  target    NRTYYVTIPLGIIITEETIITTCLEPLPVLDVFINRRLRNFYTFMRSRFIFQILYRNAELYLTALRSIDRKSEQIESQLH 2xzr.1    ----------------------------------------------------------QQHSARLDSQQRQINENHKEMK  target    QSTR--NEELIELMELEKTIVYFKASLKTNERVIKKLTSSTSNIKKYLEDEDLLEDTLIETQQAIEMADIYGNVLHSMTE 2xzr.1    QIEDKIEEILSKIYHIENEIARIKKLI-----------------------------------------------------  target    TFASIISNNQNNIMKTLALVTIVMSIPTMVFSAYGMNFKDNEIPLNGEPNAFWLIVFIAFAMSVSLTLYLIHKKWF 2xzr.1    ---------------------------------------------------------------------------- ``` | | | | | | | | | | | | | | | | | | | | | | | | | | | | | | | | | | | | | | | | | | | | | | | | | |
|  | 6vag.1.A | Phosphoprotein  *Crystal structure of the oligomerization domain of phosphoprotein from parainfluenza virus 5* | 0.05 |  | 20.00 | 0.14 | 140-186 | X-ray | 1.40 | homo-tetramer |  | HHblits | 0.32 |
| ``` target    MKQVFLSTTTEFKEIDTLEPGTWINLVNPTQNESLEIANTFDIDIADLRAPLDAEEMSRITIEDEYTLIIVDVPVTEERN 6vag.1    --------------------------------------------------------------------------------  target    NRTYYVTIPLGIIITEETIITTCLEPLPVLDVFINRRLRNFYTFMRSRFIFQILYRNAELYLTALRSIDRKSEQIESQLH 6vag.1    -----------------------------------------------------------EILNTVRNLDSRMNQLETKVD  target    QSTRNEELIELMELEKTIVYFKASLKTNERVIKKLTSSTSNIKKYLEDEDLLEDTLIETQQAIEMADIYGNVLHSMTETF 6vag.1    RI--LSSQSLIQTIKNDIVGLKAGMA------------------------------------------------------  target    ASIISNNQNNIMKTLALVTIVMSIPTMVFSAYGMNFKDNEIPLNGEPNAFWLIVFIAFAMSVSLTLYLIHKKWF 6vag.1    -------------------------------------------------------------------------- ``` | | | | | | | | | | | | | | | | | | | | | | | | | | | | | | | | | | | | | | | | | | | | | | | | | |
|  | 6vag.1.B | Phosphoprotein  *Crystal structure of the oligomerization domain of phosphoprotein from parainfluenza virus 5* | 0.04 |  | 20.00 | 0.14 | 140-186 | X-ray | 1.40 | homo-tetramer |  | HHblits | 0.32 |
| ``` target    MKQVFLSTTTEFKEIDTLEPGTWINLVNPTQNESLEIANTFDIDIADLRAPLDAEEMSRITIEDEYTLIIVDVPVTEERN 6vag.1    --------------------------------------------------------------------------------  target    NRTYYVTIPLGIIITEETIITTCLEPLPVLDVFINRRLRNFYTFMRSRFIFQILYRNAELYLTALRSIDRKSEQIESQLH 6vag.1    -----------------------------------------------------------EILNTVRNLDSRMNQLETKVD  target    QSTRNEELIELMELEKTIVYFKASLKTNERVIKKLTSSTSNIKKYLEDEDLLEDTLIETQQAIEMADIYGNVLHSMTETF 6vag.1    RI--LSSQSLIQTIKNDIVGLKAGMA------------------------------------------------------  target    ASIISNNQNNIMKTLALVTIVMSIPTMVFSAYGMNFKDNEIPLNGEPNAFWLIVFIAFAMSVSLTLYLIHKKWF 6vag.1    -------------------------------------------------------------------------- ``` | | | | | | | | | | | | | | | | | | | | | | | | | | | | | | | | | | | | | | | | | | | | | | | | | |
|  | 7kdp.1.A | Envelope glycoprotein B  *HCMV prefusion gB in complex with fusion inhibitor WAY-174865* | 0.02 |  | 15.22 | 0.15 | 264-310 | EM | 0.00 | homo-trimer | 30 x NAG, 3 x WCY | HHblits | 0.27 |
| ``` target    MKQVFLSTTTEFKEIDTLEPGTWINLVNPTQNESLEIANTFDIDIADLRAPLDAEEMSRITIEDEYTLIIVDVPVTEERN 7kdp.1    --------------------------------------------------------------------------------  target    NRTYYVTIPLGIIITEETIITTCLEPLPVLDVFINRRLRNFYTFMRSRFIFQILYRNAELYLTALRSIDRKSEQIESQLH 7kdp.1    --------------------------------------------------------------------------------  target    QSTRNEELIELMELEKTIVYFKASLKTNERVIKKLTSSTSNIKKYLEDEDLLEDTLIETQQAIEMADIYGNVLHSMTETF 7kdp.1    --------------------------------------------------------------------------------  target    ASIISNNQNNIMKTLALVTIVMSIPTMVFSAYGMNFKDNEIPLNGEPNAFWLIVFIAFAMSVSLTLYLIHKKWF 7kdp.1    -----------------------AIGAVGGAVASVVEG-VATFLKNPFGAFTIILVAIAVVIIIYLIYTR---- ``` | | | | | | | | | | | | | | | | | | | | | | | | | | | | | | | | | | | | | | | | | | | | | | | | | |
|  | 5c3l.1.A | Nup54  *Structure of the metazoan Nup62.Nup58.Nup54 nucleoporin complex.* | 0.04 |  | 4.76 | 0.13 | 137-183 | X-ray | 2.90 | hetero-oligomer |  | HHblits | 0.27 |
| ``` target    MKQVFLSTTTEFKEIDTLEPGTWINLVNPTQNESLEIANTFDIDIADLRAPLDAEEMSRITIEDEYTLIIVDVPVTEERN 5c3l.1    --------------------------------------------------------------------------------  target    NRTYYVTIPLGIIITEETIITTCLEPLPVLDVFINRRLRNFYTFMRSRFIFQILYRNAELYLTALRSIDRKSEQIESQLH 5c3l.1    --------------------------------------------------------MTKQHQSRLDIISEDIGELQKNQ-  target    QSTRNEELIELMELEKTIVYFKASLKTNERVIKKLTSSTSNIKKYLEDEDLLEDTLIETQQAIEMADIYGNVLHSMTETF 5c3l.1    ----TTTMAKIGQYKRKLMELSH---------------------------------------------------------  target    ASIISNNQNNIMKTLALVTIVMSIPTMVFSAYGMNFKDNEIPLNGEPNAFWLIVFIAFAMSVSLTLYLIHKKWF 5c3l.1    -------------------------------------------------------------------------- ``` | | | | | | | | | | | | | | | | | | | | | | | | | | | | | | | | | | | | | | | | | | | | | | | | | |
|  | 6dlc.1.A | Designed protein DHD1:234\_A  *Designed protein DHD1:234\_A, Designed protein DHD1:234\_B* | 0.02 |  | 18.42 | 0.12 | 137-174 | X-ray | 3.26 | hetero-2-2-mer |  | HHblits | 0.31 |
| ``` target    MKQVFLSTTTEFKEIDTLEPGTWINLVNPTQNESLEIANTFDIDIADLRAPLDAEEMSRITIEDEYTLIIVDVPVTEERN 6dlc.1    --------------------------------------------------------------------------------  target    NRTYYVTIPLGIIITEETIITTCLEPLPVLDVFINRRLRNFYTFMRSRFIFQILYRNAELYLTALRSIDRKSEQIESQLH 6dlc.1    --------------------------------------------------------FLENLRRHLDRLDKHIKQLRDILS  target    QSTRNEELIELMELEKTIVYFKASLKTNERVIKKLTSSTSNIKKYLEDEDLLEDTLIETQQAIEMADIYGNVLHSMTETF 6dlc.1    ENPEDERVKDAIDL------------------------------------------------------------------  target    ASIISNNQNNIMKTLALVTIVMSIPTMVFSAYGMNFKDNEIPLNGEPNAFWLIVFIAFAMSVSLTLYLIHKKWF 6dlc.1    -------------------------------------------------------------------------- ``` | | | | | | | | | | | | | | | | | | | | | | | | | | | | | | | | | | | | | | | | | | | | | | | | | |
|  | 6xns.1.A | C3\_crown-05  *C3\_crown-05* | 0.02 |  | 18.92 | 0.12 | 139-175 | X-ray | 3.19 | homo-trimer |  | HHblits | 0.32 |
| ``` target    MKQVFLSTTTEFKEIDTLEPGTWINLVNPTQNESLEIANTFDIDIADLRAPLDAEEMSRITIEDEYTLIIVDVPVTEERN 6xns.1    --------------------------------------------------------------------------------  target    NRTYYVTIPLGIIITEETIITTCLEPLPVLDVFINRRLRNFYTFMRSRFIFQILYRNAELYLTALRSIDRKSEQIESQLH 6xns.1    ----------------------------------------------------------ENLRRHLDRLDKHIKQLRDILS  target    QSTRNEELIELMELEKTIVYFKASLKTNERVIKKLTSSTSNIKKYLEDEDLLEDTLIETQQAIEMADIYGNVLHSMTETF 6xns.1    ENPEDERVKDVIDLS-----------------------------------------------------------------  target    ASIISNNQNNIMKTLALVTIVMSIPTMVFSAYGMNFKDNEIPLNGEPNAFWLIVFIAFAMSVSLTLYLIHKKWF 6xns.1    -------------------------------------------------------------------------- ``` | | | | | | | | | | | | | | | | | | | | | | | | | | | | | | | | | | | | | | | | | | | | | | | | | |
|  | 6xns.1.B | C3\_crown-05  *C3\_crown-05* | 0.02 |  | 18.92 | 0.12 | 139-175 | X-ray | 3.19 | homo-trimer |  | HHblits | 0.32 |
| ``` target    MKQVFLSTTTEFKEIDTLEPGTWINLVNPTQNESLEIANTFDIDIADLRAPLDAEEMSRITIEDEYTLIIVDVPVTEERN 6xns.1    --------------------------------------------------------------------------------  target    NRTYYVTIPLGIIITEETIITTCLEPLPVLDVFINRRLRNFYTFMRSRFIFQILYRNAELYLTALRSIDRKSEQIESQLH 6xns.1    ----------------------------------------------------------ENLRRHLDRLDKHIKQLRDILS  target    QSTRNEELIELMELEKTIVYFKASLKTNERVIKKLTSSTSNIKKYLEDEDLLEDTLIETQQAIEMADIYGNVLHSMTETF 6xns.1    ENPEDERVKDVIDLS-----------------------------------------------------------------  target    ASIISNNQNNIMKTLALVTIVMSIPTMVFSAYGMNFKDNEIPLNGEPNAFWLIVFIAFAMSVSLTLYLIHKKWF 6xns.1    -------------------------------------------------------------------------- ``` | | | | | | | | | | | | | | | | | | | | | | | | | | | | | | | | | | | | | | | | | | | | | | | | | |
|  | 6xns.1.C | C3\_crown-05  *C3\_crown-05* | 0.02 |  | 18.92 | 0.12 | 139-175 | X-ray | 3.19 | homo-trimer |  | HHblits | 0.32 |
| ``` target    MKQVFLSTTTEFKEIDTLEPGTWINLVNPTQNESLEIANTFDIDIADLRAPLDAEEMSRITIEDEYTLIIVDVPVTEERN 6xns.1    --------------------------------------------------------------------------------  target    NRTYYVTIPLGIIITEETIITTCLEPLPVLDVFINRRLRNFYTFMRSRFIFQILYRNAELYLTALRSIDRKSEQIESQLH 6xns.1    ----------------------------------------------------------ENLRRHLDRLDKHIKQLRDILS  target    QSTRNEELIELMELEKTIVYFKASLKTNERVIKKLTSSTSNIKKYLEDEDLLEDTLIETQQAIEMADIYGNVLHSMTETF 6xns.1    ENPEDERVKDVIDLS-----------------------------------------------------------------  target    ASIISNNQNNIMKTLALVTIVMSIPTMVFSAYGMNFKDNEIPLNGEPNAFWLIVFIAFAMSVSLTLYLIHKKWF 6xns.1    -------------------------------------------------------------------------- ``` | | | | | | | | | | | | | | | | | | | | | | | | | | | | | | | | | | | | | | | | | | | | | | | | | |
|  | 6xns.2.A | C3\_crown-05  *C3\_crown-05* | 0.02 |  | 18.92 | 0.12 | 139-175 | X-ray | 3.19 | homo-trimer |  | HHblits | 0.32 |
| ``` target    MKQVFLSTTTEFKEIDTLEPGTWINLVNPTQNESLEIANTFDIDIADLRAPLDAEEMSRITIEDEYTLIIVDVPVTEERN 6xns.2    --------------------------------------------------------------------------------  target    NRTYYVTIPLGIIITEETIITTCLEPLPVLDVFINRRLRNFYTFMRSRFIFQILYRNAELYLTALRSIDRKSEQIESQLH 6xns.2    ----------------------------------------------------------ENLRRHLDRLDKHIKQLRDILS  target    QSTRNEELIELMELEKTIVYFKASLKTNERVIKKLTSSTSNIKKYLEDEDLLEDTLIETQQAIEMADIYGNVLHSMTETF 6xns.2    ENPEDERVKDVIDLS-----------------------------------------------------------------  target    ASIISNNQNNIMKTLALVTIVMSIPTMVFSAYGMNFKDNEIPLNGEPNAFWLIVFIAFAMSVSLTLYLIHKKWF 6xns.2    -------------------------------------------------------------------------- ``` | | | | | | | | | | | | | | | | | | | | | | | | | | | | | | | | | | | | | | | | | | | | | | | | | |
|  | 6xns.2.B | C3\_crown-05  *C3\_crown-05* | 0.02 |  | 18.92 | 0.12 | 139-175 | X-ray | 3.19 | homo-trimer |  | HHblits | 0.32 |
| ``` target    MKQVFLSTTTEFKEIDTLEPGTWINLVNPTQNESLEIANTFDIDIADLRAPLDAEEMSRITIEDEYTLIIVDVPVTEERN 6xns.2    --------------------------------------------------------------------------------  target    NRTYYVTIPLGIIITEETIITTCLEPLPVLDVFINRRLRNFYTFMRSRFIFQILYRNAELYLTALRSIDRKSEQIESQLH 6xns.2    ----------------------------------------------------------ENLRRHLDRLDKHIKQLRDILS  target    QSTRNEELIELMELEKTIVYFKASLKTNERVIKKLTSSTSNIKKYLEDEDLLEDTLIETQQAIEMADIYGNVLHSMTETF 6xns.2    ENPEDERVKDVIDLS-----------------------------------------------------------------  target    ASIISNNQNNIMKTLALVTIVMSIPTMVFSAYGMNFKDNEIPLNGEPNAFWLIVFIAFAMSVSLTLYLIHKKWF 6xns.2    -------------------------------------------------------------------------- ``` | | | | | | | | | | | | | | | | | | | | | | | | | | | | | | | | | | | | | | | | | | | | | | | | | |
|  | 6xns.2.C | C3\_crown-05  *C3\_crown-05* | 0.02 |  | 18.92 | 0.12 | 139-175 | X-ray | 3.19 | homo-trimer |  | HHblits | 0.32 |
| ``` target    MKQVFLSTTTEFKEIDTLEPGTWINLVNPTQNESLEIANTFDIDIADLRAPLDAEEMSRITIEDEYTLIIVDVPVTEERN 6xns.2    --------------------------------------------------------------------------------  target    NRTYYVTIPLGIIITEETIITTCLEPLPVLDVFINRRLRNFYTFMRSRFIFQILYRNAELYLTALRSIDRKSEQIESQLH 6xns.2    ----------------------------------------------------------ENLRRHLDRLDKHIKQLRDILS  target    QSTRNEELIELMELEKTIVYFKASLKTNERVIKKLTSSTSNIKKYLEDEDLLEDTLIETQQAIEMADIYGNVLHSMTETF 6xns.2    ENPEDERVKDVIDLS-----------------------------------------------------------------  target    ASIISNNQNNIMKTLALVTIVMSIPTMVFSAYGMNFKDNEIPLNGEPNAFWLIVFIAFAMSVSLTLYLIHKKWF 6xns.2    -------------------------------------------------------------------------- ``` | | | | | | | | | | | | | | | | | | | | | | | | | | | | | | | | | | | | | | | | | | | | | | | | | |
|  | 6znl.1.O | Dynactin subunit 3  *Cryo-EM structure of the dynactin complex* | 0.02 |  | 8.11 | 0.12 | 144-180 | EM | 0.00 | hetero-8-1-1-1-1-4-… | 9 x ADP, 1 x ATP, 3 x ZN | HHblits | 0.27 |
| ``` target    MKQVFLSTTTEFKEIDTLEPGTWINLVNPTQNESLEIANTFDIDIADLRAPLDAEEMSRITIEDEYTLIIVDVPVTEERN 6znl.1    --------------------------------------------------------------------------------  target    NRTYYVTIPLGIIITEETIITTCLEPLPVLDVFINRRLRNFYTFMRSRFIFQILYRNAELYLTALRSIDRKSEQIESQLH 6znl.1    ---------------------------------------------------------------DVQRLQARLEELERWVY  target    QSTR----NEELIELMELEKTIVYFKASLKTNERVIKKLTSSTSNIKKYLEDEDLLEDTLIETQQAIEMADIYGNVLHSM 6znl.1    GPGGSRGSRKVADGLVKVQVALGN--------------------------------------------------------  target    TETFASIISNNQNNIMKTLALVTIVMSIPTMVFSAYGMNFKDNEIPLNGEPNAFWLIVFIAFAMSVSLTLYLIHKKWF 6znl.1    ------------------------------------------------------------------------------ ``` | | | | | | | | | | | | | | | | | | | | | | | | | | | | | | | | | | | | | | | | | | | | | | | | | |
|  | 6znl.1.V | Dynactin subunit 3  *Cryo-EM structure of the dynactin complex* | 0.03 |  | 8.11 | 0.12 | 144-180 | EM | 0.00 | hetero-8-1-1-1-1-4-… | 9 x ADP, 1 x ATP, 3 x ZN | HHblits | 0.27 |
| ``` target    MKQVFLSTTTEFKEIDTLEPGTWINLVNPTQNESLEIANTFDIDIADLRAPLDAEEMSRITIEDEYTLIIVDVPVTEERN 6znl.1    --------------------------------------------------------------------------------  target    NRTYYVTIPLGIIITEETIITTCLEPLPVLDVFINRRLRNFYTFMRSRFIFQILYRNAELYLTALRSIDRKSEQIESQLH 6znl.1    ---------------------------------------------------------------DVQRLQARLEELERWVY  target    QSTR----NEELIELMELEKTIVYFKASLKTNERVIKKLTSSTSNIKKYLEDEDLLEDTLIETQQAIEMADIYGNVLHSM 6znl.1    GPGGSRGSRKVADGLVKVQVALGN--------------------------------------------------------  target    TETFASIISNNQNNIMKTLALVTIVMSIPTMVFSAYGMNFKDNEIPLNGEPNAFWLIVFIAFAMSVSLTLYLIHKKWF 6znl.1    ------------------------------------------------------------------------------ ``` | | | | | | | | | | | | | | | | | | | | | | | | | | | | | | | | | | | | | | | | | | | | | | | | | |
|  | 6ye4.1.A | Biopolymer transport protein ExbB  *Structure of ExbB pentamer from Serratia marcescens by single particle cryo electron microscopy* | 0.02 |  | 13.89 | 0.11 | 241-276 | EM | 0.00 | homo-pentamer | 5 x PGT | HHblits | 0.28 |
| ``` target    MKQVFLSTTTEFKEIDTLEPGTWINLVNPTQNESLEIANTFDIDIADLRAPLDAEEMSRITIEDEYTLIIVDVPVTEERN 6ye4.1    --------------------------------------------------------------------------------  target    NRTYYVTIPLGIIITEETIITTCLEPLPVLDVFINRRLRNFYTFMRSRFIFQILYRNAELYLTALRSIDRKSEQIESQLH 6ye4.1    --------------------------------------------------------------------------------  target    QSTRNEELIELMELEKTIVYFKASLKTNERVIKKLTSSTSNIKKYLEDEDLLEDTLIETQQAIEMADIYGNVLHSMTETF 6ye4.1    --------------------------------------------------------------------------------  target    ASIISNNQNNIMKTLALVTIVMSIPTMVFSAYGMNFKDNEIPLNGEPNAFWLIVFIAFAMSVSLTLYLIHKKWF 6ye4.1    VAAYSRNMGRGNGFLATIGAISPFVGLFGTVWGIMN-------------------------------------- ``` | | | | | | | | | | | | | | | | | | | | | | | | | | | | | | | | | | | | | | | | | | | | | | | | | |
|  | 6f0k.1.C | Polysulphide reductase NrfD  *Alternative complex III* | 0.02 |  | 13.89 | 0.11 | 248-287 | EM | 0.00 | hetero-1-1-1-1-1-1-… | 6 x HEC, 1 x F3S, 3 x SF4 | HHblits | 0.28 |
| ``` target    MKQVFLSTTTEFKEIDTLEPGTWINLVNPTQNESLEIANTFDIDIADLRAPLDAEEMSRITIEDEYTLIIVDVPVTEERN 6f0k.1    --------------------------------------------------------------------------------  target    NRTYYVTIPLGIIITEETIITTCLEPLPVLDVFINRRLRNFYTFMRSRFIFQILYRNAELYLTALRSIDRKSEQIESQLH 6f0k.1    --------------------------------------------------------------------------------  target    QSTRNEELIELMELEKTIVYFKASLKTNERVIKKLTSSTSNIKKYLEDEDLLEDTLIETQQAIEMADIYGNVLHSMTETF 6f0k.1    --------------------------------------------------------------------------------  target    ASIISNNQNNIMKTLALVTIVMSI--PTMVFSAYGMNFKDNEIPLNGEPNAFWLIVFIAFAMSVSLTLYLIHKKWF 6f0k.1    -------YEKVYMLLAGLATPLVLSVHSVVSFDFAVSI----IPGWHTT--------------------------- ``` | | | | | | | | | | | | | | | | | | | | | | | | | | | | | | | | | | | | | | | | | | | | | | | | | |
|  | 6lod.1.C | Polysulphide reductase NrfD  *Cryo-EM structure of the air-oxidized photosynthetic alternative complex III from Roseiflexus castenholzii* | 0.02 |  | 8.11 | 0.12 | 247-287 | EM | 0.00 | hetero-1-1-1-1-1-1-… | 6 x HEC, 2 x EL6, 3 x SF4, 1 x F3S | HHblits | 0.25 |
| ``` target    MKQVFLSTTTEFKEIDTLEPGTWINLVNPTQNESLEIANTFDIDIADLRAPLDAEEMSRITIEDEYTLIIVDVPVTEERN 6lod.1    --------------------------------------------------------------------------------  target    NRTYYVTIPLGIIITEETIITTCLEPLPVLDVFINRRLRNFYTFMRSRFIFQILYRNAELYLTALRSIDRKSEQIESQLH 6lod.1    --------------------------------------------------------------------------------  target    QSTRNEELIELMELEKTIVYFKASLKTNERVIKKLTSSTSNIKKYLEDEDLLEDTLIETQQAIEMADIYGNVLHSMTETF 6lod.1    --------------------------------------------------------------------------------  target    ASIISNNQNNIMKTLALVTIVMS--IPTMVFSAYGMNFKDNEIPLNGEPNAFWLIVFIAFAMSVSLTLYLIHKKWF 6lod.1    ------RYEVASLILAGLSTPLVLSVHSIISLDFAISQ----LPGWHVT--------------------------- ``` | | | | | | | | | | | | | | | | | | | | | | | | | | | | | | | | | | | | | | | | | | | | | | | | | |
|  | 4n21.1.A | GP2 Ectodomain  *Crystal structure of the GP2 Core Domain from the California Academy of Science Virus* | 0.02 |  | 17.14 | 0.11 | 165-199 | X-ray | 1.99 | homo-trimer |  | HHblits | 0.27 |
| ``` target    MKQVFLSTTTEFKEIDTLEPGTWINLVNPTQNESLEIANTFDIDIADLRAPLDAEEMSRITIEDEYTLIIVDVPVTEERN 4n21.1    --------------------------------------------------------------------------------  target    NRTYYVTIPLGIIITEETIITTCLEPLPVLDVFINRRLRNFYTFMRSRFIFQILYRNAELYLTALRSIDRKSEQIESQLH 4n21.1    --------------------------------------------------------------------------------  target    QSTRNEELIELMELEKTIVYFKASLKTNERVIKKLTSSTSNIKKYLEDEDLLEDTLIETQQAIEMADIYGNVLHSMTETF 4n21.1    ----NYTTNALFLLNKEESEIRDHVVEHELALNYLLAHQ-----------------------------------------  target    ASIISNNQNNIMKTLALVTIVMSIPTMVFSAYGMNFKDNEIPLNGEPNAFWLIVFIAFAMSVSLTLYLIHKKWF 4n21.1    -------------------------------------------------------------------------- ``` | | | | | | | | | | | | | | | | | | | | | | | | | | | | | | | | | | | | | | | | | | | | | | | | | |
|  | 4n21.2.B | GP2 Ectodomain  *Crystal structure of the GP2 Core Domain from the California Academy of Science Virus* | 0.03 |  | 17.14 | 0.11 | 165-199 | X-ray | 1.99 | homo-trimer |  | HHblits | 0.27 |
| ``` target    MKQVFLSTTTEFKEIDTLEPGTWINLVNPTQNESLEIANTFDIDIADLRAPLDAEEMSRITIEDEYTLIIVDVPVTEERN 4n21.2    --------------------------------------------------------------------------------  target    NRTYYVTIPLGIIITEETIITTCLEPLPVLDVFINRRLRNFYTFMRSRFIFQILYRNAELYLTALRSIDRKSEQIESQLH 4n21.2    --------------------------------------------------------------------------------  target    QSTRNEELIELMELEKTIVYFKASLKTNERVIKKLTSSTSNIKKYLEDEDLLEDTLIETQQAIEMADIYGNVLHSMTETF 4n21.2    ----NYTTNALFLLNKEESEIRDHVVEHELALNYLLAHQ-----------------------------------------  target    ASIISNNQNNIMKTLALVTIVMSIPTMVFSAYGMNFKDNEIPLNGEPNAFWLIVFIAFAMSVSLTLYLIHKKWF 4n21.2    -------------------------------------------------------------------------- ``` | | | | | | | | | | | | | | | | | | | | | | | | | | | | | | | | | | | | | | | | | | | | | | | | | |
|  | 6zz6.1.B | Structural maintenance of chromosomes protein 3,Structural maintenance of chromosomes protein 3,Structural maintenance of chromosomes protein 3  *Cryo-EM structure of S.cerevisiae cohesin-Scc2-DNA complex* | 0.01 |  | 5.41 | 0.12 | 217-253 | EM | 0.00 | hetero-1-1-1-1-mer | 2 x ATP, 2 x MG | HHblits | 0.23 |
| ``` target    MKQVFLSTTTEFKEIDTLEPGTWINLVNPTQNESLEIANTFDIDIADLRAPLDAEEMSRITIEDEYTLIIVDVPVTEERN 6zz6.1    --------------------------------------------------------------------------------  target    NRTYYVTIPLGIIITEETIITTCLEPLPVLDVFINRRLRNFYTFMRSRFIFQILYRNAELYLTALRSIDRKSEQIESQLH 6zz6.1    --------------------------------------------------------------------------------  target    QSTRNEELIELMELEKTIVYFKASLKTNERVIKKLTSSTSNIKKYLEDEDLLEDTLIETQQAIEMADIYGNVLHSMTETF 6zz6.1    --------------------------------------------------------SELDESKDSIQDLIVKLKQQKVNA  target    ASIISNNQNNIMKTLALVTIVMSIPTMVFSAYGMNFKDNEIPLNGEPNAFWLIVFIAFAMSVSLTLYLIHKKWF 6zz6.1    VDSTFQKVSENFE------------------------------------------------------------- ``` | | | | | | | | | | | | | | | | | | | | | | | | | | | | | | | | | | | | | | | | | | | | | | | | | |
|  | 4jpr.1.A | ASLV fusion TM  *Structure of the ASLV fusion subunit core* | 0.02 |  | 17.65 | 0.11 | 165-198 | X-ray | 2.00 | homo-trimer |  | HHblits | 0.28 |
| ``` target    MKQVFLSTTTEFKEIDTLEPGTWINLVNPTQNESLEIANTFDIDIADLRAPLDAEEMSRITIEDEYTLIIVDVPVTEERN 4jpr.1    --------------------------------------------------------------------------------  target    NRTYYVTIPLGIIITEETIITTCLEPLPVLDVFINRRLRNFYTFMRSRFIFQILYRNAELYLTALRSIDRKSEQIESQLH 4jpr.1    --------------------------------------------------------------------------------  target    QSTRNEELIELMELEKTIVYFKASLKTNERVIKKLTSSTSNIKKYLEDEDLLEDTLIETQQAIEMADIYGNVLHSMTETF 4jpr.1    ----NLTTSLLGDLLDDVTSIRHAVLQNRAAIDFLLLA------------------------------------------  target    ASIISNNQNNIMKTLALVTIVMSIPTMVFSAYGMNFKDNEIPLNGEPNAFWLIVFIAFAMSVSLTLYLIHKKWF 4jpr.1    -------------------------------------------------------------------------- ``` | | | | | | | | | | | | | | | | | | | | | | | | | | | | | | | | | | | | | | | | | | | | | | | | | |
|  | 6ncn.1.A | Apolipoprotein E  *Fragment-based Discovery of an apoE4 Stabilizer* | 0.02 |  | 15.63 | 0.10 | 225-256 | X-ray | 1.82 | monomer | 1 x KJM | HHblits | 0.27 |
| ``` target    MKQVFLSTTTEFKEIDTLEPGTWINLVNPTQNESLEIANTFDIDIADLRAPLDAEEMSRITIEDEYTLIIVDVPVTEERN 6ncn.1    --------------------------------------------------------------------------------  target    NRTYYVTIPLGIIITEETIITTCLEPLPVLDVFINRRLRNFYTFMRSRFIFQILYRNAELYLTALRSIDRKSEQIESQLH 6ncn.1    --------------------------------------------------------------------------------  target    QSTRNEELIELMELEKTIVYFKASLKTNERVIKKLTSSTSNIKKYLEDEDLLEDTLIETQQAIEMADIYGNVLHSMTETF 6ncn.1    ----------------------------------------------------------------STEELRVRLASHLRKL  target    ASIISNNQNNIMKTLALVTIVMSIPTMVFSAYGMNFKDNEIPLNGEPNAFWLIVFIAFAMSVSLTLYLIHKKWF 6ncn.1    RKRLLRDADDLQKRLA---------------------------------------------------------- ``` | | | | | | | | | | | | | | | | | | | | | | | | | | | | | | | | | | | | | | | | | | | | | | | | | |
|  | 5zuv.1.A | Spike glycoprotein,Spike glycoprotein,inhibitor EK1  *Crystal Structure of the Human Coronavirus 229E HR1 motif in complex with pan-CoVs inhibitor EK1* | 0.00 |  | 26.67 | 0.10 | 168-197 | X-ray | 2.21 | homo-trimer |  | HHblits | 0.32 |
| ``` target    MKQVFLSTTTEFKEIDTLEPGTWINLVNPTQNESLEIANTFDIDIADLRAPLDAEEMSRITIEDEYTLIIVDVPVTEERN 5zuv.1    --------------------------------------------------------------------------------  target    NRTYYVTIPLGIIITEETIITTCLEPLPVLDVFINRRLRNFYTFMRSRFIFQILYRNAELYLTALRSIDRKSEQIESQLH 5zuv.1    --------------------------------------------------------------------------------  target    QSTRNEELIELMELEKTIVYFKASLKTNERVIKKLTSSTSNIKKYLEDEDLLEDTLIETQQAIEMADIYGNVLHSMTETF 5zuv.1    -------GGSLDQINVTFLDLEYEMKKLEEAIKKLEE-------------------------------------------  target    ASIISNNQNNIMKTLALVTIVMSIPTMVFSAYGMNFKDNEIPLNGEPNAFWLIVFIAFAMSVSLTLYLIHKKWF 5zuv.1    -------------------------------------------------------------------------- ``` | | | | | | | | | | | | | | | | | | | | | | | | | | | | | | | | | | | | | | | | | | | | | | | | | |
|  | 5zuv.1.B | Spike glycoprotein,Spike glycoprotein,inhibitor EK1  *Crystal Structure of the Human Coronavirus 229E HR1 motif in complex with pan-CoVs inhibitor EK1* | 0.00 |  | 26.67 | 0.10 | 168-197 | X-ray | 2.21 | homo-trimer |  | HHblits | 0.32 |
| ``` target    MKQVFLSTTTEFKEIDTLEPGTWINLVNPTQNESLEIANTFDIDIADLRAPLDAEEMSRITIEDEYTLIIVDVPVTEERN 5zuv.1    --------------------------------------------------------------------------------  target    NRTYYVTIPLGIIITEETIITTCLEPLPVLDVFINRRLRNFYTFMRSRFIFQILYRNAELYLTALRSIDRKSEQIESQLH 5zuv.1    --------------------------------------------------------------------------------  target    QSTRNEELIELMELEKTIVYFKASLKTNERVIKKLTSSTSNIKKYLEDEDLLEDTLIETQQAIEMADIYGNVLHSMTETF 5zuv.1    -------GGSLDQINVTFLDLEYEMKKLEEAIKKLEE-------------------------------------------  target    ASIISNNQNNIMKTLALVTIVMSIPTMVFSAYGMNFKDNEIPLNGEPNAFWLIVFIAFAMSVSLTLYLIHKKWF 5zuv.1    -------------------------------------------------------------------------- ``` | | | | | | | | | | | | | | | | | | | | | | | | | | | | | | | | | | | | | | | | | | | | | | | | | |
|  | 5zuv.1.C | Spike glycoprotein,Spike glycoprotein,inhibitor EK1  *Crystal Structure of the Human Coronavirus 229E HR1 motif in complex with pan-CoVs inhibitor EK1* | 0.00 |  | 26.67 | 0.10 | 168-197 | X-ray | 2.21 | homo-trimer |  | HHblits | 0.32 |
| ``` target    MKQVFLSTTTEFKEIDTLEPGTWINLVNPTQNESLEIANTFDIDIADLRAPLDAEEMSRITIEDEYTLIIVDVPVTEERN 5zuv.1    --------------------------------------------------------------------------------  target    NRTYYVTIPLGIIITEETIITTCLEPLPVLDVFINRRLRNFYTFMRSRFIFQILYRNAELYLTALRSIDRKSEQIESQLH 5zuv.1    --------------------------------------------------------------------------------  target    QSTRNEELIELMELEKTIVYFKASLKTNERVIKKLTSSTSNIKKYLEDEDLLEDTLIETQQAIEMADIYGNVLHSMTETF 5zuv.1    -------GGSLDQINVTFLDLEYEMKKLEEAIKKLEE-------------------------------------------  target    ASIISNNQNNIMKTLALVTIVMSIPTMVFSAYGMNFKDNEIPLNGEPNAFWLIVFIAFAMSVSLTLYLIHKKWF 5zuv.1    -------------------------------------------------------------------------- ``` | | | | | | | | | | | | | | | | | | | | | | | | | | | | | | | | | | | | | | | | | | | | | | | | | |
|  | 6zyw.1.C | Dynein heavy chain, outer arm protein  *Outer Dynein Arm-Shulin complex - overall structure (Tetrahymena thermophila)* | 0.02 |  | 9.68 | 0.10 | 133-163 | EM | 0.00 | hetero-1-1-1-2-2-1-… | 3 x ADP, 1 x ATP, 1 x GTP | HHblits | 0.26 |
| ``` target    MKQVFLSTTTEFKEIDTLEPGTWINLVNPTQNESLEIANTFDIDIADLRAPLDAEEMSRITIEDEYTLIIVDVPVTEERN 6zyw.1    --------------------------------------------------------------------------------  target    NRTYYVTIPLGIIITEETIITTCLEPLPVLDVFINRRLRNFYTFMRSRFIFQILYRNAELYLTALRSIDRKSEQIESQLH 6zyw.1    ----------------------------------------------------YSQDLHKRARQLLDSLTEQTKMLSTKLS  target    QSTRNEELIELMELEKTIVYFKASLKTNERVIKKLTSSTSNIKKYLEDEDLLEDTLIETQQAIEMADIYGNVLHSMTETF 6zyw.1    KPV-----------------------------------------------------------------------------  target    ASIISNNQNNIMKTLALVTIVMSIPTMVFSAYGMNFKDNEIPLNGEPNAFWLIVFIAFAMSVSLTLYLIHKKWF 6zyw.1    -------------------------------------------------------------------------- ``` | | | | | | | | | | | | | | | | | | | | | | | | | | | | | | | | | | | | | | | | | | | | | | | | | |
|  | 5j1g.1.A | Plectin  *Structure of the spectrin repeats 7 and 8 of the plakin domain of plectin* | 0.02 |  | 27.59 | 0.09 | 211-239 | X-ray | 1.80 | monomer |  | HHblits | 0.31 |
| ``` target    MKQVFLSTTTEFKEIDTLEPGTWINLVNPTQNESLEIANTFDIDIADLRAPLDAEEMSRITIEDEYTLIIVDVPVTEERN 5j1g.1    --------------------------------------------------------------------------------  target    NRTYYVTIPLGIIITEETIITTCLEPLPVLDVFINRRLRNFYTFMRSRFIFQILYRNAELYLTALRSIDRKSEQIESQLH 5j1g.1    --------------------------------------------------------------------------------  target    QSTRNEELIELMELEKTIVYFKASLKTNERVIKKLTSSTSNIKKYLEDEDLLEDTLIETQQAIEMADIYGNVLHSMTETF 5j1g.1    --------------------------------------------------ELELTLGKLEQVRSLSAIYLEKLKTISLV-  target    ASIISNNQNNIMKTLALVTIVMSIPTMVFSAYGMNFKDNEIPLNGEPNAFWLIVFIAFAMSVSLTLYLIHKKWF 5j1g.1    -------------------------------------------------------------------------- ``` | | | | | | | | | | | | | | | | | | | | | | | | | | | | | | | | | | | | | | | | | | | | | | | | | |
|  | 2osz.1.C | Nucleoporin p58/p45  *Structure of Nup58/45 suggests flexible nuclear pore diameter by intermolecular sliding* | 0.01 |  | 13.33 | 0.10 | 132-161 | X-ray | 2.85 | homo-tetramer |  | HHblits | 0.28 |
| ``` target    MKQVFLSTTTEFKEIDTLEPGTWINLVNPTQNESLEIANTFDIDIADLRAPLDAEEMSRITIEDEYTLIIVDVPVTEERN 2osz.1    --------------------------------------------------------------------------------  target    NRTYYVTIPLGIIITEETIITTCLEPLPVLDVFINRRLRNFYTFMRSRFIFQILYRNAELYLTALRSIDRKSEQIESQLH 2osz.1    ---------------------------------------------------DYFRVLVQQFEVQLQQYRQQIEELENHLA  target    QSTRNEELIELMELEKTIVYFKASLKTNERVIKKLTSSTSNIKKYLEDEDLLEDTLIETQQAIEMADIYGNVLHSMTETF 2osz.1    T-------------------------------------------------------------------------------  target    ASIISNNQNNIMKTLALVTIVMSIPTMVFSAYGMNFKDNEIPLNGEPNAFWLIVFIAFAMSVSLTLYLIHKKWF 2osz.1    -------------------------------------------------------------------------- ``` | | | | | | | | | | | | | | | | | | | | | | | | | | | | | | | | | | | | | | | | | | | | | | | | | |
|  | 3t98.1.B | Nucleoporin Nup58/Nup45  *Molecular Architecture of the Transport Channel of the Nuclear Pore Complex: Nup54/Nup58* | 0.02 |  | 13.33 | 0.10 | 132-161 | X-ray | 2.50 | hetero-oligomer |  | HHblits | 0.28 |
| ``` target    MKQVFLSTTTEFKEIDTLEPGTWINLVNPTQNESLEIANTFDIDIADLRAPLDAEEMSRITIEDEYTLIIVDVPVTEERN 3t98.1    --------------------------------------------------------------------------------  target    NRTYYVTIPLGIIITEETIITTCLEPLPVLDVFINRRLRNFYTFMRSRFIFQILYRNAELYLTALRSIDRKSEQIESQLH 3t98.1    ---------------------------------------------------DYFRVLVQQFEVQLQQYRQQIEELENHLA  target    QSTRNEELIELMELEKTIVYFKASLKTNERVIKKLTSSTSNIKKYLEDEDLLEDTLIETQQAIEMADIYGNVLHSMTETF 3t98.1    T-------------------------------------------------------------------------------  target    ASIISNNQNNIMKTLALVTIVMSIPTMVFSAYGMNFKDNEIPLNGEPNAFWLIVFIAFAMSVSLTLYLIHKKWF 3t98.1    -------------------------------------------------------------------------- ``` | | | | | | | | | | | | | | | | | | | | | | | | | | | | | | | | | | | | | | | | | | | | | | | | | |
|  | 2osz.1.D | Nucleoporin p58/p45  *Structure of Nup58/45 suggests flexible nuclear pore diameter by intermolecular sliding* | 0.01 |  | 13.33 | 0.10 | 132-161 | X-ray | 2.85 | homo-tetramer |  | HHblits | 0.28 |
| ``` target    MKQVFLSTTTEFKEIDTLEPGTWINLVNPTQNESLEIANTFDIDIADLRAPLDAEEMSRITIEDEYTLIIVDVPVTEERN 2osz.1    --------------------------------------------------------------------------------  target    NRTYYVTIPLGIIITEETIITTCLEPLPVLDVFINRRLRNFYTFMRSRFIFQILYRNAELYLTALRSIDRKSEQIESQLH 2osz.1    ---------------------------------------------------DYFRVLVQQFEVQLQQYRQQIEELENHLA  target    QSTRNEELIELMELEKTIVYFKASLKTNERVIKKLTSSTSNIKKYLEDEDLLEDTLIETQQAIEMADIYGNVLHSMTETF 2osz.1    T-------------------------------------------------------------------------------  target    ASIISNNQNNIMKTLALVTIVMSIPTMVFSAYGMNFKDNEIPLNGEPNAFWLIVFIAFAMSVSLTLYLIHKKWF 2osz.1    -------------------------------------------------------------------------- ``` | | | | | | | | | | | | | | | | | | | | | | | | | | | | | | | | | | | | | | | | | | | | | | | | | |
|  | 5ijh.1.A | Xenotropic and polytropic retrovirus receptor 1  *Structure of the SPX domain of the human phosphate transporter XPR1 in complex with a sulfate ion* | 0.01 |  | 10.00 | 0.10 | 169-198 | X-ray | 2.43 | monomer |  | HHblits | 0.28 |
| ``` target    MKQVFLSTTTEFKEIDTLEPGTWINLVNPTQNESLEIANTFDIDIADLRAPLDAEEMSRITIEDEYTLIIVDVPVTEERN 5ijh.1    --------------------------------------------------------------------------------  target    NRTYYVTIPLGIIITEETIITTCLEPLPVLDVFINRRLRNFYTFMRSRFIFQILYRNAELYLTALRSIDRKSEQIESQLH 5ijh.1    --------------------------------------------------------------------------------  target    QSTRNEELIELMELEKTIVYFKASLKTNERVIKKLTSSTSNIKKYLEDEDLLEDTLIETQQAIEMADIYGNVLHSMTETF 5ijh.1    --------LAFSEFYLSLILLQNYQNLNFTGFRKILKK------------------------------------------  target    ASIISNNQNNIMKTLALVTIVMSIPTMVFSAYGMNFKDNEIPLNGEPNAFWLIVFIAFAMSVSLTLYLIHKKWF 5ijh.1    -------------------------------------------------------------------------- ``` | | | | | | | | | | | | | | | | | | | | | | | | | | | | | | | | | | | | | | | | | | | | | | | | | |
|  | 3x29.1.A | Claudin-19  *CRYSTAL STRUCTURE of MOUSE CLAUDIN-19 IN COMPLEX with C-TERMINAL FRAGMENT OF CLOSTRIDIUM PERFRINGENS ENTEROTOXIN* | 0.01 |  | 25.93 | 0.09 | 250-276 | X-ray | 3.70 | hetero-oligomer |  | HHblits | 0.35 |
| ``` target    MKQVFLSTTTEFKEIDTLEPGTWINLVNPTQNESLEIANTFDIDIADLRAPLDAEEMSRITIEDEYTLIIVDVPVTEERN 3x29.1    --------------------------------------------------------------------------------  target    NRTYYVTIPLGIIITEETIITTCLEPLPVLDVFINRRLRNFYTFMRSRFIFQILYRNAELYLTALRSIDRKSEQIESQLH 3x29.1    --------------------------------------------------------------------------------  target    QSTRNEELIELMELEKTIVYFKASLKTNERVIKKLTSSTSNIKKYLEDEDLLEDTLIETQQAIEMADIYGNVLHSMTETF 3x29.1    --------------------------------------------------------------------------------  target    ASIISNNQNNIMKTLALVTIVMSIPTMVFSAYGMNFKDNEIPLNGEPNAFWLIVFIAFAMSVSLTLYLIHKKWF 3x29.1    ---------QSARALMVVAVLLGFVAMVLSVVGMKA-------------------------------------- ``` | | | | | | | | | | | | | | | | | | | | | | | | | | | | | | | | | | | | | | | | | | | | | | | | | |
|  | 5ijn.1.H | Nuclear pore glycoprotein p62  *Composite structure of the inner ring of the human nuclear pore complex (32 copies of Nup205)* | 0.02 |  | 13.79 | 0.09 | 169-197 | EM | 0.00 | hetero-6-4-4-4-4-4-… |  | HHblits | 0.29 |
| ``` target    MKQVFLSTTTEFKEIDTLEPGTWINLVNPTQNESLEIANTFDIDIADLRAPLDAEEMSRITIEDEYTLIIVDVPVTEERN 5ijn.1    --------------------------------------------------------------------------------  target    NRTYYVTIPLGIIITEETIITTCLEPLPVLDVFINRRLRNFYTFMRSRFIFQILYRNAELYLTALRSIDRKSEQIESQLH 5ijn.1    --------------------------------------------------------------------------------  target    QSTRNEELIELMELEKTIVYFKASLKTNERVIKKLTSSTSNIKKYLEDEDLLEDTLIETQQAIEMADIYGNVLHSMTETF 5ijn.1    --------EKITSLHREVEKVKLDQKRLDQELDFILS-------------------------------------------  target    ASIISNNQNNIMKTLALVTIVMSIPTMVFSAYGMNFKDNEIPLNGEPNAFWLIVFIAFAMSVSLTLYLIHKKWF 5ijn.1    -------------------------------------------------------------------------- ``` | | | | | | | | | | | | | | | | | | | | | | | | | | | | | | | | | | | | | | | | | | | | | | | | | |
|  | 5ijn.1.G | NUCLEAR PORE COMPLEX PROTEIN NUP58  *Composite structure of the inner ring of the human nuclear pore complex (32 copies of Nup205)* | 0.02 |  | 13.79 | 0.09 | 133-161 | EM | 0.00 | hetero-6-4-4-4-4-4-… |  | HHblits | 0.29 |
| ``` target    MKQVFLSTTTEFKEIDTLEPGTWINLVNPTQNESLEIANTFDIDIADLRAPLDAEEMSRITIEDEYTLIIVDVPVTEERN 5ijn.1    --------------------------------------------------------------------------------  target    NRTYYVTIPLGIIITEETIITTCLEPLPVLDVFINRRLRNFYTFMRSRFIFQILYRNAELYLTALRSIDRKSEQIESQLH 5ijn.1    ----------------------------------------------------YFRILVQQFEVQLQQYRQQIEELENHLA  target    QSTRNEELIELMELEKTIVYFKASLKTNERVIKKLTSSTSNIKKYLEDEDLLEDTLIETQQAIEMADIYGNVLHSMTETF 5ijn.1    T-------------------------------------------------------------------------------  target    ASIISNNQNNIMKTLALVTIVMSIPTMVFSAYGMNFKDNEIPLNGEPNAFWLIVFIAFAMSVSLTLYLIHKKWF 5ijn.1    -------------------------------------------------------------------------- ``` | | | | | | | | | | | | | | | | | | | | | | | | | | | | | | | | | | | | | | | | | | | | | | | | | |
|  | 3dl8.1.B | Preprotein translocase subunit secY  *Structure of the complex of aquifex aeolicus SecYEG and bacillus subtilis SecA* | 0.02 |  | 20.69 | 0.09 | 247-275 | X-ray | 7.50 | hetero-oligomer |  | HHblits | 0.29 |
| ``` target    MKQVFLSTTTEFKEIDTLEPGTWINLVNPTQNESLEIANTFDIDIADLRAPLDAEEMSRITIEDEYTLIIVDVPVTEERN 3dl8.1    --------------------------------------------------------------------------------  target    NRTYYVTIPLGIIITEETIITTCLEPLPVLDVFINRRLRNFYTFMRSRFIFQILYRNAELYLTALRSIDRKSEQIESQLH 3dl8.1    --------------------------------------------------------------------------------  target    QSTRNEELIELMELEKTIVYFKASLKTNERVIKKLTSSTSNIKKYLEDEDLLEDTLIETQQAIEMADIYGNVLHSMTETF 3dl8.1    --------------------------------------------------------------------------------  target    ASIISNNQNNIMKTLALVTIVMSIPTMVFSAYGMNFKDNEIPLNGEPNAFWLIVFIAFAMSVSLTLYLIHKKWF 3dl8.1    ------KINEYTKYLTLFVATVQSLGIAFWIRGQV--------------------------------------- ``` | | | | | | | | | | | | | | | | | | | | | | | | | | | | | | | | | | | | | | | | | | | | | | | | | |
|  | 5kte.1.A | Divalent metal cation transporter MntH  *Crystal structure of Deinococcus radiodurans MntH, an Nramp-family transition metal transporter* | 0.01 |  | 6.45 | 0.10 | 248-278 | X-ray | 3.94 | hetero-1-1-1-mer |  | HHblits | 0.23 |
| ``` target    MKQVFLSTTTEFKEIDTLEPGTWINLVNPTQNESLEIANTFDIDIADLRAPLDAEEMSRITIEDEYTLIIVDVPVTEERN 5kte.1    --------------------------------------------------------------------------------  target    NRTYYVTIPLGIIITEETIITTCLEPLPVLDVFINRRLRNFYTFMRSRFIFQILYRNAELYLTALRSIDRKSEQIESQLH 5kte.1    --------------------------------------------------------------------------------  target    QSTRNEELIELMELEKTIVYFKASLKTNERVIKKLTSSTSNIKKYLEDEDLLEDTLIETQQAIEMADIYGNVLHSMTETF 5kte.1    --------------------------------------------------------------------------------  target    ASIISNNQNNIMKTLALVTIVMSIPTMVFSAYGMNFKDNEIPLNGEPNAFWLIVFIAFAMSVSLTLYLIHKKWF 5kte.1    -------PSSVLILSQVILCFGVPFALVPLLLFTAHHD------------------------------------ ``` | | | | | | | | | | | | | | | | | | | | | | | | | | | | | | | | | | | | | | | | | | | | | | | | | |
|  | 6d9w.1.A | Divalent metal cation transporter MntH  *Crystal structure of Deinococcus radiodurans MntH, an Nramp-family transition metal transporter, in the inward-open apo state* | 0.01 |  | 6.45 | 0.10 | 248-278 | X-ray | 3.94 | hetero-1-1-1-mer | 1 x OS | HHblits | 0.23 |
| ``` target    MKQVFLSTTTEFKEIDTLEPGTWINLVNPTQNESLEIANTFDIDIADLRAPLDAEEMSRITIEDEYTLIIVDVPVTEERN 6d9w.1    --------------------------------------------------------------------------------  target    NRTYYVTIPLGIIITEETIITTCLEPLPVLDVFINRRLRNFYTFMRSRFIFQILYRNAELYLTALRSIDRKSEQIESQLH 6d9w.1    --------------------------------------------------------------------------------  target    QSTRNEELIELMELEKTIVYFKASLKTNERVIKKLTSSTSNIKKYLEDEDLLEDTLIETQQAIEMADIYGNVLHSMTETF 6d9w.1    --------------------------------------------------------------------------------  target    ASIISNNQNNIMKTLALVTIVMSIPTMVFSAYGMNFKDNEIPLNGEPNAFWLIVFIAFAMSVSLTLYLIHKKWF 6d9w.1    -------PSSVLILSQVILCFGVPFALVPLLLFTAHHD------------------------------------ ``` | | | | | | | | | | | | | | | | | | | | | | | | | | | | | | | | | | | | | | | | | | | | | | | | | |
|  | 6btm.1.C | Alternative Complex III subunit C  *Structure of Alternative Complex III from Flavobacterium johnsoniae (Wild Type)* | 0.01 |  | 13.79 | 0.09 | 247-275 | EM | 3.40 | hetero-1-1-1-1-1-1-… | 6 x HEC, 1 x F3S, 1 x SF4, 2 x E87 | HHblits | 0.28 |
| ``` target    MKQVFLSTTTEFKEIDTLEPGTWINLVNPTQNESLEIANTFDIDIADLRAPLDAEEMSRITIEDEYTLIIVDVPVTEERN 6btm.1    --------------------------------------------------------------------------------  target    NRTYYVTIPLGIIITEETIITTCLEPLPVLDVFINRRLRNFYTFMRSRFIFQILYRNAELYLTALRSIDRKSEQIESQLH 6btm.1    --------------------------------------------------------------------------------  target    QSTRNEELIELMELEKTIVYFKASLKTNERVIKKLTSSTSNIKKYLEDEDLLEDTLIETQQAIEMADIYGNVLHSMTETF 6btm.1    --------------------------------------------------------------------------------  target    ASIISNNQNNIMKTLALVTIVMSI--PTMVFSAYGMNFKDNEIPLNGEPNAFWLIVFIAFAMSVSLTLYLIHKKWF 6btm.1    ------RFEEVSLVLAGLATPLVLSVHTIVSMDFATS--------------------------------------- ``` | | | | | | | | | | | | | | | | | | | | | | | | | | | | | | | | | | | | | | | | | | | | | | | | | |
|  | 5j0i.1.A | Designed protein 2L6HC3\_12  *De novo design of protein homo-oligomers with modular hydrogen bond network-mediated specificity* | 0.01 |  | 22.22 | 0.09 | 172-198 | X-ray | 2.20 | homo-trimer |  | HHblits | 0.33 |
| ``` target    MKQVFLSTTTEFKEIDTLEPGTWINLVNPTQNESLEIANTFDIDIADLRAPLDAEEMSRITIEDEYTLIIVDVPVTEERN 5j0i.1    --------------------------------------------------------------------------------  target    NRTYYVTIPLGIIITEETIITTCLEPLPVLDVFINRRLRNFYTFMRSRFIFQILYRNAELYLTALRSIDRKSEQIESQLH 5j0i.1    --------------------------------------------------------------------------------  target    QSTRNEELIELMELEKTIVYFKASLKTNERVIKKLTSSTSNIKKYLEDEDLLEDTLIETQQAIEMADIYGNVLHSMTETF 5j0i.1    -----------YELRRALEELEKALQELREMLRKLKES------------------------------------------  target    ASIISNNQNNIMKTLALVTIVMSIPTMVFSAYGMNFKDNEIPLNGEPNAFWLIVFIAFAMSVSLTLYLIHKKWF 5j0i.1    -------------------------------------------------------------------------- ``` | | | | | | | | | | | | | | | | | | | | | | | | | | | | | | | | | | | | | | | | | | | | | | | | | |
|  | 4h63.1.B | Mediator of RNA polymerase II transcription subunit 8  *Structure of the Schizosaccharomyces pombe Mediator head module* | 0.01 |  | 13.79 | 0.09 | 166-194 | X-ray | 3.40 | hetero-oligomer |  | HHblits | 0.27 |
| ``` target    MKQVFLSTTTEFKEIDTLEPGTWINLVNPTQNESLEIANTFDIDIADLRAPLDAEEMSRITIEDEYTLIIVDVPVTEERN 4h63.1    --------------------------------------------------------------------------------  target    NRTYYVTIPLGIIITEETIITTCLEPLPVLDVFINRRLRNFYTFMRSRFIFQILYRNAELYLTALRSIDRKSEQIESQLH 4h63.1    --------------------------------------------------------------------------------  target    QSTRNEELIELMELEKTIVYFKASLKTNERVIKKLTSSTSNIKKYLEDEDLLEDTLIETQQAIEMADIYGNVLHSMTETF 4h63.1    -----KTVESLEAIRHRIAQIVQSLTHFLAILHQ----------------------------------------------  target    ASIISNNQNNIMKTLALVTIVMSIPTMVFSAYGMNFKDNEIPLNGEPNAFWLIVFIAFAMSVSLTLYLIHKKWF 4h63.1    -------------------------------------------------------------------------- ``` | | | | | | | | | | | | | | | | | | | | | | | | | | | | | | | | | | | | | | | | | | | | | | | | | |
|  | 5n9j.1.J | Mediator of RNA polymerase II transcription subunit 8  *Core Mediator of transcriptional regulation* | 0.01 |  | 13.79 | 0.09 | 166-194 | X-ray | 3.40 | hetero-oligomer |  | HHblits | 0.27 |
| ``` target    MKQVFLSTTTEFKEIDTLEPGTWINLVNPTQNESLEIANTFDIDIADLRAPLDAEEMSRITIEDEYTLIIVDVPVTEERN 5n9j.1    --------------------------------------------------------------------------------  target    NRTYYVTIPLGIIITEETIITTCLEPLPVLDVFINRRLRNFYTFMRSRFIFQILYRNAELYLTALRSIDRKSEQIESQLH 5n9j.1    --------------------------------------------------------------------------------  target    QSTRNEELIELMELEKTIVYFKASLKTNERVIKKLTSSTSNIKKYLEDEDLLEDTLIETQQAIEMADIYGNVLHSMTETF 5n9j.1    -----KTVESLEAIRHRIAQIVQSLTHFLAILHQ----------------------------------------------  target    ASIISNNQNNIMKTLALVTIVMSIPTMVFSAYGMNFKDNEIPLNGEPNAFWLIVFIAFAMSVSLTLYLIHKKWF 5n9j.1    -------------------------------------------------------------------------- ``` | | | | | | | | | | | | | | | | | | | | | | | | | | | | | | | | | | | | | | | | | | | | | | | | | |
|  | 5u0p.1.C | Mediator complex subunit 8  *Cryo-EM structure of the transcriptional Mediator* | 0.02 |  | 13.79 | 0.09 | 166-194 | EM | 0.00 | hetero-1-1-1-1-1-1-… |  | HHblits | 0.27 |
| ``` target    MKQVFLSTTTEFKEIDTLEPGTWINLVNPTQNESLEIANTFDIDIADLRAPLDAEEMSRITIEDEYTLIIVDVPVTEERN 5u0p.1    --------------------------------------------------------------------------------  target    NRTYYVTIPLGIIITEETIITTCLEPLPVLDVFINRRLRNFYTFMRSRFIFQILYRNAELYLTALRSIDRKSEQIESQLH 5u0p.1    --------------------------------------------------------------------------------  target    QSTRNEELIELMELEKTIVYFKASLKTNERVIKKLTSSTSNIKKYLEDEDLLEDTLIETQQAIEMADIYGNVLHSMTETF 5u0p.1    -----KTVESLEAIRHRIAQIVQSLTHFLAILHQ----------------------------------------------  target    ASIISNNQNNIMKTLALVTIVMSIPTMVFSAYGMNFKDNEIPLNGEPNAFWLIVFIAFAMSVSLTLYLIHKKWF 5u0p.1    -------------------------------------------------------------------------- ``` | | | | | | | | | | | | | | | | | | | | | | | | | | | | | | | | | | | | | | | | | | | | | | | | | |
|  | 5u0s.1.B | Mediator complex subunit 8  *Cryo-EM structure of the Mediator-RNAPII complex* | 0.02 |  | 13.79 | 0.09 | 166-194 | EM | 0.00 | hetero-1-1-1-1-1-1-… |  | HHblits | 0.27 |
| ``` target    MKQVFLSTTTEFKEIDTLEPGTWINLVNPTQNESLEIANTFDIDIADLRAPLDAEEMSRITIEDEYTLIIVDVPVTEERN 5u0s.1    --------------------------------------------------------------------------------  target    NRTYYVTIPLGIIITEETIITTCLEPLPVLDVFINRRLRNFYTFMRSRFIFQILYRNAELYLTALRSIDRKSEQIESQLH 5u0s.1    --------------------------------------------------------------------------------  target    QSTRNEELIELMELEKTIVYFKASLKTNERVIKKLTSSTSNIKKYLEDEDLLEDTLIETQQAIEMADIYGNVLHSMTETF 5u0s.1    -----KTVESLEAIRHRIAQIVQSLTHFLAILHQ----------------------------------------------  target    ASIISNNQNNIMKTLALVTIVMSIPTMVFSAYGMNFKDNEIPLNGEPNAFWLIVFIAFAMSVSLTLYLIHKKWF 5u0s.1    -------------------------------------------------------------------------- ``` | | | | | | | | | | | | | | | | | | | | | | | | | | | | | | | | | | | | | | | | | | | | | | | | | |
|  | 5v2s.1.A | Envelope glycoprotein B  *Crystal structure of glycoprotein B from Herpes Simplex Virus type I* | 0.01 |  | 6.67 | 0.10 | 281-310 | X-ray | 3.60 | homo-trimer | 3 x NAG, 6 x NAG-NAG | HHblits | 0.24 |
| ``` target    MKQVFLSTTTEFKEIDTLEPGTWINLVNPTQNESLEIANTFDIDIADLRAPLDAEEMSRITIEDEYTLIIVDVPVTEERN 5v2s.1    --------------------------------------------------------------------------------  target    NRTYYVTIPLGIIITEETIITTCLEPLPVLDVFINRRLRNFYTFMRSRFIFQILYRNAELYLTALRSIDRKSEQIESQLH 5v2s.1    --------------------------------------------------------------------------------  target    QSTRNEELIELMELEKTIVYFKASLKTNERVIKKLTSSTSNIKKYLEDEDLLEDTLIETQQAIEMADIYGNVLHSMTETF 5v2s.1    --------------------------------------------------------------------------------  target    ASIISNNQNNIMKTLALVTIVMSIPTMVFSAYGMNFKDNEIPLNGEPNAFWLIVFIAFAMSVSLTLYLIHKKWF 5v2s.1    ----------------------------------------SSFMSNPFGALAVGLLVLAGLAAAFFAFRY---- ``` | | | | | | | | | | | | | | | | | | | | | | | | | | | | | | | | | | | | | | | | | | | | | | | | | |
|  | 6bm8.1.A | Envelope glycoprotein B  *Crystal structure of glycoprotein B from Herpes Simplex Virus type I* | 0.00 |  | 6.67 | 0.10 | 281-310 | X-ray | 4.10 | homo-trimer | 6 x NAG, 3 x NAG-NAG-BMA, 3 x NAG-NAG | HHblits | 0.24 |
| ``` target    MKQVFLSTTTEFKEIDTLEPGTWINLVNPTQNESLEIANTFDIDIADLRAPLDAEEMSRITIEDEYTLIIVDVPVTEERN 6bm8.1    --------------------------------------------------------------------------------  target    NRTYYVTIPLGIIITEETIITTCLEPLPVLDVFINRRLRNFYTFMRSRFIFQILYRNAELYLTALRSIDRKSEQIESQLH 6bm8.1    --------------------------------------------------------------------------------  target    QSTRNEELIELMELEKTIVYFKASLKTNERVIKKLTSSTSNIKKYLEDEDLLEDTLIETQQAIEMADIYGNVLHSMTETF 6bm8.1    --------------------------------------------------------------------------------  target    ASIISNNQNNIMKTLALVTIVMSIPTMVFSAYGMNFKDNEIPLNGEPNAFWLIVFIAFAMSVSLTLYLIHKKWF 6bm8.1    ----------------------------------------SSFMSNPFGALAVGLLVLAGLAAAFFAFRY---- ``` | | | | | | | | | | | | | | | | | | | | | | | | | | | | | | | | | | | | | | | | | | | | | | | | | |
|  | 6akg.1.A | Claudin-3  *Crystal structure of mouse claudin-3 P134G mutant in complex with C-terminal fragment of Clostridium perfringens enterotoxin* | 0.01 |  | 14.29 | 0.09 | 249-276 | X-ray | 4.30 | hetero-1-1-mer |  | HHblits | 0.29 |
| ``` target    MKQVFLSTTTEFKEIDTLEPGTWINLVNPTQNESLEIANTFDIDIADLRAPLDAEEMSRITIEDEYTLIIVDVPVTEERN 6akg.1    --------------------------------------------------------------------------------  target    NRTYYVTIPLGIIITEETIITTCLEPLPVLDVFINRRLRNFYTFMRSRFIFQILYRNAELYLTALRSIDRKSEQIESQLH 6akg.1    --------------------------------------------------------------------------------  target    QSTRNEELIELMELEKTIVYFKASLKTNERVIKKLTSSTSNIKKYLEDEDLLEDTLIETQQAIEMADIYGNVLHSMTETF 6akg.1    --------------------------------------------------------------------------------  target    ASIISNNQNNIMKTLALVTIVMSIPTMVFSAYGMNFKDNEIPLNGEPNAFWLIVFIAFAMSVSLTLYLIHKKWF 6akg.1    --------LQAARALIVVSILLAAFGLLVALVGAQA-------------------------------------- ``` | | | | | | | | | | | | | | | | | | | | | | | | | | | | | | | | | | | | | | | | | | | | | | | | | |
|  | 4jo7.1.C | Nucleoporin p58/p45  *Crystal structure of the human Nup49CCS2+3\* Nup57CCS3\* complex with 2:2 stoichiometry* | 0.01 |  | 14.29 | 0.09 | 134-161 | X-ray | 1.75 | hetero-oligomer |  | HHblits | 0.29 |
| ``` target    MKQVFLSTTTEFKEIDTLEPGTWINLVNPTQNESLEIANTFDIDIADLRAPLDAEEMSRITIEDEYTLIIVDVPVTEERN 4jo7.1    --------------------------------------------------------------------------------  target    NRTYYVTIPLGIIITEETIITTCLEPLPVLDVFINRRLRNFYTFMRSRFIFQILYRNAELYLTALRSIDRKSEQIESQLH 4jo7.1    -----------------------------------------------------FRILVQQFEVQLQQYRQQIEELENHLA  target    QSTRNEELIELMELEKTIVYFKASLKTNERVIKKLTSSTSNIKKYLEDEDLLEDTLIETQQAIEMADIYGNVLHSMTETF 4jo7.1    T-------------------------------------------------------------------------------  target    ASIISNNQNNIMKTLALVTIVMSIPTMVFSAYGMNFKDNEIPLNGEPNAFWLIVFIAFAMSVSLTLYLIHKKWF 4jo7.1    -------------------------------------------------------------------------- ``` | | | | | | | | | | | | | | | | | | | | | | | | | | | | | | | | | | | | | | | | | | | | | | | | | |
|  | 4jo7.1.D | Nucleoporin p58/p45  *Crystal structure of the human Nup49CCS2+3\* Nup57CCS3\* complex with 2:2 stoichiometry* | 0.02 |  | 14.29 | 0.09 | 134-161 | X-ray | 1.75 | hetero-oligomer |  | HHblits | 0.29 |
| ``` target    MKQVFLSTTTEFKEIDTLEPGTWINLVNPTQNESLEIANTFDIDIADLRAPLDAEEMSRITIEDEYTLIIVDVPVTEERN 4jo7.1    --------------------------------------------------------------------------------  target    NRTYYVTIPLGIIITEETIITTCLEPLPVLDVFINRRLRNFYTFMRSRFIFQILYRNAELYLTALRSIDRKSEQIESQLH 4jo7.1    -----------------------------------------------------FRILVQQFEVQLQQYRQQIEELENHLA  target    QSTRNEELIELMELEKTIVYFKASLKTNERVIKKLTSSTSNIKKYLEDEDLLEDTLIETQQAIEMADIYGNVLHSMTETF 4jo7.1    T-------------------------------------------------------------------------------  target    ASIISNNQNNIMKTLALVTIVMSIPTMVFSAYGMNFKDNEIPLNGEPNAFWLIVFIAFAMSVSLTLYLIHKKWF 4jo7.1    -------------------------------------------------------------------------- ``` | | | | | | | | | | | | | | | | | | | | | | | | | | | | | | | | | | | | | | | | | | | | | | | | | |
|  | 4jo7.2.A | Nucleoporin p58/p45  *Crystal structure of the human Nup49CCS2+3\* Nup57CCS3\* complex with 2:2 stoichiometry* | 0.02 |  | 14.29 | 0.09 | 134-161 | X-ray | 1.75 | hetero-oligomer |  | HHblits | 0.29 |
| ``` target    MKQVFLSTTTEFKEIDTLEPGTWINLVNPTQNESLEIANTFDIDIADLRAPLDAEEMSRITIEDEYTLIIVDVPVTEERN 4jo7.2    --------------------------------------------------------------------------------  target    NRTYYVTIPLGIIITEETIITTCLEPLPVLDVFINRRLRNFYTFMRSRFIFQILYRNAELYLTALRSIDRKSEQIESQLH 4jo7.2    -----------------------------------------------------FRILVQQFEVQLQQYRQQIEELENHLA  target    QSTRNEELIELMELEKTIVYFKASLKTNERVIKKLTSSTSNIKKYLEDEDLLEDTLIETQQAIEMADIYGNVLHSMTETF 4jo7.2    T-------------------------------------------------------------------------------  target    ASIISNNQNNIMKTLALVTIVMSIPTMVFSAYGMNFKDNEIPLNGEPNAFWLIVFIAFAMSVSLTLYLIHKKWF 4jo7.2    -------------------------------------------------------------------------- ``` | | | | | | | | | | | | | | | | | | | | | | | | | | | | | | | | | | | | | | | | | | | | | | | | | |
|  | 4jo7.2.C | Nucleoporin p58/p45  *Crystal structure of the human Nup49CCS2+3\* Nup57CCS3\* complex with 2:2 stoichiometry* | 0.01 |  | 14.29 | 0.09 | 134-161 | X-ray | 1.75 | hetero-oligomer |  | HHblits | 0.29 |
| ``` target    MKQVFLSTTTEFKEIDTLEPGTWINLVNPTQNESLEIANTFDIDIADLRAPLDAEEMSRITIEDEYTLIIVDVPVTEERN 4jo7.2    --------------------------------------------------------------------------------  target    NRTYYVTIPLGIIITEETIITTCLEPLPVLDVFINRRLRNFYTFMRSRFIFQILYRNAELYLTALRSIDRKSEQIESQLH 4jo7.2    -----------------------------------------------------FRILVQQFEVQLQQYRQQIEELENHLA  target    QSTRNEELIELMELEKTIVYFKASLKTNERVIKKLTSSTSNIKKYLEDEDLLEDTLIETQQAIEMADIYGNVLHSMTETF 4jo7.2    T-------------------------------------------------------------------------------  target    ASIISNNQNNIMKTLALVTIVMSIPTMVFSAYGMNFKDNEIPLNGEPNAFWLIVFIAFAMSVSLTLYLIHKKWF 4jo7.2    -------------------------------------------------------------------------- ``` | | | | | | | | | | | | | | | | | | | | | | | | | | | | | | | | | | | | | | | | | | | | | | | | | |
|  | 4jo9.1.B | Nucleoporin p58/p45  *Crystal structure of the human Nup49CCS2+3\* Nup57CCS3\* complex 1:2 stoichiometry* | 0.02 |  | 14.29 | 0.09 | 134-161 | X-ray | 2.50 | hetero-oligomer |  | HHblits | 0.29 |
| ``` target    MKQVFLSTTTEFKEIDTLEPGTWINLVNPTQNESLEIANTFDIDIADLRAPLDAEEMSRITIEDEYTLIIVDVPVTEERN 4jo9.1    --------------------------------------------------------------------------------  target    NRTYYVTIPLGIIITEETIITTCLEPLPVLDVFINRRLRNFYTFMRSRFIFQILYRNAELYLTALRSIDRKSEQIESQLH 4jo9.1    -----------------------------------------------------FRILVQQFEVQLQQYRQQIEELENHLA  target    QSTRNEELIELMELEKTIVYFKASLKTNERVIKKLTSSTSNIKKYLEDEDLLEDTLIETQQAIEMADIYGNVLHSMTETF 4jo9.1    T-------------------------------------------------------------------------------  target    ASIISNNQNNIMKTLALVTIVMSIPTMVFSAYGMNFKDNEIPLNGEPNAFWLIVFIAFAMSVSLTLYLIHKKWF 4jo9.1    -------------------------------------------------------------------------- ``` | | | | | | | | | | | | | | | | | | | | | | | | | | | | | | | | | | | | | | | | | | | | | | | | | |
|  | 4jq5.1.B | Nucleoporin p58/p45  *Crystal structure of the human Nup49CCS2+3\* coiled-coil segment* | 0.01 |  | 14.29 | 0.09 | 134-161 | X-ray | 2.19 | homo-tetramer |  | HHblits | 0.29 |
| ``` target    MKQVFLSTTTEFKEIDTLEPGTWINLVNPTQNESLEIANTFDIDIADLRAPLDAEEMSRITIEDEYTLIIVDVPVTEERN 4jq5.1    --------------------------------------------------------------------------------  target    NRTYYVTIPLGIIITEETIITTCLEPLPVLDVFINRRLRNFYTFMRSRFIFQILYRNAELYLTALRSIDRKSEQIESQLH 4jq5.1    -----------------------------------------------------FRILVQQFEVQLQQYRQQIEELENHLA  target    QSTRNEELIELMELEKTIVYFKASLKTNERVIKKLTSSTSNIKKYLEDEDLLEDTLIETQQAIEMADIYGNVLHSMTETF 4jq5.1    T-------------------------------------------------------------------------------  target    ASIISNNQNNIMKTLALVTIVMSIPTMVFSAYGMNFKDNEIPLNGEPNAFWLIVFIAFAMSVSLTLYLIHKKWF 4jq5.1    -------------------------------------------------------------------------- ``` | | | | | | | | | | | | | | | | | | | | | | | | | | | | | | | | | | | | | | | | | | | | | | | | | |
|  | 4jq5.1.A | Nucleoporin p58/p45  *Crystal structure of the human Nup49CCS2+3\* coiled-coil segment* | 0.01 |  | 14.29 | 0.09 | 134-161 | X-ray | 2.19 | homo-tetramer |  | HHblits | 0.29 |
| ``` target    MKQVFLSTTTEFKEIDTLEPGTWINLVNPTQNESLEIANTFDIDIADLRAPLDAEEMSRITIEDEYTLIIVDVPVTEERN 4jq5.1    --------------------------------------------------------------------------------  target    NRTYYVTIPLGIIITEETIITTCLEPLPVLDVFINRRLRNFYTFMRSRFIFQILYRNAELYLTALRSIDRKSEQIESQLH 4jq5.1    -----------------------------------------------------FRILVQQFEVQLQQYRQQIEELENHLA  target    QSTRNEELIELMELEKTIVYFKASLKTNERVIKKLTSSTSNIKKYLEDEDLLEDTLIETQQAIEMADIYGNVLHSMTETF 4jq5.1    T-------------------------------------------------------------------------------  target    ASIISNNQNNIMKTLALVTIVMSIPTMVFSAYGMNFKDNEIPLNGEPNAFWLIVFIAFAMSVSLTLYLIHKKWF 4jq5.1    -------------------------------------------------------------------------- ``` | | | | | | | | | | | | | | | | | | | | | | | | | | | | | | | | | | | | | | | | | | | | | | | | | |
|  | 4jq5.1.C | Nucleoporin p58/p45  *Crystal structure of the human Nup49CCS2+3\* coiled-coil segment* | 0.01 |  | 14.29 | 0.09 | 134-161 | X-ray | 2.19 | homo-tetramer |  | HHblits | 0.29 |
| ``` target    MKQVFLSTTTEFKEIDTLEPGTWINLVNPTQNESLEIANTFDIDIADLRAPLDAEEMSRITIEDEYTLIIVDVPVTEERN 4jq5.1    --------------------------------------------------------------------------------  target    NRTYYVTIPLGIIITEETIITTCLEPLPVLDVFINRRLRNFYTFMRSRFIFQILYRNAELYLTALRSIDRKSEQIESQLH 4jq5.1    -----------------------------------------------------FRILVQQFEVQLQQYRQQIEELENHLA  target    QSTRNEELIELMELEKTIVYFKASLKTNERVIKKLTSSTSNIKKYLEDEDLLEDTLIETQQAIEMADIYGNVLHSMTETF 4jq5.1    T-------------------------------------------------------------------------------  target    ASIISNNQNNIMKTLALVTIVMSIPTMVFSAYGMNFKDNEIPLNGEPNAFWLIVFIAFAMSVSLTLYLIHKKWF 4jq5.1    -------------------------------------------------------------------------- ``` | | | | | | | | | | | | | | | | | | | | | | | | | | | | | | | | | | | | | | | | | | | | | | | | | |
|  | 4jq5.2.A | Nucleoporin p58/p45  *Crystal structure of the human Nup49CCS2+3\* coiled-coil segment* | 0.01 |  | 14.29 | 0.09 | 134-161 | X-ray | 2.19 | homo-tetramer |  | HHblits | 0.29 |
| ``` target    MKQVFLSTTTEFKEIDTLEPGTWINLVNPTQNESLEIANTFDIDIADLRAPLDAEEMSRITIEDEYTLIIVDVPVTEERN 4jq5.2    --------------------------------------------------------------------------------  target    NRTYYVTIPLGIIITEETIITTCLEPLPVLDVFINRRLRNFYTFMRSRFIFQILYRNAELYLTALRSIDRKSEQIESQLH 4jq5.2    -----------------------------------------------------FRILVQQFEVQLQQYRQQIEELENHLA  target    QSTRNEELIELMELEKTIVYFKASLKTNERVIKKLTSSTSNIKKYLEDEDLLEDTLIETQQAIEMADIYGNVLHSMTETF 4jq5.2    T-------------------------------------------------------------------------------  target    ASIISNNQNNIMKTLALVTIVMSIPTMVFSAYGMNFKDNEIPLNGEPNAFWLIVFIAFAMSVSLTLYLIHKKWF 4jq5.2    -------------------------------------------------------------------------- ``` | | | | | | | | | | | | | | | | | | | | | | | | | | | | | | | | | | | | | | | | | | | | | | | | | |
|  | 4jq5.2.B | Nucleoporin p58/p45  *Crystal structure of the human Nup49CCS2+3\* coiled-coil segment* | 0.01 |  | 14.29 | 0.09 | 134-161 | X-ray | 2.19 | homo-tetramer |  | HHblits | 0.29 |
| ``` target    MKQVFLSTTTEFKEIDTLEPGTWINLVNPTQNESLEIANTFDIDIADLRAPLDAEEMSRITIEDEYTLIIVDVPVTEERN 4jq5.2    --------------------------------------------------------------------------------  target    NRTYYVTIPLGIIITEETIITTCLEPLPVLDVFINRRLRNFYTFMRSRFIFQILYRNAELYLTALRSIDRKSEQIESQLH 4jq5.2    -----------------------------------------------------FRILVQQFEVQLQQYRQQIEELENHLA  target    QSTRNEELIELMELEKTIVYFKASLKTNERVIKKLTSSTSNIKKYLEDEDLLEDTLIETQQAIEMADIYGNVLHSMTETF 4jq5.2    T-------------------------------------------------------------------------------  target    ASIISNNQNNIMKTLALVTIVMSIPTMVFSAYGMNFKDNEIPLNGEPNAFWLIVFIAFAMSVSLTLYLIHKKWF 4jq5.2    -------------------------------------------------------------------------- ``` | | | | | | | | | | | | | | | | | | | | | | | | | | | | | | | | | | | | | | | | | | | | | | | | | |
|  | 4jq5.2.C | Nucleoporin p58/p45  *Crystal structure of the human Nup49CCS2+3\* coiled-coil segment* | 0.01 |  | 14.29 | 0.09 | 134-161 | X-ray | 2.19 | homo-tetramer |  | HHblits | 0.29 |
| ``` target    MKQVFLSTTTEFKEIDTLEPGTWINLVNPTQNESLEIANTFDIDIADLRAPLDAEEMSRITIEDEYTLIIVDVPVTEERN 4jq5.2    --------------------------------------------------------------------------------  target    NRTYYVTIPLGIIITEETIITTCLEPLPVLDVFINRRLRNFYTFMRSRFIFQILYRNAELYLTALRSIDRKSEQIESQLH 4jq5.2    -----------------------------------------------------FRILVQQFEVQLQQYRQQIEELENHLA  target    QSTRNEELIELMELEKTIVYFKASLKTNERVIKKLTSSTSNIKKYLEDEDLLEDTLIETQQAIEMADIYGNVLHSMTETF 4jq5.2    T-------------------------------------------------------------------------------  target    ASIISNNQNNIMKTLALVTIVMSIPTMVFSAYGMNFKDNEIPLNGEPNAFWLIVFIAFAMSVSLTLYLIHKKWF 4jq5.2    -------------------------------------------------------------------------- ``` | | | | | | | | | | | | | | | | | | | | | | | | | | | | | | | | | | | | | | | | | | | | | | | | | |
|  | 4jq5.3.A | Nucleoporin p58/p45  *Crystal structure of the human Nup49CCS2+3\* coiled-coil segment* | 0.01 |  | 14.29 | 0.09 | 134-161 | X-ray | 2.19 | homo-tetramer |  | HHblits | 0.29 |
| ``` target    MKQVFLSTTTEFKEIDTLEPGTWINLVNPTQNESLEIANTFDIDIADLRAPLDAEEMSRITIEDEYTLIIVDVPVTEERN 4jq5.3    --------------------------------------------------------------------------------  target    NRTYYVTIPLGIIITEETIITTCLEPLPVLDVFINRRLRNFYTFMRSRFIFQILYRNAELYLTALRSIDRKSEQIESQLH 4jq5.3    -----------------------------------------------------FRILVQQFEVQLQQYRQQIEELENHLA  target    QSTRNEELIELMELEKTIVYFKASLKTNERVIKKLTSSTSNIKKYLEDEDLLEDTLIETQQAIEMADIYGNVLHSMTETF 4jq5.3    T-------------------------------------------------------------------------------  target    ASIISNNQNNIMKTLALVTIVMSIPTMVFSAYGMNFKDNEIPLNGEPNAFWLIVFIAFAMSVSLTLYLIHKKWF 4jq5.3    -------------------------------------------------------------------------- ``` | | | | | | | | | | | | | | | | | | | | | | | | | | | | | | | | | | | | | | | | | | | | | | | | | |
|  | 7kp4.1.A | Claudin-4  *Crystal structure of human claudin-4 in complex with Clostridium perfringens enterotoxin C-terminal domain* | 0.01 |  | 14.81 | 0.09 | 250-276 | X-ray | 3.37 | hetero-1-1-mer |  | HHblits | 0.31 |
| ``` target    MKQVFLSTTTEFKEIDTLEPGTWINLVNPTQNESLEIANTFDIDIADLRAPLDAEEMSRITIEDEYTLIIVDVPVTEERN 7kp4.1    --------------------------------------------------------------------------------  target    NRTYYVTIPLGIIITEETIITTCLEPLPVLDVFINRRLRNFYTFMRSRFIFQILYRNAELYLTALRSIDRKSEQIESQLH 7kp4.1    --------------------------------------------------------------------------------  target    QSTRNEELIELMELEKTIVYFKASLKTNERVIKKLTSSTSNIKKYLEDEDLLEDTLIETQQAIEMADIYGNVLHSMTETF 7kp4.1    --------------------------------------------------------------------------------  target    ASIISNNQNNIMKTLALVTIVMSIPTMVFSAYGMNFKDNEIPLNGEPNAFWLIVFIAFAMSVSLTLYLIHKKWF 7kp4.1    ---------QAARALVIISIIVAALGVLLSVVGGKC-------------------------------------- ``` | | | | | | | | | | | | | | | | | | | | | | | | | | | | | | | | | | | | | | | | | | | | | | | | | |
|  | 5b2g.1.A | Endolysin,Claudin-4  *Crystal structure of human claudin-4 in complex with C-terminal fragment of Clostridium perfringens enterotoxin* | 0.01 |  | 14.81 | 0.09 | 250-276 | X-ray | 3.50 | hetero-1-1-mer |  | HHblits | 0.31 |
| ``` target    MKQVFLSTTTEFKEIDTLEPGTWINLVNPTQNESLEIANTFDIDIADLRAPLDAEEMSRITIEDEYTLIIVDVPVTEERN 5b2g.1    --------------------------------------------------------------------------------  target    NRTYYVTIPLGIIITEETIITTCLEPLPVLDVFINRRLRNFYTFMRSRFIFQILYRNAELYLTALRSIDRKSEQIESQLH 5b2g.1    --------------------------------------------------------------------------------  target    QSTRNEELIELMELEKTIVYFKASLKTNERVIKKLTSSTSNIKKYLEDEDLLEDTLIETQQAIEMADIYGNVLHSMTETF 5b2g.1    --------------------------------------------------------------------------------  target    ASIISNNQNNIMKTLALVTIVMSIPTMVFSAYGMNFKDNEIPLNGEPNAFWLIVFIAFAMSVSLTLYLIHKKWF 5b2g.1    ---------QAARALVIISIIVAALGVLLSVVGGKC-------------------------------------- ``` | | | | | | | | | | | | | | | | | | | | | | | | | | | | | | | | | | | | | | | | | | | | | | | | | |
|  | 5b2g.2.A | Endolysin,Claudin-4  *Crystal structure of human claudin-4 in complex with C-terminal fragment of Clostridium perfringens enterotoxin* | 0.01 |  | 14.81 | 0.09 | 250-276 | X-ray | 3.50 | hetero-1-1-mer |  | HHblits | 0.31 |
| ``` target    MKQVFLSTTTEFKEIDTLEPGTWINLVNPTQNESLEIANTFDIDIADLRAPLDAEEMSRITIEDEYTLIIVDVPVTEERN 5b2g.2    --------------------------------------------------------------------------------  target    NRTYYVTIPLGIIITEETIITTCLEPLPVLDVFINRRLRNFYTFMRSRFIFQILYRNAELYLTALRSIDRKSEQIESQLH 5b2g.2    --------------------------------------------------------------------------------  target    QSTRNEELIELMELEKTIVYFKASLKTNERVIKKLTSSTSNIKKYLEDEDLLEDTLIETQQAIEMADIYGNVLHSMTETF 5b2g.2    --------------------------------------------------------------------------------  target    ASIISNNQNNIMKTLALVTIVMSIPTMVFSAYGMNFKDNEIPLNGEPNAFWLIVFIAFAMSVSLTLYLIHKKWF 5b2g.2    ---------QAARALVIISIIVAALGVLLSVVGGKC-------------------------------------- ``` | | | | | | | | | | | | | | | | | | | | | | | | | | | | | | | | | | | | | | | | | | | | | | | | | |
|  | 5b2g.3.A | Endolysin,Claudin-4  *Crystal structure of human claudin-4 in complex with C-terminal fragment of Clostridium perfringens enterotoxin* | 0.01 |  | 14.81 | 0.09 | 250-276 | X-ray | 3.50 | hetero-1-1-mer |  | HHblits | 0.31 |
| ``` target    MKQVFLSTTTEFKEIDTLEPGTWINLVNPTQNESLEIANTFDIDIADLRAPLDAEEMSRITIEDEYTLIIVDVPVTEERN 5b2g.3    --------------------------------------------------------------------------------  target    NRTYYVTIPLGIIITEETIITTCLEPLPVLDVFINRRLRNFYTFMRSRFIFQILYRNAELYLTALRSIDRKSEQIESQLH 5b2g.3    --------------------------------------------------------------------------------  target    QSTRNEELIELMELEKTIVYFKASLKTNERVIKKLTSSTSNIKKYLEDEDLLEDTLIETQQAIEMADIYGNVLHSMTETF 5b2g.3    --------------------------------------------------------------------------------  target    ASIISNNQNNIMKTLALVTIVMSIPTMVFSAYGMNFKDNEIPLNGEPNAFWLIVFIAFAMSVSLTLYLIHKKWF 5b2g.3    ---------QAARALVIISIIVAALGVLLSVVGGKC-------------------------------------- ``` | | | | | | | | | | | | | | | | | | | | | | | | | | | | | | | | | | | | | | | | | | | | | | | | | |
|  | 5b2g.4.A | Endolysin,Claudin-4  *Crystal structure of human claudin-4 in complex with C-terminal fragment of Clostridium perfringens enterotoxin* | 0.01 |  | 14.81 | 0.09 | 250-276 | X-ray | 3.50 | hetero-1-1-mer |  | HHblits | 0.31 |
| ``` target    MKQVFLSTTTEFKEIDTLEPGTWINLVNPTQNESLEIANTFDIDIADLRAPLDAEEMSRITIEDEYTLIIVDVPVTEERN 5b2g.4    --------------------------------------------------------------------------------  target    NRTYYVTIPLGIIITEETIITTCLEPLPVLDVFINRRLRNFYTFMRSRFIFQILYRNAELYLTALRSIDRKSEQIESQLH 5b2g.4    --------------------------------------------------------------------------------  target    QSTRNEELIELMELEKTIVYFKASLKTNERVIKKLTSSTSNIKKYLEDEDLLEDTLIETQQAIEMADIYGNVLHSMTETF 5b2g.4    --------------------------------------------------------------------------------  target    ASIISNNQNNIMKTLALVTIVMSIPTMVFSAYGMNFKDNEIPLNGEPNAFWLIVFIAFAMSVSLTLYLIHKKWF 5b2g.4    ---------QAARALVIISIIVAALGVLLSVVGGKC-------------------------------------- ``` | | | | | | | | | | | | | | | | | | | | | | | | | | | | | | | | | | | | | | | | | | | | | | | | | |
|  | 6akf.1.A | Claudin-3  *Crystal structure of mouse claudin-3 P134A mutant in complex with C-terminal fragment of Clostridium perfringens enterotoxin* | 0.01 |  | 14.81 | 0.09 | 250-276 | X-ray | 3.90 | hetero-1-1-mer |  | HHblits | 0.30 |
| ``` target    MKQVFLSTTTEFKEIDTLEPGTWINLVNPTQNESLEIANTFDIDIADLRAPLDAEEMSRITIEDEYTLIIVDVPVTEERN 6akf.1    --------------------------------------------------------------------------------  target    NRTYYVTIPLGIIITEETIITTCLEPLPVLDVFINRRLRNFYTFMRSRFIFQILYRNAELYLTALRSIDRKSEQIESQLH 6akf.1    --------------------------------------------------------------------------------  target    QSTRNEELIELMELEKTIVYFKASLKTNERVIKKLTSSTSNIKKYLEDEDLLEDTLIETQQAIEMADIYGNVLHSMTETF 6akf.1    --------------------------------------------------------------------------------  target    ASIISNNQNNIMKTLALVTIVMSIPTMVFSAYGMNFKDNEIPLNGEPNAFWLIVFIAFAMSVSLTLYLIHKKWF 6akf.1    ---------QAARALIVVSILLAAFGLLVALVGAQA-------------------------------------- ``` | | | | | | | | | | | | | | | | | | | | | | | | | | | | | | | | | | | | | | | | | | | | | | | | | |
|  | 6akf.2.A | Claudin-3  *Crystal structure of mouse claudin-3 P134A mutant in complex with C-terminal fragment of Clostridium perfringens enterotoxin* | 0.01 |  | 14.81 | 0.09 | 250-276 | X-ray | 3.90 | hetero-1-1-mer |  | HHblits | 0.30 |
| ``` target    MKQVFLSTTTEFKEIDTLEPGTWINLVNPTQNESLEIANTFDIDIADLRAPLDAEEMSRITIEDEYTLIIVDVPVTEERN 6akf.2    --------------------------------------------------------------------------------  target    NRTYYVTIPLGIIITEETIITTCLEPLPVLDVFINRRLRNFYTFMRSRFIFQILYRNAELYLTALRSIDRKSEQIESQLH 6akf.2    --------------------------------------------------------------------------------  target    QSTRNEELIELMELEKTIVYFKASLKTNERVIKKLTSSTSNIKKYLEDEDLLEDTLIETQQAIEMADIYGNVLHSMTETF 6akf.2    --------------------------------------------------------------------------------  target    ASIISNNQNNIMKTLALVTIVMSIPTMVFSAYGMNFKDNEIPLNGEPNAFWLIVFIAFAMSVSLTLYLIHKKWF 6akf.2    ---------QAARALIVVSILLAAFGLLVALVGAQA-------------------------------------- ``` | | | | | | | | | | | | | | | | | | | | | | | | | | | | | | | | | | | | | | | | | | | | | | | | | |
|  | 6akf.3.A | Claudin-3  *Crystal structure of mouse claudin-3 P134A mutant in complex with C-terminal fragment of Clostridium perfringens enterotoxin* | 0.01 |  | 14.81 | 0.09 | 250-276 | X-ray | 3.90 | hetero-1-1-mer |  | HHblits | 0.30 |
| ``` target    MKQVFLSTTTEFKEIDTLEPGTWINLVNPTQNESLEIANTFDIDIADLRAPLDAEEMSRITIEDEYTLIIVDVPVTEERN 6akf.3    --------------------------------------------------------------------------------  target    NRTYYVTIPLGIIITEETIITTCLEPLPVLDVFINRRLRNFYTFMRSRFIFQILYRNAELYLTALRSIDRKSEQIESQLH 6akf.3    --------------------------------------------------------------------------------  target    QSTRNEELIELMELEKTIVYFKASLKTNERVIKKLTSSTSNIKKYLEDEDLLEDTLIETQQAIEMADIYGNVLHSMTETF 6akf.3    --------------------------------------------------------------------------------  target    ASIISNNQNNIMKTLALVTIVMSIPTMVFSAYGMNFKDNEIPLNGEPNAFWLIVFIAFAMSVSLTLYLIHKKWF 6akf.3    ---------QAARALIVVSILLAAFGLLVALVGAQA-------------------------------------- ``` | | | | | | | | | | | | | | | | | | | | | | | | | | | | | | | | | | | | | | | | | | | | | | | | | |
|  | 6akf.4.A | Claudin-3  *Crystal structure of mouse claudin-3 P134A mutant in complex with C-terminal fragment of Clostridium perfringens enterotoxin* | 0.01 |  | 14.81 | 0.09 | 250-276 | X-ray | 3.90 | hetero-1-1-mer |  | HHblits | 0.30 |
| ``` target    MKQVFLSTTTEFKEIDTLEPGTWINLVNPTQNESLEIANTFDIDIADLRAPLDAEEMSRITIEDEYTLIIVDVPVTEERN 6akf.4    --------------------------------------------------------------------------------  target    NRTYYVTIPLGIIITEETIITTCLEPLPVLDVFINRRLRNFYTFMRSRFIFQILYRNAELYLTALRSIDRKSEQIESQLH 6akf.4    --------------------------------------------------------------------------------  target    QSTRNEELIELMELEKTIVYFKASLKTNERVIKKLTSSTSNIKKYLEDEDLLEDTLIETQQAIEMADIYGNVLHSMTETF 6akf.4    --------------------------------------------------------------------------------  target    ASIISNNQNNIMKTLALVTIVMSIPTMVFSAYGMNFKDNEIPLNGEPNAFWLIVFIAFAMSVSLTLYLIHKKWF 6akf.4    ---------QAARALIVVSILLAAFGLLVALVGAQA-------------------------------------- ``` | | | | | | | | | | | | | | | | | | | | | | | | | | | | | | | | | | | | | | | | | | | | | | | | | |
|  | 6ake.1.A | Claudin-3  *Crystal structure of mouse claudin-3 in complex with C-terminal fragment of Clostridium perfringens enterotoxin* | 0.01 |  | 14.81 | 0.09 | 250-276 | X-ray | 3.60 | hetero-1-1-mer |  | HHblits | 0.30 |
| ``` target    MKQVFLSTTTEFKEIDTLEPGTWINLVNPTQNESLEIANTFDIDIADLRAPLDAEEMSRITIEDEYTLIIVDVPVTEERN 6ake.1    --------------------------------------------------------------------------------  target    NRTYYVTIPLGIIITEETIITTCLEPLPVLDVFINRRLRNFYTFMRSRFIFQILYRNAELYLTALRSIDRKSEQIESQLH 6ake.1    --------------------------------------------------------------------------------  target    QSTRNEELIELMELEKTIVYFKASLKTNERVIKKLTSSTSNIKKYLEDEDLLEDTLIETQQAIEMADIYGNVLHSMTETF 6ake.1    --------------------------------------------------------------------------------  target    ASIISNNQNNIMKTLALVTIVMSIPTMVFSAYGMNFKDNEIPLNGEPNAFWLIVFIAFAMSVSLTLYLIHKKWF 6ake.1    ---------QAARALIVVSILLAAFGLLVALVGAQA-------------------------------------- ``` | | | | | | | | | | | | | | | | | | | | | | | | | | | | | | | | | | | | | | | | | | | | | | | | | |
|  | 6ake.2.A | Claudin-3  *Crystal structure of mouse claudin-3 in complex with C-terminal fragment of Clostridium perfringens enterotoxin* | 0.01 |  | 14.81 | 0.09 | 250-276 | X-ray | 3.60 | hetero-1-1-mer |  | HHblits | 0.30 |
| ``` target    MKQVFLSTTTEFKEIDTLEPGTWINLVNPTQNESLEIANTFDIDIADLRAPLDAEEMSRITIEDEYTLIIVDVPVTEERN 6ake.2    --------------------------------------------------------------------------------  target    NRTYYVTIPLGIIITEETIITTCLEPLPVLDVFINRRLRNFYTFMRSRFIFQILYRNAELYLTALRSIDRKSEQIESQLH 6ake.2    --------------------------------------------------------------------------------  target    QSTRNEELIELMELEKTIVYFKASLKTNERVIKKLTSSTSNIKKYLEDEDLLEDTLIETQQAIEMADIYGNVLHSMTETF 6ake.2    --------------------------------------------------------------------------------  target    ASIISNNQNNIMKTLALVTIVMSIPTMVFSAYGMNFKDNEIPLNGEPNAFWLIVFIAFAMSVSLTLYLIHKKWF 6ake.2    ---------QAARALIVVSILLAAFGLLVALVGAQA-------------------------------------- ``` | | | | | | | | | | | | | | | | | | | | | | | | | | | | | | | | | | | | | | | | | | | | | | | | | |
|  | 4p79.1.A | Claudin-15  *Crystal structure of mouse claudin-15* | 0.01 |  | 11.11 | 0.09 | 250-276 | X-ray | 2.40 | monomer | 2 x OLC | HHblits | 0.29 |
| ``` target    MKQVFLSTTTEFKEIDTLEPGTWINLVNPTQNESLEIANTFDIDIADLRAPLDAEEMSRITIEDEYTLIIVDVPVTEERN 4p79.1    --------------------------------------------------------------------------------  target    NRTYYVTIPLGIIITEETIITTCLEPLPVLDVFINRRLRNFYTFMRSRFIFQILYRNAELYLTALRSIDRKSEQIESQLH 4p79.1    --------------------------------------------------------------------------------  target    QSTRNEELIELMELEKTIVYFKASLKTNERVIKKLTSSTSNIKKYLEDEDLLEDTLIETQQAIEMADIYGNVLHSMTETF 4p79.1    --------------------------------------------------------------------------------  target    ASIISNNQNNIMKTLALVTIVMSIPTMVFSAYGMNFKDNEIPLNGEPNAFWLIVFIAFAMSVSLTLYLIHKKWF 4p79.1    ---------QGCRALMITAILLGFLGLFLGMVGLRA-------------------------------------- ``` | | | | | | | | | | | | | | | | | | | | | | | | | | | | | | | | | | | | | | | | | | | | | | | | | |
|  | 3mk7.1.A | Cytochrome c oxidase, cbb3-type, subunit N  *The structure of CBB3 cytochrome oxidase* | 0.01 |  | 3.57 | 0.09 | 243-270 | X-ray | 3.20 | hetero-oligomer | 2 x HEM, 1 x CU, 2 x CA, 3 x HEC, 1 x FC6 | HHblits | 0.23 |
| ``` target    MKQVFLSTTTEFKEIDTLEPGTWINLVNPTQNESLEIANTFDIDIADLRAPLDAEEMSRITIEDEYTLIIVDVPVTEERN 3mk7.1    --------------------------------------------------------------------------------  target    NRTYYVTIPLGIIITEETIITTCLEPLPVLDVFINRRLRNFYTFMRSRFIFQILYRNAELYLTALRSIDRKSEQIESQLH 3mk7.1    --------------------------------------------------------------------------------  target    QSTRNEELIELMELEKTIVYFKASLKTNERVIKKLTSSTSNIKKYLEDEDLLEDTLIETQQAIEMADIYGNVLHSMTETF 3mk7.1    --------------------------------------------------------------------------------  target    ASIISNNQNNIMKTLALVTIVMSIPTMVFSAYGMNFKDNEIPLNGEPNAFWLIVFIAFAMSVSLTLYLIHKKWF 3mk7.1    --LFAPKLAAFTFWGWQLVILLAAISLPLG-------------------------------------------- ``` | | | | | | | | | | | | | | | | | | | | | | | | | | | | | | | | | | | | | | | | | | | | | | | | | |
|  | 7jr7.1.B | ATP-binding cassette sub-family G member 8  *Cryo-EM structure of ABCG5/G8 in complex with Fab 2E10 and 11F4* | 0.01 |  | 3.57 | 0.09 | 253-283 | EM | 0.00 | hetero-1-1-1-1-1-1-… |  | HHblits | 0.23 |
| ``` target    MKQVFLSTTTEFKEIDTLEPGTWINLVNPTQNESLEIANTFDIDIADLRAPLDAEEMSRITIEDEYTLIIVDVPVTEERN 7jr7.1    --------------------------------------------------------------------------------  target    NRTYYVTIPLGIIITEETIITTCLEPLPVLDVFINRRLRNFYTFMRSRFIFQILYRNAELYLTALRSIDRKSEQIESQLH 7jr7.1    --------------------------------------------------------------------------------  target    QSTRNEELIELMELEKTIVYFKASLKTNERVIKKLTSSTSNIKKYLEDEDLLEDTLIETQQAIEMADIYGNVLHSMTETF 7jr7.1    --------------------------------------------------------------------------------  target    ASIISNNQNNIMKTLALVTIVMSIPTMVFSAYGMNFKDNEIPLNGEPNAFWLIVFIAFAMSVSLTLYLIHKKWF 7jr7.1    ------------MASFFSNALYNSFYLAGGFM-INLSS--LWT------------------------------- ``` | | | | | | | | | | | | | | | | | | | | | | | | | | | | | | | | | | | | | | | | | | | | | | | | | |
|  | 5aww.1.A | Protein translocase subunit SecY  *Precise Resting State of Thermus thermophilus SecYEG* | 0.01 |  | 7.14 | 0.09 | 247-274 | X-ray | 2.72 | hetero-1-1-1-mer | 9 x OLC | HHblits | 0.23 |
| ``` target    MKQVFLSTTTEFKEIDTLEPGTWINLVNPTQNESLEIANTFDIDIADLRAPLDAEEMSRITIEDEYTLIIVDVPVTEERN 5aww.1    --------------------------------------------------------------------------------  target    NRTYYVTIPLGIIITEETIITTCLEPLPVLDVFINRRLRNFYTFMRSRFIFQILYRNAELYLTALRSIDRKSEQIESQLH 5aww.1    --------------------------------------------------------------------------------  target    QSTRNEELIELMELEKTIVYFKASLKTNERVIKKLTSSTSNIKKYLEDEDLLEDTLIETQQAIEMADIYGNVLHSMTETF 5aww.1    --------------------------------------------------------------------------------  target    ASIISNNQNNIMKTLALVTIVMSIPTMVFSAYGMNFKDNEIPLNGEPNAFWLIVFIAFAMSVSLTLYLIHKKWF 5aww.1    ------IINQYTRIGGIALGAFQGFFLATAFLGA---------------------------------------- ``` | | | | | | | | | | | | | | | | | | | | | | | | | | | | | | | | | | | | | | | | | | | | | | | | | |
|  | 5ch4.1.A | Protein translocase subunit SecY  *Peptide-Bound State of Thermus thermophilus SecYEG* | 0.01 |  | 7.14 | 0.09 | 247-274 | X-ray | 3.64 | hetero-1-1-1-mer |  | HHblits | 0.23 |
| ``` target    MKQVFLSTTTEFKEIDTLEPGTWINLVNPTQNESLEIANTFDIDIADLRAPLDAEEMSRITIEDEYTLIIVDVPVTEERN 5ch4.1    --------------------------------------------------------------------------------  target    NRTYYVTIPLGIIITEETIITTCLEPLPVLDVFINRRLRNFYTFMRSRFIFQILYRNAELYLTALRSIDRKSEQIESQLH 5ch4.1    --------------------------------------------------------------------------------  target    QSTRNEELIELMELEKTIVYFKASLKTNERVIKKLTSSTSNIKKYLEDEDLLEDTLIETQQAIEMADIYGNVLHSMTETF 5ch4.1    --------------------------------------------------------------------------------  target    ASIISNNQNNIMKTLALVTIVMSIPTMVFSAYGMNFKDNEIPLNGEPNAFWLIVFIAFAMSVSLTLYLIHKKWF 5ch4.1    ------IINQYTRIGGIALGAFQGFFLATAFLGA---------------------------------------- ``` | | | | | | | | | | | | | | | | | | | | | | | | | | | | | | | | | | | | | | | | | | | | | | | | | |
|  | 2zjs.1.A | Preprotein translocase SecY subunit  *Crystal Structure of SecYE translocon from Thermus thermophilus with a Fab fragment* | 0.01 |  | 7.14 | 0.09 | 247-274 | X-ray | 3.20 | hetero-oligomer | 1 x ZN | HHblits | 0.23 |
| ``` target    MKQVFLSTTTEFKEIDTLEPGTWINLVNPTQNESLEIANTFDIDIADLRAPLDAEEMSRITIEDEYTLIIVDVPVTEERN 2zjs.1    --------------------------------------------------------------------------------  target    NRTYYVTIPLGIIITEETIITTCLEPLPVLDVFINRRLRNFYTFMRSRFIFQILYRNAELYLTALRSIDRKSEQIESQLH 2zjs.1    --------------------------------------------------------------------------------  target    QSTRNEELIELMELEKTIVYFKASLKTNERVIKKLTSSTSNIKKYLEDEDLLEDTLIETQQAIEMADIYGNVLHSMTETF 2zjs.1    --------------------------------------------------------------------------------  target    ASIISNNQNNIMKTLALVTIVMSIPTMVFSAYGMNFKDNEIPLNGEPNAFWLIVFIAFAMSVSLTLYLIHKKWF 2zjs.1    ------IINQYTRIGGIALGAFQGFFLATAFLGA---------------------------------------- ``` | | | | | | | | | | | | | | | | | | | | | | | | | | | | | | | | | | | | | | | | | | | | | | | | | |
|  | 2zqp.1.A | Preprotein translocase SecY subunit  *Crystal Structure of SecYE translocon from Thermus thermophilus* | 0.01 |  | 7.14 | 0.09 | 247-274 | X-ray | 6.00 | hetero-oligomer |  | HHblits | 0.23 |
| ``` target    MKQVFLSTTTEFKEIDTLEPGTWINLVNPTQNESLEIANTFDIDIADLRAPLDAEEMSRITIEDEYTLIIVDVPVTEERN 2zqp.1    --------------------------------------------------------------------------------  target    NRTYYVTIPLGIIITEETIITTCLEPLPVLDVFINRRLRNFYTFMRSRFIFQILYRNAELYLTALRSIDRKSEQIESQLH 2zqp.1    --------------------------------------------------------------------------------  target    QSTRNEELIELMELEKTIVYFKASLKTNERVIKKLTSSTSNIKKYLEDEDLLEDTLIETQQAIEMADIYGNVLHSMTETF 2zqp.1    --------------------------------------------------------------------------------  target    ASIISNNQNNIMKTLALVTIVMSIPTMVFSAYGMNFKDNEIPLNGEPNAFWLIVFIAFAMSVSLTLYLIHKKWF 2zqp.1    ------IINQYTRIGGIALGAFQGFFLATAFLGA---------------------------------------- ``` | | | | | | | | | | | | | | | | | | | | | | | | | | | | | | | | | | | | | | | | | | | | | | | | | |
|  | 6xp5.1.I | Mediator of RNA polymerase II transcription subunit 8  *Head-Middle module of Mediator* | 0.01 |  | 28.00 | 0.08 | 166-190 | EM | 0.00 | hetero-1-1-1-1-1-1-… |  | HHblits | 0.31 |
| ``` target    MKQVFLSTTTEFKEIDTLEPGTWINLVNPTQNESLEIANTFDIDIADLRAPLDAEEMSRITIEDEYTLIIVDVPVTEERN 6xp5.1    --------------------------------------------------------------------------------  target    NRTYYVTIPLGIIITEETIITTCLEPLPVLDVFINRRLRNFYTFMRSRFIFQILYRNAELYLTALRSIDRKSEQIESQLH 6xp5.1    --------------------------------------------------------------------------------  target    QSTRNEELIELMELEKTIVYFKASLKTNERVIKKLTSSTSNIKKYLEDEDLLEDTLIETQQAIEMADIYGNVLHSMTETF 6xp5.1    -----EELKQLELLRNRFAQLQSSLTSLAG--------------------------------------------------  target    ASIISNNQNNIMKTLALVTIVMSIPTMVFSAYGMNFKDNEIPLNGEPNAFWLIVFIAFAMSVSLTLYLIHKKWF 6xp5.1    -------------------------------------------------------------------------- ``` | | | | | | | | | | | | | | | | | | | | | | | | | | | | | | | | | | | | | | | | | | | | | | | | | |
|  | 3jc8.42.A | Type 4 fimbrial assembly protein PilC  *Architectural model of the type IVa pilus machine in a piliated state* | 0.02 |  | 15.38 | 0.08 | 215-240 | EM | 0.00 | monomer |  | HHblits | 0.28 |
| ``` target    MKQVFLSTTTEFKEIDTLEPGTWINLVNPTQNESLEIANTFDIDIADLRAPLDAEEMSRITIEDEYTLIIVDVPVTEERN 3jc8.42   --------------------------------------------------------------------------------  target    NRTYYVTIPLGIIITEETIITTCLEPLPVLDVFINRRLRNFYTFMRSRFIFQILYRNAELYLTALRSIDRKSEQIESQLH 3jc8.42   --------------------------------------------------------------------------------  target    QSTRNEELIELMELEKTIVYFKASLKTNERVIKKLTSSTSNIKKYLEDEDLLEDTLIETQQAIEMADIYGNVLHSMTETF 3jc8.42   ------------------------------------------------------LYVQLCAAGEVGGILDAILNRLAAYR  target    ASIISNNQNNIMKTLALVTIVMSIPTMVFSAYGMNFKDNEIPLNGEPNAFWLIVFIAFAMSVSLTLYLIHKKWF 3jc8.42   -------------------------------------------------------------------------- ``` | | | | | | | | | | | | | | | | | | | | | | | | | | | | | | | | | | | | | | | | | | | | | | | | | |
|  | 3jc8.43.A | Type 4 fimbrial assembly protein PilC  *Architectural model of the type IVa pilus machine in a piliated state* | 0.02 |  | 15.38 | 0.08 | 215-240 | EM | 0.00 | monomer |  | HHblits | 0.28 |
| ``` target    MKQVFLSTTTEFKEIDTLEPGTWINLVNPTQNESLEIANTFDIDIADLRAPLDAEEMSRITIEDEYTLIIVDVPVTEERN 3jc8.43   --------------------------------------------------------------------------------  target    NRTYYVTIPLGIIITEETIITTCLEPLPVLDVFINRRLRNFYTFMRSRFIFQILYRNAELYLTALRSIDRKSEQIESQLH 3jc8.43   --------------------------------------------------------------------------------  target    QSTRNEELIELMELEKTIVYFKASLKTNERVIKKLTSSTSNIKKYLEDEDLLEDTLIETQQAIEMADIYGNVLHSMTETF 3jc8.43   ------------------------------------------------------LYVQLCAAGEVGGILDAILNRLAAYR  target    ASIISNNQNNIMKTLALVTIVMSIPTMVFSAYGMNFKDNEIPLNGEPNAFWLIVFIAFAMSVSLTLYLIHKKWF 3jc8.43   -------------------------------------------------------------------------- ``` | | | | | | | | | | | | | | | | | | | | | | | | | | | | | | | | | | | | | | | | | | | | | | | | | |
|  | 6hwh.1.K | Cytochrome c oxidase polypeptide 4  *Structure of a functional obligate respiratory supercomplex from Mycobacterium smegmatis* | 0.01 |  | 12.00 | 0.08 | 252-276 | EM | 0.00 | hetero-2-2-2-2-2-4-… | 2 x FES, 8 x CDL, 4 x MQ9, 6 x CU, 4 x HAS, 4 x HEC, 4 x HEM | HHblits | 0.31 |
| ``` target    MKQVFLSTTTEFKEIDTLEPGTWINLVNPTQNESLEIANTFDIDIADLRAPLDAEEMSRITIEDEYTLIIVDVPVTEERN 6hwh.1    --------------------------------------------------------------------------------  target    NRTYYVTIPLGIIITEETIITTCLEPLPVLDVFINRRLRNFYTFMRSRFIFQILYRNAELYLTALRSIDRKSEQIESQLH 6hwh.1    --------------------------------------------------------------------------------  target    QSTRNEELIELMELEKTIVYFKASLKTNERVIKKLTSSTSNIKKYLEDEDLLEDTLIETQQAIEMADIYGNVLHSMTETF 6hwh.1    --------------------------------------------------------------------------------  target    ASIISNNQNNIMKTLALVTIVMSIPTMVFSAYGMNFKDNEIPLNGEPNAFWLIVFIAFAMSVSLTLYLIHKKWF 6hwh.1    -----------ARLFEILTAFFALAAVVYAVLTAMF-------------------------------------- ``` | | | | | | | | | | | | | | | | | | | | | | | | | | | | | | | | | | | | | | | | | | | | | | | | | |
|  | 6adq.1.D | Cytochrome c oxidase polypeptide 4  *Respiratory Complex CIII2CIV2SOD2 from Mycobacterium smegmatis* | 0.01 |  | 12.00 | 0.08 | 252-276 | EM | 0.00 | hetero-2-2-2-2-2-2-… | 8 x CU, 4 x HEA, 18 x CDL, 8 x 9Y0, 4 x PLM, 4 x 9XX, 8 x 9YF, 4 x HEM, 10 x MQ9, 4 x HEC, 2 x FES | HHblits | 0.31 |
| ``` target    MKQVFLSTTTEFKEIDTLEPGTWINLVNPTQNESLEIANTFDIDIADLRAPLDAEEMSRITIEDEYTLIIVDVPVTEERN 6adq.1    --------------------------------------------------------------------------------  target    NRTYYVTIPLGIIITEETIITTCLEPLPVLDVFINRRLRNFYTFMRSRFIFQILYRNAELYLTALRSIDRKSEQIESQLH 6adq.1    --------------------------------------------------------------------------------  target    QSTRNEELIELMELEKTIVYFKASLKTNERVIKKLTSSTSNIKKYLEDEDLLEDTLIETQQAIEMADIYGNVLHSMTETF 6adq.1    --------------------------------------------------------------------------------  target    ASIISNNQNNIMKTLALVTIVMSIPTMVFSAYGMNFKDNEIPLNGEPNAFWLIVFIAFAMSVSLTLYLIHKKWF 6adq.1    -----------ARLFEILTAFFALAAVVYAVLTAMF-------------------------------------- ``` | | | | | | | | | | | | | | | | | | | | | | | | | | | | | | | | | | | | | | | | | | | | | | | | | |
|  | 6ov2.1.A | Claudin-9  *Crystal structure of human claudin-9 in complex with Clostridium perfringens entertoxin C-terminal domain in closed form* | 0.01 |  | 8.00 | 0.08 | 251-275 | X-ray | 3.20 | hetero-1-1-mer |  | HHblits | 0.31 |
| ``` target    MKQVFLSTTTEFKEIDTLEPGTWINLVNPTQNESLEIANTFDIDIADLRAPLDAEEMSRITIEDEYTLIIVDVPVTEERN 6ov2.1    --------------------------------------------------------------------------------  target    NRTYYVTIPLGIIITEETIITTCLEPLPVLDVFINRRLRNFYTFMRSRFIFQILYRNAELYLTALRSIDRKSEQIESQLH 6ov2.1    --------------------------------------------------------------------------------  target    QSTRNEELIELMELEKTIVYFKASLKTNERVIKKLTSSTSNIKKYLEDEDLLEDTLIETQQAIEMADIYGNVLHSMTETF 6ov2.1    --------------------------------------------------------------------------------  target    ASIISNNQNNIMKTLALVTIVMSIPTMVFSAYGMNFKDNEIPLNGEPNAFWLIVFIAFAMSVSLTLYLIHKKWF 6ov2.1    ----------AARALCVIALLLALLGLLVAITGAQ--------------------------------------- ``` | | | | | | | | | | | | | | | | | | | | | | | | | | | | | | | | | | | | | | | | | | | | | | | | | |
|  | 6ov3.1.A | Claudin-9  *Crystal structure of human claudin-9 in complex with Clostridium perfringens entertoxin C-terminal domain in open form* | 0.01 |  | 8.00 | 0.08 | 251-275 | X-ray | 3.25 | hetero-1-1-mer |  | HHblits | 0.31 |
| ``` target    MKQVFLSTTTEFKEIDTLEPGTWINLVNPTQNESLEIANTFDIDIADLRAPLDAEEMSRITIEDEYTLIIVDVPVTEERN 6ov3.1    --------------------------------------------------------------------------------  target    NRTYYVTIPLGIIITEETIITTCLEPLPVLDVFINRRLRNFYTFMRSRFIFQILYRNAELYLTALRSIDRKSEQIESQLH 6ov3.1    --------------------------------------------------------------------------------  target    QSTRNEELIELMELEKTIVYFKASLKTNERVIKKLTSSTSNIKKYLEDEDLLEDTLIETQQAIEMADIYGNVLHSMTETF 6ov3.1    --------------------------------------------------------------------------------  target    ASIISNNQNNIMKTLALVTIVMSIPTMVFSAYGMNFKDNEIPLNGEPNAFWLIVFIAFAMSVSLTLYLIHKKWF 6ov3.1    ----------AARALCVIALLLALLGLLVAITGAQ--------------------------------------- ``` | | | | | | | | | | | | | | | | | | | | | | | | | | | | | | | | | | | | | | | | | | | | | | | | | |
|  | 2c5i.1.B | T-SNARE AFFECTING A LATE GOLGI COMPARTMENT PROTEIN 1  *N-TERMINAL DOMAIN OF TLG1 COMPLEXED WITH N-TERMINUS OF VPS51 IN DISTORTED CONFORMATION* | 0.00 |  | 7.69 | 0.08 | 136-161 | X-ray | 2.30 | hetero-oligomer |  | HHblits | 0.27 |
| ``` target    MKQVFLSTTTEFKEIDTLEPGTWINLVNPTQNESLEIANTFDIDIADLRAPLDAEEMSRITIEDEYTLIIVDVPVTEERN 2c5i.1    --------------------------------------------------------------------------------  target    NRTYYVTIPLGIIITEETIITTCLEPLPVLDVFINRRLRNFYTFMRSRFIFQILYRNAELYLTALRSIDRKSEQIESQLH 2c5i.1    -------------------------------------------------------DQEEEIQDILKDVEETIVDLDRSII  target    QSTRNEELIELMELEKTIVYFKASLKTNERVIKKLTSSTSNIKKYLEDEDLLEDTLIETQQAIEMADIYGNVLHSMTETF 2c5i.1    V-------------------------------------------------------------------------------  target    ASIISNNQNNIMKTLALVTIVMSIPTMVFSAYGMNFKDNEIPLNGEPNAFWLIVFIAFAMSVSLTLYLIHKKWF 2c5i.1    -------------------------------------------------------------------------- ``` | | | | | | | | | | | | | | | | | | | | | | | | | | | | | | | | | | | | | | | | | | | | | | | | | |
|  | 6tpi.1.A | Murein hydrolase activator EnvC  *EnvC bound to the FtsX periplasmic domain* | 0.01 |  | 16.00 | 0.08 | 171-195 | X-ray | 2.10 | hetero-1-2-mer |  | HHblits | 0.30 |
| ``` target    MKQVFLSTTTEFKEIDTLEPGTWINLVNPTQNESLEIANTFDIDIADLRAPLDAEEMSRITIEDEYTLIIVDVPVTEERN 6tpi.1    --------------------------------------------------------------------------------  target    NRTYYVTIPLGIIITEETIITTCLEPLPVLDVFINRRLRNFYTFMRSRFIFQILYRNAELYLTALRSIDRKSEQIESQLH 6tpi.1    --------------------------------------------------------------------------------  target    QSTRNEELIELMELEKTIVYFKASLKTNERVIKKLTSSTSNIKKYLEDEDLLEDTLIETQQAIEMADIYGNVLHSMTETF 6tpi.1    ----------IDEMNASIAKLEQQKAAQERSLAAQ---------------------------------------------  target    ASIISNNQNNIMKTLALVTIVMSIPTMVFSAYGMNFKDNEIPLNGEPNAFWLIVFIAFAMSVSLTLYLIHKKWF 6tpi.1    -------------------------------------------------------------------------- ``` | | | | | | | | | | | | | | | | | | | | | | | | | | | | | | | | | | | | | | | | | | | | | | | | | |
|  | 5do7.1.B | ATP-binding cassette sub-family G member 8  *Crystal Structure of the Human Sterol Transporter ABCG5/ABCG8* | 0.01 |  | 3.70 | 0.09 | 254-283 | X-ray | 3.93 | hetero-1-1-mer |  | HHblits | 0.23 |
| ``` target    MKQVFLSTTTEFKEIDTLEPGTWINLVNPTQNESLEIANTFDIDIADLRAPLDAEEMSRITIEDEYTLIIVDVPVTEERN 5do7.1    --------------------------------------------------------------------------------  target    NRTYYVTIPLGIIITEETIITTCLEPLPVLDVFINRRLRNFYTFMRSRFIFQILYRNAELYLTALRSIDRKSEQIESQLH 5do7.1    --------------------------------------------------------------------------------  target    QSTRNEELIELMELEKTIVYFKASLKTNERVIKKLTSSTSNIKKYLEDEDLLEDTLIETQQAIEMADIYGNVLHSMTETF 5do7.1    --------------------------------------------------------------------------------  target    ASIISNNQNNIMKTLALVTIVMSIPTMVFSAYGMNFKDNEIPLNGEPNAFWLIVFIAFAMSVSLTLYLIHKKWF 5do7.1    -------------ASFFSNALYNSFYLAGGFM-INLSS--LWT------------------------------- ``` | | | | | | | | | | | | | | | | | | | | | | | | | | | | | | | | | | | | | | | | | | | | | | | | | |
|  | 5do7.2.A | ATP-binding cassette sub-family G member 8  *Crystal Structure of the Human Sterol Transporter ABCG5/ABCG8* | 0.01 |  | 3.70 | 0.09 | 254-283 | X-ray | 3.93 | hetero-1-1-mer |  | HHblits | 0.23 |
| ``` target    MKQVFLSTTTEFKEIDTLEPGTWINLVNPTQNESLEIANTFDIDIADLRAPLDAEEMSRITIEDEYTLIIVDVPVTEERN 5do7.2    --------------------------------------------------------------------------------  target    NRTYYVTIPLGIIITEETIITTCLEPLPVLDVFINRRLRNFYTFMRSRFIFQILYRNAELYLTALRSIDRKSEQIESQLH 5do7.2    --------------------------------------------------------------------------------  target    QSTRNEELIELMELEKTIVYFKASLKTNERVIKKLTSSTSNIKKYLEDEDLLEDTLIETQQAIEMADIYGNVLHSMTETF 5do7.2    --------------------------------------------------------------------------------  target    ASIISNNQNNIMKTLALVTIVMSIPTMVFSAYGMNFKDNEIPLNGEPNAFWLIVFIAFAMSVSLTLYLIHKKWF 5do7.2    -------------ASFFSNALYNSFYLAGGFM-INLSS--LWT------------------------------- ``` | | | | | | | | | | | | | | | | | | | | | | | | | | | | | | | | | | | | | | | | | | | | | | | | | |
|  | 6qkc.1.E | Voltage-dependent calcium channel gamma-8 subunit  *GluA1/2 In complex with auxiliary subunit gamma-8* | 0.01 |  | 12.00 | 0.08 | 253-277 | EM | 4.10 | hetero-2-2-2-mer | 4 x E2Q, 7 x OLC | HHblits | 0.28 |
| ``` target    MKQVFLSTTTEFKEIDTLEPGTWINLVNPTQNESLEIANTFDIDIADLRAPLDAEEMSRITIEDEYTLIIVDVPVTEERN 6qkc.1    --------------------------------------------------------------------------------  target    NRTYYVTIPLGIIITEETIITTCLEPLPVLDVFINRRLRNFYTFMRSRFIFQILYRNAELYLTALRSIDRKSEQIESQLH 6qkc.1    --------------------------------------------------------------------------------  target    QSTRNEELIELMELEKTIVYFKASLKTNERVIKKLTSSTSNIKKYLEDEDLLEDTLIETQQAIEMADIYGNVLHSMTETF 6qkc.1    --------------------------------------------------------------------------------  target    ASIISNNQNNIMKTLALVTIVMSIPTMVFSAYGMNFKDNEIPLNGEPNAFWLIVFIAFAMSVSLTLYLIHKKWF 6qkc.1    ------------SIFPILSAILLLLGGVCVAASRVYK------------------------------------- ``` | | | | | | | | | | | | | | | | | | | | | | | | | | | | | | | | | | | | | | | | | | | | | | | | | |
|  | 1lvf.1.A | syntaxin 6  *syntaxin 6* | 0.00 |  | 20.83 | 0.08 | 137-160 | X-ray | 2.10 | monomer |  | HHblits | 0.31 |
| ``` target    MKQVFLSTTTEFKEIDTLEPGTWINLVNPTQNESLEIANTFDIDIADLRAPLDAEEMSRITIEDEYTLIIVDVPVTEERN 1lvf.1    --------------------------------------------------------------------------------  target    NRTYYVTIPLGIIITEETIITTCLEPLPVLDVFINRRLRNFYTFMRSRFIFQILYRNAELYLTALRSIDRKSEQIESQLH 1lvf.1    --------------------------------------------------------TTNELRNNLRSIEWDLEDLDETIS  target    QSTRNEELIELMELEKTIVYFKASLKTNERVIKKLTSSTSNIKKYLEDEDLLEDTLIETQQAIEMADIYGNVLHSMTETF 1lvf.1    --------------------------------------------------------------------------------  target    ASIISNNQNNIMKTLALVTIVMSIPTMVFSAYGMNFKDNEIPLNGEPNAFWLIVFIAFAMSVSLTLYLIHKKWF 1lvf.1    -------------------------------------------------------------------------- ``` | | | | | | | | | | | | | | | | | | | | | | | | | | | | | | | | | | | | | | | | | | | | | | | | | |
|  | 1lvf.2.A | syntaxin 6  *syntaxin 6* | 0.00 |  | 20.83 | 0.08 | 137-160 | X-ray | 2.10 | monomer |  | HHblits | 0.31 |
| ``` target    MKQVFLSTTTEFKEIDTLEPGTWINLVNPTQNESLEIANTFDIDIADLRAPLDAEEMSRITIEDEYTLIIVDVPVTEERN 1lvf.2    --------------------------------------------------------------------------------  target    NRTYYVTIPLGIIITEETIITTCLEPLPVLDVFINRRLRNFYTFMRSRFIFQILYRNAELYLTALRSIDRKSEQIESQLH 1lvf.2    --------------------------------------------------------TTNELRNNLRSIEWDLEDLDETIS  target    QSTRNEELIELMELEKTIVYFKASLKTNERVIKKLTSSTSNIKKYLEDEDLLEDTLIETQQAIEMADIYGNVLHSMTETF 1lvf.2    --------------------------------------------------------------------------------  target    ASIISNNQNNIMKTLALVTIVMSIPTMVFSAYGMNFKDNEIPLNGEPNAFWLIVFIAFAMSVSLTLYLIHKKWF 1lvf.2    -------------------------------------------------------------------------- ``` | | | | | | | | | | | | | | | | | | | | | | | | | | | | | | | | | | | | | | | | | | | | | | | | | |
|  | 2mjo.1.A | Tumor necrosis factor receptor superfamily member 16  *NMR structure of p75 transmembrane domain C257A mutant in DPC micelles* | 0.00 |  | 31.82 | 0.07 | 292-313 | NMR | 0.00 | homo-dimer |  | HHblits | 0.37 |
| ``` target    MKQVFLSTTTEFKEIDTLEPGTWINLVNPTQNESLEIANTFDIDIADLRAPLDAEEMSRITIEDEYTLIIVDVPVTEERN 2mjo.1    --------------------------------------------------------------------------------  target    NRTYYVTIPLGIIITEETIITTCLEPLPVLDVFINRRLRNFYTFMRSRFIFQILYRNAELYLTALRSIDRKSEQIESQLH 2mjo.1    --------------------------------------------------------------------------------  target    QSTRNEELIELMELEKTIVYFKASLKTNERVIKKLTSSTSNIKKYLEDEDLLEDTLIETQQAIEMADIYGNVLHSMTETF 2mjo.1    --------------------------------------------------------------------------------  target    ASIISNNQNNIMKTLALVTIVMSIPTMVFSAYGMNFKDNEIPLNGEPNAFWLIVFIAFAMSVSLTLYLIHKKWF 2mjo.1    ---------------------------------------------------VYASILAAVVVGLVAYIAFKRW- ``` | | | | | | | | | | | | | | | | | | | | | | | | | | | | | | | | | | | | | | | | | | | | | | | | | |
|  | 2mjo.1.B | Tumor necrosis factor receptor superfamily member 16  *NMR structure of p75 transmembrane domain C257A mutant in DPC micelles* | 0.00 |  | 31.82 | 0.07 | 292-313 | NMR | 0.00 | homo-dimer |  | HHblits | 0.37 |
| ``` target    MKQVFLSTTTEFKEIDTLEPGTWINLVNPTQNESLEIANTFDIDIADLRAPLDAEEMSRITIEDEYTLIIVDVPVTEERN 2mjo.1    --------------------------------------------------------------------------------  target    NRTYYVTIPLGIIITEETIITTCLEPLPVLDVFINRRLRNFYTFMRSRFIFQILYRNAELYLTALRSIDRKSEQIESQLH 2mjo.1    --------------------------------------------------------------------------------  target    QSTRNEELIELMELEKTIVYFKASLKTNERVIKKLTSSTSNIKKYLEDEDLLEDTLIETQQAIEMADIYGNVLHSMTETF 2mjo.1    --------------------------------------------------------------------------------  target    ASIISNNQNNIMKTLALVTIVMSIPTMVFSAYGMNFKDNEIPLNGEPNAFWLIVFIAFAMSVSLTLYLIHKKWF 2mjo.1    ---------------------------------------------------VYASILAAVVVGLVAYIAFKRW- ``` | | | | | | | | | | | | | | | | | | | | | | | | | | | | | | | | | | | | | | | | | | | | | | | | | |
|  | 4j9u.1.A | Trk system potassium uptake protein TrkH  *Crystal Structure of the TrkH/TrkA potassium transport complex* | 0.00 |  | 31.82 | 0.07 | 254-275 | X-ray | 3.80 | hetero-oligomer | 13 x TBR, 2 x K, 4 x NAD | HHblits | 0.35 |
| ``` target    MKQVFLSTTTEFKEIDTLEPGTWINLVNPTQNESLEIANTFDIDIADLRAPLDAEEMSRITIEDEYTLIIVDVPVTEERN 4j9u.1    --------------------------------------------------------------------------------  target    NRTYYVTIPLGIIITEETIITTCLEPLPVLDVFINRRLRNFYTFMRSRFIFQILYRNAELYLTALRSIDRKSEQIESQLH 4j9u.1    --------------------------------------------------------------------------------  target    QSTRNEELIELMELEKTIVYFKASLKTNERVIKKLTSSTSNIKKYLEDEDLLEDTLIETQQAIEMADIYGNVLHSMTETF 4j9u.1    --------------------------------------------------------------------------------  target    ASIISNNQNNIMKTLALVTIVMSIPTMVFSAYGMNFKDNEIPLNGEPNAFWLIVFIAFAMSVSLTLYLIHKKWF 4j9u.1    -------------LLALFSVTMLAPALVALLYRDG--------------------------------------- ``` | | | | | | | | | | | | | | | | | | | | | | | | | | | | | | | | | | | | | | | | | | | | | | | | | |
|  | 6v4j.1.D | Trk system potassium uptake protein TrkH  *Structure of TrkH-TrkA in complex with ATP* | 0.00 |  | 31.82 | 0.07 | 254-275 | EM | 0.00 | hetero-4-4-mer |  | HHblits | 0.35 |
| ``` target    MKQVFLSTTTEFKEIDTLEPGTWINLVNPTQNESLEIANTFDIDIADLRAPLDAEEMSRITIEDEYTLIIVDVPVTEERN 6v4j.1    --------------------------------------------------------------------------------  target    NRTYYVTIPLGIIITEETIITTCLEPLPVLDVFINRRLRNFYTFMRSRFIFQILYRNAELYLTALRSIDRKSEQIESQLH 6v4j.1    --------------------------------------------------------------------------------  target    QSTRNEELIELMELEKTIVYFKASLKTNERVIKKLTSSTSNIKKYLEDEDLLEDTLIETQQAIEMADIYGNVLHSMTETF 6v4j.1    --------------------------------------------------------------------------------  target    ASIISNNQNNIMKTLALVTIVMSIPTMVFSAYGMNFKDNEIPLNGEPNAFWLIVFIAFAMSVSLTLYLIHKKWF 6v4j.1    -------------LLALFSVTMLAPALVALLYRDG--------------------------------------- ``` | | | | | | | | | | | | | | | | | | | | | | | | | | | | | | | | | | | | | | | | | | | | | | | | | |
|  | 6v4j.1.B | Trk system potassium uptake protein TrkH  *Structure of TrkH-TrkA in complex with ATP* | 0.00 |  | 31.82 | 0.07 | 254-275 | EM | 0.00 | hetero-4-4-mer |  | HHblits | 0.35 |
| ``` target    MKQVFLSTTTEFKEIDTLEPGTWINLVNPTQNESLEIANTFDIDIADLRAPLDAEEMSRITIEDEYTLIIVDVPVTEERN 6v4j.1    --------------------------------------------------------------------------------  target    NRTYYVTIPLGIIITEETIITTCLEPLPVLDVFINRRLRNFYTFMRSRFIFQILYRNAELYLTALRSIDRKSEQIESQLH 6v4j.1    --------------------------------------------------------------------------------  target    QSTRNEELIELMELEKTIVYFKASLKTNERVIKKLTSSTSNIKKYLEDEDLLEDTLIETQQAIEMADIYGNVLHSMTETF 6v4j.1    --------------------------------------------------------------------------------  target    ASIISNNQNNIMKTLALVTIVMSIPTMVFSAYGMNFKDNEIPLNGEPNAFWLIVFIAFAMSVSLTLYLIHKKWF 6v4j.1    -------------LLALFSVTMLAPALVALLYRDG--------------------------------------- ``` | | | | | | | | | | | | | | | | | | | | | | | | | | | | | | | | | | | | | | | | | | | | | | | | | |
|  | 6v4l.1.E | Trk system potassium uptake protein TrkH  *Structure of TrkH-TrkA in complex with ATPgammaS* | 0.01 |  | 31.82 | 0.07 | 254-275 | X-ray | 3.80 | hetero-4-4-mer | 8 x AGS | HHblits | 0.35 |
| ``` target    MKQVFLSTTTEFKEIDTLEPGTWINLVNPTQNESLEIANTFDIDIADLRAPLDAEEMSRITIEDEYTLIIVDVPVTEERN 6v4l.1    --------------------------------------------------------------------------------  target    NRTYYVTIPLGIIITEETIITTCLEPLPVLDVFINRRLRNFYTFMRSRFIFQILYRNAELYLTALRSIDRKSEQIESQLH 6v4l.1    --------------------------------------------------------------------------------  target    QSTRNEELIELMELEKTIVYFKASLKTNERVIKKLTSSTSNIKKYLEDEDLLEDTLIETQQAIEMADIYGNVLHSMTETF 6v4l.1    --------------------------------------------------------------------------------  target    ASIISNNQNNIMKTLALVTIVMSIPTMVFSAYGMNFKDNEIPLNGEPNAFWLIVFIAFAMSVSLTLYLIHKKWF 6v4l.1    -------------LLALFSVTMLAPALVALLYRDG--------------------------------------- ``` | | | | | | | | | | | | | | | | | | | | | | | | | | | | | | | | | | | | | | | | | | | | | | | | | |
|  | 6v4k.1.D | Trk system potassium uptake protein  *Structure of TrkH-TrkA in complex with ADP* | 0.01 |  | 31.82 | 0.07 | 254-275 | X-ray | 3.53 | hetero-4-4-mer | 4 x ADP | HHblits | 0.35 |
| ``` target    MKQVFLSTTTEFKEIDTLEPGTWINLVNPTQNESLEIANTFDIDIADLRAPLDAEEMSRITIEDEYTLIIVDVPVTEERN 6v4k.1    --------------------------------------------------------------------------------  target    NRTYYVTIPLGIIITEETIITTCLEPLPVLDVFINRRLRNFYTFMRSRFIFQILYRNAELYLTALRSIDRKSEQIESQLH 6v4k.1    --------------------------------------------------------------------------------  target    QSTRNEELIELMELEKTIVYFKASLKTNERVIKKLTSSTSNIKKYLEDEDLLEDTLIETQQAIEMADIYGNVLHSMTETF 6v4k.1    --------------------------------------------------------------------------------  target    ASIISNNQNNIMKTLALVTIVMSIPTMVFSAYGMNFKDNEIPLNGEPNAFWLIVFIAFAMSVSLTLYLIHKKWF 6v4k.1    -------------LLALFSVTMLAPALVALLYRDG--------------------------------------- ``` | | | | | | | | | | | | | | | | | | | | | | | | | | | | | | | | | | | | | | | | | | | | | | | | | |
|  | 6v4k.1.B | Trk system potassium uptake protein  *Structure of TrkH-TrkA in complex with ADP* | 0.01 |  | 31.82 | 0.07 | 254-275 | X-ray | 3.53 | hetero-4-4-mer | 4 x ADP | HHblits | 0.35 |
| ``` target    MKQVFLSTTTEFKEIDTLEPGTWINLVNPTQNESLEIANTFDIDIADLRAPLDAEEMSRITIEDEYTLIIVDVPVTEERN 6v4k.1    --------------------------------------------------------------------------------  target    NRTYYVTIPLGIIITEETIITTCLEPLPVLDVFINRRLRNFYTFMRSRFIFQILYRNAELYLTALRSIDRKSEQIESQLH 6v4k.1    --------------------------------------------------------------------------------  target    QSTRNEELIELMELEKTIVYFKASLKTNERVIKKLTSSTSNIKKYLEDEDLLEDTLIETQQAIEMADIYGNVLHSMTETF 6v4k.1    --------------------------------------------------------------------------------  target    ASIISNNQNNIMKTLALVTIVMSIPTMVFSAYGMNFKDNEIPLNGEPNAFWLIVFIAFAMSVSLTLYLIHKKWF 6v4k.1    -------------LLALFSVTMLAPALVALLYRDG--------------------------------------- ``` | | | | | | | | | | | | | | | | | | | | | | | | | | | | | | | | | | | | | | | | | | | | | | | | | |
|  | 6v4k.1.C | Trk system potassium uptake protein  *Structure of TrkH-TrkA in complex with ADP* | 0.01 |  | 31.82 | 0.07 | 254-275 | X-ray | 3.53 | hetero-4-4-mer | 4 x ADP | HHblits | 0.35 |
| ``` target    MKQVFLSTTTEFKEIDTLEPGTWINLVNPTQNESLEIANTFDIDIADLRAPLDAEEMSRITIEDEYTLIIVDVPVTEERN 6v4k.1    --------------------------------------------------------------------------------  target    NRTYYVTIPLGIIITEETIITTCLEPLPVLDVFINRRLRNFYTFMRSRFIFQILYRNAELYLTALRSIDRKSEQIESQLH 6v4k.1    --------------------------------------------------------------------------------  target    QSTRNEELIELMELEKTIVYFKASLKTNERVIKKLTSSTSNIKKYLEDEDLLEDTLIETQQAIEMADIYGNVLHSMTETF 6v4k.1    --------------------------------------------------------------------------------  target    ASIISNNQNNIMKTLALVTIVMSIPTMVFSAYGMNFKDNEIPLNGEPNAFWLIVFIAFAMSVSLTLYLIHKKWF 6v4k.1    -------------LLALFSVTMLAPALVALLYRDG--------------------------------------- ``` | | | | | | | | | | | | | | | | | | | | | | | | | | | | | | | | | | | | | | | | | | | | | | | | | |
|  | 6v4k.1.A | Trk system potassium uptake protein  *Structure of TrkH-TrkA in complex with ADP* | 0.01 |  | 31.82 | 0.07 | 254-275 | X-ray | 3.53 | hetero-4-4-mer | 4 x ADP | HHblits | 0.35 |
| ``` target    MKQVFLSTTTEFKEIDTLEPGTWINLVNPTQNESLEIANTFDIDIADLRAPLDAEEMSRITIEDEYTLIIVDVPVTEERN 6v4k.1    --------------------------------------------------------------------------------  target    NRTYYVTIPLGIIITEETIITTCLEPLPVLDVFINRRLRNFYTFMRSRFIFQILYRNAELYLTALRSIDRKSEQIESQLH 6v4k.1    --------------------------------------------------------------------------------  target    QSTRNEELIELMELEKTIVYFKASLKTNERVIKKLTSSTSNIKKYLEDEDLLEDTLIETQQAIEMADIYGNVLHSMTETF 6v4k.1    --------------------------------------------------------------------------------  target    ASIISNNQNNIMKTLALVTIVMSIPTMVFSAYGMNFKDNEIPLNGEPNAFWLIVFIAFAMSVSLTLYLIHKKWF 6v4k.1    -------------LLALFSVTMLAPALVALLYRDG--------------------------------------- ``` | | | | | | | | | | | | | | | | | | | | | | | | | | | | | | | | | | | | | | | | | | | | | | | | | |
|  | 6yqf.1.A | Synaptonemal complex central element protein 2  *Crystal structure of the SYCE2-TEX12 delta-Ctip complex in a 4:4 assembly* | 0.00 |  | 17.39 | 0.07 | 166-188 | X-ray | 3.33 | hetero-2-2-mer |  | HHblits | 0.31 |
| ``` target    MKQVFLSTTTEFKEIDTLEPGTWINLVNPTQNESLEIANTFDIDIADLRAPLDAEEMSRITIEDEYTLIIVDVPVTEERN 6yqf.1    --------------------------------------------------------------------------------  target    NRTYYVTIPLGIIITEETIITTCLEPLPVLDVFINRRLRNFYTFMRSRFIFQILYRNAELYLTALRSIDRKSEQIESQLH 6yqf.1    --------------------------------------------------------------------------------  target    QSTRNEELIELMELEKTIVYFKASLKTNERVIKKLTSSTSNIKKYLEDEDLLEDTLIETQQAIEMADIYGNVLHSMTETF 6yqf.1    -----QKMAKISHLETELKQVCHSVETV----------------------------------------------------  target    ASIISNNQNNIMKTLALVTIVMSIPTMVFSAYGMNFKDNEIPLNGEPNAFWLIVFIAFAMSVSLTLYLIHKKWF 6yqf.1    -------------------------------------------------------------------------- ``` | | | | | | | | | | | | | | | | | | | | | | | | | | | | | | | | | | | | | | | | | | | | | | | | | |
|  | 6r17.1.B | Synaptonemal complex central element protein 2  *Crystal structure of the SYCE2-TEX12 delta-Ctip 2:2 complex* | 0.00 |  | 17.39 | 0.07 | 166-188 | X-ray | 2.42 | hetero-2-2-mer |  | HHblits | 0.31 |
| ``` target    MKQVFLSTTTEFKEIDTLEPGTWINLVNPTQNESLEIANTFDIDIADLRAPLDAEEMSRITIEDEYTLIIVDVPVTEERN 6r17.1    --------------------------------------------------------------------------------  target    NRTYYVTIPLGIIITEETIITTCLEPLPVLDVFINRRLRNFYTFMRSRFIFQILYRNAELYLTALRSIDRKSEQIESQLH 6r17.1    --------------------------------------------------------------------------------  target    QSTRNEELIELMELEKTIVYFKASLKTNERVIKKLTSSTSNIKKYLEDEDLLEDTLIETQQAIEMADIYGNVLHSMTETF 6r17.1    -----QKMAKISHLETELKQVCHSVETV----------------------------------------------------  target    ASIISNNQNNIMKTLALVTIVMSIPTMVFSAYGMNFKDNEIPLNGEPNAFWLIVFIAFAMSVSLTLYLIHKKWF 6r17.1    -------------------------------------------------------------------------- ``` | | | | | | | | | | | | | | | | | | | | | | | | | | | | | | | | | | | | | | | | | | | | | | | | | |
|  | 6r17.1.A | Synaptonemal complex central element protein 2  *Crystal structure of the SYCE2-TEX12 delta-Ctip 2:2 complex* | 0.00 |  | 17.39 | 0.07 | 166-188 | X-ray | 2.42 | hetero-2-2-mer |  | HHblits | 0.31 |
| ``` target    MKQVFLSTTTEFKEIDTLEPGTWINLVNPTQNESLEIANTFDIDIADLRAPLDAEEMSRITIEDEYTLIIVDVPVTEERN 6r17.1    --------------------------------------------------------------------------------  target    NRTYYVTIPLGIIITEETIITTCLEPLPVLDVFINRRLRNFYTFMRSRFIFQILYRNAELYLTALRSIDRKSEQIESQLH 6r17.1    --------------------------------------------------------------------------------  target    QSTRNEELIELMELEKTIVYFKASLKTNERVIKKLTSSTSNIKKYLEDEDLLEDTLIETQQAIEMADIYGNVLHSMTETF 6r17.1    -----QKMAKISHLETELKQVCHSVETV----------------------------------------------------  target    ASIISNNQNNIMKTLALVTIVMSIPTMVFSAYGMNFKDNEIPLNGEPNAFWLIVFIAFAMSVSLTLYLIHKKWF 6r17.1    -------------------------------------------------------------------------- ``` | | | | | | | | | | | | | | | | | | | | | | | | | | | | | | | | | | | | | | | | | | | | | | | | | |
|  | 6yqf.1.B | Synaptonemal complex central element protein 2  *Crystal structure of the SYCE2-TEX12 delta-Ctip complex in a 4:4 assembly* | 0.00 |  | 17.39 | 0.07 | 166-188 | X-ray | 3.33 | hetero-2-2-mer |  | HHblits | 0.31 |
| ``` target    MKQVFLSTTTEFKEIDTLEPGTWINLVNPTQNESLEIANTFDIDIADLRAPLDAEEMSRITIEDEYTLIIVDVPVTEERN 6yqf.1    --------------------------------------------------------------------------------  target    NRTYYVTIPLGIIITEETIITTCLEPLPVLDVFINRRLRNFYTFMRSRFIFQILYRNAELYLTALRSIDRKSEQIESQLH 6yqf.1    --------------------------------------------------------------------------------  target    QSTRNEELIELMELEKTIVYFKASLKTNERVIKKLTSSTSNIKKYLEDEDLLEDTLIETQQAIEMADIYGNVLHSMTETF 6yqf.1    -----QKMAKISHLETELKQVCHSVETV----------------------------------------------------  target    ASIISNNQNNIMKTLALVTIVMSIPTMVFSAYGMNFKDNEIPLNGEPNAFWLIVFIAFAMSVSLTLYLIHKKWF 6yqf.1    -------------------------------------------------------------------------- ``` | | | | | | | | | | | | | | | | | | | | | | | | | | | | | | | | | | | | | | | | | | | | | | | | | |
|  | 3pjz.1.A | Potassium uptake protein TrkH  *Crystal Structure of the Potassium Transporter TrkH from Vibrio parahaemolyticus* | 0.00 |  | 31.82 | 0.07 | 253-274 | X-ray | 3.51 | homo-dimer | 2 x K | HHblits | 0.34 |
| ``` target    MKQVFLSTTTEFKEIDTLEPGTWINLVNPTQNESLEIANTFDIDIADLRAPLDAEEMSRITIEDEYTLIIVDVPVTEERN 3pjz.1    --------------------------------------------------------------------------------  target    NRTYYVTIPLGIIITEETIITTCLEPLPVLDVFINRRLRNFYTFMRSRFIFQILYRNAELYLTALRSIDRKSEQIESQLH 3pjz.1    --------------------------------------------------------------------------------  target    QSTRNEELIELMELEKTIVYFKASLKTNERVIKKLTSSTSNIKKYLEDEDLLEDTLIETQQAIEMADIYGNVLHSMTETF 3pjz.1    --------------------------------------------------------------------------------  target    ASIISNNQNNIMKTLALVTIVMSIPTMVFSAYGMNFKDNEIPLNGEPNAFWLIVFIAFAMSVSLTLYLIHKKWF 3pjz.1    ------------LLLALFSVTMLAPALVALLYRD---------------------------------------- ``` | | | | | | | | | | | | | | | | | | | | | | | | | | | | | | | | | | | | | | | | | | | | | | | | | |
|  | 6s7o.1.F | Dolichyl-diphosphooligosaccharide--protein glycosyltransferase subunit 2  *Cryo-EM structure of human oligosaccharyltransferase complex OST-A* | 0.00 |  | 18.18 | 0.07 | 261-284 | EM | 0.00 | hetero-1-1-1-1-1-1-… | 9 x KZB, 7 x EGY, 2 x MG, 1 x KZE, 1 x NAG-NAG-BMA, 2 x NAG-NAG-BMA-MAN-MAN-MAN-MAN-MAN | HHblits | 0.33 |
| ``` target    MKQVFLSTTTEFKEIDTLEPGTWINLVNPTQNESLEIANTFDIDIADLRAPLDAEEMSRITIEDEYTLIIVDVPVTEERN 6s7o.1    --------------------------------------------------------------------------------  target    NRTYYVTIPLGIIITEETIITTCLEPLPVLDVFINRRLRNFYTFMRSRFIFQILYRNAELYLTALRSIDRKSEQIESQLH 6s7o.1    --------------------------------------------------------------------------------  target    QSTRNEELIELMELEKTIVYFKASLKTNERVIKKLTSSTSNIKKYLEDEDLLEDTLIETQQAIEMADIYGNVLHSMTETF 6s7o.1    --------------------------------------------------------------------------------  target    ASIISNNQNNIMKTLALVTIVMSIPTMVFSAY---GMNFKDNEIPLNGEPNAFWLIVFIAFAMSVSLTLYLIHKKWF 6s7o.1    --------------------ILSPLLLLFALWIRIGANVSN--FTFA------------------------------ ``` | | | | | | | | | | | | | | | | | | | | | | | | | | | | | | | | | | | | | | | | | | | | | | | | | |
|  | 6s7t.1.F | Dolichyl-diphosphooligosaccharide--protein glycosyltransferase subunit 2  *Cryo-EM structure of human oligosaccharyltransferase complex OST-B* | 0.01 |  | 18.18 | 0.07 | 261-284 | EM | 0.00 | hetero-1-1-1-1-1-1-… | 10 x EGY, 13 x KZB, 2 x MG, 1 x 0K3, 1 x ALA-ALA-ASN-ALA-THR-ALA-ALA, 2 x NAG-NAG, 2 x NAG-NAG-BMA-MAN-MAN-MAN-MAN-MAN | HHblits | 0.33 |
| ``` target    MKQVFLSTTTEFKEIDTLEPGTWINLVNPTQNESLEIANTFDIDIADLRAPLDAEEMSRITIEDEYTLIIVDVPVTEERN 6s7t.1    --------------------------------------------------------------------------------  target    NRTYYVTIPLGIIITEETIITTCLEPLPVLDVFINRRLRNFYTFMRSRFIFQILYRNAELYLTALRSIDRKSEQIESQLH 6s7t.1    --------------------------------------------------------------------------------  target    QSTRNEELIELMELEKTIVYFKASLKTNERVIKKLTSSTSNIKKYLEDEDLLEDTLIETQQAIEMADIYGNVLHSMTETF 6s7t.1    --------------------------------------------------------------------------------  target    ASIISNNQNNIMKTLALVTIVMSIPTMVFSAY---GMNFKDNEIPLNGEPNAFWLIVFIAFAMSVSLTLYLIHKKWF 6s7t.1    --------------------ILSPLLLLFALWIRIGANVSN--FTFA------------------------------ ``` | | | | | | | | | | | | | | | | | | | | | | | | | | | | | | | | | | | | | | | | | | | | | | | | | |
|  | 7jr7.1.A | ATP-binding cassette sub-family G member 5  *Cryo-EM structure of ABCG5/G8 in complex with Fab 2E10 and 11F4* | 0.00 |  | 12.50 | 0.08 | 248-271 | EM | 0.00 | hetero-1-1-1-1-1-1-… |  | HHblits | 0.26 |
| ``` target    MKQVFLSTTTEFKEIDTLEPGTWINLVNPTQNESLEIANTFDIDIADLRAPLDAEEMSRITIEDEYTLIIVDVPVTEERN 7jr7.1    --------------------------------------------------------------------------------  target    NRTYYVTIPLGIIITEETIITTCLEPLPVLDVFINRRLRNFYTFMRSRFIFQILYRNAELYLTALRSIDRKSEQIESQLH 7jr7.1    --------------------------------------------------------------------------------  target    QSTRNEELIELMELEKTIVYFKASLKTNERVIKKLTSSTSNIKKYLEDEDLLEDTLIETQQAIEMADIYGNVLHSMTETF 7jr7.1    --------------------------------------------------------------------------------  target    ASIISNNQNNIMKTLALVTIVMSIPTMVF-SAYGMNFKDNEIPLNGEPNAFWLIVFIAFAMSVSLTLYLIHKKWF 7jr7.1    -------VQNPNIVNSVVALLSIAGVLVGSGF------------------------------------------- ``` | | | | | | | | | | | | | | | | | | | | | | | | | | | | | | | | | | | | | | | | | | | | | | | | | |
|  | 7kzm.1.Q | Dynein gamma chain, flagellar outer arm  *Outer dynein arm bound to doublet microtubules from C. reinhardtii* | 0.00 |  | 12.50 | 0.08 | 173-196 | EM | 0.00 | hetero-8-6-1-1-1-1-… | 7 x GTP, 7 x MG, 8 x GDP | HHblits | 0.25 |
| ``` target    MKQVFLSTTTEFKEIDTLEPGTWINLVNPTQNESLEIANTFDIDIADLRAPLDAEEMSRITIEDEYTLIIVDVPVTEERN 7kzm.1    --------------------------------------------------------------------------------  target    NRTYYVTIPLGIIITEETIITTCLEPLPVLDVFINRRLRNFYTFMRSRFIFQILYRNAELYLTALRSIDRKSEQIESQLH 7kzm.1    --------------------------------------------------------------------------------  target    QSTRNEELIELMELEKTIVYFKASLKTNERVIKKLTSSTSNIKKYLEDEDLLEDTLIETQQAIEMADIYGNVLHSMTETF 7kzm.1    ------------EVREKESEIDNLIGPIEEMYGLLM--------------------------------------------  target    ASIISNNQNNIMKTLALVTIVMSIPTMVFSAYGMNFKDNEIPLNGEPNAFWLIVFIAFAMSVSLTLYLIHKKWF 7kzm.1    -------------------------------------------------------------------------- ``` | | | | | | | | | | | | | | | | | | | | | | | | | | | | | | | | | | | | | | | | | | | | | | | | | |
|  | 6tqe.1.A | ABC transporter ATP-binding protein/permease  *The structure of ABC transporter Rv1819c without addition of substrate* | 0.00 |  | 8.70 | 0.07 | 255-277 | EM | 0.00 | homo-dimer | 2 x ATP, 2 x MG | HHblits | 0.28 |
| ``` target    MKQVFLSTTTEFKEIDTLEPGTWINLVNPTQNESLEIANTFDIDIADLRAPLDAEEMSRITIEDEYTLIIVDVPVTEERN 6tqe.1    --------------------------------------------------------------------------------  target    NRTYYVTIPLGIIITEETIITTCLEPLPVLDVFINRRLRNFYTFMRSRFIFQILYRNAELYLTALRSIDRKSEQIESQLH 6tqe.1    --------------------------------------------------------------------------------  target    QSTRNEELIELMELEKTIVYFKASLKTNERVIKKLTSSTSNIKKYLEDEDLLEDTLIETQQAIEMADIYGNVLHSMTETF 6tqe.1    --------------------------------------------------------------------------------  target    ASIISNNQNNIMKTLALVTIVMSIPTMVFSAYGMNFKDNEIPLNGEPNAFWLIVFIAFAMSVSLTLYLIHKKWF 6tqe.1    --------------FGAVQSIISVISFTAILWNLSGT------------------------------------- ``` | | | | | | | | | | | | | | | | | | | | | | | | | | | | | | | | | | | | | | | | | | | | | | | | | |
|  | 6tqf.1.A | ABC transporter ATP-binding protein/permease  *The structure of ABC transporter Rv1819c in AMP-PNP bound state* | 0.00 |  | 8.70 | 0.07 | 255-277 | EM | 0.00 | homo-dimer | 10 x LMT, 2 x MG, 2 x ANP | HHblits | 0.28 |
| ``` target    MKQVFLSTTTEFKEIDTLEPGTWINLVNPTQNESLEIANTFDIDIADLRAPLDAEEMSRITIEDEYTLIIVDVPVTEERN 6tqf.1    --------------------------------------------------------------------------------  target    NRTYYVTIPLGIIITEETIITTCLEPLPVLDVFINRRLRNFYTFMRSRFIFQILYRNAELYLTALRSIDRKSEQIESQLH 6tqf.1    --------------------------------------------------------------------------------  target    QSTRNEELIELMELEKTIVYFKASLKTNERVIKKLTSSTSNIKKYLEDEDLLEDTLIETQQAIEMADIYGNVLHSMTETF 6tqf.1    --------------------------------------------------------------------------------  target    ASIISNNQNNIMKTLALVTIVMSIPTMVFSAYGMNFKDNEIPLNGEPNAFWLIVFIAFAMSVSLTLYLIHKKWF 6tqf.1    --------------FGAVQSIISVISFTAILWNLSGT------------------------------------- ``` | | | | | | | | | | | | | | | | | | | | | | | | | | | | | | | | | | | | | | | | | | | | | | | | | |
|  | 5zgg.1.A | Tumor necrosis factor receptor superfamily member 16  *NMR structure of p75NTR transmembrane domain in complex with NSC49652* | 0.00 |  | 35.00 | 0.06 | 294-313 | NMR | 0.00 | homo-dimer | 1 x 9F6 | HHblits | 0.38 |
| ``` target    MKQVFLSTTTEFKEIDTLEPGTWINLVNPTQNESLEIANTFDIDIADLRAPLDAEEMSRITIEDEYTLIIVDVPVTEERN 5zgg.1    --------------------------------------------------------------------------------  target    NRTYYVTIPLGIIITEETIITTCLEPLPVLDVFINRRLRNFYTFMRSRFIFQILYRNAELYLTALRSIDRKSEQIESQLH 5zgg.1    --------------------------------------------------------------------------------  target    QSTRNEELIELMELEKTIVYFKASLKTNERVIKKLTSSTSNIKKYLEDEDLLEDTLIETQQAIEMADIYGNVLHSMTETF 5zgg.1    --------------------------------------------------------------------------------  target    ASIISNNQNNIMKTLALVTIVMSIPTMVFSAYGMNFKDNEIPLNGEPNAFWLIVFIAFAMSVSLTLYLIHKKWF 5zgg.1    -----------------------------------------------------CSILAAVVVGLVAYIAFKRW- ``` | | | | | | | | | | | | | | | | | | | | | | | | | | | | | | | | | | | | | | | | | | | | | | | | | |
|  | 5zgg.1.B | Tumor necrosis factor receptor superfamily member 16  *NMR structure of p75NTR transmembrane domain in complex with NSC49652* | 0.00 |  | 35.00 | 0.06 | 294-313 | NMR | 0.00 | homo-dimer | 1 x 9F6 | HHblits | 0.38 |
| ``` target    MKQVFLSTTTEFKEIDTLEPGTWINLVNPTQNESLEIANTFDIDIADLRAPLDAEEMSRITIEDEYTLIIVDVPVTEERN 5zgg.1    --------------------------------------------------------------------------------  target    NRTYYVTIPLGIIITEETIITTCLEPLPVLDVFINRRLRNFYTFMRSRFIFQILYRNAELYLTALRSIDRKSEQIESQLH 5zgg.1    --------------------------------------------------------------------------------  target    QSTRNEELIELMELEKTIVYFKASLKTNERVIKKLTSSTSNIKKYLEDEDLLEDTLIETQQAIEMADIYGNVLHSMTETF 5zgg.1    --------------------------------------------------------------------------------  target    ASIISNNQNNIMKTLALVTIVMSIPTMVFSAYGMNFKDNEIPLNGEPNAFWLIVFIAFAMSVSLTLYLIHKKWF 5zgg.1    -----------------------------------------------------CSILAAVVVGLVAYIAFKRW- ``` | | | | | | | | | | | | | | | | | | | | | | | | | | | | | | | | | | | | | | | | | | | | | | | | | |
|  | 2mic.1.A | Tumor necrosis factor receptor superfamily member 16  *NMR structure of p75 transmembrane domain in DPC micelles* | 0.00 |  | 35.00 | 0.06 | 294-313 | NMR | 0.00 | homo-dimer |  | HHblits | 0.38 |
| ``` target    MKQVFLSTTTEFKEIDTLEPGTWINLVNPTQNESLEIANTFDIDIADLRAPLDAEEMSRITIEDEYTLIIVDVPVTEERN 2mic.1    --------------------------------------------------------------------------------  target    NRTYYVTIPLGIIITEETIITTCLEPLPVLDVFINRRLRNFYTFMRSRFIFQILYRNAELYLTALRSIDRKSEQIESQLH 2mic.1    --------------------------------------------------------------------------------  target    QSTRNEELIELMELEKTIVYFKASLKTNERVIKKLTSSTSNIKKYLEDEDLLEDTLIETQQAIEMADIYGNVLHSMTETF 2mic.1    --------------------------------------------------------------------------------  target    ASIISNNQNNIMKTLALVTIVMSIPTMVFSAYGMNFKDNEIPLNGEPNAFWLIVFIAFAMSVSLTLYLIHKKWF 2mic.1    -----------------------------------------------------CSILAAVVVGLVAYIAFKRW- ``` | | | | | | | | | | | | | | | | | | | | | | | | | | | | | | | | | | | | | | | | | | | | | | | | | |
|  | 2mic.1.B | Tumor necrosis factor receptor superfamily member 16  *NMR structure of p75 transmembrane domain in DPC micelles* | 0.00 |  | 35.00 | 0.06 | 294-313 | NMR | 0.00 | homo-dimer |  | HHblits | 0.38 |
| ``` target    MKQVFLSTTTEFKEIDTLEPGTWINLVNPTQNESLEIANTFDIDIADLRAPLDAEEMSRITIEDEYTLIIVDVPVTEERN 2mic.1    --------------------------------------------------------------------------------  target    NRTYYVTIPLGIIITEETIITTCLEPLPVLDVFINRRLRNFYTFMRSRFIFQILYRNAELYLTALRSIDRKSEQIESQLH 2mic.1    --------------------------------------------------------------------------------  target    QSTRNEELIELMELEKTIVYFKASLKTNERVIKKLTSSTSNIKKYLEDEDLLEDTLIETQQAIEMADIYGNVLHSMTETF 2mic.1    --------------------------------------------------------------------------------  target    ASIISNNQNNIMKTLALVTIVMSIPTMVFSAYGMNFKDNEIPLNGEPNAFWLIVFIAFAMSVSLTLYLIHKKWF 2mic.1    -----------------------------------------------------CSILAAVVVGLVAYIAFKRW- ``` | | | | | | | | | | | | | | | | | | | | | | | | | | | | | | | | | | | | | | | | | | | | | | | | | |
|  | 6eti.1.A | ATP-binding cassette sub-family G member 2  *Structure of inhibitor-bound ABCG2* | 0.00 |  | 13.04 | 0.07 | 250-272 | EM | 0.00 | hetero-2-2-2-mer | 2 x BWQ, 2 x NAG-NAG | HHblits | 0.26 |
| ``` target    MKQVFLSTTTEFKEIDTLEPGTWINLVNPTQNESLEIANTFDIDIADLRAPLDAEEMSRITIEDEYTLIIVDVPVTEERN 6eti.1    --------------------------------------------------------------------------------  target    NRTYYVTIPLGIIITEETIITTCLEPLPVLDVFINRRLRNFYTFMRSRFIFQILYRNAELYLTALRSIDRKSEQIESQLH 6eti.1    --------------------------------------------------------------------------------  target    QSTRNEELIELMELEKTIVYFKASLKTNERVIKKLTSSTSNIKKYLEDEDLLEDTLIETQQAIEMADIYGNVLHSMTETF 6eti.1    --------------------------------------------------------------------------------  target    ASIISNNQNNIMKTLALVTIVMSIPTMVFSAYGMNFKDNEIPLNGEPNAFWLIVFIAFAMSVSLTLYLIHKKWF 6eti.1    ---------VVSVATLLMTICFVFMMIFSGLL------------------------------------------ ``` | | | | | | | | | | | | | | | | | | | | | | | | | | | | | | | | | | | | | | | | | | | | | | | | | |
|  | 6hij.1.A | ATP-binding cassette sub-family G member 2  *Cryo-EM structure of the human ABCG2-MZ29-Fab complex with cholesterol and PE lipids docked* | 0.00 |  | 13.04 | 0.07 | 250-272 | EM | 0.00 | homo-dimer | 8 x PEE, 10 x CLR, 2 x BWQ | HHblits | 0.26 |
| ``` target    MKQVFLSTTTEFKEIDTLEPGTWINLVNPTQNESLEIANTFDIDIADLRAPLDAEEMSRITIEDEYTLIIVDVPVTEERN 6hij.1    --------------------------------------------------------------------------------  target    NRTYYVTIPLGIIITEETIITTCLEPLPVLDVFINRRLRNFYTFMRSRFIFQILYRNAELYLTALRSIDRKSEQIESQLH 6hij.1    --------------------------------------------------------------------------------  target    QSTRNEELIELMELEKTIVYFKASLKTNERVIKKLTSSTSNIKKYLEDEDLLEDTLIETQQAIEMADIYGNVLHSMTETF 6hij.1    --------------------------------------------------------------------------------  target    ASIISNNQNNIMKTLALVTIVMSIPTMVFSAYGMNFKDNEIPLNGEPNAFWLIVFIAFAMSVSLTLYLIHKKWF 6hij.1    ---------VVSVATLLMTICFVFMMIFSGLL------------------------------------------ ``` | | | | | | | | | | | | | | | | | | | | | | | | | | | | | | | | | | | | | | | | | | | | | | | | | |
|  | 6vxi.1.A | Broad substrate specificity ATP-binding cassette transporter ABCG2  *Structure of ABCG2 bound to mitoxantrone* | 0.00 |  | 13.04 | 0.07 | 250-272 | EM | 0.00 | homo-dimer | 2 x CLR, 1 x MIX | HHblits | 0.26 |
| ``` target    MKQVFLSTTTEFKEIDTLEPGTWINLVNPTQNESLEIANTFDIDIADLRAPLDAEEMSRITIEDEYTLIIVDVPVTEERN 6vxi.1    --------------------------------------------------------------------------------  target    NRTYYVTIPLGIIITEETIITTCLEPLPVLDVFINRRLRNFYTFMRSRFIFQILYRNAELYLTALRSIDRKSEQIESQLH 6vxi.1    --------------------------------------------------------------------------------  target    QSTRNEELIELMELEKTIVYFKASLKTNERVIKKLTSSTSNIKKYLEDEDLLEDTLIETQQAIEMADIYGNVLHSMTETF 6vxi.1    --------------------------------------------------------------------------------  target    ASIISNNQNNIMKTLALVTIVMSIPTMVFSAYGMNFKDNEIPLNGEPNAFWLIVFIAFAMSVSLTLYLIHKKWF 6vxi.1    ---------VVSVATLLMTICFVFMMIFSGLL------------------------------------------ ``` | | | | | | | | | | | | | | | | | | | | | | | | | | | | | | | | | | | | | | | | | | | | | | | | | |
|  | 6vxh.1.B | Broad substrate specificity ATP-binding cassette transporter ABCG2  *Structure of ABCG2 bound to imatinib* | 0.00 |  | 13.04 | 0.07 | 250-272 | EM | 0.00 | homo-dimer | 2 x CLR, 1 x STI | HHblits | 0.26 |
| ``` target    MKQVFLSTTTEFKEIDTLEPGTWINLVNPTQNESLEIANTFDIDIADLRAPLDAEEMSRITIEDEYTLIIVDVPVTEERN 6vxh.1    --------------------------------------------------------------------------------  target    NRTYYVTIPLGIIITEETIITTCLEPLPVLDVFINRRLRNFYTFMRSRFIFQILYRNAELYLTALRSIDRKSEQIESQLH 6vxh.1    --------------------------------------------------------------------------------  target    QSTRNEELIELMELEKTIVYFKASLKTNERVIKKLTSSTSNIKKYLEDEDLLEDTLIETQQAIEMADIYGNVLHSMTETF 6vxh.1    --------------------------------------------------------------------------------  target    ASIISNNQNNIMKTLALVTIVMSIPTMVFSAYGMNFKDNEIPLNGEPNAFWLIVFIAFAMSVSLTLYLIHKKWF 6vxh.1    ---------VVSVATLLMTICFVFMMIFSGLL------------------------------------------ ``` | | | | | | | | | | | | | | | | | | | | | | | | | | | | | | | | | | | | | | | | | | | | | | | | | |
|  | 6vxf.1.B | Broad substrate specificity ATP-binding cassette transporter ABCG2  *Structure of apo-closed ABCG2* | 0.01 |  | 13.04 | 0.07 | 250-272 | EM | 0.00 | homo-dimer |  | HHblits | 0.26 |
| ``` target    MKQVFLSTTTEFKEIDTLEPGTWINLVNPTQNESLEIANTFDIDIADLRAPLDAEEMSRITIEDEYTLIIVDVPVTEERN 6vxf.1    --------------------------------------------------------------------------------  target    NRTYYVTIPLGIIITEETIITTCLEPLPVLDVFINRRLRNFYTFMRSRFIFQILYRNAELYLTALRSIDRKSEQIESQLH 6vxf.1    --------------------------------------------------------------------------------  target    QSTRNEELIELMELEKTIVYFKASLKTNERVIKKLTSSTSNIKKYLEDEDLLEDTLIETQQAIEMADIYGNVLHSMTETF 6vxf.1    --------------------------------------------------------------------------------  target    ASIISNNQNNIMKTLALVTIVMSIPTMVFSAYGMNFKDNEIPLNGEPNAFWLIVFIAFAMSVSLTLYLIHKKWF 6vxf.1    ---------VVSVATLLMTICFVFMMIFSGLL------------------------------------------ ``` | | | | | | | | | | | | | | | | | | | | | | | | | | | | | | | | | | | | | | | | | | | | | | | | | |
|  | 7nez.1.A | ATP-binding cassette sub-family G member 2  *Structure of topotecan-bound ABCG2* | 0.00 |  | 13.04 | 0.07 | 250-272 | EM | 0.00 | hetero-2-2-2-mer | 1 x TTC, 2 x NAG | HHblits | 0.26 |
| ``` target    MKQVFLSTTTEFKEIDTLEPGTWINLVNPTQNESLEIANTFDIDIADLRAPLDAEEMSRITIEDEYTLIIVDVPVTEERN 7nez.1    --------------------------------------------------------------------------------  target    NRTYYVTIPLGIIITEETIITTCLEPLPVLDVFINRRLRNFYTFMRSRFIFQILYRNAELYLTALRSIDRKSEQIESQLH 7nez.1    --------------------------------------------------------------------------------  target    QSTRNEELIELMELEKTIVYFKASLKTNERVIKKLTSSTSNIKKYLEDEDLLEDTLIETQQAIEMADIYGNVLHSMTETF 7nez.1    --------------------------------------------------------------------------------  target    ASIISNNQNNIMKTLALVTIVMSIPTMVFSAYGMNFKDNEIPLNGEPNAFWLIVFIAFAMSVSLTLYLIHKKWF 7nez.1    ---------VVSVATLLMTICFVFMMIFSGLL------------------------------------------ ``` | | | | | | | | | | | | | | | | | | | | | | | | | | | | | | | | | | | | | | | | | | | | | | | | | |
|  | 7nfd.1.F | ATP-binding cassette sub-family G member 2  *Structure of mitoxantrone-bound ABCG2* | 0.00 |  | 13.04 | 0.07 | 250-272 | EM | 0.00 | hetero-2-2-2-mer | 1 x MIX, 2 x NAG-NAG | HHblits | 0.26 |
| ``` target    MKQVFLSTTTEFKEIDTLEPGTWINLVNPTQNESLEIANTFDIDIADLRAPLDAEEMSRITIEDEYTLIIVDVPVTEERN 7nfd.1    --------------------------------------------------------------------------------  target    NRTYYVTIPLGIIITEETIITTCLEPLPVLDVFINRRLRNFYTFMRSRFIFQILYRNAELYLTALRSIDRKSEQIESQLH 7nfd.1    --------------------------------------------------------------------------------  target    QSTRNEELIELMELEKTIVYFKASLKTNERVIKKLTSSTSNIKKYLEDEDLLEDTLIETQQAIEMADIYGNVLHSMTETF 7nfd.1    --------------------------------------------------------------------------------  target    ASIISNNQNNIMKTLALVTIVMSIPTMVFSAYGMNFKDNEIPLNGEPNAFWLIVFIAFAMSVSLTLYLIHKKWF 7nfd.1    ---------VVSVATLLMTICFVFMMIFSGLL------------------------------------------ ``` | | | | | | | | | | | | | | | | | | | | | | | | | | | | | | | | | | | | | | | | | | | | | | | | | |
|  | 7neq.1.F | ATP-binding cassette sub-family G member 2  *Structure of tariquidar-bound ABCG2* | 0.00 |  | 13.04 | 0.07 | 250-272 | EM | 0.00 | hetero-2-2-2-mer | 2 x NAG, 1 x U9N, 3 x CLR, 1 x R1H | HHblits | 0.26 |
| ``` target    MKQVFLSTTTEFKEIDTLEPGTWINLVNPTQNESLEIANTFDIDIADLRAPLDAEEMSRITIEDEYTLIIVDVPVTEERN 7neq.1    --------------------------------------------------------------------------------  target    NRTYYVTIPLGIIITEETIITTCLEPLPVLDVFINRRLRNFYTFMRSRFIFQILYRNAELYLTALRSIDRKSEQIESQLH 7neq.1    --------------------------------------------------------------------------------  target    QSTRNEELIELMELEKTIVYFKASLKTNERVIKKLTSSTSNIKKYLEDEDLLEDTLIETQQAIEMADIYGNVLHSMTETF 7neq.1    --------------------------------------------------------------------------------  target    ASIISNNQNNIMKTLALVTIVMSIPTMVFSAYGMNFKDNEIPLNGEPNAFWLIVFIAFAMSVSLTLYLIHKKWF 7neq.1    ---------VVSVATLLMTICFVFMMIFSGLL------------------------------------------ ``` | | | | | | | | | | | | | | | | | | | | | | | | | | | | | | | | | | | | | | | | | | | | | | | | | |
|  | 7neq.1.A | ATP-binding cassette sub-family G member 2  *Structure of tariquidar-bound ABCG2* | 0.00 |  | 13.04 | 0.07 | 250-272 | EM | 0.00 | hetero-2-2-2-mer | 2 x NAG, 1 x U9N, 3 x CLR, 1 x R1H | HHblits | 0.26 |
| ``` target    MKQVFLSTTTEFKEIDTLEPGTWINLVNPTQNESLEIANTFDIDIADLRAPLDAEEMSRITIEDEYTLIIVDVPVTEERN 7neq.1    --------------------------------------------------------------------------------  target    NRTYYVTIPLGIIITEETIITTCLEPLPVLDVFINRRLRNFYTFMRSRFIFQILYRNAELYLTALRSIDRKSEQIESQLH 7neq.1    --------------------------------------------------------------------------------  target    QSTRNEELIELMELEKTIVYFKASLKTNERVIKKLTSSTSNIKKYLEDEDLLEDTLIETQQAIEMADIYGNVLHSMTETF 7neq.1    --------------------------------------------------------------------------------  target    ASIISNNQNNIMKTLALVTIVMSIPTMVFSAYGMNFKDNEIPLNGEPNAFWLIVFIAFAMSVSLTLYLIHKKWF 7neq.1    ---------VVSVATLLMTICFVFMMIFSGLL------------------------------------------ ``` | | | | | | | | | | | | | | | | | | | | | | | | | | | | | | | | | | | | | | | | | | | | | | | | | |
|  | 5ijn.1.L | NUCLEAR PORE COMPLEX PROTEIN NUP54  *Composite structure of the inner ring of the human nuclear pore complex (32 copies of Nup205)* | 0.00 |  | 13.64 | 0.07 | 137-158 | EM | 0.00 | hetero-6-4-4-4-4-4-… |  | HHblits | 0.29 |
| ``` target    MKQVFLSTTTEFKEIDTLEPGTWINLVNPTQNESLEIANTFDIDIADLRAPLDAEEMSRITIEDEYTLIIVDVPVTEERN 5ijn.1    --------------------------------------------------------------------------------  target    NRTYYVTIPLGIIITEETIITTCLEPLPVLDVFINRRLRNFYTFMRSRFIFQILYRNAELYLTALRSIDRKSEQIESQLH 5ijn.1    --------------------------------------------------------MTKQHQTRLDIISEDISELQKN--  target    QSTRNEELIELMELEKTIVYFKASLKTNERVIKKLTSSTSNIKKYLEDEDLLEDTLIETQQAIEMADIYGNVLHSMTETF 5ijn.1    --------------------------------------------------------------------------------  target    ASIISNNQNNIMKTLALVTIVMSIPTMVFSAYGMNFKDNEIPLNGEPNAFWLIVFIAFAMSVSLTLYLIHKKWF 5ijn.1    -------------------------------------------------------------------------- ``` | | | | | | | | | | | | | | | | | | | | | | | | | | | | | | | | | | | | | | | | | | | | | | | | | |
|  | 5ijn.1.F | NUCLEAR PORE COMPLEX PROTEIN NUP54  *Composite structure of the inner ring of the human nuclear pore complex (32 copies of Nup205)* | 0.00 |  | 13.64 | 0.07 | 137-158 | EM | 0.00 | hetero-6-4-4-4-4-4-… |  | HHblits | 0.29 |
| ``` target    MKQVFLSTTTEFKEIDTLEPGTWINLVNPTQNESLEIANTFDIDIADLRAPLDAEEMSRITIEDEYTLIIVDVPVTEERN 5ijn.1    --------------------------------------------------------------------------------  target    NRTYYVTIPLGIIITEETIITTCLEPLPVLDVFINRRLRNFYTFMRSRFIFQILYRNAELYLTALRSIDRKSEQIESQLH 5ijn.1    --------------------------------------------------------MTKQHQTRLDIISEDISELQKN--  target    QSTRNEELIELMELEKTIVYFKASLKTNERVIKKLTSSTSNIKKYLEDEDLLEDTLIETQQAIEMADIYGNVLHSMTETF 5ijn.1    --------------------------------------------------------------------------------  target    ASIISNNQNNIMKTLALVTIVMSIPTMVFSAYGMNFKDNEIPLNGEPNAFWLIVFIAFAMSVSLTLYLIHKKWF 5ijn.1    -------------------------------------------------------------------------- ``` | | | | | | | | | | | | | | | | | | | | | | | | | | | | | | | | | | | | | | | | | | | | | | | | | |
|  | 5ijn.1.R | NUCLEAR PORE COMPLEX PROTEIN NUP54  *Composite structure of the inner ring of the human nuclear pore complex (32 copies of Nup205)* | 0.00 |  | 13.64 | 0.07 | 137-158 | EM | 0.00 | hetero-6-4-4-4-4-4-… |  | HHblits | 0.29 |
| ``` target    MKQVFLSTTTEFKEIDTLEPGTWINLVNPTQNESLEIANTFDIDIADLRAPLDAEEMSRITIEDEYTLIIVDVPVTEERN 5ijn.1    --------------------------------------------------------------------------------  target    NRTYYVTIPLGIIITEETIITTCLEPLPVLDVFINRRLRNFYTFMRSRFIFQILYRNAELYLTALRSIDRKSEQIESQLH 5ijn.1    --------------------------------------------------------MTKQHQTRLDIISEDISELQKN--  target    QSTRNEELIELMELEKTIVYFKASLKTNERVIKKLTSSTSNIKKYLEDEDLLEDTLIETQQAIEMADIYGNVLHSMTETF 5ijn.1    --------------------------------------------------------------------------------  target    ASIISNNQNNIMKTLALVTIVMSIPTMVFSAYGMNFKDNEIPLNGEPNAFWLIVFIAFAMSVSLTLYLIHKKWF 5ijn.1    -------------------------------------------------------------------------- ``` | | | | | | | | | | | | | | | | | | | | | | | | | | | | | | | | | | | | | | | | | | | | | | | | | |
|  | 6dnc.1.c | Peptide chain release factor 1  *E.coli RF1 bound to E.coli 70S ribosome in response to UAU sense A-site codon* | 0.00 |  | 14.29 | 0.07 | 141-161 | EM | 0.00 | hetero-1-1-1-1-1-1-… |  | HHblits | 0.33 |
| ``` target    MKQVFLSTTTEFKEIDTLEPGTWINLVNPTQNESLEIANTFDIDIADLRAPLDAEEMSRITIEDEYTLIIVDVPVTEERN 6dnc.1    --------------------------------------------------------------------------------  target    NRTYYVTIPLGIIITEETIITTCLEPLPVLDVFINRRLRNFYTFMRSRFIFQILYRNAELYLTALRSIDRKSEQIESQLH 6dnc.1    ------------------------------------------------------------IVAKLEALHERHEEVQALLG  target    QSTRNEELIELMELEKTIVYFKASLKTNERVIKKLTSSTSNIKKYLEDEDLLEDTLIETQQAIEMADIYGNVLHSMTETF 6dnc.1    D-------------------------------------------------------------------------------  target    ASIISNNQNNIMKTLALVTIVMSIPTMVFSAYGMNFKDNEIPLNGEPNAFWLIVFIAFAMSVSLTLYLIHKKWF 6dnc.1    -------------------------------------------------------------------------- ``` | | | | | | | | | | | | | | | | | | | | | | | | | | | | | | | | | | | | | | | | | | | | | | | | | |
|  | 1zbt.1.A | Peptide chain release factor 1  *Crystal structure of Peptide chain release factor 1 (RF-1) (SMU.1085) from Streptococcus mutans at 2.34 A resolution* | 0.00 |  | 14.29 | 0.07 | 141-161 | X-ray | 2.34 | monomer |  | HHblits | 0.31 |
| ``` target    MKQVFLSTTTEFKEIDTLEPGTWINLVNPTQNESLEIANTFDIDIADLRAPLDAEEMSRITIEDEYTLIIVDVPVTEERN 1zbt.1    --------------------------------------------------------------------------------  target    NRTYYVTIPLGIIITEETIITTCLEPLPVLDVFINRRLRNFYTFMRSRFIFQILYRNAELYLTALRSIDRKSEQIESQLH 1zbt.1    ------------------------------------------------------------IYDQLQAVEDRYEELGELLS  target    QSTRNEELIELMELEKTIVYFKASLKTNERVIKKLTSSTSNIKKYLEDEDLLEDTLIETQQAIEMADIYGNVLHSMTETF 1zbt.1    D-------------------------------------------------------------------------------  target    ASIISNNQNNIMKTLALVTIVMSIPTMVFSAYGMNFKDNEIPLNGEPNAFWLIVFIAFAMSVSLTLYLIHKKWF 1zbt.1    -------------------------------------------------------------------------- ``` | | | | | | | | | | | | | | | | | | | | | | | | | | | | | | | | | | | | | | | | | | | | | | | | | |
|  | 6gxn.1.r | Peptide chain release factor RF1  *Cryo-EM structure of an E. coli 70S ribosome in complex with RF3-GDPCP, RF1(GAQ) and Pint-tRNA (State III)* | 0.00 |  | 15.00 | 0.06 | 142-161 | EM | 0.00 | hetero-1-1-1-1-1-1-… | 1 x GCP, 1 x A-U-G-U-A-A-A | HHblits | 0.33 |
| ``` target    MKQVFLSTTTEFKEIDTLEPGTWINLVNPTQNESLEIANTFDIDIADLRAPLDAEEMSRITIEDEYTLIIVDVPVTEERN 6gxn.1    --------------------------------------------------------------------------------  target    NRTYYVTIPLGIIITEETIITTCLEPLPVLDVFINRRLRNFYTFMRSRFIFQILYRNAELYLTALRSIDRKSEQIESQLH 6gxn.1    -------------------------------------------------------------VAKLEALHERHEEVQALLG  target    QSTRNEELIELMELEKTIVYFKASLKTNERVIKKLTSSTSNIKKYLEDEDLLEDTLIETQQAIEMADIYGNVLHSMTETF 6gxn.1    D-------------------------------------------------------------------------------  target    ASIISNNQNNIMKTLALVTIVMSIPTMVFSAYGMNFKDNEIPLNGEPNAFWLIVFIAFAMSVSLTLYLIHKKWF 6gxn.1    -------------------------------------------------------------------------- ``` | | | | | | | | | | | | | | | | | | | | | | | | | | | | | | | | | | | | | | | | | | | | | | | | | |
|  | 6n9h.1.A | amantadine-binding protein  *De novo designed homo-trimeric amantadine-binding protein* | 0.00 |  | 20.00 | 0.06 | 139-158 | X-ray | 1.04 | homo-trimer | 3 x 308 | HHblits | 0.33 |
| ``` target    MKQVFLSTTTEFKEIDTLEPGTWINLVNPTQNESLEIANTFDIDIADLRAPLDAEEMSRITIEDEYTLIIVDVPVTEERN 6n9h.1    --------------------------------------------------------------------------------  target    NRTYYVTIPLGIIITEETIITTCLEPLPVLDVFINRRLRNFYTFMRSRFIFQILYRNAELYLTALRSIDRKSEQIESQLH 6n9h.1    ----------------------------------------------------------KQLERALRELKKSLDELERS--  target    QSTRNEELIELMELEKTIVYFKASLKTNERVIKKLTSSTSNIKKYLEDEDLLEDTLIETQQAIEMADIYGNVLHSMTETF 6n9h.1    --------------------------------------------------------------------------------  target    ASIISNNQNNIMKTLALVTIVMSIPTMVFSAYGMNFKDNEIPLNGEPNAFWLIVFIAFAMSVSLTLYLIHKKWF 6n9h.1    -------------------------------------------------------------------------- ``` | | | | | | | | | | | | | | | | | | | | | | | | | | | | | | | | | | | | | | | | | | | | | | | | | |
|  | 6naf.1.A | amantadine-binding protein  *De novo designed homo-trimeric amantadine-binding protein* | 0.00 |  | 20.00 | 0.06 | 139-158 | neutron diff. | 2.50 | homo-trimer | 3 x 308 | HHblits | 0.33 |
| ``` target    MKQVFLSTTTEFKEIDTLEPGTWINLVNPTQNESLEIANTFDIDIADLRAPLDAEEMSRITIEDEYTLIIVDVPVTEERN 6naf.1    --------------------------------------------------------------------------------  target    NRTYYVTIPLGIIITEETIITTCLEPLPVLDVFINRRLRNFYTFMRSRFIFQILYRNAELYLTALRSIDRKSEQIESQLH 6naf.1    ----------------------------------------------------------KQLERALRELKKSLDELERS--  target    QSTRNEELIELMELEKTIVYFKASLKTNERVIKKLTSSTSNIKKYLEDEDLLEDTLIETQQAIEMADIYGNVLHSMTETF 6naf.1    --------------------------------------------------------------------------------  target    ASIISNNQNNIMKTLALVTIVMSIPTMVFSAYGMNFKDNEIPLNGEPNAFWLIVFIAFAMSVSLTLYLIHKKWF 6naf.1    -------------------------------------------------------------------------- ``` | | | | | | | | | | | | | | | | | | | | | | | | | | | | | | | | | | | | | | | | | | | | | | | | | |
|  | 5zuv.1.A | Spike glycoprotein,Spike glycoprotein,inhibitor EK1  *Crystal Structure of the Human Coronavirus 229E HR1 motif in complex with pan-CoVs inhibitor EK1* | 0.00 |  | 14.29 | 0.07 | 177-197 | X-ray | 2.21 | homo-trimer |  | HHblits | 0.28 |
| ``` target    MKQVFLSTTTEFKEIDTLEPGTWINLVNPTQNESLEIANTFDIDIADLRAPLDAEEMSRITIEDEYTLIIVDVPVTEERN 5zuv.1    --------------------------------------------------------------------------------  target    NRTYYVTIPLGIIITEETIITTCLEPLPVLDVFINRRLRNFYTFMRSRFIFQILYRNAELYLTALRSIDRKSEQIESQLH 5zuv.1    --------------------------------------------------------------------------------  target    QSTRNEELIELMELEKTIVYFKASLKTNERVIKKLTSSTSNIKKYLEDEDLLEDTLIETQQAIEMADIYGNVLHSMTETF 5zuv.1    ----------------ALNKIQDVVNQQGNSLNHLTS-------------------------------------------  target    ASIISNNQNNIMKTLALVTIVMSIPTMVFSAYGMNFKDNEIPLNGEPNAFWLIVFIAFAMSVSLTLYLIHKKWF 5zuv.1    -------------------------------------------------------------------------- ``` | | | | | | | | | | | | | | | | | | | | | | | | | | | | | | | | | | | | | | | | | | | | | | | | | |
|  | 5zuv.1.B | Spike glycoprotein,Spike glycoprotein,inhibitor EK1  *Crystal Structure of the Human Coronavirus 229E HR1 motif in complex with pan-CoVs inhibitor EK1* | 0.00 |  | 14.29 | 0.07 | 177-197 | X-ray | 2.21 | homo-trimer |  | HHblits | 0.28 |
| ``` target    MKQVFLSTTTEFKEIDTLEPGTWINLVNPTQNESLEIANTFDIDIADLRAPLDAEEMSRITIEDEYTLIIVDVPVTEERN 5zuv.1    --------------------------------------------------------------------------------  target    NRTYYVTIPLGIIITEETIITTCLEPLPVLDVFINRRLRNFYTFMRSRFIFQILYRNAELYLTALRSIDRKSEQIESQLH 5zuv.1    --------------------------------------------------------------------------------  target    QSTRNEELIELMELEKTIVYFKASLKTNERVIKKLTSSTSNIKKYLEDEDLLEDTLIETQQAIEMADIYGNVLHSMTETF 5zuv.1    ----------------ALNKIQDVVNQQGNSLNHLTS-------------------------------------------  target    ASIISNNQNNIMKTLALVTIVMSIPTMVFSAYGMNFKDNEIPLNGEPNAFWLIVFIAFAMSVSLTLYLIHKKWF 5zuv.1    -------------------------------------------------------------------------- ``` | | | | | | | | | | | | | | | | | | | | | | | | | | | | | | | | | | | | | | | | | | | | | | | | | |
|  | 5zuv.1.C | Spike glycoprotein,Spike glycoprotein,inhibitor EK1  *Crystal Structure of the Human Coronavirus 229E HR1 motif in complex with pan-CoVs inhibitor EK1* | 0.00 |  | 14.29 | 0.07 | 177-197 | X-ray | 2.21 | homo-trimer |  | HHblits | 0.28 |
| ``` target    MKQVFLSTTTEFKEIDTLEPGTWINLVNPTQNESLEIANTFDIDIADLRAPLDAEEMSRITIEDEYTLIIVDVPVTEERN 5zuv.1    --------------------------------------------------------------------------------  target    NRTYYVTIPLGIIITEETIITTCLEPLPVLDVFINRRLRNFYTFMRSRFIFQILYRNAELYLTALRSIDRKSEQIESQLH 5zuv.1    --------------------------------------------------------------------------------  target    QSTRNEELIELMELEKTIVYFKASLKTNERVIKKLTSSTSNIKKYLEDEDLLEDTLIETQQAIEMADIYGNVLHSMTETF 5zuv.1    ----------------ALNKIQDVVNQQGNSLNHLTS-------------------------------------------  target    ASIISNNQNNIMKTLALVTIVMSIPTMVFSAYGMNFKDNEIPLNGEPNAFWLIVFIAFAMSVSLTLYLIHKKWF 5zuv.1    -------------------------------------------------------------------------- ``` | | | | | | | | | | | | | | | | | | | | | | | | | | | | | | | | | | | | | | | | | | | | | | | | | |
|  | 1u4q.1.A | Spectrin alpha chain, brain  *Crystal Structure of Repeats 15, 16 and 17 of Chicken Brain Alpha Spectrin* | 0.00 |  | 21.05 | 0.06 | 141-159 | X-ray | 2.50 | monomer |  | HHblits | 0.34 |
| ``` target    MKQVFLSTTTEFKEIDTLEPGTWINLVNPTQNESLEIANTFDIDIADLRAPLDAEEMSRITIEDEYTLIIVDVPVTEERN 1u4q.1    --------------------------------------------------------------------------------  target    NRTYYVTIPLGIIITEETIITTCLEPLPVLDVFINRRLRNFYTFMRSRFIFQILYRNAELYLTALRSIDRKSEQIESQLH 1u4q.1    ------------------------------------------------------------FNTGIKDFDFWLSEVEALL-  target    QSTRNEELIELMELEKTIVYFKASLKTNERVIKKLTSSTSNIKKYLEDEDLLEDTLIETQQAIEMADIYGNVLHSMTETF 1u4q.1    --------------------------------------------------------------------------------  target    ASIISNNQNNIMKTLALVTIVMSIPTMVFSAYGMNFKDNEIPLNGEPNAFWLIVFIAFAMSVSLTLYLIHKKWF 1u4q.1    -------------------------------------------------------------------------- ``` | | | | | | | | | | | | | | | | | | | | | | | | | | | | | | | | | | | | | | | | | | | | | | | | | |
|  | 1u4q.2.A | Spectrin alpha chain, brain  *Crystal Structure of Repeats 15, 16 and 17 of Chicken Brain Alpha Spectrin* | 0.01 |  | 21.05 | 0.06 | 141-159 | X-ray | 2.50 | monomer |  | HHblits | 0.34 |
| ``` target    MKQVFLSTTTEFKEIDTLEPGTWINLVNPTQNESLEIANTFDIDIADLRAPLDAEEMSRITIEDEYTLIIVDVPVTEERN 1u4q.2    --------------------------------------------------------------------------------  target    NRTYYVTIPLGIIITEETIITTCLEPLPVLDVFINRRLRNFYTFMRSRFIFQILYRNAELYLTALRSIDRKSEQIESQLH 1u4q.2    ------------------------------------------------------------FNTGIKDFDFWLSEVEALL-  target    QSTRNEELIELMELEKTIVYFKASLKTNERVIKKLTSSTSNIKKYLEDEDLLEDTLIETQQAIEMADIYGNVLHSMTETF 1u4q.2    --------------------------------------------------------------------------------  target    ASIISNNQNNIMKTLALVTIVMSIPTMVFSAYGMNFKDNEIPLNGEPNAFWLIVFIAFAMSVSLTLYLIHKKWF 1u4q.2    -------------------------------------------------------------------------- ``` | | | | | | | | | | | | | | | | | | | | | | | | | | | | | | | | | | | | | | | | | | | | | | | | | |
|  | 6o0c.1.A | Design construct XAA\_GVDQ mutant M4L  *NMR ensemble of computationally designed protein XAA\_GVDQ mutant M4L* | 0.00 |  | 21.05 | 0.06 | 180-198 | NMR | 0.00 | homo-trimer |  | HHblits | 0.33 |
| ``` target    MKQVFLSTTTEFKEIDTLEPGTWINLVNPTQNESLEIANTFDIDIADLRAPLDAEEMSRITIEDEYTLIIVDVPVTEERN 6o0c.1    --------------------------------------------------------------------------------  target    NRTYYVTIPLGIIITEETIITTCLEPLPVLDVFINRRLRNFYTFMRSRFIFQILYRNAELYLTALRSIDRKSEQIESQLH 6o0c.1    --------------------------------------------------------------------------------  target    QSTRNEELIELMELEKTIVYFKASLKTNERVIKKLTSSTSNIKKYLEDEDLLEDTLIETQQAIEMADIYGNVLHSMTETF 6o0c.1    -------------------DLKYSLERLREILERLEEN------------------------------------------  target    ASIISNNQNNIMKTLALVTIVMSIPTMVFSAYGMNFKDNEIPLNGEPNAFWLIVFIAFAMSVSLTLYLIHKKWF 6o0c.1    -------------------------------------------------------------------------- ``` | | | | | | | | | | | | | | | | | | | | | | | | | | | | | | | | | | | | | | | | | | | | | | | | | |
|  | 5xei.1.A | Chromosome partition protein Smc  *Crystal structure of the Smc head domain with a coiled coil and joint derived from Pyrococcus yayanosii* | 0.00 |  | 15.00 | 0.06 | 238-257 | X-ray | 2.60 | monomer |  | HHblits | 0.28 |
| ``` target    MKQVFLSTTTEFKEIDTLEPGTWINLVNPTQNESLEIANTFDIDIADLRAPLDAEEMSRITIEDEYTLIIVDVPVTEERN 5xei.1    --------------------------------------------------------------------------------  target    NRTYYVTIPLGIIITEETIITTCLEPLPVLDVFINRRLRNFYTFMRSRFIFQILYRNAELYLTALRSIDRKSEQIESQLH 5xei.1    --------------------------------------------------------------------------------  target    QSTRNEELIELMELEKTIVYFKASLKTNERVIKKLTSSTSNIKKYLEDEDLLEDTLIETQQAIEMADIYGNVLHSMTETF 5xei.1    -----------------------------------------------------------------------------MRT  target    ASIISNNQNNIMKTLALVTIVMSIPTMVFSAYGMNFKDNEIPLNGEPNAFWLIVFIAFAMSVSLTLYLIHKKWF 5xei.1    LEAIAKNFSELFAKLSP--------------------------------------------------------- ``` | | | | | | | | | | | | | | | | | | | | | | | | | | | | | | | | | | | | | | | | | | | | | | | | | |
|  | 3pdy.1.A | Plectin  *Structure of the third and fourth spectrin repeats of the plakin domain of plectin* | 0.01 |  | 15.00 | 0.06 | 140-159 | X-ray | 2.22 | monomer |  | HHblits | 0.27 |
| ``` target    MKQVFLSTTTEFKEIDTLEPGTWINLVNPTQNESLEIANTFDIDIADLRAPLDAEEMSRITIEDEYTLIIVDVPVTEERN 3pdy.1    --------------------------------------------------------------------------------  target    NRTYYVTIPLGIIITEETIITTCLEPLPVLDVFINRRLRNFYTFMRSRFIFQILYRNAELYLTALRSIDRKSEQIESQLH 3pdy.1    -----------------------------------------------------------STLRYLQDLLAWVEENQHRV-  target    QSTRNEELIELMELEKTIVYFKASLKTNERVIKKLTSSTSNIKKYLEDEDLLEDTLIETQQAIEMADIYGNVLHSMTETF 3pdy.1    --------------------------------------------------------------------------------  target    ASIISNNQNNIMKTLALVTIVMSIPTMVFSAYGMNFKDNEIPLNGEPNAFWLIVFIAFAMSVSLTLYLIHKKWF 3pdy.1    -------------------------------------------------------------------------- ``` | | | | | | | | | | | | | | | | | | | | | | | | | | | | | | | | | | | | | | | | | | | | | | | | | |
|  | 3pdy.2.A | Plectin  *Structure of the third and fourth spectrin repeats of the plakin domain of plectin* | 0.01 |  | 15.00 | 0.06 | 140-159 | X-ray | 2.22 | monomer |  | HHblits | 0.27 |
| ``` target    MKQVFLSTTTEFKEIDTLEPGTWINLVNPTQNESLEIANTFDIDIADLRAPLDAEEMSRITIEDEYTLIIVDVPVTEERN 3pdy.2    --------------------------------------------------------------------------------  target    NRTYYVTIPLGIIITEETIITTCLEPLPVLDVFINRRLRNFYTFMRSRFIFQILYRNAELYLTALRSIDRKSEQIESQLH 3pdy.2    -----------------------------------------------------------STLRYLQDLLAWVEENQHRV-  target    QSTRNEELIELMELEKTIVYFKASLKTNERVIKKLTSSTSNIKKYLEDEDLLEDTLIETQQAIEMADIYGNVLHSMTETF 3pdy.2    --------------------------------------------------------------------------------  target    ASIISNNQNNIMKTLALVTIVMSIPTMVFSAYGMNFKDNEIPLNGEPNAFWLIVFIAFAMSVSLTLYLIHKKWF 3pdy.2    -------------------------------------------------------------------------- ``` | | | | | | | | | | | | | | | | | | | | | | | | | | | | | | | | | | | | | | | | | | | | | | | | | |
|  | 6nz3.1.A | Design construct XAA\_GGHN  *Crystal structure of computationally designed protein XAA\_GGHN* | 0.00 |  | 22.22 | 0.06 | 181-198 | X-ray | 2.30 | homo-trimer |  | HHblits | 0.34 |
| ``` target    MKQVFLSTTTEFKEIDTLEPGTWINLVNPTQNESLEIANTFDIDIADLRAPLDAEEMSRITIEDEYTLIIVDVPVTEERN 6nz3.1    --------------------------------------------------------------------------------  target    NRTYYVTIPLGIIITEETIITTCLEPLPVLDVFINRRLRNFYTFMRSRFIFQILYRNAELYLTALRSIDRKSEQIESQLH 6nz3.1    --------------------------------------------------------------------------------  target    QSTRNEELIELMELEKTIVYFKASLKTNERVIKKLTSSTSNIKKYLEDEDLLEDTLIETQQAIEMADIYGNVLHSMTETF 6nz3.1    --------------------LKYSLERLREILERLEEN------------------------------------------  target    ASIISNNQNNIMKTLALVTIVMSIPTMVFSAYGMNFKDNEIPLNGEPNAFWLIVFIAFAMSVSLTLYLIHKKWF 6nz3.1    -------------------------------------------------------------------------- ``` | | | | | | | | | | | | | | | | | | | | | | | | | | | | | | | | | | | | | | | | | | | | | | | | | |
|  | 6nxm.1.A | Design construct XAA\_GVDQ  *Crystal structure of computationally designed protein XAA\_GVDQ* | 0.00 |  | 22.22 | 0.06 | 181-198 | X-ray | 2.20 | homo-hexamer |  | HHblits | 0.34 |
| ``` target    MKQVFLSTTTEFKEIDTLEPGTWINLVNPTQNESLEIANTFDIDIADLRAPLDAEEMSRITIEDEYTLIIVDVPVTEERN 6nxm.1    --------------------------------------------------------------------------------  target    NRTYYVTIPLGIIITEETIITTCLEPLPVLDVFINRRLRNFYTFMRSRFIFQILYRNAELYLTALRSIDRKSEQIESQLH 6nxm.1    --------------------------------------------------------------------------------  target    QSTRNEELIELMELEKTIVYFKASLKTNERVIKKLTSSTSNIKKYLEDEDLLEDTLIETQQAIEMADIYGNVLHSMTETF 6nxm.1    --------------------LKYSLERLREILERLEEN------------------------------------------  target    ASIISNNQNNIMKTLALVTIVMSIPTMVFSAYGMNFKDNEIPLNGEPNAFWLIVFIAFAMSVSLTLYLIHKKWF 6nxm.1    -------------------------------------------------------------------------- ``` | | | | | | | | | | | | | | | | | | | | | | | | | | | | | | | | | | | | | | | | | | | | | | | | | |
|  | 6ny8.1.A | Design construct XAA\_GVDQ  *Crystal structure of computationally designed protein XAA\_GVDQ with calcium* | 0.00 |  | 22.22 | 0.06 | 181-198 | X-ray | 2.30 | homo-trimer | 1 x CA | HHblits | 0.34 |
| ``` target    MKQVFLSTTTEFKEIDTLEPGTWINLVNPTQNESLEIANTFDIDIADLRAPLDAEEMSRITIEDEYTLIIVDVPVTEERN 6ny8.1    --------------------------------------------------------------------------------  target    NRTYYVTIPLGIIITEETIITTCLEPLPVLDVFINRRLRNFYTFMRSRFIFQILYRNAELYLTALRSIDRKSEQIESQLH 6ny8.1    --------------------------------------------------------------------------------  target    QSTRNEELIELMELEKTIVYFKASLKTNERVIKKLTSSTSNIKKYLEDEDLLEDTLIETQQAIEMADIYGNVLHSMTETF 6ny8.1    --------------------LKYSLERLREILERLEEN------------------------------------------  target    ASIISNNQNNIMKTLALVTIVMSIPTMVFSAYGMNFKDNEIPLNGEPNAFWLIVFIAFAMSVSLTLYLIHKKWF 6ny8.1    -------------------------------------------------------------------------- ``` | | | | | | | | | | | | | | | | | | | | | | | | | | | | | | | | | | | | | | | | | | | | | | | | | |
|  | 6hbu.1.A | ATP-binding cassette sub-family G member 2  *Cryo-EM structure of the ABCG2 E211Q mutant bound to ATP and Magnesium* | 0.00 |  | 15.79 | 0.06 | 254-272 | EM | 0.00 | homo-dimer | 2 x ATP, 2 x MG | HHblits | 0.27 |
| ``` target    MKQVFLSTTTEFKEIDTLEPGTWINLVNPTQNESLEIANTFDIDIADLRAPLDAEEMSRITIEDEYTLIIVDVPVTEERN 6hbu.1    --------------------------------------------------------------------------------  target    NRTYYVTIPLGIIITEETIITTCLEPLPVLDVFINRRLRNFYTFMRSRFIFQILYRNAELYLTALRSIDRKSEQIESQLH 6hbu.1    --------------------------------------------------------------------------------  target    QSTRNEELIELMELEKTIVYFKASLKTNERVIKKLTSSTSNIKKYLEDEDLLEDTLIETQQAIEMADIYGNVLHSMTETF 6hbu.1    --------------------------------------------------------------------------------  target    ASIISNNQNNIMKTLALVTIVMSIPTMVFSAYGMNFKDNEIPLNGEPNAFWLIVFIAFAMSVSLTLYLIHKKWF 6hbu.1    -------------ATLLMTICFVFMMIFSGLL------------------------------------------ ``` | | | | | | | | | | | | | | | | | | | | | | | | | | | | | | | | | | | | | | | | | | | | | | | | | |
|  | 5do7.1.A | ATP-binding cassette sub-family G member 5  *Crystal Structure of the Human Sterol Transporter ABCG5/ABCG8* | 0.00 |  | 10.53 | 0.06 | 251-269 | X-ray | 3.93 | hetero-1-1-mer |  | HHblits | 0.25 |
| ``` target    MKQVFLSTTTEFKEIDTLEPGTWINLVNPTQNESLEIANTFDIDIADLRAPLDAEEMSRITIEDEYTLIIVDVPVTEERN 5do7.1    --------------------------------------------------------------------------------  target    NRTYYVTIPLGIIITEETIITTCLEPLPVLDVFINRRLRNFYTFMRSRFIFQILYRNAELYLTALRSIDRKSEQIESQLH 5do7.1    --------------------------------------------------------------------------------  target    QSTRNEELIELMELEKTIVYFKASLKTNERVIKKLTSSTSNIKKYLEDEDLLEDTLIETQQAIEMADIYGNVLHSMTETF 5do7.1    --------------------------------------------------------------------------------  target    ASIISNNQNNIMKTLALVTIVMSIPTMVFSAYGMNFKDNEIPLNGEPNAFWLIVFIAFAMSVSLTLYLIHKKWF 5do7.1    ----------PNIVNSVVALLSIAGVLVG--------------------------------------------- ``` | | | | | | | | | | | | | | | | | | | | | | | | | | | | | | | | | | | | | | | | | | | | | | | | | |
|  | 5do7.2.B | ATP-binding cassette sub-family G member 5  *Crystal Structure of the Human Sterol Transporter ABCG5/ABCG8* | 0.00 |  | 10.53 | 0.06 | 251-269 | X-ray | 3.93 | hetero-1-1-mer |  | HHblits | 0.25 |
| ``` target    MKQVFLSTTTEFKEIDTLEPGTWINLVNPTQNESLEIANTFDIDIADLRAPLDAEEMSRITIEDEYTLIIVDVPVTEERN 5do7.2    --------------------------------------------------------------------------------  target    NRTYYVTIPLGIIITEETIITTCLEPLPVLDVFINRRLRNFYTFMRSRFIFQILYRNAELYLTALRSIDRKSEQIESQLH 5do7.2    --------------------------------------------------------------------------------  target    QSTRNEELIELMELEKTIVYFKASLKTNERVIKKLTSSTSNIKKYLEDEDLLEDTLIETQQAIEMADIYGNVLHSMTETF 5do7.2    --------------------------------------------------------------------------------  target    ASIISNNQNNIMKTLALVTIVMSIPTMVFSAYGMNFKDNEIPLNGEPNAFWLIVFIAFAMSVSLTLYLIHKKWF 5do7.2    ----------PNIVNSVVALLSIAGVLVG--------------------------------------------- ``` | | | | | | | | | | | | | | | | | | | | | | | | | | | | | | | | | | | | | | | | | | | | | | | | | |
|  | 5j1i.1.A | Plectin  *Structure of the spectrin repeats 7, 8, and 9 of the plakin domain of plectin* | 0.00 |  | 23.53 | 0.05 | 143-159 | X-ray | 2.80 | monomer |  | HHblits | 0.32 |
| ``` target    MKQVFLSTTTEFKEIDTLEPGTWINLVNPTQNESLEIANTFDIDIADLRAPLDAEEMSRITIEDEYTLIIVDVPVTEERN 5j1i.1    --------------------------------------------------------------------------------  target    NRTYYVTIPLGIIITEETIITTCLEPLPVLDVFINRRLRNFYTFMRSRFIFQILYRNAELYLTALRSIDRKSEQIESQLH 5j1i.1    --------------------------------------------------------------SELKDIRLQLEACETRT-  target    QSTRNEELIELMELEKTIVYFKASLKTNERVIKKLTSSTSNIKKYLEDEDLLEDTLIETQQAIEMADIYGNVLHSMTETF 5j1i.1    --------------------------------------------------------------------------------  target    ASIISNNQNNIMKTLALVTIVMSIPTMVFSAYGMNFKDNEIPLNGEPNAFWLIVFIAFAMSVSLTLYLIHKKWF 5j1i.1    -------------------------------------------------------------------------- ``` | | | | | | | | | | | | | | | | | | | | | | | | | | | | | | | | | | | | | | | | | | | | | | | | | |
|  | 6c5l.1.Y | Peptide chain release factor 2  *Conformation of methylated GGQ in the Peptidyl Transferase Center during translation termination (T. thermophilus)* | 0.00 |  | 11.76 | 0.05 | 145-161 | X-ray | 3.20 | hetero-1-1-1-1-1-1-… | 26 x MG, 3 x ZN | HHblits | 0.31 |
| ``` target    MKQVFLSTTTEFKEIDTLEPGTWINLVNPTQNESLEIANTFDIDIADLRAPLDAEEMSRITIEDEYTLIIVDVPVTEERN 6c5l.1    --------------------------------------------------------------------------------  target    NRTYYVTIPLGIIITEETIITTCLEPLPVLDVFINRRLRNFYTFMRSRFIFQILYRNAELYLTALRSIDRKSEQIESQLH 6c5l.1    ----------------------------------------------------------------IPQKETRLKELERRLE  target    QSTRNEELIELMELEKTIVYFKASLKTNERVIKKLTSSTSNIKKYLEDEDLLEDTLIETQQAIEMADIYGNVLHSMTETF 6c5l.1    D-------------------------------------------------------------------------------  target    ASIISNNQNNIMKTLALVTIVMSIPTMVFSAYGMNFKDNEIPLNGEPNAFWLIVFIAFAMSVSLTLYLIHKKWF 6c5l.1    -------------------------------------------------------------------------- ``` | | | | | | | | | | | | | | | | | | | | | | | | | | | | | | | | | | | | | | | | | | | | | | | | | |
|  | 6c5l.2.Y | Peptide chain release factor 2  *Conformation of methylated GGQ in the Peptidyl Transferase Center during translation termination (T. thermophilus)* | 0.00 |  | 11.76 | 0.05 | 145-161 | X-ray | 3.20 | hetero-1-1-1-1-1-1-… | 27 x MG, 3 x ZN | HHblits | 0.31 |
| ``` target    MKQVFLSTTTEFKEIDTLEPGTWINLVNPTQNESLEIANTFDIDIADLRAPLDAEEMSRITIEDEYTLIIVDVPVTEERN 6c5l.2    --------------------------------------------------------------------------------  target    NRTYYVTIPLGIIITEETIITTCLEPLPVLDVFINRRLRNFYTFMRSRFIFQILYRNAELYLTALRSIDRKSEQIESQLH 6c5l.2    ----------------------------------------------------------------IPQKETRLKELERRLE  target    QSTRNEELIELMELEKTIVYFKASLKTNERVIKKLTSSTSNIKKYLEDEDLLEDTLIETQQAIEMADIYGNVLHSMTETF 6c5l.2    D-------------------------------------------------------------------------------  target    ASIISNNQNNIMKTLALVTIVMSIPTMVFSAYGMNFKDNEIPLNGEPNAFWLIVFIAFAMSVSLTLYLIHKKWF 6c5l.2    -------------------------------------------------------------------------- ``` | | | | | | | | | | | | | | | | | | | | | | | | | | | | | | | | | | | | | | | | | | | | | | | | | |
|  | 4v67.1.Y | Bacterial peptide chain release factor 2 (RF-2)  *Crystal structure of a translation termination complex formed with release factor RF2.* | 0.00 |  | 11.76 | 0.05 | 145-161 | X-ray | 3.00 | hetero-oligomer | 115 x MG, 2 x ZN | HHblits | 0.31 |
| ``` target    MKQVFLSTTTEFKEIDTLEPGTWINLVNPTQNESLEIANTFDIDIADLRAPLDAEEMSRITIEDEYTLIIVDVPVTEERN 4v67.1    --------------------------------------------------------------------------------  target    NRTYYVTIPLGIIITEETIITTCLEPLPVLDVFINRRLRNFYTFMRSRFIFQILYRNAELYLTALRSIDRKSEQIESQLH 4v67.1    ----------------------------------------------------------------IPQKETRLKELERRLE  target    QSTRNEELIELMELEKTIVYFKASLKTNERVIKKLTSSTSNIKKYLEDEDLLEDTLIETQQAIEMADIYGNVLHSMTETF 4v67.1    D-------------------------------------------------------------------------------  target    ASIISNNQNNIMKTLALVTIVMSIPTMVFSAYGMNFKDNEIPLNGEPNAFWLIVFIAFAMSVSLTLYLIHKKWF 4v67.1    -------------------------------------------------------------------------- ``` | | | | | | | | | | | | | | | | | | | | | | | | | | | | | | | | | | | | | | | | | | | | | | | | | |
|  | 5mdy.1.G | Peptide chain release factor 2  *Structure of ArfA and TtRF2 bound to the 70S ribosome (pre-accommodated state)* | 0.00 |  | 11.76 | 0.05 | 145-161 | EM | 3.35 | hetero-1-1-1-1-1-1-… | 19 x MG, 2 x ZN | HHblits | 0.31 |
| ``` target    MKQVFLSTTTEFKEIDTLEPGTWINLVNPTQNESLEIANTFDIDIADLRAPLDAEEMSRITIEDEYTLIIVDVPVTEERN 5mdy.1    --------------------------------------------------------------------------------  target    NRTYYVTIPLGIIITEETIITTCLEPLPVLDVFINRRLRNFYTFMRSRFIFQILYRNAELYLTALRSIDRKSEQIESQLH 5mdy.1    ----------------------------------------------------------------IPQKETRLKELERRLE  target    QSTRNEELIELMELEKTIVYFKASLKTNERVIKKLTSSTSNIKKYLEDEDLLEDTLIETQQAIEMADIYGNVLHSMTETF 5mdy.1    D-------------------------------------------------------------------------------  target    ASIISNNQNNIMKTLALVTIVMSIPTMVFSAYGMNFKDNEIPLNGEPNAFWLIVFIAFAMSVSLTLYLIHKKWF 5mdy.1    -------------------------------------------------------------------------- ``` | | | | | | | | | | | | | | | | | | | | | | | | | | | | | | | | | | | | | | | | | | | | | | | | | |
|  | 5ijn.1.G | NUCLEAR PORE COMPLEX PROTEIN NUP58  *Composite structure of the inner ring of the human nuclear pore complex (32 copies of Nup205)* | 0.00 |  | 11.76 | 0.05 | 143-159 | EM | 0.00 | hetero-6-4-4-4-4-4-… |  | HHblits | 0.30 |
| ``` target    MKQVFLSTTTEFKEIDTLEPGTWINLVNPTQNESLEIANTFDIDIADLRAPLDAEEMSRITIEDEYTLIIVDVPVTEERN 5ijn.1    --------------------------------------------------------------------------------  target    NRTYYVTIPLGIIITEETIITTCLEPLPVLDVFINRRLRNFYTFMRSRFIFQILYRNAELYLTALRSIDRKSEQIESQLH 5ijn.1    --------------------------------------------------------------KAMLKVQEDIKALKQLL-  target    QSTRNEELIELMELEKTIVYFKASLKTNERVIKKLTSSTSNIKKYLEDEDLLEDTLIETQQAIEMADIYGNVLHSMTETF 5ijn.1    --------------------------------------------------------------------------------  target    ASIISNNQNNIMKTLALVTIVMSIPTMVFSAYGMNFKDNEIPLNGEPNAFWLIVFIAFAMSVSLTLYLIHKKWF 5ijn.1    -------------------------------------------------------------------------- ``` | | | | | | | | | | | | | | | | | | | | | | | | | | | | | | | | | | | | | | | | | | | | | | | | | |
|  | 6nyi.1.A | Design construct XXA  *Crystal structure of computationally designed protein XXA* | 0.00 |  | 18.75 | 0.05 | 183-198 | X-ray | 2.30 | homo-trimer |  | HHblits | 0.33 |
| ``` target    MKQVFLSTTTEFKEIDTLEPGTWINLVNPTQNESLEIANTFDIDIADLRAPLDAEEMSRITIEDEYTLIIVDVPVTEERN 6nyi.1    --------------------------------------------------------------------------------  target    NRTYYVTIPLGIIITEETIITTCLEPLPVLDVFINRRLRNFYTFMRSRFIFQILYRNAELYLTALRSIDRKSEQIESQLH 6nyi.1    --------------------------------------------------------------------------------  target    QSTRNEELIELMELEKTIVYFKASLKTNERVIKKLTSSTSNIKKYLEDEDLLEDTLIETQQAIEMADIYGNVLHSMTETF 6nyi.1    ----------------------YSLERLREILERLEEN------------------------------------------  target    ASIISNNQNNIMKTLALVTIVMSIPTMVFSAYGMNFKDNEIPLNGEPNAFWLIVFIAFAMSVSLTLYLIHKKWF 6nyi.1    -------------------------------------------------------------------------- ``` | | | | | | | | | | | | | | | | | | | | | | | | | | | | | | | | | | | | | | | | | | | | | | | | | |
|  | 6nyi.1.B | Design construct XXA  *Crystal structure of computationally designed protein XXA* | 0.00 |  | 18.75 | 0.05 | 183-198 | X-ray | 2.30 | homo-trimer |  | HHblits | 0.33 |
| ``` target    MKQVFLSTTTEFKEIDTLEPGTWINLVNPTQNESLEIANTFDIDIADLRAPLDAEEMSRITIEDEYTLIIVDVPVTEERN 6nyi.1    --------------------------------------------------------------------------------  target    NRTYYVTIPLGIIITEETIITTCLEPLPVLDVFINRRLRNFYTFMRSRFIFQILYRNAELYLTALRSIDRKSEQIESQLH 6nyi.1    --------------------------------------------------------------------------------  target    QSTRNEELIELMELEKTIVYFKASLKTNERVIKKLTSSTSNIKKYLEDEDLLEDTLIETQQAIEMADIYGNVLHSMTETF 6nyi.1    ----------------------YSLERLREILERLEEN------------------------------------------  target    ASIISNNQNNIMKTLALVTIVMSIPTMVFSAYGMNFKDNEIPLNGEPNAFWLIVFIAFAMSVSLTLYLIHKKWF 6nyi.1    -------------------------------------------------------------------------- ``` | | | | | | | | | | | | | | | | | | | | | | | | | | | | | | | | | | | | | | | | | | | | | | | | | |
|  | 6nyi.1.C | Design construct XXA  *Crystal structure of computationally designed protein XXA* | 0.00 |  | 18.75 | 0.05 | 183-198 | X-ray | 2.30 | homo-trimer |  | HHblits | 0.33 |
| ``` target    MKQVFLSTTTEFKEIDTLEPGTWINLVNPTQNESLEIANTFDIDIADLRAPLDAEEMSRITIEDEYTLIIVDVPVTEERN 6nyi.1    --------------------------------------------------------------------------------  target    NRTYYVTIPLGIIITEETIITTCLEPLPVLDVFINRRLRNFYTFMRSRFIFQILYRNAELYLTALRSIDRKSEQIESQLH 6nyi.1    --------------------------------------------------------------------------------  target    QSTRNEELIELMELEKTIVYFKASLKTNERVIKKLTSSTSNIKKYLEDEDLLEDTLIETQQAIEMADIYGNVLHSMTETF 6nyi.1    ----------------------YSLERLREILERLEEN------------------------------------------  target    ASIISNNQNNIMKTLALVTIVMSIPTMVFSAYGMNFKDNEIPLNGEPNAFWLIVFIAFAMSVSLTLYLIHKKWF 6nyi.1    -------------------------------------------------------------------------- ``` | | | | | | | | | | | | | | | | | | | | | | | | | | | | | | | | | | | | | | | | | | | | | | | | | |
|  | 6o0i.1.A | Design construct XAA  *NMR ensemble of computationally designed protein XAA* | 0.00 |  | 18.75 | 0.05 | 183-198 | NMR | 0.00 | homo-trimer |  | HHblits | 0.33 |
| ``` target    MKQVFLSTTTEFKEIDTLEPGTWINLVNPTQNESLEIANTFDIDIADLRAPLDAEEMSRITIEDEYTLIIVDVPVTEERN 6o0i.1    --------------------------------------------------------------------------------  target    NRTYYVTIPLGIIITEETIITTCLEPLPVLDVFINRRLRNFYTFMRSRFIFQILYRNAELYLTALRSIDRKSEQIESQLH 6o0i.1    --------------------------------------------------------------------------------  target    QSTRNEELIELMELEKTIVYFKASLKTNERVIKKLTSSTSNIKKYLEDEDLLEDTLIETQQAIEMADIYGNVLHSMTETF 6o0i.1    ----------------------YSLERLREILERLEEN------------------------------------------  target    ASIISNNQNNIMKTLALVTIVMSIPTMVFSAYGMNFKDNEIPLNGEPNAFWLIVFIAFAMSVSLTLYLIHKKWF 6o0i.1    -------------------------------------------------------------------------- ``` | | | | | | | | | | | | | | | | | | | | | | | | | | | | | | | | | | | | | | | | | | | | | | | | | |
|  | 6nz1.1.A | Design construct XXA\_GVDQ  *Crystal structure of computationally designed protein XXA\_GVDQ* | 0.00 |  | 18.75 | 0.05 | 183-198 | X-ray | 1.90 | homo-trimer |  | HHblits | 0.33 |
| ``` target    MKQVFLSTTTEFKEIDTLEPGTWINLVNPTQNESLEIANTFDIDIADLRAPLDAEEMSRITIEDEYTLIIVDVPVTEERN 6nz1.1    --------------------------------------------------------------------------------  target    NRTYYVTIPLGIIITEETIITTCLEPLPVLDVFINRRLRNFYTFMRSRFIFQILYRNAELYLTALRSIDRKSEQIESQLH 6nz1.1    --------------------------------------------------------------------------------  target    QSTRNEELIELMELEKTIVYFKASLKTNERVIKKLTSSTSNIKKYLEDEDLLEDTLIETQQAIEMADIYGNVLHSMTETF 6nz1.1    ----------------------YSLERLREILERLEEN------------------------------------------  target    ASIISNNQNNIMKTLALVTIVMSIPTMVFSAYGMNFKDNEIPLNGEPNAFWLIVFIAFAMSVSLTLYLIHKKWF 6nz1.1    -------------------------------------------------------------------------- ``` | | | | | | | | | | | | | | | | | | | | | | | | | | | | | | | | | | | | | | | | | | | | | | | | | |
|  | 6nz1.1.B | Design construct XXA\_GVDQ  *Crystal structure of computationally designed protein XXA\_GVDQ* | 0.00 |  | 18.75 | 0.05 | 183-198 | X-ray | 1.90 | homo-trimer |  | HHblits | 0.33 |
| ``` target    MKQVFLSTTTEFKEIDTLEPGTWINLVNPTQNESLEIANTFDIDIADLRAPLDAEEMSRITIEDEYTLIIVDVPVTEERN 6nz1.1    --------------------------------------------------------------------------------  target    NRTYYVTIPLGIIITEETIITTCLEPLPVLDVFINRRLRNFYTFMRSRFIFQILYRNAELYLTALRSIDRKSEQIESQLH 6nz1.1    --------------------------------------------------------------------------------  target    QSTRNEELIELMELEKTIVYFKASLKTNERVIKKLTSSTSNIKKYLEDEDLLEDTLIETQQAIEMADIYGNVLHSMTETF 6nz1.1    ----------------------YSLERLREILERLEEN------------------------------------------  target    ASIISNNQNNIMKTLALVTIVMSIPTMVFSAYGMNFKDNEIPLNGEPNAFWLIVFIAFAMSVSLTLYLIHKKWF 6nz1.1    -------------------------------------------------------------------------- ``` | | | | | | | | | | | | | | | | | | | | | | | | | | | | | | | | | | | | | | | | | | | | | | | | | |
|  | 6nz1.1.C | Design construct XXA\_GVDQ  *Crystal structure of computationally designed protein XXA\_GVDQ* | 0.00 |  | 18.75 | 0.05 | 183-198 | X-ray | 1.90 | homo-trimer |  | HHblits | 0.33 |
| ``` target    MKQVFLSTTTEFKEIDTLEPGTWINLVNPTQNESLEIANTFDIDIADLRAPLDAEEMSRITIEDEYTLIIVDVPVTEERN 6nz1.1    --------------------------------------------------------------------------------  target    NRTYYVTIPLGIIITEETIITTCLEPLPVLDVFINRRLRNFYTFMRSRFIFQILYRNAELYLTALRSIDRKSEQIESQLH 6nz1.1    --------------------------------------------------------------------------------  target    QSTRNEELIELMELEKTIVYFKASLKTNERVIKKLTSSTSNIKKYLEDEDLLEDTLIETQQAIEMADIYGNVLHSMTETF 6nz1.1    ----------------------YSLERLREILERLEEN------------------------------------------  target    ASIISNNQNNIMKTLALVTIVMSIPTMVFSAYGMNFKDNEIPLNGEPNAFWLIVFIAFAMSVSLTLYLIHKKWF 6nz1.1    -------------------------------------------------------------------------- ``` | | | | | | | | | | | | | | | | | | | | | | | | | | | | | | | | | | | | | | | | | | | | | | | | | |
|  | 6dfp.1.A | VCA0883  *Crystal Structure of a Tripartite Toxin Component VCA0883 from Vibrio cholerae* | 0.00 |  | 18.75 | 0.05 | 172-187 | X-ray | 1.50 | monomer |  | HHblits | 0.30 |
| ``` target    MKQVFLSTTTEFKEIDTLEPGTWINLVNPTQNESLEIANTFDIDIADLRAPLDAEEMSRITIEDEYTLIIVDVPVTEERN 6dfp.1    --------------------------------------------------------------------------------  target    NRTYYVTIPLGIIITEETIITTCLEPLPVLDVFINRRLRNFYTFMRSRFIFQILYRNAELYLTALRSIDRKSEQIESQLH 6dfp.1    --------------------------------------------------------------------------------  target    QSTRNEELIELMELEKTIVYFKASLKTNERVIKKLTSSTSNIKKYLEDEDLLEDTLIETQQAIEMADIYGNVLHSMTETF 6dfp.1    -----------TALSSQVSGIEATVKG-----------------------------------------------------  target    ASIISNNQNNIMKTLALVTIVMSIPTMVFSAYGMNFKDNEIPLNGEPNAFWLIVFIAFAMSVSLTLYLIHKKWF 6dfp.1    -------------------------------------------------------------------------- ``` | | | | | | | | | | | | | | | | | | | | | | | | | | | | | | | | | | | | | | | | | | | | | | | | | |
|  | 6m3p.1.B | Spectrin beta chain, non-erythrocytic 1  *Crystal structure of AnkG/beta2-spectrin complex* | 0.00 |  | 5.88 | 0.05 | 143-159 | X-ray | 3.31 | hetero-1-1-mer |  | HHblits | 0.24 |
| ``` target    MKQVFLSTTTEFKEIDTLEPGTWINLVNPTQNESLEIANTFDIDIADLRAPLDAEEMSRITIEDEYTLIIVDVPVTEERN 6m3p.1    --------------------------------------------------------------------------------  target    NRTYYVTIPLGIIITEETIITTCLEPLPVLDVFINRRLRNFYTFMRSRFIFQILYRNAELYLTALRSIDRKSEQIESQLH 6m3p.1    --------------------------------------------------------------FDAAEAEAWMSEQELYM-  target    QSTRNEELIELMELEKTIVYFKASLKTNERVIKKLTSSTSNIKKYLEDEDLLEDTLIETQQAIEMADIYGNVLHSMTETF 6m3p.1    --------------------------------------------------------------------------------  target    ASIISNNQNNIMKTLALVTIVMSIPTMVFSAYGMNFKDNEIPLNGEPNAFWLIVFIAFAMSVSLTLYLIHKKWF 6m3p.1    -------------------------------------------------------------------------- ``` | | | | | | | | | | | | | | | | | | | | | | | | | | | | | | | | | | | | | | | | | | | | | | | | | |
|  | 6z5s.1.A | Light harvesting complex 1 Protein W  *RC-LH1(14)-W complex from Rhodopseudomonas palustris* | 0.00 |  | 0.00 | 0.05 | 297-312 | EM | 0.00 | hetero-1-1-1-1-14-1… | 1 x QAK, 32 x BCL, 2 x BPH, 3 x U10, 3 x 6PL, 6 x CDL, 1 x FE, 29 x LMT, 13 x CRT, 1 x PGT | HHblits | 0.25 |
| ``` target    MKQVFLSTTTEFKEIDTLEPGTWINLVNPTQNESLEIANTFDIDIADLRAPLDAEEMSRITIEDEYTLIIVDVPVTEERN 6z5s.1    --------------------------------------------------------------------------------  target    NRTYYVTIPLGIIITEETIITTCLEPLPVLDVFINRRLRNFYTFMRSRFIFQILYRNAELYLTALRSIDRKSEQIESQLH 6z5s.1    --------------------------------------------------------------------------------  target    QSTRNEELIELMELEKTIVYFKASLKTNERVIKKLTSSTSNIKKYLEDEDLLEDTLIETQQAIEMADIYGNVLHSMTETF 6z5s.1    --------------------------------------------------------------------------------  target    ASIISNNQNNIMKTLALVTIVMSIPTMVFSAYGMNFKDNEIPLNGEPNAFWLIVFIAFAMSVSLTLYLIHKKWF 6z5s.1    --------------------------------------------------------TLFVGPVVFWFFRRQR-- ``` | | | | | | | | | | | | | | | | | | | | | | | | | | | | | | | | | | | | | | | | | | | | | | | | | |
|  | 6m3q.1.B | Spectrin beta chain  *Crystal structure of AnkB/beta4-spectrin complex* | 0.00 |  | 6.67 | 0.05 | 145-159 | X-ray | 3.44 | hetero-1-1-mer |  | HHblits | 0.26 |
| ``` target    MKQVFLSTTTEFKEIDTLEPGTWINLVNPTQNESLEIANTFDIDIADLRAPLDAEEMSRITIEDEYTLIIVDVPVTEERN 6m3q.1    --------------------------------------------------------------------------------  target    NRTYYVTIPLGIIITEETIITTCLEPLPVLDVFINRRLRNFYTFMRSRFIFQILYRNAELYLTALRSIDRKSEQIESQLH 6m3q.1    ----------------------------------------------------------------VAEVEAWLGEQELLM-  target    QSTRNEELIELMELEKTIVYFKASLKTNERVIKKLTSSTSNIKKYLEDEDLLEDTLIETQQAIEMADIYGNVLHSMTETF 6m3q.1    --------------------------------------------------------------------------------  target    ASIISNNQNNIMKTLALVTIVMSIPTMVFSAYGMNFKDNEIPLNGEPNAFWLIVFIAFAMSVSLTLYLIHKKWF 6m3q.1    -------------------------------------------------------------------------- ``` | | | | | | | | | | | | | | | | | | | | | | | | | | | | | | | | | | | | | | | | | | | | | | | | | |
|  | 6m3r.1.B | Spectrin beta chain  *Crystal structure of AnkG/beta4-spectrin complex* | 0.00 |  | 6.67 | 0.05 | 145-159 | X-ray | 4.31 | hetero-1-1-mer |  | HHblits | 0.26 |
| ``` target    MKQVFLSTTTEFKEIDTLEPGTWINLVNPTQNESLEIANTFDIDIADLRAPLDAEEMSRITIEDEYTLIIVDVPVTEERN 6m3r.1    --------------------------------------------------------------------------------  target    NRTYYVTIPLGIIITEETIITTCLEPLPVLDVFINRRLRNFYTFMRSRFIFQILYRNAELYLTALRSIDRKSEQIESQLH 6m3r.1    ----------------------------------------------------------------VAEVEAWLGEQELLM-  target    QSTRNEELIELMELEKTIVYFKASLKTNERVIKKLTSSTSNIKKYLEDEDLLEDTLIETQQAIEMADIYGNVLHSMTETF 6m3r.1    --------------------------------------------------------------------------------  target    ASIISNNQNNIMKTLALVTIVMSIPTMVFSAYGMNFKDNEIPLNGEPNAFWLIVFIAFAMSVSLTLYLIHKKWF 6m3r.1    -------------------------------------------------------------------------- ``` | | | | | | | | | | | | | | | | | | | | | | | | | | | | | | | | | | | | | | | | | | | | | | | | | |
|  | 6gap.1.A | Outer capsid protein sigma-1  *Crystal structure of the T3D reovirus sigma1 coiled coil tail and body* | 0.00 |  | 6.67 | 0.05 | 260-274 | X-ray | 2.15 | homo-trimer |  | HHblits | 0.21 |
| ``` target    MKQVFLSTTTEFKEIDTLEPGTWINLVNPTQNESLEIANTFDIDIADLRAPLDAEEMSRITIEDEYTLIIVDVPVTEERN 6gap.1    --------------------------------------------------------------------------------  target    NRTYYVTIPLGIIITEETIITTCLEPLPVLDVFINRRLRNFYTFMRSRFIFQILYRNAELYLTALRSIDRKSEQIESQLH 6gap.1    --------------------------------------------------------------------------------  target    QSTRNEELIELMELEKTIVYFKASLKTNERVIKKLTSSTSNIKKYLEDEDLLEDTLIETQQAIEMADIYGNVLHSMTETF 6gap.1    --------------------------------------------------------------------------------  target    ASIISNNQNNIMKTLALVTIVMSIPTMVFSAYGMNFKDNEIPLNGEPNAFWLIVFIAFAMSVSLTLYLIHKKWF 6gap.1    -------------------SAGAPLSIRNNRMTM---------------------------------------- ``` | | | | | | | | | | | | | | | | | | | | | | | | | | | | | | | | | | | | | | | | | | | | | | | | | |
|  | 6gap.1.B | Outer capsid protein sigma-1  *Crystal structure of the T3D reovirus sigma1 coiled coil tail and body* | 0.00 |  | 6.67 | 0.05 | 260-274 | X-ray | 2.15 | homo-trimer |  | HHblits | 0.21 |
| ``` target    MKQVFLSTTTEFKEIDTLEPGTWINLVNPTQNESLEIANTFDIDIADLRAPLDAEEMSRITIEDEYTLIIVDVPVTEERN 6gap.1    --------------------------------------------------------------------------------  target    NRTYYVTIPLGIIITEETIITTCLEPLPVLDVFINRRLRNFYTFMRSRFIFQILYRNAELYLTALRSIDRKSEQIESQLH 6gap.1    --------------------------------------------------------------------------------  target    QSTRNEELIELMELEKTIVYFKASLKTNERVIKKLTSSTSNIKKYLEDEDLLEDTLIETQQAIEMADIYGNVLHSMTETF 6gap.1    --------------------------------------------------------------------------------  target    ASIISNNQNNIMKTLALVTIVMSIPTMVFSAYGMNFKDNEIPLNGEPNAFWLIVFIAFAMSVSLTLYLIHKKWF 6gap.1    -------------------SAGAPLSIRNNRMTM---------------------------------------- ``` | | | | | | | | | | | | | | | | | | | | | | | | | | | | | | | | | | | | | | | | | | | | | | | | | |
|  | 6gap.1.C | Outer capsid protein sigma-1  *Crystal structure of the T3D reovirus sigma1 coiled coil tail and body* | 0.00 |  | 6.67 | 0.05 | 260-274 | X-ray | 2.15 | homo-trimer |  | HHblits | 0.21 |
| ``` target    MKQVFLSTTTEFKEIDTLEPGTWINLVNPTQNESLEIANTFDIDIADLRAPLDAEEMSRITIEDEYTLIIVDVPVTEERN 6gap.1    --------------------------------------------------------------------------------  target    NRTYYVTIPLGIIITEETIITTCLEPLPVLDVFINRRLRNFYTFMRSRFIFQILYRNAELYLTALRSIDRKSEQIESQLH 6gap.1    --------------------------------------------------------------------------------  target    QSTRNEELIELMELEKTIVYFKASLKTNERVIKKLTSSTSNIKKYLEDEDLLEDTLIETQQAIEMADIYGNVLHSMTETF 6gap.1    --------------------------------------------------------------------------------  target    ASIISNNQNNIMKTLALVTIVMSIPTMVFSAYGMNFKDNEIPLNGEPNAFWLIVFIAFAMSVSLTLYLIHKKWF 6gap.1    -------------------SAGAPLSIRNNRMTM---------------------------------------- ``` | | | | | | | | | | | | | | | | | | | | | | | | | | | | | | | | | | | | | | | | | | | | | | | | | |

**Export Alignment**
  
FASTA format
Clustal Format
PNG Image

**Secondary Structure**
  
None
DSSP
PSIPRED
SSpro

**Colour Scheme** 


Fade Mismatches
Enhance Mismatches

Clustal
Hydrophobic
Size
Charged
Polar
Proline
Ser/Thr
Cysteine
Aliphatic
Aromatic
QMEAN
Indels
Chain
Unique Chain
Rainbow
Structure
No Colour

 Use QMEANBrane values

|  |  |  |  |
| --- | --- | --- | --- |
| Background |  |  |  |

**3D Viewer**  
NGL
PV

FASTA
Multi FASTA
ClustalW
PNG
