# Supplementary material for: A bacterial pan-genome makes gene essentiality strain-dependent and evolvable
Source: Nat Microbiol. 2022 Sep 12;7(10):1580–92. doi: 10.1038/s41564-022-01208-7 (PMC9519441; doi:10.1038/s41564-022-01208-7)
Supplement: Source Data Fig. 4 — Swiss models data, genetic interactions (a subset of Supplementary Data 11), growth curves data and RNA-seq data for the two transporters (taken from Supplementary Data 6). [file 41564_2022_1208_MOESM8_ESM.zip › Fig_4a_models/SP_0185_swissmodel/SP_0185/report.html]

SP\_0185 | Report


|  |  |  |
| --- | --- | --- |
|  |  | SWISS-MODEL Homology Modelling Report |

## Model Building Report

This document lists the results for the homology modelling project "SP\_0185" submitted to SWISS-MODEL workspace
on May 24, 2021, 4:25 a.m..The submitted primary amino acid sequence is given in Table T1.

If you use any results in your research, please cite the relevant publications:

- Waterhouse, A., Bertoni, M., Bienert, S., Studer, G., Tauriello, G., Gumienny, R.,
  Heer, F.T., de Beer, T.A.P., Rempfer, C., Bordoli, L., Lepore, R., Schwede, T.
  SWISS-MODEL: homology modelling of protein structures and complexes.
  Nucleic Acids Res. 46(W1), W296-W303 (2018).
- Bienert, S., Waterhouse, A., de Beer, T.A.P., Tauriello, G., Studer,
  G., Bordoli, L., Schwede, T. The SWISS-MODEL Repository - new features and
  functionality. Nucleic Acids Res. 45, D313-D319 (2017).
- Studer, G., Tauriello, G., Bienert, S.,
  Biasini, M., Johner, N., Schwede, T. ProMod3 - A versatile homology
  modelling toolbox. PLOS Comp. Biol. 17(1), e1008667 (2021).
- Studer, G., Rempfer, C., Waterhouse, A.M.,
  Gumienny, G., Haas, J., Schwede, T. QMEANDisCo - distance constraints
  applied on model quality estimation. Bioinformatics 36, 1765-1771 (2020).
- Bertoni, M., Kiefer, F., Biasini, M., Bordoli, L.,
  Schwede, T. Modeling protein quaternary structure of homo- and
  hetero-oligomers beyond binary interactions by homology. Scientific
  Reports 7 (2017).

## Results

The SWISS-MODEL template library (SMTL version 2021-05-19, PDB release 2021-05-14) was searched with
BLAST (Camacho et al.) and HHblits (Steinegger et al.)
for evolutionary related structures matching the target sequence in Table T1. For details on the template search, see Materials and Methods. Overall 296 templates were found (Table T2).

## Models

The following models were built (see Materials and Methods "Model Building"):

| Model #01 | File | Built with | Oligo-State | Ligands | GMQE | QMEAN |
| --- | --- | --- | --- | --- | --- | --- |
|  | PDB | ProMod3 3.2.0 | homo-pentamer (matching prediction) | 1 x MG: MAGNESIUM ION; | 0.65 | -2.27 |

|  |  |  |
| --- | --- | --- |
|  |  |  |

| Template | Seq Identity | Oligo-state | QSQE | Found by | Method | Resolution | Seq Similarity | Range | Coverage | Description |
| --- | --- | --- | --- | --- | --- | --- | --- | --- | --- | --- |
| 4ev6.1.A | 26.55 | homo-pentamer | 0.54 | HHblits | X-ray | 3.20Å | 0.34 | 17 - 314 | 0.92 | Magnesium transport protein CorA |

  

### Included Ligands

| Ligand | Description |
| --- | --- |
| 1 x MG | MAGNESIUM ION |

  

### Excluded ligands

| Ligand Name.Number | Reason for Exclusion | Description |
| --- | --- | --- |
| MG.3 | Not in contact with model. | MAGNESIUM ION |
| MG.4 | Not in contact with model. | MAGNESIUM ION |
| MG.5 | Binding site not conserved. | MAGNESIUM ION |
| MG.6 | Not in contact with model. | MAGNESIUM ION |
| MG.7 | Not in contact with model. | MAGNESIUM ION |
| MG.8 | Not in contact with model. | MAGNESIUM ION |
| MG.9 | Binding site not conserved. | MAGNESIUM ION |
| MG.12 | Not in contact with model. | MAGNESIUM ION |
| MG.13 | Not in contact with model. | MAGNESIUM ION |
| MG.14 | Not in contact with model. | MAGNESIUM ION |
| MG.16 | Not in contact with model. | MAGNESIUM ION |
| MG.17 | Binding site not conserved. | MAGNESIUM ION |
| MG.18 | Not in contact with model. | MAGNESIUM ION |
| MG.20 | Binding site not conserved. | MAGNESIUM ION |
| MG.21 | Binding site not conserved. | MAGNESIUM ION |
| MG.22 | Not in contact with model. | MAGNESIUM ION |
| MG.23 | Binding site not conserved. | MAGNESIUM ION |
| MG.24 | Not in contact with model. | MAGNESIUM ION |
| MG.25 | Binding site not conserved. | MAGNESIUM ION |
| MG.26 | Not in contact with model. | MAGNESIUM ION |
| MG.27 | Binding site not conserved. | MAGNESIUM ION |
| MG.28 | Binding site not conserved. | MAGNESIUM ION |
| MG.29 | Binding site not conserved. | MAGNESIUM ION |
| MG.32 | Not in contact with model. | MAGNESIUM ION |
| MG.33 | Binding site not conserved. | MAGNESIUM ION |
| MG.34 | Binding site not conserved. | MAGNESIUM ION |
| MG.35 | Binding site not conserved. | MAGNESIUM ION |
| MG.36 | Binding site not conserved. | MAGNESIUM ION |
| MG.37 | Not in contact with model. | MAGNESIUM ION |
| MG.39 | Binding site not conserved. | MAGNESIUM ION |
| MG.40 | Binding site not conserved. | MAGNESIUM ION |
| UMQ.1 | Binding site not conserved. | UNDECYL-MALTOSIDE |
| UMQ.2 | Binding site not conserved. | UNDECYL-MALTOSIDE |
| UMQ.10 | Binding site not conserved. | UNDECYL-MALTOSIDE |
| UMQ.11 | Binding site not conserved. | UNDECYL-MALTOSIDE |
| UMQ.15 | Binding site not conserved. | UNDECYL-MALTOSIDE |
| UMQ.19 | Binding site not conserved. | UNDECYL-MALTOSIDE |
| UMQ.30 | Binding site not conserved. | UNDECYL-MALTOSIDE |
| UMQ.31 | Binding site not conserved. | UNDECYL-MALTOSIDE |

  

```
Target    MKQVFLSTTTEFKEIDTLEPGTWINLVNPTQNESLEIANTFDIDIADLRAPLDAEEMSRITIEDEYTLIIVDVPVTEERN  
4ev6.1.A  ----------------EDYRLIWIDCYDPKDEELYKLSKKIGISVSDLQIGLDEQEIPRVEEDEDFYLIIYKAPLFEE--  
  
Target    NRTYYVTIPLGIIITEETIITTCLEPLPVLDVFINRRLRNFYT----FMRSRFIFQILYRNAELYLTALRSIDRKSEQIE  
4ev6.1.A  ---DITTTSLGIYIKNNLLLTIHSDKIKAIGRLHKLISTKKPRIVFERGIGFLLYHILNEITRSYSRILMNLEDELEELE  
  
Target    SQLHQSTRNEELIELMELEKTIVYFKASLKTNERVIKKLTSSTSNIKKYLEDEDLLEDTLIETQQAIEMADIYGNVLHSM  
4ev6.1.A  DKLLAGYDREVMEKILGLRKTLVYFHKSLIANRDVLVLLKRKYLPI-TTKEDRENFEDLYYDTLQLIDMSATYREVLTSM  
  
Target    TETFASIISNNQNNIMKTLALVTIVMSIPTMVFSAYGMNFKDNEIPLNGEPNAFWLIVFIAFAMSVSLTLYLIHKKWF  
4ev6.1.A  MDITLSLENIKMNQIMKILTMVTTIFAVPMWITGIYGMNFSY--LPLANNPQGFWLVMALMVVIIMIFVYIFRRSGWI  
  
  
Target    MKQVFLSTTTEFKEIDTLEPGTWINLVNPTQNESLEIANTFDIDIADLRAPLDAEEMSRITIEDEYTLIIVDVPVTEERN  
4ev6.1.B  ----------------EDYRLIWIDCYDPKDEELYKLSKKIGISVSDLQIGLDEQEIPRVEEDEDFYLIIYKAPLFEE--  
  
Target    NRTYYVTIPLGIIITEETIITTCLEPLPVLDVFINRRLRNFYT----FMRSRFIFQILYRNAELYLTALRSIDRKSEQIE  
4ev6.1.B  ---DITTTSLGIYIKNNLLLTIHSDKIKAIGRLHKLISTKKPRIVFERGIGFLLYHILNEITRSYSRILMNLEDELEELE  
  
Target    SQLHQSTRNEELIELMELEKTIVYFKASLKTNERVIKKLTSSTSNIKKYLEDEDLLEDTLIETQQAIEMADIYGNVLHSM  
4ev6.1.B  DKLLAGYDREVMEKILGLRKTLVYFHKSLIANRDVLVLLKRKYLPI-TTKEDRENFEDLYYDTLQLIDMSATYREVLTSM  
  
Target    TETFASIISNNQNNIMKTLALVTIVMSIPTMVFSAYGMNFKDNEIPLNGEPNAFWLIVFIAFAMSVSLTLYLIHKKWF  
4ev6.1.B  MDITLSLENIKMNQIMKILTMVTTIFAVPMWITGIYGMNFSY--LPLANNPQGFWLVMALMVVIIMIFVYIFRRSGWI  
  
  
Target    MKQVFLSTTTEFKEIDTLEPGTWINLVNPTQNESLEIANTFDIDIADLRAPLDAEEMSRITIEDEYTLIIVDVPVTEERN  
4ev6.1.C  ----------------EDYRLIWIDCYDPKDEELYKLSKKIGISVSDLQIGLDEQEIPRVEEDEDFYLIIYKAPLFEE--  
  
Target    NRTYYVTIPLGIIITEETIITTCLEPLPVLDVFINRRLRNFYT----FMRSRFIFQILYRNAELYLTALRSIDRKSEQIE  
4ev6.1.C  ---DITTTSLGIYIKNNLLLTIHSDKIKAIGRLHKLISTKKPRIVFERGIGFLLYHILNEITRSYSRILMNLEDELEELE  
  
Target    SQLHQSTRNEELIELMELEKTIVYFKASLKTNERVIKKLTSSTSNIKKYLEDEDLLEDTLIETQQAIEMADIYGNVLHSM  
4ev6.1.C  DKLLAGYDREVMEKILGLRKTLVYFHKSLIANRDVLVLLKRKYLPI-TTKEDRENFEDLYYDTLQLIDMSATYREVLTSM  
  
Target    TETFASIISNNQNNIMKTLALVTIVMSIPTMVFSAYGMNFKDNEIPLNGEPNAFWLIVFIAFAMSVSLTLYLIHKKWF  
4ev6.1.C  MDITLSLENIKMNQIMKILTMVTTIFAVPMWITGIYGMNFSY--LPLANNPQGFWLVMALMVVIIMIFVYIFRRSGWI  
  
  
Target    MKQVFLSTTTEFKEIDTLEPGTWINLVNPTQNESLEIANTFDIDIADLRAPLDAEEMSRITIEDEYTLIIVDVPVTEERN  
4ev6.1.D  ----------------EDYRLIWIDCYDPKDEELYKLSKKIGISVSDLQIGLDEQEIPRVEEDEDFYLIIYKAPLFEE--  
  
Target    NRTYYVTIPLGIIITEETIITTCLEPLPVLDVFINRRLRNFYT----FMRSRFIFQILYRNAELYLTALRSIDRKSEQIE  
4ev6.1.D  ---DITTTSLGIYIKNNLLLTIHSDKIKAIGRLHKLISTKKPRIVFERGIGFLLYHILNEITRSYSRILMNLEDELEELE  
  
Target    SQLHQSTRNEELIELMELEKTIVYFKASLKTNERVIKKLTSSTSNIKKYLEDEDLLEDTLIETQQAIEMADIYGNVLHSM  
4ev6.1.D  DKLLAGYDREVMEKILGLRKTLVYFHKSLIANRDVLVLLKRKYLPI-TTKEDRENFEDLYYDTLQLIDMSATYREVLTSM  
  
Target    TETFASIISNNQNNIMKTLALVTIVMSIPTMVFSAYGMNFKDNEIPLNGEPNAFWLIVFIAFAMSVSLTLYLIHKKWF  
4ev6.1.D  MDITLSLENIKMNQIMKILTMVTTIFAVPMWITGIYGMNFSY--LPLANNPQGFWLVMALMVVIIMIFVYIFRRSGWI  
  
  
Target    MKQVFLSTTTEFKEIDTLEPGTWINLVNPTQNESLEIANTFDIDIADLRAPLDAEEMSRITIEDEYTLIIVDVPVTEERN  
4ev6.1.E  ----------------EDYRLIWIDCYDPKDEELYKLSKKIGISVSDLQIGLDEQEIPRVEEDEDFYLIIYKAPLFEE--  
  
Target    NRTYYVTIPLGIIITEETIITTCLEPLPVLDVFINRRLRNFYT----FMRSRFIFQILYRNAELYLTALRSIDRKSEQIE  
4ev6.1.E  ---DITTTSLGIYIKNNLLLTIHSDKIKAIGRLHKLISTKKPRIVFERGIGFLLYHILNEITRSYSRILMNLEDELEELE  
  
Target    SQLHQSTRNEELIELMELEKTIVYFKASLKTNERVIKKLTSSTSNIKKYLEDEDLLEDTLIETQQAIEMADIYGNVLHSM  
4ev6.1.E  DKLLAGYDREVMEKILGLRKTLVYFHKSLIANRDVLVLLKRKYLPI-TTKEDRENFEDLYYDTLQLIDMSATYREVLTSM  
  
Target    TETFASIISNNQNNIMKTLALVTIVMSIPTMVFSAYGMNFKDNEIPLNGEPNAFWLIVFIAFAMSVSLTLYLIHKKWF  
4ev6.1.E  MDITLSLENIKMNQIMKILTMVTTIFAVPMWITGIYGMNFSY--LPLANNPQGFWLVMALMVVIIMIFVYIFRRSGWI
```

  


---

  

| Model #02 | File | Built with | Oligo-State | Ligands | GMQE | QMEAN |
| --- | --- | --- | --- | --- | --- | --- |
|  | PDB | ProMod3 3.2.0 | homo-trimer (matching prediction) | None | 0.07 | -3.73 |

|  |  |  |
| --- | --- | --- |
|  |  |  |

| Template | Seq Identity | Oligo-state | QSQE | Found by | Method | Resolution | Seq Similarity | Range | Coverage | Description |
| --- | --- | --- | --- | --- | --- | --- | --- | --- | --- | --- |
| 6b7n.1.A | 14.29 | homo-trimer | 0.00 | HHblits | EM | - | 0.28 | 137 - 227 | 0.27 | Spike protein |

  

### Excluded ligands

| Ligand Name.Number | Reason for Exclusion | Description |
| --- | --- | --- |
| NAG.16 | Binding site not conserved. | 2-acetamido-2-deoxy-beta-D-glucopyranose |
| NAG.17 | Binding site not conserved. | 2-acetamido-2-deoxy-beta-D-glucopyranose |
| NAG.18 | Binding site not conserved. | 2-acetamido-2-deoxy-beta-D-glucopyranose |
| NAG.19 | Binding site not conserved. | 2-acetamido-2-deoxy-beta-D-glucopyranose |
| NAG.20 | Binding site not conserved. | 2-acetamido-2-deoxy-beta-D-glucopyranose |
| NAG.21 | Not in contact with model. | 2-acetamido-2-deoxy-beta-D-glucopyranose |
| NAG.22 | Binding site not conserved. | 2-acetamido-2-deoxy-beta-D-glucopyranose |
| NAG.23 | Binding site not conserved. | 2-acetamido-2-deoxy-beta-D-glucopyranose |
| NAG.24 | Binding site not conserved. | 2-acetamido-2-deoxy-beta-D-glucopyranose |
| NAG.25 | Binding site not conserved. | 2-acetamido-2-deoxy-beta-D-glucopyranose |
| NAG.26 | Not in contact with model. | 2-acetamido-2-deoxy-beta-D-glucopyranose |
| NAG.27 | Binding site not conserved. | 2-acetamido-2-deoxy-beta-D-glucopyranose |
| NAG.28 | Binding site not conserved. | 2-acetamido-2-deoxy-beta-D-glucopyranose |
| NAG.29 | Not in contact with model. | 2-acetamido-2-deoxy-beta-D-glucopyranose |
| NAG.30 | Binding site not conserved. | 2-acetamido-2-deoxy-beta-D-glucopyranose |
| NAG.31 | Binding site not conserved. | 2-acetamido-2-deoxy-beta-D-glucopyranose |
| NAG.32 | Binding site not conserved. | 2-acetamido-2-deoxy-beta-D-glucopyranose |
| NAG.33 | Binding site not conserved. | 2-acetamido-2-deoxy-beta-D-glucopyranose |
| NAG.34 | Binding site not conserved. | 2-acetamido-2-deoxy-beta-D-glucopyranose |
| NAG.35 | Binding site not conserved. | 2-acetamido-2-deoxy-beta-D-glucopyranose |
| NAG.36 | Binding site not conserved. | 2-acetamido-2-deoxy-beta-D-glucopyranose |
| NAG.37 | Not in contact with model. | 2-acetamido-2-deoxy-beta-D-glucopyranose |
| NAG.38 | Binding site not conserved. | 2-acetamido-2-deoxy-beta-D-glucopyranose |
| NAG.39 | Binding site not conserved. | 2-acetamido-2-deoxy-beta-D-glucopyranose |
| NAG-NAG.1 | Binding site not conserved. | 2-acetamido-2-deoxy-beta-D-glucopyranose-(1-4)-2-acetamido-2-deoxy-beta-D-glucopyranose |
| NAG-NAG.2 | Binding site not conserved. | 2-acetamido-2-deoxy-beta-D-glucopyranose-(1-4)-2-acetamido-2-deoxy-beta-D-glucopyranose |
| NAG-NAG.3 | Binding site not conserved. | 2-acetamido-2-deoxy-beta-D-glucopyranose-(1-4)-2-acetamido-2-deoxy-beta-D-glucopyranose |
| NAG-NAG.5 | Binding site not conserved. | 2-acetamido-2-deoxy-beta-D-glucopyranose-(1-4)-2-acetamido-2-deoxy-beta-D-glucopyranose |
| NAG-NAG.6 | Binding site not conserved. | 2-acetamido-2-deoxy-beta-D-glucopyranose-(1-4)-2-acetamido-2-deoxy-beta-D-glucopyranose |
| NAG-NAG.7 | Binding site not conserved. | 2-acetamido-2-deoxy-beta-D-glucopyranose-(1-4)-2-acetamido-2-deoxy-beta-D-glucopyranose |
| NAG-NAG.8 | Binding site not conserved. | 2-acetamido-2-deoxy-beta-D-glucopyranose-(1-4)-2-acetamido-2-deoxy-beta-D-glucopyranose |
| NAG-NAG.10 | Binding site not conserved. | 2-acetamido-2-deoxy-beta-D-glucopyranose-(1-4)-2-acetamido-2-deoxy-beta-D-glucopyranose |
| NAG-NAG.11 | Binding site not conserved. | 2-acetamido-2-deoxy-beta-D-glucopyranose-(1-4)-2-acetamido-2-deoxy-beta-D-glucopyranose |
| NAG-NAG.12 | Binding site not conserved. | 2-acetamido-2-deoxy-beta-D-glucopyranose-(1-4)-2-acetamido-2-deoxy-beta-D-glucopyranose |
| NAG-NAG.13 | Binding site not conserved. | 2-acetamido-2-deoxy-beta-D-glucopyranose-(1-4)-2-acetamido-2-deoxy-beta-D-glucopyranose |
| NAG-NAG.15 | Binding site not conserved. | 2-acetamido-2-deoxy-beta-D-glucopyranose-(1-4)-2-acetamido-2-deoxy-beta-D-glucopyranose |
| NAG-NAG-NAG.4 | Binding site not conserved. | 2-acetamido-2-deoxy-beta-D-glucopyranose-(1-4)-2-acetamido-2-deoxy-beta-D-glucopyranose-(1-4)-2-acetamido-2-deoxy-beta-D-glucopyranose |
| NAG-NAG-NAG.9 | Binding site not conserved. | 2-acetamido-2-deoxy-beta-D-glucopyranose-(1-4)-2-acetamido-2-deoxy-beta-D-glucopyranose-(1-4)-2-acetamido-2-deoxy-beta-D-glucopyranose |
| NAG-NAG-NAG.14 | Binding site not conserved. | 2-acetamido-2-deoxy-beta-D-glucopyranose-(1-4)-2-acetamido-2-deoxy-beta-D-glucopyranose-(1-4)-2-acetamido-2-deoxy-beta-D-glucopyranose |

  

```
Target    MKQVFLSTTTEFKEIDTLEPGTWINLVNPTQNESLEIANTFDIDIADLRAPLDAEEMSRITIEDEYTLIIVDVPVTEERN  
6b7n.1.A  --------------------------------------------------------------------------------  
  
Target    NRTYYVTIPLGIIITEETIITTCLEPLPVLDVFINRRLRNFYTFMRSRFIFQILYRNAELYLTALRSIDRKSEQIESQLH  
6b7n.1.A  --------------------------------------------------------LAESFNQAVGNISLALSSVNDAIQ  
  
Target    QSTRNEELIELMELEKTIVYFKASLKTNERVIKKLTSSTSNIKKYLEDEDLLEDTLIETQQAIEMADIYGNVLHSMTETF  
6b7n.1.A  QTS-----EALNTVAIAIKKIQTVVNQQGEALSHLTAQLSN--NFQAISTSIQDIYNRLEEVEANQQ-------------  
  
Target    ASIISNNQNNIMKTLALVTIVMSIPTMVFSAYGMNFKDNEIPLNGEPNAFWLIVFIAFAMSVSLTLYLIHKKWF  
6b7n.1.A  --------------------------------------------------------------------------  
  
  
Target    MKQVFLSTTTEFKEIDTLEPGTWINLVNPTQNESLEIANTFDIDIADLRAPLDAEEMSRITIEDEYTLIIVDVPVTEERN  
6b7n.1.B  --------------------------------------------------------------------------------  
  
Target    NRTYYVTIPLGIIITEETIITTCLEPLPVLDVFINRRLRNFYTFMRSRFIFQILYRNAELYLTALRSIDRKSEQIESQLH  
6b7n.1.B  --------------------------------------------------------LAESFNQAVGNISLALSSVNDAIQ  
  
Target    QSTRNEELIELMELEKTIVYFKASLKTNERVIKKLTSSTSNIKKYLEDEDLLEDTLIETQQAIEMADIYGNVLHSMTETF  
6b7n.1.B  QTS-----EALNTVAIAIKKIQTVVNQQGEALSHLTAQLSN--NFQAISTSIQDIYNRLEEVEANQQ-------------  
  
Target    ASIISNNQNNIMKTLALVTIVMSIPTMVFSAYGMNFKDNEIPLNGEPNAFWLIVFIAFAMSVSLTLYLIHKKWF  
6b7n.1.B  --------------------------------------------------------------------------  
  
  
Target    MKQVFLSTTTEFKEIDTLEPGTWINLVNPTQNESLEIANTFDIDIADLRAPLDAEEMSRITIEDEYTLIIVDVPVTEERN  
6b7n.1.C  --------------------------------------------------------------------------------  
  
Target    NRTYYVTIPLGIIITEETIITTCLEPLPVLDVFINRRLRNFYTFMRSRFIFQILYRNAELYLTALRSIDRKSEQIESQLH  
6b7n.1.C  --------------------------------------------------------LAESFNQAVGNISLALSSVNDAIQ  
  
Target    QSTRNEELIELMELEKTIVYFKASLKTNERVIKKLTSSTSNIKKYLEDEDLLEDTLIETQQAIEMADIYGNVLHSMTETF  
6b7n.1.C  QTS-----EALNTVAIAIKKIQTVVNQQGEALSHLTAQLSN--NFQAISTSIQDIYNRLEEVEANQQ-------------  
  
Target    ASIISNNQNNIMKTLALVTIVMSIPTMVFSAYGMNFKDNEIPLNGEPNAFWLIVFIAFAMSVSLTLYLIHKKWF  
6b7n.1.C  --------------------------------------------------------------------------
```

  


---

  

## Materials and Methods

## Template Search

Template search with BLAST and HHblits
has been performed against the SWISS-MODEL template library (SMTL, last update: 2021-05-19, last included PDB release: 2021-05-14).

The target sequence was searched with BLAST against the primary amino acid sequence contained in the SMTL.
A total of 5 templates were found.

An initial HHblits profile has been built using the procedure outlined in (Steinegger et al.), followed by 1 iteration of HHblits against Uniclust30 (Mirdita, von den Driesch et al.). The obtained profile has then be searched against all profiles of the SMTL. A total of 335 templates were found.

## Template Selection

For each identified template, the template's quality has been predicted from features of the target-template alignment.
The templates with the highest quality have then been selected for model building.

## Model Building

Models are built based on the target-template alignment using ProMod3 (Studer et al.). Coordinates which are conserved between the target and the template are copied from the template to the model. Insertions and deletions are remodelled using a fragment library. Side chains are then rebuilt. Finally, the geometry of the resulting model is regularized by using a force field.

## Model Quality Estimation

The global and per-residue model quality has been assessed using the QMEAN scoring function (Studer et al.).

## Ligand Modelling

Ligands present in the template structure are transferred by homology to the model when the following criteria are met: (a) The ligands are annotated as biologically relevant in the template library, (b) the ligand is in contact with the model, (c) the ligand is not clashing with the protein, (d) the residues in contact with the ligand are conserved between the target and the template. If any of these four criteria is not satisfied, a certain ligand will not be included in the model. The model summary includes information on why and which ligand has not been included.

## Oligomeric State Conservation

The quaternary structure annotation of the template is used to model the target sequence in its oligomeric form. The method (Bertoni et al.) is based on a supervised machine learning algorithm, Support Vector Machines (SVM), which combines interface conservation, structural clustering, and other template features to provide a quaternary structure quality estimate (QSQE). The QSQE score is a number between 0 and 1, reflecting the expected accuracy of the interchain contacts for a model built based a given alignment and template. Higher numbers indicate higher reliability. This complements the GMQE score which estimates the accuracy of the tertiary structure of the resulting model.

## References

- **BLAST**  
  Camacho, C., Coulouris, G., Avagyan, V., Ma, N., Papadopoulos, J.,
  Bealer, K., Madden, T.L. BLAST+: architecture and applications. BMC
  Bioinformatics 10, 421-430 (2009).
- **HHblits**  
  Steinegger, M., Meier, M., Mirdita, M., Vöhringer, H., Haunsberger,
  S. J., Söding, J. HH-suite3 for fast remote homology detection and
  deep protein annotation. BMC Bioinformatics 20, 473 (2019).
- **Uniclust30**  
  Mirdita, M., von den Driesch, L., Galiez, C., Martin, M.J., Söding,
  J., Steinegger, M. Uniclust databases of clustered and deeply annotated
  protein sequences and alignments. Nucleic Acids Research 45, D170–D176
  (2016).

## Table T1:

Primary amino acid sequence for which templates were searched and models were built.

MKQVFLSTTTEFKEIDTLEPGTWINLVNPTQNESLEIANTFDIDIADLRAPLDAEEMSRITIEDEYTLIIVDVPVTEERNNRTYYVTIPLGIIITEETII  
TTCLEPLPVLDVFINRRLRNFYTFMRSRFIFQILYRNAELYLTALRSIDRKSEQIESQLHQSTRNEELIELMELEKTIVYFKASLKTNERVIKKLTSSTS  
NIKKYLEDEDLLEDTLIETQQAIEMADIYGNVLHSMTETFASIISNNQNNIMKTLALVTIVMSIPTMVFSAYGMNFKDNEIPLNGEPNAFWLIVFIAFAM  
SVSLTLYLIHKKWF

## Table T2:

| Template | Seq Identity | Oligo-state | QSQE | Found by | Method | Resolution | Seq Similarity | Coverage | Description |
| --- | --- | --- | --- | --- | --- | --- | --- | --- | --- |
| 4ev6.1.E | 26.55 | homo-pentamer | 0.54 | HHblits | X-ray | 3.20Å | 0.34 | 0.92 | Magnesium transport protein CorA |
| 4ev6.1.C | 26.55 | homo-pentamer | 0.54 | HHblits | X-ray | 3.20Å | 0.34 | 0.92 | Magnesium transport protein CorA |
| 4ev6.1.A | 26.55 | homo-pentamer | 0.54 | HHblits | X-ray | 3.20Å | 0.34 | 0.92 | Magnesium transport protein CorA |
| 4ev6.1.D | 26.55 | homo-pentamer | 0.54 | HHblits | X-ray | 3.20Å | 0.34 | 0.92 | Magnesium transport protein CorA |
| 4ev6.1.B | 26.55 | homo-pentamer | 0.54 | HHblits | X-ray | 3.20Å | 0.34 | 0.92 | Magnesium transport protein CorA |
| 4eed.1.C | 16.84 | homo-pentamer | 0.54 | HHblits | X-ray | 3.92Å | 0.30 | 0.95 | Magnesium transport protein CorA |
| 3jcf.1.E | 16.78 | homo-pentamer | 0.50 | HHblits | EM | NA | 0.30 | 0.93 | Magnesium transport protein CorA |
| 4eeb.1.A | 16.84 | homo-pentamer | 0.42 | HHblits | X-ray | 3.80Å | 0.30 | 0.95 | Magnesium transport protein CorA |
| 4eeb.1.B | 16.84 | homo-pentamer | 0.42 | HHblits | X-ray | 3.80Å | 0.30 | 0.95 | Magnesium transport protein CorA |
| 2hn2.1.C | 16.78 | homo-pentamer | 0.48 | HHblits | X-ray | 3.70Å | 0.30 | 0.93 | Magnesium transport protein corA |
| 2bbj.1.A | 16.78 | homo-pentamer | 0.44 | HHblits | X-ray | 3.90Å | 0.30 | 0.93 | divalent cation transport-related protein |
| 4ev6.1.C | 31.47 | homo-pentamer | 0.41 | BLAST | X-ray | 3.20Å | 0.36 | 0.80 | Magnesium transport protein CorA |
| 4ev6.1.E | 31.47 | homo-pentamer | 0.41 | BLAST | X-ray | 3.20Å | 0.36 | 0.80 | Magnesium transport protein CorA |
| 4ev6.1.A | 31.47 | homo-pentamer | 0.41 | BLAST | X-ray | 3.20Å | 0.36 | 0.80 | Magnesium transport protein CorA |
| 4ev6.1.D | 31.47 | homo-pentamer | 0.41 | BLAST | X-ray | 3.20Å | 0.36 | 0.80 | Magnesium transport protein CorA |
| 4ev6.1.B | 31.47 | homo-pentamer | 0.41 | BLAST | X-ray | 3.20Å | 0.36 | 0.80 | Magnesium transport protein CorA |
| 2iub.1.C | 16.78 | homo-pentamer | 0.32 | HHblits | X-ray | 2.90Å | 0.30 | 0.93 | DIVALENT CATION TRANSPORT-RELATED PROTEIN |
| 2iub.1.A | 16.78 | homo-pentamer | 0.32 | HHblits | X-ray | 2.90Å | 0.30 | 0.93 | DIVALENT CATION TRANSPORT-RELATED PROTEIN |
| 5n9y.1.B | 14.43 | homo-pentamer | 0.30 | HHblits | EM | NA | 0.27 | 0.93 | Zinc transport protein ZntB |
| 5n9y.1.C | 14.43 | homo-pentamer | 0.30 | HHblits | EM | NA | 0.27 | 0.93 | Zinc transport protein ZntB |
| 5n9y.1.D | 14.43 | homo-pentamer | 0.30 | HHblits | EM | NA | 0.27 | 0.93 | Zinc transport protein ZntB |
| 5n9y.1.A | 14.43 | homo-pentamer | 0.30 | HHblits | EM | NA | 0.27 | 0.93 | Zinc transport protein ZntB |
| 5n9y.1.E | 14.43 | homo-pentamer | 0.30 | HHblits | EM | NA | 0.27 | 0.93 | Zinc transport protein ZntB |
| 5n77.1.A | 16.19 | homo-pentamer | 0.30 | HHblits | X-ray | 2.80Å | 0.28 | 0.79 | Magnesium transport protein CorA |
| 4egw.1.B | 26.41 | homo-dimer | 0.06 | HHblits | X-ray | 2.50Å | 0.33 | 0.74 | Magnesium transport protein CorA |
| 4egw.1.A | 26.41 | homo-dimer | 0.06 | HHblits | X-ray | 2.50Å | 0.33 | 0.74 | Magnesium transport protein CorA |
| 3ck6.1.E | 12.55 | homo-pentamer | 0.14 | HHblits | X-ray | 1.90Å | 0.26 | 0.76 | Putative membrane transport protein |
| 3ck6.1.B | 12.55 | homo-pentamer | 0.14 | HHblits | X-ray | 1.90Å | 0.26 | 0.76 | Putative membrane transport protein |
| 3ck6.1.A | 12.55 | homo-pentamer | 0.14 | HHblits | X-ray | 1.90Å | 0.26 | 0.76 | Putative membrane transport protein |
| 3nwi.1.A | 12.50 | homo-pentamer | 0.10 | HHblits | X-ray | 3.13Å | 0.26 | 0.71 | Zinc transport protein zntB |
| 3nvo.1.A | 12.44 | homo-dimer | 0.02 | HHblits | X-ray | 2.30Å | 0.26 | 0.72 | Zinc transport protein zntB |
| 2hn1.1.A | 15.50 | homo-dimer | 0.25 | HHblits | X-ray | 2.90Å | 0.28 | 0.64 | Magnesium and cobalt transporter |
| 6v1v.1.C | 15.56 | homo-tetramer | - | HHblits | X-ray | 3.19Å | 0.28 | 0.29 | Vegetative insecticidal protein |
| 6v1v.1.D | 15.56 | homo-tetramer | - | HHblits | X-ray | 3.19Å | 0.28 | 0.29 | Vegetative insecticidal protein |
| 6yrf.1.A | 15.56 | homo-tetramer | - | HHblits | EM | NA | 0.28 | 0.29 | Vegetative insecticidal protein |
| 6v1v.1.A | 15.56 | homo-tetramer | - | HHblits | X-ray | 3.19Å | 0.28 | 0.29 | Vegetative insecticidal protein |
| 6yrf.1.B | 15.56 | homo-tetramer | - | HHblits | EM | NA | 0.28 | 0.29 | Vegetative insecticidal protein |
| 6b7n.1.A | 14.29 | homo-trimer | - | HHblits | EM | NA | 0.28 | 0.27 | Spike protein |
| 5aqt.1.B | 9.84 | monomer | - | HHblits | X-ray | 1.90Å | 0.25 | 0.19 | BAG FAMILY MOLECULAR CHAPERONE REGULATOR 1 |
| 1t7s.1.A | 13.64 | monomer | - | HHblits | X-ray | 2.80Å | 0.28 | 0.21 | BAG-1 cochaperone |
| 1t7s.2.A | 13.64 | monomer | - | HHblits | X-ray | 2.80Å | 0.28 | 0.21 | BAG-1 cochaperone |
| 5aqf.2.B | 9.84 | monomer | - | HHblits | X-ray | 1.88Å | 0.25 | 0.19 | BAG FAMILY MOLECULAR CHAPERONE REGULATOR 1 |
| 5aqf.1.B | 9.84 | monomer | - | HHblits | X-ray | 1.88Å | 0.25 | 0.19 | BAG FAMILY MOLECULAR CHAPERONE REGULATOR 1 |
| 2ncj.1.A | 11.86 | monomer | - | HHblits | NMR | NA | 0.28 | 0.19 | Uncharacterized protein |
| 1hx1.1.B | 10.00 | monomer | - | HHblits | X-ray | 1.90Å | 0.25 | 0.19 | BAG family molecular chaperone regulator 1 |
| 3fzh.2.A | 10.00 | monomer | - | HHblits | X-ray | 2.00Å | 0.25 | 0.19 | BAG family molecular chaperone regulator 1 |
| 1quu.1.A | 16.95 | homo-dimer | - | HHblits | X-ray | 2.50Å | 0.28 | 0.19 | HUMAN SKELETAL MUSCLE ALPHA-ACTININ 2 |
| 6yrg.1.A | 15.56 | homo-tetramer | - | HHblits | EM | 7.00Å | 0.28 | 0.29 | Vegetative insecticidal protein |
| 5aqg.2.B | 9.84 | monomer | - | HHblits | X-ray | 2.24Å | 0.25 | 0.19 | BAG FAMILY MOLECULAR CHAPERONE REGULATOR 1 |
| 6yrg.1.B | 15.56 | homo-tetramer | - | HHblits | EM | 7.00Å | 0.28 | 0.29 | Vegetative insecticidal protein |

  
The table above shows the top 50 filtered templates. A further 240 templates were found which were considered to be less suitable for modelling than the filtered list.  
1i6z.1.A, 1lvf.1.A, 1lvf.2.A, 1u4q.1.A, 1u4q.2.A, 1zbt.1.A, 2bbh.1.A, 2c5i.1.B, 2iak.1.A, 2mic.1.A, 2mic.1.B, 2mjo.1.A, 2mjo.1.B, 2osz.1.C, 2osz.1.D, 2wz7.1.A, 2wz7.1.B, 2wz7.1.C, 2wz7.2.A, 2wz7.2.B, 2wz7.2.C, 2xdj.1.A, 2xdj.1.B, 2xdj.1.C, 2xdj.2.A, 2xdj.2.B, 2xdj.2.C, 2xzr.1.A, 2zjs.1.A, 2zqp.1.A, 3dl8.1.B, 3ghg.1.B, 3ghg.1.E, 3ghg.2.B, 3ghg.2.E, 3jc8.42.A, 3jc8.43.A, 3jcg.1.A, 3jcg.1.B, 3jcg.1.C, 3jcg.1.D, 3jcg.1.E, 3jch.1.A, 3jch.1.B, 3jch.1.C, 3jch.1.D, 3mk7.1.A, 3okq.1.A, 3onx.1.A, 3onx.1.B, 3pdy.1.A, 3pdy.2.A, 3pjz.1.A, 3rkg.1.A, 3stq.1.A, 3stq.1.B, 3stq.2.A, 3stq.2.B, 3stq.3.A, 3stq.4.A, 3t98.1.B, 3vpv.1.A, 3vpv.1.B, 3x29.1.A, 3zsu.1.A, 4eij.1.A, 4eij.1.B, 4h63.1.B, 4i0u.1.A, 4i0u.1.B, 4i0u.1.C, 4i0u.1.D, 4i0u.1.E, 4i0u.2.A, 4i0u.2.B, 4i0u.2.C, 4i0u.2.D, 4i0u.2.E, 4j9u.1.A, 4jo7.1.C, 4jo7.1.D, 4jo7.2.A, 4jo7.2.C, 4jo9.1.B, 4jpr.1.A, 4jq5.1.A, 4jq5.1.B, 4jq5.1.C, 4jq5.2.A, 4jq5.2.B, 4jq5.2.C, 4jq5.3.A, 4n21.1.A, 4n21.2.B, 4p79.1.A, 4v67.1.Y, 4xng.1.A, 4xng.1.B, 5aww.1.A, 5b2g.1.A, 5b2g.2.A, 5b2g.3.A, 5b2g.4.A, 5c3l.1.A, 5ch4.1.A, 5do7.1.A, 5do7.1.B, 5do7.2.A, 5do7.2.B, 5ijh.1.A, 5ijn.1.F, 5ijn.1.G, 5ijn.1.H, 5ijn.1.L, 5ijn.1.R, 5j0i.1.A, 5j1g.1.A, 5j1i.1.A, 5jrw.1.A, 5jrw.1.B, 5jrw.1.C, 5jrw.1.D, 5jtg.1.A, 5jtg.1.B, 5jtg.1.C, 5jtg.1.D, 5kte.1.A, 5kuc.1.A, 5mdy.1.G, 5n9j.1.J, 5u0p.1.C, 5u0s.1.B, 5v2s.1.A, 5xei.1.A, 5zgg.1.A, 5zgg.1.B, 5zuv.1.A, 5zuv.1.B, 5zuv.1.C, 6adq.1.D, 6ake.1.A, 6ake.2.A, 6akf.1.A, 6akf.2.A, 6akf.3.A, 6akf.4.A, 6akg.1.A, 6bm8.1.A, 6btm.1.C, 6c5l.1.Y, 6c5l.2.Y, 6d9w.1.A, 6dfp.1.A, 6dlc.1.A, 6dnc.1.c, 6eti.1.A, 6f0k.1.C, 6gap.1.A, 6gap.1.B, 6gap.1.C, 6gxn.1.r, 6hbu.1.A, 6hij.1.A, 6hwh.1.K, 6lod.1.C, 6m3p.1.B, 6m3q.1.B, 6m3r.1.B, 6n9h.1.A, 6naf.1.A, 6ncn.1.A, 6nxm.1.A, 6ny8.1.A, 6nyi.1.A, 6nyi.1.B, 6nyi.1.C, 6nyk.1.A, 6nz1.1.A, 6nz1.1.B, 6nz1.1.C, 6nz3.1.A, 6o0c.1.A, 6o0i.1.A, 6ov2.1.A, 6ov3.1.A, 6qkc.1.E, 6r17.1.A, 6r17.1.B, 6s7o.1.F, 6s7t.1.F, 6tpi.1.A, 6tqe.1.A, 6tqf.1.A, 6v4j.1.B, 6v4j.1.D, 6v4k.1.A, 6v4k.1.B, 6v4k.1.C, 6v4k.1.D, 6v4l.1.E, 6vag.1.A, 6vag.1.B, 6vja.1.A, 6vxf.1.B, 6vxh.1.B, 6vxi.1.A, 6xns.1.A, 6xns.1.B, 6xns.1.C, 6xns.2.A, 6xns.2.B, 6xns.2.C, 6xp5.1.I, 6y97.1.A, 6y97.1.B, 6y9a.1.A, 6y9a.1.B, 6ye4.1.A, 6yqf.1.A, 6yqf.1.B, 6ysl.1.A, 6ysl.1.D, 6ysl.1.E, 6ysl.1.F, 6ysl.1.G, 6z5s.1.A, 6znl.1.O, 6znl.1.V, 6zyw.1.B, 6zyw.1.C, 6zz6.1.B, 7jr7.1.A, 7jr7.1.B, 7kdp.1.A, 7kp4.1.A, 7kzm.1.Q, 7neq.1.A, 7neq.1.F, 7nez.1.A, 7nfd.1.F

Swiss Institute of Bioinformatics
Contact Us
