# Supplementary figures and images for: A bacterial pan-genome makes gene essentiality strain-dependent and evolvable
Source: Nat Microbiol. 2022 Sep 12;7(10):1580–92. doi: 10.1038/s41564-022-01208-7 (PMC9519441; doi:10.1038/s41564-022-01208-7)

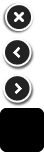

Supplement: Source Data Fig. 4 — Swiss models data, genetic interactions (a subset of Supplementary Data 11), growth curves data and RNA-seq data for the two transporters (taken from Supplementary Data 6). [file 41564_2022_1208_MOESM8_ESM.zip › Fig_4a_models/SP_1751_swissmodel/web/js/fancybox_sprite.png]

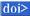

Supplement: Source Data Fig. 4 — Swiss models data, genetic interactions (a subset of Supplementary Data 11), growth curves data and RNA-seq data for the two transporters (taken from Supplementary Data 6). [file 41564_2022_1208_MOESM8_ESM.zip › Fig_4a_models/SP_1751_swissmodel/web/images/doi.jpg]

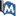

Supplement: Source Data Fig. 4 — Swiss models data, genetic interactions (a subset of Supplementary Data 11), growth curves data and RNA-seq data for the two transporters (taken from Supplementary Data 6). [file 41564_2022_1208_MOESM8_ESM.zip › Fig_4a_models/SP_1751_swissmodel/web/images/pubmed.png]

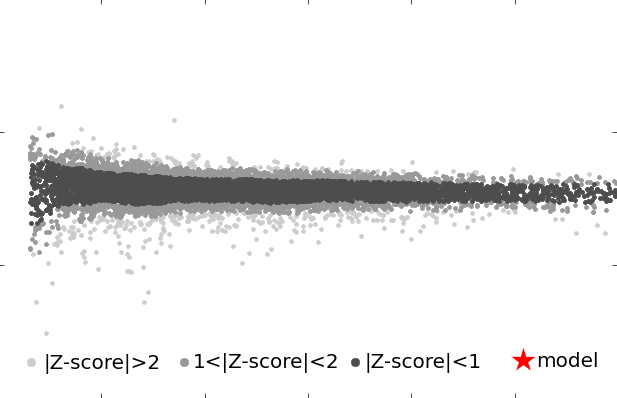

Supplement: Source Data Fig. 4 — Swiss models data, genetic interactions (a subset of Supplementary Data 11), growth curves data and RNA-seq data for the two transporters (taken from Supplementary Data 6). [file 41564_2022_1208_MOESM8_ESM.zip › Fig_4a_models/SP_1751_swissmodel/web/images/qmean_norm.png]

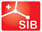

Supplement: Source Data Fig. 4 — Swiss models data, genetic interactions (a subset of Supplementary Data 11), growth curves data and RNA-seq data for the two transporters (taken from Supplementary Data 6). [file 41564_2022_1208_MOESM8_ESM.zip › Fig_4a_models/SP_1751_swissmodel/web/images/sib_logo_small.png]

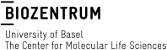

Supplement: Source Data Fig. 4 — Swiss models data, genetic interactions (a subset of Supplementary Data 11), growth curves data and RNA-seq data for the two transporters (taken from Supplementary Data 6). [file 41564_2022_1208_MOESM8_ESM.zip › Fig_4a_models/SP_1751_swissmodel/web/images/biozentrum_en.png]

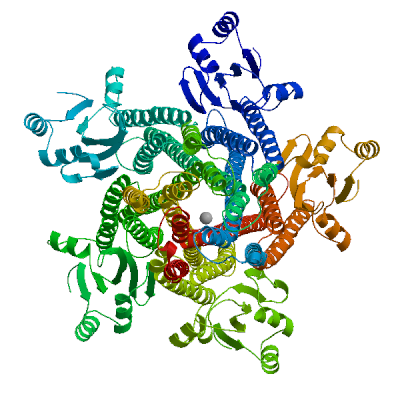

Supplement: Source Data Fig. 4 — Swiss models data, genetic interactions (a subset of Supplementary Data 11), growth curves data and RNA-seq data for the two transporters (taken from Supplementary Data 6). [file 41564_2022_1208_MOESM8_ESM.zip › Fig_4a_models/SP_1751_swissmodel/SP_1751/models/01/01.png]

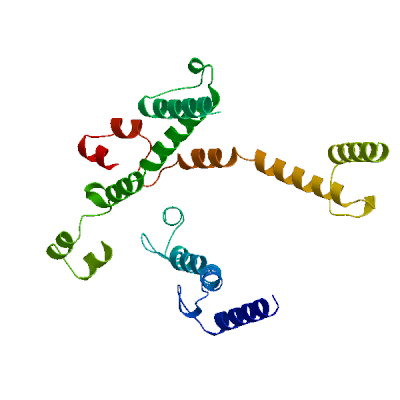

Supplement: Source Data Fig. 4 — Swiss models data, genetic interactions (a subset of Supplementary Data 11), growth curves data and RNA-seq data for the two transporters (taken from Supplementary Data 6). [file 41564_2022_1208_MOESM8_ESM.zip › Fig_4a_models/SP_0185_swissmodel/SP_0185/models/02/02.png]

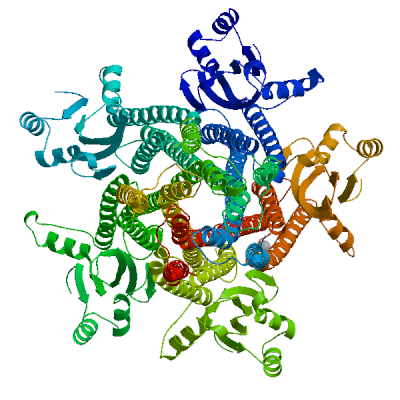

Supplement: Source Data Fig. 4 — Swiss models data, genetic interactions (a subset of Supplementary Data 11), growth curves data and RNA-seq data for the two transporters (taken from Supplementary Data 6). [file 41564_2022_1208_MOESM8_ESM.zip › Fig_4a_models/SP_0185_swissmodel/SP_0185/models/01/01.png]

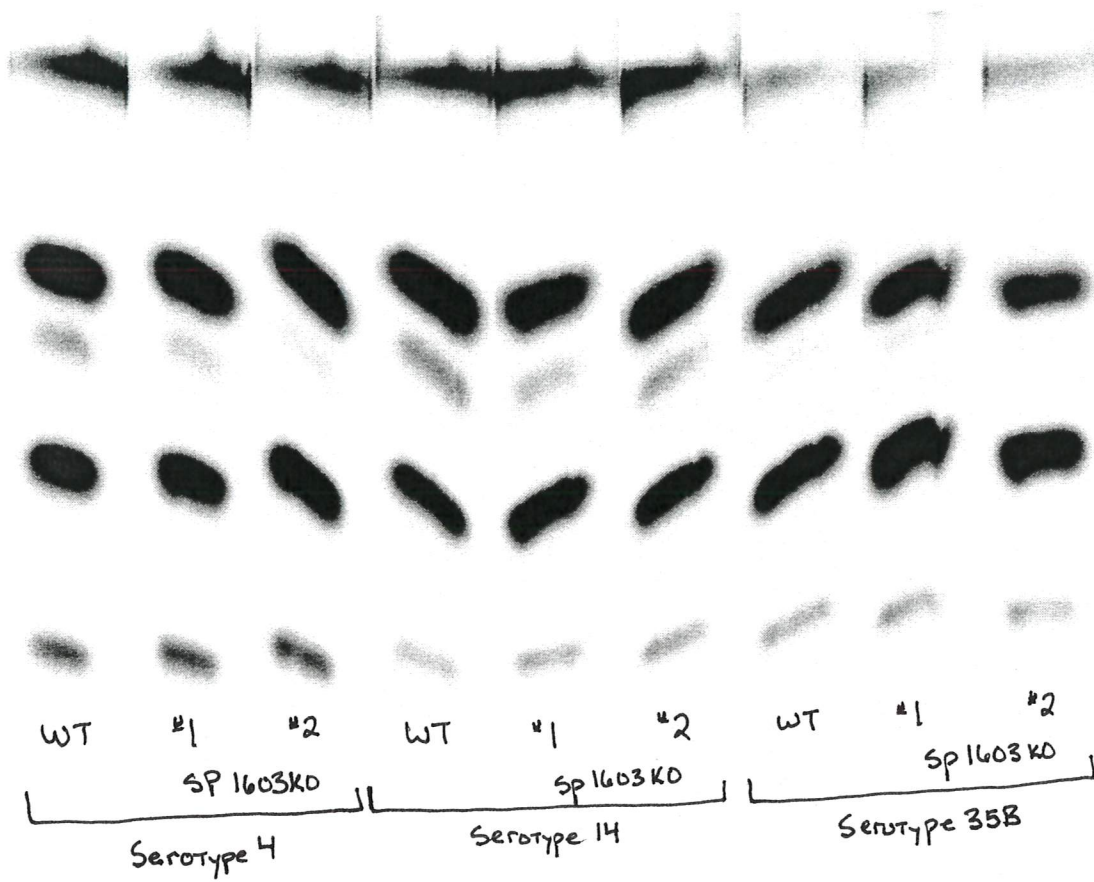

Supplement: Source Data Extended Data Fig. 4 — Growth curves data. Acetate-labelling TLC blot. [file 41564_2022_1208_MOESM14_ESM.zip › TLC_blot.pdf]
